# Stereoselective synthesis of atropisomeric amides enabled by intramolecular acyl transfer†

**DOI:** 10.1039/d4sc05760k

**Published:** 2025-01-23

**Authors:** Jack M. Wootton, Natalie J. Roper, Catrin E. Morris, Victoria E. Maguire, Lee C. Duff, Paul G. Waddell, Adrian C. Whitwood, Richard J. Gammons, Afjal H. Miah, Jason M. Lynam, Roly J. Armstrong, William P. Unsworth

**Affiliations:** a Department of Chemistry, University of York York YO10 5DD UK william.unsworth@york.ac.uk; b School of Natural and Environmental Sciences, Newcastle University Newcastle Upon Tyne NE1 7RU UK roly.armstrong@newcastle.ac.uk; c GSK Gunnels Wood Rd Stevenage SG1 2NY UK

## Abstract

C–N atropisomeric amides are important compounds in medicinal chemistry and agrochemistry. Atropselective methods for their synthesis are therefore important. In this study, a novel strategy to make C–N atropisomeric amides based on intramolecular acyl transfer *via* a tethered Lewis basic pyridine or tertiary amine group is reported. The reactions operate under kinetic control and in most cases are highly atropselective, with the products isolable as pure, single diastereoisomers following chromatography. The kinetically favored atropisomer can also be isomerised into the alternative thermodynamically favored atropisomer upon heating. The kinetic and thermodynamic outcomes are supported by computational studies, while additional mechanistic studies support operation *via* initial fast acylation of the Lewis basic group, followed by rate-determining acyl transfer, which also enables control over the atropisomer formed.

## Abstract 2

A stereoselective strategy to make C–N atropisomeric amides based on intramolecular acyl transfer *via* a tethered Lewis basic pyridine or tertiary amine group is reported.

## Graphical abstract

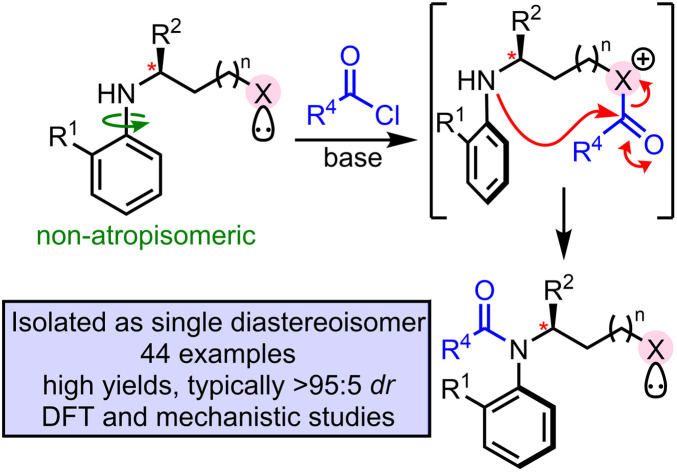

## Introduction

Molecular rearrangement reactions that operate *via* cascade processes are of great importance in synthetic chemistry.^1^ In large part, this is due to their ability to generate complex organic architectures with high efficiency from comparatively simple precursors, often *via* non-intuitive retrosynthetic disconnections. Bond formation *via* rearrangement can also bring additional synthetic advantages, notably including the control of stereochemistry.^2,3^

**Scheme 1:**
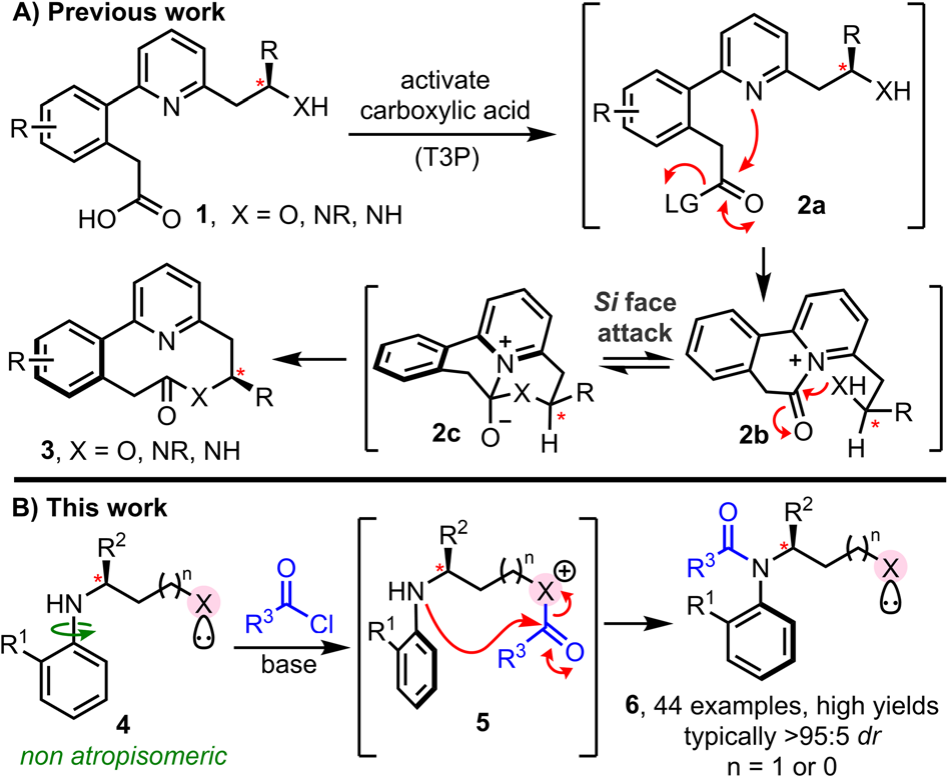
(A) Previous work: atropselective medium-sized ring synthesis enabled *via* point-to-axial chiral transfer; (B) this work: atropselective amide synthesis enabled by intramolecular acyl transfer.

The cascade ring expansion method^4–7^ summarised in Scheme 1A, recently developed by our York group, is one such example.^4*a*^ When this method was conceived, the primary focus was to develop a general strategy for the synthesis of medium-sized rings that does not require high-dilution reaction conditions.^8^ Reaction *via* a ring expansion cascade enabled this to be achieved, by splitting the inefficient end-to-end cyclisation into two more kinetically favourable steps; thus, a cyclisation (1 → 2a → 2b) and ring expansion cascade (2b → 2c → 3) enabled medium-sized lactones and lactams to be prepared in high yields, at 0.1 M concentration. The cascade proceeding solely *via* 6-membered ring cyclisation steps is key to the kinetic improvements in this cyclisation reaction. Furthermore, the same feature also enabled biaryl containing linear starting materials 1 to undergo cyclisation to form medium-sized ring lactones/lactams 3 as single atropisomers, with the point stereogenic centre (labelled*) enabling control of the axial chirality of the biaryl unit in the product;^9,10^ the stereogenic centre present in 1 is able to direct the facial selectivity of nucleophilic attack at prochiral acyl ammonium intermediate 2b. Si face attack is proposed to be favoured, due to the preference for the *R* group of the labelled stereogenic centre to adopt a pseudo-equatorial orientation during the key cyclisation step (2b → 2c). This concept was later extended to longer cascade ring expansion methods, with the same high level of atropselectivity observed. Our aim in this study was to establish whether the concept of intramolecular acyl transfer can be used much more broadly to facilitate the stereoselective synthesis of C–N atropisomeric amides, an emerging class of chiral materials with significant potential in pharmaceuticals and agriscience.^11^ A variety of elegant methods have been reported for the synthesis of such materials, including stereoselective *N*-alkylation, amination, annulation and C–H activation.^12^ However, while *N*-acylation is the most widespread approach for conventional amide synthesis, it has scarcely been applied to prepare C–N atropisomeric amides. To the best of our knowledge, the only examples reported to date involve acylation of acidified nitrogen nucleophiles, for example, in elegant studies reported by Lu, Zhao, Dong and Miller involving acylation of sulfonamides and carboxamides.^13^ In contrast, methods for the direct atropselective acylation of unactivated anilines are undeveloped.^14^ The major challenge here is that atropisomeric systems are by definition sterically congested, and hence achieving efficient acylation whilst simultaneously controlling the configuration of a new stereogenic axis is extremely challenging.

With these points in mind, we set out to design a new method for the stereoselective synthesis of atropisomeric amides enabled by intramolecular acyl transfer. Our reaction design is summarised in Scheme 1B. We proposed that pro-atropisomeric secondary anilines substituted with a Lewis-basic activating group (*i.e.*X in 4) could promote amide bond formation *via* an initial acylation of the more nucleophilic site (4 → 5), followed by intramolecular acyl transfer onto the aniline (5 → 6). In such a design the activating group should play two key roles: (1) act an internal nucleophilic catalyst to accelerate aniline *N*-acylation intramolecularly; (2) deliver the acyl group to the aniline stereoselectively. For both points to succeed, ensuring that acyl transfer proceeds *via* a 5- or 6-membered cyclic transition state is a key design feature, as this should help to accelerate the key acyl transfer step, and enable a point stereogenic centre (labelled*) adjacent to the Lewis base to impart control over the atropisomer formed.

The successful realisation of this idea is reported herein. In total, 44 C–N atropisomeric amides have been prepared using this concept, most in high yield. The products obtained all contain two chiral elements, with the stereoselective synthesis of compounds containing multiple chiral elements of much current interest.^15^ Atropselectivity is high (typically >95 : 5 dr) and the products are isolable as single diastereoisomers in all cases. Mechanistic and computational studies support our proposed acyl transfer mechanism, with the reactions thought to operate under kinetic control. Isomerisation (*via* C–N bond rotation) to allow formation of the thermodynamically favoured atropisomer is possible upon heating.

## Results and discussion

**Scheme 2:**
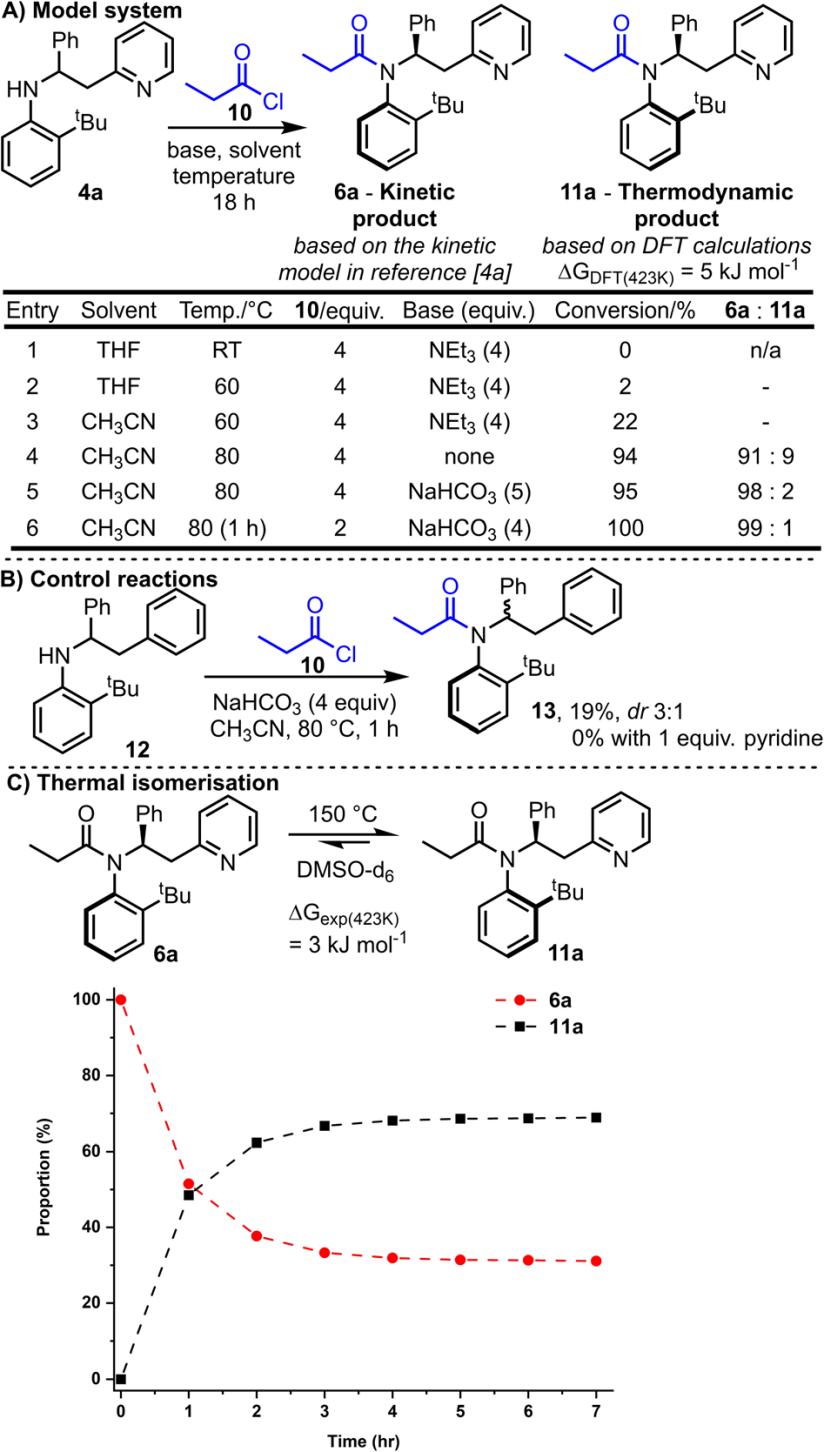
Optimisation of the synthesis (A), control reactions (B) and isomerisation studies for our model substrate, amide 6a (C). In (C), the dashed lines are shown as a guide to the eye.

We started by exploring model substrate 4a, which contains a 2-pyridyl group as the Lewis-basic activator. Before embarking on synthetic work, computational studies were performed to examine the energy of the two atropisomers potentially accessible from the *N*-acylation of 4a with propionyl chloride 10 (Scheme 2A). Thus, the relative Gibbs free energy of atropisomers 6a and 11a were calculated using Density Functional Theory (DFT, B3LYP/6-31G*)^4*a*^ and atropisomer 11a was calculated to be lower in energy by 5 kJ mol^−1^. This relatively small energy difference suggests that a mixture of atropisomers 6a and 11a would likely be obtained if the reaction operates under thermodynamic control. Intriguingly, this predicted outcome differs to that expected if the kinetic model used to explain the stereoselectivity in our analogous ring expansion system^4*a*^ is applicable here; this kinetic model predicts the formation of isomer 6a.

Synthetic studies commenced by reacting racemic aniline 4a and propionyl chloride 10 in the presence of base. Conversion into products 6a and 11a and their ratio was measured by ^1^H NMR analysis of the unpurified reaction mixture. Selected reaction optimisation results are summarised in the Scheme 2A, with full details included in the ESI.†

No conversion was observed when the reaction was performed at RT (entry 1), but upon increasing the temperature to 80 °C (entries 4–6) good conversion into the desired *N*-acylated products 6a and 11a was achieved. Optimal conditions were found based upon heating for 1 h at 80 °C with solid NaHCO_3_ (4 equivalents) and 2 equivalents of propionyl chloride 10 (entry 6). These conditions enabled full conversion, and the formation of a ≈99 : 1 mixture of atropisomers, with isomer 6a (the predicted kinetic product) being the major product formed.^16^

Note that the ratio of acid chloride to base has a significant influence on the reaction conversion; for example, higher conversion was observed using the optimised conditions (entry 6) compared with the entry 5 conditions, despite fewer equivalents of acid chloride being used and shorter reaction time.

The importance of the pyridine nitrogen is demonstrated by the control reactions summarised in Scheme 2B. Thus, an alternative aniline substrate 12 was prepared, analogous to the model substrate 4a but lacking the pyridine nitrogen group (N is replaced by CH in 12). When this aniline 12 was reacted under the optimised reaction conditions, only 19% conversion into 13 was observed (compared with 100% conversion for 4a), and the acylated product 13 was obtained with a much-reduced dr (3 : 1, compared with 99 : 1 for 4a). To probe whether merely the presence of pyridine in the reaction aids the reaction, for example by acting as a nucleophilic catalyst, the same control reaction was repeated with the additional inclusion of 1 equivalent of pyridine in the reaction. In this case, no conversion of 12 was observed; surprisingly, the exogenous pyridine inhibited, rather than catalysed, the acylation of aniline 12.

With the major atropisomer 6a being the predicted kinetic product, we postulated that heating 6a might allow isomerisation into atropisomer 11a based on the DFT calculations. Heating a pure sample of amide 6a at 80 °C for 18 h in acetonitrile resulted in no change in the dr; this suggests that 6a is configurationally stable under the reaction conditions, in line with the barriers for C–N rotation in related systems.^17^ Isomerisation of 6a could be achieved with more vigorous heating however. Thus, heating a *d*_6_-DMSO solution of pure 6a at 150 °C resulted in partial isomerisation into a roughly 2 : 1 mixture of 11a : 6a (Scheme 2C).^18^ After ≈4 h, an equilibrium appeared to have been established between the two isomers, in favour of 11a. The 2 : 1 equilibrium ratio can be extrapolated into a Gibbs free energy difference of ≈3 kJ mol^−1^ between 6a and 11a at 150 °C, supporting the small difference (5 kJ mol^−1^) calculated using DFT. A relatively high activation barrier to Ar–N rotation was determined from this experimental data (6a → 11a = 134.7 kJ mol^−1^, see ESI† for details), attesting to the very high configurational stability of the amides at RT.

**Scheme 3:**
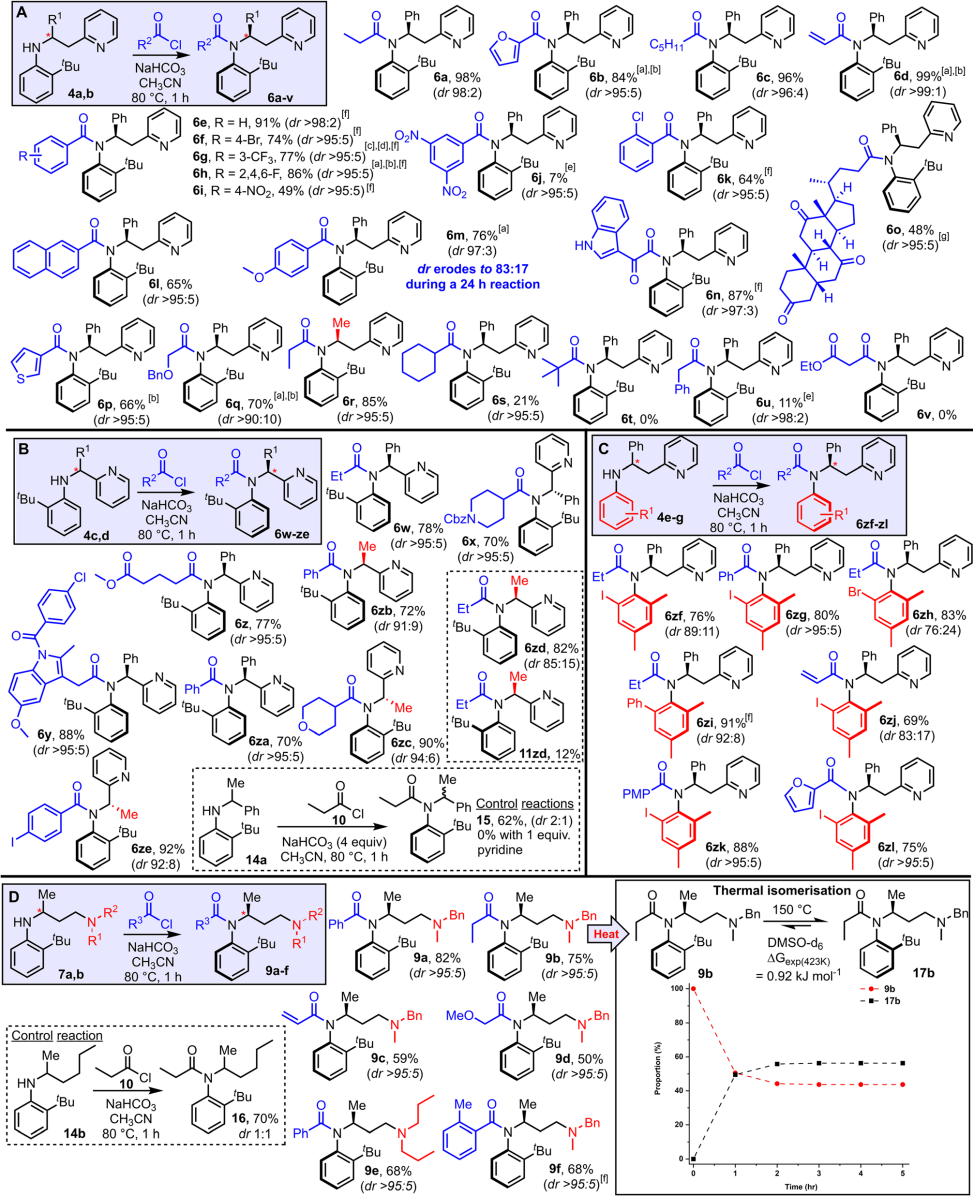
Reaction scope (A–D). Reactions were performed by heating the aniline (1 equiv.), acid chloride (2 equiv.), NaHCO_3_ (4 equiv.) in CH_3_CN for 1 h at 80 °C unless stated. ^*a*^3 equiv. acid chloride used. ^*b*^2 h rection time. ^*c*^4 equiv. acid chloride used. ^*d*^3 h reaction time. ^*e*^24 h reaction time. ^*f*^As a mixture of rotamers (amide bond), see ESI† for details. ^*g*^In this case >95 : 5 dr refers to the atropselectivity of the atropisomeric amide portion only; the product is a 1 : 1 mixture of diastereomers with respect to the steroid portion. (D) The dashed lines are shown as a guide to the eye. X-ray crystallographic data were obtained to support the assignments for products 6b, 6e, 6w, 6zf, 6zi, and 9a.

Attention next turned to examining the scope of this new method for the stereoselective synthesis of atropisomeric amides (Scheme 3). The scope of the method with respect to different acyl groups was tested first, by reacting aniline 4a with various acid chlorides under the standard conditions. Highly atropselective *N*-acylation was observed in the majority of cases tested, enabling the isolation of a wide array of functionalised amides (6a–q, Scheme 3A) as single atropisomers in high yields. Note that in all examples featured in Scheme 3, the dr quoted relates to the dr of the unpurified reaction mixture, and unless stated, the yields refer to the isolated yield of the major atropisomer shown; in most cases separation of the major and minor isomers was straightforward using standard silica-based flash column chromatography. The assignment of the relative stereochemistry is supported by X-ray crystallographic studies, with an X-ray crystal structure having been obtained for products (6b, 6e, 6w, 6zf and 6zi, 9a) which includes at least one example in each substrate class featured in Schemes 3A–D. Broad functional group compatibility was demonstrated, with successful atropselective examples including products generated from alkyl (6a, 6c), aryl (6e–k), heteroaryl (6b, 6n, 6p), naphthyl (6l), ether (6q) and steroidal (6o) acyl chlorides, all isolated as single atropisomers and with at least 90 : 10 dr in the unpurified reaction mixture. Replacing the phenyl substituent at the stereogenic center with a methyl group was also well tolerated, both in terms of yield and dr (6r). Substrates that performed less well in terms of reaction conversion were those using a very electron-poor acid chloride (6j), sterically demanding acid chlorides (6s, t) and acid chlorides with acidic methylene groups 6u, v); although notably in these cases in which low conversion was observed, the atropselectivity remained high. In most substrates, the chiral axis appears to be configurationally stable under the reaction conditions and hence the reactions are thought to be under kinetic control. However, products 6m represents a notable exception. In this case, some erosion of the dr (97 : 3 → 83 : 17) was observed upon increasing the reaction time from 1 h to 24 h, which enabled the minor atropisomer (11m, see ESI†) to be isolated in 17% yield using the longer reaction time. Electron-rich acyl substituents are known to lower the barrier to Ar–N rotation in related amide systems, explaining the anomalous behavior in this case.^19^

The effect of shortening the tether length between the aniline and pyridine groups was examined next, through *N*-acylation of anilines 4c and 4d (Scheme 3B). In these cases, acyl transfer *via* a 5-membered cyclic transition state, rather than 6-membered, is required, and pleasingly the strategy was similarly effective. Thus, amides 6w–ze were all obtained in good yields. The dr in the unpurified mixture were high in all cases, with the lowest dr being the 85 : 15 ratio observed for 6zd; in this case, both isomers 6zd and 11zd were isolated following column chromatography in yields in line with this ratio. A control reaction was also performed for this system, with aniline 14a (*cf.*4d, with pyridine replaced by phenyl), with a drop in yield (62%) and a marked reduction in dr (2 : 1) observed, again attesting to the importance of the Lewis basic pyridine group (Scheme 3B box). The results summarised in Scheme 3C show that other substituted anilines able to impart atropisomerism following *N*-acylation are also compatible with this approach. Thus, 2,4,6-trisubstituted aniline derivatives with iodide (6zf–zg, 6zj–zl), bromide (6zh) and phenyl (6zi) substituents were all obtained in good yields and with high atropselectivity in most cases tested.

Finally, the results in Scheme 3D show that the Lewis basic group does not need to be pyridine, and that the concept can be extended to aliphatic tertiary amines. Thus, anilines 7a and 7b were reacted under the standard reaction conditions and converted into amides 9a–f as single atropisomers in all cases. As before, a control substrate lacking the Lewis basic amine group (14b) was reacted under the same conditions, and while *N*-acylation took place to afford amide 16 in 70% yield, it was obtained as a 1 : 1 mixture of atropisomers. As for the pyridine-containing systems, it is likely that these reactions are also under kinetic control. To explore this notion, DFT calculations were performed to calculate the relative Gibbs free energies of amide 9b and its atropisomer 17b, using the same computational methodology described earlier. These calculations reveal that the observed isomer 9b lies 2 kJ mol^−1^ higher in energy than 17b, which means at thermodynamic equilibrium both species would be present in similar amounts. Based on these data, partial isomerism to the thermodynamic distribution of products should be possible, and indeed heating a sample of pure 9b to 150 °C in DMSO-*d*_6_ allowed smooth isomerisation into a 58 : 42 mixture of 17b : 9b, with equilibrium reached after around 2 h.

**Scheme 4:**
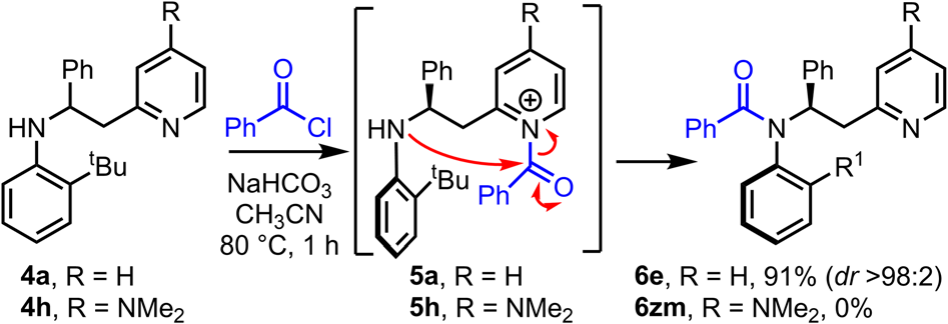
A comparison of the efficiency of acyl transfer for substituted pyridines.

Having established that the method has broad scope, we then moved onto better understanding the acyl transfer mechanism which underpins the observed atropselectivity. As the pyridine group in our model system 4a is proposed to act as a nucleophilic catalyst, one might expect that the installation of an electron donating 4-NMe_2_ group onto the pyridine would lead to enhanced Lewis-basicity, thereby improving the rate of reaction – akin to the rate enhancement observed in intermolecular acylation reactions catalyzed by the well-known nucleophilic catalyst, 4-dimethylaminopyridine (DMAP) (Scheme 4). However, while model system 4a, which has an unsubstituted pyridine group, undergoes atropselective acylation in high yield and dr to form 6e using the standard protocol, the analogous DMAP-like substrate 4h failed to afford the expected amide product 6zm. Acylation of the DMAP-like pyridine moiety (4h → 5h) was expected to occur quickly under the reaction conditions, therefore, this suggested that failure of the acyl transfer step was responsible for this outcome. This is presumably a result of the 4-NMe_2_ substituted *N*-acyl pyridinium intermediate 5h being more stable, and hence less reactive, than its unsubstituted analogue 5a. Evidence in support of this notion was obtained by ^1^H NMR, in which observation of signals consistent with an analogue of *N*-acyl pyridinium intermediate 5h were observed (see ESI,† Section 6). These results therefore suggests that the intramolecular acyl transfer is likely the rate limiting step in this transformation.

**Fig. 1:**
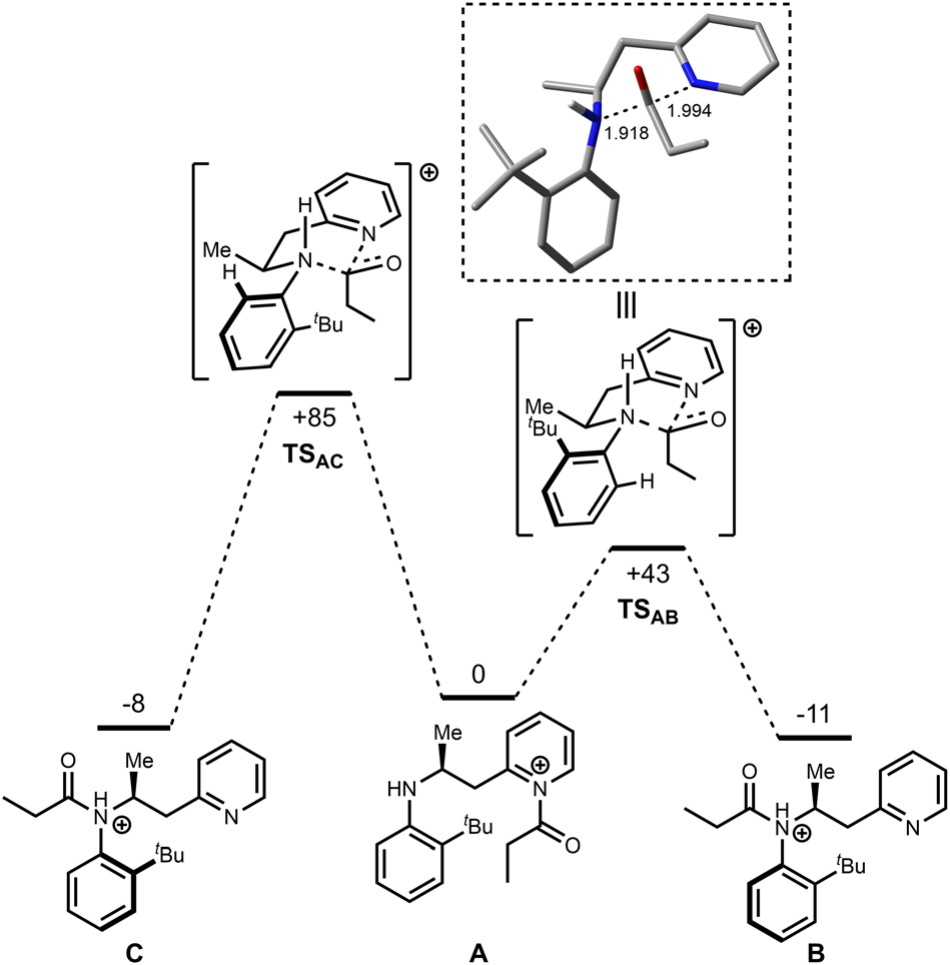
DFT-calculated pathway for the acyl transfer reaction. Energies are Gibbs energies in kJ mol^−1^ at the B3LYP/6-31G* level at 353 K relative to A. Bond lengths are in Ångstroms.

**Scheme 5:**
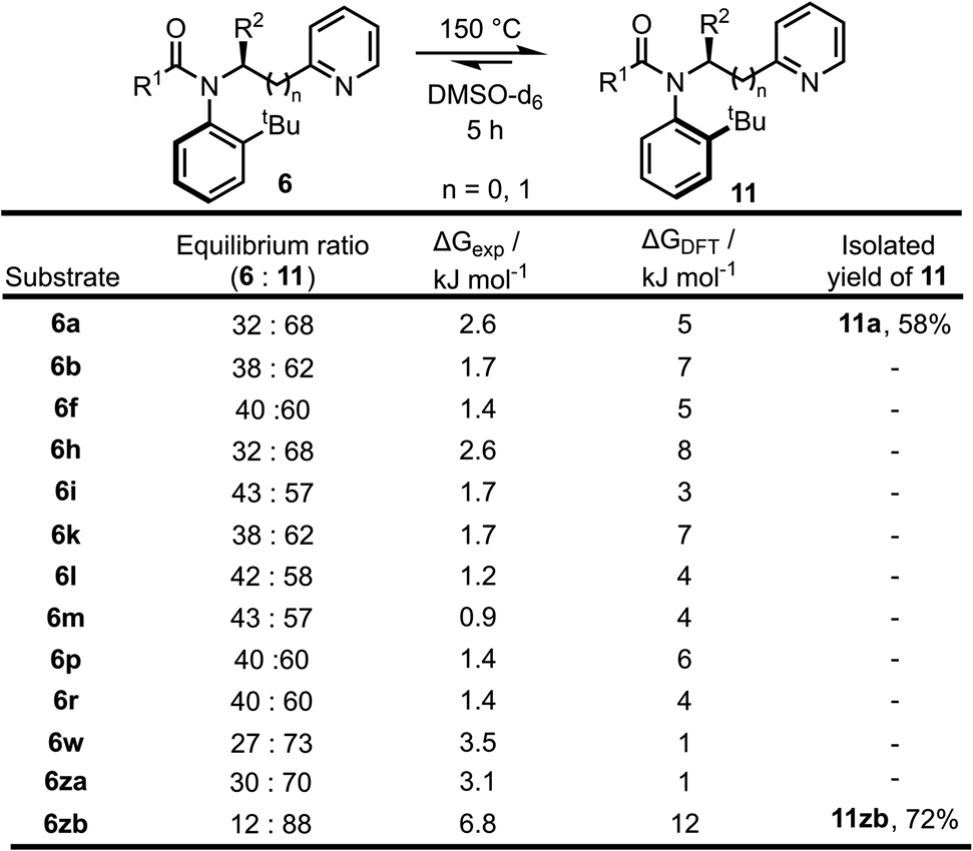
Equilibration of atropisomers 6 into 11 upon heating to 150 °C with experimental and calculated (DFT, B3LYP/6-31G*) Δ*G* values at 150 °C.

Based on the theory that the intramolecular acyl transfer is rate limiting, this step was examined in more detail using DFT calculations and the resulting potential energy surface is shown in Fig. 1. Two potential atropisomers (B and C) were located that arise from acyl transfer from initially formed product A. In the calculations B and C correspond to the *N*-protonated versions of the isolated products (B would form 6r, for example). Transition states connecting A to B (TS_AB_) and A to C (TS_AC_) were located which correspond to the synchronous transfer of the acyl group from the pyridine to amine groups. In TS_AB_ for example, the acyl group is essentially equidistant between the two nitrogen atoms (Fig. 1 box) and is reminiscent of an S_N_2-type transition state, albeit at an sp^2^-hybridised carbon center. Importantly, TS_AB_ lies at considerably lower energy (+43 kJ mol^−1^ relative to A) than TS_AC_ (+85 kJ mol^−1^) an effect that may be linked to an unfavorable steric interaction between the ^*t*^Bu-substituent and the acyl group in the higher energy transition state. This supports the premise that the reaction is under kinetic control, determined by the topology of this transition state, especially when considering that B (−11 kJ mol^−1^) and C (−8 kJ mol^−1^) are very similar in energy, implying that there is no significant thermodynamic preference for either product. Note, the calculated relative energies of cationic species B and C (*i.e.*B being slightly lower in energy than C) differ from the trend in relative energies seen when comparing the analogous neutral isomers (6 and 11), but it is the latter which will ultimately dictate the equilibrium ratio obtained following thermal isomerization (*vide infra*, Scheme 5).

To further support idea that the reactions are under kinetic control, a range of other substrates 6 prepared in the substrate scoping series were heated in an attempt to promote isomerisation to the thermodynamic atropisomer 11 (Scheme 5). In total, 13 isomerically pure pyridine-containing products 6 were heated for 5 hours at 150 °C in *d*_6_-DMSO. The samples were then cooled to RT and analysed by ^1^H NMR to determine the dr. The samples were then re-heated for a further 1 hour at 150 °C, and if no change in dr was observed it was assumed that equilibrium had been reached. In all cases, atropisomer 11 was found to be the major isomer after heating. In two cases, (11a and 11zb), the newly formed isomer was isolated in good yield as a single atropisomer following column chromatography. These experimentally determined equilibrium diastereomeric ratios were used to calculate the difference in free energy between the two atropisomers (Δ*G*_exp_) and for selected cases, this was compared to the free energy difference calculated using DFT (Δ*G*_DFT_). In general, Δ*G*_DFT_ was larger than Δ*G*_exp_, although the values were within the error of the calculation in most cases and the calculated and experimental results were in agreement that 11 is the more stable atropisomer.

**Scheme 6:**
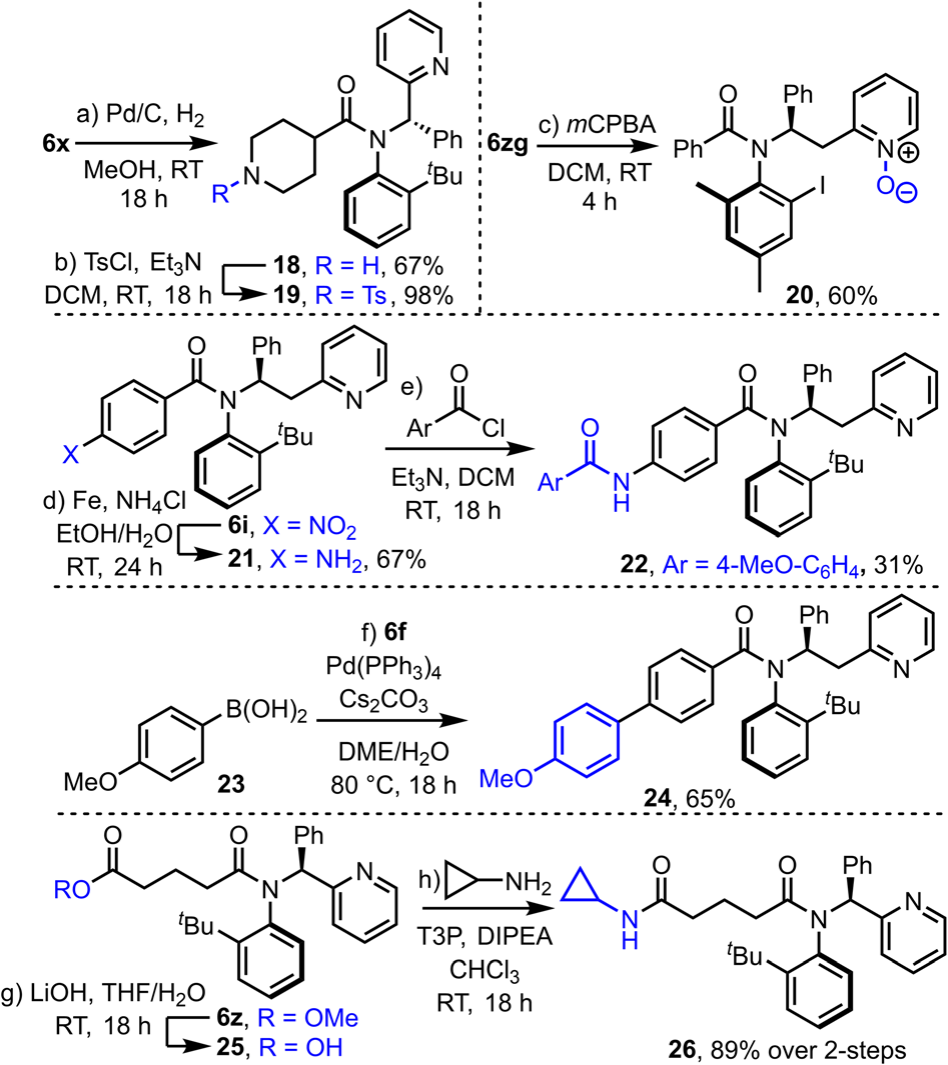
Various synthetic derivatisation reactions of atroisomeric amides produced in this study.

Finally, to show the potential of the products for further synthetic transformations, their compatibility with a range of further derivatization reactions was demonstrated (Scheme 6). For example, hydrogenolysis of Cbz-protected piperidine derivative 6x yielded amine 18, which has been converted into sulfonamide 19 in a straightforward manner. *N*-oxide formation is also relatively straightforward (6zg → 20) using *m*CPBA. Nitro-benzene derivative 6i was converted into aniline 21*via* an iron-mediated reduction, and subsequently acylated to form amide 22. Product 6f is compatible with typical Suzuki–Miyaura cross coupling with boronic acid 23 to form coupled product 24, and ester tethered substrate 6z underwent hydrolysis and coupling to form amide 26 in high yield over this 2-step sequence.

## Conclusions

In summary, a new stereoselective synthesis for the synthesis of C–N atropisomeric amides has been developed, enabled by intramolecular acyl transfer from an internal Lewis-basic activating group. The method is broad in scope, high yielding and in most cases highly atropselective, with the products isolable as pure, single atropisomers following chromatography. The reactions are thought to be under kinetic control and form products that are configurationally stable at RT. The kinetic products can also be isomerised into the more stable thermodynamic atropisomer upon heating. Supporting DFT studies align well with the synthetic outcomes, and along with mechanistic studies performed on a DMAP-like analogue, support operation *via* initial fast acylation of the Lewis basic group, followed by rate-determining acyl transfer, which also controls formation of the kinetic atropisomer.

The importance of C–N atropisomeric amides in medicinal chemistry and agrochemistry (and indeed interest in the control of axial chirality more broadly) means that this concept is expected to be of much interest to scientists in these fields, especially those working to prepare biologically active chiral materials. The ability of ubiquitous nitrogen-containing functional groups (pyridines and tertiary amines) to serve as Lewis bases in these atroposelective transformations is also noteworthy.

## Data availability

The data that support the findings of this study are available in the ESI† of this article, including detailed experimental procedures, characterization data for new compounds, and details of computational methods. Crystallographic data have been deposited with the Cambridge Crystallographic Data Centre: CCDC 2363258 (6b), 2363259 (6e) 2363260 (6w) 2362628 (6zf), 2362629 (6zi) and 2363322 (9a).

## Author contributions

The project was conceived and designed by WPU and RJA. Initial method development and optimisation was done by JMW. Reaction scope and further method development was done by JMW, NJR, CEM, VEW and LCD. The manuscript was written through contributions from all authors. X-ray crystallography data acquisition, processing and analysis was done by PCW, ACW and RJG. Computattional chemistry was done by JML and JMW. AHM provided general advice and industrial steer. The project was directed and managed by WPU and RJA.

## Conflicts of interest

There are no conflicts to declare.

## Supplementary Material

SC-016-D4SC05760K-s001

SC-016-D4SC05760K-s002
