# Supplementary material for: Stereoselective synthesis of atropisomeric amides enabled by intramolecular acyl transfer
Source: Chem Sci. 2025 Jan 23;16(9):3938–45. doi: 10.1039/d4sc05760k (PMC11776508; doi:10.1039/d4sc05760k)
Supplement: SC-016-D4SC05760K-s001 [file SC-016-D4SC05760K-s001.pdf]

Supplementary Information (SI) for Chemical Science.
This journal is © The Royal Society of Chemistry 2025

Contents

1. General Information 2–5
2. Reaction Optimisation 6
3. Compound Characterisation Data and Procedures 7–59
4. Amide Rotamers 60
5. Thermal Isomerisation Kinetics 61–62
6. Evidence for the Formation of an Acyl Pyridinium Intermediate 63
7. Computational Chemistry 64–199
8. ^{1}H and ^{13}C NMR Spectra 200–283
9. References 284–285

1) General Information

For all results except those relating to Scheme 3C

Unless otherwise stated, all reactions were carried out at RT under an inert (N_2 or Ar) atmosphere in oven-dried glassware. Except where stated all reagents were purchased from commercial sources: Merck (Sigma Aldrich), Alfa Aesar, Acros Organics, Fisher Chemicals, VWR, TCI, Across chemicals and Fluorochem and were used without further purification. Anhydrous CH_2Cl_2, toluene, MeCN, Et_2O and DMF were obtained from an Innovative Technology Inc. PureSolv® solvent purification system. Dry THF was obtained from the SPS laboratory system and used immediately after being dispensed. Dry Et_3N and DIPEA obtained by drying with CaH_2 and then distilling and storing over KOH or 3 Å molecular sieves, under Ar. Anhydrous MeOH, DMSO, acetone, ^{t}BuOH, benzene, CCl_4 and ^{n}BuOH were purchased from Sigma Aldrich and used as supplied.

^{1}H NMR spectra were recorded at 400 MHz on Bruker AV400 or Bruker AMX 400/JEOL ECS-400 and at 500 MHz on Bruker DRX500 MHz Ultra ShieldTM spectrometry. ^{13}C NMR spectra were recorded at 101 MHz on Bruker AV 400 or Bruker AMX 400 MHz Ultra ShiledTM and 126 MHz on Bruker DRX500 MHz Ultra ShiledTM spectrometry. ^{19}F NMR spectra were recorded at 376 MHz on Bruker AV400 or Bruker AMX 400/JEOL ECS-400 spectrometry. ^{31}P NMR spectra were recorded at 162 MHz on Bruker AV400 or Bruker AMX 400/JEOL ECS-400 spectrometry.

All spectral data was acquired at 298 K (25 °C) unless stated otherwise and samples were dissolved in $CDCl_3$ unless specified otherwise. Chemical shifts (δ) are reported in parts per million (ppm), with residual solvent peaks: $CDCl_3$: δ_H = 7.26, $CDCl_3$: δ_C = 77.0, $(CD_3)_2SO$: δ_H = 2.50, δ_C = 39.5, CD_3OD: δ_H = 3.31, δ_C = 49.0, C_6D_6: δ_H = 7.16, δ_C = 128.1, CD_2Cl_2: δ_H = 5.32, δ_C = 53.8, $(CD_3)_2CO$: δ_H = 2.05, δ_C = 206.3, D_2O: δ_H = 4.79, $DCON(CD_3)_2$: δ_H = 8.03, δ_C = 163.2, being used for internal reference. The multiplicity abbreviations used are: s, singlet; d, doublet; t, triplet; q, quartet; p, pentet; m, multiplet; dd, doublet of doublets; dt doublet of triplets; td, triplet of doublets; tt, triplet of triplets; ddd, doublet of doublets of doublets; pd, pentet of doublets; where br indicates a broad signal, and app. indicates an apparent. ^{1}H experiments are reported as: chemical shift in ppm, quoted to the nearest 0.01 ppm, (integration, multiplicity, coupling constant and assignment (where possible)). ^{13}C experiments are reported as: chemical shift in ppm, quoted to the nearest 0.1 ppm, (carbon assignment (where

possible) or multiplicity, coupling constant and assignment (where applicable)). ^{19}F experiments are reported as: chemical shift in ppm, quoted to the nearest 0.1 ppm, (multiplicity, coupling constant and assignment (where possible)). ^{31}P experiments are reported as: chemical shift in ppm, quoted to the nearest 0.1 ppm, (multiplicity, and assignment (where possible)).

Assignment of compounds was achieved through use of ^{135}DEPT, COSY, HSQC and HMBC experiments. Spectra were analysed using MestReNova 12.0.3-21384 software and values of coupling constant (*J*) are reported in Hertz (Hz) to the nearest 0.5 Hz. The term "overlapping" is used to describe resonance peak, which is behind another resonance peak, *i.e.*, compound resonance behind the solvent peak or combination of two resonance peaks. The systematic chemical names were generated using the IUPAC name generator tool option is included within the ChemBioFDaw Ultra 19.1 software.

Infrared (IR) spectra were recorded on a PerkinElmer UATR 2 or Pekin Elmer Spectrum 100 spectrometer fitted with a universal Attenuated Total Reflectance (ATR) accessory; data was recorded as a thin film dispersed from either CH_2Cl_2 or $CDCl_3$, neat or solid state by ATR-FTIR. IR-recorded experiments are reported as: IR (method of recorded) ν_{max} (IR absorption maxima) / unit (cm^{-1}) chemical absorption (assignment (where possible)). The intensity of each absorbance band gives the annotated appearance, and each bond was described as w (weak), m (medium), s (strong), sh (sharp) and with the prefix v (very) and suffix br (broad).

High Resolution Mass Spectra (HRMS) were obtained by the University of York Mass Spectrometry Service, recorded on a Waters XEVO G2-XS TOF, Waters Synapt G2S TOF or Bruker Micro-TOF mass spectrometer, with HRMS mode incorporating a lock-in mass into the mobile phase (leucine enkehalin) or on a Bruker Daltonics, Micro-TOF spectrometer, using Electrospray Ionisation (ESI) or Atmospheric Pressure Chemical Ionisation (APCI), positive or negative generative modes.

Thin Layer Chromatography (TLC) was carried out on Merck silica gel $60F_{254}$ pre-coated aluminium foil sheets and was visualised using UV light (λ = 254 nm, short wavelength) or UV light (λ = 366 nm, long wavelength) and stained with basic aqueous potassium permanganate ($KMnO_4$), ninhydrin or vanillin solution dip. Concentration under reduced pressure or *vacuo*

was performed using a Büchi® Rotavapor® R-210 evaporator with jack and water bath, 29/32 joint, 240V rotary evaporator using a mixture of acetone and dry ice or ice/water as the coolant. Flash column chromatography was conducted using Aldrich technical grade silica gel (SiO_2), 60 Å, 230-400 mesh, 40-63 µm particle size, under a light positive pressure of air, eluting with the specified solvent system.

Melting points were recorded as decomposition temperature range and measured on a Stuart SMP10 or Gallenkamp apparatus using open tubes with no corrections. Before measuring the melting point, in most instances, the solids were purified by recrystallisation after purification by column chromatography, where "(from [solvent])" donating solvent systems were used, e.g. single or multiple.

X-ray crystallography data was collected, solved, and refined by Dr Adrian C. Whitwood or Dr Richard J. Gammons in the School of Chemistry at the University of York or by Dr Paul G. Waddell in the School of Natural and Environmental Sciences at Newcastle University. Diffraction data were collected at 100 K on an Oxford Diffraction SuperNova diffractometer with Cu-K_α radiation (λ = 1.54184 Å) using a HyPix-6000HE detector. The crystal was cooled with an Oxford Instruments Cryojet. Diffractometer control, data collection, initial unit cell determination, frame integration and unit-cell refinement were carried out with CrysAlisPro, Face-indexed absorption corrections were applied using spherical harmonics, implemented in SCALE3 ABSPACK scaling algorithm within CrysAlisPro. OLEX2 was used for overall structure solution, refinement and preparation of computer graphics and publication data. Within OLEX2, the algorithms used for structure solution were 'Superflip charge-flipping smtbx-flip charge-flipping ShelXT dual-spaceRefinement by full-matrix least-squares used the SHELXL algorithm within OLEX2. All non-hydrogen atoms were refined anisotropically. Hydrogen atoms were placed using a riding model and included in the refinement at calculated positions. CrystalMaker® 10 software was also used to visualise the X-ray structures with their corresponding CCDC deposit numbers given in the manuscript.

For all results relating to Scheme 3C

Reactions were carried out under an atmosphere of nitrogen. Room temperature (RT) refers to 20-25 °C. Temperatures of 0 °C were obtained using an ice/water bath. Reactions at

elevated temperature were performed using an oil bath equipped with a contact thermometer.

All solvents (including anhydrous solvents) were used as supplied without prior purification. All other reagents were used directly as supplied by major chemical suppliers, or following purification procedures described by Perrin and Armarego.[1]

Thin layer chromatography was performed on Merck Kieselgel 60 F_{254} 0.25 mm pre-coated aluminium plates. Product spots were visualized under UV light (λ = 254 nm) and/or by staining with potassium permanganate solution. Flash chromatography was performed using VWR silica gel 60 (40-63 µm particle size) using head pressure by means of a nitrogen line.

NMR spectroscopy was carried out using Bruker 300 MHz, 400 MHz, 500 MHz, Cryo 700 MHz spectrometers in the deuterated solvent stated, using the residual non-deuterated solvent signal as an internal reference. Chemical shifts are quoted in ppm with signal splitting recorded as singlet (s), doublet (d), triplet (t), quartet (q), quintet (qn), sextet (sext), septet (sept), octet (oct), nonet (non) and multiplet (m). The abbreviation br denotes broad. Coupling constants, *J*, are measured to the nearest 0.1 Hz and are presented as observed.

Infrared spectra were recorded on a PerkinElmer UATR Two spectrometer with attenuated total reflectance. Absorption maxima (λ_{max}) are reported in wavenumbers (cm^{-1}).

HRMS was recorded on a Waters Xevo G2-XS Quadrupole Time-of-Flight (QToF) spectrometer equipped with a Waters Acquity UPLC i-Class LC system, under conditions of electrospray ionisation (ESI). The mass reported is that containing the most abundant isotopes, with each value rounded to 4 decimal places and within 10 ppm of the calculated mass.

Normal phase chiral HPLC was performed on a Dionex Ultimate 3000 HPLC unit equipped with UV-vis diode-array detector, fitted with the appropriate DAICEL column (dimensions: 0.46 cm ø x 25 cm) along with the corresponding guard column (0.4 cm ø x 1 cm). Wavelengths (λ) are reported in nm, retention times (t_R) are reported in minutes and solvent flow rates are reported in mL min^{-1}

2) Reaction Optimisation

All optimisation reactions were performed on a 0.151 mmol scale at 0.2 M concentration under an argon atmosphere by mixing **4a** (0.151 mmol) with acid chloride **10** (equivalents in the Table) in with the solvent, base and temperature combination noted in the Table, for 18 h unless stated. Each reaction was quenched with sat. aq. $NaHCO_3$ (10 mL) and extracted with DCM (3 x 10 mL). The combined organic layers were dried over $MgSO_4$, filtered and concentrated *in vacuo*. ^{1}H NMR spectra of the crude reaction mixtures were collected and used to determine the conversion and diastereomeric ratio (*dr*).

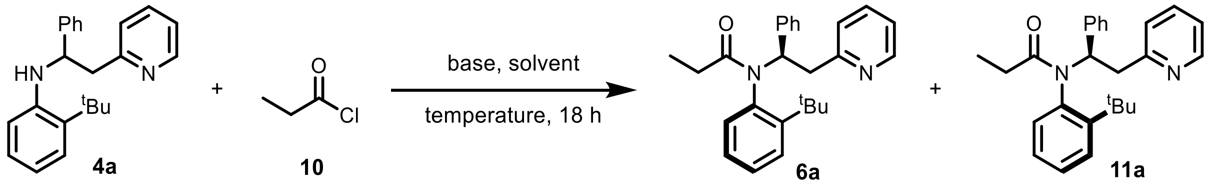

Entry	Solvent	Temp / °C	10 / equiv.	Base (equiv.)	Conversion / %	6a : 11a
1	DCM	RT	4	NEt_3 (4)	0	n/a
2	$CHCl_3$	RT	4	NEt_3 (4)	0	n/a
3	Benzene	RT	4	NEt_3 (4)	0	n/a
4	THF	RT	4	NEt_3 (4)	0	n/a
5	MeCN	60	4	NEt_3 (4)	22	-
6	$CHCl_3$	60	4	NEt_3 (4)	22	-
7	Benzene	60	4	NEt_3 (4)	0	n/a
8	THF	60	4	NEt_3 (4)	2	-
9	MeCN	80	4	NEt_3 (4)	10	-
10	MeCN	80	1	NEt_3 (4)	4	-
11	MeCN	80	2	NEt_3 (4)	28	-
12	MeCN	80	3	NEt_3 (4)	40	-
13	MeCN	80	4	Pyridine (4)	78	-
14	MeCN	80	4	DIPEA (4)	62	90 : 10
15	MeCN	80	4	K_2CO_3 (4)	60	-
16	MeCN	80	4	$NaHCO_3$ (4)	96	98 : 2
17	MeCN	80	4	None	94	91 : 9
18	MeCN	RT	4	None	76	96 : 4
19	MeCN	RT (7 days)	4	None	84	95 : 5
20	MeCN	40	4	None	88	92 : 8
21	MeCN	60	4	None	98	90 : 10
22	MeCN	80	2 + 2 eq. HCl	None	79	77 : 23
23	MeCN	80	4	$NaHCO_3$ (5)	95	98 : 2
24	MeCN	80	4	$NaHCO_3$ (3)	100	97 : 3
25	MeCN	80	2	$NaHCO_3$ (4)	100	98 : 2
26	MeCN	80	4	$NaHCO_3$ (2)	100	96 : 4
27	MeCN	80	1.2	$NaHCO_3$ (1.5)	90	98 : 2
28	MeCN	80	1.5	$NaHCO_3$ (2)	96	98 : 2
29	MeCN	80	1.5	$NaHCO_3$ (3)	98	98 : 2
**30**	**MeCN**	**80 (1 h)**	**2**	**$NaHCO_3$ (4)**	**100**	**99 : 1**
31	MeCN	RT (1 h)	2	$NaHCO_3$ (4)	100	99 : 1

3) Compound Characterisation Data and Procedures

General Procedure for the Preparation of Acyl Chlorides.

To a suspension of carboxylic acid (4 mmol) in DCM (20 mL) was added DMF (4 drops) followed by oxalyl chloride (12 mmol). The resulting solution was stirred at room temperature for 1–4 h. The reaction mixture was concentrated *in vacuo* and the products were used without further purification.

N-(2-(*tert*-Butyl)phenyl)-1-phenylmethanimine (S1)

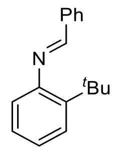

To a solution of 2-*tert*-butylaniline (3.90 mL, 3.73 g, 25.0 mmol) in ethanol (100 mL) was added benzaldehyde (2.50 mL, 2.65 g, 25.0 mL) and the resulting solution was heated to reflux for 18 h. The reaction mixture was concentrated *in vacuo* to yield the title compound (5.93 g, 100%) as an orange oil that was used without further purification. δ_H (400 MHz, $CDCl_3$) 8.34 (1H, s, NCH), 7.96–7.90 (2H, m, ArH), 7.54–7.48 (3H, m, ArH), 7.42 (1H, dd, *J* = 7.5, 2.0 Hz, ArH), 7.27–7.16 (2H, m, ArH), 6.87 (1H, dd, *J* = 7.5, 2.0 Hz, ArH), 1.47 (9H, s, CH_3). Characterisation data matched those reported in the literature.[1]

2-(*tert*-Butyl)-*N*-(1-phenyl-2-(pyridin-2-yl)ethyl)aniline (4a)

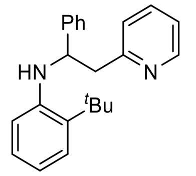

Diisopropylamine (7.00 mL, 5.06 g, 50.0 mmol) was dissolved in dry THF (120 mL) and cooled to –78 °C. *n*-BuLi (20.0 mL, 2.5 M in hexanes, 50.0 mmol) was added slowly and the resulting solution stirred for 15 minutes. 2-Methylpyridine (4.90 mL, 4.66 g, 50.0 mmol) was added slowly and the resulting solution was stirred for 15 minutes. *N*-(2-(*tert*-butyl)phenyl)-1-phenylmethanimine **S1** (5.93 g, 25.0 mmol) was dissolved in dry THF (25 mL) and added dropwise to the reaction mixture. The reaction mixture was warmed to room temperature and stirred for 1 h. The reaction was quenched with sat. aq. NH_4Cl (150 mL) and extracted with ethyl acetate (3 x 150 mL). The combined organic layers were dried over $MgSO_4$, filtered and concentrated *in vacuo*. The crude product was then purified by column chromatography (SiO_2, 19:1 hexane:ethyl acetate) to yield the title compound (5.70 g, 69%) as an off-white solid. R_f 0.18 (19:1 hexane:ethyl acetate); m.p. 70–73 °C; δ_H (400 MHz, $CDCl_3$) 8.62–8.58 (1H, m, ArH), 7.53 (1H, td, *J* = 7.5, 2.0 Hz, ArH), 7.36–7.21 (6H, m, ArH), 7.19–7.13 (1H, m, ArH), 6.98–6.89 (2H, m, ArH), 6.62 (1H, td, *J* = 7.5, 1.5 Hz, ArH), 6.36 (1H, dd, *J* = 8.5, 1.5 Hz, ArH), 5.61 (1H, d, *J* = 4.0 Hz, NH), 4.91 (1H, ddd, *J* = 8.0, 4.0, 4.0 Hz, NCH), 3.47 (1H, dd, *J* = 13.5, 4.0 Hz, C**H**H'), 3.25 (1H, dd, *J* = 13.5, 8.0 Hz, CH**H'**), 1.53 (9H, s, CH_3); δ_C (101 MHz, $CDCl_3$) 158.5 (ArC), 149.2 (ArC), 144.8 (ArC), 143.6 (ArC), 136.5 (ArC), 133.3 (ArC), 128.7 (ArC), 127.0 (ArC), 126.9 (ArC), 126.4 (ArC), 126.1 (ArC), 124.0 (ArC), 121.9 (ArC), 116.5 (ArC), 112.6 (ArC), 58.6

(NCH), 47.0 (CH_2), 34.4 (**C**(CH_3)$_3$), 29.8 (C(**C**H_3)$_3$); ν_{max}/cm^{-1} (thin film) 3422, 2956, 1593, 1577, 1510, 1474, 1446, 1311, 1273, 1055, 743, 701; HRMS (ESI); calcd. for $C_{23}H_{27}N_2^+$, 331.2169. Found: [MH]$^+$, 331.2181 (–3.6 error ppm).

***N*-(2-(*tert*-Butyl)phenyl)-*N*-(1-phenyl-2-(pyridin-2-yl)ethyl)propionamide (6a)**

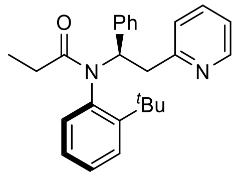

To a mixture of 2-(*tert*-butyl)-*N*-(1-phenyl-2-(pyridin-2-yl)ethyl)aniline **4a** (200 mg, 0.605 mmol) and sodium bicarbonate (203 mg, 2.42 mmol) in dry acetonitrile (3 mL) was added propionyl chloride (0.11 mL, 112 mg, 1.21 mmol) and the resulting mixture was heated to 80 °C for 1 h. The reaction was quenched with sat. aq. $NaHCO_3$ (15 mL) and extracted with DCM (3 x 15 mL). The combined organic layers were dried over $MgSO_4$, filtered and concentrated *in vacuo*. The crude product was then purified by column chromatography (SiO_2, 7:3 hexane:ethyl acetate) to yield the title compound (229 mg, 98%) as a colourless oil. R_f 0.17 (8:2 hexane:ethyl acetate); δ_H (400 MHz, $CDCl_3$) 8.45–8.41 (1H, m, ArH), 7.61 (1H, dd, *J* = 8.5, 1.5 Hz, ArH), 7.45–7.32 (4H, m, ArH), 7.24–7.11 (4H, m, ArH), 7.03–6.93 (2H, m, ArH), 6.80 (1H, d, *J* = 8.0 Hz, ArH), 5.69 (1H, dd, *J* = 12.0, 4.5 Hz, NCH), 3.38 (1H, dd, *J* = 13.5, 4.5 Hz, ArC**H**H'), 2.93 (1H, dd, *J* = 13.5, 12.0 Hz, ArCH**H'**), 2.05–1.81 (2H, m, $COCH_2$), 1.41 (9H, s, C(CH_3)$_3$), 0.97 (3H, t, *J* = 7.5 Hz, CH_3); δ_C (101 MHz, $CDCl_3$) 174.2 (CO), 158.2 (ArC), 149.2 (ArC), 147.7 (ArC), 140.8 (ArC), 138.1 (ArC), 135.9 (ArC), 132.0 (ArC), 131.2 (ArC), 129.1 (ArC), 128.5 (ArC), 127.9 (ArC), 127.1 (ArC), 126.8 (ArC), 123.9 (ArC), 121.3 (ArC), 62.9 (NCH), 42.1 ($ArCH_2$), 36.7 (**C**(CH_3)$_3$), 32.9 (C(**C**H_3)$_3$), 30.0 (CO**C**H_2), 9.0 (CH_3); ν_{max}/cm^{-1} (thin film) 2970, 1656, 1590, 1570, 1487, 1436, 1478, 1263, 1147, 994, 836, 760, 748, 733, 700, 549; HRMS (ESI); calcd. for $C_{26}H_{31}N_2O^+$, 387.2431. Found: [MH]$^+$, 387.2436 (–1.4 error ppm).

***N*-(2-(*tert*-Butyl)phenyl)-*N*-(1-phenyl-2-(pyridin-2-yl)ethyl)propionamide (11a)**

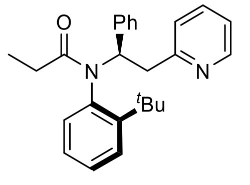

A solution of *N*-(2-(*tert*-butyl)phenyl)-*N*-(1-phenyl-2-(pyridin-2-yl)ethyl)propionamide **6a** (53 mg, 0.137 mmol) in d_6-DMSO (1.0 mL) was heated to 150 °C for 5 h. The reaction mixture was dissolved in DCM (10 mL) and washed with water (3 x 10 mL). The organic layer was dried over $MgSO_4$, filtered and concentrated *in vacuo*. The crude product was then purified by column chromatography (SiO_2, 8:2 → 6:4 hexane:ethyl acetate) to yield the title compound (31 mg, 58%) as a colourless oil. R_f 0.16 (7:3 hexane:ethyl acetate); δ_H (400 MHz, $CDCl_3$) 8.35–8.30 (1H, m, ArH), 7.45–7.29 (3H, m, ArH), 7.26–7.17 (2H, m, ArH), 7.13 (1H, d, *J* = 8.0 Hz, ArH), 7.07–6.92 (4H, m, ArH), 6.82–6.77 (2H, m, ArH), 5.88 (1H, dd, *J* = 10.5, 4.5 Hz, NCH), 3.79 (1H, dd, *J* = 13.0, 4.5 Hz, ArC**H**H'), 3.62 (1H, dd, *J* = 13.0, 10.5 Hz, ArC**H**H'), 2.03 (2H, q, *J* = 7.5 Hz, $COCH_2$), 1.06 (3H, t, *J* = 7.5 Hz, CH_3), 0.95 (9H, s, C(CH_3)$_3$); δ_C (101 MHz, $CDCl_3$) 174.5 (CO), 158.6 (ArC),

148.9 (ArC), 147.8 (ArC), 137.8 (ArC), 137.4 (ArC), 136.0 (ArC), 132.0 (ArC), 131.1 (ArC), 130.9 (ArC), 128.6 (ArC), 127.8 (ArC), 127.7 (ArC), 126.7 (ArC), 124.0 (ArC), 121.2 (ArC), 63.9 (NCH), 44.4 ($ArCH_2$), 36.4 (**C**(CH_3)$_3$), 32.2 (C(**C**H_3)$_3$), 30.1 (CO**C**H_2), 9.4 (CH_3); ν_{max}/cm^{-1} (thin film) 2954, 1650, 1591, 1569, 1489, 1435, 1388, 1248, 1077, 1052, 916, 760, 747, 731, 701; HRMS (ESI); calcd. for $C_{26}H_{30}N_2ONa^+$, 409.2250. Found: $[MNa]^+$, 409.2248 (0.6 error ppm).

2-(*tert*-Butyl)-*N*-(1,2-diphenylethyl)aniline (12)

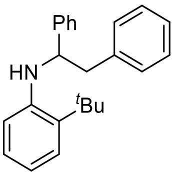

A solution of *N*-(2-(*tert*-butyl)phenyl)-1-phenylmethanimine **S1** (942 mg, 3.97 mmol) in THF (17 mL) was cooled to –78 °C before adding benzylmagnesium chloride (4.00 mL, 2 M in THF, 8.00 mmol) dropwise. The reaction mixture was warmed to room temperature and stirred for 2 h. The reaction was quenched with sat. aq. NH_4Cl (20 mL) and extracted with Et_2O (3 x 20 mL). The combined organic layers were dried over $MgSO_4$, filtered and concentrated *in vacuo*. The crude product was then purified by column chromatography (SiO_2, 19:1 hexane:ethyl acetate) to yield the title compound (1.25 g, 96%) as a yellow solid. R_f 0.61 (19:1 hexane:ethyl acetate); m.p. 66–69 °C; δ_H (400 MHz, $CDCl_3$) 7.37–7.13 (11H, m, ArH), 6.90–6.84 (1H, m, ArH), 6.61–6.55 (1H, m, ArH), 6.34 (1H, dd, *J* = 8.0, 1.5 Hz, ArH), 4.71 (1H, ddd, *J* = 9.0, 5.0, 3.5 Hz, NCH), 4.33 (1H, d, *J* = 3.5 Hz, NH), 3.26 (1H, dd, *J* = 14.0, 5.0 Hz, C**H**H'), 3.00 (1H, dd, *J* = 14.0, 9.0 Hz, CH**H'**), 1.28 (9H, s, CH_3); δ_C (101 MHz, $CDCl_3$) 145.0 (ArC), 144.0 (ArC), 137.7 (ArC), 133.4 (ArC), 129.5 (ArC), 128.8 (2 x ArC), 127.1 (2 x ArC), 126.9 (ArC), 126.5 (ArC), 126.1 (ArC), 117.0 (ArC), 113.1 (ArC), 59.3 (NCH), 45.9 (CH_2), 34.2 (**C**(CH_3)$_3$), 29.8 (C(**C**H_3)$_3$); ν_{max}/cm^{-1} (thin film) 3461, 3061, 2966, 1599, 1578, 1505, 1447, 1302, 1263, 1056, 742, 698; HRMS (ESI); calcd. for $C_{24}H_{28}N^+$, 330.2216. Found: $[MH]^+$, 330.2243 (–8.0 error ppm).

***N*-(2-(*tert*-Butyl)phenyl)-*N*-(1,2-diphenylethyl)propionamide (13)**

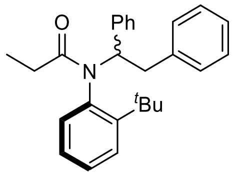

To a mixture of 2-(*tert*-butyl)-*N*-(1,2-diphenylethyl)aniline **12** (100 mg, 0.303 mmol) and sodium bicarbonate (102 mg, 1.12 mmol) in dry acetonitrile (1.5 mL) was added propionyl chloride (0.050 mL, 56 mg, 0.606 mmol) and the resulting mixture was heated to 80 °C for 1 h. The reaction was quenched with sat. aq. $NaHCO_3$ (10 mL) and extracted with DCM (3 x 10 mL). The combined organic layers were dried over $MgSO_4$, filtered and concentrated *in vacuo*. The crude product was then purified by column chromatography (SiO_2, 9:1 hexane:ethyl acetate) to yield the title compound (22 mg, 19%) as a colourless oil and a 3:1 mixture of diastereoisomers. R_f 0.29 (9:1 hexane:ethyl acetate); δ_H (400 MHz, $CDCl_3$) 7.63 (1H, dd, *J* = 8.0, 1.5 Hz, ArH, major diastereoisomer), 7.42–7.27 (6H, m, ArH, both diastereoisomers), 7.26–7.02 (15H, m, ArH, both diastereoisomers), 7.00–6.94 (2H, m, ArH), 6.92–6.86 (2H, m, ArH),

6.70–6.65 (2H, m, ArH), 5.62 (1H, dd, *J* = 11.5, 3.0 Hz, NCH, minor diastereoisomer), 5.36 (1H, dd, *J* = 12.0, 4.0 Hz, NCH, major diastereoisomer), 3.58 (1H, dd, *J* = 12.0, 3.0 Hz, ArC**H**H', minor diastereoisomer), 3.32 (1H, dd, *J* = 12.0, 11.5 Hz, ArCH**H'**, minor diastereoisomer), 3.18 (1H, dd, *J* = 13.5, 4.0 Hz, ArC**H**H', major diastereoisomer), 2.63 (1H, dd, *J* = 13.5, 12.0 Hz, ArCH**H'**, major diastereoisomer), 2.09 (2H, q, *J* = 7.5 Hz, $COCH_2$, minor diastereoisomer), 2.04–1.81 (2H, m, $COCH_2$, major diastereoisomer), 1.45 (9H, s, $C(CH_3)_3$, major diastereoisomer), 1.14 (3H, t, *J* = 7.5 Hz, CH_3, minor diastereoisomer), 0.98 (3H, t, *J* = 7.5 Hz, CH_3, major diastereoisomer), 0.91 (9H, s, $C(CH_3)_3$, minor diastereoisomer); δ_C (101 MHz, $CDCl_3$) 174.1 (CO, minor diastereoisomer), 174.0 (CO, major diastereoisomer), 147.9 (ArC), 147.7 (ArC), 140.9 (ArC), 138.4 (ArC), 138.1 (ArC), 138.0 (ArC), 137.5 (ArC), 137.0 (ArC), 132.2 (ArC), 131.7 (ArC), 131.2 (2 x ArC), 131.1 (ArC), 129.4 (ArC), 129.3 (ArC), 129.2 (ArC), 128.6 (ArC), 128.5 (ArC), 128.1 (ArC), 128.0 (ArC), 127.9 (ArC), 127.7 (2 x ArC), 127.1 (ArC), 126.8 (ArC), 126.6 (ArC), 126.2 (ArC), 126.0 (ArC), 65.6 (NCH, minor diastereoisomer), 64.6 (NCH, major diastereoisomer), 42.7 ($ArCH_2$, minor diastereoisomer), 39.8 ($ArCH_2$, major diastereoisomer), 36.7 (**C**$(CH_3)_3$, major diastereoisomer), 36.4 (**C**$(CH_3)_3$, minor diastereoisomer), 32.9 (C(**C**$H_3)_3$, major diastereoisomer), 32.1 (C(**C**$H_3)_3$, minor diastereoisomer), 30.0 (CO**C**H_2, minor diastereoisomer), 29.9 (CO**C**H_2, major diastereoisomer), 9.3 (CH_3, minor diastereoisomer), 9.0 (CH_3, major diastereoisomer); ν_{max}/cm^{-1} (thin film) 2971, 1651, 1487, 1436, 1378, 1261, 1079, 1051, 909, 760, 729, 697, 645, 543; HRMS (ESI); calcd. for $C_{27}H_{32}NO^+$, 386.2478. Found: $[MH]^+$, 386.2480 (–0.4 error ppm).

1-(Pyridin-2-yl)propan-2-one (S2)

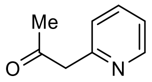

A solution of *N*,*N*-diisopropylamine (1.40 mL, 1.01 g, 10.0 mmol) was dissolved in dry THF (25 mL) and cooled to –78 °C before adding *n*-BuLi (4.00 mL, 2.5 M in hexane, 10.0 mmol) and stirring for 15 minutes. 2-Methylpyridine (0.490 mL, 466 mg, 5.00 mmol) was added dropwise and the mixture was stirred for 15 min. *N*-Methoxy-*N*-methylacetamide (1.10 mL, 1.03 g, 10.0 mmol) was added dropwise and the resulting mixture was warmed to room temperature and stirred for 2 h. The reaction was quenched with water (30 mL) and extracted with diethyl ether (3 x 30 mL). The combined organic layers were dried over $MgSO_4$, filtered and concentrated *in vacuo*. The crude product was then purified by column chromatography (SiO_2, ethyl acetate) to yield the title compound (578 mg, 86%, 91:9 keto:enol) as a yellow oil. R_f 0.41 (ethyl acetate); δ_H (400 MHz, $CDCl_3$) 8.50–8.45 (1H, m, ArH, keto), 8.14–8.09 (1H, m, ArH, enol), 7.58 (1H, td, *J* = 7.5, 2.0 Hz, ArH, keto), 7.48–7.43 (1H, m, ArH, enol), 7.16–7.08 (2H, m, ArH, keto), 6.84–6.76 (2H, m, ArH, enol), 5.23 (1H, s, CH, enol), 3.85 (2H, s, CH_2, keto), 2.15 (3H, s, CH_3, keto), 1.95 (3H, s, CH_3, enol). Characterisation data matched those reported in the literature.[2]

2-(*tert*-Butyl)-*N*-(1-(pyridin-2-yl)propan-2-yl)aniline (4b)

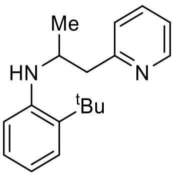

To a solution of 1-(pyridin-2-yl)propan-2-one **S2** (513 mg, 3.80 mmol) in dry 1,2-dichloroethane (6.0 mL) was added 2-*tert*-butylaniline (0.540 mL, 515 mg, 3.45 mmol), acetic acid (0.220 mL, 228 mg, 3.80 mmol) and sodium triacetoxyborohydride (1.17 g, 5.52 mmol) and the mixture was stirred at 60 °C for 72 h. The reaction mixture was diluted with water (40 mL) and extracted with DCM (3 x 40 mL). The combined organic layers were dried over $MgSO_4$, filtered and concentrated *in vacuo*. The crude product was then purified by column chromatography (SiO_2, 9:1 hexane:ethyl acetate) to yield the title compound (131 mg, 14%) as an orange oil. R_f 0.24 (9:1 hexane:ethyl acetate); δ_H (400 MHz, $CDCl_3$) 8.60–8.56 (1H, m, ArH), 7.61 (1H, td, *J* = 7.5, 2.0 Hz, ArH), 7.26 (1H, dd, *J* = 8.0, 1.5 Hz, ArH), 7.23–7.19 (1H, m, ArH), 7.17–7.12 (2H, m, ArH), 6.78 (1H, d, *J* = 8.0 Hz, ArH), 6.67 (1H, td, *J* = 7.5, 1.5 Hz, ArH), 4.57 (1H, s, NH), 4.15–4.04 (1H, m, NCH), 3.15 (1H, dd, *J* = 13.5, 6.0 Hz, C**H**H'), 3.06 (1H, dd, *J* = 13.5, 6.0 Hz, CH**H'**), 1.43 (9H, s, $C(CH_3)_3$), 1.27 (3H, d, *J* = 6.0 Hz, CH_3); δ_C (101 MHz, $CDCl_3$) 159.5 (ArC), 149.3 (ArC), 145.2 (ArC), 136.4 (ArC), 133.2 (ArC), 127.2 (ArC), 126.5 (ArC), 124.0 (ArC), 121.5 (ArC), 116.3 (ArC), 111.9 (ArC), 48.8 (NCH), 44.9 (CH_2), 34.2 (**C**$(CH_3)_3$), 29.9 (C(**C**$H_3)_3$), 20.2 (CH_3); ν_{max}/cm^{-1} (thin film) 3411, 1961, 1590, 1575, 1508, 1445, 1307, 1259, 1054, 995, 742; HRMS (ESI); calcd. for $C_{18}H_{25}N_2^+$, 269.2012. Found: $[MH]^+$, 269.2016 (–1.3 error ppm).

***N*-(2-(*tert*-Butyl)phenyl)-*N*-(1-phenyl-2-(pyridin-2-yl)ethyl)furan-2-carboxamide (6b)**

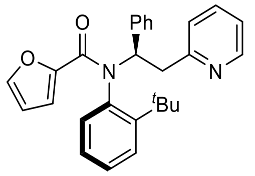

To a mixture of 2-(*tert*-butyl)-*N*-(1-phenyl-2-(pyridin-2-yl)ethyl)aniline **4a** (200 mg, 0.605 mmol) and sodium bicarbonate (203 mg, 2.42 mmol) in dry acetonitrile (3 mL) was added 2-furoyl chloride (0.120 mL, 158 mg, 1.21 mmol) and the resulting mixture was heated to 80 °C for 1 h. After 1 hour the reaction was deemed incomplete by TLC and so additional 2-furoyl chloride (0.060 mL, 79 mg, 0.605 mmol) was added and the mixture was stirred for a further 1 h. The reaction was quenched with sat. aq. $NaHCO_3$ (15 mL) and extracted with DCM (3 x 15 mL). The combined organic layers were dried over $MgSO_4$, filtered and concentrated *in vacuo*. At this point, a ^{1}H NMR spectrum of the crude product was taken to determine the *dr* (>95:5) of the reaction before chromatography. The crude product was then purified by column chromatography (SiO_2, 8:2 hexane:ethyl acetate) to yield the title compound (216 mg, 84%) as a white solid. R_f 0.11 (8:2 hexane:ethyl acetate); m.p. 130–134 °C; δ_H (400 MHz, $CDCl_3$) 8.45–8.40 (1H, m, ArH), 7.57–7.47 (3H, m, ArH), 7.41–7.27 (3H, m, ArH), 7.26–7.08 (5H, m, ArH), 6.97–6.91 (1H, m, ArH), 6.85 (1H, d, *J* = 8.0 Hz, ArH), 6.06 (1H, dd, *J* = 3.5, 2.0 Hz, ArH), 5.92 (1H, dd, *J* = 11.5, 4.5 Hz, NCH), 5.19 (1H, d, *J* = 3.5 Hz, ArH), 3.44 (1H, dd, *J* = 13.5, 4.5 Hz,

CHH'), 3.09 (1H, dd, J = 13.5, 11.5 Hz, CH**H'**), 1.20 (9H, s, CH_3); δ_C (101 MHz, $CDCl_3$) 159.6 (CO), 158.0 (ArC), 149.3 (ArC), 148.5 (ArC), 147.9 (ArC), 144.4 (ArC), 140.0 (ArC), 137.6 (ArC), 136.0 (ArC), 132.3 (ArC), 131.4 (ArC), 129.3 (ArC), 128.9 (ArC), 128.0 (ArC), 127.4 (ArC), 126.7 (ArC), 124.0 (ArC), 121.4 (ArC), 116.1 (ArC), 110.9 (ArC), 63.6 (NCH), 41.6 (CH_2), 36.6 (**C**(CH_3)$_3$), 32.7 (C(**C**H_3)$_3$); ν_{max}/cm^{-1} (thin film) 2961, 2243, 1634, 1591, 1470, 1436, 1394, 1354, 1305, 1183, 1140, 1092, 1012, 911, 699, 756, 726, 557; HRMS (ESI); calcd. for $C_{28}H_{29}N_2O_2^+$, 425.2224. Found: $[MH]^+$, 425.2214 (2.3 error ppm).

_N_-(2-(_tert_-Butyl)phenyl)-_N_-(1-phenyl-2-(pyridin-2-yl)ethyl)hexanamide (6c)

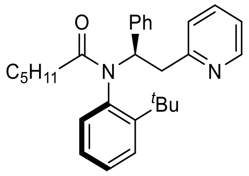

To a mixture of 2-(*tert*-butyl)-*N*-(1-phenyl-2-(pyridin-2-yl)ethyl)aniline **4a** (200 mg, 0.605 mmol) and sodium bicarbonate (203 mg, 2.42 mmol) in dry acetonitrile (3 mL) was added hexanoyl chloride (0.250 mL, 245 mg, 1.82 mmol) and the resulting mixture was heated to 80 °C for 1 h. The reaction was quenched with sat. aq. $NaHCO_3$ (15 mL) and extracted with DCM (3 x 15 mL). The combined organic layers were dried over $MgSO_4$, filtered and concentrated *in vacuo*. At this point, a ^{1}H NMR spectrum of the crude product was taken to determine the *dr* (>96:4) of the reaction before chromatography. The crude product was then purified by column chromatography (SiO_2, 8:2 hexane:ethyl acetate) to yield the title compound (250 mg, 96%) as a colourless oil. R_f 0.23 (8:2 hexane:ethyl acetate); δ_H (400 MHz, $CDCl_3$) 8.43–8.38 (1H, m, ArH), 7.60 (1H, dd, J = 8.0, 1.5 Hz, ArH), 7.44–7.29 (4H, m, ArH), 7.23–7.09 (4H, m, ArH), 7.02 (1H, dd, J = 8.0, 1.5 Hz, ArH), 6.94 (1H, dd, J = 7.5, 5.0 Hz, ArH), 6.78 (1H, d, J = 8.0 Hz, ArH), 5.72 (1H, dd, J = 12.0, 4.5 Hz, NCH), 3.35 (1H, dd, J = 13.5, 4.5 Hz, ArC**H**H'), 2.92 (1H, dd, J = 13.5, 12.0 Hz, ArCH**H'**), 1.98–1.81 (2H, m, CH_2), 1.54–1.45 (2H, m, CH_2), 1.42 (9H, s, $C(CH_3)_3$), 1.20–0.99 (4H, m, 2 x CH_2), 0.77 (3H, t, J = 7.0 Hz, CH_3); δ_C (101 MHz, $CDCl_3$) 173.5 (CO), 158.2 (ArC), 149.2 (ArC), 147.7 (ArC), 140.8 (ArC), 138.1 (ArC), 135.9 (ArC), 132.1 (ArC), 131.2 (ArC), 129.1 (ArC), 128.5 (ArC), 127.9 (ArC), 127.1 (ArC), 126.7 (ArC), 123.9 (ArC), 121.2 (ArC), 62.7 (NCH), 42.1 ($ArCH_2$), 36.7 (CH_2, **C**(CH_3)$_3$), 32.9 (C(**C**H_3)$_3$), 31.5 (CH_2), 24.6 (CH_2), 22.4 (CH_2), 13.9 (CH_3); ν_{max}/cm^{-1} (thin film) 2956, 1653, 1590, 1569, 1486, 1436, 1383, 1262, 1218, 1091, 1051, 760, 699, 550; HRMS (ESI); calcd. for $C_{29}H_{37}N_2O^+$, 429.2900. Found: $[MH]^+$, 429.2903 (–0.7 error ppm).

_N_-(2-(_tert_-Butyl)phenyl)-_N_-(1-phenyl-2-(pyridin-2-yl)ethyl)acrylamide (6d)

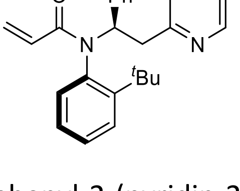

To a mixture of 2-(*tert*-butyl)-*N*-(1-phenyl-2-(pyridin-2-yl)ethyl)aniline **4a** (200 mg, 0.605 mmol) and sodium bicarbonate (203 mg, 2.42 mmol) in dry acetonitrile (3 mL) was added acryloyl chloride (0.100 mL, 110 mg, 1.21 mmol) and the resulting mixture was heated to 80

°C for 1 h. After 1 h the reaction was deemed incomplete by TLC and so acryloyl chloride (0.020 mL, 27 mg, 0.303 mmol) was added and the mixture stirred for a further 1 h. The reaction was quenched with sat. aq. $NaHCO_3$ (15 mL) and extracted with DCM (3 x 15 mL). The combined organic layers were dried over $MgSO_4$, filtered and concentrated *in vacuo*. At this point, a ^{1}H NMR spectrum of the crude product was taken to determine the *dr* (>99:1) of the reaction before chromatography. The crude product was then purified by column chromatography (SiO_2, 7:3 hexane:ethyl acetate) to yield the title compound (230 mg, 99%) as a colourless oil. R_f 0.32 (7:3 hexane:ethyl acetate); δ_H (400 MHz, $CDCl_3$) 8.45–8.42 (1H, m, ArH), 7.62 (1H, dd, *J* = 8.0, 1.5 Hz, ArH), 7.48–7.42 (2H, m, ArH), 7.40–7.31 (2H, m, ArH), 7.24–7.11 (4H, m, ArH), 7.06 (1H, dd, *J* = 8.0, 1.5 Hz, ArH), 6.99–6.94 (1H, m, ArH), 6.81 (1H, d, *J* = 8.0 Hz, ArH), 6.32 (1H, dd, *J* = 17.0, 2.0 Hz, H'**H**C=CH), 5.88 (1H, dd, *J* = 17.0, 10.5 Hz, H'HC=C**H**), 5.78 (1H, dd, *J* = 12.0, 4.5 Hz, NCH), 5.42 (1H, dd, *J* = 10.5, 2.0 Hz, **H'**HC=CH), 3.40 (1H, dd, *J* = 13.5, 4.5 Hz, ArC**H**H'), 3.00 (1H, dd, *J* = 13.5, 12.0 Hz, ArCH**H'**), 1.39 (9H, s, CH_3); δ_C (101 MHz, $CDCl_3$) 166.1 (CO), 158.0 (ArC), 149.2 (ArC), 148.6 (ArC), 140.4 (ArC), 137.3 (ArC), 136.0 (ArC), 132.0 (ArC), 131.2 (ArC), 130.3 (H_2C=**C**H), 129.2 (ArC), 128.7 (ArC), 128.0 (ArC), 127.5 (H_2**C**=CH), 127.3 (ArC), 126.8 (ArC), 124.0 (ArC), 121.4 (ArC), 63.2 (NCH), 42.1 ($ArCH_2$), 36.7 (**C**$(CH_3)_3$), 32.9 (C(**C**$H_3)_3$); ν_{max}/cm^{-1} (thin film) 2961, 2242, 1655, 1616, 1591, 1487, 1436, 1406, 1336, 1262, 1076, 982, 910, 747, 729, 699, 579; HRMS (ESI); calcd. for $C_{26}H_{29}N_2O^+$, 385.2274. Found: $[MH]^+$, 385.2274 (0.2 error ppm).

***N*-(2-(*tert*-Butyl)phenyl)-*N*-(1-phenyl-2-(pyridin-2-yl)ethyl)benzamide (6e)**

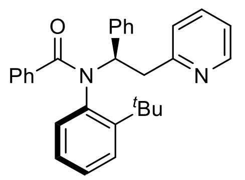

To a mixture of 2-(*tert*-butyl)-*N*-(1-phenyl-2-(pyridin-2-yl)ethyl)aniline **4a** (200 mg, 0.605 mmol) and sodium bicarbonate (203 mg, 2.42 mmol) in dry acetonitrile (3 mL) was added benzoyl chloride (0.140 mL, 170 mg, 1.21 mmol) and the resulting mixture was heated to 80 °C for 1 h. The reaction was quenched with sat. aq. $NaHCO_3$ (15 mL) and extracted with DCM (3 x 15 mL). The combined organic layers were dried over $MgSO_4$, filtered and concentrated *in vacuo*. At this point, a ^{1}H NMR spectrum of the crude product was taken to determine the *dr* (>98:2) of the reaction before chromatography. The crude product was then purified by column chromatography (SiO_2, 8:2 hexane:ethyl acetate → 7:3 hexane:ethyl acetate) to yield the title compound (240 mg, 91%) as a white solid. In solution in $CDCl_3$, the product exists as a 95:5 mixture of rotamers (amide bond); full NMR data is given for the major rotamer only. R_f 0.40 (7:3 hexane:ethyl acetate); m.p. 119–123 °C; δ_H (400 MHz, $CDCl_3$) 8.53–8.48 (1H, m, ArH), 7.62–7.57 (2H, m, ArH), 7.44–7.38 (2H, m, ArH), 7.35–7.32 (1H, m, ArH), 7.30–7.11 (8H, m, ArH), 7.09–7.01 (3H, m, ArH), 6.95 (1H, d, *J* = 8.0 Hz, ArH), 5.99 (1H, dd, *J* = 11.0, 5.0 Hz, NCH), 3.54 (1H, dd, *J* = 13.5, 5.0 Hz, C**H**H'), 3.15 (1H, dd, *J* = 13.5, 11.0 Hz, CH**H'**), 1.08 (9H, s, CH_3); δ_C (101 MHz, $CDCl_3$) 169.8 (CO), 158.4 (ArC), 149.4 (ArC), 147.8 (ArC), 140.6 (ArC), 138.4 (ArC), 137.1 (ArC), 136.2 (ArC), 132.5 (ArC), 131.5 (ArC), 129.8 (ArC), 129.6 (ArC), 129.2 (ArC), 128.2 (2 x ArC), 127.5 (ArC), 127.3 (ArC), 126.1 (ArC), 124.2 (ArC), 121.6 (ArC), 64.8 (NCH), 42.0 (CH_2), 36.4 (**C**$(CH_3)_3$), 32.4 (C(**C**$H_3)_3$); ν_{max}/cm^{-1} (thin film) 2960, 1634, 1591, 1576, 1488,

1436, 1357, 1305, 1092, 1076, 910, 732, 717, 697; HRMS (ESI); calcd. for $C_{30}H_{31}N_2O^+$, 435.2431. Found: $[MH]^+$, 435.2423 (1.9 error ppm).

Characteristic data for the minor rotamer can be found at: δ_H (400 MHz, $CDCl_3$) 8.44–8.40 (1H, m, ArH), 5.82 (1H, dd, *J* = 12.0, 4.0 Hz, NCH), 3.30 (1H, dd, *J* = 13.0, 4.0 Hz, C**H**H'), 2.72 (1H, dd, *J* = 13.0, 12.0 Hz, CH**H'**).

4-Bromo-*N*-(2-(*tert*-butyl)phenyl)-*N*-(1-phenyl-2-(pyridin-2-yl)ethyl)benzamide (6f)

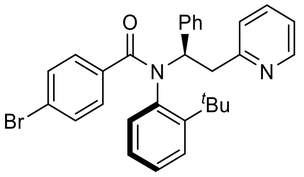

To a mixture of 2-(*tert*-butyl)-*N*-(1-phenyl-2-(pyridin-2-yl)ethyl)aniline **4a** (200 mg, 0.605 mmol) and sodium bicarbonate (203 mg, 2.42 mmol) in dry acetonitrile (3 mL) was added 4-bromobenzoyl chloride (266 mg, 1.21 mmol) and the resulting mixture was heated to 80 °C for 1 h. The reaction was quenched with sat. aq. $NaHCO_3$ (15 mL) and extracted with DCM (3 x 15 mL). The combined organic layers were dried over $MgSO_4$, filtered and concentrated *in vacuo*. At this point, a ^{1}H NMR spectrum of the crude product was taken to determine the *dr* (>95:5) of the reaction before chromatography. The crude product was then purified by column chromatography (SiO_2, 8:2 hexane:ethyl acetate) to yield the title compound (231 mg, 74%) as a white solid. In solution in $CDCl_3$, the product exists as a 95:5 mixture of rotamers (amide bond); full NMR data is given for the major rotamer only. R_f 0.26 (8:2 hexane:ethyl acetate); m.p. 49–53 °C; δ_H (400 MHz, $CDCl_3$) 8.55–8.50 (1H, m, ArH), 7.59–7.54 (2H, m, ArH), 7.44–7.34 (3H, m, ArH), 7.30–7.18 (7H, m, ArH), 7.10–7.01 (3H, m, ArH), 6.92 (1H, d, *J* = 7.5 Hz, ArH), 5.94 (1H, dd, *J* = 11.0, 5.5 Hz, NCH), 3.53 (1H, dd, *J* = 13.5, 5.5 Hz, C**H**H'), 3.13 (1H, dd, *J* = 13.5, 11.0 Hz, CH**H'**), 1.08 (9H, s, CH_3); δ_C (101 MHz, $CDCl_3$) 168.8 (CO), 158.3 (ArC), 149.5 (ArC), 147.9 (ArC), 140.4 (ArC), 138.2 (ArC), 136.2 (ArC), 136.0 (ArC), 132.3 (ArC), 131.7 (ArC), 131.4 (ArC), 130.6 (ArC), 129.1 (ArC), 128.4 (ArC), 128.3 (ArC), 127.6 (ArC), 126.3 (ArC), 124.2 (ArC), 124.1 (ArC), 121.6 (ArC), 65.1 (NCH), 42.0 (CH_2), 36.4 (**C**(CH_3)$_3$), 32.5 (C(**C**H_3)$_3$); ν_{max}/cm^{-1} (thin film) 2961, 1634, 1589, 1487, 1436, 1359, 1304, 1073, 1010, 909, 838, 751, 731, 705; HRMS (ESI); calcd. for $C_{30}H_{30}{}^{79}BrN_2O^+$, 513.1536. Found: $[MH]^+$, 513.1515 (4.0 error ppm).

Characteristic data for the minor rotamer can be found at: δ_H (400 MHz, $CDCl_3$) 8.45–8.41 (1H, m, ArH), 5.73 (1H, dd, *J* = 11.5, 4.0 Hz, NCH), 3.24 (1H, dd, *J* = 13.0, 4.0 Hz, C**H**H'), 2.68 (1H, dd, *J* = 13.0, 11.5 Hz, CH**H'**).

***N*-(2-(*tert*-Butyl)phenyl)-*N*-(1-phenyl-2-(pyridin-2-yl)ethyl)-3-(trifluoromethyl)benzamide (6g)**

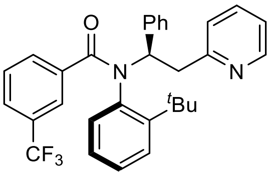

To a mixture of 2-(*tert*-butyl)-*N*-(1-phenyl-2-(pyridin-2-yl)ethyl)aniline **4a** (200 mg, 0.605 mmol) and sodium bicarbonate (203 mg, 2.42 mmol) in dry acetonitrile (3 mL) was added 3-(trifluoromethyl)benzoyl chloride (252 mg, 1.21 mmol) and the resulting mixture was heated to 80 °C for 1 h. After this time the reaction was deemed incomplete by TLC and so 3-(trifluoromethyl)benzoyl chloride (126 mg, 0.605 mmol) was added and the mixture stirred for a further 1 h. After this time the reaction was deemed incomplete by TLC and so 3-(trifluoromethyl)benzoyl chloride (126 mg, 0.605 mmol) was added and the mixture stirred for a further 1 h (3 h total reaction time). The reaction was quenched with sat. aq. $NaHCO_3$ (15 mL) and extracted with DCM (3 x 15 mL). The combined organic layers were dried over $MgSO_4$, filtered and concentrated *in vacuo*. At this point, a ^{1}H NMR spectrum of the crude product was taken to determine the *dr* (>95:5) of the reaction before chromatography. The crude product was then purified by column chromatography (SiO_2, 8:2 hexane:ethyl acetate → 6:4 hexane:ethyl acetate) to yield the title compound (234 mg, 77%) as a yellow oil. In solution in $CDCl_3$, the product exists as a 95:5 mixture of rotamers (amide bond); full NMR data is given for the major rotamer only. R_f 0.23 (8:2 hexane:ethyl acetate); δ_H (400 MHz, $CDCl_3$) 8.59–8.53 (1H, m, ArH), 7.64–7.57 (2H, m, ArH), 7.49–7.16 (12H, m, ArH), 7.05 (1H, dd, *J* = 7.5, 5.0 Hz, ArH), 6.94 (1H, d, *J* = 8.0 Hz, ArH), 5.96 (1H, dd, *J* = 11.0, 5.5 Hz, NCH), 3.59 (1H, dd, *J* = 13.5, 5.5 Hz, C**H**H'), 3.21 (1H, dd, *J* = 13.5, 11.0 Hz, CH**H'**), 1.06 (9H, s, CH_3); δ_C (101 MHz, $CDCl_3$) 168.2 (CO), 158.0 (ArC), 149.2 (ArC), 147.7 (ArC), 140.1 (ArC), 137.9 (ArC), 137.7 (ArC), 136.4 (ArC), 133.0 (ArC), 132.2 (ArC), 131.7 (ArC), 129.7 (ArC, q, $^2J_{CF}$ = 32.6 Hz), 129.1 (ArC), 128.6 (ArC), 128.3 (ArC), 127.9 (ArC), 127.6 (ArC), 126.7 (ArC, q, $^3J_{CF}$ = 3.8 Hz), 126.4 (ArC), 126.1 (ArC, q, $^3J_{CF}$ = 3.4 Hz), 124.3 (ArC), 123.6 (CF_3, q, $^1J_{CF}$ = 274.7), 121.7 (ArC), 65.3 (NCH), 41.7 (CH_2), 36.3 (**C**(CH_3)$_3$), 32.4 (C(**C**H_3)$_3$); δ_F (376 MHz, $CDCl_3$) –62.8 (3F, s, CF_3); v_{max}/cm^{-1} (thin film) 2963, 1636, 1590, 1488, 1436, 1325, 1301, 1278, 1168, 1127, 1073, 907, 728, 698, 554; HRMS (ESI); calcd. for $C_{31}H_{30}F_3N_2O^+$, 503.2305. Found: $[MH]^+$, 503.2310 (–1.0 error ppm).

Characteristic data for the minor rotamer can be found at: δ_H (400 MHz, $CDCl_3$) 5.66 (1H, dd, *J* = 11.5, 4.0 Hz, NCH), 2.66 (1H, dd, *J* = 13.0, 11.5 Hz, C**H**H'), 1.64 (9H, s, C(CH_3)$_3$).

***N*-(2-(*tert*-Butyl)phenyl)-2,4,6-trifluoro-*N*-(1-phenyl-2-(pyridin-2-yl)ethyl)benzamide (6h)**

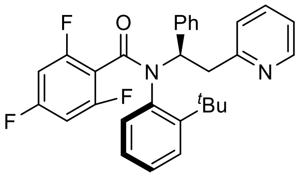

To a mixture of 2-(*tert*-butyl)-*N*-(1-phenyl-2-(pyridin-2-yl)ethyl)aniline **4a** (200 mg, 0.605 mmol) and sodium bicarbonate (203 mg, 2.42 mmol) in dry acetonitrile (3 mL) was added 2,4,6-trifluorobenzoyl chloride (235 mg, 1.21 mmol) and the resulting mixture was heated to 80 °C for 1 h. After this time the reaction was deemed incomplete by TLC and so 2,4,6-trifluorobenzoyl chloride (118 mg, 0.605 mmol) was added and the reaction mixture was stirred for a further 1 h. The reaction was quenched with sat. aq. $NaHCO_3$ (15 mL) and extracted with DCM (3 x 15 mL). The combined organic layers were dried over $MgSO_4$, filtered and concentrated *in vacuo*. At this point, a ^{1}H NMR spectrum of the crude product was taken

to determine the *dr* (>95:5) of the reaction before chromatography. The crude product was then purified by column chromatography (SiO_2, 8:2 hexane:ethyl acetate) to yield the title compound (253 mg, 86%) as a 74:26 mixture of rotamers and as a white solid. R_f 0.25 (8:2 hexane:ethyl acetate); δ_H (400 MHz, $CDCl_3$) 8.46–8.42 (1H, m, ArH, major rotamer), 8.39–8.36 (1H, m, ArH, minor rotamer), 7.74–7.65 (2H, m, ArH), 7.53–7.14 (15H, m, ArH), 7.04–6.87 (3H, m, ArH), 6.83–6.78 (2H, m, ArH), 6.71–6.59 (2H, m, ArH), 6.54–6.44 (1H, m, ArH), 6.38–6.26 (2H, m, ArH), 6.20–6.12 (1H, m, ArH), 5.80 (1H, dd, *J* = 12.5, 4.0 Hz, NCH, major rotamer), 5.46 (1H, dd, *J* = 12.0, 4.0 Hz, NCH, minor rotamer), 3.29 (1H, dd, *J* = 12.5, 4.0 Hz, C**H**H', minor rotamer), 3.23 (1H, dd, *J* = 13.0, 4.0 Hz, C**H**H', major rotamer), 2.88 (1H, dd, *J* = 13.0, 12.5 Hz, CH**H'**, major rotamer), 2.59 (1H, dd, *J* = 12.5, 12.0 Hz, CH**H'**, minor rotamer), 1.63 (9H, s, CH_3, minor rotamer), 1.49 (9H, s, CH_3, major rotamer); δ_C (101 MHz, $CDCl_3$) [Due to the combination of the fluorine atoms causing peak splitting and the presence of rotamers, it was not possible to assign all peaks in the aromatic region. Peaks that could be identified have been listed.] 164.1 (CO), 163.9 (CO), 161.7 (ArC), 161.6 (ArC), 157.6 (ArC), 157.0 (ArC), 149.4 (ArC), 149.3 (ArC), 149.0 (ArC), 148.8 (ArC), 141.0 (ArC), 140.4 (ArC), 136.8 (ArC), 135.9 (ArC), 135.7 (ArC), 135.4 (ArC), 132.6 (ArC), 132.0 (ArC), 131.3 (ArC), 130.2 (ArC), 129.0 (ArC), 128.8 (ArC), 128.7 (ArC), 128.0 (ArC), 127.9 (ArC), 127.3 (ArC), 127.2 (ArC), 127.1 (ArC), 126.9 (ArC), 126.0 (ArC), 124.1 (ArC), 124.0 (ArC), 121.4 (ArC), 121.4 (ArC), 100.2 (ArC), 65.2 (NCH, minor rotamer), 64.7 (NCH, major rotamer), 45.1 (CH_2, minor rotamer), 43.0 (CH_2, major rotamer), 36.8 (**C**(CH_3)$_3$, major rotamer), 36.4 (**C**(CH_3)$_3$, minor rotamer), 32.8 (C(**C**H_3)$_3$, major rotamer), 32.2 (C(**C**H_3)$_3$, minor rotamer); δ_F (376 MHz, $CDCl_3$) –103.0 (1F, br m, ArF, major rotamer), –104.9 (1F, apparent t (broad), *J* = 8.5 Hz, ArF, minor rotamer), –105.0 (1F, br m, ArF, major rotamer), –105.7 (1F, apparent p (broad), *J* = 8.5 Hz, ArF, major rotamer), –106.3 (1F, apparent p (broad), *J* = 8.0 Hz, ArF, minor rotamer), –108.2 (1F, apparent t (broad), *J* = 8.5 Hz, ArF, minor rotamer); ν_{max}/cm^{-1} (thin film) 2963, 1651, 1638, 1599, 1488, 1437, 1365, 1302, 1120, 1036, 999, 910, 841, 728, 547; HRMS (ESI); calcd. for $C_{30}H_{28}F_3N_2O^+$, 489.2148. Found: $[MH]^+$, 489.2156 (–1.5 error ppm).

***N*-(2-(*tert*-Butyl)phenyl)-4-nitro-*N*-(1-phenyl-2-(pyridin-2-yl)ethyl)benzamide (6i)**

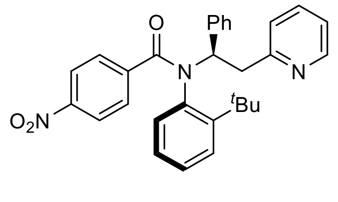

To a mixture of 2-(*tert*-butyl)-*N*-(1-phenyl-2-(pyridin-2-yl)ethyl)aniline **4a** (200 mg, 0.605 mmol) and sodium bicarbonate (203 mg, 2.42 mmol) in dry acetonitrile (3 mL) was added 4-nitrobenzoyl chloride (225 mg, 1.21 mmol) and the resulting mixture was heated to 80 °C for 1 h. The reaction was quenched with sat. aq. $NaHCO_3$ (15 mL) and extracted with DCM (3 x 15 mL). The combined organic layers were dried over $MgSO_4$, filtered and concentrated *in vacuo*. At this point, a ^1H NMR spectrum of the crude product was taken to determine the *dr* (>95:5) of the reaction before chromatography. The crude product was then purified by column chromatography (SiO_2, 8:2 hexane:ethyl acetate → 6:4 hexane:ethyl acetate) to yield the title compound (143 mg, 49%) as a white solid. In solution in $CDCl_3$, the product exists as a 94:6 mixture of rotamers (amide bond); full NMR data is given for the major rotamer only. R_f 0.38

(6:4 hexane:ethyl acetate); m.p. 48–50 °C; δ_H (400 MHz, $CDCl_3$) 8.56–8.52 (1H, m, ArH), 7.95–7.90 (2H, m, ArH), 7.60–7.54 (2H, m, ArH), 7.46–7.33 (5H, m, ArH), 7.30–7.19 (5H, m, ArH), 7.07–7.02 (1H, m, ArH), 6.90 (1H, d, *J* = 8.0 Hz, ArH), 5.94 (1H, dd, *J* = 11.0, 5.5 Hz, NCH), 3.54 (1H, dd, *J* = 13.5, 5.5 Hz, C**H**H'), 3.14 (1H, dd, *J* = 13.5, 11.0 Hz, CH**H'**), 1.10 (9H, s, CH_3); δ_C (101 MHz, $CDCl_3$) 167.7 (CO), 158.0 (ArC), 149.5 (ArC), 148.0 (ArC), 147.7 (ArC), 143.1 (ArC), 140.0 (ArC), 137.5 (ArC), 136.2 (ArC), 132.3 (ArC), 131.8 (ArC), 130.6 (ArC), 129.1 (ArC), 128.9 (ArC), 128.3 (ArC), 127.8 (ArC), 126.5 (ArC), 124.2 (ArC), 122.5 (ArC), 121.7 (ArC), 65.3 (NCH), 41.9 (CH_2), 36.4 (**C**(CH_3)$_3$), 32.6 (C(**C**H_3)$_3$); ν_{max}/cm^{-1} (thin film) 2961, 1637, 1595, 1521, 1487, 1436, 1343, 1305, 1092, 1051, 908, 864, 723, 699, 557; HRMS (ESI); calcd. for $C_{30}H_{30}N_3O_3^+$, 480.2282. Found: $[MH]^+$, 480.2261 (4.2 error ppm).

Characteristic data for the minor rotamer can be found at: δ_H (400 MHz, $CDCl_3$) 8.46–8.42 (1H, m, ArH), 5.65 (1H, dd, *J* = 11.5, 4.0 Hz, NCH), 2.64 (1H, dd, *J* = 13.0, 11.5 Hz, C**H**H').

***N*-(2-(*tert*-Butyl)phenyl)-3,5-dinitro-*N*-(1-phenyl-2-(pyridin-2-yl)ethyl)benzamide (6j)**

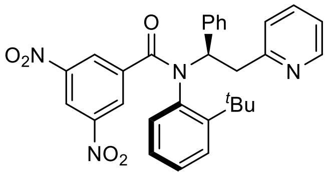

To a mixture of 2-(*tert*-butyl)-*N*-(1-phenyl-2-(pyridin-2-yl)ethyl)aniline **4a** (200 mg, 0.605 mmol) and sodium bicarbonate (203 mg, 2.42 mmol) in dry acetonitrile (3 mL) was added 3,5-dinitrobenzoyl chloride (279 mg, 1.21 mmol) and the resulting mixture was heated to 80 °C for 24 h. The reaction was quenched with sat. aq. $NaHCO_3$ (15 mL) and extracted with DCM (3 x 15 mL). The combined organic layers were dried over $MgSO_4$, filtered and concentrated *in vacuo*. At this point, a ^1H NMR spectrum of the crude product was taken to determine the *dr* (>95:5) of the reaction before chromatography. The crude product was then purified by column chromatography (SiO_2, 8:2 hexane:ethyl acetate) to yield the title compound (21 mg, 7%) as a yellow oil. R_f 0.24 (8:2 hexane:ethyl acetate); δ_H (400 MHz, $CDCl_3$) 8.84 (1H, t, *J* = 2.0 Hz, ArH), 8.59–8.56 (1H, m, ArH), 8.36 (2H, d, *J* = 2.0 Hz, ArH), 7.60–7.55 (2H, m, ArH), 7.53–7.49 (1H, m, ArH), 7.46–7.23 (7H, m, ArH), 7.11–7.06 (1H, m, ArH), 6.90 (1H, d, *J* = 8.0 Hz, ArH), 5.90 (1H, dd, *J* = 10.5, 5.5 Hz, NCH), 3.61 (1H, dd, *J* = 13.5, 5.5 Hz, C**H**H'), 3.19 (1H, dd, *J* = 13.5, 10.5 Hz, CH**H'**), 1.10 (9H, s, CH_3); δ_C (101 MHz, $CDCl_3$) 164.9 (CO), 157.7 (ArC), 149.7 (ArC), 147.9 (ArC), 147.6 (ArC), 140.5 (ArC), 139.5 (ArC), 137.2 (ArC), 136.4 (ArC), 132.1 (ArC), 131.9 (ArC), 129.7 (ArC), 129.6 (ArC), 129.2 (ArC), 128.5 (ArC), 128.1 (ArC), 127.4 (ArC), 124.3 (ArC), 121.9 (ArC), 119.4 (ArC), 66.3 (NCH), 41.9 (CH_2), 36.5 (**C**(CH_3)$_3$), 32.7 (C(**C**H_3)$_3$); ν_{max}/cm^{-1} (thin film) 2961, 1644, 1590, 1541, 1436, 1341, 1305, 1077, 911, 764, 728, 700; HRMS (ESI); calcd. for $C_{30}H_{29}N_4O_5^+$, 525.2132. Found: $[MH]^+$, 525.2140 (–1.4 error ppm).

***N*-(2-(*tert*-Butyl)phenyl)-2-chloro-*N*-(1-phenyl-2-(pyridin-2-yl)ethyl)benzamide (6k)**

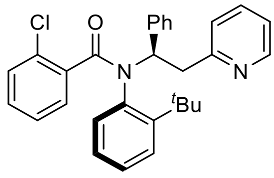

To a mixture of 2-(*tert*-butyl)-*N*-(1-phenyl-2-(pyridin-2-yl)ethyl)aniline **4a** (200 mg, 0.605 mmol) and sodium bicarbonate (203 mg, 2.42 mmol) in dry acetonitrile (3 mL) was added 2-chlorobenzoyl chloride (0.150 mL, 212 mg, 1.21 mmol) and the resulting mixture was heated to 80 °C for 1 h. The reaction was quenched with sat. aq. $NaHCO_3$ (15 mL) and extracted with DCM (3 x 15 mL). The combined organic layers were dried over $MgSO_4$, filtered and concentrated *in vacuo*. At this point, a ^{1}H NMR spectrum of the crude product was taken to determine the *dr* (>95:5) of the reaction before chromatography. The crude product was then purified by column chromatography (SiO_2, 7:3 hexane:ethyl acetate) to yield the title compound (183 mg, 64%) as a 64:36 mixture of rotamers and as a white solid. R_f 0.27 (7:3 hexane:ethyl acetate); m.p. 42–46 °C; δ_H (400 MHz, $CDCl_3$) 8.47–8.43 (1H, m, ArH, major rotamer), 8.38 (1H, d, *J* = 4.5 Hz, ArH, minor rotamer), 7.70–7.54 (4H, m, ArH), 7.45–6.83 (25H, m, ArH), 6.76 (1H, d, *J* = 7.5 Hz, ArH, major rotamer), 6.66 (1H, d, *J* = 7.5 Hz, ArH, major rotamer), 6.31 (1H, d, *J* = 7.5 Hz, ArH, minor rotamer), 5.91 (1H, dd, *J* = 12.5, 4.0 Hz, NCH, major rotamer), 5.47 (1H, dd, *J* = 12.0, 4.0 Hz, NCH, minor rotamer), 3.40 (2H, m, C**H**H', both rotamers), 2.97 (1H, dd, *J* = 13.0, 12.5 Hz, CH**H'**, major rotamer), 2.68 (1H, dd, *J* = 12.5, 12.0 Hz, CH**H'**, minor rotamer), 1.69 (9H, s, CH_3, minor rotamer), 1.52 (9H, s, CH_3, major rotamer); δ_C (101 MHz, $CDCl_3$) 169.4 (CO, minor rotamer), 167.7 (CO, major rotamer), 157.8 (ArC), 157.2 (ArC), 149.4 (ArC), 149.2 (ArC), 148.3 (ArC), 148.1 (ArC), 139.9 (ArC), 139.6 (ArC), 137.0 (ArC), 136.7 (ArC), 136.6 (ArC), 136.3 (ArC), 135.9 (ArC), 135.8 (ArC), 132.9 (ArC), 132.8 (ArC), 131.9 (ArC), 131.6 (ArC), 131.1 (ArC), 130.4 (ArC), 130.3 (ArC), 129.9 (ArC), 129.8 (ArC), 129.5 (2 x ArC), 128.7 (ArC), 128.5 (ArC), 128.4 (ArC), 128.3 (ArC), 127.8 (2 x ArC), 127.4 (ArC), 127.2 (ArC), 126.7 (ArC), 126.4 (ArC), 126.1 (ArC), 125.7 (ArC), 125.5 (ArC), 124.1 (ArC), 123.9 (ArC), 121.4 (ArC), 121.2 (ArC), 65.5 (NCH, minor rotamer), 63.6 (NCH, major rotamer), 44.4 (CH_2, minor rotamer), 42.2 (CH_2, major rotamer), 37.1 (**C**(CH_3)$_3$, minor rotamer), 36.6 (**C**(CH_3)$_3$, major rotamer), 33.1 (C(**C**H_3)$_3$, major rotamer), 32.7 (C(**C**H_3)$_3$, minor rotamer); ν_{max}/cm^{-1} (thin film) 2962, 1643, 1591, 1487, 1473, 1434, 1361, 1304, 1153, 1041, 909, 727, 699, 644, 554; HRMS (ESI); calcd. for $C_{30}H_{30}{}^{35}ClN_2O^+$, 469.2041. Found: [MH]$^+$, 469.2029 (2.5 error ppm).

_N_-(2-(_tert_-Butyl)phenyl)-_N_-(1-phenyl-2-(pyridin-2-yl)ethyl)-2-naphthamide (6l)

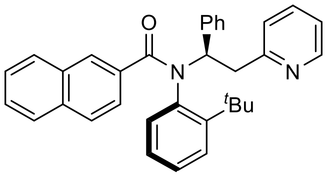

To a mixture of 2-(*tert*-butyl)-*N*-(1-phenyl-2-(pyridin-2-yl)ethyl)aniline **4a** (200 mg, 0.605 mmol) and sodium bicarbonate (203 mg, 2.42 mmol) in dry acetonitrile (3 mL) was added 2-naphthoyl chloride (231 mg, 1.21 mmol) and the resulting mixture was heated to 80 °C for 1 h. The reaction was quenched with sat. aq. $NaHCO_3$ (15 mL) and extracted with DCM (3 x 15 mL). The combined organic layers were dried over $MgSO_4$, filtered and concentrated *in vacuo*. At this point, a ^{1}H NMR spectrum of the crude product was taken to determine the *dr* (>95:5) of the reaction before chromatography. The crude product was then purified by column chromatography (SiO_2, 8:2 hexane:ethyl acetate → 7:3 hexane:ethyl acetate) to yield the title compound (191 mg, 65%) as a white solid. R_f 0.15 (8:2 hexane:ethyl acetate); m.p. 63–66 °C; δ_H (400 MHz, $CDCl_3$) 8.58–8.53 (1H, m, ArH), 7.70–7.61 (4H, m, ArH), 7.59–7.51 (3H, m, ArH),

7.45–7.20 (10H, m, ArH), 7.07–7.02 (1H, m, ArH), 6.98 (1H, d, *J* = 7.5 Hz, ArH), 6.06 (1H, dd, *J* = 11.0, 5.5 Hz, NCH), 3.61 (1H, dd, *J* = 13.5, 5.5 Hz, C**H**H'), 3.22 (1H, dd, *J* = 13.5, 11.5 Hz, CH**H'**), 1.07 (9H, s, CH_3); δ_C (101 MHz, $CDCl_3$), 169.7 (CO), 158.4 (ArC), 149.4 (ArC), 147.9 (ArC), 140.6 (ArC), 138.5 (ArC), 136.2 (ArC), 134.2 (ArC), 133.4 (ArC), 132.5 (ArC), 132.0 (ArC), 131.6 (ArC), 130.4 (ArC), 129.2 (ArC), 128.8 (ArC), 128.2 (2 x ArC), 127.5 (ArC), 127.4 (ArC), 127.2 (ArC), 126.9 (ArC), 126.6 (ArC), 126.1 (2 x ArC), 124.3 (ArC), 121.6 (ArC), 64.9 (NCH), 42.0 (CH_2), 36.4 (**C**(CH_3)$_3$), 32.5 (C(**C**H_3)$_3$); v_{max}/cm^{-1} (thin film) 2962, 1635, 1624, 1592, 1487, 1435, 1386, 1303, 1200, 1089, 907, 758, 726, 699, 476; HRMS (ESI); calcd. for $C_{34}H_{33}N_2O^+$, 485.2587. Found: $[MH]^+$, 485.2563 (5.1 error ppm).

***N*-(2-(*tert*-Butyl)phenyl)-4-methoxy-*N*-(1-phenyl-2-(pyridin-2-yl)ethyl)benzamide (6m)**

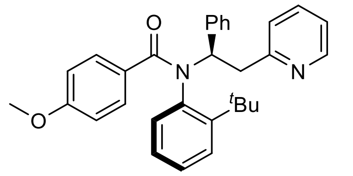

To a mixture of 2-(*tert*-butyl)-*N*-(1-phenyl-2-(pyridin-2-yl)ethyl)aniline **4a** (200 mg, 0.605 mmol) and sodium bicarbonate (203 mg, 2.42 mmol) in dry acetonitrile (3 mL) was added 4-methoxybenzoyl chloride (310 mg, 1.82 mmol) and the resulting mixture was heated to 80 °C for 1 h. The reaction was quenched with sat. aq. $NaHCO_3$ (15 mL) and extracted with DCM (3 x 15 mL). The combined organic layers were dried over $MgSO_4$, filtered and concentrated *in vacuo*. At this point, a ^{1}H NMR spectrum of the crude product was taken to determine the *dr* (97:3) of the reaction before chromatography. The crude product was then purified by column chromatography (SiO_2, 8:2 hexane:ethyl acetate → 6:4 hexane:ethyl acetate) to yield the title compound (213 mg, 76%) as a white solid. R_f 0.30 (6:4 hexane:ethyl acetate); m.p. 55–57 °C; δ_H (400 MHz, $CDCl_3$) 8.53–8.50 (1H, m, ArH), 7.60–7.54 (2H, m, ArH), 7.43–7.33 (3H, m, ArH), 7.29–7.13 (7H, m, ArH), 7.02 (1H, dd, *J* = 7.5, 5.0 Hz, ArH), 6.94 (1H, d, *J* = 8.0 Hz, ArH), 6.58–6.53 (2H, m, ArH), 5.98 (1H, dd, *J* = 11.0, 5.0 Hz, NCH), 3.67 (3H, s, OCH_3), 3.52 (1H, dd, *J* = 13.5, 5.0 Hz, C**H**H'), 3.14 (1H, dd, *J* = 13.5, 11.0 Hz, CH**H'**), 1.07 (9H, s, C(CH_3)$_3$); δ_C (101 MHz, $CDCl_3$) 169.3 (CO), 130.4 (ArC), 158.5 (ArC), 149.4 (ArC), 147.8 (ArC), 140.7 (ArC), 138.7 (ArC), 136.1 (ArC), 132.3 (ArC), 131.7 (ArC), 131.5 (ArC), 129.4 (ArC), 129.1 (ArC), 128.1 (ArC), 128.0 (ArC), 127.3 (ArC), 126.1 (ArC), 124.2 (ArC), 121.5 (ArC), 112.5 (ArC), 64.7 (NCH), 55.2 (OCH_3), 42.0 (CH_2), 36.4 (**C**(CH_3)$_3$), 32.4 (C(**C**H_3)$_3$); v_{max}/cm^{-1} (thin film) 2960, 1628, 1603, 1570, 1510, 1435, 1355, 1303, 1252, 1175, 1030, 909, 840, 763, 727, 699, 552; HRMS (ESI); calcd. for $C_{31}H_{33}N_2O_2^+$, 465.2537. Found: $[MH]^+$, 465.2546 (–1.9 error ppm).

***N*-(2-(*tert*-Butyl)phenyl)-4-methoxy-*N*-(1-phenyl-2-(pyridin-2-yl)ethyl)benzamide (11m)**

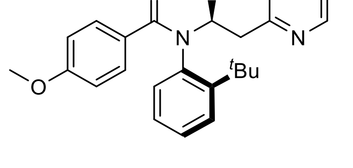

To a mixture of 2-(*tert*-butyl)-*N*-(1-phenyl-2-(pyridin-2-yl)ethyl)aniline **4a** (200 mg, 0.605 mmol) and sodium bicarbonate (203 mg, 2.42 mmol) in dry acetonitrile (3 mL) was added 4-

methoxybenzoyl chloride (310 mg, 1.82 mmol) and the resulting mixture was heated to 80 °C for 24 h. The reaction was quenched with sat. aq. $NaHCO_3$ (15 mL) and extracted with DCM (3 x 15 mL). The combined organic layers were dried over $MgSO_4$, filtered and concentrated *in vacuo*. At this point, a ^{1}H NMR spectrum of the crude product was taken to determine the *dr* (83:17 **6m**:**11m**) of the reaction before chromatography. The crude product was then purified by column chromatography (SiO_2, 8:2 hexane:ethyl acetate → 6:4 hexane:ethyl acetate) to yield the title compound (47 mg, 17%) as a gummy white solid. R_f 0.05 (8:2 hexane:ethyl acetate); δ_H (400 MHz, $CDCl_3$) 8.38 (1H, d, *J* = 5.0 Hz, ArH), 7.66–7.60 (1H, m, ArH), 7.52–7.45 (1H, m, ArH), 7.32–7.20 (6H, m, ArH), 7.08–6.90 (6H, m, ArH), 6.64–6.57 (2H, m, ArH), 6.26 (1H, dd, *J* = 10.0, 5.0 Hz, NCH), 3.97 (1H, dd, *J* = 13.0, 5.0 Hz, C**H**H'), 3.76–3.66 (4H, m, CH**H'**, OCH_3), 0.78 (9H, s, $C(CH_3)_3$); δ_C (101 MHz, $CDCl_3$) 169.0 (CO), 160.3 (ArC), 158.6 (ArC), 148.9 (ArC), 147.9 (ArC), 137.6 (ArC), 137.2 (ArC), 136.2 (ArC), 133.4 (ArC), 131.5 (ArC), 131.4 (ArC), 131.2 (ArC), 129.6 (ArC), 128.4 (ArC), 127.8 (2 x ArC), 125.9 (ArC), 124.1 (ArC), 121.4 (ArC), 112.6 (ArC), 64.6 (NCH), 55.2 (OCH_3), 44.8 (CH_2), 36.3 (**C**$(CH_3)_3$), 32.1 (C(**C**$H_3)_3$); ν_{max}/cm^{-1} (thin film) 2957, 1730, 1626, 1605, 1510, 1489, 1435, 1339, 1304, 1252, 1176, 1031, 910, 840, 760, 729, 701, 610; HRMS (ESI); calcd. for $C_{31}H_{33}N_2O_2^+$, 465.2537. Found: $[MH]^+$, 465.2542 (–1.3 error ppm).

***N*-(2-(*tert*-Butyl)phenyl)-2-(1H-indol-3-yl)-2-oxo-*N*-(1-phenyl-2-(pyridin-2-yl)ethyl)acetamide (6n)**

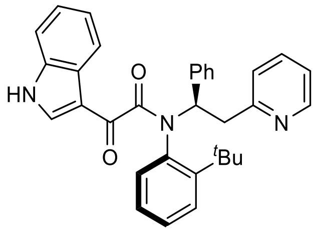

To a mixture of 2-(*tert*-butyl)-*N*-(1-phenyl-2-(pyridin-2-yl)ethyl)aniline **4a** (200 mg, 0.605 mmol) and sodium bicarbonate (203 mg, 2.42 mmol) in dry acetonitrile (3 mL) was added 3-indoleglyoxylyl chloride (251 mg, 1.21 mmol) and the resulting mixture was heated to 80 °C for 1 h. The reaction was quenched with sat. aq. $NaHCO_3$ (15 mL) and extracted with DCM (3 x 15 mL). The combined organic layers were dried over $MgSO_4$, filtered and concentrated *in vacuo*. At this point, a ^{1}H NMR spectrum of the crude product was taken to determine the *dr* (>97:3) of the reaction before chromatography. The crude product was then purified by column chromatography (SiO_2, 6:4 hexane:ethyl acetate) to yield the title compound (265 mg, 87%) as a 67:37 mixture of rotamers and as a yellow solid. R_f 0.22 (6:4 hexane:ethyl acetate); m.p. 126–129 °C; δ_H (400 MHz, $CDCl_3$) 10.17–10.08 (2H, m, NH, both rotamers), 8.47–8.43 (1H, m, ArH, major rotamer), 8.33–8.29 (1H, m, ArH, minor rotamer), 8.13 (1H, d, *J* = 8.0 Hz, ArH, minor rotamer), 7.82–7.68 (2H, m, ArH), 7.56 (1H, dd, *J* = 8.0, 1.5 Hz, ArH, major rotamer), 7.50–7.83 (26H, m, ArH), 6.74 (1H, d, *J* = 8.0 Hz, ArH, major rotamer), 6.60–6.52 (2H, m, ArH), 6.42 (1H, t, *J* = 7.5 Hz, ArH, minor rotamer), 5.93–5.83 (2H, m, NCH, both rotamers), 3.36 (1H, dd, *J* = 13.0, 4.0 Hz, C**H**H', major rotamer), 3.16 (1H, dd, *J* = 12.5, 4.0 Hz, C**H**H', minor rotamer), 2.83 (1H, dd, *J* = 13.0, 13.0 Hz, CH**H'**, major rotamer), 2.71 (1H, dd, *J* = 12.5, 12.5 Hz, CH**H'**, minor rotamer), 1.65–1.59 (18H, m, CH_3, both rotamers); δ_C (101 MHz, $CDCl_3$) 185.2 (CO, minor rotamer), 184.6 (CO, major rotamer), 169.1 (CO, minor rotamer), 167.7 (CO, major

rotamer), 157.4 (ArC), 157.1 (ArC), 149.6 (ArC), 149.3 (ArC), 149.1 (ArC), 149.0 (ArC), 140.4 (ArC), 139.8 (ArC), 136.4 (ArC), 136.2 (ArC), 136.0 (2 x ArC), 134.7 (ArC), 134.6 (ArC), 132.2 (ArC), 132.1 (ArC), 131.3 (ArC), 130.1 (ArC), 129.3 (ArC), 129.2 (ArC), 129.1 (ArC), 128.9 (ArC), 128.1 (ArC), 127.7 (ArC), 126.9 (ArC), 126.1 (ArC), 125.7 (ArC), 125.1 (ArC), 124.1 (ArC), 123.9 (ArC), 123.5 (ArC), 123.4 (ArC), 122.6 (ArC), 122.5 (ArC), 121.7 (ArC), 121.6 (ArC), 121.5 (ArC), 114.8 (ArC), 113.6 (ArC), 112.0 (ArC), 111.9 (ArC), 64.7 (NCH, minor rotamer), 62.7 (NCH, major rotamer), 44.7 (CH_2, minor rotamer), 41.4 (CH_2, major rotamer), 37.2 (**C**(CH_3)$_3$, major rotamer), 36.8 (**C**(CH_3)$_3$, minor rotamer), 32.9 (C(**C**H_3)$_3$major rotamer), 32.3 (C(**C**H_3)$_3$), minor rotamer); v_{max}/cm^{-1} (thin film) 3242, 2961, 1617, 1595, 1517, 1487, 1436, 1406, 1352, 1300, 1244, 1154, 1134, 1032, 908, 749, 729, 705; HRMS (ESI); calcd. for $C_{33}H_{32}N_3O_2^+$, 502.2489. Found: [MH]$^+$, 502.2504 (–3.1 error ppm).

(4*R*)-*N*-(2-(*tert*-Butyl)phenyl)-4-((5*S*,8*R*,9*S*,10*S*,13*R*,14*S*,17*R*)-10,13-dimethyl-3,7,12-trioxohexadecahydro-1*H*-cyclopenta[a]phenanthren-17-yl)-*N*-(1-phenyl-2-(pyridin-2-yl)ethyl)pentanamide (6o)

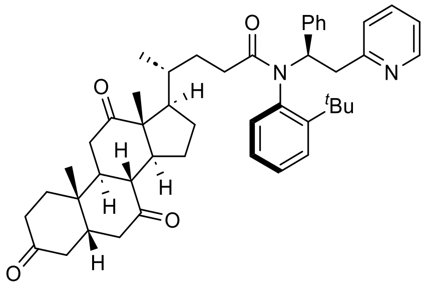

To a mixture of 2-(*tert*-butyl)-*N*-(1-phenyl-2-(pyridin-2-yl)ethyl)aniline **4a** (200 mg, 0.605 mmol) and sodium bicarbonate (203 mg, 2.42 mmol) in dry acetonitrile (3 mL) was added dehydrocholic acid chloride (509 mg, 1.21 mmol) and the resulting mixture was heated to 80 °C for 1 h. The reaction was quenched with sat. aq. $NaHCO_3$ (15 mL) and extracted with DCM (3 x 15 mL). The combined organic layers were dried over $MgSO_4$, filtered and concentrated *in vacuo*. At this point, a ^{1}H NMR spectrum of the crude product was taken to determine the *dr* (>95:5) of the reaction before chromatography with respect to the atropisomeric amide portion. The crude product was then purified by column chromatography (SiO_2, 6:4 hexane:ethyl acetate) to yield the title compound (209 mg, 48%) as a 1:1 mixture of diastereoisomers and as a white solid. R_f 0.28 (6:4 hexane:ethyl acetate); m.p. 102–106 °C; δ_H (400 MHz, $CDCl_3$) 8.44–8.38 (2H, m, ArH, both diastereoisomers), 7.63–7.57 (2H, m, ArH, both diastereoisomers), 7.44–7.30 (8H, m, ArH), 7.24–7.09 (8H, m, ArH), 7.04–6.92 (4H, m, ArH), 6.81–6.76 (2H, m, ArH, both diastereoisomers), 5.70 (2H, dd, *J* = 12.0, 4.5 Hz, NCH, both diastereoisomers), 7.39–7.29 (2H, m, ArC**H**H′, both diastereoisomers), 2.98–2.72 (8H, m, 6 x CH, ArCHH′, both diastereoisomers), 2.37–1.50 (32H, m, CH), 1.41 (18H, s, C(CH_3)$_3$, both diastereoisomers), 1.37 (6H, s, CH_3, both diastereoisomers), 1.22–1.05 (8H, m, CH), 1.00–0.82 (8H, m, 2 x CH, CH_3, both diastereoisomers), 0.60–0.50 (6H, m, CH_3, both diastereoisomers); δ_C (101 MHz, $CDCl_3$) [Due to significant overlap of peaks of the different diastereoisomers, it was not possible to fully assign the peaks of both diastereoisomers.] 212.1 (2 x CO, both diastereoisomers), 209.2 (2 x CO, both diastereoisomers), 208.8 (2 x CO, both diastereoisomers), 173.8 (CO), 173.7 (CO), 158.2 (2 x ArC), 149.3 (ArC), 147.9 (ArC), 147.6

(ArC), 140.8 (ArC), 136.0 (ArC), 132.1 (ArC), 132.0 (ArC), 131.3 (ArC), 131.2 (ArC), 129.2 (2 x ArC), 128.6 (2 x ArC), 127.9 (2 x ArC), 127.2 (ArC), 127.1 (ArC), 126.8 (ArC), 126.7 (ArC), 124.0 (ArC), 123.9 (ArC), 121.3 (ArC), 62.7 (NCH, both diastereoisomers), 56.9 (C), 51.7 (C), 49.0 (C), 46.9 (C), 45.8 (C), 45.6 (C), 45.0 (C), 42.9 (C), 42.1 ($ArCH_2$), 38.7 (C), 36.7 (2 x C), 36.6 (C), 36.1 (C), 35.5 (C), 35.4 (C), 35.3 (C), 33.9 (2 x C), 33.0 ($C(\mathbf{C}H_3)_3$, both diastereoisomers), 30.7 (C), 30.6 (C), 27.5 (C), 25.2 (C), 22.0 (CH_3, both diastereoisomers), 19.0 (CH_3), 18.8 (CH_3), 11.9 (CH_3, both diastereoisomers); ν_{max}/cm^{-1} (thin film) 2961, 1707, 1647, 1591, 1487, 1435, 1385, 1273, 1051, 910, 727, 700, 646; HRMS (ESI); calcd. for $C_{47}H_{59}N_2O_4^+$, 715.4469. Found: $[MH]^+$, 715.4486 (–2.4 error ppm).

_N_-(2-(_tert_-Butyl)phenyl)-_N_-(1-phenyl-2-(pyridin-2-yl)ethyl)thiophene-3-carboxamide (6p)

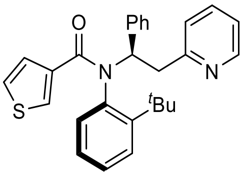

To a mixture of 2-(*tert*-butyl)-*N*-(1-phenyl-2-(pyridin-2-yl)ethyl)aniline **4a** (200 mg, 0.605 mmol) and sodium bicarbonate (203 mg, 2.42 mmol) in dry acetonitrile (3 mL) was added thiophene-3-carbonyl chloride (177 mg, 1.21 mmol) and the resulting mixture was heated to 80 °C for 2 h. The reaction was quenched with sat. aq. $NaHCO_3$ (15 mL) and extracted with DCM (3 x 15 mL). The combined organic layers were dried over $MgSO_4$, filtered and concentrated *in vacuo*. At this point, a ^1H NMR spectrum of the crude product was taken to determine the *dr* (>95:5) of the reaction before chromatography. The crude product was then purified by column chromatography (SiO_2, 8:2 hexane:ethyl acetate → 7:3 hexane:ethyl acetate) to yield the title compound (175 mg, 66%) as a white solid. R_f 0.17 (8:2 hexane:ethyl acetate); m.p. 98–100 °C; δ_H (400 MHz, $CDCl_3$) 8.50–8.46 (1H, m, ArH), 7.57–7.48 (3H, m, ArH), 7.42–7.32 (3H, m, ArH), 7.31–7.13 (4H, m, ArH), 7.03–6.95 (3H, m, ArH), 6.89 (1H, d, *J* = 8.0 Hz, ArH), 6.69–6.66 (1H, m, ArH), 5.87 (1H, dd, *J* = 11.5, 4.5 Hz, NCH), 3.45 (1H, dd, *J* = 13.5, 4.5 Hz, C**H**H'), 3.11 (1H, dd, *J* = 13.5, 11.5 Hz, CH**H'**), 1.15 (9H, s, CH_3); δ_C (101 MHz, $CDCl_3$) 164.2 (CO), 158.3 (ArC), 149.4 (ArC), 148.5 (ArC), 140.6 (ArC), 138.4 (ArC), 138.2 (ArC), 136.1 (ArC), 132.3 (ArC), 131.8 (ArC), 130.2 (ArC), 130.0 (ArC), 129.2 (ArC), 128.8 (ArC), 128.1 (ArC), 127.4 (ArC), 126.6 (ArC), 124.0 (ArC), 123.8 (ArC), 121.5 (ArC), 64.0 (NCH), 42.0 (CH_2), 36.6 ($\mathbf{C}(CH_3)_3$), 32.6 ($C(\mathbf{C}H_3)_3$); ν_{max}/cm^{-1} (thin film) 2962, 1623, 1592, 1487, 1436, 1417, 1335, 1296, 1077, 906, 726, 698, 645, 554; HRMS (ESI); calcd. for $C_{28}H_{29}N_2OS^+$, 441.1995. Found: $[MH]^+$, 441.2003 (–1.7 error ppm).

2-(Benzyloxy)-_N_-(2-(_tert_-butyl)phenyl)-_N_-(1-phenyl-2-(pyridin-2-yl)ethyl)acetamide (6q)

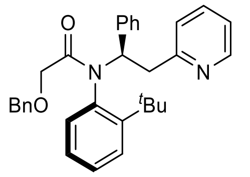

To a mixture of 2-(*tert*-butyl)-*N*-(1-phenyl-2-(pyridin-2-yl)ethyl)aniline **4a** (200 mg, 0.605 mmol) and sodium bicarbonate (203 mg, 2.42 mmol) in dry acetonitrile (3 mL) was added

benzyloxyacetyl chloride (223 mg, 1.21 mmol) and the resulting mixture was heated to 80 °C for 1 h. After this time the reaction was deemed incomplete by TLC and so benzyloxyacetyl chloride (112 mg, 0.605 mmol) was added and the mixture was stirred for a further 1 h. The reaction was quenched with sat. aq. $NaHCO_3$ (15 mL) and extracted with DCM (3 x 15 mL). The combined organic layers were dried over $MgSO_4$, filtered and concentrated *in vacuo*. At this point, a ^{1}H NMR spectrum of the crude product was taken to determine the *dr* (>90:10) of the reaction before chromatography. The crude product was then purified by column chromatography (SiO_2, 6:4 hexane:ethyl acetate) to yield the title compound (202 mg, 70%) as a colourless oil. R_f 0.33 (6:4 hexane:ethyl acetate); δ_H (400 MHz, $CDCl_3$) 8.47–8.40 (1H, m, ArH), 7.55 (1H, dd, *J* = 8.0, 1.5 Hz, ArH), 7.49–7.44 (2H, m, ArH), 7.38–7.30 (2H, m, ArH), 7.29–7.12 (9H, m, ArH), 6.97 (1H, dd, *J* = 7.5, 5.0 Hz, ArH), 6.89 (1H, dd, *J* = 8.0, 1.5 HZ, ArH), 6.83 (1H, d, *J* = 8.0 Hz, ArH), 5.72 (1H, dd, *J* = 12.0, 4.5 Hz, NCH), 4.60 (1H, d, *J* = 12.0 Hz, PhC**H**H'), 4.52 (1H, d, *J* = 12.0 Hz, PhCH**H'**), 3.71 (1H, d, *J* = 15.5 Hz, COC**H**H'), 3.61 (1H, d, *J* = 15.5 Hz, COCH**H'**), 3.43 (1H, dd, *J* = 13.5, 4.5 Hz, ArC**H**H'), 2.99 (1H, dd, *J* = 13.5, 12.0 Hz, ArCH**H'**), 1.33 (9H, s, CH_3); δ_C (101 MHz, $CDCl_3$) 169.7 (CO), 158.0 (ArC), 149.3 (ArC), 147.8 (ArC), 140.1 (ArC), 137.6 (ArC), 136.1 (ArC), 136.0 (ArC), 131.9 (ArC), 131.3 (ArC), 129.3 (ArC), 129.0 (ArC), 128.4 (ArC), 128.0 (2 x ArC), 127.7 (ArC), 127.4 (ArC), 126.9 (ArC), 124.0 (ArC), 121.4 (ArC), 72.9 ($PhCH_2$), 69.3 (CO**C**H_2), 63.4 (NCH), 41.5 ($ArCH_2$), 36.6 (**C**$(CH_3)_3$), 32.8 (C(**C**$H_3)_3$); ν_{max}/cm^{-1} (thin film) 2958, 1735, 1719, 1670, 1590, 1474, 1395, 1277, 1198, 1115, 1028, 909, 728, 697; HRMS (ESI); calcd. for $C_{32}H_{35}N_2O_2^+$, 479.2693. Found: $[MH]^+$, 479.2698 (–0.9 error ppm).

***N*-(2-(*tert*-Butyl)phenyl)-*N*-(1-(pyridin-2-yl)propan-2-yl)propionamide (6r)**

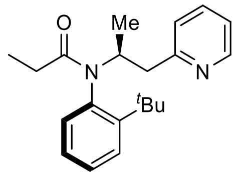

To a mixture of 2-(*tert*-butyl)-*N*-(1-(pyridin-2-yl)propan-2-yl)aniline **4b** (127 mg, 0.473 mmol) and sodium bicarbonate (159 mg, 1.89 mmol) in dry acetonitrile (2.4 mL) was added propionyl chloride (0.080 mL, 88 mg, 0.946 mmol) and the resulting mixture was heated to 80 °C for 1 h. The reaction was quenched with sat. aq. $NaHCO_3$ (15 mL) and extracted with DCM (3 x 15 mL). The combined organic layers were dried over $MgSO_4$, filtered and concentrated *in vacuo*. At this point, a ^{1}H NMR spectrum of the crude product was taken to determine the *dr* (>95:5) of the reaction before chromatography. The crude product was then purified by column chromatography (SiO_2, 7:3 hexane:ethyl acetate → 6:4 hexane:ethyl acetate) to yield the title compound (131 mg, 85%) as a yellow oil. R_f 0.24 (7:3 hexane:ethyl acetate); δ_H (400 MHz, $CDCl_3$) 8.47–8.42 (1H, m, ArH), 7.58–7.50 (2H, m, ArH), 7.33–7.27 (1H, m, ArH), 7.20–7.11 (2H, m, ArH), 7.09–7.03 (1H, m, ArH), 6.99–6.95 (1H, m, ArH), 4.52 (1H, dqd, *J* = 12.5, 6.5, 3.5 Hz, NCH), 3.09 (1H, dd, *J* = 12.5, 3.5 Hz, ArC**H**H'), 2.52 (1H, dd, *J* = 12.5, 12.5 Hz, ArCH**H'**), 2.03–2.84 (2H, m, $COCH_2$), 1.40 (9H, s, $C(CH_3)_3$), 1.30 (3H, d, *J* = 6.5 Hz, CHC**H**$_3$), 1.01 (1H, t, *J* = 7.5 Hz, CH_2C**H**$_3$); δ_C (101 MHz, $CDCl_3$) 174.4 (CO), 159.2 (ArC), 149.3 (ArC), 146.9 (ArC), 138.6 (ArC), 136.4 (ArC), 131.3 (ArC), 130.7 (ArC), 128.5 (ArC), 127.0 (ArC), 123.8 (ArC), 121.5 (ArC), 55.4 (NCH), 42.6 ($ArCH_2$), 36.6 (**C**$(CH_3)_3$), 32.6 (C(**C**$H_3)_3$), 29.9 (CO**C**H_2), 18.7 (CH**C**H_3), 9.3 (CH_2**C**H_3); ν_{max}/cm^{-1} (thin film) 2970, 1650, 1590, 1569, 1487, 1436, 1375, 1267, 1240, 1050, 922, 760,

729, 574, 557; HRMS (ESI); calcd. for $C_{21}H_{29}N_2O^+$, 325.2274. Found: $[MH]^+$, 325.2277 (–0.8 error ppm).

***N*-(2-(*tert*-Butyl)phenyl)-*N*-(1-phenyl-2-(pyridin-2-yl)ethyl)cyclohexanecarboxamide (6s)**

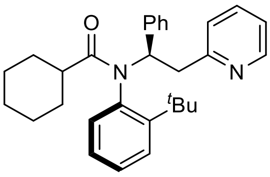

To a mixture of 2-(*tert*-butyl)-*N*-(1-phenyl-2-(pyridin-2-yl)ethyl)aniline **4a** (200 mg, 0.605 mmol) and sodium bicarbonate (203 mg, 2.42 mmol) in dry acetonitrile (3 mL) was added cyclohexanecarbonyl chloride (0.160 mL, 177 mg, 1.21 mmol) and the resulting mixture was heated to 80 °C for 1 h. The reaction was quenched with sat. aq. $NaHCO_3$ (15 mL) and extracted with DCM (3 x 15 mL). The combined organic layers were dried over $MgSO_4$, filtered and concentrated *in vacuo*. At this point, a ^{1}H NMR spectrum of the crude product was taken to determine the *dr* (>95:5) of the reaction before chromatography. The crude product was then purified by column chromatography (SiO_2, 8:2 hexane:ethyl acetate → 6:4 hexane:ethyl acetate) to yield the title compound (55 mg, 21%) as a white solid. R_f 0.16 (8:2 hexane:ethyl acetate); m.p. 119–124 °C; δ_H (400 MHz, $CDCl_3$) 8.44–8.39 (1H, m, ArH), 7.62 (1H, dd, *J* = 8.0, 1.5 Hz, ArH), 7.41–7.31 (4H, m, ArH), 7.23–7.09 (5H, m, ArH), 6.96 (1H, dd, *J* = 7.5, 5.0 Hz, ArH), 6.79 (1H, d, *J* = 8.0 Hz, ArH), 7.72 (1H, dd, *J* = 12.0, 4.5 Hz, NCH), 3.29 (1H, dd, *J* = 13.5, 4.5 Hz, ArC**H**H'), 2.90 (1H, dd, *J* = 13.5, 12.0 Hz, ArCH**H'**), 1.95 (1H, tt, *J* = 11.5, 3.0 Hz, COCH), 1.77–1.62 (3H, m, CHH'), 1.55–1.46 (3H, m, CHH'), 1.41 (9H, s, CH_3), 1.15–1.02 (2H, m, CHH'), 0.99–0.91 (1H, m, CHH'), 0.80–0.66 (1H, m, CHH'); δ_C (101 MHz, $CDCl_3$) 176.5 (CO), 158.3 (ArC), 149.3 (ArC), 147.8 (ArC), 141.1 (ArC), 137.7 (ArC), 135.9 (ArC), 131.9 (ArC), 131.1 (ArC), 128.9 (ArC), 128.5 (ArC), 127.9 (ArC), 127.1 (ArC), 126.5 (ArC), 123.9 (ArC), 121.3 (ArC), 62.6 (NCH), 43.4 (CO**C**H), 42.4 ($ArCH_2$), 36.8 (**C**(CH_3)$_3$), 33.1 (C(**C**H_3)$_3$), 29.7 (CH_2), 27.9 (CH_2), 26.0 (CH_2), 25.8 (CH_2), 25.3 (CH_2); ν_{max}/cm^{-1} (thin film) 2928, 1645, 1590, 1486, 1436, 1299, 1266, 1214, 1051, 909, 760, 728, 698; HRMS (ESI); calcd. for $C_{30}H_{37}N_2O^+$, 441.2900. Found: $[MH]^+$, 441.2904 (–0.8 error ppm).

***N*-(2-(*tert*-Butyl)phenyl)-*N*-(1-phenyl-2-(pyridin-2-yl)ethyl)pivalamide (6t)**

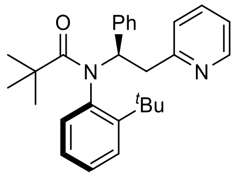

To a mixture of 2-(*tert*-butyl)-*N*-(1-phenyl-2-(pyridin-2-yl)ethyl)aniline **4a** (200 mg, 0.605 mmol) and sodium bicarbonate (203 mg, 2.42 mmol) in dry acetonitrile (3 mL) was added pivaloyl chloride (0.150 mL, 146 mg, 1.21 mmol) and the resulting mixture was heated to 80 °C for 1 h. The reaction was quenched with sat. aq. $NaHCO_3$ (15 mL) and extracted with DCM (3 x 15 mL). The combined organic layers were dried over $MgSO_4$, filtered and concentrated *in vacuo*. No evidence of product formation was observed by TLC or ^{1}H NMR.

N-(2-(*tert*-Butyl)phenyl)-2-phenyl-N-(1-phenyl-2-(pyridin-2-yl)ethyl)acetamide (6u)

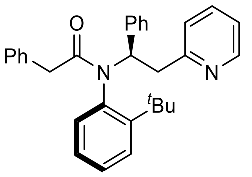

To a mixture of 2-(*tert*-butyl)-*N*-(1-phenyl-2-(pyridin-2-yl)ethyl)aniline **4a** (200 mg, 0.605 mmol) and sodium bicarbonate (203 mg, 2.42 mmol) in dry acetonitrile (3 mL) was added phenylacetyl chloride (0.160 mL, 187 mg, 1.21 mmol) and the resulting mixture was heated to 80 °C for 24 h. The reaction was quenched with sat. aq. $NaHCO_3$ (15 mL) and extracted with DCM (3 x 15 mL). The combined organic layers were dried over $MgSO_4$, filtered and concentrated *in vacuo*. At this point, a ^{1}H NMR spectrum of the crude product was taken to determine the *dr* (>98:2) of the reaction before chromatography. The crude product was then purified by column chromatography (SiO_2, 7:3 hexane:ethyl acetate) to yield the title compound (29 mg, 11%) as a colourless oil. R_f 0.33 (7:3 hexane:ethyl acetate); δ_H (400 MHz, $CDCl_3$) 8.45–8.40 (1H, m, ArH), 7.64 (1H, dd, *J* = 8.0, 1.5 Hz, ArH), 7.40–7.24 (5H, m, ArH), 7.20–7.28 (6H, m, ArH), 7.00–6.95 (1H, m, ArH), 6.94–6.89 (2H, m, ArH), 7.79–6.72 (2H, m, ArH), 5.65 (1H, dd, *J* = 11.5, 5.0 Hz, NCH), 3.46 (1H, dd, *J* = 13.0, 5.0 Hz, ArC**H**H'), 3.38 (1H, d, *J* = 15.0 Hz, COC**H**H'), 3.26 (1H, d, *J* = 15.0 Hz, COCH**H'**), 2.99 (1H, dd, *J* = 13.0, 11.5 Hz, ArCH**H'**), 1.45 (9H, s, CH_3); δ_C (101 MHz, $CDCl_3$) 171.4 (CO), 158.2 (ArC), 149.3 (ArC), 147.7 (ArC), 140.5 (ArC), 138.3 (ArC), 136.1 (ArC), 135.0 (ArC), 132.4 (ArC), 131.2 (ArC), 129.5 (ArC), 129.2 (ArC), 128.7 (ArC), 128.2 (ArC), 128.0 (ArC), 127.3 (ArC), 126.8 (ArC), 126.6 (ArC), 124.1 (ArC), 121.4 (ArC), 64.2 (NCH), 43.6 (CO**C**H_2), 42.2 ($ArCH_2$), 36.8 (**C**(CH_3)$_3$), 33.1 (C(**C**H_3)$_3$); ν_{max}/cm^{-1} (thin film) 2959, 1702, 1652, 1591, 1486, 1436, 1371, 1267, 1147, 910, 759, 730, 699, 548; HRMS (ESI); calcd. for $C_{31}H_{33}N_2O^+$, 449.2587. Found: $[MH]^+$, 449.2587 (0.1 error ppm).

Ethyl 3-((2-(*tert*-butyl)phenyl)(1-phenyl-2-(pyridin-2-yl)ethyl)amino)-3-oxopropanoate (6v)

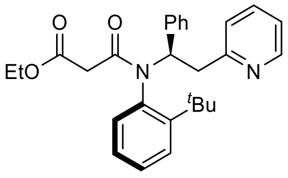

To a mixture of 2-(*tert*-butyl)-*N*-(1-phenyl-2-(pyridin-2-yl)ethyl)aniline **4a** (200 mg, 0.605 mmol) and sodium bicarbonate (203 mg, 2.42 mmol) in dry acetonitrile (3 mL) was added ethyl malonyl chloride (0.150 mL, 182 mg, 1.21 mmol) and the resulting mixture was heated to 80 °C for 1 h. The reaction was quenched with sat. aq. $NaHCO_3$ (15 mL) and extracted with DCM (3 x 15 mL). The combined organic layers were dried over $MgSO_4$, filtered and concentrated *in vacuo*. No evidence of product formation was observed by TLC or ^{1}H NMR.

2-(*tert*-Butyl)-*N*-(phenyl(pyridin-2-yl)methyl)aniline (4c)

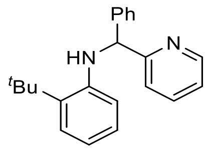

2-Bromopyridine (7.90 g, 50.0 mmol) was dissolved in dry THF (120 mL) and cooled to –78 °C before adding *n*-BuLi (20 mL, 2.5 M in hexane, 50 mmol). The mixture was stirred for 15 minutes before adding *N*-(2-(*tert*-butyl)phenyl)-1-phenylmethanimine **S1** (5.93 g, 25 mmol), dissolved in dry THF (20 mL), dropwise. The resulting mixture was warmed to room temperature and stirred for 1 h. The reaction was quenched with sat. aq. NH_4Cl (150 mL) and extracted with ethyl acetate (3 x 150 mL). The combined organic layers were dried over $MgSO_4$, filtered and concentrated *in vacuo*. The crude product was then purified by column chromatography (SiO_2, 9:1 hexane:ethyl acetate) to yield the title compound (4.15 g, 52%) as a white solid. R_f 0.39 (9:1 hexane:ethyl acetate); m.p. 74–75 °C; δ_H (400 MHz, $CDCl_3$) 8.68–8.63 (1H, m, ArH), 7.64 (1H, td, *J* = 8.0, 2.0 Hz, ArH), 7.58–7.52 (2H, m, ArH), 7.43–7.24 (5H, m, ArH), 7.21–7.15 (1H, m, ArH), 7.04 (1H, td, *J* = 7.5, 1.5 Hz, ArH), 6.71 (1H, td, *J* = 7.5, 1.5 Hz, ArH), 6.59–6.54 (1H, m, ArH), 6.28 (1H, d, *J* = 4.0 Hz, NH), 5.70 (1H, d, *J* = 4.0 Hz, CH), 1.62 (9H, s, CH_3); δ_C (101 MHz, $CDCl_3$) 170.0 (ArC), 149.1 (ArC), 144.8 (ArC), 143.2 (ArC), 136.9 (ArC), 133.6 (ArC), 129.0 (ArC), 127.5 (ArC), 127.3 (ArC), 127.1 (ArC), 126.3 (ArC), 122.2 (ArC), 122.0 (ArC), 116.8 (ArC), 112.6 (ArC), 63.5 (CH), 34.4 (**C**(CH_3)$_3$), 30.0 (C(**C**H_3)$_3$); ν_{max}/cm^{-1} (thin film) 3438, 2957, 1595, 1573, 1504, 1447, 1428, 1309, 1262, 1055, 908, 741, 699, 591; HRMS (ESI); calcd. for $C_{22}H_{25}N_2^+$, 317.2012. Found: [MH]$^+$, 317.2016 (–1.1 error ppm).

***N*-(2-(*tert*-Butyl)phenyl)-1-(pyridin-2-yl)methanimine (S3)**

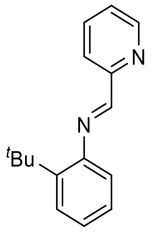

To a solution of 2-*tert*-butylaniline (1.56 mL, 1.49 g, 10.0 mmol) in ethanol (40 mL) was added 2-pyridinecarboxaldehyde (0.950 mL, 1.07 g, 10.0 mmol) and the solution was heated to reflux for 18 h. The reaction mixture was concentrated *in vacuo* to yield the crude product (2.38 g) which was used directly in the next step without further purification.

2-(*tert*-Butyl)-*N*-(1-(pyridin-2-yl)ethyl)aniline (4d)

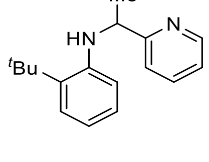

Methyllithium (12.5 mL, 1.6 M in Et_2O, 20.0 mmol) was diluted with THF (30 mL) and cooled to –78 °C. To this was added *N*-(2-(*tert*-butyl)phenyl)-1-(pyridin-2-yl)methanimine **S3** (2.38 g, 10.0 mmol), dissolved in dry THF (10 mL), dropwise. The mixture was warmed to room

temperature and stirred for 1 h. The reaction was quenched with sat. aq. NH_4Cl (50 mL) and extracted with ethyl acetate (3 x 50 mL). The combined organic layers were dried over $MgSO_4$, filtered and concentrated *in vacuo*. The crude product was then purified by column chromatography (SiO_2, 9:1 hexane:ethyl acetate) to yield the title compound (2.31 g, 91%) as a yellow oil. R_f 0.30 (9:1 hexane:ethyl acetate); δ_H (400 MHz, $CDCl_3$) 8.67–8.64 (1H, m, ArH), 7.64 (1H, td, *J* = 7.5, 1.5 Hz, ArH), 7.42–7.38 (1H, m, ArH), 7.33 (1H, dd, *J* = 8.0, 1.5 Hz, ArH), 7.18 (1H, ddd, *J* = 7.5, 4.5, 1.0 Hz, ArH), 7.07 (1H, td, *J* = 7.5, 1.5 Hz, ArH), 6.72 (1H, td, *J* = 7.5, 1.5 Hz, ArH), 6.55 (1H, dd, *J* = 8.0, 1.5 Hz, ArH), 4.97 (1H, d, *J* = 5.5 Hz, NH), 4.82–4.73 (1H, m, CH), 1.67 (3H, d, *J* = 7.0 Hz, CH_3), 1.59 (9H, s, $C(CH_3)_3$); δ_C (101 MHz, $CDCl_3$) 164.1 (ArC), 149.3 (ArC), 144.9 (ArC), 136.9 (ArC), 133.1 (ArC), 127.1 (ArC), 126.3 (ArC), 121.9 (ArC), 120.2 (ArC), 116.8 (ArC), 112.5 (ArC), 55.0 (CH), 34.2 (**C**(CH_3)$_3$), 30.0 (C(**C**H_3)$_3$), 23.5 (CH_3); ν_{max}/cm^{-1} (thin film) 3443, 2965, 1592, 1573, 1505, 1446, 1434, 1368, 1309, 1054, 781, 741; HRMS (ESI); calcd. for $C_{17}H_{23}N_2^+$, 255.1856. Found: $[MH]^+$, 255.1858 (–0.7 error ppm).

***N*-(2-(*tert*-Butyl)phenyl)-*N*-(phenyl(pyridin-2-yl)methyl)propionamide (6w)**

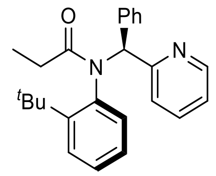

To a mixture of 2-(*tert*-butyl)-*N*-(phenyl(pyridin-2-yl)methyl)aniline **4c** (191 mg, 0.605 mmol) and sodium bicarbonate (203 mg, 2.42 mmol) in dry acetonitrile (3 mL) was added propionyl chloride (0.110 mL, 112 mg, 1.21 mmol) and the resulting mixture was heated to 80 °C for 1 h. The reaction was quenched with sat. aq. $NaHCO_3$ (15 mL) and extracted with DCM (3 x 15 mL). The combined organic layers were dried over $MgSO_4$, filtered and concentrated *in vacuo*. At this point, a ^1H NMR spectrum of the crude product was taken to determine the *dr* (>95:5) of the reaction before chromatography. The crude product was then purified by column chromatography (SiO_2, 8:2 hexane:ethyl acetate) to yield the title compound (176 mg, 78%) as a white solid. R_f 0.30 (8:2 hexane:ethyl acetate); m.p. 133–136 °C; δ_H (400 MHz, $CDCl_3$) 8.51–8.47 (1H, m, ArH), 7.59 (1H, dd, *J* = 8.0, 1.5 Hz, ArH), 7.47–7.21 (8H, m, ArH), 7.19–7.13 (1H, m, ArH), 7.07–7.01 (1H, m, ArH), 6.83–6.77 (1H, m, ArH), 6.60 (1H, s, CH), 2.11 (2H, qd, *J* = 7.5, 2.5 Hz, CH_2), 1.15–1.05 (12H, m, $C(CH_3)_3$, CH_3); δ_C (101 MHz, $CDCl_3$) 174.9 (CO), 159.0 (ArC), 148.6 (ArC), 147.5 (ArC), 140.5 (ArC), 139.7 (ArC), 135.7 (ArC), 132.3 (ArC), 130.7 (ArC), 129.0 (ArC), 128.4 (ArC), 128.3 (ArC), 127.2 (ArC), 126.7 (ArC), 126.0 (ArC), 122.2 (ArC), 70.5 (CH), 36.4 (**C**(CH_3)$_3$), 32.4 (C(**C**H_3)$_3$), 30.0 (CH_2), 9.3 (CH_3); ν_{max}/cm^{-1} (thin film) 2969, 1659, 1588, 1570, 1488, 1434, 1378, 1247, 1077, 910, 749, 730, 700, 614; HRMS (ESI); calcd. for $C_{25}H_{29}N_2O^+$, 373.2274. Found: $[MH]^+$, 373.2288 (–3.7 error ppm).

N-(2-(tert-Butyl)phenyl)-N-(phenyl(pyridin-2-yl)methyl)cyclohexanecarboxamide (S4)

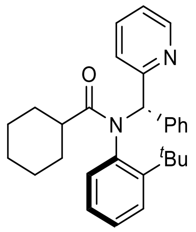

To a mixture of 2-(*tert*-butyl)-*N*-(phenyl(pyridin-2-yl)methyl)aniline **4c** (191 mg, 0.605 mmol) and sodium bicarbonate (203 mg, 2.42 mmol) in dry acetonitrile (3 mL) was added cyclohexanecarbonyl chloride (0.160 mL, 177 mg, 1.21 mmol) and the resulting mixture was heated to 80 °C for 1 h. The reaction was quenched with sat. aq. $NaHCO_3$ (15 mL) and extracted with DCM (3 x 15 mL). The combined organic layers were dried over $MgSO_4$, filtered and concentrated *in vacuo*. At this point, a ^{1}H NMR spectrum of the crude product was taken to determine the *dr* (>95:5) of the reaction before chromatography. The crude product was then purified by column chromatography (SiO_2, 89:10:1 hexane:ethyl acetate:triethylamine → 79:20:1 hexane:ethyl acetate:triethylamine) to yield the title compound (197 mg, 76%) as a yellow oil. R_f 0.19 (89:10:1 hexane:ethyl acetate:triethylamine); δ_H (400 MHz, $CDCl_3$) 8.49–8.45 (1H, m, ArH), 7.59 (1H, dd, *J* = 8.0, 1.5 Hz, ArH), 7.45–7.19 (8H, m, ArH), 7.15–7.09 (1H, m, ArH), 7.04–6.99 (1H, m, ArH), 6.77 (1H, d, *J* = 8.0 Hz, ArH), 6.64 (1H, s, NCH), 2.15 (1H, tt, *J* = 11.5, 3.5 Hz, COCH), 1.98–1.89 (1H, m, CHH'), 1.82–1.49 (5H, m, CHH'), 1.39–0.75 (13H, $C(CH_3)_3$, CHH'); δ_C (101 MHz, $CDCl_3$) 176.8 (CO), 158.9 (ArC), 148.5 (ArC), 147.4 (ArC), 140.6 (ArC), 138.8 (ArC), 135.6 (ArC), 132.2 (ArC), 130.6 (ArC), 128.7 (ArC), 128.3 (ArC), 128.1 (ArC), 127.1 (ArC), 126.3 (ArC), 126.0 (ArC), 122.1 (ArC), 69.9 (NCH), 43.1 (CO**C**H), 36.5 (**C**$(CH_3)_3$), 32.6 (C(**C**$H_3)_3$), 29.9 (CH_2), 28.1 (CH_2), 26.0 (CH_2), 25.8 (CH_2), 25.3 (CH_2); ν_{max}/cm^{-1} (thin film) 2930, 1646, 1588, 1570, 1488, 1434, 1337, 1214, 1051, 908, 727, 699, 610; HRMS (ESI); calcd. for $C_{29}H_{35}N_2O^+$, 427.2744. Found: $[MH]^+$, 427.2747 (–0.7 error ppm).

Benzyl 4-((2-(tert-butyl)phenyl)(phenyl(pyridin-2-yl)methyl)carbamoyl)piperidine-1-carboxylate (6x)

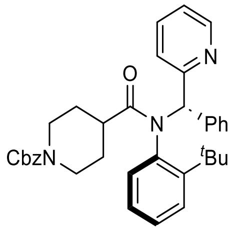

To a mixture of 2-(*tert*-butyl)-*N*-(phenyl(pyridin-2-yl)methyl)aniline **4c** (191 mg, 0.605 mmol) and sodium bicarbonate (203 mg, 2.42 mmol) in dry acetonitrile (3 mL) was added benzyl 4-(chlorocarbonyl)piperidine-1-carboxylate (341 mg, 1.21 mmol) and the resulting mixture was heated to 80 °C for 1 h. The reaction was quenched with sat. aq. $NaHCO_3$ (15 mL) and extracted with DCM (3 x 15 mL). The combined organic layers were dried over $MgSO_4$, filtered and concentrated *in vacuo*. At this point, a ^{1}H NMR spectrum of the crude product was taken to determine the *dr* (>95:5) of the reaction before chromatography. The crude product was then purified by column chromatography (SiO_2, 79:20:1 hexane:ethyl acetate:triethylamine →

69:30:1 hexane:ethyl acetate:triethylamine) to yield the title compound (213 mg, 70%) as a colourless oil. R_f 0.42 (69:30:1 hexane:ethyl acetate:triethylamine); δ_H (400 MHz, $CDCl_3$) 8.52–8.48 (1H, m, ArH), 7.77 (1H, d, *J* = 8.0 Hz, ArH), 7.50–7.22 (13H, m, ArH), 7.18 (1H, t, *J* = 7.5 Hz, ArH), 7.08–7.02 (1H, m, ArH), 6.80 (1H, d, *J* = 8.0 Hz, ArH), 6.45 (1H, s, NCH), 5.10 (2H, s, $PhCH_2$), 4.27–3.93 (2H, m, NCH_2), 2.72–2.26 (3H, m, NCH_2, COCH), 2.03–1.81 (2H, m, CH_2), 1.75–1.48 (2H, m, CH_2), 1.12 (9H, s, $C(CH_3)_3$); δ_C (101 MHz, $CDCl_3$) 175.1 (CO), 158.8 (CO), 155.2 (ArC), 148.5 (ArC), 147.2 (ArC), 140.0 (ArC), 139.4 (ArC), 136.9 (ArC), 135.8 (ArC), 132.0 (ArC), 130.6 (ArC), 129.2 (ArC), 128.5 (2 x ArC), 128.4 (ArC), 127.9 (ArC), 127.8 (ArC), 127.5 (ArC), 126.7 (ArC), 125.4 (ArC), 122.1 (ArC), 71.2 (NCH), 67.0 ($PhCH_2$), 43.5 (NCH_2), 43.0 (NCH_2), 41.0 (CO**C**H), 36.5 (**C**$(CH_3)_3$), 32.6 (C(**C**$H_3)_3$), 28.6 (CH_2), 27.2 (CH_2); ν_{max}/cm^{-1} (thin film) 2956, 1694, 1652, 1588, 1488, 1433, 1339, 1210, 1122, 909, 761, 727, 697, 611; HRMS (ESI); calcd. for $C_{36}H_{40}N_3O_3^+$, 562.3064. Found: $[MH]^+$, 562.3079 (–2.6 error ppm).

***N*-(2-(*tert*-Butyl)phenyl)-2-(1-(4-chlorobenzoyl)-5-methoxy-2-methyl-1*H*-indol-3-yl)-*N*-(phenyl(pyridin-2-yl)methyl)acetamide (6y)**

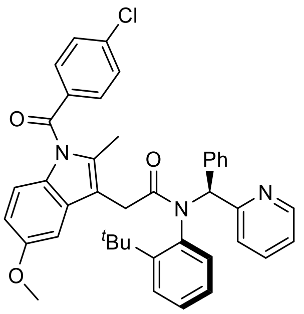

To a mixture of 2-(*tert*-butyl)-*N*-(phenyl(pyridin-2-yl)methyl)aniline **4c** (191 mg, 0.605 mmol) and sodium bicarbonate (203 mg, 2.42 mmol) in dry acetonitrile (3 mL) was added 2-(1-(4-chlorobenzoyl)-5-methoxy-2-methyl-1*H*-indol-3-yl)acetyl chloride (455 mg, 1.21 mmol) and the resulting mixture was heated to 80 °C for 1 h. The reaction was quenched with sat. aq. $NaHCO_3$ (15 mL) and extracted with DCM (3 x 15 mL). The combined organic layers were dried over $MgSO_4$, filtered and concentrated *in vacuo*. At this point, a ^1H NMR spectrum of the crude product was taken to determine the *dr* (>95:5) of the reaction before chromatography. The crude product was then purified by column chromatography (SiO_2, 79:20:1: hexane:ethyl acetate:triethylamine) to yield the title compound (321 mg, 88%) as a yellow solid. R_f 0.52 (7:3 hexane:ethyl acetate); m.p. 74–79 °C; δ_H (400 MHz, $CDCl_3$) 8.46–8.43 (1H, m, ArH), 7.67–7.58 (3H, m, ArH), 7.49–7.43 (3H, m, ArH), 7.39–7.18 (7H, m, ArH), 7.15 (1H, td, *J* = 7.5, 1.5 Hz, ArH), 7.05–6.99 (2H, m, ArH), 6.87 (1H, d, *J* = 9.0 Hz, ArH), 6.72 (1H, d, *J* = 8.0 Hz, ArH), 6.66 (1H, dd, *J* = 9.0, 2.5 Hz, ArH), 6.56 (1H, s, NCH), 3.73 (3H, s, OCH_3), 3.64 (1H, d, *J* = 15.5 Hz, C**H**H’), 3.46 (1H, d, *J* = 15.5 Hz, CH**H’**), 1.92 (3H, s, $ArCH_3$), 1.27 (9H, s, $C(CH_3)_3$); δ_C (101 MHz, $CDCl_3$) 170.7 (CO), 168.4 (CO), 158.7 (ArC), 155.9 (ArC), 148.6 (ArC), 147.7 (ArC), 140.0 (ArC), 139.3 (ArC), 139.1 (ArC), 135.9 (ArC), 135.8 (ArC), 134.3 (ArC), 132.7 (ArC), 131.3 (ArC), 131.2 (ArC), 131.1 (ArC), 130.9 (ArC), 129.1 (2 x ArC), 128.6 (ArC), 128.4 (ArC), 127.4 (ArC), 126.9 (ArC), 125.8 (ArC), 122.2 (ArC), 114.8 (ArC), 113.6 (ArC), 111.8 (ArC), 102.2 (ArC), 71.6 (NCH), 55.6 (OCH_3), 36.6 (**C**$(CH_3)_3$), 33.7 (CH_2), 32.7 (C(**C**$H_3)_3$), 13.1 ($ArCH_3$); ν_{max}/cm^{-1} (thin film) 2959, 1666, 1589, 1475, 1434, 1361, 1316, 1223, 1147, 1088, 909, 754, 727, 701; HRMS (ESI); calcd. for $C_{41}H_{39}{}^{35}ClN_3O_3^+$, 656.2674. Found: $[MH]^+$, 656.2695 (–3.2 error ppm).

Methyl 5-((2-(*tert*-Butyl)phenyl)(phenyl(pyridin-2-yl)methyl)amino)-5-oxopentanoate (6z)

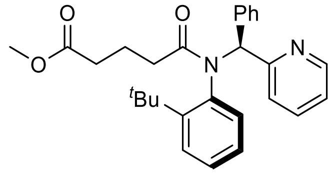

To a mixture of 2-(*tert*-butyl)-*N*-(phenyl(pyridin-2-yl)methyl)aniline **4c** (191 mg, 0.605 mmol) and sodium bicarbonate (203 mg, 2.42 mmol) in dry acetonitrile (3 mL) was added methyl 4-(chloroformyl)butyrate (0.170 mL, 199 mg, 1.21 mmol) and the resulting mixture was heated to 80 °C for 1 h. The reaction was quenched with sat. aq. $NaHCO_3$ (15 mL) and extracted with DCM (3 x 15 mL). The combined organic layers were dried over $MgSO_4$, filtered and concentrated *in vacuo*. At this point, a ^{1}H NMR spectrum of the crude product was taken to determine the *dr* (>95:5) of the reaction before chromatography. The crude product was then purified by column chromatography (SiO_2, 79:20:1 hexane:ethyl acetate:triethylamine → 69:30:1 hexane:ethyl acetate:triethylamine) to yield the title compound (208 mg, 77%) as a colourless oil. R_f 0.39 (69:30:1 hexane:ethyl acetate:triethylamine); δ_H (400 MHz, $CDCl_3$) 8.51–8.46 (1H, m, ArH), 7.65 (1H, dd, *J* = 8.0, 1.5 Hz, ArH), 7.48–7.43 (2H, m, ArH), 7.40–7.13 (7H, m, ArH), 7.06–7.00 (1H, m, ArH), 7.68 (1H, d, *J* = 8.0 Hz, ArH), 6.49 (1H, s, NCH), 3.58 (3H, s, OCH_3), 2.32 (2H, t, *J* = 7.5 Hz, CH_2), 2.20–2.05 (2H, m, CH_2), 2.00–1.83 (2H, m, CH_2), 1.10 (9H, s, $C(CH_3)_3$); δ_C (101 MHz, $CDCl_3$) 173.7 (CO), 173.1 (CO), 158.8 (ArC), 148.5 (ArC), 147.4 (ArC), 140.2 (ArC), 139.5 (ArC), 135.7 (ArC), 132.2 (ArC), 130.6 (ArC), 129.1 (ArC), 128.4 (ArC), 128.3 (ArC), 127.3 (ArC), 126.8 (ArC), 125.6 (ArC), 122.1 (ArC), 77.1 (NCH), 51.4 (OCH_3), 36.3 (**C**$(CH_3)_3$), 35.4 (CH_2), 33.2 (CH_2), 32.3 (C(**C**$H_3)_3$), 20.3 (CH_2); ν_{max}/cm^{-1} (thin film) 2957, 1734, 1655, 1588, 1570, 1488, 1434, 1386, 1214, 1152, 914, 749, 730, 701, 614; HRMS (ESI); calcd. for $C_{28}H_{32}N_2O_3Na^+$, 467.2305. Found: $[MNa]^+$, 467.2316 (–2.3 error ppm).

***N*-(2-(*tert*-Butyl)phenyl)-*N*-(phenyl(pyridin-2-yl)methyl)benzamide (6za)**

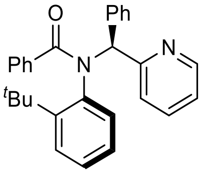

To a mixture of 2-(*tert*-butyl)-*N*-(phenyl(pyridin-2-yl)methyl)aniline **4c** (191 mg, 0.605 mmol) and sodium bicarbonate (203 mg, 2.42 mmol) in dry acetonitrile (3 mL) was added benzoyl chloride (0.140 mL, 170 mg, 1.21 mmol) and the resulting mixture was heated to 80 °C for 1 h. The reaction was quenched with sat. aq. $NaHCO_3$ (15 mL) and extracted with DCM (3 x 15 mL). The combined organic layers were dried over $MgSO_4$, filtered and concentrated *in vacuo*. At this point, a ^{1}H NMR spectrum of the crude product was taken to determine the *dr* (>95:5) of the reaction before chromatography. The crude product was then purified by column chromatography (SiO_2, 8:2 hexane:ethyl acetate) to yield the title compound (178 mg, 70%) as a white solid. R_f 0.35 (8:2 hexane:ethyl acetate); m.p. 114–117 °C; δ_H (400 MHz, $CDCl_3$) 8.51–8.46 (1H, m, ArH), 7.76–7.71 (1H, m, ArH), 7.60–7.56 (2H, m, ArH), 7.43–7.36 (5H, m, ArH), 7.35–7.29 (1H, m, ArH), 7.23–7.17 (1H, m, ArH), 7.16–7.07 (5H, m, ArH), 7.05–7.00 (2H, m, ArH, NCH), 6.96–6.91 (1H, m, ArH), 0.91 (9H, s, CH_3); δ_C (101 MHz, $CDCl_3$) 169.7 (CO), 158.7

(ArC), 148.8 (ArC), 147.6 (ArC), 140.2 (ArC), 138.7 (ArC), 136.8 (ArC), 135.6 (ArC), 132.7 (ArC), 131.2 (ArC), 130.1 (ArC), 129.7 (ArC), 128.8 (ArC), 128.6 (ArC), 127.8 (ArC), 127.4 (2 x ArC), 126.4 (ArC), 125.6 (ArC), 122.2 (ArC), 71.0 (NCH), 36.3 (**C**(CH_3)$_3$), 32.1 (C(**C**H_3)$_3$); $ν_{max}$/cm^{-1} (thin film) 2960, 1636, 1588, 1572, 1489, 1434, 1342, 1094 1029, 910, 760, 729, 697, 610; HRMS (ESI); calcd. for $C_{29}H_{29}N_2O^+$, 421.2274. Found: $[MH]^+$, 421.2287 (–3.1 error ppm).

***N*-(2-(*tert*-Butyl)phenyl)-*N*-(1-(pyridin-2-yl)ethyl)benzamide (6zb)**

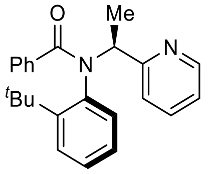

To a mixture of 2-(*tert*-butyl)-*N*-(1-(pyridin-2-yl)ethyl)aniline **4d** (155 mg, 0.605 mmol) and sodium bicarbonate (203 mg, 2.42 mmol) in dry acetonitrile (3 mL) was added benzoyl chloride (0.140 mL, 170 mg, 1.21 mmol) and the resulting mixture was heated to 80 °C for 1 h. The reaction was quenched with sat. aq. $NaHCO_3$ (15 mL) and extracted with DCM (3 x 15 mL). The combined organic layers were dried over $MgSO_4$, filtered and concentrated *in vacuo*. At this point, a ^{1}H NMR spectrum of the crude product was taken to determine the *dr* (91:9) of the reaction before chromatography. The crude product was then purified by column chromatography (SiO_2, 8:2 hexane:ethyl acetate → 7:3 hexane:ethyl acetate) to yield the title compound (157 mg, 72%) as a white solid. R_f 0.16 (8:2 hexane:ethyl acetate); m.p. 129–131 °C; $δ_H$ (400 MHz, $CDCl_3$) 8.43 (1H, d, *J* = 5.0 Hz, ArH), 7.53–7.45 (2H, m, ArH), 7.31–7.23 (3H, m, ArH), 7.19–7.03 (7H, m, ArH), 5.80 (1H, q, *J* = 7.0 Hz, NCH), 2.01 (3H, d, *J* = 7.0 Hz, CH_3), 0.93 (9H, s, C(CH_3)$_3$); $δ_C$ (101 MHz, $CDCl_3$) 169.4 (CO), 159.4 (ArC), 148.5 (ArC), 146.8 (ArC), 138.1 (ArC), 137.2 (ArC), 136.0 (ArC), 133.1 (ArC), 131.0 (ArC), 129.5 (ArC), 129.2 (ArC), 128.1 (ArC), 127.2 (ArC), 125.6 (ArC), 124.4 (ArC), 122.4 (ArC), 62.8 (NCH), 36.2 (**C**(CH_3)$_3$), 32.1 (C(**C**H_3)$_3$), 20.1 (CH_3); $ν_{max}$/cm^{-1} (thin film) 2959, 1631, 1567, 1489, 1435, 1369, 1340, 1233, 1051, 760, 717, 697; HRMS (ESI); calcd. for $C_{24}H_{27}N_2O^+$, 359.2118. Found: $[MH]^+$, 359.2111 (1.8 error ppm).

***N*-(2-(*tert*-Butyl)phenyl)-*N*-(1-(pyridin-2-yl)ethyl)benzamide (11zb)**

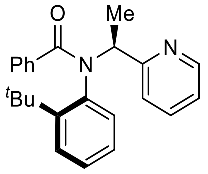

A solution of *N*-(2-(*tert*-butyl)phenyl)-*N*-(1-(pyridin-2-yl)ethyl)benzamide **6zb** (53 mg, 0.148 mmol) in d_6-DMSO (1.0 mL) was heated to 150 °C for 2 h. The reaction mixture was dissolved in DCM (10 mL) and washed with water (3 x 10 mL). The organic layer was dried over $MgSO_4$, filtered and concentrated *in vacuo*. The crude product was then purified by column chromatography (SiO_2, 8:2 hexane:ethyl acetate) to yield the title compound (38 mg, 72%) as a white solid. R_f 0.25 (8:2 hexane:ethyl acetate); m.p. 125–128 °C; $δ_H$ (400 MHz, $CDCl_3$) 8.67–8.62 (1H, m, ArH), 8.40–8.35 (1H, m, ArH), 7.67 (1H, td, *J* = 7.5, 2.0 Hz, ArH), 7.58 (1H, d, *J* = 8.0 Hz, ArH), 7.35–7.22 (5H, m, ArH), 7.18–7.11 (2H, m, ArH), 7.09–7.03 (2H, m, ArH), 5.59

(1H, q, J = 7.5 Hz, NCH), 1.28 (3H, d, J = 7.5 Hz, CH_3), 1.14 (9H, s, $C(CH_3)_3$); δ_C (101 MHz, $CDCl_3$) 169.6 (CO), 162.8 (ArC), 149.3 (ArC), 147.6 (ArC), 136.8 (ArC), 136.5 (ArC), 136.3 (ArC), 134.7 (ArC), 130.7 (ArC), 129.7 (ArC), 129.5 (ArC), 128.2 (ArC), 127.3 (ArC), 125.6 (ArC), 123.2 (ArC), 121.9 (ArC), 59.1 (NCH), 36.3 (**C**(CH_3)$_3$), 32.4 (C(**C**H_3)$_3$), 20.1 (CH**C**H_3); ν_{max}/cm^{-1} (thin film) 2968, 1629, 1594, 1572, 1489, 1435, 1366, 1281, 1243, 1102, 916, 764, 695, 602; HRMS (ESI); calcd. for $C_{24}H_{27}N_2O^+$, 359.2118. Found: $[MH]^+$, 359.2122 (–1.0 error ppm).

***N*-(2-(*tert*-Butyl)phenyl)-*N*-(1-(pyridin-2-yl)ethyl)tetrahydro-2*H*-pyran-4-carboxamide (6zc)**

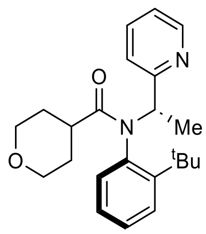

To a mixture of 2-(*tert*-butyl)-*N*-(1-(pyridin-2-yl)ethyl)aniline **4d** (155 mg, 0.605 mmol) and sodium bicarbonate (203 mg, 2.42 mmol) in dry acetonitrile (3 mL) was added tetrahydro-2*H*-pyran-4-carbonyl chloride (0.150 mL, 180 mg, 1.21 mmol) and the resulting mixture was heated to 80 °C for 1 h. The reaction was quenched with sat. aq. $NaHCO_3$ (15 mL) and extracted with DCM (3 x 15 mL). The combined organic layers were dried over $MgSO_4$, filtered and concentrated *in vacuo*. At this point, a ^1H NMR spectrum of the crude product was taken to determine the *dr* (94:6) of the reaction before chromatography. The crude product was then purified by column chromatography (SiO_2, 7:3 hexane:ethyl acetate → 6:4 hexane:ethyl acetate) to yield the title compound (200 mg, 90%) as a white solid. R_f 0.23 (6:4 hexane:ethyl acetate); m.p. 149–151 °C; δ_H (400 MHz, $CDCl_3$) 8.39–8.33 (1H, m, ArH), 7.46 (1H, td, J = 8.0, 2.0 Hz, ArH), 7.39 (1H, dd, J = 8.0, 1.5 Hz, ArH), 7.27–7.22 (1H, m, ArH), 7.15–6.99 (4H, m, ArH), 5.39 (1H, q, J = 7.0 Hz, NCH), 3.95–3.87 (1H, m, OCHH'), 3.82–3.73 (1H, m, OCHH'), 3.18–3.07 (1H, m, OCHH'), 2.98–2.88 (1H, m, OCHH'), 2.22 (1H, tt, J = 11.0, 3.5 Hz, COCH), 2.16–2.03 (1H, m, CHH'), 1.80 (3H, d, J = 7.0 Hz, CH_3), 1.75–1.62 (1H, m, CHH'), 1.58–1.44 (2H, m, 2 x CHH'), 1.12 (9H, s, $C(CH_3)_3$); δ_C (101 MHz, $CDCl_3$) 175.0 (CO), 160.1 (ArC), 148.5 (ArC), 146.8 (ArC), 138.3 (ArC), 136.2 (ArC), 131.8 (ArC), 130.6 (ArC), 128.6 (ArC), 126.3 (ArC), 124.0 (ArC), 122.4 (ArC), 67.2 (OCH_2), 66.9 (OCH_2), 62.2 (NCH), 40.8 (CO**C**H), 36.4 (**C**(CH_3)$_3$), 32.4 (C(**C**H_3)$_3$), 29.8 (CH_2), 28.1 (CH_2), 20.5 (CH**C**H_3); ν_{max}/cm^{-1} (thin film) 2956, 1643, 1591, 1570, 1488, 1435, 1385, 1301, 1240, 1129, 1083, 916, 759, 728, 559; HRMS (ESI); calcd. for $C_{23}H_{31}N_2O_2^+$, 367.2380. Found: $[MH]^+$, 367.2378 (0.5 error ppm).

***N*-(2-(*tert*-Butyl)phenyl)-*N*-(1-(pyridin-2-yl)ethyl)propionamide (6zd)**

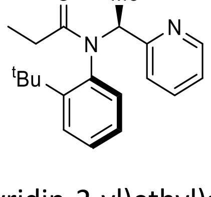

To a mixture of 2-(*tert*-butyl)-*N*-(1-(pyridin-2-yl)ethyl)aniline **4d** (155 mg, 0.605 mmol) and sodium bicarbonate (203 mg, 2.42 mmol) in dry acetonitrile (3 mL) was added propionyl chloride (0.110 mL, 112 mg, 1.21 mmol) and the resulting mixture was heated to 80 °C for 1

h. The reaction was quenched with sat. aq. $NaHCO_3$ (15 mL) and extracted with DCM (3 x 15 mL). The combined organic layers were dried over $MgSO_4$, filtered and concentrated *in vacuo*. At this point, a ^1H NMR spectrum of the crude product was taken to determine the *dr* (85:15) of the reaction before chromatography. The crude product was then purified by column chromatography (SiO_2, 8:2 hexane:ethyl acetate → 7:3 hexane:ethyl acetate) to yield the title compound **6zd** (154 mg, 82%) as a white solid and atropisomer **11zd** (22 mg, 12%) as a colourless oil. Data for **6zd**: R_f 0.10 (8:2 hexane:ethyl acetate); m.p. 76–80 °C; δ_H (400 MHz, $CDCl_3$) 8.42–8.35 (1H, m, ArH), 7.53–7.45 (1H, m, ArH), 7.42–7.46 (1H, m, ArH), 7.29–7.05 (4H, m, ArH), 6.98–6.93 (1H, m, ArH), 5.43 (1H, q, *J* = 7.0 Hz, NCH), 2.05–1.91 (2H, m, CH_2), 1.84 (3H, d, *J* = 7.0 Hz, CHC**H**$_3$), 1.12 (9H, s, C(CH_3)$_3$), 1.04 (3H, t, *J* = 7.5 Hz, CH_2C**H**$_3$); δ_C (101 MHz, $CDCl_3$) 174.8 (CO), 160.3 (ArC), 148.5 (ArC), 146.8 (ArC), 139.0 (ArC), 136.2 (ArC), 131.9 (ArC), 130.5 (ArC), 128.4 (ArC), 126.6 (ArC), 124.1 (ArC), 122.4 (ArC), 62.3 (NCH), 36.3 (**C**(CH_3)$_3$), 32.1 (C(**C**H_3)$_3$), 30.0 (CH_2), 20.5 (CH**C**H_3), 9.3 (CH_2**C**H_3); ν_{max}/cm^{-1} (thin film) 2968, 1651, 1589, 1570, 1488, 1435, 1382, 1265, 1233, 1081, 1051, 796, 757; HRMS (ESI); calcd. for $C_{20}H_{26}N_2ONa^+$, 333.1937. Found: $[MNa]^+$, 333.1929 (2.5 error ppm).

***N*-(2-(*tert*-Butyl)phenyl)-*N*-(1-(pyridin-2-yl)ethyl)propionamide (11zd)**

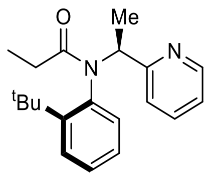

Following the above procedure the title compound **11zd** (22 mg, 12%) was isolated as a minor product, as a colourless oil. R_f 0.20 (8:2 hexane:ethyl acetate); δ_H (400 MHz, $CDCl_3$) 8.59–8.54 (1H, m, ArH), 7.98 (1H, dd, *J* = 8.0, 1.5 Hz, ArH), 7.64 (1H, td, *J* = 7.5, 2.0 Hz, ArH), 7.57 (1H, dd, *J* = 8.0, 1.5 Hz, ArH), 7.44 (1H, d, *J* = 8.0 Hz, ArH), 7.38–7.31 (1H, m, ArH), 7.28–7.22 (1H, m, ArH), 7.15–7.10 (1H, m, ArH), 5.32 (1H, q, *J* = 7.5 Hz, NCH), 2.03–1.84 (2H, m, CH_2), 1.42 (9H, s, C(CH_3)$_3$), 1.13 (3H, d, *J* = 7.5 Hz, CHC**H**$_3$), 0.93 (3H, t, *J* = 7.5 Hz, CH_2C**H**$_3$); δ_C (101 MHz, $CDCl_3$) 174.4 (CO), 163.3 (ArC), 149.1 (ArC), 147.5 (ArC), 137.0 (ArC), 136.3 (ArC), 133.8 (ArC), 130.4 (ArC), 128.4 (ArC), 126.6 (ArC), 122.8 (ArC), 121.7 (ArC), 58.4 (NCH), 36.5 (**C**(CH_3)$_3$), 32.8 (C(**C**H_3)$_3$), 29.5 (CH_2), 20.1 (CH**C**H_3), 9.0 (CH_2**C**H_3); ν_{max}/cm^{-1} (thin film) 2968, 1653, 1592, 1570, 1488, 1474, 1435, 1380, 1273, 1241, 1074, 785, 762, 747; HRMS (ESI); calcd. for $C_{20}H_{27}N_2O^+$, 311.2118. Found: $[MH]^+$, 311.2125 (–2.4 error ppm).

***N*-(2-(*tert*-Butyl)phenyl)-4-iodo-*N*-(1-(pyridin-2-yl)ethyl)benzamide (6ze)**

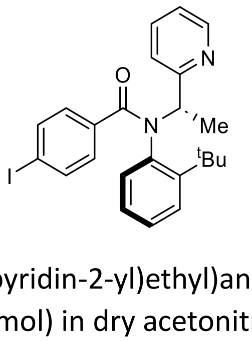

To a mixture of 2-(*tert*-butyl)-*N*-(1-(pyridin-2-yl)ethyl)aniline **4d** (155 mg, 0.605 mmol) and sodium bicarbonate (203 mg, 2.42 mmol) in dry acetonitrile (3 mL) was added 4-iodobenzoyl chloride (322 mg, 1.21 mmol) and the resulting mixture was heated to 80 °C for 1 h. The

reaction was quenched with sat. aq. $NaHCO_3$ (15 mL) and extracted with DCM (3 x 15 mL). The combined organic layers were dried over $MgSO_4$, filtered and concentrated *in vacuo*. At this point, a ^{1}H NMR spectrum of the crude product was taken to determine the *dr* (92:8) of the reaction before chromatography. The crude product was then purified by column chromatography (SiO_2, 79:20:1 hexane:ethyl acetate:triethylamine) to yield the title compound (270 mg, 92%) as a colourless oil. R_f 0.26 (79:20:1 hexane:ethyl acetate:triethylamine); δ_H (400 MHz, $CDCl_3$) 8.42 (1H, d, *J* = 4.5 Hz, ArH), 7.52–7.40 (4H, m, ArH), 7.25–6.97 (7H, m, ArH), 5.75 (1H, q, *J* = 7.0 Hz, NCH), 1.99 (3H, d, *J* = 7.0 Hz, CH_3), 0.94 (9H, s, $C(CH_3)_3$); δ_C (101 MHz, $CDCl_3$) 168.5 (CO), 159.3 (ArC), 148.7 (ArC), 146.9 (ArC), 138.1 (ArC), 136.9 (ArC), 136.5 (ArC), 136.0 (ArC), 133.0 (ArC), 131.3 (ArC), 131.2 (ArC), 128.3 (ArC), 125.9 (ArC), 124.4 (ArC), 122.5 (ArC), 96.0 (ArC), 63.0 (NCH), 36.3 (**C**$(CH_3)_3$), 32.3 ($C($**C**$H_3)_3$), 20.1 (CH**C**H_3); ν_{max}/cm^{-1} (thin film) 2964, 1627, 1584, 1488, 1435, 1392, 1338, 1006, 909, 835, 748, 727; HRMS (ESI); calcd. for $C_{24}H_{26}IN_2O^+$, 485.1084. Found: $[MH]^+$, 485.1082 (0.4 error ppm).

2-(*tert*-Butyl)-*N*-(1-phenylethyl)aniline (14)

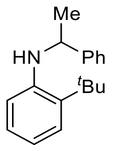

Methyllithium (6.30 mL, 1.6 M in Et_2O, 10.0 mmol) was diluted with THF (20 mL) and cooled to –78 °C. To this was added *N*-(2-(*tert*-butyl)phenyl)-1-phenylmethanimine **S1** (1.19 g, 5.00 mmol), dissolved in dry THF (5 mL), dropwise. The mixture was warmed to room temperature and stirred for 1 h. The reaction was quenched with sat. aq. NH_4Cl (25 mL) and extracted with ethyl acetate (3 x 25 mL). The combined organic layers were dried over $MgSO_4$, filtered and concentrated *in vacuo*. The crude product was then purified by column chromatography (SiO_2, 19:1 hexane:ethyl acetate) to yield the title compound (1.18 g, 93%) as a yellow oil. R_f 0.68 (19:1 hexane:ethyl acetate); δ_H (400 MHz, $CDCl_3$) 7.51–7.45 (2H, m, ArH), 7.44–7.39 (2H, m, ArH), 7.36–7.29 (2H, m, ArH), 7.08–7.01 (1H, m, ArH), 6.73 (1H, td, *J* = 7.5, 1.5 Hz, ArH), 6.50 (1H, dd, *J* = 8.0, 1.5 Hz, ArH), 4.69–4.59 (1H, qd, *J* = 6.5, 4.5 Hz, NCH), 4.43 (1H, d, *J* = 4.0 Hz, NH), 1.68 (3H, d, *J* = 6.5 Hz, CH_3), 1.62 (9H, s, $C(CH_3)_3$); δ_C (101 MHz, $CDCl_3$) 145.6 (ArC), 145.2 (ArC), 132.8 (ArC), 128.8 (ArC), 127.1 (ArC), 126.9 (ArC), 126.2 (ArC), 125.9 (ArC), 116.9 (ArC), 112.8 (ArC), 53.9 (NCH), 34.3 (**C**$(CH_3)_3$), 30.1 ($C($**C**$H_3)_3$), 25.6 (CH_3); ν_{max}/cm^{-1} (thin film) 3487, 2963, 1599, 1578, 1506, 1446, 1370, 1306, 1261, 1205, 1055, 742, 699; HRMS (ESI); calcd. for $C_{18}H_{24}N^+$, 254.1903. Found: $[MH]^+$, 254.1911 (–3.2 error ppm).

***N*-(2-(*tert*-Butyl)phenyl)-*N*-(1-phenylethyl)propionamide (15)**

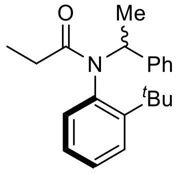

To a mixture of 2-(*tert*-butyl)-*N*-(1-phenylethyl)aniline **14** (100 mg, 0.395 mmol) and sodium bicarbonate (133 mg, 1.58 mmol) in dry acetonitrile (2 mL) was added propionyl chloride

(0.070 mL, 73 mg, 0.790 mmol) and the resulting mixture was heated to 80 °C for 1 h. The reaction was quenched with sat. aq. $NaHCO_3$ (15 mL) and extracted with DCM (3 x 15 mL). The combined organic layers were dried over $MgSO_4$, filtered and concentrated *in vacuo*. The crude product was then purified by column chromatography (SiO_2, 9:1 hexane:ethyl acetate) to yield the title compound (76 mg, 62%) as a 2:1 mixture of diastereoisomers and as a colourless oil. R_f 0.23 (9:1 hexane:ethyl acetate); δ_H (400 MHz, $CDCl_3$) 7.61 (1H, dd, *J* = 8.0, 1.5 Hz, ArH, major diastereoisomer), 7.49–7.45 (2H, m, ArH), 7.39–7.19 (7H, m, ArH), 7.16–7.01 (4H, m, ArH), 6.92–6.85 (4H, m, ArH), 5.66–5.85 (2H, m, NCH, both diastereoisomers), 2.06–1.82 (4H, m, $COCH_2$, both diastereoisomers), 1.70 (3H, d, *J* = 7.0 Hz, CHC**H**$_3$, minor diastereoisomer), 1.42 (9H, s, $C(CH_3)_3$, major diastereoisomer), 1.21 (3H, d, *J* = 7.5 Hz, CHC**H**$_3$, major diastereoisomer), 1.08 (3H, t, *J* = 7.5 Hz, CH_2C**H**$_3$, minor diastereoisomer), 0.99 (3H, t, *J* = 7.5 Hz, CH_2C**H**$_3$, major diastereoisomer), 0.93 (9H, s, $C(CH_3)_3$, minor diastereoisomer); δ_C (101 MHz, $CDCl_3$) 174.2 (CO, major diastereoisomer), 174.1 (CO, minor diastereoisomer), 147.8 (ArC), 147.7 (ArC), 143.9 (ArC), 140.0 (ArC), 137.5 (ArC), 136.6 (ArC), 132.2 (ArC), 131.9 (ArC), 131.2 (ArC), 131.0 (ArC), 130.2 (ArC), 128.4 (2 x ArC), 128.2 (ArC), 218.0 (ArC), 127.7 (2 x ArC), 127.0 (ArC), 126.4 (2 x ArC), 58.6 (NCH, minor diastereoisomer), 55.3 (NCH, major diastereoisomer), 36.7 (**C**$(CH_3)_3$, major diastereoisomer), 36.4 (**C**$(CH_3)_3$, minor diastereoisomer), 32.9 (C(**C**$H_3)_3$, major diastereoisomer), 32.1 (C(**C**$H_3)_3$, minor diastereoisomer), 30.0 (CO**C**H_2, minor diastereoisomer), 29.9 (CO**C**H_2, major diastereoisomer), 22.0 (CH**C**H_3, minor diastereoisomer), 19.5 (CH**C**H_3, major diastereoisomer), 9.4 (CH_2**C**H_3, minor diastereoisomer), 9.1 (CH_2**C**H_3, major diastereoisomer); ν_{max}/cm^{-1} (thin film) 2972, 1652, 1487, 1436, 1381, 1266, 1231, 1052, 918, 760, 733, 699; HRMS (ESI); calcd. for $C_{21}H_{28}NO^+$, 310.2165. Found: $[MH]^+$, 310.2158 (2.2 error ppm).

2-Iodo-4,6-dimethylaniline (S5)

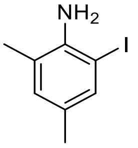

According, to a literature procedure,[3] 2,4-dimethylaniline (4.85 g, 40.0 mmol), iodine (10.2 g, 40.0 mmol) and sodium bicarbonate (5.04 g, 60.0 mmol) were combined in toluene and water (50 mL, 9:1). The reaction was stirred at room temperature for 22 h. The mixture was diluted with ethyl acetate and sat. aq. $Na_2S_2O_3$. The layers were separated, and the aqueous layer was extracted twice with ethyl acetate. The combined organic layers were dried over $MgSO_4$, filtered and concentrated *in vacuo*. The crude mixture was purified by column chromatography (SiO_2, petrol 40-60:diethyl ether 95:5) to yield the title compound (7.35 g, 74%) as a brown solid. δ_H (300 MHz, $CDCl_3$) 7.40–7.33 (1H, m, ArH), 6.88–6.81 (1H, m, ArH), 3.93 (2H, s, NH_2), 2.20 (3H, s, CH_3), 2.19 (3H, s, CH_3). Characterisation data matched those reported in the literature.[3]

N-(2-Iodo-4,6-dimethylphenyl)-1-phenylmethanimine (S6)

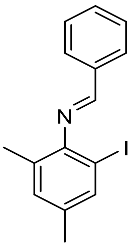

2-Iodo-4,6-dimethylaniline **S5** (4.94 g, 20.0 mmol) was dissolved in ethanol (80 mL) under nitrogen. To this was added benzaldehyde (2.03 mL, 20.0 mmol) and the solution was heated to reflux for 18 h. The mixture was concentrated *in vacuo* and the crude mixture purified by column chromatography (SiO_2, 98:2 petrol 40-60:diethyl ether) and subsequently recrystallised from hexane to yield the title compound (3.10 g, 46%) as a yellow solid. δ_H (400 MHz, $CDCl_3$) 8.21 (1H, s, NCH), 7.99–7.92 (2H, m, ArH), 7.59–7.46 (4H, m, ArH), 7.00 (1H, s, ArH), 2.28 (3H, s, CH_3), 2.17 (3H, s, CH_3). Characterisation data matched those reported in the literature.[4]

(2-Bromo-4,6-dimethylphenyl)-1-phenylmethanimine (S7)

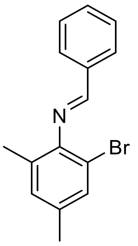

2-Bromo-4,6-dimethylaniline (2.00 g, 10.0 mmol) was dissolved in ethanol (40 mL) under nitrogen. To this was added benzaldehyde (1.02 mL, 10.0 mmol) and the solution was heated to reflux for 20 h. The reaction mixture was concentrated *in vacuo* and the crude mixture was purified by column chromatography (SiO_2, 98:2, petrol 40-60:diethyl ether) and subsequently recrystallised from hexane to yield the title compound (649 mg, 23%) as a yellow solid. R_f 0.34 (98:2 petrol 40-60:diethyl ether); m.p. 53–54 °C; δ_H (400 MHz, $CDCl_3$) 8.27 (1H, s, NCH), 7.94 (2H, dd, *J* = 7.5, 2.0 Hz, ArH), 7.58–7.46 (3H, m, ArH), 7.29 (1H, d, *J* = 2.0 Hz, ArH), 6.97 (1H, d, *J* = 2.0 Hz, ArH), 2.30 (3H, s, CH_3), 2.17 (3H, s, CH_3); δ_C (101 MHz, $CDCl_3$) 165.0 (NCH), 147.9 (ArC), 135.8 (ArC), 134.8 (ArC), 131.9 (ArC), 131.0 (ArC), 130.4 (ArC), 130.0 (ArC), 128.9 (2 x ArC), 113.5 (ArC), 20.6 (CH_3), 18.9 (CH_3); ν_{max}/cm^{-1} (thin film) 3008, 2951, 2918, 1591, 1569, 1473, 1437, 764, 748, 700; HRMS (ESI); calcd. for $C_{15}H_{15}{}^{79}BrN^+$, 288.0382. Found: $[MH]^+$ 288.0388 (2.1 error ppm).

3,5-Dimethyl-[1,1'-biphenyl]-2-amine (S8)

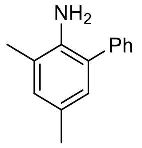

According to a literature procedure,[5] 2-bromo-4,6-dimethylaniline (3.00 g, 15.0 mmol) and phenylboronic acid (2.20 g, 18.0 mmol) were combined in a round bottomed flask. The flask was evacuated and refilled three times with nitrogen. 1,2-Dimethoxyethane (15 mL) and an aqueous 2M solution of K_2CO_3 (30 mL), both degassed by sparging with nitrogen, were added to the flask and the solution was stirred for 30 minutes at room temperature. To this mixture, a solution of $PdCl_2(PPh_3)_2$ (211 mg, 0.30 mmol) in 1,2-dimethoxyethane (15 mL) was added. The reaction was then stirred for 18 h at 80 °C. The mixture was then cooled to room temperature and diluted with ethyl acetate. The layers were separated, and the aqueous layer extracted with 2 x ethyl acetate. The combined organic layers were dried over $MgSO_4$, filtered and concentrated *in vacuo*. The crude mixture was purified by column chromatography (SiO_2, petrol 40-60:ethyl acetate 97:3) to yield the title compound (2.64 g, 89%) as a yellow oil. δ_H (300 MHz, $CDCl_3$) 7.49–7.26 (5H, m, ArH), 6.90 (1H, s, ArH), 6.84 – 6.82 (1H, m, ArH), 3.56 (2H, s, NH_2), 2.25 (3H, s, CH_3), 2.19 (3H, s, CH_3). Characterisation data matched those reported in the literature.[6]

***N*-(5'-(*tert*-Butyl)-[1,1':3',1''-terphenyl]-4'-yl)-1-phenylmethanimine (S9)**

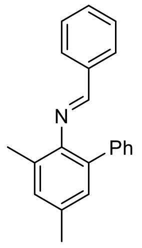

3,5-Dimethyl-[1,1'-biphenyl]-2-amine **S8** (1.97 g, 10.0 mmol) was dissolved in ethanol (40 mL) under nitrogen. To this was added benzaldehyde (1.02 mL, 10.0 mmol) and the solution was heated to reflux for 17 h. The crude mixture was concentrated *in vacuo* and purified by column chromatography (SiO_2, 98:2 petrol 40-60:diethyl ether) and subsequently recrystallised from hexane and ethyl acetate to yield the title compound (1.43 g, 50%) as a yellow solid. R_f 0.43 (98:2 petrol 40-60:diethyl ether); m.p. 91–93 °C; δ_H (400 MHz, $CDCl_3$) 7.88 (1H, s, NCH), 7.58 (2H, dd, *J* = 8.0, 1.5 Hz, ArH), 7.36–7.26 (5H, m, ArH), 7.17 (2H, t, *J* = 7.5 Hz, ArH), 7.09–7.03 (1H, m, ArH), 6.99–6.96 (2H, m, ArH), 2.28 (3H, s, CH_3), 2.15 (3H, s, CH_3); δ_C (101 MHz, $CDCl_3$) 163.6 (NCH), 147.5 (ArC), 140.2 (ArC), 136.4 (ArC), 133.7 (ArC), 132.2 (ArC), 131.2 (ArC), 130.4 (ArC), 130.2 (ArC), 129.4 (ArC), 129.2 (ArC), 128.7 (ArC), 128.4 (ArC), 128.1 (ArC), 126.4 (ArC), 21.0 (CH_3), 18.7 (CH_3); ν_{max}/cm^{-1} (thin film) 3027, 2981, 2916, 1634, 1579, 1470, 1450, 1197, 767, 699, 692; HRMS (ESI); calcd. for $C_{21}H_{20}N^+$, 286.1590. Found: $[MH]^+$, 286.1584 (–2.1 error ppm).

2-Iodo-4,6-dimethyl-*N*-(1-phenyl-2-(pyridin-2-yl)ethyl)aniline (4e)

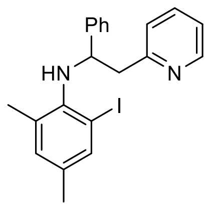

A round bottom flask was evacuated and refilled with N_2 three times, after which diisopropylamine (1.75 mL, 12.5 mmol) and dry THF (30 mL) were added. The solution was cooled to –78 °C and *n*-BuLi (5.00 mL, 2.5 M in hexanes, 12.5 mmol) was added dropwise. The resulting solution was stirred for 15 minutes before 2-picoline (1.23 mL, 12.5 mmol) was added dropwise. The resulting orange solution was stirred for a further 15 minutes before adding a solution of *N*-(2-iodo-4,6-dimethylphenyl)-1-phenylmethanimine **S6** (2.09 g, 6.25 mmol) in dry THF (6.25 mL) dropwise. The reaction mixture was warmed to room temperature and stirred for 1 h. The reaction was quenched with sat. aq. $NaHCO_3$, (40 mL) and the product was extracted with ethyl acetate (3 x 40 mL). The combined organic layers were dried over $MgSO_4$, filtered and concentrated *in vacuo*. The mixture was purified by column chromatography (SiO_2, 85:15 petrol 40-60:ethyl acetate) to yield the title compound (2.08 g, 78%) as a yellow oil. R_f 0.28 (85:15 petrol 40-60:ethyl acetate); δ_H (400 MHz, $CDCl_3$) 8.53–8.47 (1H, m, ArH), 7.43 (1H, td, *J* = 7.5, 2.0 Hz, ArH), 7.38–7.34 (1H, m, ArH), 7.28–7.12 (5H, m, ArH), 7.04 (1H, dd, *J* = 7.5, 5.0 Hz, ArH), 6.97–6.91 (1H, m, ArH), 6.77 (1H, d, *J* = 2.0 Hz, ArH), 4.90 (1H, t, *J* = 7.0 Hz, CH), 4.09 (1H, s, NH), 3.38 (1H, dd, *J* = 13.5, 7.0 Hz, C**H**H'), 3.30 (1H, dd, *J* = 13.5, 7.5 Hz, CH**H'**), 2.17 (3H, s, CH_3), 2.13 (3H, s, CH_3); δ_C (101 MHz, $CDCl_3$) 159.0 (ArC), 149.3 (ArC), 143.8 (ArC), 142.7 (ArC), 137.3 (ArC), 136.0 (ArC), 132.9 (2 x ArC), 130.3 (ArC), 128.4 (ArC), 127.3 (ArC), 127.2 (ArC), 124.2 (ArC), 121.3 (ArC), 95.7 (ArC), 62.1 (CH), 45.9 (CH_2), 20.4 (CH_3), 20.0 (CH_3); ν_{max}/cm^{-1} (thin film) 2981, 1590, 1569, 1472, 1436, 1235, 763, 699; HRMS (ESI); calcd. for $C_{21}H_{22}IN_2^+$, 429.0822. Found: $[MH]^+$, 429.0827 (1.2 error ppm).

2-Bromo-4,6-dimethyl-*N*-(1-phenyl-2-(pyridin-2-yl)ethyl)aniline (4f)

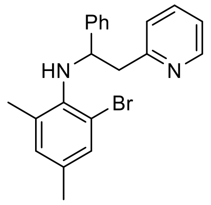

A round bottom flask was evacuated and refilled with N_2 three times, after which diisopropylamine (0.560 mL, 4.00 mmol) and dry THF (9.6 mL) were added. The solution was cooled to –78 °C and *n*-BuLi (1.60 mL, 2.5 M in hexanes, 4.00 mmol) was added dropwise. The resulting solution was stirred for 15 minutes before 2-picoline (0.400 mL, 4.00 mmol) was added dropwise. The resulting orange solution was then stirred for a further 15 minutes and then a solution of *N*-(2-bromo-4,6-dimethylphenyl)-1-phenylmethanimine **S7** (576 mg, 2.00 mmol) in dry THF (2.0 mL) was added dropwise. The reaction mixture warmed to room temperature and stirred for 1 h. The reaction was quenched with sat. aq. $NaHCO_3$ (15 mL), and the product was extracted with ethyl acetate (3 x 15 mL). The combined organic layers were

dried over $MgSO_4$, filtered and concentrated *in vacuo*. The mixture was purified by column chromatography (SiO_2, 9:1 petrol 40-60:ethyl acetate) to yield the title compound (588 mg, 77%) as a pale-yellow oil. R_f 0.16 (9:1 petrol 40-60:ethyl acetate); δ_H (400 MHz, $CDCl_3$) 8.51 (1H, ddd, *J* = 5.0, 2.0, 1.0 Hz, ArH), 7.44 (1H, td, *J* = 7.5, 2.0 Hz, ArH), 7.23–7.12 (5H, m, ArH), 7.09–7.01 (2H, m, ArH), 6.93 (1H, dt, *J* = 8.0, 1.0 Hz, ArH), 6.76–6.71 (1H, m, ArH), 4.91 (1H, t, *J* = 7.0 Hz, CH), 4.36 (1H, s, NH), 3.34 (1H, dd, *J* = 13.5, 7.0 Hz, C**H**H'), 3.28 (1H, dd, *J* = 13.5, 7.0 Hz, CH**H'**), 2.18 (3H, s, CH_3), 2.14 (3H, s, CH_3); δ_C (101 MHz, $CDCl_3$) 158.9 (ArC), 149.3 (ArC), 142.9 (ArC), 141.2 (ArC), 136.0 (ArC), 132.0 (ArC), 131.8 (ArC), 131.0 (ArC), 130.6 (ArC), 128.3 (ArC), 127.2 (ArC), 127.1 (ArC), 124.2 (ArC), 121.4 (ArC), 117.7 (ArC), 61.8 (CH), 46.2 (CH_2), 20.3 (CH_3), 20.2 (CH_3); v_{max}/cm^{-1} (thin film) 3008, 2951, 2918, 1591, 1569, 1473, 1437, 764, 748, 700; HRMS (ESI); calcd. for $C_{21}H_{22}{}^{79}BrN_2^+$, 381.0961. Found: $[MH]^+$, 381.0957 (–1.0 error ppm).

N-(3,5-Dimethyl-[1,1'-biphenyl]-2-yl)-1-phenylmethanimine (4g)

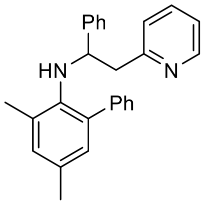

A round bottom flask was evacuated and refilled with nitrogen three times, after which diisopropylamine (0.560 mL, 4.00 mmol) and dry THF (9.6 mL) were added. The solution was cooled to –78 °C and *n*-BuLi (1.60 mL, 2.5 M in hexanes, 4.00 mmol) was added dropwise. The resulting solution was stirred for 15 minutes and 2-picoline (0.400 mL, 4.00 mmol) was added dropwise. The resulting orange solution was then stirred for a further 15 minutes and then a solution of *N*-(3,5-dimethyl-[1,1'-biphenyl]-2-yl)-1-phenylmethanimine **S9** (571 mg, 2.00 mmol) in dry THF (2.0 mL) was added dropwise. The reaction mixture was warmed to room temperature and stirred for 1.5 h. The reaction was quenched with sat. aq. $NaHCO_3$ (15 mL) and the product was extracted with ethyl acetate (3 x 15 mL). The combined organic layers were dried over $MgSO_4$, filtered and concentrated *in vacuo*. The mixture was purified by column chromatography (SiO_2, 9:1, petrol 40-60:ethyl acetate) to yield the title compound (730 mg, 96%) as a yellow oil. R_f 0.21 (9:1 petrol 40-60:ethyl acetate); δ_H (400 MHz, $CDCl_3$) 8.43 (1H, dt, *J* = 5.0, 1.5 Hz, ArH), 7.39 (1H, td, *J* = 7.5, 2.0 Hz, ArH), 7.36–7.31 (3H, m, ArH), 7.22 (2H, dd, *J* = 7.5, 2.0 Hz, ArH), 7.08–7.04 (3H, m, ArH), 7.01 (1H, ddd, *J* = 7.5, 5.0, 1.0 Hz, ArH), 6.85 (1H, d, *J* = 2.0 Hz, ArH), 6.77–6.71 (3H, m, ArH), 6.68 (1H, d, *J* = 8.0 Hz, ArH), 4.36 (1H, t, *J* = 7.0 Hz, CH), 4.24 (1H, s, NH), 3.12–2.97 (2H, m, CH_2), 2.24 (3H, s, CH_3), 2.21 (3H, s, CH_3); δ_C (101 MHz, $CDCl_3$) 159.1 (ArC), 149.0 (ArC), 143.0 (ArC), 141.6 (ArC), 140.8 (ArC), 135.9 (ArC), 133.3 (ArC), 131.2 (ArC), 129.8 (ArC), 129.6 (ArC), 129.4 (ArC), 128.6 (ArC), 128.4 (ArC), 128.1 (ArC), 126.8 (ArC), 126.8 (ArC), 126.7 (ArC), 123.9 (ArC), 121.2 (ArC), 61.2 (CH), 45.6 (CH_2), 20.6 (CH_3), 19.3 (CH_3); v_{max}/cm^{-1} (thin film) 3027, 2981, 2917, 1590, 1569, 1473, 1435, 766, 748, 698; HRMS (ESI); calcd. for $C_{27}H_{27}N_2^+$, 379.2169. Found: $[MH]^+$, 379.2163 (–1.6 error ppm).

N-(2-Iodo-4,6-dimethylphenyl)-N-(1-phenyl-2-(pyridin-2-yl)ethyl)propionamide (6zf)

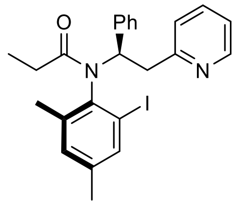

2-Iodo-4,6-dimethyl-*N*-(1-phenyl-2-(pyridin-2-yl)ethyl)aniline **4e** (259 mg, 0.605 mmol) and sodium bicarbonate (203 mg, 2.42 mmol) were added to a round bottom flask under nitrogen with a condenser attached. MeCN (3.0 mL) was added and stirred until **4e** was fully dissolved then propionyl chloride (0.110 mL, 1.21 mmol) was added, and the reaction mixture was heated to 80 °C for 1 h. The reaction was cooled to room temperature and quenched with sat. aq. $NaHCO_3$ (15 mL), and the product was extracted dichloromethane (3 x 15 mL). The combined organic layers were dried over $MgSO_4$, filtered and concentrated *in vacuo*. At this point, a ^{1}H NMR spectrum of the crude product was taken to determine the *dr* (89:11) of the reaction before chromatography. The crude product was then purified by column chromatography (SiO_2, 8:2 petrol 40-60:ethyl acetate) to yield the title compound (222 mg, 76%, 90:10 *dr*) as a white solid. R_f 0.16 (8:2 petrol 40-60:ethyl acetate); m.p. 83–85 °C; δ_H (400 MHz, $CDCl_3$) (*major diastereoisomer*) 8.35 (1H, dt, *J* = 5.0, 1.5 Hz, ArH), 7.58 (1H, s, ArH), 7.39 (1H, td, *J* = 7.5, 2.0 Hz, ArH), 7.16–7.09 (2H, m, ArH), 7.10–6.99 (4H, m, ArH), 6.93 (1H, ddd, *J* = 8.0, 5.0, 1.0 Hz, ArH), 6.87–6.77 (1H, m, ArH), 5.62 (1H, dd, *J* = 11.5, 3.5 Hz, CH), 4.01 (1H, dd, *J* = 13.0, 3.5 Hz, CHC**H**H'), 3.82 (1H, dd, *J* = 13.0, 11.5 Hz, CHCH**H'**), 2.23 (3H, s, CH_3), 2.10 (1H, dq, *J* = 16.5, 7.5 Hz, C**H**H'CH_3), 1.89 (3H, s, CH_3), 1.84–1.74 (1H, m, CH**H'**CH_3), 1.11 (3H, t, *J* = 7.5 Hz, CHH'C**H₃**) (*minor diastereoisomer, diagnostic peaks*): δ_H 7.53 (1H, s, ArH), 7.25–7.22 (2H, m, ArH), 5.10 (1H, dd, *J* = 10.5, 4.5 Hz, CH), 4.21–4.07 (2H, m, CHC**HH'**), 2.42–2.30 (1H, m, C**H**H'CH_3); δ_C (101 MHz, $CDCl_3$) (*major diastereoisomer*) 174.2 (CO), 159.3 (ArC), 149.1 (ArC), 140.5 (ArC), 139.8 (ArC), 139.0 (ArC), 138.7 (ArC), 137.8 (ArC), 136.0 (ArC), 132.3 (ArC), 130.6 (ArC), 127.7 (ArC), 127.6 (ArC), 123.9 (ArC), 121.1 (ArC), 103.6 (ArC), 65.7 (CH), 44.7 (CH**C**HH'), 28.9 (**C**HH'CH_3), 20.5 (CH_3), 20.1 (CH_3), 9.5 (CHH'**C**H_3) (*minor diastereoisomer, diagnostic peaks*): 130.3 (ArC), 127.9 (ArC), 124.2 (ArC), 67.7 (CH), 44.0 (CH**C**HH'), 19.5 (CH_3); ν_{max}/cm^{-1} (thin film) 2935, 1661, 1590, 1460, 1435, 1375, 1247, 793, 700; HRMS (ESI); calcd. for $C_{24}H_{26}IN_2O^+$, 485.1084. Found: $[MH]^+$, 485.1093 (1.9 error ppm).

N-(2-Iodo-4,6-dimethylphenyl)-N-(1-phenyl-2-(pyridin-2-yl)ethyl)benzamide (6zg)

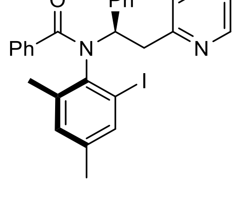

2-Iodo-4,6-dimethyl-*N*-(1-phenyl-2-(pyridin-2-yl)ethyl)aniline **4e** (259 mg, 0.605 mmol) and sodium bicarbonate (203 mg, 2.42 mmol) were added to a 25 mL round bottom flask under nitrogen with a condenser attached. MeCN (3.0 mL) was added and stirred until **4e** had fully dissolved then benzoyl chloride (0.140 mL, 1.21 mmol) was added and the reaction mixture

was heated to 80 °C for 1 h. The reaction was cooled to room temperature and quenched with sat. aq. $NaHCO_3$ (15 mL), and the product was extracted with dichloromethane (3 x 15 mL). The combined organic layers were dried over $MgSO_4$, filtered and concentrated *in vacuo.* At this point, a ^{1}H NMR spectrum of the crude product was taken to determine the *dr* (>95:5) of the reaction before chromatography. The crude product was then purified by column chromatography (SiO_2, 9:1 dichloromethane:diethyl ether) to yield the title compound (259 mg, 80%, >95:5 *dr*) as a white solid. R_f 0.32 (9:1 dichloromethane:diethyl ether); m.p. 165–166 °C; δ_H (400 MHz, $CDCl_3$) 8.44 (1H, dd, *J* = 5.0, 2.0 Hz, ArH), 7.48–7.39 (3H, m, ArH), 7.38–7.31 (2H, m, ArH), 7.29 (1H, d, *J* = 2.0 Hz, ArH), 7.22 (1H, t, *J* = 7.5 Hz, ArH), 7.17–7.08 (6H, m, ArH), 7.03–6.98 (1H, m, ArH), 6.88–6.85 (1H, m, ArH), 5.42 (1H, dd, *J* = 10.5, 4.5 Hz, CH), 4.17 (1H, dd, *J* = 13.5, 10.5 Hz, C**H**H'), 4.09 (1H, dd, *J* = 13.5, 4.5 Hz, CH**H'**), 2.27 (3H, s, CH_3), 2.13 (3H, s, CH_3); δ_C (101 MHz, $CDCl_3$) 170.8 (CO), 159.1 (ArC), 149.0 (ArC), 141.7 (ArC), 139.3 (ArC), 138.9 (ArC), 138.3 (ArC), 137.6 (ArC), 137.2 (ArC), 136.4 (ArC), 132.4 (ArC), 130.8 (ArC), 129.9 (ArC), 128.4 (ArC), 128.0 (ArC), 127.9 (ArC), 127.3 (ArC), 124.4 (ArC), 121.5 (ArC), 103.4 (ArC), 67.7 (CH), 43.1 (CH_2), 20.8 (CH_3), 20.4 (CH_3); ν_{max}/cm^{-1} (thin film) 2992, 1641, 1591, 1445, 1435, 1331, 1293, 909, 728, 698; HRMS (ESI); calcd. for $C_{28}H_{26}IN_2O^+$, 533.1084. Found: $[MH]^+$, 533.1078 (–1.1 error ppm).

N-(2-Bromo-4,6-dimethylphenyl)-N-(1-phenyl-2-(pyridin-2-yl)ethyl)propionamide (6zh)

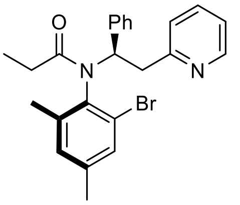

2-Bromo-4,6-dimethyl-*N*-(1-phenyl-2-(pyridin-2-yl)ethyl)aniline **4f** (231 mg, 0.605 mmol) and sodium bicarbonate (203 mg, 2.42 mmol) were added to a round bottom flask under nitrogen with a condenser attached. MeCN (3.0 mL) was added and stirred until **4f** had fully dissolved, then propionyl chloride (0.110 mL, 1.21 mmol) was added and the reaction mixture was heated to 80 °C for 1 h. The reaction was cooled to room temperature and quenched with sat. aq. $NaHCO_3$ (15 mL), and the product was extracted with dichloromethane (3 x 15 mL). The combined organic layers were dried over $MgSO_4$, filtered and concentrated *in vacuo*. At this point, a ^{1}H NMR spectrum of the crude product was taken to determine the *dr* (76:24) of the reaction before chromatography. The crude mixture was purified by column chromatography (SiO_2, 7:3 hexane:ethyl acetate) to yield the title compound (219 mg, 83%, 77:23 *dr*) as a white solid. R_f 0.34 (7:3 hexane:ethyl acetate); m.p. 109–110 °C; δ_H (400 MHz, $CDCl_3$) *(major diastereoisomer)*: 8.36 (1H, dd, *J* = 5.0, 2.0 Hz, ArH), 7.41 (1H, td, *J* = 7.5, 2.0 Hz, ArH), 7.33 (1H, d, *J* = 2.0 Hz, ArH), 7.16–6.98 (6H, m, ArH), 6.94 (1H, dd, *J* = 7.5, 4.0 Hz, ArH), 6.81 (1H, d, *J* = 2.0 Hz, ArH), 5.80 (1H, dd, *J* = 11.5, 3.5 Hz, CH), 4.00 (1H, dt, *J* = 13.5, 3.5 Hz, CHC**H**H'), 3.73 (1H, dd, *J* = 13.5, 11.5 Hz, CHCH**H'**), 2.27 (3H, s, CH_3), 2.08 (1H, dq, *J* = 15.0, 7.5 Hz, C**H**H'CH_3), 1.85–1.79 (1H, m, CH**H'**CH_3), 1.77 (3H, s, CH_3), 1.11 (3H, t, *J* = 7.5 Hz, CHH'C**H₃**) *(minor diastereoisomer, diagnostic peaks)*: 8.41–8.38 (1H, m, ArH), 7.29–7.22 (3H, m, ArH), 5.15 (1H, dd, *J* = 11.0, 4.0 Hz, CH), 4.10 (1H, dd, *J* = 13.5, 11.0 Hz, CHCH**H'**), 1.97 (1H, dt, *J* = 15.5, 7.5 Hz, C**H**H'CH_3), 1.84 (3H, s, CH_3); δ_C (101 MHz, $CDCl_3$) *(major diastereoisomer)*: 174.2 (CO), 159.2

(ArC), 149.1 (ArC), 139.9 (ArC), 139.7 (ArC), 137.6 (ArC), 136.7 (ArC), 136.0 (ArC), 131.9 (ArC), 131.2 (ArC), 130.4 (ArC), 127.7 (ArC), 127.6 (ArC), 125.9 (ArC), 123.8 (ArC), 121.1 (ArC), 64.7 (CH), 43.7 (CH**C**HH'), 28.4 (CH_2, **C**HH'CH_3), 20.8 (CH_3), 19.3 (CH_3), 9.4 (CHH'**C**H_3) *(minor diastereoisomer, diagnostic peaks)*: 174.5 (CO), 139.4 (ArC), 139.1 (ArC), 138.9 (ArC), 138.3 (ArC), 132.1 (ArC), 130.0 (ArC), 127.9 (ArC), 127.6 (ArC), 125.3 (ArC), 124.1 (ArC), 67.4 (CH), 43.6 (CH**C**HH'), 28.7 (**C**H_2CH_3), 19.1 (CH_3), 9.4 (CH_2**C**H_3); v_{max}/cm^{-1} (thin film) 2978, 2937, 1661, 1590, 1472, 1376, 1249, 748, 700; HRMS (ESI); calcd. for $C_{24}H_{26}{}^{79}BrN_2O^+$, 437.1223. Found: $[MH]^+$, 437.1234 (2.5 error ppm).

***N*-(3,5-Dimethyl-[1,1'-biphenyl]-2-yl)-*N*-(1-phenyl-2-(pyridin-2-yl)ethyl)propionamide (6zi)**

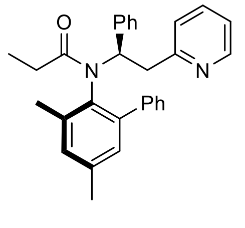

N-(3,5-Dimethyl-[1,1'-biphenyl]-2-yl)-1-phenylmethanimine **4g** (229 mg, 0.605 mmol) and sodium bicarbonate (203 mg, 2.42 mmol) were added to a round bottom flask under nitrogen with a condenser attached. MeCN (3.0 mL) was added and stirred until **4g** was fully dissolved then propionyl chloride (0.110 mL, 1.21 mmol) was added and the reaction mixture was heated to 80 °C for 1 h. The reaction was cooled to room temperature and quenched with sat. aq. $NaHCO_3$ (15 mL) and the product was extracted with dichloromethane (3 x 15 mL). The combined organic layers were dried over $MgSO_4$, filtered and concentrated *in vacuo*. At this point, a ^1H NMR spectrum of the crude product was taken to determine the *dr* (92:8) of the reaction before chromatography. The crude mixture was purified by column chromatography (SiO_2, 7:3 petrol 40-60:ethyl acetate) to yield the title compound (238 mg, 91%, 91:9 *dr*) as a white solid. An analytical sample of each diastereoisomer was subsequently separated by column chromatography (SiO_2, 7:3 petrol 40-60:ethyl acetate).

<u>Data for major diastereoisomer</u>: white solid: R_f 0.23 (7:3 petrol 40-60:ethyl acetate); m.p. 121–122 °C; δ_H (400 MHz, $CDCl_3$, 298 K, 94:6 mixture of rotamers) 8.28–8.15 (1H, m, ArH), 7.44–7.25 (6H, m, ArH), 7.07 (1H, d, *J* = 2.0 Hz, ArH), 7.02–6.82 (7H, m, ArH), 6.80 (1H, d, *J* = 2.0 Hz, ArH), 5.87 (1H, dd, *J* = 12.0, 3.5 Hz, CH), 2.85 (1H, dd, *J* = 13.0, 3.5 Hz, CHC**H**H'), 2.47–2.33 (2H, m, CHCH**H'** and C**H**H'CH_3), 2.30 (3H, s, CH_3), 2.14 (1H, dq, *J* = 16.5, 7.5 Hz, CH**H'**CH_3), 1.67 (3H, s, CH_3), 1.25 (3H, t, *J* = 7.5 Hz, CHH'**C**$\mathbf{H_3}$) (*minor rotamer, diagnostic peaks*) 8.34 (1H, dd, *J* = 4.5, 1.5 Hz, ArH), 7.66–7.55 (2H, m, ArH), 6.31 (1H, d, *J* = 7.5 Hz, ArH), 5.53 (1H, dd, *J* = 12.0, 3.5 Hz, CH), 2.61 (1H, dd, *J* = 13.5, 12.0 Hz, CHC**H**H'); δ_H (400 MHz, d_6-DMSO, 298 K, 86:14 mixture of rotamers) 8.20 (1H, dd, *J* = 5.0, 2.0 Hz, ArH), 7.52–7.44 (2H, m, ArH), 7.44–7.28 (4H, m, ArH), 7.10 (1H, d, *J* = 2.0 Hz, ArH), 7.04–6.89 (4H, m, ArH), 6.89–6.82 (3H, m, ArH), 6.71 (1H, t, *J* = 7.0 Hz, ArH), 6.00 (1H, dd, *J* = 12.0, 3.5 Hz, CH), 2.55 (1H, dd, *J* = 14.0, 4.0 Hz, CHC**H**H'), 2.35–2.19 (5H, m, CHCH**H'**, CH_3C**H**H' and CH_3), 2.16–1.96 (1H, m, CH_3CH**H'**), 1.57 (3H, s, CH_3), 1.10 (3H, t, *J* = 7.5 Hz, C$\mathbf{H_3}$CHH') (*minor rotamer, diagnostic peaks*) 8.31 (1H, d, *J* = 5.0 Hz, ArH), 7.56 (2H, d, *J* = 7.5 Hz, ArH), 7.31 (1H, dd, *J* = 7.5, 2.0 Hz, ArH), 6.50 (1H, d, *J* = 8.0 Hz, ArH), 5.58 (1H, dd, *J* = 12.5, 3.5 Hz, CH), 2.96 (1H, dq, *J* = 15.0, 7.5 Hz, CH_3C**H**H'), 2.77 (1H, dq, *J* = 15.0, 7.5 Hz, CH_3CH**H'**), 1.30 (3H, s, CH_3), 1.26 (3H, t, *J* = 7.5 Hz, C$\mathbf{H_3}$CHH'). N.B. the identity of

the minor species as an amide rotamer is supported by the observation of EXSY correlations with the major isomer (d_6-DMSO, 353 K, mixing time = 300 ms, see section 4); δ_C (101 MHz, $CDCl_3$, 298 K) 174.7 (CO), 159.3 (ArC), 148.9 (ArC), 141.2 (ArC), 140.8 (ArC), 138.3 (ArC), 138.1 (ArC), 136.9 (ArC), 135.7 (ArC), 134.6 (ArC), 131.2 (ArC), 130.4 (ArC), 130.4 (ArC), 129.4 (ArC), 128.7 (ArC), 127.7 (ArC), 127.4 (ArC), 127.4 (ArC), 123.6 (ArC), 120.8 (ArC), 64.1 (CH), 42.4 (CH**C**HH'), 28.8 (**C**HH'CH_3), 21.1 (CH_3), 18.6 (CH_3), 9.7 (CHH'**C**H_3); ν_{max}/cm^{-1} (thin film) 3030, 2981, 2937, 1646, 1434, 1377, 1248, 751, 731, 700; HRMS (ESI); calcd. for $C_{30}H_{31}N_2O^+$, 435.2431. Found: $[MH]^+$, 435.2451 (4.6 error ppm).

Data for minor diastereoisomer: white gummy solid; R_f 0.31 (7:3 petrol 40-60:ethyl acetate); δ_H (400 MHz, $CDCl_3$) 8.25 (1H, d, *J* = 4.5 Hz, ArH), 7.34–7.22 (6H, m, ArH), 7.10–6.93 (7H, m, ArH), 6.83 (1H, dd, *J* = 7.5, 5.0 Hz, ArH), 6.59 (1H, d, *J* = 8.0 Hz, ArH), 4.77 (1H, dd, *J* = 11.5, 3.0 Hz, CH), 3.89 (1H, dd, *J* = 14.0, 11.5 Hz, CHC**H**H'), 2.75 (1H, dd, *J* = 14.0, 3.0 Hz, CHCH**H'**), 2.35 (3H, s, CH_3), 2.32–2.22 (1H, m, C**H**H'CH_3), 2.17–2.01 (1H, m, CH**H'**CH_3), 1.74 (3H, s, CH_3), 1.17 (3H, t, *J* = 7.5 Hz, CHH'C**H**$_3$); δ_C (101 MHz, $CDCl_3$) 174.9 (CO), 159.1 (ArC), 148.7 (ArC), 140.7 (ArC), 140.1 (ArC), 139.3 (ArC), 137.9 (ArC), 137.7 (ArC), 137.1, (ArC), 135.5 (ArC), 131.3 (ArC), 130.5 (ArC), 129.7 (ArC), 129.2 (ArC), 128.4 (ArC), 127.8 (ArC), 127.4 (ArC), 127.1 (ArC), 123.7 (ArC), 120.7 (ArC), 68.0 (CH), 42.4 (CH**C**HH'), 28.9 (**C**HH'CH_3), 21.1 (CH_3), 18.3 (CH_3), 9.6 (CHH'**C**H_3); ν_{max}/cm^{-1} (thin film) 3061, 2981, 2935, 1656, 1592, 1433, 1248, 749, 731, 699; HRMS (ESI); calcd. for $C_{30}H_{31}N_2O^+$, 435.2431. Found: $[MH]^+$, 435.2438 (1.6 error ppm).

_N_-(2-Iodo-4,6-dimethylphenyl)-_N_-(1-phenyl-2-(pyridin-2-yl)ethyl)acrylamide (6zj)

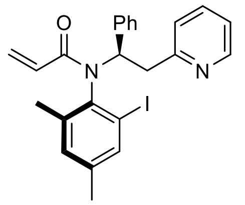

2-Iodo-4,6-dimethyl-*N*-(1-phenyl-2-(pyridin-2-yl)ethyl)aniline **4e** (259 mg, 0.605 mmol) and sodium bicarbonate (203 mg, 2.42 mmol) were added to a round bottom flask under nitrogen with a condenser attached. MeCN (3.0 mL) was added until **4e** had fully dissolved, then acryloyl chloride (98 µL, 1.21 mmol) was added and the reaction mixture was heated to 80 °C for 1 h. The reaction was cooled to room temperature and quenched with sat. aq. $NaHCO_3$ (15 mL), and the product was extracted with dichloromethane (3 x 15 mL). The combined organic layers were dried over $MgSO_4$, filtered and concentrated *in vacuo*. At this point, a ^1H NMR spectrum of the crude product was taken to determine the *dr* (83:17) of the reaction before chromatography. The mixture was purified by column chromatography (SiO_2, 8:2 hexane:ethyl acetate) to yield the title compound (200 mg, 69%, 83:17 *dr*) as a gummy white solid. R_f 0.12 (8:2 hexane:ethyl acetate); δ_H (400 MHz, $CDCl_3$) *(major diastereoisomer)* 8.41–8.32 (1H, m, ArH), 7.57 (1H, d, *J* = 2.0 Hz, ArH), 7.40 (1H, td, *J* = 7.5, 2.0 Hz, ArH), 7.17 (2H, dd, *J* = 8.0, 2.0 Hz, ArH), 7.12–7.01 (4H, m, ArH), 6.95 (1H, ddd, *J* = 7.5, 5.0, 1.0 Hz, ArH), 6.85 (1H, d, *J* = 2.0 Hz, ArH), 6.47 (1H, dd, *J* = 16.5, 2.0 Hz, CH=C**H**H'), 5.82 (1H, dd, *J* = 16.5, 10.5 Hz, C**H**=CHH'), 5.65 (1H, dd, *J* = 11.5, 3.5 Hz, C**H**CHH'), 5.51 (1H, dd, *J* = 10.5, 2.0 Hz, CH=CH**H'**), 4.04 (1H, dd, *J* = 13.0, 3.5 Hz, CHC**H**H'), 3.89 (1H, dd, *J* = 13.5, 11.5 Hz, CHCH**H'**), 2.23 (3H, s, CH_3), 1.90 (3H, s, CH_3) (*minor diastereoisomer, diagnostic peaks*): 7.30 (2H, dd, *J* = 7.5, 2.0 Hz, ArH), 6.92–6.89

(1H, m, ArH), 5.10 (1H, dd, J = 11.5, 3.5 Hz, C**H**CHH'), 4.29 (1H, dd, J = 13.5, 11.5 Hz, CHCH**H'**), 4.15 (1H, dd, J = 13.5, 3.5 Hz, C**H**H'), 2.25 (3H, s, CH_3), 1.65 (3H, s, CH_3); δ_C (101 MHz, $CDCl_3$) *(major diastereoisomer)* 166.2 (CO), 159.0 (ArC), 149.1 (ArC), 140.1 (ArC), 139.8 (ArC), 139.3 (ArC), 138.6 (ArC), 137.5 (ArC), 136.1 (ArC), 132.3 (ArC), 130.5 (ArC), 129.4 (**C**H=CHH), 128.3 (CH=**C**HH), 127.8 (ArC), 127.8 (ArC), 124.0 (ArC), 121.2 (ArC), 103.5 (ArC), 65.8 (**C**HCHH'), 44.2 (CH**C**HH'), 20.5 (CH_3), 20.1 (CH_3) (*minor diastereoisomer, diagnostic peaks*): 166.5 (CO), 140.8 (ArC), 139.9 (ArC), 139.2 (ArC), 138.8 (ArC), 138.4 (ArC), 132.2 (ArC), 130.2 (ArC), 129.9 (ArC), 128.0 (ArC), 124.3 (ArC), 102.6 (ArC), 68.0 (**C**HCHH'), 43.2 (CH**C**HH'), 19.4 (CH_3); ν_{max}/cm^{-1} (thin film) 3030, 2922, 1658, 1615, 1589, 1405, 1252, 728, 699; HRMS (ESI); calcd. for $C_{24}H_{24}IN_2O^+$, 483.0928. Found: $[MH]^+$, 483.0914 (–2.9 error ppm).

***N*-(2-Iodo-4,6-dimethylphenyl)-4-methoxy-*N*-(1-phenyl-2-(pyridin-2-yl)ethyl)benzamide (6zk)**

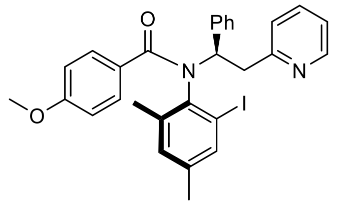

2-Iodo-4,6-dimethyl-*N*-(1-phenyl-2-(pyridin-2-yl)ethyl)aniline **4e** (259 mg, 0.605 mmol) and sodium bicarbonate (203 mg, 2.42 mmol) were added to a round bottom flask under nitrogen with a condenser attached. MeCN (3.0 mL) was added and stirred until **4e** was fully dissolved then 4-methoxybenzoyl chloride (0.160 mL, 1.21 mmol) was subsequently added, and the reaction mixture was heated to 80 °C for 1 h. The reaction was cooled to room temperature and quenched with sat. aq. $NaHCO_3$ (15 mL), and the product was extracted with dichloromethane (3 x 15 mL). The combined organic layers were dried over $MgSO_4$, filtered and concentrated *in vacuo*. At this point, a ^1H NMR spectrum of the crude product was taken to determine the *dr* (>95:5) of the reaction before chromatography. The crude mixture was purified by column chromatography (SiO_2, 7:3 petrol 40-60:ethyl acetate) to yield the title compound (299 mg, 88%, >95:5 *dr*) as a white solid. R_f 0.19 (7:3 petrol 40-60:ethyl acetate); m.p. 156–157 °C; δ_H (400 MHz, $CDCl_3$) 8.44 (1H, dd, J = 5.0, 2.0 Hz, ArH), 7.53–7.42 (1H, m, ArH), 7.40–7.32 (4H, m, ArH), 7.30 (1H, d, J = 2.0 Hz, ArH), 7.20–7.10 (4H, m, ArH), 7.06–7.00 (1H, m, ArH), 6.89 (1H, d, J = 2.0 Hz, ArH), 6.62 (2H, d, J = 9.0 Hz, ArH), 5.38 (1H, dd, J = 10.5, 4.5 Hz, CH), 4.20 (1H, dd, J = 13.5, 10.5 Hz, C**H**H'), 4.08 (1H, dd, J = 13.5, 4.5 Hz, CH**H'**), 3.71 (3H, s, OCH_3), 2.27 (3H, s, CH_3), 2.15 (3H, s, CH_3); δ_C (101 MHz, $CDCl_3$) 170.3 (CO), 160.8 (ArC), 159.1 (ArC), 148.6 (ArC), 142.1 (ArC), 139.2 (ArC), 138.9 (ArC), 138.2 (ArC), 137.5 (ArC), 136.7 (ArC), 132.4 (ArC), 130.8 (ArC), 130.5 (ArC), 129.5 (ArC), 128.0 (ArC), 127.9 (ArC), 124.6 (ArC), 121.6 (ArC), 112.6 (ArC), 103.4 (ArC), 67.7 (CH), 55.2 (OCH_3), 42.7 (CH_2), 20.7 (CH_3), 20.4 (CH_3); ν_{max}/cm^{-1} (thin film) 2981, 1628, 1594, 1334, 1248, 1174, 1030, 846, 759, 693; HRMS (ESI); calcd. for $C_{29}H_{28}IN_2O_2^+$, 563.1190. Found: $[MH]^+$, 563.1204 (2.5 error ppm).

N-(2-Iodo-4,6-dimethylphenyl)-*N*-(1-phenyl-2-(pyridin-2-yl)ethyl)furan-2-carboxamide (6zl)

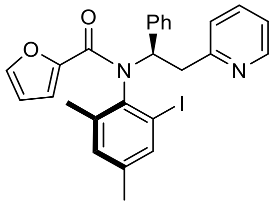

2-Iodo-4,6-dimethyl-*N*-(1-phenyl-2-(pyridin-2-yl)ethyl)aniline **4e** (259 mg, 0.605 mmol) and sodium bicarbonate (203 mg, 2.42 mmol) were added to a round bottom flask under nitrogen with a condenser attached. MeCN (3.0 mL) was added and stirred until **4e** had fully dissolved, then 2-furoyl chloride (0.120 mL, 1.21 mmol) was added and the reaction mixture was heated to 80 °C for 1 h. The reaction was cooled to room temperature and quenched with sat. aq. $NaHCO_3$ (15 mL) and the product was extracted with dichloromethane (3 x 15 mL). The combined organic layers were dried over $MgSO_4$, filtered and concentrated *in vacuo*. At this point, a ^{1}H NMR spectrum of the crude product was taken to determine the *dr* (>95:5) of the reaction before chromatography. The mixture was purified by column chromatography (SiO_2, 7:3 petrol 40-60:ethyl acetate) to yield the title compound (238 mg, 75%, >95:5 *dr*) as a white solid. R_f 0.18 (7:3 petrol 40-60:ethyl acetate); m.p. 156–157 °C; δ_H (400 MHz, $CDCl_3$) 8.39 (1H, d, *J* = 4.5 Hz, ArH), 7.50 (1H, d, *J* = 2.0 Hz, ArH), 7.42 (1H, td, *J* = 7.5, 2.0 Hz, ArH), 7.37 (1H, d, *J* = 1.5 Hz, ArH), 7.31 (2H, dd, *J* = 7.5, 2.0 Hz, ArH), 7.15–7.06 (4H, m, ArH), 6.99–6.94 (2H, m, ArH), 6.18 (1H, dd, *J* = 3.5, 1.5 Hz, ArH), 5.57 (1H, d, *J* = 3.5 Hz, ArH), 5.50 (1H, dd, *J* = 10.5, 4.0 Hz, CH), 4.15–3.99 (2H, m, CH_2), 2.27 (3H, s, CH_3), 2.10 (3H, s, CH_3); δ_C (101 MHz, $CDCl_3$) 159.5 (CO), 159.1 (ArC), 149.1 (ArC), 147.9 (ArC), 144.7 (ArC), 140.8 (ArC), 140.2 (ArC), 139.3 (ArC), 138.8 (ArC), 137.3 (ArC), 136.2 (ArC), 132.5 (ArC), 130.8 (ArC), 128.0 (ArC), 127.9 (ArC), 124.2 (ArC), 121.3 (ArC), 115.3 (ArC), 111.4 (ArC), 103.2 (ArC), 67.6 (CH), 43.6 (CH_2), 20.6 (CH_3), 20.3 (CH_3); ν_{max}/cm^{-1} (thin film) 2923, 1711, 1638, 1591, 1471, 1334, 751, 729, 699; HRMS (ESI); calcd. for $C_{26}H_{24}IN_2O_2^+$, 523.0877. Found: $[MH]^+$, 523.0864 (–2.5 error ppm).

3-((2-(*tert*-Butyl)phenyl)amino)butan-1-ol (S10)

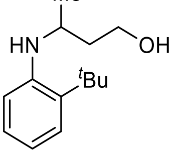

To a stirred solution of 1-hydroxy-butan-3-one (0.150 mL, 150 mg, 1.70 mmol) in 1,2-dichloroethane (3.5 mL) was added 2-*tert*-butylaniline (0.300 mL, 284 mg, 1.87 mmol), acetic acid (0.110 mL, 114 mg, 1.87 mmol) and sodium triacetoxyborohydride (551 mg, 2.55 mmol). The reaction was heated to 60 °C for 15 h. The solution was diluted with dichloromethane (20 mL) and sat. aq. $NaHCO_3$ (10 mL) and the aqueous layer was extracted with dichloromethane (2 x 10 mL). The combined organic layers were washed with brine (30 mL), then dried over $MgSO_4$, filtered and concentrated *in vacuo*. Purification by flash column chromatography (SiO_2, 9:1 hexane:ethyl acetate) gave the title compound (240 mg, 64%) as a yellow oil; R_f 0.07 (9:1 hexane:ethyl acetate); δ_H (400 MHz, $CDCl_3$) 7.26–7.24 (1H, m, ArH), 7.15–7.11 (1H, m,

ArH), 6.79–6.77 (1H, m, ArH), 6.71–6.67 (1H, m, ArH), 3.90–3.81 (4H, m, C**HH'**OH, NCH, NH or OH), 2.13–2.12 (1H, m, NH or OH), 1.89–1.83 (2H, m, CH_2), 1.42 (9H, s, $C(CH_3)_3$), 1.26 (3H, d, *J* = 6.0 Hz, CH_3); δ_C (101 MHz, $CDCl_3$) 145.3 (ArC), 133.6 (ArC), 127.1 (ArC), 126.5 (ArC), 117.1 (ArC), 112.6 (ArC), 61.0 (CH_2OH), 47.4 (NCH), 39.6 (CH_2), 34.1 (**C**$(CH_3)_3$), 30.1 (C(**C**$H_3)_3$), 20.8 (CH_3); ν_{max}/cm^{-1} (thin film) 3351, 2959, 1507, 1446, 1054, 741; HRMS (ESI); calcd. for $C_{14}H_{24}NO^+$, 222.1852. Found: $[MH]^+$, 222.1853 (–1.3 error ppm)

***N*-(4-Bromobutan-2-yl)-2-(*tert*-butyl)aniline (S11)**

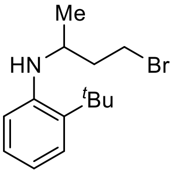

To a stirred solution of 3-((2-(*tert*-butyl)phenyl)amino)butan-1-ol **S10** (1.31 g, 5.92 mmol) in dichloromethane (20 mL) was added tetrabromomethane (2.94 g, 8.88 mmol), and the resulting yellow solution was cooled to 0 °C. Triphenylphosphine (2.33 g, 8.88 mmol) was added in portions and the reaction was stirred for 5 min at 0 °C, then allowed to warm to room temperature and continued stirring for 2 h. The reaction was quenched with sat. aq. $NaHCO_3$ (15 mL) and extracted with dichloromethane (3 x 15 mL). The combined organic layers were washed sequentially with sat. aq. $NaHCO_3$ (50 mL) and brine (50 mL), then dried over $MgSO_4$, filtered and concentrated *in vacuo*. The crude product was redissolved in diethyl ether (50 mL) then filtered and concentrated *in vacuo*. Purification by flash column chromatography (SiO_2, 9:1 hexane:dichloromethane → 19:1 hexane:ethyl acetate) gave the title compound (1.37 g, 82%) as a yellow oil. R_f 0.39 (19:1 hexane:ethyl acetate); δ_H (400 MHz, $CDCl_3$) 7.28–7.25 (1H, m, ArH), 7.17–7.12 (1H, m, ArH), 6.78–6.75 (1H, m , ArH), 6.72–6.68 (1H, m, ArH), 3.90–3.85 (1H, m, NCH) 3.72–3.65 (1H, m, NH), 3.60–3.49 (2H, m, C**HH'**Br), 2.26–2.18 (1H, m, C**H**H'), 2.12–2.03 (1H, m, CH**H'**), 1.43 (9H, s, $C(CH_3)_3$), 1.29 (3H, d, *J* = 6.0 Hz, CH_3); δ_C (101 MHz, $CDCl_3$) 145.1 (ArC), 133.1 (ArC), 127.2 (ArC), 126.4 (ArC), 116.9 (ArC), 112.1 (ArC), 47.3 (NCH), 40.4 (CH_2), 34.0 (**C**$(CH_3)_3$) 30.3 (CH_2Br), 30.1 (C(**C**$H_3)_3$) 20.6 (CH_3); ν_{max}/cm^{-1} (thin film) 3477, 2966, 1600, 1578, 1508, 1447, 1309, 1258, 744; HRMS (ESI); calcd. for $C_{14}H_{23}{}^{79}BrN^+$, 284.1008. Found: $[MH]^+$, 284.1006 (0.9 error ppm).

***N*¹-Benzyl-*N*¹-methyl-*N*³-phenylbutane-1,3-diamine (7a)**

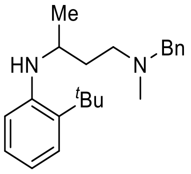

To a stirred solution of *N*-(4-bromobutan-2-yl)-2-(*tert*-butyl)aniline **S11** (1.00 g, 3.52 mmol) in acetonitrile (23 mL) was added *N*-benzylmethylamine (0.680 mL, 640 mg, 5.28 mmol) and triethylamine (1.47 mL, 1.07 g, 10.6 mmol) and the reaction was heated to reflux for 60 h. On completion the solution was diluted with ethyl acetate (80 mL) and sat. aq. $NaHCO_3$ (40 mL). The aqueous layer was extracted with ethyl acetate (2 x 60 mL) and the combined organic layers were washed with brine (60 mL), then dried over $MgSO_4$, filtered and concentrated *in vacuo*. Purification by flash column chromatography (SiO_2, 1:1 hexane:ethyl acetate) gave the title compound (663 mg, 58%) as a yellow oil. R_f 0.38 (1:1 hexane:ethyl acetate); δ_H (400 MHz, $CDCl_3$) 7.33–7.22 (6H, m, ArH), 7.14–7.09 (1H, m, ArH), 6.72–6.70 (1H, m, ArH), 6.68–6.63 (1H, m, ArH), 3.87 (1H, d, *J* = 8.0 Hz, NH), 3.74–3.67 (1H, m, NCH), 3.56 (1H, d, *J* = 13.0 Hz, C**H**H'Ph),

3.48 (1H, d, J = 13.0 Hz, CH**H'**Ph), 2.58–2.46 (2H, m, NCH_2), 2.24 (3H, s, NCH_3), 1.90–1.69 (2H, m, CH_2), 1.40 (9H, s, $C(CH_3)_3$), 1.21 (3H, d, J = 6.5 Hz, CH_3); δ_C (101 MHz, $CDCl_3$) 145.4 (ArC), 138.9 (ArC), 132.9 (ArC), 129.0 (ArC), 128.2 (ArC), 127.1 (ArC), 126.9 (ArC), 126.3 (ArC), 116.2 (ArC), 111.8 (ArC), 62.6 (CH_2Ph), 54.3 (NCH_2), 46.9 (NCH), 42.4 (NCH_3), 35.0 (CH_2), 34.0 (**C**$(CH_3)_3$), 29.9 (C(**C**$H_3)_3$), 20.7 (CH_3); ν_{max}/cm^{-1} (thin film) 2961, 1600, 1508, 1448, 740, 699; HRMS (ESI); calcd. for $C_{22}H_{33}N_2^+$, 325.2638. Found: $[MH]^+$, 325.2644 (0.5 error ppm).

***N*^3^-(2-(*tert*-Butyl)phenyl)-*N*^1^,*N*^1^-dipropylbutane-1,3-diamine (7b)**

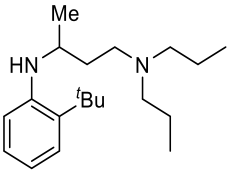

To a stirred solution of *N*-(4-bromobutan-2-yl)-2-(*tert*-butyl)aniline **S11** (200 mg, 0.704 mmol) in acetonitrile (4 mL) was added dipropylamine (0.140 mL, 107 mg, 1.06 mmol) and triethylamine (0.290 mL, 214 mg, 2.11 mmol) and the reaction was heated to reflux for 30 h. On completion the solution was diluted with ethyl acetate (20 mL) and sat. aq. $NaHCO_3$ (10 mL). The aqueous layer was extracted with ethyl acetate (2 x 20 mL) and the combined organic layers were washed with brine (15 mL) and dried over $MgSO_4$, filtered and concentrated *in vacuo*. Purification by flash column chromatography (SiO_2, 9:1 ethyl acetate:hexane) gave the title compound (86 mg, 40%) as a yellow oil. R_f 0.17 (9:1 ethyl acetate:hexane); δ_H (400 MHz, $CDCl_3$) 7.24–7.22 (1H, m, ArH), 7.12–7.08 (1H, m, ArH), 6.70–6.62 (2H, m, ArH), 3.82 (1H, d, J = 8.0 Hz, NH), 3.68–3.61 (1H, m, NCH), 2.64–2.48 (2H, m, NC**HH'**), 2.44–2.32 (4H, m, 2 x NC**HH'**), 1.83–1.76 (1H, m, NCHC**H**H'), 1.68–1.59 (1H, m, NCHCH**H'**), 1.49–1.43 (4H, m, CH_3C**H**$_2$), 1.42 (9H, s, $C(CH_3)_3$), 1.23 (3H, d, J = 6.5 Hz, CHC**H**$_3$), 0.87 (6H, t, J = 7.5 Hz, CH_2C**H**$_3$); δ_C (101 MHz, $CDCl_3$) 145.6 (ArC), 133.0 (ArC), 127.2 (ArC), 126.5 (ArC), 116.3 (ArC), 112.0 (ArC), 56.5 (NCH_2), 51.4 (NCH_2), 47.1 (NCH), 35.1 (NCH**C**H_2), 34.2 (**C**$(CH_3)_3$), 30.1 (C(**C**$H_3)_3$), 20.9 (CH_3), 20.2 (CH_3**C**H_2), 12.1 (CH_2**C**H_3); ν_{max}/cm^{-1} (thin film) 2957, 2932, 1507, 1447, 741; HRMS (ESI); calcd. for $C_{20}H_{37}N_2^+$, 305.2951. Found: $[MH]^+$, 305.2957 (–2.3 error ppm).

***N*-(4-(Benzyl(methyl)amino)butan-2-yl)-*N*-(2-(*tert*-butyl)phenyl)benzamide (9a)**

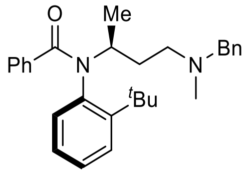

To a stirred solution of *N*^1^-benzyl-*N*^1^-methyl-*N*^3^-phenylbutane-1,3-diamine **7a** (100 mg, 0.308 mmol) in acetonitrile (1.5 mL) was added sodium bicarbonate (104 mg, 1.23 mmol) and benzoyl chloride (0.070 mL, 87 mg, 0.619 mmol), and the reaction was heated to 80 °C for 1 h. The reaction was quenched with sat. aq. $NaHCO_3$ (15 mL) and the aqueous layer was extracted with dichloromethane (3 x 15 mL). The combined organic layers were dried over $MgSO_4$, filtered and concentrated *in vacuo*. At this point, a ^{1}H NMR spectrum of the crude

product was taken to determine the *dr* (>95:5) of the reaction before chromatography. Purification by flash column chromatography (SiO_2, 3:2 ethyl acetate:hexane) gave the title compound (109 mg, 82%) as a white solid. R_f 0.68 (9:1 ethyl acetate:hexane); m.p. 93–97 °C; δ_H (400 MHz, $CDCl_3$) 7.32–7.20 (10H, m, ArH), 7.18–7.14 (2H, m, ArH), 7.09–7.06 (2H, m, ArH), 4.56–4.51 (1H, m, NCH), 3.47 (1H, d, *J* = 12.5 Hz, C**H**H'Ph), 3.36 (1H, d, *J* = 12.5 Hz, CH**H'**Ph), 2.44–2.40 (2H, m, NC**HH'**), 2.10 (3H, s, NCH_3), 1.66–1.57 (1H, m, C**H**H'), 1.54 (3H, d, *J* = 6.5 Hz, CH_3), 1.26–1.18 (1H, m, CH**H'**), 1.07 (9H, s, $C(CH_3)_3$); δ_C (101 MHz, $CDCl_3$) 169.1 (CO), 147.2 (ArC), 139.0 (ArC), 137.5 (ArC), 137.4 (ArC), 132.1 (ArC), 131.3 (ArC), 129.6 (ArC), 129.4 (ArC), 129.1 (ArC), 128.3 (ArC), 128.2 (ArC), 127.4 (ArC), 127.1 (ArC), 126.0 (ArC), 62.6 (CH_2Ph), 54.8 (NCH_2), 53.0 (NCH), 42.3 (NCH_3), 36.4 (**C**$(CH_3)_3$), 32.4 (C(**C**$H_3)_3$), 20.2 (CH_3); ν_{max}/cm^{-1} (thin film) 2964, 1632, 1489, 1371, 1351, 698; HRMS (ESI); calcd. for $C_{29}H_{37}N_2O^+$, 429.2900. Found: $[MH]^+$, 429.2911 (–2.6 error ppm).

_N_-(4-(Benzyl(methyl)amino)butan-2-yl)-_N_-(2-(_tert_-butyl)phenyl)propionamide (9b)

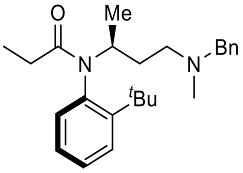

To a stirred solution of N^1-benzyl-N^1-methyl-N^3-phenylbutane-1,3-diamine **7a** (100 mg, 0.308 mmol) in acetonitrile (1.5 mL) was added sodium bicarbonate (104 mg, 1.23 mmol) and propionyl chloride (0.050 mL, 57 mg, 0.616 mmol) and the reaction was heated to 80 °C for 1 h. The reaction was quenched with sat. aq. $NaHCO_3$ (15 mL) and the aqueous layer was extracted with dichloromethane (3 x 15 mL). The combined organic layers were dried over $MgSO_4$, filtered and concentrated *in vacuo*. At this point, a ^1H NMR spectrum of the crude product was taken to determine the *dr* (>95:5) of the reaction before chromatography. Purification by flash column chromatography (SiO_2, 3:2 ethyl acetate:hexane) gave the title compound (89 mg, 75%) as a colourless oil. R_f 0.65 (9:1 ethyl acetate:methanol); δ_H (400 MHz, $CDCl_3$) 7.56–7.54 (1H, m, ArH), 7.33–7.14 (7H, m, ArH), 6.97–6.95 (1H, m, ArH), 4.22–4.16 (1H, m, NCH), 3.45 (1H, d, *J* = 13.0 Hz, C**H**H'Ph), 3.33 (1H, d, *J* = 13.0 Hz, CH**H'**Ph), 2.41–2.28 (2H, m, NCH_2), 2.08 (3H, s, NCH_3), 1.98–1.85 (2H, m, CH_2CO), 1.76–1.68 (1H, m, C**H**H'), 1.37 (9H, s, $C(CH_3)_3$), 1.35 (3H, d, *J* = 6.5 Hz, CH_3), 1.22–1.13 (1H, m, CH**H'**), 1.01 (3H, t, *J* = 7.5 Hz, C**H**$_3CH_2$); δ_C (101 MHz, $CDCl_3$) 174.1 (CO), 147.1 (ArC), 139.1 (ArC), 138.3 (ArC), 131.3 (ArC), 130.8 (ArC), 129.1 (ArC), 128.4 (ArC), 128.3 (ArC), 127.1 (ArC), 126.8 (ArC), 62.5 (CH_2Ph), 54.7 (NCH_2), 52.7 (NCH), 42.3 (NCH_3), 36.6 (**C**$(CH_3)_3$), 32.8 (C(**C**$H_3)_3$), 31.8 (CH_2), 29.9 (**C**H_2CO), 19.4 (**C**H_3CH), 9.4 (**C**H_3CH_2); ν_{max}/cm^{-1} (thin film) 2967, 1654, 1488, 1378, 1272, 738; HRMS (ESI); calcd. for $C_{25}H_{37}N_2O^+$, 381.2900. Found: $[MH]^+$, 381.2895 (1.4 error ppm).

N-(4-(Benzyl(methyl)amino)butan-2-yl)-N-(2-(tert-butyl)phenyl)acrylamide (9c)

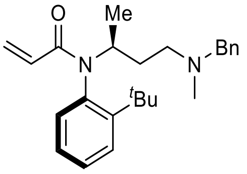

To a stirred solution of N^1-benzyl-N^1-methyl-N^3-phenylbutane-1,3-diamine **7a** (100 mg, 0.308 mmol) in acetonitrile (1.5 mL) was added sodium bicarbonate (104 mg, 1.23 mmol) and acryloyl chloride (0.050 mL, 56 mg, 0.619 mmol) and the reaction was heated to 80 °C for 1 h. The reaction was quenched with sat. aq. $NaHCO_3$ (15 mL) and the aqueous layer was extracted with dichloromethane (3 x 15 mL). The combined organic layers were dried over $MgSO_4$, filtered and concentrated *in vacuo*. At this point, a ^{1}H NMR spectrum of the crude product was taken to determine the *dr* (>95:5) of the reaction before chromatography. Purification by flash column chromatography (SiO_2, 1:1 hexane:ethyl acetate) gave the title compound (69 mg, 59%) as a colourless oil. R_f 0.71 (4:1 ethyl acetate:methanol); δ_H (400 MHz, $CDCl_3$) 7.56–7.54 (1H, m, ArH), 7.33–7.13 (7H, m, ArH), 6.98–6.95 (1H, m, ArH), 6.32 (1H, dd, *J* = 16.5, 2.0 Hz, C**H**H'=CH), 5.84 (1H, dd, *J* = 16.5, 10.5 Hz, CH_2=C**H**), 5.41 (1H, dd, *J* = 10.5, 2.0 Hz, CH**H'**=CH), 4.29–4.21 (1H, m, NCH), 3.45 (1H, d, *J* = 13.0 Hz, C**H**H'Ph), 3.31 (1H, d, *J* = 13.0 Hz, CH**H'**Ph), 2.41–2.29 (2H, m, NCH_2), 2.07 (3H, s, NCH_3), 1.77–1.69 (1H, m, C**H**H'), 1.40 (3H, d, *J* = 6.0 Hz, CH_3), 1.34 (9H, s, $C(CH_3)_3$), 1.27–1.17 (1H, m, CH**H'**); δ_C (101 MHz, $CDCl_3$) 165.8 (CO), 147.7 (ArC), 139.1 (ArC), 137.6 (ArC), 131.5 (ArC), 130.8 (ArC), 130.5 (**C**H=CHH'), 129.1 (ArC), 128.5 (ArC), 128.3 (ArC), 127.0 (ArC), 126.9 (CH=**C**HH'), 126.8 (ArC), 62.5 (CH_2Ph), 54.7 (NCH_2), 53.1 (NCH), 42.3 (NCH_3), 36.6 (**C**$(CH_3)_3$), 32.7 (C(**C**$H_3)_3$), 31.8 (CH_2), 19.2 (CH_3); ν_{max}/cm^{-1} (thin film) 2964, 1656, 1488, 1407, 1268, 760, 736, 699; HRMS (ESI); calcd. for $C_{25}H_{35}N_2O^+$, 379.2744. Found: $[MH]^+$, 379.2741 (1.0 error ppm).

N-(4-(Benzyl(methyl)amino)butan-2-yl)-N-(2-(tert-butyl)phenyl)-2-methoxyacetamide (9d)

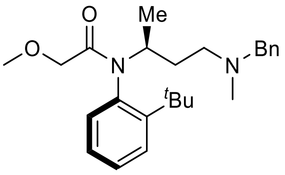

To a stirred solution of N^1-benzyl-N^1-methyl-N^3-phenylbutane-1,3-diamine **7a** (100 mg, 0.308 mmol) in acetonitrile (1.5 mL) was added sodium bicarbonate (104 mg, 1.23 mmol) and methoxyacetyl chloride (0.060 mL, 67 mg, 0.617 mmol), and the reaction was heated to 80 °C for 1 h. The reaction was quenched with sat. aq. $NaHCO_3$ (15 mL) and the aqueous layer was extracted with dichloromethane (3 x 15 mL). The combined organic layers were dried over $MgSO_4$, filtered and concentrated *in vacuo*. At this point, a ^{1}H NMR spectrum of the crude product was taken to determine the *dr* (>95:5) of the reaction before chromatography. Purification by flash column chromatography (SiO_2, 1:1 hexane:ethyl acetate) gave the title compound (62 mg, 50%) as a white solid. R_f 0.50 (9:1 ethyl acetate:methanol); m.p. 42–45 °C;

δ_H (400 MHz, $CDCl_3$) 7.57–7.55 (1H, m, ArH), 7.36–7.15 (7H, m, ArH), 6.98–6.96 (1H, m, ArH), 4.24–4.19 (1H, m, NCH), 3.62 (1H, d, *J* = 15.0 Hz, C**H**H'O), 3.58 (1H, d, *J* = 15.0 Hz, CH**H'**O), 3.44 (1H, d, *J* = 13.0 Hz, C**H**H'Ph), 3.34 (3H, s, OCH_3), 3.32 (1H, d, *J* = 13.0 Hz, CH**H'**Ph), 2.39–2.32 (2H, m, NCH_2), 2.08 (3H, s, NCH_3), 1.75–1.67 (1H, m, C**H**H'CH), 1.40–1.38 (12H, m, CH_3 and $C(CH_3)_3$), 1.25–1.16 (1H, m, CH**H'**CH); δ_C (101 MHz, $CDCl_3$) 169.3 (CO), 147.3 (ArC), 139.0 (ArC), 136.1 (ArC), 131.3 (ArC), 131.1 (ArC), 129.1 (ArC), 129.0 (ArC), 128.3 (ArC), 127.1 (2 x ArC), 72.1 (CH_2O), 62.5 (CH_2Ph), 59.5 (CH_3O), 54.6 (NCH_2), 53.1 (NCH), 42.4 (NCH_3), 36.7 (**C**$(CH_3)_3$), 32.8 (C(**C**$H_3)_3$), 31.5 (**C**H_2CH), 19.2 (CH_3); ν_{max}/cm^{-1} (thin film) 2964, 2791, 1670, 1488, 1279, 1199, 1131, 763, 739, 700; HRMS (ESI); calcd. for $C_{25}H_{37}N_2O_2^+$, 397.2850. Found: $[MH]^+$, 397.2853 (–1.7 ppm error).

***N*-(2-(*tert*-Butyl)phenyl)-*N*-(4-(dipropylamino)butan-2-yl)benzamide (9e)**

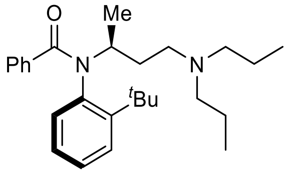

To a stirred solution of N^3-(2-(*tert*-butyl)phenyl)-N^1,N^1-dipropylbutane-1,3-diamine **7b** (100 mg, 0.328 mmol) in acetonitrile (1.7 mL) was added sodium bicarbonate (110 mg, 1.31 mmol) and benzoyl chloride (0.080 mL, 92 mg, 0.654 mmol), and the reaction was heated to 80 °C for 1 h. The reaction was quenched with sat. aq. $NaHCO_3$ (15 mL) and the aqueous layer was extracted with dichloromethane (3 x 15 mL). The combined organic layers were dried over $MgSO_4$, filtered and concentrated *in vacuo*. At this point, a ^1H NMR spectrum of the crude product was taken to determine the *dr* (>95:5) of the reaction before chromatography. Purification by flash column chromatography (SiO_2, ethyl acetate) gave the title compound (92 mg, 68%) as a colourless oil. R_f 0.38 (9:1 ethyl acetate:methanol); δ_H (400 MHz, $CDCl_3$) 7.28–7.24 (2H, m ArH), 7.21–7.11 (5H, m, ArH), 7.07–7.03 (2H, m, ArH), 4.49–4.43 (1H, m, NCH), 2.48–2.41 (2H, m, NC**HH'**), 2.26–2.21 (4H, m, 2 x NC**HH'**), 1.57 (3H, d, *J* = 6.5 Hz, CH_3), 1.53–1.46 (1H, m, CHC**H**H'), 1.38–1.32 (4H, m, 2 x CH_3CH_2), 1.13–1.07 (1H, m, CHCH**H'**), 1.04 (9H, s, $C(CH_3)_3$), 0.78 (6H, t, *J* = 7.5 Hz, 2 x C**H**$_3CH_2$); δ_C (101 MHz, $CDCl_3$) 169.0 (CO), 147.1 (ArC), 137.4 (ArC), 137.3 (ArC), 132.0 (ArC), 131.2 (ArC), 129.6 (ArC), 129.3 (ArC), 128.1 (ArC), 127.3 (ArC), 125.9 (ArC), 56.3 (NCH_2), 53.0 (NCH), 51.5 (NCH_2), 36.3 (**C**$(CH_3)_3$), 32.4 (C(**C**$H_3)_3$), 31.9 (CH**C**H_2), 20.3 (CH_3), 20.3 (CH_3**C**H_2), 12.0 (**C**H_3CH_2); ν_{max}/cm^{-1} (thin film) 2960, 2872, 1632, 1489, 1371, 1350, 760, 716; HRMS (ESI); calcd. for $C_{27}H_{41}N_2O^+$, 409.3213. Found: $[MH]^+$, 409.3218 (–1.7 ppm error).

***N*-(4-(Benzyl(methyl)amino)butan-2-yl)-*N*-(2-(*tert*-butyl)phenyl)-2-methylbenzamide (9f)**

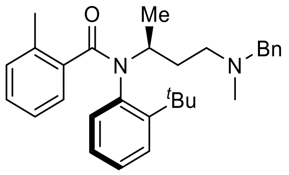

To a stirred solution of N^1-benzyl-N^1-methyl-N^3-phenylbutane-1,3-diamine **7a** (100 mg, 0.308 mmol) in acetonitrile (1.5 mL) was added sodium bicarbonate (104 mg, 1.23 mmol) and 2-methylbenzoyl chloride (0.080 mL, 96 mg, 0.621 mmol), and the reaction was heated to 80 °C for 1 h. The reaction was quenched with sat. aq. $NaHCO_3$ (15 mL) and the aqueous layer was extracted with dichloromethane (3 x 15 mL). The combined organic layers were dried over $MgSO_4$, filtered and concentrated *in vacuo*. At this point, a ^{1}H NMR spectrum of the crude product was taken to determine the *dr* (>95:5) of the reaction before chromatography. Purification by flash column chromatography (SiO_2, 7:3 hexane:ethyl acetate) gave the title compound (94 mg, 68%) as a 3:2 mixture of rotamers and as a colourless oil. R_f 0.69 (ethyl acetate); δ_H (400 MHz, $CDCl_3$) 7.61 (1H, dd, *J* = 8.0, 1.5 Hz, ArH, major rotamer), 7.42–6.94 (24H, m, ArH), 6.83–6.77 (1H, m, ArH, minor rotamer), 4.46–4.28 (2H, m, NCH, both rotamers), 3.54–3.46 (2H, m, PhC**H**H′, both rotamers), 3.38 (1H, d, *J* = 13.0 Hz, PhCH**H′**, minor rotamer), 3.23 (1H, d, *J* = 13.0 Hz, PhCH**H′**, major rotamer), 2.50 (3H, s, $ArCH_3$, minor rotamer), 2.48–2.41 (4H, m, $ArCH_3$, major rotamer, NC**H**H′, minor rotamer), 2.29–2.19 (1H, m, NC**H**H′, major rotamer), 2.16–2.07 (7H, m, NCH_3, both rotamers, NCH**H′**, major rotamer), 2.00–1.85 (2H, m, NCH**H′**, minor rotamer, CHC**H**H′, major rotamer), 1.80–1.70 (1H, m, CHC**H**H′, minor rotamer), 1.60 (3H, d, *J* = 6.0 Hz, CHC**H**$_3$, minor rotamer), 1.55 (9H, s, $C(CH_3)_3$, major rotamer), 1.33–1.26 (10H, m, $C(CH_3)_3$, minor rotamer, CHCH**H′**, minor rotamer), 0.99 (3H, d, *J* = 6.5 Hz, CHC**H**$_3$, major rotamer), 0.92–0.82 (1H, m, CHCH**H′**, major rotamer); δ_C (101 MHz, $CDCl_3$) 172.3 (CO, major rotamer), 170.2 (CO, major rotamer), 147.8 (ArC), 146.9 (ArC), 139.1 (ArC), 139.0 (ArC), 137.6 (ArC), 137.1 (ArC), 136.7 (ArC), 136.6 (ArC), 136.5 (ArC), 136.0 (ArC), 131.9 (ArC), 131.3 (ArC), 131.0 (2 x ArC), 130.9 (ArC), 130.7 (ArC), 129.1 (ArC), 128.9 (ArC), 128.8 (ArC), 128.4 (ArC), 128.3 (2 x ArC), 128.0 (ArC), 127.9 (2 x ArC), 127.1 (ArC), 127.0 (ArC), 126.7 (ArC), 125.8 (ArC), 125.4 (ArC), 125.0 (ArC), 124.2 (ArC), 62.6 ($PhCH_2$, minor rotamer), 62.4 ($PhCH_2$, major rotamer), 54.9 (NCH_2, minor rotamer), 53.9 (NCH, both rotamers), 53.7 (NCH_2, major rotamer), 42.4 (NCH_3, major rotamer), 42.3 (NCH_3, minor rotamer), 37.2 (**C**$(CH_3)_3$, major rotamer), 36.5 (**C**$(CH_3)_3$, minor rotamer), 32.8 (C(**C**H$_3$)$_3$, major rotamer), 32.7 (C(**C**H$_3$)$_3$, minor rotamer), 32.6 (CH**C**H$_2$, major rotamer), 32.1 (CH**C**H$_2$, minor rotamer), 20.7 (CH**C**H$_3$, major rotamer), 20.6 ($ArCH_3$, minor rotamer), 20.0 ($ArCH_3$, major rotamer), 19.6 (CH**C**H$_3$, minor rotamer); ν_{max}/cm^{-1} (thin film) 2964, 1639, 1489, 1367, 1346, 760, 738; HRMS (ESI); calcd. for $C_{30}H_{39}N_2O^+$, 443.3057. Found: $[MH]^+$, 443.3059 (–0.6 error ppm).

2-(*tert*-Butyl)-*N*-(hexan-2-yl)aniline (15)

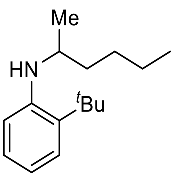

To a stirred solution of 2-hexanone (0.120 mL, 100 mg, 1.00 mmol) in 1,2-dichloroethane (1.8 mL) was added 2-*tert*-butylaniline (0.170 mL, 164 mg, 1.10 mmol), acetic acid (0.060 mL, 66 mg, 1.10 mmol) and sodium triacetoxyborohydride (318 mg, 1.50 mmol) and the reaction was heated to 80 °C for 16 h. The reaction was diluted with dichloromethane (10 mL) and sat. aq.

$NaHCO_3$ (5 mL) and the aqueous layer was extracted with dichloromethane (2 x 10 mL). The combined organic layers were washed with brine (15 mL) then dried over $MgSO_4$, filtered and concentrated *in vacuo*. Purification by flash column chromatography (SiO_2, 7:3 hexane:dichloromethane) afforded the title compound (89 mg, 38%) as a pale-yellow oil. R_f 0.41 (3:1 hexane:dichloromethane); δ_H (400 MHz, $CDCl_3$) 7.27–7.25 (1H, m, ArH), 7.15–7.11 (1H, m, ArH), 6.70–6.64 (2H, m, ArH), 3.78 (1H, d, *J* = 7.5 Hz, NH), 3.63–3.56 (1H, m, NCH), 1.72–1.64 (1H, m, CHC**H**H'), 1.60–1.49 (1H, m, CHCH**H'**), 1.45 (9H, s, $C(CH_3)_3$), 1.43–1.36 (4H, m, 2 x CH_2), 1.24 (3H, d, *J* = 6.5 Hz, CHC**H**$_3$), 0.94 (3H, t, *J* = 7.0 Hz, CH_2C**H**$_3$); δ_C (101 MHz, $CDCl_3$) 145.6 (ArC), 132.8 (ArC), 127.2 (ArC), 126.5 (ArC), 116.2 (ArC), 111.8 (ArC), 48.4 (NCH), 37.2 (CH**C**H_2), 34.2 (**C**$(CH_3)_3$), 30.1 (C(**C**$H_3)_3$), 28.6 (CH_2), 22.9 (CH_2), 20.8 (CH**C**H_3), 14.3 (CH_2**C**H_3); ν_{max}/cm^{-1} (thin film) 2958, 2929, 1601, 1507, 1447, 1309, 741; HRMS (ESI); calcd. for $C_{16}H_{28}N^+$, 234.2216. Found: $[MH]^+$, 234.2222 (–1.4 error ppm).

***N*-(2-(*tert*-Butyl)phenyl)-*N*-(hexan-2-yl)propionamide (16)**

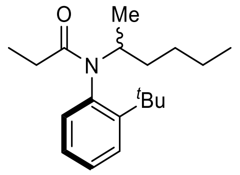

To a stirred solution of 2-(*tert*-butyl)-*N*-(hexan-2-yl)aniline **15** (100 mg, 0.43 mmol) in acetonitrile (2.2 mL) was added sodium bicarbonate (144 mg, 1.71 mmol) and propionyl chloride (0.080 mL, 80 mg, 0.86 mmol), and the reaction was heated to 80 °C for 1 h. The reaction was quenched with sat. aq. $NaHCO_3$ (15 mL) and the aqueous layer was extracted with dichloromethane (3 x 15 mL). The combined organic layers were dried over $MgSO_4$, filtered and concentrated *in vacuo*. Purification by flash column chromatography (SiO_2, 9:1 hexane:ethyl acetate) gave the title compound (87 mg, 70%) as a 1:1 mixture of diastereomers and as a yellow oil. R_f 0.15 (9:1 hexane:ethyl acetate); δ_H (400 MHz, $CDCl_3$) 7.58–7.53 (2H, m, ArH, both diastereoisomers), 7.35–7.28 (2H, m, ArH, both diastereoisomers), 7.20–7.14 (2H, m, ArH, both diastereoisomers), 6.98–6.92 (2H, m, ArH, both diastereoisomers), 4.36–4.08 (2H, m, NCH, both diastereoisomers), 2.06–1.82 (5H, m, $COCH_2$, both diastereoisomers, CHC**H**H', diastereoisomer A or B), 1.57–1.13 (32H, m, CHC**H**H', diastereoisomer A or B, CHC**H**$_3$, 5 x CH_2, $C(CH_3)_3$, both diastereoisomers), 1.05–1.98 (6H, m, $COCH_2C$**H**$_3$, both isomers), 0.92 (3H, t, *J* = 7.0 Hz, CH_2C**H**$_3$, diastereoisomer A), 0.86 (3H, d, *J* = 7.0 Hz, CHC**H**$_3$), 0.82 (3H, t, *J* = 7.0 Hz, CH_2C**H**$_3$, diastereoisomer B); δ_C (101 MHz, $CDCl_3$) 173.9 (2 x CO), 147.2 (2 x ArC), 138.0 (ArC), 137.5 (ArC), 131.5 (ArC), 131.3 (ArC), 131.0 (ArC), 130.9 (ArC), 128.3 (ArC), 128.2 (ArC), 126.7 (ArC), 126.6 (ArC), 54.0 (NCH), 53.7 (NCH), 36.7 (**C**$(CH_3)_3$, both diastereoisomers, CH_2), 33.8 (CH_2), 32.8 (C(**C**$H_3)_3$, both diastereoisomers), 29.9 (CH_2), 29.8 (CH_2), 29.5 (CH_2), 29.2 (CH_2), 22.8 (CH_2), 19.4 (CH**C**H_3), 17.0 (CH**C**H_3), 14.3 (CH_2**C**H_3), 14.2 (CH_2**C**H_3), 9.4 ($COCH_2$**C**H_3), 9.3 ($COCH_2$**C**H_3); ν_{max}/cm^{-1} (thin film) 2959, 2872, 1654, 1488, 1385, 1274, 760; HRMS (ESI); calcd. for $C_{19}H_{32}NO^+$, 290.2478. Found: $[MH]^+$, 290.2483 (–1.9 error ppm).

_N_,_N_,2-Trimethylpyridin-4-amine (S12)

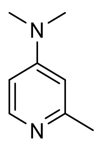

To a solution of 4-bromo-2-methylpyridine (2.60 mL, 3.82 g, 22.2 mmol) in ethanol (33 mL) was added dimethylamine (100 mL, 2 M in methanol, 200 mmol) and the solution was heated to 75 °C for 7 days. The reaction mixture was concentrated *in vacuo* before adding water (150 mL) and extracting with DCM (3 x 150 mL). The combined organic layers were dried over $MgSO_4$, filtered and concentrated *in vacuo*. The crude product was then purified by column chromatography (SiO_2, 94:5:1 ethyl acetate:methanol:triethylamine) to yield the title compound (2.16 g, 71%) as a yellow oil. R_f 0.27 (SiO_2, 94:5:1 ethyl acetate:methanol:triethylamine); δ_H (400 MHz, $CDCl_3$) 8.12 (1H, d, *J* = 6.0 Hz, ArH), 6.38–6.31 (2H, m, ArH), 2.98 (6H, s, NCH_3), 2.45 (3H, s, $ArCH_3$). Characterisation data matched those reported in the literature.[7]

2-(2-((2-(_tert_-Butyl)phenyl)amino)-2-phenylethyl)-_N_,_N_-dimethylpyridin-4-amine (4h)

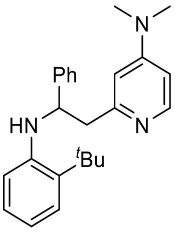

Diisopropylamine (2.00 mL, 1.48 g, 14.6 mmol) was dissolved in dry THF (30 mL) and cooled to –78 °C. *n*-BuLi (5.80 mL, 2.5 M in hexanes, 14.6 mmol) was added slowly and the resulting solution stirred for 15 minutes. To this was added *N*,*N*,2-trimethylpyridin-4-amine **S12** (1.99 g, 14.6 mmol), dissolved in THF (5 mL), dropwise and the resulting solution was stirred for 15 minutes. *N*-(2-(*tert*-butyl)phenyl)-1-phenylmethanimine **S1** (1.73 g, 7.30 mmol) was dissolved in dry THF (7 mL) and added dropwise to the reaction mixture. The reaction mixture was warmed to room temperature and stirred for 1 h. The reaction was quenched with sat. aq. NH_4Cl (50 mL) and extracted with ethyl acetate (3 x 50 mL). The combined organic layers were dried over $MgSO_4$, filtered and concentrated *in vacuo*. The crude product was then purified by column chromatography (SiO_2, 50:49:1 ethyl acetate:hexane:triethylamine) to yield the title compound (2.44 g, 89%) as an off-white solid. R_f 0.30 (50:49:1 ethyl acetate:hexane:triethylamine); m.p. 38–43 °C; δ_H (400 MHz, $CDCl_3$) 8.21 (1H, d, *J* = 6.0 Hz, ArH), 7.41–7.36 (2H, m, ArH), 7.34–7.29 (2H, m, ArH), 7.26–7.20 (2H, m, ArH), 6.93–6.87 (1H, m, ArH), 6.60 (1H, td, *J* = 7.5, 1.5 Hz, ArH), 6.39 (1H, dd, *J* = 6.0, 2.5 Hz, ArH), 6.35 (1H, dd, *J* = 8.5, 1.5 Hz, ArH), 6.14 (1H, d, *J* = 2.5 Hz, ArH), 5.60 (1H, d, *J* = 4.0 Hz, NH), 4.86 (1H, ddd, *J* = 8.0, 4.0, 4.0 Hz, NCH), 3.35 (1H, dd, *J* = 13.5, 4.0 Hz, C**H**H'), 3.09 (1H, dd, *J* = 13.5, 8.0 Hz, CH**H'**), 2.91 (6H, s, NCH_3), 1.52 (9H, s, $C(CH_3)_3$); δ_C (101 MHz, $CDCl_3$) 158.1 (ArC), 154.8 (ArC), 149.3 (ArC), 145.0 (ArC), 144.1 (ArC), 133.3 (ArC), 128.6 (ArC), 126.8 (ArC), 126.5 (2 x ArC), 126.0 (ArC), 116.2 (ArC), 112.7 (ArC), 106.5 (ArC), 105.2 (ArC), 58.8 (NCH), 47.7 (CH_2), 39.1 (NCH_3), 34.4 (**C**$(CH_3)_3$), 29.8 (C(**C**$H_3)_3$); ν_{max}/cm^{-1} (thin film) 3380, 2953, 1599, 1576, 1548, 1509, 1446,

1375, 1276, 1224, 1056, 995, 909, 740, 700; HRMS (ESI); calcd. for $C_{25}H_{32}N_3^+$, 374.2591. Found: $[MH]^+$, 374.2592 (–0.3 error ppm).

_N_-(2-(_tert_-Butyl)phenyl)-_N_-(2-(4-(dimethylamino)pyridin-2-yl)-1-phenylethyl)benzamide (6zm)

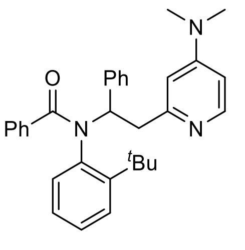

To a mixture of 2-(2-((2-(*tert*-butyl)phenyl)amino)-2-phenylethyl)-*N*,*N*-dimethylpyridin-4-amine **4h** (226 mg, 0.605 mmol) and sodium bicarbonate (203 mg, 2.42 mmol) in dry acetonitrile (3 mL) was added benzoyl chloride (0.140 mL, 170 mg, 1.21 mmol) and the resulting mixture was heated to 80 °C for 24 h. The reaction was quenched with sat. aq. $NaHCO_3$ (15 mL) and extracted with DCM (3 x 15 mL). The combined organic layers were dried over $MgSO_4$, filtered and concentrated *in vacuo*. No evidence of product formation was observed by TLC or ^{1}H NMR.

_N_-(2-(_tert_-Butyl)phenyl)-_N_-(phenyl(pyridin-2-yl)methyl)piperidine-4-carboxamide (18)

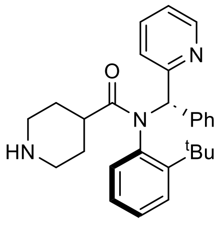

To a solution of benzyl 4-((2-(*tert*-butyl)phenyl)(phenyl(pyridin-2-yl)methyl)carbamoyl)piperidine-1-carboxylate **6x** (82 mg, 0.146 mmol) in dry degassed methanol (1.0 mL) was added 10% palladium on carbon (8 mg). The reaction mixture was stirred under a hydrogen atmosphere at room temperature for 18 h. The suspension was filtered through Celite and the filtrate concentrated *in vacuo* to yield the title compound (42 mg, 67%) as a white solid. m.p. 99–103 °C; δ_H (400 MHz, $CDCl_3$) 9.19 (1H, br s, NH), 8.50–8.44 (1H, m, ArH), 7.94–7.86 (1H, m, ArH), 7.51–7.40 (4H, m, ArH), 7.37–7.17 (5H, m, ArH), 7.07 (1H, dd, *J* = 7.5, 5.0 Hz, ArH), 6.78 (1H, d, *J* = 8.0 Hz, ArH), 6.12 (1H, s, NCH), 3.65–3.51 (1H, m, NC**H**H'), 3.19–3.08 (1H, m, NC**H**H'), 2.89–2.72 (2H, m, 2 x NCH**H'**), 2.50–2.37 (1H, m, CHCO), 2.12–1.75 (4H, m, CHH'), 1.06 (9H, s, $C(CH_3)_3$); δ_C (101 MHz, $CDCl_3$) 174.3 (CO), 158.7 (ArC), 148.5 (ArC), 146.9 (ArC), 140.3 (ArC), 139.3 (ArC), 136.1 (ArC), 131.5 (ArC), 130.6 (ArC), 129.9 (ArC), 128.7 (ArC), 128.6 (ArC), 127.9 (ArC), 127.2 (ArC), 124.5 (ArC), 122.2 (ArC), 72.9 (NCH), 41.9 (NCH_2), 41.8 (NCH_2), 36.4 (**C**HCO), 36.3 (**C**$(CH_3)_3$), 32.6 (C(**C**$H_3)_3$), 24.6 (CH_2), 24.0 (CH_2); ν_{max}/cm^{-1} (thin film) 3401, 2958, 2803, 2494, 1651, 1588, 1488, 1469, 1434, 1389, 1230, 1093, 891, 760, 730, 701; HRMS (ESI); calcd. for $C_{28}H_{34}N_3O^+$, 428.2696. Found: $[MH]^+$, 428.2698 (–0.3 error ppm).

N-(2-(tert-Butyl)phenyl)-N-(phenyl(pyridin-2-yl)methyl)-1-tosylpiperidine-4-carboxamide (19)

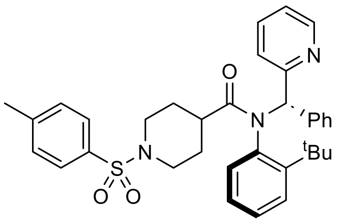

To a solution of *N*-(2-(*tert*-butyl)phenyl)-*N*-(phenyl(pyridin-2-yl)methyl)piperidine-4-carboxamide **18** (42 mg, 0.0982 mmol) in DCM (1.0 mL) was added triethylamine (0.040 mL, 30 mg, 0.294 mmol) followed by *p*-toluenesulfonyl chloride (37 mg, 0.196 mmol) and the resulting mixture was stirred at room temperature for 18 h. The reaction mixture was concentrated *in vacuo* and purified by column chromatography (SiO_2, 8:2 hexane:ethyl acetate → 7:3 hexane:ethyl acetate) to yield the title compound (56 mg, 98%) as a white solid. R_f 0.33 (7:3 hexane:ethyl acetate); m.p. 114–118 °C; δ_H (400 MHz, $CDCl_3$) 8.50–8.45 (1H, m, ArH), 7.76 (1H, dd, *J* = 8.0, 1.5 Hz, ArH), 7.57 (2H, d, *J* = 8.0 Hz, ArH), 7.46–7.12 (12H, m, ArH, 7.06 (1H, dd, *J* = 7.5, 4.5 Hz, ArH), 6.78 (1H, d, *J* = 8.0 Hz, ArH), 6.33 (1H, s, NCH), 3.79–3.73 (1H, m, NC**H**H'), 3.66–3.59 (1H, m, NC**H**H'), 2.40 (3H, s, $ArCH_3$), 2.17–2.05 (3H, m, NCH**H'**, CHCO, C**H**H'), 2.01–1.89 (2H, m, NCH**H'**, C**H**H'), 1.77–1.64 (2H, m, 2 x CH**H'**), 1.04 (9H, s, $C(CH_3)_3$); δ_C (101 MHz, $CDCl_3$) 174.7 (CO), 158.8 (ArC), 148.5 (ArC), 147.3 (ArC), 143.5 (ArC), 139.8 (ArC), 139.7 (ArC), 135.9 (ArC), 133.8 (ArC), 132.0 (ArC), 130.5 (ArC), 129.7 (ArC), 129.4 (ArC), 128.6 (ArC), 128.3 (ArC), 127.7 (ArC), 127.6 (ArC), 126.8 (ArC), 125.2 (ArC), 122.1 (ArC), 71.7 (NCH), 45.5 (NCH_2), 45.0 (NCH_2), 40.2 (**C**HCO), 36.4 (**C**$(CH_3)_3$), 32.6 (C(**C**$H_3)_3$), 28.3 (CH_2), 27.0 (CH_2), 21.6 ($ArCH_3$); ν_{max}/cm^{-1} (thin film) 2959, 1651, 1588, 1488, 1434, 1340, 1222, 1162, 1053, 931, 909, 723, 701; HRMS (ESI); calcd. for $C_{35}H_{40}N_3O_3S^+$, 582.2785. Found: $[MH]^+$, 582.2784 (0.2 error ppm).

2-(2-(N-(2-Iodo-4,6-dimethylphenyl)benzamido)-2-phenylethyl)pyridine 1-oxide (20)

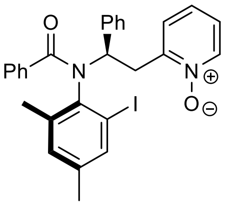

N-(2-Iodo-4,6-dimethylphenyl)-*N*-(1-phenyl-2-(pyridin-2-yl)ethyl)benzamide **6zg** (20 mg, 0.0380 mmol) was dissolved in DCM (0.38 mL) in a microwave vial. The solution was cooled to 0 °C and *m*-chloroperbenzoic acid was added (77% w/w, 12.6 mg, 0.0560 mmol). The reaction was warmed to room temperature and stirred for 4 h. The reaction mixture was concentrated and diluted with ethyl acetate and sat. aq. $NaHCO_3$. The layers were separated, and the aqueous layer was extracted twice with ethyl acetate. The combined organic layers were dried over $MgSO_4$, filtered and concentrated *in vacuo*. The crude product was purified by column chromatography (SiO_2, DCM:MeOH 96:4) to yield the title compound (12.4 mg, 60 %, >95:5 d.r.) as a white gum. R_f 0.27 (96:4 DCM:MeOH); δ_H (400 MHz, $CDCl_3$) 8.22 (1H, d, *J* = 6.5 Hz,

ArH), 7.48 (2H, dd, J = 7.0, 3.0 Hz, ArH), 7.43–7.36 (2H, m, ArH), 7.24–7.17 (6H, m, ArH), 7.15–7.02 (4H, m, ArH), 6.98 (1H, d, J = 2.0 Hz, ArH), 5.20 (1H dd, J = 10.0, 6.0 Hz, CH), 4.53 (1H, dd, J = 13.5, 10.0 Hz, C**H**H'), 4.34 (1H, dd, J = 13.5, 6.0 Hz, CH**H'**), 2.58 (3H, s, CH_3), 2.13 (3H, s, CH_3); δ_C (101 MHz, $CDCl_3$) 171.1 (CO), 149.5 (ArC), 142.3 (ArC), 139.6 (ArC), 139.3 (ArC), 139.0 (ArC), 138.0 (ArC), 137.9 (ArC), 137.3 (ArC), 132.8 (ArC), 130.6 (ArC), 130.1 (ArC), 128.7 (ArC), 128.6 (ArC), 128.3 (ArC), 128.3 (ArC), 127.3 (ArC), 124.2 (ArC), 102.6 (ArC), 64.7 (CH), 35.3 (CH_2), 20.4 (CH_3), 20.0 (CH_3). N.B. one aromatic carbon peak was not observed, presumably due to overlapping signals; ν_{max}/cm^{-1} (thin film) 3061, 2924, 2854, 1644, 1489, 1445, 1245, 729, 700. HRMS (ESI); calcd. for $C_{28}H_{26}IN_2O_2^+$, 549.1034. Found: $[MH]^+$, 549.1028 (–1.1 error ppm).

4-Amino-*N*-(2-(*tert*-butyl)phenyl)-*N*-(1-phenyl-2-(pyridin-2-yl)ethyl)benzamide (21)

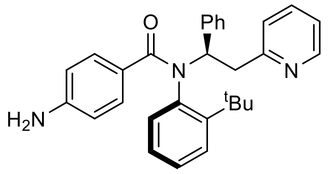

To a solution of *N*-(2-(*tert*-butyl)phenyl)-4-nitro-*N*-(1-phenyl-2-(pyridin-2-yl)ethyl)benzamide **6i** (67 mg, 0.140 mmol) in ethanol (0.4 mL) and water (0.4 mL) was added ammonium chloride (37 mg, 0.700 mmol) and iron powder (39 mg, 0.700 mmol) and the mixture was stirred at room temperature for 24 h. The solids were filtered off and washed with DCM and MeOH and the filtrate was concentrated *in vacuo*. The crude product was purified by column chromatography (SiO_2, 1:1 hexane:ethyl acetate) to yield the title compound (42 mg, 67%) as an orange solid. R_f 0.23 (1:1 hexane:ethyl acetate). m.p. 61–65 °C; δ_H (400 MHz, $CDCl_3$) 8.54–8.45 (1H, m, ArH), 7.60–6.91 (15H, m, ArH), 6.29 (1H, d, J = 8.5 Hz, ArH), 5.98–5.87 (1H, m, CH), 4.32 (2H, br s, NH_2), 3.58–3.46 (1H, m, C**H**H'), 3.25–3.12 (1H, m, CH**H'**), 1.07 (9H, s, $C(CH_3)_3$); δ_C (101 MHz, $CDCl_3$) 169.7 (CO), 158.5 (ArC), 149.2 (ArC), 147.8 (ArC), 140.8 (ArC), 138.9 (ArC), 136.3 (ArC), 132.4 (ArC), 131.8 (ArC), 131.5 (ArC), 130.9 (ArC), 129.0 (ArC), 128.1 (ArC), 127.9 (ArC), 127.3 (ArC), 126.1 (ArC), 124.3 (ArC), 121.6 (ArC), 113.3 (ArC), 111.1 (ArC), 64.7 (CH), 42.0 (CH_2), 36.5 (**C**(CH_3)$_3$), 32.4 (C(**C**H_3)$_3$); ν_{max}/cm^{-1} (thin film) 3343, 2959, 1692, 1598, 1435, 1358, 1303, 1239, 1183, 1145, 909, 764, 729, 699; HRMS (ESI); calcd. for $C_{30}H_{32}N_3O^+$, 450.2540. Found: $[MH]^+$, 450.2538 (0.4 error ppm).

***N*-(2-(*tert*-Butyl)phenyl)-4-(4-methoxybenzamido)-*N*-(1-phenyl-2-(pyridin-2-yl)ethyl)benzamide (22)**

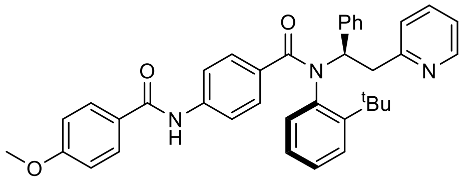

To a solution of 4-amino-*N*-(2-(*tert*-butyl)phenyl)-*N*-(1-phenyl-2-(pyridin-2-yl)ethyl)benzamide **21** (40 mg, 0.0890 mmol) in DCM (0.90 mL) was added triethylamine (0.030 mL, 18 mg, 0.178 mmol) followed by 4-methoxybenzoyl chloride (18 mg, 0.107 mmol) and the mixture was stirred at room temperature for 18 h. The reaction was quenched with

sat. aq. $NaHCO_3$ (5 mL) and extracted with DCM (3 x 10 mL). The combined organic layers were dried over $MgSO_4$, filtered and concentrated *in vacuo*. The crude product was purified by column chromatography (SiO_2, 1:1 ethyl acetate:hexane → 7:3 ethyl acetate:hexane) to yield the title compound (16 mg, 31%) as a yellow oil. R_f 0.30 (1:1 hexane:ethyl acetate); δ_H (400 MHz, $CDCl_3$) 8.56–8.50 (1H, m, ArH), 7.79–7.73 (2H, m, ArH), 7.60–7.54 (2H, m, ArH), 7.44–7.34 (5H, m, ArH), 7.29–7.17 (8H, m, ArH), 7.07–7.02 (1H, m, ArH), 6.96–6.90 (3H, m, ArH), 5.97 (1H, dd, *J* = 11.0, 5.0 Hz, CH), 3.85 (3H, s, OCH_3), 3.53 (1H, dd, *J* = 13.5, 5.0 Hz, C**H**H'), 3.14 (1H, dd, *J* = 13.5, 11.0 Hz, CH**H'**), 1.09 (9H, s, $C(CH_3)_3$); δ_C (101 MHz, $CDCl_3$) 169.2 (CO), 165.1 (CO), 162.7 (ArC), 158.5 (ArC), 149.5 (ArC), 147.9 (ArC), 140.7 (ArC), 139.4 (ArC), 138.5 (ArC), 136.2 (ArC), 132.6 (ArC), 132.4 (ArC), 131.7 (ArC), 131.1 (ArC), 129.2 (ArC), 129.0 (ArC), 128.3 (ArC), 128.2 (ArC), 127.5 (ArC), 126.9 (ArC), 126.3 (ArC), 124.3 (ArC), 121.6 (ArC), 118.1 (ArC), 114.1 (ArC), 65.0 (CH), 55.6 (OCH_3), 42.1 (CH_2), 36.5 (**C**$(CH_3)_3$), 32.6 (C(**C**$H_3)_3$); ν_{max}/cm^{-1} (thin film) 3310, 2923, 1731, 1599, 1512, 1361, 1308, 1247, 1177, 1029, 907, 763, 729, 699; HRMS (ESI); calcd. for $C_{38}H_{38}N_3O_3^+$, 584.2908. Found: $[MH]^+$, 584.2927 (–3.2 error ppm).

***N*-(2-(*tert*-Butyl)phenyl)-4'-methoxy-*N*-(1-phenyl-2-(pyridin-2-yl)ethyl)-[1,1'-biphenyl]-4-carboxamide (24)**

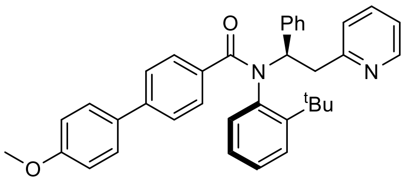

To a solution of 4-bromo-*N*-(2-(*tert*-butyl)phenyl)-*N*-(1-phenyl-2-(pyridin-2-yl)ethyl)benzamide **6f** (63 mg, 0.123 mmol) in degassed 1,2-dimethoxyethane (1.0 mL) was added caesium carbonate (0.20 mL, 2.1 M in water, 0.431 mmol), 4-methoxyphenylboronic acid **23** (28 mg, 0.185 mmol) and tetrakis(triphenylphosphine)palladium (7 mg, 6.15 µmol) and the reaction mixture was heated to 80 °C for 18 h. The reaction mixture was concentrated *in vacuo* and redissolved in ethyl acetate (10 mL). This was washed with water (10 mL) and brine (10 mL). The combined aqueous layers were extracted with ethyl acetate (2 x 10 mL). The combined organic layers were dried over $MgSO_4$, filtered and concentrated *in vacuo*. The crude product was purified by column chromatography (SiO_2, 7:3 hexane:ethyl acetate) to yield the title compound (43 mg, 65%) as a white solid. R_f 0.24 (7:3 hexane:ethyl acetate); m.p. 42–44 °C; δ_H (400 MHz, $CDCl_3$) 8.56–8.52 (1H, m, ArH), 7.63–7.58 (2H, m, ArH), 7.46–7.34 (5H, m, ArH), 7.31–7.19 (9H, m, ArH), 7.06 (1H, dd, *J* = 7.5, 5.0 Hz, ArH), 6.98 (1H, d, *J* = 8.0 Hz, ArH), 6.94–6.88 (2H, m, ArH), 6.00 (1H, dd, *J* = 11.0, 5.0 Hz, CH), 3.81 (3H, s, OCH_3), 3.58 (1H, dd, *J* = 13.5, 5.0 Hz, C**H**H'), 3.18 (1H, dd, *J* = 13.5, 11.0 Hz, CH**H'**), 1.09 (9H, s, $C(CH_3)_3$); δ_C (101 MHz, $CDCl_3$) 169.6 (CO), 159.6 (ArC), 158.5 (ArC), 149.4 (ArC), 147.9 (ArC), 141.7 (ArC), 140.6 (ArC), 138.5 (ArC), 136.2 (ArC), 135.2 (ArC), 132.5 (ArC), 132.4 (ArC), 131.6 (ArC), 130.4 (ArC), 129.2 (ArC), 128.2 (3 x ArC), 127.5 (ArC), 126.2 (ArC), 125.3 (ArC), 124.3 (ArC), 121.6 (ArC), 114.3 (ArC), 64.9 (CH), 55.4 (OCH_3), 42.0 (CH_2), 36.5 (**C**$(CH_3)_3$), 32.5 (C(**C**$H_3)_3$); ν_{max}/cm^{-1} (thin film) 2959, 1631, 1605, 1490, 1436, 1357, 1293, 1247, 1039, 909, 766, 729, 699; HRMS (ESI); calcd. for $C_{37}H_{37}N_2O_2^+$, 541.2850. Found: $[MH]^+$, 541.2831 (3.4 error ppm).

N^1-(2-(*tert*-Butyl)phenyl)-N^5-cyclopropyl-N^1-(phenyl(pyridin-2-yl)methyl)glutaramide (26)

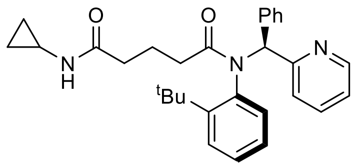

To a solution of methyl 5-((2-(*tert*-Butyl)phenyl)(phenyl(pyridin-2-yl)methyl)amino)-5-oxopentanoate **6z** (64 mg, 0.144 mmol) in THF (0.50 mL) was added lithium hydroxide (0.32 mL, 0.5 M in water, 0.158 mmol) and the mixture was stirred at room temperature for 18 h. The reaction mixture was concentrated *in vacuo* and chloroform was added and removed *in vacuo* three times to form an azeotropic mixture and remove traces of water ahead of the next step. The intermediate lithium salt was dissolved in chloroform (1.4 mL) and to this was added DIPEA (0.130 mL, 93 mg, 0.720 mmol), T3P (0.080 mL, 50% in ethyl acetate, 0.216 mmol) and cyclopropylamine (0.020 mL, 12 mg, 0.216 mmol) and the reaction mixture was stirred at room temperature for 18 h. The mixture was concentrated *in vacuo* and purified by column chromatography (SiO_2, 8:2 ethyl acetate:hexane → ethyl acetate) to yield the title compound (60 mg, 89%) as a yellow oil. R_f 0.49 (ethyl acetate); δ_H (400 MHz, $CDCl_3$) 8.50–8.44 (1H, m, ArH), 7.67 (1H, dd, *J* = 8.0, 2.0 Hz, ArH), 7.48–7.15 (9H, m, ArH), 7.07 (1H, dd, *J* = 7.5, 5.0 Hz, ArH), 6.77 (1H, d, *J* = 8.0 Hz, ArH), 6.49 (1H, br s, NH), 6.39 (1H, s, NCH), 2.67–2.59 (1H, m, C**H**CH_2), 2.22–2.02 (4H, m, 2 x $COCH_2$), 1.95–1.77 (2H, m, CH_2), 1.07 (9H, s, $C(CH_3)_3$), 0.75–0.67 (2H, m, CHC**H**$_2$), 0.47–0.39 (2H, m, CHC**H**$_2$); δ_C (101 MHz, $CDCl_3$) 174.6 (CO), 173.5 (CO), 158.9 (ArC), 148.4 (ArC), 147.4 (ArC), 139.9 (ArC), 139.6 (ArC), 136.0 (ArC), 131.9 (ArC), 130.9 (ArC), 129.3 (ArC), 128.5 (ArC), 128.4 (ArC), 127.6 (ArC), 127.0 (ArC), 125.5 (ArC), 122.2 (ArC), 71.8 (NCH), 36.4 (**C**$(CH_3)_3$), 35.6 (CO**C**H_2), 35.0 (CO**C**H_2), 32.4 (C(**C**$H_3)_3$), 22.6 (**C**HCH_2), 21.4 (CH_2), 6.6 (CH**C**H_2), 6.3 (CH**C**H_2); ν_{max}/cm^{-1} (thin film) 3297, 2961, 1644, 1589, 1488, 1434, 1389, 1248, 909, 729, 700, 614; HRMS (ESI); calcd. for $C_{30}H_{36}N_3O_2^+$, 470.2802. Found: $[MH]^+$, 470.2815 (–2.7 error ppm).

4) Amide Rotamers

In a number of examples, the isolated products had a set of minor peaks, these could be from the presence of two atropisomers or amide rotamers. To provide evidence for the presence of amide rotamers, 2D NOESY data (d_6-DMSO, 353 K, mixing time = 300 ms) for the major diastereomer of **6zi** was collected showing EXSY correlations (highlighted with black circles). This data implies exchange between the major and minor components of this mixture, supporting their assignment as geometrical isomers of the amide.

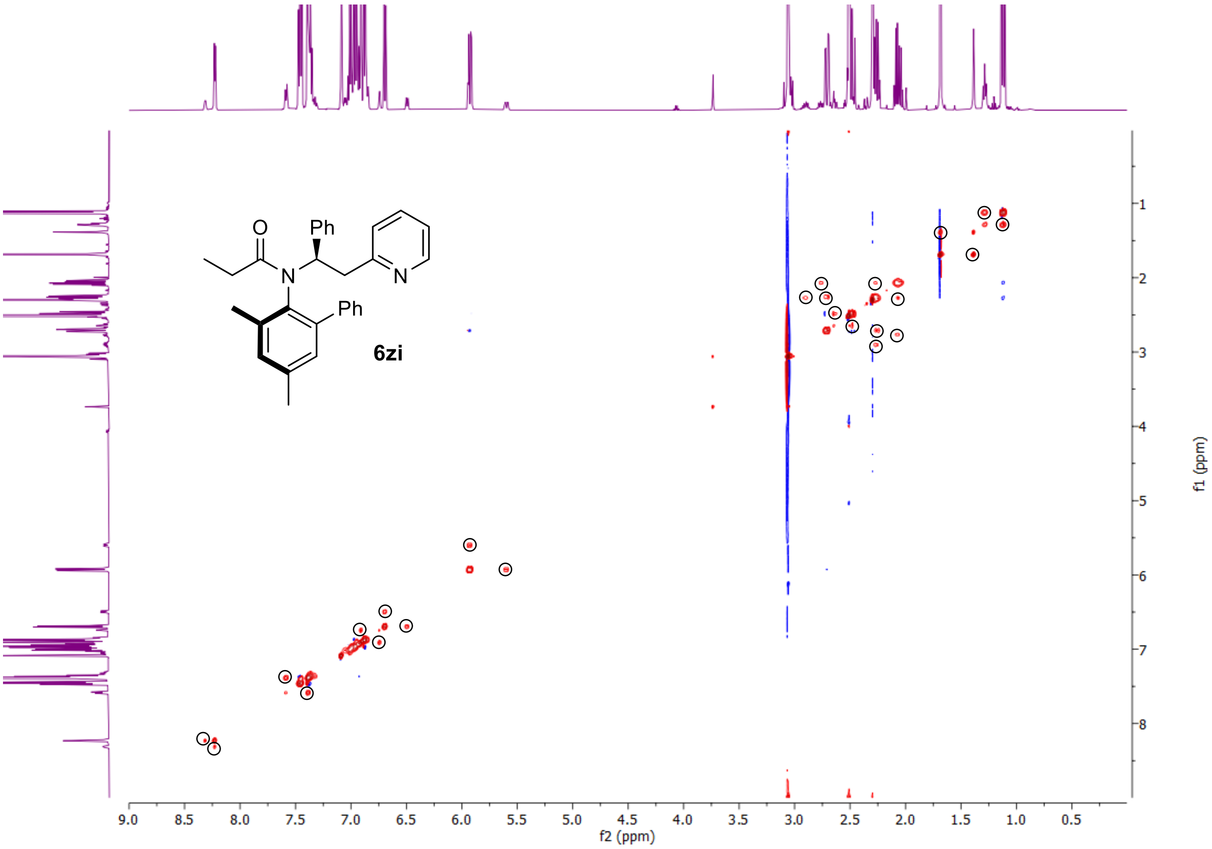

5) Thermal Isomerisation Kinetics

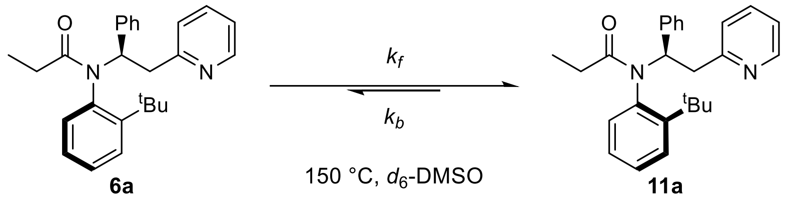

To investigate the thermal isomerisation a solution of **6a** (20 mg) in d_6-DMSO (0.65 mL) was heated to 150 °C in an oil bath in an NMR tube. At 1 h intervals the NMR tube was removed from the oil bath, cooled to room temperature and a ^{1}H NMR spectrum was collected before returning the sample to the oil bath and repeating this process (7 h in total). The *dr* of the sample was determined by ^{1}H NMR and the results are shown in Table S1. This data was used to plot the time course shown in Fig. S1.

Table S1. Thermal epimerisation of **6a** at 150 °C in d_6-DMSO.

Time / h	Fraction 6a	Fraction 11a	$\ln([6a]_t - [6a]_{eq})$
0	1	0	-0.37
1	0.515	0.485	-1.59
2	0.377	0.623	-2.72
3	0.333	0.667	-3.82
4	0.319	0.681	-4.83
5	0.314	0.686	-5.81
6	0.313	0.687	–
7	0.311	0.689	–

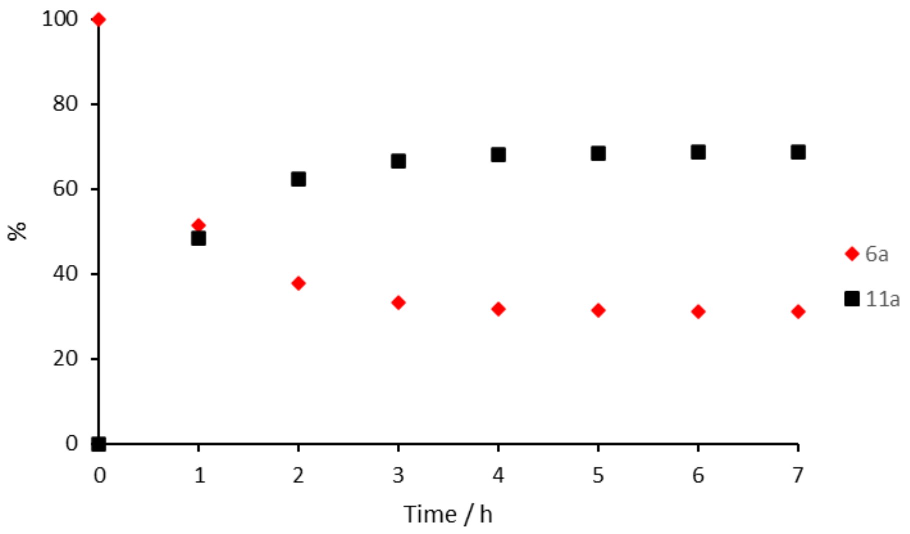

Figure S1. Thermal epimerisation plot of **6a** at 150 °C.

From these data, the rates and energy barrier for epimerization were calculated by plotting the deviation from equilibrium. i.e., ln([**6a**]$_t$ – [**6a**]$_{eq}$) against time to give a straight line with a gradient = –(k_f + k_b), where [**6a**]$_t$ and [**6a**]$_{eq}$ are the respective molar fractions of **6a** at time t and at equilibrium and k_f and k_b are the forward and backward rate constants respectively. This analysis generated the straight-line graph shown in Fig. S2:

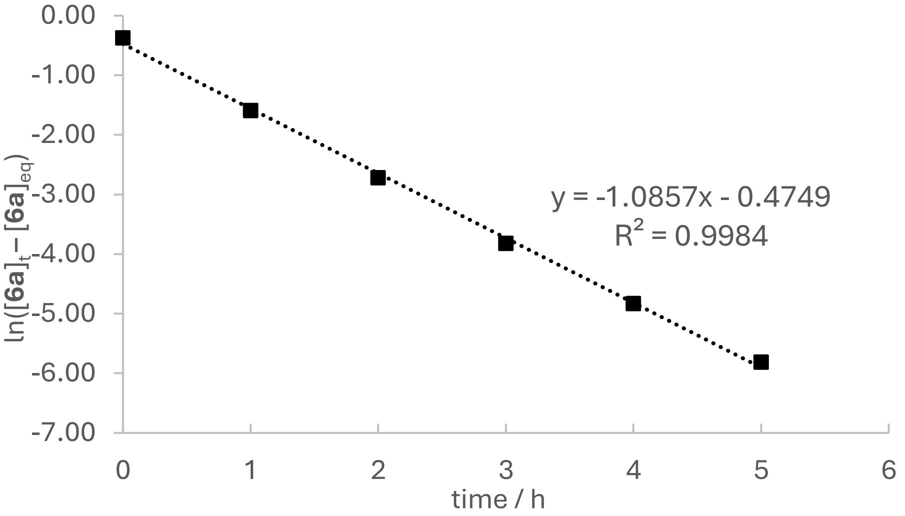

Figure S2. Plot of $ln([\boldsymbol{6a}]_t - [\boldsymbol{6a}]_{eq})$ vs t at 150 °C.

Therefore, (k_f + k_b) = 1.086 h^{-1}

Given the equilibrium ratio of **6a**:**11a** of 0.311:0.689 measured after 7 hours, we calculated:

$$K_{eq} = \frac{0.689}{0.311} = 2.215$$

And because $K_{eq} = \frac{k_f}{k_b}$ we were able to calculate that:

k_f = 0.748 h^{-1} and k_b = 0.338 h^{-1} (at 150 °C)

The forward and backwards energy barriers ($\Delta G_f^{\ddagger}$ and $\Delta G_b^{\ddagger}$) were then calculated by substituting k_f and k_b into the Eyring equation: $\Delta G^{\ddagger} = -RT \ln\left(\frac{kh}{k_B T}\right)$ to give the values shown in Table S2.

Table S2. Energy barriers for the forward and backward reactions and half-life of epimerisation at 150 °C.

**Temperature / K**	**$\Delta G_f^{\ddagger}$ / kJ mol^{-1}**	**$\Delta G_b^{\ddagger}$ / kJ mol^{-1}**
423.15	134.7	137.5

The half-life at room temperature was then estimated by substituting $\Delta G_f^{\ddagger}$ and $\Delta G_b^{\ddagger}$ into the Eyring equation to find k_f and k_b. These values were used to calculate the overall observed rate constant at room temperature, k_{obs} (=k_f + k_b), which was used to estimate $t_{1/2}$.

Table S3. Calculated rate constants and half-life of epimerisation at 25 °C.

**Temperature / K**	**k_f / s^{-1}**	**k_b / s^{-1}**	**k_{obs} / s^{-1}**	**$t_{1/2}$ / years**
298.15	1.564 x 10^{-11}	5.059 x 10^{-12}	2.070 x 10^{-11}	1062

6) Evidence for the Formation of an Acyl Pyridinium Intermediate

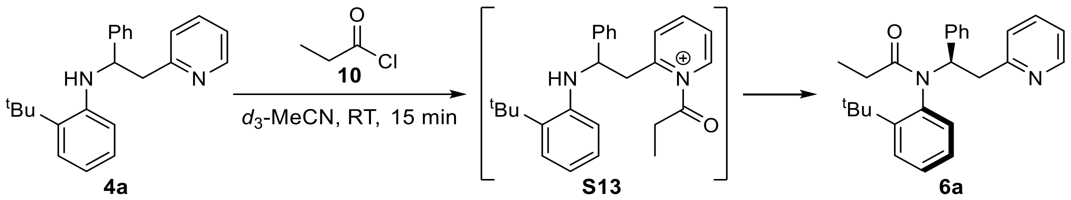

^{1}H NMR studies were attempted to observe the acyl pyridinium intermediate **S13**. In an NMR tube, aniline **4a** was reacted with propionyl chloride (4 equiv.) in d_3-MeCN at RT and after 15 minutes a ^{1}H NMR spectrum was collected. This is shown in spectrum **C** which is zoomed in on the region of the benzylic CH peak (CH highlighted in red). The spectra of starting material **4a** and product **6a**, in d_3-MeCN, are shown in spectra **A** and **B** respectively. From these spectra it can be seen that starting material **4a** is rapidly consumed at room temperature and two new species can be observed that do not correspond to the starting material **4a** or product **6a**. We propose that these species correspond to intermediate acyl pyridinium **S13** (the peak ≈5.4 ppm) as well as the acyl pyridinium form of product **6a** (the peak ≈5.7 ppm). If this is the case, these species should react with water to give a mixture of **4a** and **6a** in the same ratio. To test this, water (10 equiv.) and $NaHCO_3$ (5 equiv.) were then added to the NMR tube and a ^{1}H NMR spectrum was collected (spectrum **D**). Pleasingly, this led to the formation of starting material **4a** and product **6a** in the expected ratio, providing evidence that the species observed in spectrum **C** are as proposed (note that the absence of the NH peak of **4a** in spectrum **D** is likely due to the excess of water facilitating proton exchange). An alternative explanation considered to account for the peak ≈5.7 ppm is that this is simply protonated **4a** or 6a, but was ruled out by comparing to a sample of the HCl salts of both **4a** or **6a** (spectra not shown).

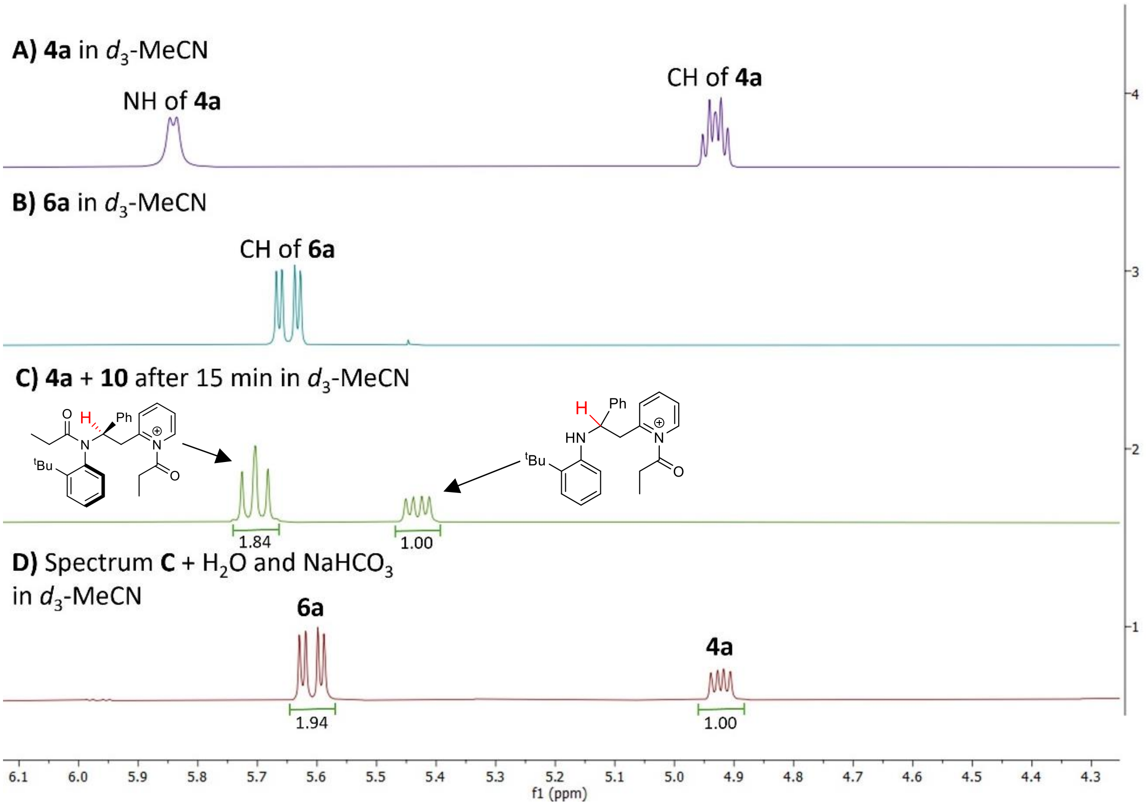

7) Computational Chemistry

The structures were loaded in PCModel,[9] and a conformational analysis was performed using the Molecular Mechanics Force Field (MMFF94) level of theory.[10–14] The structures within 3.5 kcal mol^{-1} of the lowest energy conformation were kept and the geometry of each structure was optimised using the Gaussian 16, Revision C.02 package,[15] at the B3LYP/6-31G* level of theory.[16–20] The lowest energy structure was then reoptimised with tight convergence criteria followed by frequency calculations, which confirmed the structures were minima due to the absence of imaginary frequencies.

For the methodology screening, single point calculations were carried out on the B3LYP/6-31G* optimised structures using the stated functional (B3LYP, M06-2X[21] or PBE0[22,23]) and basis set (6-31G* or def2-TZVPP[24–27]). All minima were again confirmed as such by the absence of imaginary frequencies. The SCF energies were corrected for their zero-point energies, thermal energies and entropies are at 298 K unless stated otherwise, obtained from the frequency calculations. Optimisations were performed with tight convergence criteria and no symmetry constraints were applied. An ultrafine integral grid was used for all calculations. Where used, solvent corrections were applied with the SMD model.[28] Where used, dispersion effects were modelled with Grimme's D3 method with additional Becke–Johnson damping.[29] Energies in Hartrees and xyz coordinates are reported.

Transition states were located by performing a scan of the bond length of the relevant bond being formed/broken. The highest energy structures from the scans were retained and optimised to a transition state using the Berny algorithm[30] at the B3LYP/6-31G* level of theory. This was followed by a frequency calculation to confirm there was a single imaginary frequency. Intrinsic Reaction Coordinate (IRC) analysis[31–33] confirmed that the transition states were connected to the appropriate minima.

Energies and coordinates can be found in the following pages, while the raw computational data is also available online via DOI: 10.15124/731c87bb-e34a-4170-a808-e8d8d1f2cd48

Methodology Screen

Table S5. Relative energies of the two diastereomeric products using different levels of theory at 298 K.

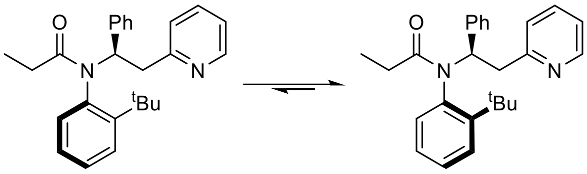

6a   11a

Entry	Functional	Basis Set	Solvent correction	Empirical dispersion correction	6a	11a
1	B3LYP	6-31G*	N	N	0	–6
2	B3LYP	6-31G*	N	D3(BJ)	0	–7
3	B3LYP	6-31G*	SMD (MeCN)	N	0	–4
4	B3LYP	6-31G*	SMD (DMSO)	N	0	–5
5	B3LYP	6-31G*	SMD (toluene)	N	0	–6
6	M06-2X	6-31G*	N	N	0	–9
7	PBE0	def2-TZVPP	N	N	0	–6

The energy difference of the two atropisomers (**6a** and **11a**) was investigated by DFT using a range of different methods (Table S5). From this screen it can be seen that all results are in close agreement with each other and so B3LYP/6-31G* was chosen for subsequent calculations.

Transition State Calculations

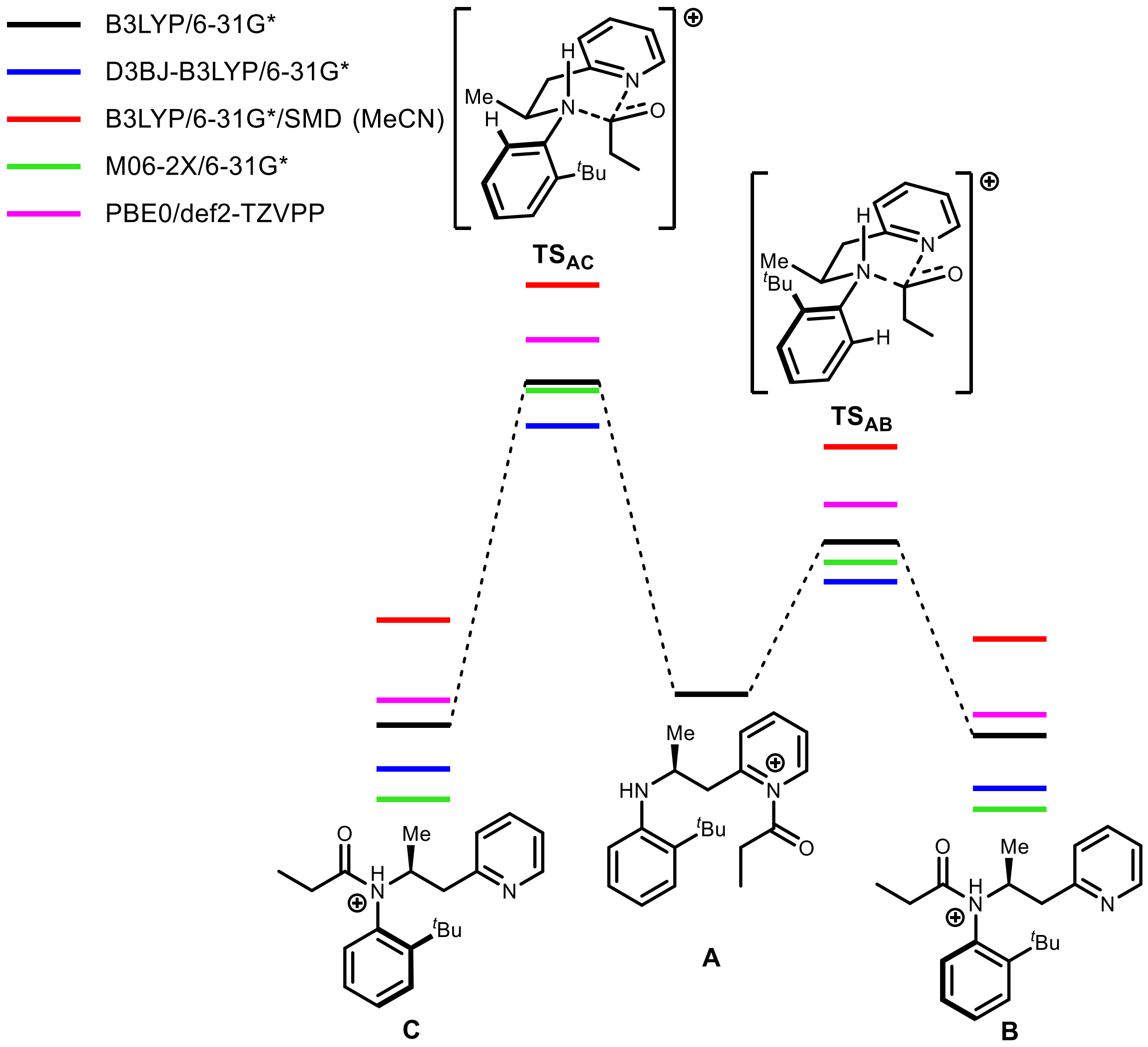

Figure S3. Relative Gibbs free energies of key intermediates and transition states at various levels of theory at 298 K.

Table S6. DFT calculated relative energies of key intermediates and transition states at various levels of theory. Energies are Gibbs free energies at 298 K in kJ mol^{-1}.

Entry	Functional	Basis Set	Solvent correction	Empirical dispersion correction	A	B	C	TS$_{AB}$	TS$_{AC}$
1	B3LYP	6-31G*	N	N	0	−12	−9	40	83
2	B3LYP	6-31G*	N	D3(BJ)	0	−24	−20	30	72
3	B3LYP	6-31G*	SMD (MeCN)	N	0	16	20	65	107
4	M06-2X	6-31G*	N	N	0	−30	−27	35	81
5	PBE0	def2-TZVPP	N	N	0	−5	−1	51	94

The DFT calculated transition states were also investigated using various methods (Figure S3 and Table S6) and although the absolute value of the numbers differ, all methods show the same trends in relative energies.

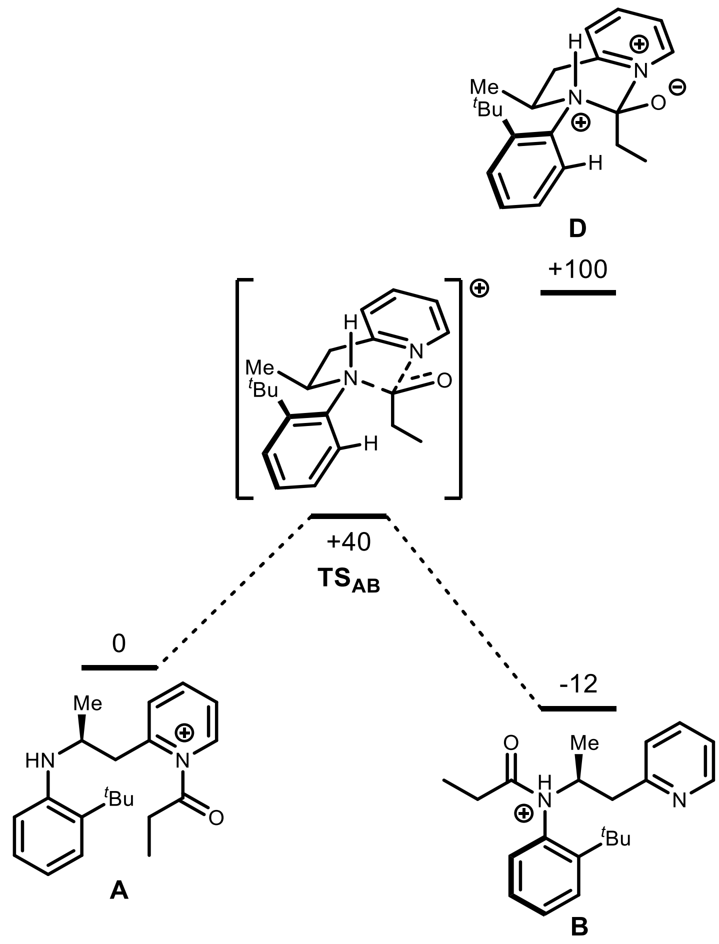

Figure S4. DFT calculated relative energies of the intermediates for an addition-elimination type mechanism vs an S_N2 like mechanism. Energies are Gibbs free energies at 298 K in kJ mol^{-1}.

An interesting note about the DFT calculated transition states is that the acyl transfer proceeds in a single step, through an S_N2 like transition state. This contrasts with the expected addition-elimination mechanism that would be expected to proceed in two steps through a tetrahedral intermediate. Initial attempts focused on an addition-elimination mechanism; however, the tetrahedral intermediate **D** could not be optimised to a minimum using an unconstrained optimisation. Intermediate **D** was optimised to a minimum by freezing the C–O bond at 1.374 Å. When this was done, it was found that intermediate **D** is 60 kJ mol^{-1} higher in energy than the previously found S_N2 like transition state (**TS$_{AB}$**) and therefore the addition-elimination mechanism through a tetrahedral intermediate was ruled out.

Energies and xyz Coordinates

6a

B3LYP/6-31G*

SCF Done: E(RB3LYP) = -1193.57372466

Zero-point correction= 0.505105
Thermal correction to Gibbs Free Energy= 0.445146
Thermal correction to Gibbs Free Energy (423.15 K)= 0.403615

C	-1.75649900	2.85528200	-1.79888200
C	-1.36826100	1.56453500	-1.43256200
C	-1.08144400	1.24628900	-0.09772300
C	-1.20160300	2.25508300	0.87179300
C	-1.58616300	3.54507100	0.50531200
C	-1.86539400	3.85118300	-0.82888100
H	-1.97548300	3.07823400	-2.84002100
H	-1.29827700	0.78947700	-2.19246500
H	-0.96854800	2.02259800	1.90511500
H	-1.66784900	4.31589500	1.26767600
H	-2.16605400	4.85760700	-1.10860800
C	-0.70391400	-0.19116900	0.24975200
H	-0.73207200	-0.75678300	-0.68352100
C	-1.75329000	-0.86989300	1.17082500
H	-1.36293600	-1.85157900	1.46619400
H	-1.89892700	-0.29019000	2.08200100
C	-3.06670700	-1.07942800	0.44860400
C	-4.24686400	-0.45811800	0.87427600
C	-5.43073000	-0.69859900	0.17932300
H	-4.22985900	0.20398700	1.73472900
C	-4.17704000	-2.11953100	-1.27804100
C	-5.40096200	-1.55044300	-0.92322400
H	-6.35867100	-0.22751300	0.49302800
H	-4.10980800	-2.78928900	-2.13463200
H	-6.29707000	-1.76914000	-1.49628000
N	-3.03566000	-1.89846900	-0.61817200
N	0.70544700	-0.38092300	0.73338200
C	1.57576100	-1.10969800	-0.16586400
C	1.31001000	-2.48897500	-0.25862800
C	2.59441200	-0.52350200	-0.95590900
C	2.00705800	-3.32107600	-1.12261100
H	0.52123700	-2.89655100	0.36684800
C	3.27378300	-1.40256400	-1.82788100

C	3.00173200	-2.76177400	-1.92243800
H	1.77502600	-4.38112000	-1.17172300
H	4.04875500	-0.99967500	-2.47069500
H	3.56551100	-3.37718800	-2.61845400
C	3.04867200	0.96326500	-1.01530400
C	2.45589500	1.91179200	0.04524900
H	2.86873900	2.91351800	-0.12489600
H	1.37178500	1.98941500	-0.01280800
H	2.72863400	1.62350400	1.06454100
C	4.58977800	1.03601300	-0.84689400
H	5.13644000	0.51039200	-1.63487100
H	4.91109700	2.08370300	-0.87114600
H	4.90130100	0.61268400	0.11528900
C	2.66103400	1.52930500	-2.40529700
H	3.08906400	0.94160300	-3.22412900
H	1.57273800	1.54215100	-2.52933500
H	3.02310300	2.55977800	-2.50527200
C	1.02781600	-0.20296100	2.06255100
C	2.36308900	-0.77625500	2.54637000
H	2.28908600	-1.87208200	2.50535500
H	3.16448300	-0.51932800	1.84659100
C	2.70768700	-0.31604400	3.96236000
H	3.64575900	-0.77529500	4.29252100
H	2.82220100	0.77173100	4.00663400
H	1.91636600	-0.58761900	4.66554200
O	0.26982500	0.36326000	2.84783300

D3(BJ)-B3LYP/6-31G*

SCF Done: E(RB3LYP) = -1193.71527127

Zero-point correction= 0.505344
Thermal correction to Gibbs Free Energy= 0.446209

C	1.75642300	2.85527500	-1.79896900
C	1.36819400	1.56453100	-1.43262900
C	1.08144500	1.24628800	-0.09777400
C	1.20166100	2.25508100	0.87173500
C	1.58621100	3.54506600	0.50523300
C	1.86537500	3.85117500	-0.82897500
H	1.97535300	3.07822500	-2.84012100
H	1.29816400	0.78947300	-2.19252800
H	0.96865600	2.02259900	1.90506800
H	1.66794200	4.31589000	1.26759200
H	2.16602900	4.85759700	-1.10871700
C	0.70392400	-0.19116900	0.24972000
H	0.73203500	-0.75678100	-0.68355500
C	1.75334000	-0.86990000	1.17074200
H	1.89901800	-0.29020300	2.08191500
H	1.36299500	-1.85158700	1.46612100
C	3.06672400	-1.07943600	0.44846100
C	4.24690300	-0.45813600	0.87408300
C	5.43073600	-0.69861700	0.17907300
H	4.22994200	0.20396000	1.73454300
C	4.17697100	-2.11953000	-1.27824600
C	5.40091200	-1.55045000	-0.92348100
H	6.35869400	-0.22753900	0.49273900
H	4.10969600	-2.78927900	-2.13483900
H	6.29699200	-1.76914700	-1.49658100
N	3.03562200	-1.89846800	-0.61832100
N	-0.70541500	-0.38091600	0.73341700
C	-1.57577300	-1.10969400	-0.16578400
C	-1.31003300	-2.48897400	-0.25854900
C	-2.59445900	-0.52350000	-0.95578500
C	-2.00712400	-3.32107800	-1.12249400
H	-0.52123400	-2.89654800	0.36689500
C	-3.27387300	-1.40256700	-1.82772000
C	-3.00183200	-2.76177800	-1.92228000
H	-1.77509900	-4.38112300	-1.17160800
H	-4.04887400	-0.99967900	-2.47050100
H	-3.56564500	-3.37719500	-2.61826500

C	-3.04871700	0.96326700	-1.01517000
C	-2.45589000	1.91180000	0.04534900
H	-2.86873400	2.91352800	-0.12478900
H	-2.72858900	1.62352400	1.06465600
H	-1.37178200	1.98941500	-0.01275200
C	-2.66113900	1.52929500	-2.40518400
H	-3.08920100	0.94158500	-3.22399400
H	-3.02321500	2.55976600	-2.50515400
H	-1.57284900	1.54214400	-2.52926700
C	-4.58981500	1.03601900	-0.84669100
H	-4.90129300	0.61270800	0.11551400
H	-4.91113500	2.08370800	-0.87094700
H	-5.13651500	0.51038300	-1.63463300
C	-1.02772200	-0.20294400	2.06260100
C	-2.36297200	-0.77623700	2.54648500
H	-3.16439900	-0.51931300	1.84674400
H	-2.28896900	-1.87206400	2.50547000
C	-2.70750100	-0.31602400	3.96249000
H	-1.91614800	-0.58760200	4.66563600
H	-2.82200600	0.77175300	4.00676900
H	-3.64556000	-0.77526800	4.29269700
O	-0.26969100	0.36327400	2.84784600

B3LYP/6-31G*/SMD (MeCN)

SCF Done: E(RB3LYP) = -1193.60063509

Zero-point correction= 0.504599
Thermal correction to Gibbs Free Energy= 0.443663

C	1.75642300	2.85527500	-1.79896900
C	1.36819400	1.56453100	-1.43262900
C	1.08144500	1.24628800	-0.09777400
C	1.20166100	2.25508100	0.87173500
C	1.58621100	3.54506600	0.50523300
C	1.86537500	3.85117500	-0.82897500
H	1.97535300	3.07822500	-2.84012100
H	1.29816400	0.78947300	-2.19252800
H	0.96865600	2.02259900	1.90506800
H	1.66794200	4.31589000	1.26759200
H	2.16602900	4.85759700	-1.10871700
C	0.70392400	-0.19116900	0.24972000
H	0.73203500	-0.75678100	-0.68355500
C	1.75334000	-0.86990000	1.17074200
H	1.89901800	-0.29020300	2.08191500
H	1.36299500	-1.85158700	1.46612100
C	3.06672400	-1.07943600	0.44846100
C	4.24690300	-0.45813600	0.87408300
C	5.43073600	-0.69861700	0.17907300
H	4.22994200	0.20396000	1.73454300
C	4.17697100	-2.11953000	-1.27824600
C	5.40091200	-1.55045000	-0.92348100
H	6.35869400	-0.22753900	0.49273900
H	4.10969600	-2.78927900	-2.13483900
H	6.29699200	-1.76914700	-1.49658100
N	3.03562200	-1.89846800	-0.61832100
N	-0.70541500	-0.38091600	0.73341700
C	-1.57577300	-1.10969400	-0.16578400
C	-1.31003300	-2.48897400	-0.25854900
C	-2.59445900	-0.52350000	-0.95578500
C	-2.00712400	-3.32107800	-1.12249400
H	-0.52123400	-2.89654800	0.36689500
C	-3.27387300	-1.40256700	-1.82772000
C	-3.00183200	-2.76177800	-1.92228000
H	-1.77509900	-4.38112300	-1.17160800
H	-4.04887400	-0.99967900	-2.47050100
H	-3.56564500	-3.37719500	-2.61826500

C	-3.04871700	0.96326700	-1.01517000
C	-2.45589000	1.91180000	0.04534900
H	-2.86873400	2.91352800	-0.12478900
H	-2.72858900	1.62352400	1.06465600
H	-1.37178200	1.98941500	-0.01275200
C	-2.66113900	1.52929500	-2.40518400
H	-3.08920100	0.94158500	-3.22399400
H	-3.02321500	2.55976600	-2.50515400
H	-1.57284900	1.54214400	-2.52926700
C	-4.58981500	1.03601900	-0.84669100
H	-4.90129300	0.61270800	0.11551400
H	-4.91113500	2.08370800	-0.87094700
H	-5.13651500	0.51038300	-1.63463300
C	-1.02772200	-0.20294400	2.06260100
C	-2.36297200	-0.77623700	2.54648500
H	-3.16439900	-0.51931300	1.84674400
H	-2.28896900	-1.87206400	2.50547000
C	-2.70750100	-0.31602400	3.96249000
H	-1.91614800	-0.58760200	4.66563600
H	-2.82200600	0.77175300	4.00676900
H	-3.64556000	-0.77526800	4.29269700
O	-0.26969100	0.36327400	2.84784600

B3LYP/6-31G*/SMD (DMSO)

SCF Done: E(RB3LYP) = -1193.59277081

Zero-point correction= 0.504666
Thermal correction to Gibbs Free Energy= 0.443672

C	1.75642300	2.85527500	-1.79896900
C	1.36819400	1.56453100	-1.43262900
C	1.08144500	1.24628800	-0.09777400
C	1.20166100	2.25508100	0.87173500
C	1.58621100	3.54506600	0.50523300
C	1.86537500	3.85117500	-0.82897500
H	1.97535300	3.07822500	-2.84012100
H	1.29816400	0.78947300	-2.19252800
H	0.96865600	2.02259900	1.90506800
H	1.66794200	4.31589000	1.26759200
H	2.16602900	4.85759700	-1.10871700
C	0.70392400	-0.19116900	0.24972000
H	0.73203500	-0.75678100	-0.68355500
C	1.75334000	-0.86990000	1.17074200
H	1.89901800	-0.29020300	2.08191500
H	1.36299500	-1.85158700	1.46612100
C	3.06672400	-1.07943600	0.44846100
C	4.24690300	-0.45813600	0.87408300
C	5.43073600	-0.69861700	0.17907300
H	4.22994200	0.20396000	1.73454300
C	4.17697100	-2.11953000	-1.27824600
C	5.40091200	-1.55045000	-0.92348100
H	6.35869400	-0.22753900	0.49273900
H	4.10969600	-2.78927900	-2.13483900
H	6.29699200	-1.76914700	-1.49658100
N	3.03562200	-1.89846800	-0.61832100
N	-0.70541500	-0.38091600	0.73341700
C	-1.57577300	-1.10969400	-0.16578400
C	-1.31003300	-2.48897400	-0.25854900
C	-2.59445900	-0.52350000	-0.95578500
C	-2.00712400	-3.32107800	-1.12249400
H	-0.52123400	-2.89654800	0.36689500
C	-3.27387300	-1.40256700	-1.82772000
C	-3.00183200	-2.76177800	-1.92228000
H	-1.77509900	-4.38112300	-1.17160800
H	-4.04887400	-0.99967900	-2.47050100
H	-3.56564500	-3.37719500	-2.61826500

C	-3.04871700	0.96326700	-1.01517000
C	-2.45589000	1.91180000	0.04534900
H	-2.86873400	2.91352800	-0.12478900
H	-2.72858900	1.62352400	1.06465600
H	-1.37178200	1.98941500	-0.01275200
C	-2.66113900	1.52929500	-2.40518400
H	-3.08920100	0.94158500	-3.22399400
H	-3.02321500	2.55976600	-2.50515400
H	-1.57284900	1.54214400	-2.52926700
C	-4.58981500	1.03601900	-0.84669100
H	-4.90129300	0.61270800	0.11551400
H	-4.91113500	2.08370800	-0.87094700
H	-5.13651500	0.51038300	-1.63463300
C	-1.02772200	-0.20294400	2.06260100
C	-2.36297200	-0.77623700	2.54648500
H	-3.16439900	-0.51931300	1.84674400
H	-2.28896900	-1.87206400	2.50547000
C	-2.70750100	-0.31602400	3.96249000
H	-1.91614800	-0.58760200	4.66563600
H	-2.82200600	0.77175300	4.00676900
H	-3.64556000	-0.77526800	4.29269700
O	-0.26969100	0.36327400	2.84784600

B3LYP/6-31G*/SMD (toluene)

SCF Done: E(RB3LYP) = -1193.59783119

Zero-point correction= 0.505166
Thermal correction to Gibbs Free Energy= 0.445279

C	1.75642300	2.85527500	-1.79896900
C	1.36819400	1.56453100	-1.43262900
C	1.08144500	1.24628800	-0.09777400
C	1.20166100	2.25508100	0.87173500
C	1.58621100	3.54506600	0.50523300
C	1.86537500	3.85117500	-0.82897500
H	1.97535300	3.07822500	-2.84012100
H	1.29816400	0.78947300	-2.19252800
H	0.96865600	2.02259900	1.90506800
H	1.66794200	4.31589000	1.26759200
H	2.16602900	4.85759700	-1.10871700
C	0.70392400	-0.19116900	0.24972000
H	0.73203500	-0.75678100	-0.68355500
C	1.75334000	-0.86990000	1.17074200
H	1.89901800	-0.29020300	2.08191500
H	1.36299500	-1.85158700	1.46612100
C	3.06672400	-1.07943600	0.44846100
C	4.24690300	-0.45813600	0.87408300
C	5.43073600	-0.69861700	0.17907300
H	4.22994200	0.20396000	1.73454300
C	4.17697100	-2.11953000	-1.27824600
C	5.40091200	-1.55045000	-0.92348100
H	6.35869400	-0.22753900	0.49273900
H	4.10969600	-2.78927900	-2.13483900
H	6.29699200	-1.76914700	-1.49658100
N	3.03562200	-1.89846800	-0.61832100
N	-0.70541500	-0.38091600	0.73341700
C	-1.57577300	-1.10969400	-0.16578400
C	-1.31003300	-2.48897400	-0.25854900
C	-2.59445900	-0.52350000	-0.95578500
C	-2.00712400	-3.32107800	-1.12249400
H	-0.52123400	-2.89654800	0.36689500
C	-3.27387300	-1.40256700	-1.82772000
C	-3.00183200	-2.76177800	-1.92228000
H	-1.77509900	-4.38112300	-1.17160800
H	-4.04887400	-0.99967900	-2.47050100
H	-3.56564500	-3.37719500	-2.61826500

C	-3.04871700	0.96326700	-1.01517000
C	-2.45589000	1.91180000	0.04534900
H	-2.86873400	2.91352800	-0.12478900
H	-2.72858900	1.62352400	1.06465600
H	-1.37178200	1.98941500	-0.01275200
C	-2.66113900	1.52929500	-2.40518400
H	-3.08920100	0.94158500	-3.22399400
H	-3.02321500	2.55976600	-2.50515400
H	-1.57284900	1.54214400	-2.52926700
C	-4.58981500	1.03601900	-0.84669100
H	-4.90129300	0.61270800	0.11551400
H	-4.91113500	2.08370800	-0.87094700
H	-5.13651500	0.51038300	-1.63463300
C	-1.02772200	-0.20294400	2.06260100
C	-2.36297200	-0.77623700	2.54648500
H	-3.16439900	-0.51931300	1.84674400
H	-2.28896900	-1.87206400	2.50547000
C	-2.70750100	-0.31602400	3.96249000
H	-1.91614800	-0.58760200	4.66563600
H	-2.82200600	0.77175300	4.00676900
H	-3.64556000	-0.77526800	4.29269700
O	-0.26969100	0.36327400	2.84784600

M06-2X/6-31G*

SCF Done: E(RM062X) = -1193.06190579

Zero-point correction= 0.508722
Thermal correction to Gibbs Free Energy= 0.452182

C	1.75642300	2.85527500	-1.79896900
C	1.36819400	1.56453100	-1.43262900
C	1.08144500	1.24628800	-0.09777400
C	1.20166100	2.25508100	0.87173500
C	1.58621100	3.54506600	0.50523300
C	1.86537500	3.85117500	-0.82897500
H	1.97535300	3.07822500	-2.84012100
H	1.29816400	0.78947300	-2.19252800
H	0.96865600	2.02259900	1.90506800
H	1.66794200	4.31589000	1.26759200
H	2.16602900	4.85759700	-1.10871700
C	0.70392400	-0.19116900	0.24972000
H	0.73203500	-0.75678100	-0.68355500
C	1.75334000	-0.86990000	1.17074200
H	1.89901800	-0.29020300	2.08191500
H	1.36299500	-1.85158700	1.46612100
C	3.06672400	-1.07943600	0.44846100
C	4.24690300	-0.45813600	0.87408300
C	5.43073600	-0.69861700	0.17907300
H	4.22994200	0.20396000	1.73454300
C	4.17697100	-2.11953000	-1.27824600
C	5.40091200	-1.55045000	-0.92348100
H	6.35869400	-0.22753900	0.49273900
H	4.10969600	-2.78927900	-2.13483900
H	6.29699200	-1.76914700	-1.49658100
N	3.03562200	-1.89846800	-0.61832100
N	-0.70541500	-0.38091600	0.73341700
C	-1.57577300	-1.10969400	-0.16578400
C	-1.31003300	-2.48897400	-0.25854900
C	-2.59445900	-0.52350000	-0.95578500
C	-2.00712400	-3.32107800	-1.12249400
H	-0.52123400	-2.89654800	0.36689500
C	-3.27387300	-1.40256700	-1.82772000
C	-3.00183200	-2.76177800	-1.92228000
H	-1.77509900	-4.38112300	-1.17160800
H	-4.04887400	-0.99967900	-2.47050100
H	-3.56564500	-3.37719500	-2.61826500

C	-3.04871700	0.96326700	-1.01517000
C	-2.45589000	1.91180000	0.04534900
H	-2.86873400	2.91352800	-0.12478900
H	-2.72858900	1.62352400	1.06465600
H	-1.37178200	1.98941500	-0.01275200
C	-2.66113900	1.52929500	-2.40518400
H	-3.08920100	0.94158500	-3.22399400
H	-3.02321500	2.55976600	-2.50515400
H	-1.57284900	1.54214400	-2.52926700
C	-4.58981500	1.03601900	-0.84669100
H	-4.90129300	0.61270800	0.11551400
H	-4.91113500	2.08370800	-0.87094700
H	-5.13651500	0.51038300	-1.63463300
C	-1.02772200	-0.20294400	2.06260100
C	-2.36297200	-0.77623700	2.54648500
H	-3.16439900	-0.51931300	1.84674400
H	-2.28896900	-1.87206400	2.50547000
C	-2.70750100	-0.31602400	3.96249000
H	-1.91614800	-0.58760200	4.66563600
H	-2.82200600	0.77175300	4.00676900
H	-3.64556000	-0.77526800	4.29269700
O	-0.26969100	0.36327400	2.84784600

PBE0/def2-TZVPP

SCF Done: E(RPBE1PBE) = -1192.58289473

Zero-point correction= 0.501908
Thermal correction to Gibbs Free Energy= 0.447815

C	1.75642300	2.85527500	-1.79896900
C	1.36819400	1.56453100	-1.43262900
C	1.08144500	1.24628800	-0.09777400
C	1.20166100	2.25508100	0.87173500
C	1.58621100	3.54506600	0.50523300
C	1.86537500	3.85117500	-0.82897500
H	1.97535300	3.07822500	-2.84012100
H	1.29816400	0.78947300	-2.19252800
H	0.96865600	2.02259900	1.90506800
H	1.66794200	4.31589000	1.26759200
H	2.16602900	4.85759700	-1.10871700
C	0.70392400	-0.19116900	0.24972000
H	0.73203500	-0.75678100	-0.68355500
C	1.75334000	-0.86990000	1.17074200
H	1.89901800	-0.29020300	2.08191500
H	1.36299500	-1.85158700	1.46612100
C	3.06672400	-1.07943600	0.44846100
C	4.24690300	-0.45813600	0.87408300
C	5.43073600	-0.69861700	0.17907300
H	4.22994200	0.20396000	1.73454300
C	4.17697100	-2.11953000	-1.27824600
C	5.40091200	-1.55045000	-0.92348100
H	6.35869400	-0.22753900	0.49273900
H	4.10969600	-2.78927900	-2.13483900
H	6.29699200	-1.76914700	-1.49658100
N	3.03562200	-1.89846800	-0.61832100
N	-0.70541500	-0.38091600	0.73341700
C	-1.57577300	-1.10969400	-0.16578400
C	-1.31003300	-2.48897400	-0.25854900
C	-2.59445900	-0.52350000	-0.95578500
C	-2.00712400	-3.32107800	-1.12249400
H	-0.52123400	-2.89654800	0.36689500
C	-3.27387300	-1.40256700	-1.82772000
C	-3.00183200	-2.76177800	-1.92228000
H	-1.77509900	-4.38112300	-1.17160800
H	-4.04887400	-0.99967900	-2.47050100
H	-3.56564500	-3.37719500	-2.61826500

C	-3.04871700	0.96326700	-1.01517000
C	-2.45589000	1.91180000	0.04534900
H	-2.86873400	2.91352800	-0.12478900
H	-2.72858900	1.62352400	1.06465600
H	-1.37178200	1.98941500	-0.01275200
C	-2.66113900	1.52929500	-2.40518400
H	-3.08920100	0.94158500	-3.22399400
H	-3.02321500	2.55976600	-2.50515400
H	-1.57284900	1.54214400	-2.52926700
C	-4.58981500	1.03601900	-0.84669100
H	-4.90129300	0.61270800	0.11551400
H	-4.91113500	2.08370800	-0.87094700
H	-5.13651500	0.51038300	-1.63463300
C	-1.02772200	-0.20294400	2.06260100
C	-2.36297200	-0.77623700	2.54648500
H	-3.16439900	-0.51931300	1.84674400
H	-2.28896900	-1.87206400	2.50547000
C	-2.70750100	-0.31602400	3.96249000
H	-1.91614800	-0.58760200	4.66563600
H	-2.82200600	0.77175300	4.00676900
H	-3.64556000	-0.77526800	4.29269700
O	-0.26969100	0.36327400	2.84784600

11a

B3LYP/6-31G*

SCF Done: E(RB3LYP) = -1193.57675889

Zero-point correction= 0.505437
Thermal correction to Gibbs Free Energy= 0.445927
Thermal correction to Gibbs Free Energy (423.15 K)= 0.404620

C	-2.44523800	-2.42224800	-2.23026600
C	-1.73312200	-1.34796400	-1.69322800
C	-1.34493100	-1.33701900	-0.34653200
C	-1.69219200	-2.43492400	0.45853000
C	-2.39812400	-3.51134500	-0.07968200
C	-2.77862600	-3.51004100	-1.42386500
H	-2.73665800	-2.40715000	-3.27741900
H	-1.47824200	-0.50232800	-2.32748500
H	-1.39614900	-2.44422300	1.50167900
H	-2.65562400	-4.35432100	0.55686200
H	-3.33165800	-4.34958300	-1.83727600
C	-0.58558600	-0.12589000	0.19475100
H	-0.48524200	0.57413200	-0.63645100
C	-1.36972700	0.63559200	1.29263100
H	-1.64063500	-0.03447700	2.10704200
H	-0.71012100	1.40445300	1.71233800
C	-2.59696200	1.31492200	0.72304300
C	-3.88429100	1.00487700	1.17784100
C	-4.97839800	1.67329000	0.63179500
H	-4.01896000	0.24903900	1.94571200
C	-3.43996200	2.87001800	-0.75178700
C	-4.75643100	2.63105300	-0.35612400
H	-5.98620000	1.44818000	0.97098800
H	-3.22183900	3.60875400	-1.52200700
H	-5.57652000	3.17798900	-0.81161500
N	-2.38321400	2.23599400	-0.23365500
N	0.83172600	-0.42581500	0.58087500
C	1.78924200	-0.38807000	-0.50665100
C	1.88776800	-1.57099200	-1.25599100
C	2.57234700	0.75021400	-0.84310200
C	2.74261900	-1.68375100	-2.34666200
H	1.26506000	-2.40993100	-0.96234500
C	3.42169300	0.59419400	-1.95623200
C	3.51515200	-0.58221900	-2.69717600
H	2.79793800	-2.61245600	-2.90753700

H	4.04473400	1.42357900	-2.26376600
H	4.19542500	-0.62880200	-3.54355600
C	2.53719500	2.13442100	-0.13325900
C	2.50346300	2.03700700	1.40957600
H	2.52414800	3.04802100	1.83301700
H	1.60917100	1.54076800	1.78341900
H	3.38111700	1.50398600	1.79355800
C	3.79352500	2.97501200	-0.47359100
H	3.82416200	3.28645900	-1.52276900
H	3.78019000	3.89060000	0.12725500
H	4.72133100	2.43836300	-0.24454300
C	1.30985100	2.93897400	-0.62909100
H	1.32177000	3.02850700	-1.72173700
H	0.35312200	2.49765900	-0.34116700
H	1.33937400	3.95306900	-0.21061400
C	1.12606800	-1.09061300	1.75647200
C	2.57333300	-1.54075800	1.97205300
H	2.71628000	-2.46759900	1.39902200
H	3.27197400	-0.82068600	1.53769100
C	2.88077300	-1.78557700	3.44996400
H	2.17877800	-2.50460300	3.87874600
H	3.89924700	-2.17129200	3.56717100
H	2.79650600	-0.85988400	4.02907100
O	0.27266400	-1.31830700	2.61195900

D3(BJ)-B3LYP/6-31G*

SCF Done: E(RB3LYP) = -1193.71858614

Zero-point correction= 0.505677
Thermal correction to Gibbs Free Energy= 0.446843

C	-2.44523800	-2.42224800	-2.23026600
C	-1.73312200	-1.34796400	-1.69322800
C	-1.34493100	-1.33701900	-0.34653200
C	-1.69219200	-2.43492400	0.45853000
C	-2.39812400	-3.51134500	-0.07968200
C	-2.77862600	-3.51004100	-1.42386500
H	-2.73665800	-2.40715000	-3.27741900
H	-1.47824200	-0.50232800	-2.32748500
H	-1.39614900	-2.44422300	1.50167900
H	-2.65562400	-4.35432100	0.55686200
H	-3.33165800	-4.34958300	-1.83727600
C	-0.58558600	-0.12589000	0.19475100
H	-0.48524200	0.57413200	-0.63645100
C	-1.36972700	0.63559200	1.29263100
H	-1.64063500	-0.03447700	2.10704200
H	-0.71012100	1.40445300	1.71233800
C	-2.59696200	1.31492200	0.72304300
C	-3.88429100	1.00487700	1.17784100
C	-4.97839800	1.67329000	0.63179500
H	-4.01896000	0.24903900	1.94571200
C	-3.43996200	2.87001800	-0.75178700
C	-4.75643100	2.63105300	-0.35612400
H	-5.98620000	1.44818000	0.97098800
H	-3.22183900	3.60875400	-1.52200700
H	-5.57652000	3.17798900	-0.81161500
N	-2.38321400	2.23599400	-0.23365500
N	0.83172600	-0.42581500	0.58087500
C	1.78924200	-0.38807000	-0.50665100
C	1.88776800	-1.57099200	-1.25599100
C	2.57234700	0.75021400	-0.84310200
C	2.74261900	-1.68375100	-2.34666200
H	1.26506000	-2.40993100	-0.96234500
C	3.42169300	0.59419400	-1.95623200
C	3.51515200	-0.58221900	-2.69717600
H	2.79793800	-2.61245600	-2.90753700
H	4.04473400	1.42357900	-2.26376600
H	4.19542500	-0.62880200	-3.54355600

C	2.53719500	2.13442100	-0.13325900
C	2.50346300	2.03700700	1.40957600
H	2.52414800	3.04802100	1.83301700
H	1.60917100	1.54076800	1.78341900
H	3.38111700	1.50398600	1.79355800
C	3.79352500	2.97501200	-0.47359100
H	3.82416200	3.28645900	-1.52276900
H	3.78019000	3.89060000	0.12725500
H	4.72133100	2.43836300	-0.24454300
C	1.30985100	2.93897400	-0.62909100
H	1.32177000	3.02850700	-1.72173700
H	0.35312200	2.49765900	-0.34116700
H	1.33937400	3.95306900	-0.21061400
C	1.12606800	-1.09061300	1.75647200
C	2.57333300	-1.54075800	1.97205300
H	2.71628000	-2.46759900	1.39902200
H	3.27197400	-0.82068600	1.53769100
C	2.88077300	-1.78557700	3.44996400
H	2.17877800	-2.50460300	3.87874600
H	3.89924700	-2.17129200	3.56717100
H	2.79650600	-0.85988400	4.02907100
O	0.27266400	-1.31830700	2.61195900

B3LYP/6-31G*/SMD (MeCN)

SCF Done: E(RB3LYP) = -1193.60285220

Zero-point correction= 0.504841
Thermal correction to Gibbs Free Energy= 0.444368

C	-2.44523800	-2.42224800	-2.23026600
C	-1.73312200	-1.34796400	-1.69322800
C	-1.34493100	-1.33701900	-0.34653200
C	-1.69219200	-2.43492400	0.45853000
C	-2.39812400	-3.51134500	-0.07968200
C	-2.77862600	-3.51004100	-1.42386500
H	-2.73665800	-2.40715000	-3.27741900
H	-1.47824200	-0.50232800	-2.32748500
H	-1.39614900	-2.44422300	1.50167900
H	-2.65562400	-4.35432100	0.55686200
H	-3.33165800	-4.34958300	-1.83727600
C	-0.58558600	-0.12589000	0.19475100
H	-0.48524200	0.57413200	-0.63645100
C	-1.36972700	0.63559200	1.29263100
H	-1.64063500	-0.03447700	2.10704200
H	-0.71012100	1.40445300	1.71233800
C	-2.59696200	1.31492200	0.72304300
C	-3.88429100	1.00487700	1.17784100
C	-4.97839800	1.67329000	0.63179500
H	-4.01896000	0.24903900	1.94571200
C	-3.43996200	2.87001800	-0.75178700
C	-4.75643100	2.63105300	-0.35612400
H	-5.98620000	1.44818000	0.97098800
H	-3.22183900	3.60875400	-1.52200700
H	-5.57652000	3.17798900	-0.81161500
N	-2.38321400	2.23599400	-0.23365500
N	0.83172600	-0.42581500	0.58087500
C	1.78924200	-0.38807000	-0.50665100
C	1.88776800	-1.57099200	-1.25599100
C	2.57234700	0.75021400	-0.84310200
C	2.74261900	-1.68375100	-2.34666200
H	1.26506000	-2.40993100	-0.96234500
C	3.42169300	0.59419400	-1.95623200
C	3.51515200	-0.58221900	-2.69717600
H	2.79793800	-2.61245600	-2.90753700
H	4.04473400	1.42357900	-2.26376600
H	4.19542500	-0.62880200	-3.54355600

C	2.53719500	2.13442100	-0.13325900
C	2.50346300	2.03700700	1.40957600
H	2.52414800	3.04802100	1.83301700
H	1.60917100	1.54076800	1.78341900
H	3.38111700	1.50398600	1.79355800
C	3.79352500	2.97501200	-0.47359100
H	3.82416200	3.28645900	-1.52276900
H	3.78019000	3.89060000	0.12725500
H	4.72133100	2.43836300	-0.24454300
C	1.30985100	2.93897400	-0.62909100
H	1.32177000	3.02850700	-1.72173700
H	0.35312200	2.49765900	-0.34116700
H	1.33937400	3.95306900	-0.21061400
C	1.12606800	-1.09061300	1.75647200
C	2.57333300	-1.54075800	1.97205300
H	2.71628000	-2.46759900	1.39902200
H	3.27197400	-0.82068600	1.53769100
C	2.88077300	-1.78557700	3.44996400
H	2.17877800	-2.50460300	3.87874600
H	3.89924700	-2.17129200	3.56717100
H	2.79650600	-0.85988400	4.02907100
O	0.27266400	-1.31830700	2.61195900

B3LYP/6-31G*/SMD (DMSO)

SCF Done: E(RB3LYP) = -1193.59499350

Zero-point correction= 0.504895
Thermal correction to Gibbs Free Energy= 0.444102

C	-2.44523800	-2.42224800	-2.23026600
C	-1.73312200	-1.34796400	-1.69322800
C	-1.34493100	-1.33701900	-0.34653200
C	-1.69219200	-2.43492400	0.45853000
C	-2.39812400	-3.51134500	-0.07968200
C	-2.77862600	-3.51004100	-1.42386500
H	-2.73665800	-2.40715000	-3.27741900
H	-1.47824200	-0.50232800	-2.32748500
H	-1.39614900	-2.44422300	1.50167900
H	-2.65562400	-4.35432100	0.55686200
H	-3.33165800	-4.34958300	-1.83727600
C	-0.58558600	-0.12589000	0.19475100
H	-0.48524200	0.57413200	-0.63645100
C	-1.36972700	0.63559200	1.29263100
H	-1.64063500	-0.03447700	2.10704200
H	-0.71012100	1.40445300	1.71233800
C	-2.59696200	1.31492200	0.72304300
C	-3.88429100	1.00487700	1.17784100
C	-4.97839800	1.67329000	0.63179500
H	-4.01896000	0.24903900	1.94571200
C	-3.43996200	2.87001800	-0.75178700
C	-4.75643100	2.63105300	-0.35612400
H	-5.98620000	1.44818000	0.97098800
H	-3.22183900	3.60875400	-1.52200700
H	-5.57652000	3.17798900	-0.81161500
N	-2.38321400	2.23599400	-0.23365500
N	0.83172600	-0.42581500	0.58087500
C	1.78924200	-0.38807000	-0.50665100
C	1.88776800	-1.57099200	-1.25599100
C	2.57234700	0.75021400	-0.84310200
C	2.74261900	-1.68375100	-2.34666200
H	1.26506000	-2.40993100	-0.96234500
C	3.42169300	0.59419400	-1.95623200
C	3.51515200	-0.58221900	-2.69717600
H	2.79793800	-2.61245600	-2.90753700
H	4.04473400	1.42357900	-2.26376600
H	4.19542500	-0.62880200	-3.54355600

C	2.53719500	2.13442100	-0.13325900
C	2.50346300	2.03700700	1.40957600
H	2.52414800	3.04802100	1.83301700
H	1.60917100	1.54076800	1.78341900
H	3.38111700	1.50398600	1.79355800
C	3.79352500	2.97501200	-0.47359100
H	3.82416200	3.28645900	-1.52276900
H	3.78019000	3.89060000	0.12725500
H	4.72133100	2.43836300	-0.24454300
C	1.30985100	2.93897400	-0.62909100
H	1.32177000	3.02850700	-1.72173700
H	0.35312200	2.49765900	-0.34116700
H	1.33937400	3.95306900	-0.21061400
C	1.12606800	-1.09061300	1.75647200
C	2.57333300	-1.54075800	1.97205300
H	2.71628000	-2.46759900	1.39902200
H	3.27197400	-0.82068600	1.53769100
C	2.88077300	-1.78557700	3.44996400
H	2.17877800	-2.50460300	3.87874600
H	3.89924700	-2.17129200	3.56717100
H	2.79650600	-0.85988400	4.02907100
O	0.27266400	-1.31830700	2.61195900

B3LYP/6-31G*/SMD (toluene)

SCF Done: E(RB3LYP) = -1193.60083233

Zero-point correction= 0.505495
Thermal correction to Gibbs Free Energy= 0.446042

C	-2.44523800	-2.42224800	-2.23026600
C	-1.73312200	-1.34796400	-1.69322800
C	-1.34493100	-1.33701900	-0.34653200
C	-1.69219200	-2.43492400	0.45853000
C	-2.39812400	-3.51134500	-0.07968200
C	-2.77862600	-3.51004100	-1.42386500
H	-2.73665800	-2.40715000	-3.27741900
H	-1.47824200	-0.50232800	-2.32748500
H	-1.39614900	-2.44422300	1.50167900
H	-2.65562400	-4.35432100	0.55686200
H	-3.33165800	-4.34958300	-1.83727600
C	-0.58558600	-0.12589000	0.19475100
H	-0.48524200	0.57413200	-0.63645100
C	-1.36972700	0.63559200	1.29263100
H	-1.64063500	-0.03447700	2.10704200
H	-0.71012100	1.40445300	1.71233800
C	-2.59696200	1.31492200	0.72304300
C	-3.88429100	1.00487700	1.17784100
C	-4.97839800	1.67329000	0.63179500
H	-4.01896000	0.24903900	1.94571200
C	-3.43996200	2.87001800	-0.75178700
C	-4.75643100	2.63105300	-0.35612400
H	-5.98620000	1.44818000	0.97098800
H	-3.22183900	3.60875400	-1.52200700
H	-5.57652000	3.17798900	-0.81161500
N	-2.38321400	2.23599400	-0.23365500
N	0.83172600	-0.42581500	0.58087500
C	1.78924200	-0.38807000	-0.50665100
C	1.88776800	-1.57099200	-1.25599100
C	2.57234700	0.75021400	-0.84310200
C	2.74261900	-1.68375100	-2.34666200
H	1.26506000	-2.40993100	-0.96234500
C	3.42169300	0.59419400	-1.95623200
C	3.51515200	-0.58221900	-2.69717600
H	2.79793800	-2.61245600	-2.90753700
H	4.04473400	1.42357900	-2.26376600
H	4.19542500	-0.62880200	-3.54355600

C	2.53719500	2.13442100	-0.13325900
C	2.50346300	2.03700700	1.40957600
H	2.52414800	3.04802100	1.83301700
H	1.60917100	1.54076800	1.78341900
H	3.38111700	1.50398600	1.79355800
C	3.79352500	2.97501200	-0.47359100
H	3.82416200	3.28645900	-1.52276900
H	3.78019000	3.89060000	0.12725500
H	4.72133100	2.43836300	-0.24454300
C	1.30985100	2.93897400	-0.62909100
H	1.32177000	3.02850700	-1.72173700
H	0.35312200	2.49765900	-0.34116700
H	1.33937400	3.95306900	-0.21061400
C	1.12606800	-1.09061300	1.75647200
C	2.57333300	-1.54075800	1.97205300
H	2.71628000	-2.46759900	1.39902200
H	3.27197400	-0.82068600	1.53769100
C	2.88077300	-1.78557700	3.44996400
H	2.17877800	-2.50460300	3.87874600
H	3.89924700	-2.17129200	3.56717100
H	2.79650600	-0.85988400	4.02907100
O	0.27266400	-1.31830700	2.61195900

M06-2X/6-31G*

SCF Done: E(RM062X) = -1193.06544987

Zero-point correction= 0.508876
Thermal correction to Gibbs Free Energy= 0.452180

C	-2.44523800	-2.42224800	-2.23026600
C	-1.73312200	-1.34796400	-1.69322800
C	-1.34493100	-1.33701900	-0.34653200
C	-1.69219200	-2.43492400	0.45853000
C	-2.39812400	-3.51134500	-0.07968200
C	-2.77862600	-3.51004100	-1.42386500
H	-2.73665800	-2.40715000	-3.27741900
H	-1.47824200	-0.50232800	-2.32748500
H	-1.39614900	-2.44422300	1.50167900
H	-2.65562400	-4.35432100	0.55686200
H	-3.33165800	-4.34958300	-1.83727600
C	-0.58558600	-0.12589000	0.19475100
H	-0.48524200	0.57413200	-0.63645100
C	-1.36972700	0.63559200	1.29263100
H	-1.64063500	-0.03447700	2.10704200
H	-0.71012100	1.40445300	1.71233800
C	-2.59696200	1.31492200	0.72304300
C	-3.88429100	1.00487700	1.17784100
C	-4.97839800	1.67329000	0.63179500
H	-4.01896000	0.24903900	1.94571200
C	-3.43996200	2.87001800	-0.75178700
C	-4.75643100	2.63105300	-0.35612400
H	-5.98620000	1.44818000	0.97098800
H	-3.22183900	3.60875400	-1.52200700
H	-5.57652000	3.17798900	-0.81161500
N	-2.38321400	2.23599400	-0.23365500
N	0.83172600	-0.42581500	0.58087500
C	1.78924200	-0.38807000	-0.50665100
C	1.88776800	-1.57099200	-1.25599100
C	2.57234700	0.75021400	-0.84310200
C	2.74261900	-1.68375100	-2.34666200
H	1.26506000	-2.40993100	-0.96234500
C	3.42169300	0.59419400	-1.95623200
C	3.51515200	-0.58221900	-2.69717600
H	2.79793800	-2.61245600	-2.90753700
H	4.04473400	1.42357900	-2.26376600
H	4.19542500	-0.62880200	-3.54355600

C	2.53719500	2.13442100	-0.13325900
C	2.50346300	2.03700700	1.40957600
H	2.52414800	3.04802100	1.83301700
H	1.60917100	1.54076800	1.78341900
H	3.38111700	1.50398600	1.79355800
C	3.79352500	2.97501200	-0.47359100
H	3.82416200	3.28645900	-1.52276900
H	3.78019000	3.89060000	0.12725500
H	4.72133100	2.43836300	-0.24454300
C	1.30985100	2.93897400	-0.62909100
H	1.32177000	3.02850700	-1.72173700
H	0.35312200	2.49765900	-0.34116700
H	1.33937400	3.95306900	-0.21061400
C	1.12606800	-1.09061300	1.75647200
C	2.57333300	-1.54075800	1.97205300
H	2.71628000	-2.46759900	1.39902200
H	3.27197400	-0.82068600	1.53769100
C	2.88077300	-1.78557700	3.44996400
H	2.17877800	-2.50460300	3.87874600
H	3.89924700	-2.17129200	3.56717100
H	2.79650600	-0.85988400	4.02907100
O	0.27266400	-1.31830700	2.61195900

PBE0/def2-TZVPP

SCF Done: E(RPBE1PBE) = -1192.58547918

Zero-point correction= 0.502163
Thermal correction to Gibbs Free Energy= 0.448122

C	-2.44523800	-2.42224800	-2.23026600
C	-1.73312200	-1.34796400	-1.69322800
C	-1.34493100	-1.33701900	-0.34653200
C	-1.69219200	-2.43492400	0.45853000
C	-2.39812400	-3.51134500	-0.07968200
C	-2.77862600	-3.51004100	-1.42386500
H	-2.73665800	-2.40715000	-3.27741900
H	-1.47824200	-0.50232800	-2.32748500
H	-1.39614900	-2.44422300	1.50167900
H	-2.65562400	-4.35432100	0.55686200
H	-3.33165800	-4.34958300	-1.83727600
C	-0.58558600	-0.12589000	0.19475100
H	-0.48524200	0.57413200	-0.63645100
C	-1.36972700	0.63559200	1.29263100
H	-1.64063500	-0.03447700	2.10704200
H	-0.71012100	1.40445300	1.71233800
C	-2.59696200	1.31492200	0.72304300
C	-3.88429100	1.00487700	1.17784100
C	-4.97839800	1.67329000	0.63179500
H	-4.01896000	0.24903900	1.94571200
C	-3.43996200	2.87001800	-0.75178700
C	-4.75643100	2.63105300	-0.35612400
H	-5.98620000	1.44818000	0.97098800
H	-3.22183900	3.60875400	-1.52200700
H	-5.57652000	3.17798900	-0.81161500
N	-2.38321400	2.23599400	-0.23365500
N	0.83172600	-0.42581500	0.58087500
C	1.78924200	-0.38807000	-0.50665100
C	1.88776800	-1.57099200	-1.25599100
C	2.57234700	0.75021400	-0.84310200
C	2.74261900	-1.68375100	-2.34666200
H	1.26506000	-2.40993100	-0.96234500
C	3.42169300	0.59419400	-1.95623200
C	3.51515200	-0.58221900	-2.69717600
H	2.79793800	-2.61245600	-2.90753700
H	4.04473400	1.42357900	-2.26376600
H	4.19542500	-0.62880200	-3.54355600

C	2.53719500	2.13442100	-0.13325900
C	2.50346300	2.03700700	1.40957600
H	2.52414800	3.04802100	1.83301700
H	1.60917100	1.54076800	1.78341900
H	3.38111700	1.50398600	1.79355800
C	3.79352500	2.97501200	-0.47359100
H	3.82416200	3.28645900	-1.52276900
H	3.78019000	3.89060000	0.12725500
H	4.72133100	2.43836300	-0.24454300
C	1.30985100	2.93897400	-0.62909100
H	1.32177000	3.02850700	-1.72173700
H	0.35312200	2.49765900	-0.34116700
H	1.33937400	3.95306900	-0.21061400
C	1.12606800	-1.09061300	1.75647200
C	2.57333300	-1.54075800	1.97205300
H	2.71628000	-2.46759900	1.39902200
H	3.27197400	-0.82068600	1.53769100
C	2.88077300	-1.78557700	3.44996400
H	2.17877800	-2.50460300	3.87874600
H	3.89924700	-2.17129200	3.56717100
H	2.79650600	-0.85988400	4.02907100
O	0.27266400	-1.31830700	2.61195900

6b

B3LYP/6-31G*

SCF Done: E(RB3LYP) = -1343.76969719

Zero-point correction= 0.499196
Thermal correction to Gibbs Free Energy (423.15 K)= 0.394582

C	-2.55287000	3.23597100	0.12549500
C	-2.01941400	1.98847600	0.45699300
C	-1.52213300	1.12724800	-0.53145500
C	-1.57930500	1.54343200	-1.87146700
C	-2.10891200	2.79111500	-2.20213600
C	-2.59783500	3.64182600	-1.20788900
H	-2.93437600	3.88552000	0.90917000
H	-1.99935300	1.67339000	1.49776000
H	-1.18540800	0.89149000	-2.64296800
H	-2.13948100	3.09994400	-3.24412200
H	-3.01104000	4.61203700	-1.47151700
C	-0.99474000	-0.24139900	-0.10861400
H	-1.13072200	-0.30654200	0.97194700
C	-1.83205900	-1.41025600	-0.69266200
H	-1.88758500	-1.34444100	-1.77855700
H	-1.32212000	-2.35009300	-0.44757800
C	-3.21787500	-1.44750300	-0.08422800
C	-4.36744700	-1.28907700	-0.86747800
C	-5.61924300	-1.34742100	-0.25761300
H	-4.27510500	-1.12028400	-1.93616200
C	-4.48770900	-1.69903600	1.81853400
C	-5.68671000	-1.55889000	1.11819300
H	-6.52478800	-1.22780600	-0.84677500
H	-4.49582500	-1.86340000	2.89544600
H	-6.63830800	-1.61213100	1.63864000
N	-3.28162300	-1.64743400	1.24445700
N	0.48889200	-0.43374900	-0.27810300
C	1.23184700	-0.49169100	0.96620600
C	1.03610500	-1.67088400	1.70720900
C	2.03269300	0.55459900	1.47906100
C	1.59189900	-1.85275100	2.96471100
H	0.42038100	-2.45040600	1.26870300
C	2.56858400	0.33557900	2.76676500
C	2.36404400	-0.82485800	3.50271700
H	1.42099800	-2.77485600	3.51292000
H	3.17309400	1.11604600	3.21610000

H	2.80786300	-0.92253100	4.49002800
C	2.40253100	1.91390600	0.82225600
C	1.75335400	3.05079800	1.65133600
H	2.05224900	4.02634000	1.24875000
H	0.66058400	2.98931100	1.60604600
H	2.05152100	3.01715900	2.70445900
C	3.94558000	2.07934000	0.84299000
H	4.42873900	1.26460700	0.29252200
H	4.21981300	3.02442500	0.35966000
H	4.36345200	2.09725500	1.85374000
C	1.97225800	2.11117200	-0.64404400
H	2.44300200	1.38573400	-1.31352800
H	0.89273900	2.06364800	-0.78093700
H	2.30139900	3.10673400	-0.96571400
C	0.98602600	-0.82598300	-1.49777300
O	0.29849900	-0.81291100	-2.52421700
C	2.38351100	-1.29885600	-1.65354200
C	2.97476700	-1.70547000	-2.82226100
O	3.28286100	-1.41429100	-0.62372500
C	4.30695900	-2.09241100	-2.49695400
H	2.49075500	-1.71960200	-3.78700100
C	4.43686900	-1.89484300	-1.15291800
H	5.06635000	-2.46723900	-3.16883400
H	5.24476600	-2.04120100	-0.45172900

11b

B3LYP/6-31G*

SCF Done: E(RB3LYP) = -1343.77337655

Zero-point correction= 0.499403
Thermal correction to Gibbs Free Energy (423.15 K)= 0.395738

C	2.64533700	-2.29052100	2.48839500
C	2.07091400	-1.23036400	1.78394500
C	1.48734000	-1.43097500	0.52669300
C	1.49023500	-2.72670200	-0.01552700
C	2.05718900	-3.78730500	0.69122800
C	2.63836900	-3.57422200	1.94389500
H	3.09373500	-2.11143700	3.46231700
H	2.07796600	-0.23214200	2.21505000
H	1.03760300	-2.89477200	-0.98719800
H	2.04801400	-4.78502400	0.25922700
H	3.08195900	-4.40307700	2.48965300
C	0.89804100	-0.23578100	-0.22079400
H	1.02743600	0.63702700	0.42303400
C	1.67232700	0.08609500	-1.52292500
H	1.83164000	-0.81469500	-2.11414000
H	1.06617800	0.76336400	-2.13434000
C	2.99276100	0.76422700	-1.21858800
C	4.20696200	0.23945100	-1.67725000
C	5.39169700	0.91711900	-1.39577800
H	4.21570800	-0.68691900	-2.24377000
C	4.07781800	2.54444000	-0.23904200
C	5.33120700	2.09989100	-0.66106200
H	6.34488700	0.52641700	-1.74244400
H	3.98548000	3.46243900	0.33988000
H	6.22665300	2.66330600	-0.41641200
N	2.93522200	1.90267600	-0.50379800
N	-0.58765400	-0.30709900	-0.40666900
C	-1.34328200	-0.05158400	0.80368500
C	-1.63605600	-1.19192600	1.57111600
C	-1.70940200	1.23676600	1.25839600
C	-2.27627200	-1.10509600	2.80015600
H	-1.32931900	-2.15810700	1.18363100
C	-2.32605000	1.28420200	2.52557000
C	-2.60995100	0.15612700	3.28759400
H	-2.49145400	-2.00379200	3.37120200
H	-2.60877100	2.25043700	2.93007300

H	-3.09363100	0.26690600	4.25463000
C	-1.55549100	2.60065200	0.53037500
C	-0.99929100	2.55109300	-0.90612200
H	-1.04671800	3.56370600	-1.32545000
H	0.04767200	2.24693600	-0.93529800
H	-1.58454400	1.89623600	-1.55556100
C	-2.95574100	3.26273900	0.43158000
H	-3.41438800	3.44029000	1.40862200
H	-2.87140500	4.23217400	-0.07370900
H	-3.63672200	2.63358300	-0.15170300
C	-0.61204900	3.51160500	1.35535100
H	-0.96124100	3.65071700	2.38373600
H	0.40021000	3.09356900	1.39200400
H	-0.54652100	4.50170700	0.88774200
C	-1.12200900	-1.02218200	-1.45574900
O	-0.43405900	-1.65173500	-2.26497700
C	-2.58660000	-1.02025800	-1.67996000
C	-3.29098100	-1.80807200	-2.55215200
O	-3.44459100	-0.15437300	-1.04987400
C	-4.65751200	-1.41503600	-2.44882300
H	-2.86039100	-2.56928900	-3.18531300
C	-4.68972500	-0.40924300	-1.52734100
H	-5.50226200	-1.82216900	-2.98659300
H	-5.48025900	0.20159000	-1.11798200

6f

B3LYP/6-31G*

SCF Done: E(RB3LYP) = -3917.09670491

Zero-point correction= 0.519828
Thermal correction to Gibbs Free Energy (423.15 K)= 0.409025

C	-4.30343600	2.60088400	-1.00431800
C	-3.58401200	1.65420800	-0.27171500
C	-2.77405600	0.70560800	-0.91182700
C	-2.70574000	0.72173100	-2.31414100
C	-3.42403400	1.66722700	-3.04624900
C	-4.22511200	2.60948000	-2.39661600
H	-4.92635600	3.32537600	-0.48604600
H	-3.65886900	1.64679200	0.81339200
H	-2.06452400	0.00890700	-2.81982200
H	-3.35557900	1.66867700	-4.13126100
H	-4.78354600	3.34331100	-2.97202100
C	-2.03850700	-0.32279200	-0.05632500
H	-2.39321300	-0.17994300	0.96511200
C	-2.40383900	-1.78736800	-0.41202100
H	-2.29972500	-1.97104000	-1.48087400
H	-1.69710500	-2.44933300	0.10261700
C	-3.80074700	-2.12753200	0.06116700
C	-4.82672100	-2.44852200	-0.83496000
C	-6.08979800	-2.76407400	-0.33692200
H	-4.63284100	-2.44631600	-1.90333000
C	-5.21127000	-2.40883100	1.85809000
C	-6.29167300	-2.74700400	1.04177800
H	-6.90115700	-3.01716000	-1.01437600
H	-5.32722700	-2.38189900	2.94088100
H	-7.25719000	-2.98626900	1.47724900
N	-3.99536100	-2.10615000	1.39239500
N	-0.55471500	-0.10275000	0.06197100
C	-0.11119800	0.47130100	1.31338400
C	-0.35678400	-0.33726400	2.44341200
C	0.48579300	1.74532300	1.46425500
C	0.00802800	0.04921800	3.72327600
H	-0.84522900	-1.29526500	2.28885200
C	0.86743800	2.08637300	2.78261600
C	0.64728500	1.27802600	3.88887600
H	-0.19230500	-0.60028900	4.57059900
H	1.34970500	3.04346500	2.94846000

H	0.96374500	1.61158800	4.87344200
C	0.75690400	2.84582400	0.39826400
C	0.32726100	2.53902000	-1.04851100
H	0.49662700	3.43933200	-1.65135200
H	0.91675700	1.74097900	-1.50254900
H	-0.72928500	2.28355500	-1.12906200
C	-0.02491600	4.11967300	0.81857800
H	0.24745100	4.47625300	1.81630200
H	0.17975800	4.93002500	0.10899000
H	-1.10420400	3.92990500	0.81083800
C	2.27481300	3.15822700	0.36472200
H	2.65736900	3.50511100	1.32965200
H	2.84966000	2.27232700	0.07434300
H	2.47353600	3.94635400	-0.37136300
C	0.28299600	-0.60154100	-0.91021400
O	-0.15514400	-0.98129600	-1.99823700
C	1.77417600	-0.70991600	-0.67839200
C	2.58090400	-0.54732100	-1.81648700
C	2.38639000	-1.08048000	0.52701000
C	3.96229000	-0.70169300	-1.75192700
H	2.10445400	-0.30897000	-2.76131200
C	3.76816700	-1.26177300	0.60029400
H	1.79660200	-1.24394200	1.41985500
C	4.54538400	-1.05842100	-0.53664300
H	4.57774600	-0.55863800	-2.63333100
H	4.23326900	-1.55824200	1.53403000
Br	6.44107000	-1.28421500	-0.43323700

11f

B3LYP/6-31G*

SCF Done: E(RB3LYP) = -3917.09897974

Zero-point correction= 0.519809
Thermal correction to Gibbs Free Energy (423.15 K)= 0.409538

C	2.56572400	-3.66772100	1.63985400
C	2.07250400	-2.75635300	0.70593800
C	2.37597800	-1.38917800	0.81539100
C	3.18831300	-0.96874300	1.87655000
C	3.68795400	-1.88140500	2.80767700
C	3.37605700	-3.23560800	2.69265000
H	2.31863700	-4.72195900	1.54185800
H	1.44111100	-3.09628000	-0.10770900
H	3.43514500	0.08576500	1.97318900
H	4.31779800	-1.53184000	3.62171400
H	3.76127600	-3.94989500	3.41578200
C	1.87541900	-0.35405500	-0.19137400
H	2.30196800	0.60516400	0.10711300
C	2.39812900	-0.61460700	-1.62456300
H	2.25336300	-1.65515300	-1.91030400
H	1.81408200	-0.01108800	-2.32825300
C	3.85874600	-0.22912500	-1.75131100
C	4.81566700	-1.14084500	-2.21302400
C	6.14118200	-0.73068700	-2.34210600
H	4.51916600	-2.15491100	-2.46390600
C	5.45793500	1.41643700	-1.54535900
C	6.47553300	0.57929100	-2.00342600
H	6.89928100	-1.42320800	-2.69850100
H	5.67577600	2.44664100	-1.26697600
H	7.49445300	0.94527400	-2.08638100
N	4.18317300	1.03329800	-1.41829500
N	0.39282700	-0.10992900	-0.11396400
C	0.00000600	0.64353500	1.05933800
C	-0.39233800	-0.13231900	2.16452500
C	0.07525100	2.05525900	1.16265400
C	-0.71727800	0.43837600	3.38714000
H	-0.42025700	-1.21033700	2.04377000
C	-0.22629800	2.59045600	2.43270500
C	-0.61654400	1.82078000	3.52276900
H	-1.02137300	-0.18789500	4.22082100
H	-0.15988100	3.66387800	2.57322000

H	-0.84121200	2.30333200	4.47018700
C	0.42703200	3.09776200	0.06038200
C	1.75573400	3.79970900	0.43942600
H	1.98206300	4.58744600	-0.28955500
H	1.70977000	4.26330300	1.43036500
H	2.59048700	3.09053200	0.42973800
C	0.59094000	2.55479700	-1.37437800
H	-0.27246400	1.97582500	-1.71229600
H	0.69794700	3.41019000	-2.05233600
H	1.49074000	1.95100700	-1.48865700
C	-0.70321300	4.16110800	-0.00443000
H	-1.66178900	3.70673000	-0.28166400
H	-0.84936700	4.69203000	0.94003300
H	-0.45801400	4.90955600	-0.76660900
C	-0.47616300	-0.97953300	-0.74482900
O	-0.06999000	-1.93160500	-1.41443800
C	-1.97010500	-0.76531600	-0.65741700
C	-2.75863300	-1.91306400	-0.83471800
C	-2.61420300	0.47020000	-0.50910100
C	-4.14838600	-1.84200400	-0.83493500
H	-2.26100000	-2.86471000	-0.98461600
C	-4.00564800	0.56001200	-0.52729000
H	-2.04031600	1.37805000	-0.38110900
C	-4.76126000	-0.59950800	-0.68118200
H	-4.74887900	-2.73631500	-0.96058100
H	-4.49584500	1.52152200	-0.42121300
Br	-6.66951300	-0.48369400	-0.68831900

6h

B3LYP/6-31G*

SCF Done: E(RB3LYP) = -1643.68704870

Zero-point correction= 0.505128
Thermal correction to Gibbs Free Energy (423.15 K)= 0.394133

C	-3.77471400	2.89769400	-0.25287900
C	-3.01558500	1.83084400	0.23337800
C	-2.23087000	1.05136000	-0.62716000
C	-2.22759100	1.35975700	-1.99676000
C	-2.98344800	2.42653200	-2.48193600
C	-3.75995000	3.19890400	-1.61423500
H	-4.37678300	3.48861800	0.43256500
H	-3.03904300	1.59550100	1.29502100
H	-1.60763800	0.77568100	-2.66814400
H	-2.96382600	2.65715300	-3.54413300
H	-4.34785700	4.02883400	-1.99769300
C	-1.46053800	-0.13053800	-0.04813900
H	-1.69355600	-0.16740200	1.01727200
C	-1.94023600	-1.48940800	-0.62552100
H	-1.92981900	-1.47353700	-1.71579200
H	-1.23560000	-2.26525900	-0.30412700
C	-3.31695500	-1.84670100	-0.10879100
C	-4.41450900	-1.98287100	-0.96661600
C	-5.65476400	-2.32921700	-0.43317300
H	-4.29269600	-1.81613900	-2.03264000
C	-4.61591500	-2.36146000	1.71984000
C	-5.76304900	-2.52619200	0.94210900
H	-6.52051100	-2.44125100	-1.08066200
H	-4.65783600	-2.50368500	2.79903400
H	-6.70750300	-2.79751100	1.40421000
N	-3.42096500	-2.03157400	1.21968300
N	0.03912300	-0.00850500	-0.06629700
C	0.68422200	0.20792500	1.21541100
C	0.56993100	-0.86166800	2.12509900
C	1.35138100	1.39971100	1.59157300
C	1.14339200	-0.82111200	3.38651700
H	0.01971500	-1.74187900	1.81349500
C	1.94532300	1.38505700	2.87392700
C	1.85896100	0.31667600	3.75591900
H	1.04085200	-1.66557300	4.06199800
H	2.48693900	2.26547800	3.20216700

H	2.33661700	0.38076700	4.72999500
C	1.47755000	2.74825300	0.82525500
C	0.80479900	2.82693500	-0.55720200
H	0.93856400	3.84536600	-0.94228100
H	1.24628600	2.15019900	-1.28798500
H	-0.26666400	2.63552400	-0.50704800
C	0.80898000	3.84855200	1.69385600
H	1.26751500	3.94776400	2.68226200
H	0.89270100	4.81836200	1.18933300
H	-0.25622100	3.63442300	1.83638600
C	2.97480200	3.09584200	0.62674000
H	3.53383500	3.12523900	1.56704100
H	3.45978400	2.37200000	-0.03494000
H	3.06269500	4.08358500	0.15862200
C	0.74213900	-0.42241200	-1.16888800
O	0.25373200	-0.52348800	-2.28961200
C	2.18127600	-0.86763600	-0.97198300
C	3.27015700	-0.20921400	-1.54534100
C	2.46440800	-2.11707200	-0.41412200
C	4.57043300	-0.69707700	-1.51265900
C	3.73656300	-2.66926600	-0.35382900
C	4.77489000	-1.93186100	-0.90956500
H	5.38714500	-0.13849400	-1.95278900
H	3.90485800	-3.64148500	0.09235700
F	6.02056900	-2.43443900	-0.86849700
F	3.06754700	0.98209500	-2.14202600
F	1.44017200	-2.84386200	0.07993700

11h

B3LYP/6-31G*

SCF Done: E(RB3LYP) = -1643.69082357

Zero-point correction= 0.505424
Thermal correction to Gibbs Free Energy (423.15 K)= 0.394785

C	2.99559700	-2.61235500	2.33761200
C	2.55866500	-1.44782000	1.70275700
C	1.83780300	-1.50720600	0.50352500
C	1.56035400	-2.76712600	-0.05205600
C	1.98908600	-3.93072800	0.58648000
C	2.70962500	-3.85866400	1.78142300
H	3.55519900	-2.54298100	3.26688600
H	2.78350600	-0.47886200	2.14179600
H	1.00063900	-2.83172900	-0.97931900
H	1.76209300	-4.89827700	0.14595100
H	3.04517400	-4.76792800	2.27344600
C	1.40235400	-0.20805900	-0.17100700
H	1.73705000	0.61229300	0.46607900
C	2.09381600	0.00705000	-1.53956500
H	2.01503300	-0.88481100	-2.15959900
H	1.57648100	0.81102300	-2.07310300
C	3.54635800	0.40025400	-1.35857900
C	4.57933300	-0.31987400	-1.97048000
C	5.89687100	0.10024700	-1.79864100
H	4.34711000	-1.19559400	-2.56913700
C	5.05608200	1.87794300	-0.44021100
C	6.14680000	1.22696300	-1.01718100
H	6.71346700	-0.44470600	-2.26504000
H	5.20649800	2.76053100	0.18012300
H	7.15611200	1.59239600	-0.85346100
N	3.78842600	1.48386400	-0.59924400
N	-0.08991700	-0.04204400	-0.20681700
C	-0.68961900	0.38244100	1.04571000
C	-1.06483900	-0.66756500	1.90437300
C	-0.85356800	1.73225100	1.44442100
C	-1.63663600	-0.43460400	3.14687400
H	-0.90270200	-1.68357800	1.56414100
C	-1.43862400	1.92171200	2.71551200
C	-1.82661400	0.88384100	3.55360900
H	-1.92240900	-1.26728300	3.78316800
H	-1.60180900	2.93592500	3.06320300

H	-2.27205300	1.10882100	4.51908300
C	-0.50000600	3.04131700	0.68054100
C	0.44703200	3.89618200	1.56351000
H	0.70431800	4.82188800	1.03528300
H	0.00106800	4.17762200	2.52212600
H	1.37934700	3.35823900	1.76895800
C	0.20658400	2.88276600	-0.67943900
H	1.21666600	2.48003400	-0.57572500
H	-0.36123800	2.26927800	-1.37792400
H	0.31063300	3.87950700	-1.12560300
C	-1.81118200	3.82898400	0.42353800
H	-2.36987400	4.02843500	1.34319200
H	-1.57736900	4.79472400	-0.04058800
H	-2.46443400	3.27807300	-0.25968200
C	-0.83973100	-0.66241700	-1.17187100
O	-0.36981600	-1.25983400	-2.13475100
C	-2.35354600	-0.61191300	-1.04027600
C	-3.13447400	0.42617600	-1.54217300
C	-3.05503800	-1.73540500	-0.59783000
C	-4.52357400	0.40168500	-1.57305500
C	-4.43974400	-1.82820200	-0.59586900
C	-5.15016600	-0.74096600	-1.09230800
H	-5.09001800	1.23551300	-1.96884000
H	-4.94200500	-2.71476900	-0.22922600
F	-6.49318800	-0.79908200	-1.10905900
F	-2.51465000	1.52408400	-2.02667400
F	-2.34677400	-2.78159000	-0.12780000

6i

B3LYP/6-31G*

SCF Done: E(RB3LYP) = -1550.49279636

Zero-point correction= 0.532389
Thermal correction to Gibbs Free Energy (423.15 K)= 0.419052

C	-3.88247800	2.81165300	-0.72814100
C	-3.20041300	1.77013100	-0.09538300
C	-2.44682000	0.84548100	-0.83200900
C	-2.39695800	0.98348300	-2.22835800
C	-3.07755000	2.02410300	-2.86061400
C	-3.82240700	2.94132700	-2.11533900
H	-4.46229700	3.51544900	-0.13664700
H	-3.26101600	1.66886700	0.98587400
H	-1.80014800	0.28846300	-2.80794000
H	-3.02410500	2.11939800	-3.94220900
H	-4.35194400	3.74947500	-2.61313000
C	-1.75054900	-0.28874200	-0.08528300
H	-2.08007000	-0.22180900	0.95218400
C	-2.19009900	-1.69691300	-0.56249500
H	-2.11907600	-1.78662500	-1.64606500
H	-1.50369000	-2.43441400	-0.12924700
C	-3.59048600	-2.01721200	-0.08464400
C	-4.64593900	-2.22943700	-0.97853000
C	-5.91012600	-2.53411100	-0.47621900
H	-4.47411200	-2.15342600	-2.04798700
C	-4.97365100	-2.38142300	1.71844100
C	-6.08292100	-2.61481700	0.90411600
H	-6.74451200	-2.70374700	-1.15165900
H	-5.06627900	-2.43305000	2.80250900
H	-7.04807000	-2.84980600	1.34261100
N	-3.75652100	-2.09041500	1.24846900
N	-0.25476400	-0.15012700	0.02195300
C	0.24145800	0.28116200	1.31153800
C	-0.02155000	-0.61974100	2.36512000
C	0.90413200	1.50505400	1.56620100
C	0.39043000	-0.37627700	3.66557500
H	-0.56353300	-1.53246900	2.13375100
C	1.33148100	1.69832800	2.90051800
C	1.09525400	0.79829600	3.92987100
H	0.17552000	-1.09337300	4.45255000
H	1.86402600	2.61097800	3.14423300

H	1.45003700	1.01941800	4.93276900
C	1.20033400	2.69191600	0.60454300
C	0.72677400	2.54480500	-0.85346400
H	0.92355300	3.49062900	-1.37213800
H	1.26997000	1.77018600	-1.39738900
H	-0.34153100	2.34335000	-0.93144900
C	0.48222800	3.94894100	1.16568800
H	0.79098000	4.19467800	2.18594400
H	0.70581700	4.81587200	0.53294200
H	-0.60399000	3.80420300	1.16723900
C	2.72880700	2.94593700	0.56251200
H	3.14768000	3.17838200	1.54634300
H	3.26006200	2.07228400	0.16958700
H	2.94397000	3.79546000	-0.09641600
C	0.54096300	-0.59657200	-1.00415100
O	0.08128900	-0.86579800	-2.11514000
C	2.03192100	-0.79378700	-0.80663400
C	2.83989200	-0.50656300	-1.92014100
C	2.62103600	-1.36533000	0.33095300
C	4.21025500	-0.73406700	-1.88946300
H	2.37131900	-0.11470400	-2.81595100
C	3.98995200	-1.62144200	0.36758800
H	2.01939500	-1.62120800	1.19339200
C	4.76709200	-1.28994100	-0.73855800
H	4.84566700	-0.50212500	-2.73510600
H	4.45770700	-2.06918900	1.23556100
N	6.21645000	-1.54312600	-0.69539400
O	6.87917100	-1.23913300	-1.68696000
O	6.67980900	-2.04268600	0.32975100

11i

B3LYP/6-31G*

SCF Done: E(RB3LYP) = -1550.49510590

Zero-point correction=   0.532449
Thermal correction to Gibbs Free Energy (423.15 K)=   0.420213

C	2.25121800	-3.72422800	1.50553400
C	1.78523800	-2.77854000	0.59198300
C	2.05161700	-1.41248200	0.78221000
C	2.79981600	-1.02756900	1.90231100
C	3.27248400	-1.97431900	2.81321000
C	2.99738800	-3.32732000	2.61790900
H	2.03385500	-4.77722400	1.34468900
H	1.20447600	-3.09269300	-0.26847100
H	3.01782300	0.02570300	2.06132700
H	3.85283400	-1.65233400	3.67397700
H	3.36201000	-4.06802200	3.32485500
C	1.57733200	-0.34046700	-0.19757000
H	1.97557300	0.61083300	0.15916700
C	2.15607700	-0.52975300	-1.61987300
H	2.04037300	-1.55884800	-1.95621100
H	1.58851900	0.09504900	-2.31850000
C	3.61379200	-0.11610300	-1.67389300
C	4.60170700	-0.98678900	-2.14871800
C	5.92341600	-0.54907000	-2.20876500
H	4.33235600	-1.99061500	-2.46332000
C	5.17492200	1.54160300	-1.32479600
C	6.22237000	0.74612800	-1.78978100
H	6.70562300	-1.20933500	-2.57402000
H	5.36458700	2.55882200	-0.98505800
H	7.23687200	1.13210200	-1.81700400
N	3.90356000	1.13174600	-1.26357900
N	0.08777400	-0.12341700	-0.16627200
C	-0.37011100	0.57831700	1.01658800
C	-0.79087300	-0.24839200	2.07365000
C	-0.33485100	1.98643400	1.17382300
C	-1.18816000	0.26786400	3.29902900
H	-0.78153500	-1.32178700	1.91421300
C	-0.71273600	2.46569700	2.44586700
C	-1.13379100	1.64661400	3.48736200
H	-1.51296700	-0.39635800	4.09455300
H	-0.68286200	3.53440600	2.62799600

H	-1.41759900	2.08823700	4.43875900
C	0.04954600	3.07928100	0.13279200
C	0.31451400	2.59825300	-1.30891600
H	0.43800300	3.48241000	-1.94568100
H	1.23675700	2.02353100	-1.39107600
H	-0.50955800	2.01155100	-1.72324500
C	-1.10378300	4.11550000	0.04242200
H	-1.31837700	4.60435600	0.99640400
H	-0.83583100	4.89960700	-0.67478700
H	-2.03282500	3.64969300	-0.30700600
C	1.33457300	3.79901300	0.61570700
H	1.21802900	4.22528400	1.61737500
H	2.18664700	3.11082900	0.63220300
H	1.58290400	4.61769900	-0.07059100
C	-0.74078600	-0.97834000	-0.85983800
O	-0.30987600	-1.88999400	-1.56727000
C	-2.24419200	-0.79838900	-0.79284600
C	-3.00420900	-1.97109900	-0.93297100
C	-2.90618400	0.43453400	-0.70864400
C	-4.39302600	-1.92557600	-0.95387000
H	-2.48495400	-2.91687200	-1.03744900
C	-4.29615500	0.49939800	-0.75001600
H	-2.34307400	1.35289800	-0.61323300
C	-5.02046400	-0.68445300	-0.86216300
H	-4.99126200	-2.82333700	-1.04782500
H	-4.82112500	1.44488900	-0.69465800
N	-6.49071100	-0.62180300	-0.89068300
O	-7.01761500	0.48834300	-0.81779100
O	-7.10622900	-1.68342800	-0.98494700

6k

B3LYP/6-31G*

SCF Done: E(RB3LYP) = -1805.57930046

Zero-point correction=   0.519716
Thermal correction to Gibbs Free Energy (423.15 K)=   0.410307

C	-3.44212500	2.96220100	-0.59674400
C	-2.68596600	1.95932900	0.01401100
C	-1.94455500	1.04607000	-0.74895700
C	-1.98747300	1.15177900	-2.14834500
C	-2.73940800	2.15608700	-2.75788800
C	-3.46893400	3.06475400	-1.98713400
H	-4.00938900	3.65889000	0.01515700
H	-2.67907900	1.87756300	1.09846600
H	-1.41041100	0.45628500	-2.74762100
H	-2.75538800	2.22867200	-3.84254500
H	-4.05407300	3.84467000	-2.46761500
C	-1.18167600	-0.05924300	-0.02530500
H	-1.34176900	0.09464000	1.04309000
C	-1.76874000	-1.46255200	-0.33217500
H	-1.76486800	-1.65609500	-1.40509500
H	-1.12371600	-2.21479600	0.13625900
C	-3.16504700	-1.61152600	0.23347900
C	-4.26281300	-1.89490000	-0.58787100
C	-5.52261800	-2.04500900	-0.01122400
H	-4.12522300	-1.99279400	-1.66051600
C	-4.50184900	-1.61328500	2.10671300
C	-5.65054400	-1.90345500	1.36928600
H	-6.38834800	-2.26649100	-0.62998500
H	-4.55844100	-1.49189500	3.18786100
H	-6.61084000	-2.01123000	1.86470900
N	-3.28791800	-1.47002100	1.56548500
N	0.32091300	-0.02225000	-0.15643600
C	1.04686700	0.26351600	1.07022500
C	1.07940400	-0.79609000	1.99580500
C	1.63437000	1.51138800	1.39262800
C	1.69584000	-0.68104400	3.23270700
H	0.60618000	-1.73026000	1.71596600
C	2.25511700	1.58388400	2.65960000
C	2.29698400	0.53114600	3.56408300
H	1.70629900	-1.52208500	3.91994400
H	2.72188500	2.51733700	2.95441200

H	2.79191400	0.66285300	4.52262600
C	1.67654500	2.82921800	0.56702200
C	1.04065400	2.79305200	-0.83440800
H	1.16080800	3.78280000	-1.29138300
H	1.50897400	2.06997900	-1.50407300
H	-0.02527900	2.57546100	-0.80147600
C	0.91496800	3.92375100	1.36070600
H	1.33325500	4.08683700	2.35861600
H	0.96104500	4.87630300	0.81921600
H	-0.14067300	3.65425200	1.47675800
C	3.15383000	3.26767600	0.38787800
H	3.65729600	3.46176600	1.33955000
H	3.73026500	2.50088000	-0.14051200
H	3.19623100	4.19189200	-0.20055400
C	0.93347600	-0.61353700	-1.23530800
O	0.34583100	-0.88620300	-2.27831500
C	2.41020800	-0.96954300	-1.13571400
C	3.39697200	-0.07862800	-1.57845000
C	2.82328900	-2.25966500	-0.77657400
C	4.74277100	-0.43927800	-1.61149000
H	3.10311100	0.90895200	-1.91504700
C	4.16596300	-2.63500200	-0.79887400
C	5.13026300	-1.71758600	-1.21138800
H	5.48359900	0.27622400	-1.95634900
H	4.44329000	-3.64161700	-0.50462600
H	6.17630200	-2.00960500	-1.23098400
Cl	1.64440000	-3.48440600	-0.30875400

11k

B3LYP/6-31G*

SCF Done: E(RB3LYP) = -1805.58401730

Zero-point correction= 0.520044
Thermal correction to Gibbs Free Energy (423.15 K)= 0.412223

C	1.63271200	-3.80944500	0.98375300
C	1.27280300	-2.71100600	0.20292100
C	1.53816200	-1.40513000	0.64705000
C	2.17559600	-1.23480900	1.88262600
C	2.54254500	-2.33442900	2.66106600
C	2.27019200	-3.62670100	2.21346400
H	1.41591800	-4.81341800	0.62763200
H	0.77342000	-2.86031300	-0.74839700
H	2.39003100	-0.22962900	2.23767100
H	3.03779900	-2.17865800	3.61607900
H	2.55192100	-4.48592500	2.81671300
C	1.18017600	-0.17333100	-0.18231700
H	1.52729300	0.69721400	0.37722900
C	1.92892800	-0.13234500	-1.53692900
H	1.83716500	-1.08246500	-2.06092800
H	1.46314300	0.62740800	-2.17346700
C	3.38765800	0.22800800	-1.34072700
C	4.41519100	-0.60745000	-1.79465000
C	5.74060900	-0.21756100	-1.61222500
H	4.17249500	-1.54790300	-2.28020100
C	4.91691900	1.76065200	-0.55359300
C	6.00345600	0.99570500	-0.97861500
H	6.55315700	-0.85181900	-1.95690800
H	5.07729500	2.71412700	-0.05200400
H	7.01922300	1.34194700	-0.81309000
N	3.64185500	1.39635100	-0.72397600
N	-0.30017400	0.05288400	-0.30021100
C	-0.92323800	0.59957700	0.89087400
C	-1.42155700	-0.35994300	1.79067700
C	-0.99239500	1.98012800	1.20262000
C	-2.01599500	-0.00520400	2.99341600
H	-1.32503900	-1.40621200	1.52251400
C	-1.59225600	2.29509500	2.44075300
C	-2.09757800	1.34566800	3.32082800
H	-2.39753300	-0.77136100	3.66212300
H	-1.67420700	3.33820200	2.72658900

H	-2.55000100	1.66483000	4.25603900
C	-0.52692600	3.20783800	0.36632200
C	0.49658700	4.02567700	1.19677800
H	0.81549600	4.90684300	0.62711500
H	0.08603800	4.37749900	2.14788600
H	1.38841000	3.42709100	1.41315100
C	0.15557300	2.91703700	-0.98505400
H	-0.44805700	2.29384800	-1.64720000
H	0.32220000	3.87334400	-1.49567900
H	1.13608000	2.45273900	-0.86249300
C	-1.76618300	4.09400200	0.07179900
H	-2.26095300	4.44003500	0.98411700
H	-1.46387800	4.98005800	-0.49902200
H	-2.50957600	3.54682400	-0.51820500
C	-1.04183700	-0.65756200	-1.21833000
O	-0.54810100	-1.40886100	-2.05399800
C	-2.54427500	-0.44245500	-1.23879000
C	-3.07873900	0.76952100	-1.69512400
C	-3.43949100	-1.48706800	-0.96939200
C	-4.45129200	0.94178200	-1.86440900
H	-2.40343400	1.58319100	-1.93591500
C	-4.81605700	-1.32438100	-1.11917100
C	-5.32253900	-0.10642900	-1.57034700
H	-4.83610100	1.89092000	-2.22607900
H	-5.47862000	-2.15126600	-0.88667900
H	-6.39486900	0.01623900	-1.69311000
Cl	-2.85836800	-3.04822400	-0.39975600

6l

B3LYP/6-31G*

SCF Done: E(RB3LYP) = -1499.63609370

Zero-point correction= 0.576783
Thermal correction to Gibbs Free Energy (423.15 K)= 0.464714

C	-4.09950100	2.75156400	-0.04406000
C	-3.32192400	1.65833100	0.34419700
C	-2.59085800	0.91857000	-0.59598500
C	-2.66209300	1.29724800	-1.94619900
C	-3.43850200	2.38925500	-2.33442800
C	-4.16001000	3.12032800	-1.38758400
H	-4.65909700	3.30883100	0.70286900
H	-3.28827400	1.37030600	1.39254400
H	-2.08218400	0.74843300	-2.67936400
H	-3.47799900	2.67174800	-3.38354600
H	-4.76421700	3.96973700	-1.69574200
C	-1.78534000	-0.28477400	-0.11191200
H	-2.03456600	-0.41417900	0.94200400
C	-2.20296300	-1.61380400	-0.79361900
H	-2.20742900	-1.51518000	-1.87856600
H	-1.45663800	-2.37573800	-0.53891700
C	-3.55005000	-2.08325500	-0.28817800
C	-4.66480200	-2.17210500	-1.12992300
C	-5.87632800	-2.62246800	-0.60820100
H	-4.57811900	-1.88869000	-2.17451700
C	-4.77885700	-2.84144500	1.50441200
C	-5.93981600	-2.96840200	0.74021900
H	-6.75453700	-2.70008300	-1.24387800
H	-4.78611300	-3.09946900	2.56274900
H	-6.86046400	-3.32512300	1.19223300
N	-3.61117800	-2.41253400	1.01510200
N	-0.29533300	-0.08052700	-0.09079100
C	0.27690600	0.15814900	1.21596500
C	0.13536600	-0.91724400	2.11822400
C	0.89695200	1.36009200	1.63223100
C	0.62342900	-0.86589400	3.41437100
H	-0.37274800	-1.81182600	1.76932600
C	1.40442700	1.35854800	2.95204100
C	1.28410100	0.29023000	3.82967200
H	0.49947300	-1.71442200	4.08127700
H	1.90772400	2.24927900	3.31182500

H	1.69476600	0.36607900	4.83291700
C	1.07747400	2.70014600	0.86262500
C	0.51751300	2.76469200	-0.57064000
H	0.63765100	3.79170000	-0.93627400
H	1.06020000	2.11891200	-1.26281000
H	-0.54342100	2.51987100	-0.62074000
C	0.34626400	3.81187000	1.66234100
H	0.70977500	3.90559200	2.68997700
H	0.49228500	4.77994400	1.16863500
H	-0.73075800	3.61324400	1.70212500
C	2.58839700	3.03463200	0.77279800
H	3.06060800	3.13061300	1.75545300
H	3.12614600	2.26036100	0.21531500
H	2.72503700	3.98708000	0.24666600
C	0.43430500	-0.30014000	-1.23924600
O	-0.11965800	-0.39561100	-2.33694000
C	1.93780400	-0.44233000	-1.19947500
C	2.63046300	0.03486200	-2.35102400
C	2.64946900	-1.10844800	-0.21985500
C	3.98896500	-0.11676400	-2.47384900
H	2.05132200	0.51120000	-3.13445600
C	4.05288500	-1.30094000	-0.32929500
C	4.74411400	-0.78639000	-1.47248200
H	4.50424000	0.26481500	-3.35224700
H	2.15096900	-1.50899600	0.65473600
C	6.14816000	-0.97308100	-1.56784000
C	4.79371300	-1.98731600	0.67067300
C	6.83846600	-1.64101000	-0.58059000
H	7.91311900	-1.77840400	-0.66568500
C	6.15504100	-2.15356700	0.54943800
H	4.26505900	-2.37536900	1.53810600
H	6.71130000	-2.67819300	1.32149700
H	6.67073000	-0.58060500	-2.43709500

11l

B3LYP/6-31G*

SCF Done: E(RB3LYP) = -1499.63802799

Zero-point correction= 0.576683
Thermal correction to Gibbs Free Energy (423.15 K)= 0.465152

C	2.68997000	-3.66910100	1.33294100
C	2.11405900	-2.73653600	0.46997000
C	2.25261800	-1.35994600	0.71306500
C	2.98632700	-0.95101000	1.83428600
C	3.56884300	-1.88383600	2.69459800
C	3.42058600	-3.24800500	2.44678500
H	2.57025800	-4.73081000	1.13149100
H	1.54487500	-3.06820700	-0.39156800
H	3.10542200	0.11106100	2.03448500
H	4.13498300	-1.54242300	3.55747500
H	3.87075300	-3.97828100	3.11435100
C	1.65700900	-0.29862400	-0.21171000
H	1.95793700	0.67033900	0.19027100
C	2.24636900	-0.35763900	-1.64171500
H	2.23329800	-1.37552500	-2.02761400
H	1.61415200	0.23903100	-2.30862000
C	3.65411700	0.20346600	-1.67694500
C	4.72317700	-0.54344200	-2.18661200
C	5.99443500	0.02571200	-2.22723400
H	4.55399800	-1.55529200	-2.54259300
C	5.04406300	1.99252400	-1.25786800
C	6.16428700	1.32584300	-1.75454300
H	6.83740300	-0.53772100	-2.61879600
H	5.13247000	3.00869300	-0.87585200
H	7.13518100	1.81198700	-1.76471000
N	3.82003200	1.45603400	-1.21481100
N	0.15472300	-0.23841300	-0.16784200
C	-0.35433600	0.34942500	1.05402700
C	-0.68237800	-0.56737300	2.06853700
C	-0.44489300	1.74410000	1.29031700
C	-1.10093300	-0.15662000	3.32619600
H	-0.58434900	-1.62515600	1.84737700
C	-0.83673200	2.11817900	2.59310000
C	-1.16282600	1.20897500	3.59288800
H	-1.35174600	-0.89016900	4.08699000
H	-0.89637700	3.17388800	2.83483600

H	-1.46437700	1.57024600	4.57247000
C	-0.18545100	2.92039500	0.30293100
C	-1.42371200	3.85772700	0.30027300
H	-1.24430700	4.69955300	-0.37830000
H	-2.31819500	3.33118800	-0.05312300
H	-1.65122700	4.27422500	1.28514200
C	1.04843000	3.72190200	0.78975900
H	1.95686000	3.11191400	0.74040400
H	1.20219500	4.59693200	0.14631200
H	0.92899400	4.07871800	1.81812100
C	0.06859000	2.53919300	-1.17033000
H	1.03369200	2.05374600	-1.31173300
H	-0.71560800	1.90140800	-1.58626300
H	0.09085600	3.46204900	-1.76262100
C	-0.58644500	-1.14138800	-0.90864600
O	-0.04608900	-1.96654000	-1.64897900
C	-2.09557200	-1.12254700	-0.86006200
C	-2.73030800	-2.36047200	-1.17006500
C	-2.88013100	-0.00618300	-0.64289100
C	-4.09788200	-2.46045200	-1.22518900
H	-2.09981900	-3.21893300	-1.37166700
C	-4.29687000	-0.07256000	-0.71179700
C	-4.92478000	-1.32639800	-1.00112100
H	-4.56722500	-3.41493100	-1.45204600
H	-2.42879800	0.95113300	-0.41429000
C	-6.34192200	-1.38773700	-1.05959700
C	-5.11327000	1.07093500	-0.49646700
C	-7.10536000	-0.26172500	-0.84471400
H	-8.18936400	-0.32159500	-0.89312700
C	-6.48534600	0.97998600	-0.56045200
H	-4.63434600	2.02197100	-0.27532300
H	-7.09877000	1.86093900	-0.39212200
H	-6.81579400	-2.34162500	-1.27925300

6m

B3LYP/6-31G*

SCF Done: E(RB3LYP) = -1460.51565699

Zero-point correction= 0.562725
Thermal correction to Gibbs Free Energy (423.15 K)= 0.450576

C	-3.60517300	2.78660700	-1.09513000
C	-2.97320400	1.80285000	-0.33124300
C	-2.23519100	0.77670700	-0.93788100
C	-2.14910400	0.75406800	-2.33920400
C	-2.78034100	1.73626200	-3.10259700
C	-3.51048400	2.75530900	-2.48604900
H	-4.17319600	3.57140700	-0.60206700
H	-3.06101000	1.82791500	0.75264700
H	-1.55984100	-0.01959800	-2.81792300
H	-2.69896800	1.70624000	-4.18638500
H	-4.00107400	3.51770000	-3.08578700
C	-1.59590300	-0.28755000	-0.04861700
H	-1.94685500	-0.08721500	0.96451200
C	-2.08528600	-1.72408100	-0.36956200
H	-1.98553700	-1.94552300	-1.43156600
H	-1.44449800	-2.42989600	0.17223500
C	-3.51142600	-1.92828300	0.09353900
C	-4.55358500	-2.17114000	-0.80885200
C	-5.84507700	-2.36209500	-0.32070800
H	-4.34921900	-2.20545800	-1.87472400
C	-4.96051100	-2.04859700	1.87803700
C	-6.05881700	-2.30227100	1.05506400
H	-6.66906600	-2.55272300	-1.00343300
H	-5.08465000	-1.99183500	2.95883700
H	-7.04647500	-2.44541800	1.48312800
N	-3.71744300	-1.86546500	1.42188200
N	-0.10122900	-0.19288900	0.07915900
C	0.37613700	0.38278800	1.31611900
C	0.04690500	-0.35982300	2.47000000
C	1.08124700	1.60428100	1.43367700
C	0.42651200	0.03919900	3.74170600
H	-0.51890800	-1.27808800	2.34045300
C	1.47381500	1.95799100	2.74532000
C	1.16839800	1.21281300	3.87551100
H	0.15940600	-0.56023200	4.60741400
H	2.03672800	2.87432900	2.88564900

H	1.49973200	1.55280400	4.85304100
C	1.46599600	2.63611200	0.33443900
C	1.02817400	2.31823600	-1.10747400
H	1.28808000	3.17704100	-1.73836100
H	1.54650300	1.45261800	-1.52229100
H	-0.04656300	2.15908800	-1.19551100
C	0.80124800	3.99137900	0.69729000
H	1.09283300	4.35788400	1.68601800
H	1.08888900	4.75295200	-0.03726300
H	-0.29083600	3.90172300	0.67922700
C	3.00688700	2.80388700	0.31426900
H	3.40724800	3.15076300	1.27211800
H	3.49819800	1.85669000	0.06753800
H	3.28842600	3.54096900	-0.44750900
C	0.70009000	-0.79448900	-0.87104900
O	0.22850400	-1.15514100	-1.95296800
C	2.16777600	-1.03009200	-0.62127200
C	2.98885200	-1.02124000	-1.75783000
C	2.75538600	-1.37090100	0.61039800
C	4.35287100	-1.29319900	-1.68236600
H	2.53512100	-0.80502300	-2.71917900
C	4.10929900	-1.66645100	0.69697200
H	2.15765600	-1.42384800	1.51114500
C	4.92271000	-1.61866500	-0.44439100
H	4.95278900	-1.26036700	-2.58460500
H	4.56163900	-1.94093600	1.64475600
O	6.23883300	-1.90965700	-0.24786000
C	7.10873200	-1.88934900	-1.36973200
H	7.14781100	-0.89422200	-1.83170200
H	8.09711700	-2.14781600	-0.98562400
H	6.80894500	-2.62665700	-2.12576500

11m

B3LYP/6-31G*

SCF Done: E(RB3LYP) = -1460.51798548

Zero-point correction= 0.562715
Thermal correction to Gibbs Free Energy (423.15 K)= 0.451495

C	3.14450100	-1.77672000	2.90971700
C	2.66342900	-0.89969700	1.93549300
C	1.86433700	-1.35787400	0.87988200
C	1.55467500	-2.72678300	0.82102000
C	2.02953100	-3.60245000	1.79766300
C	2.82707400	-3.13292500	2.84422400
H	3.76404300	-1.39778700	3.71859600
H	2.91419800	0.15672800	1.99388400
H	0.93196400	-3.09395800	0.01257600
H	1.77767200	-4.65853900	1.73838500
H	3.19775900	-3.81966500	3.60097600
C	1.38575200	-0.36070600	-0.17553100
H	1.80758900	0.60826100	0.09774900
C	1.94427000	-0.67383300	-1.58493800
H	1.80155700	-1.72329500	-1.83676100
H	1.38027200	-0.09341400	-2.32351900
C	3.40898200	-0.29852800	-1.68918500
C	4.37463100	-1.23360900	-2.08137700
C	5.70509900	-0.83452000	-2.19200300
H	4.08035100	-2.25719400	-2.29316700
C	5.00986700	1.34922000	-1.51448500
C	6.03608000	0.48858900	-1.90485900
H	6.46960400	-1.54542100	-2.49462600
H	5.22469700	2.39048900	-1.27779600
H	7.05862400	0.84699700	-1.97603100
N	3.73029800	0.97686300	-1.40564700
N	-0.09631600	-0.11192500	-0.14465200
C	-0.51155700	0.67803900	0.99522000
C	-0.93443600	-0.05990600	2.11499700
C	-0.42745600	2.09175400	1.05807100
C	-1.27757500	0.55034100	3.31333300
H	-0.97187800	-1.14056800	2.02507000
C	-0.74652800	2.66806800	2.30561100
C	-1.16448700	1.93507800	3.41058300
H	-1.60422400	-0.04784100	4.15916400
H	-0.67245600	3.74470400	2.41498200

H	-1.40157700	2.44770600	4.33909000
C	-0.05244400	3.09669500	-0.07096000
C	0.12322200	2.50706900	-1.48584600
H	0.24392400	3.33977600	-2.18950900
H	1.01936200	1.89311700	-1.57068800
H	-0.74106400	1.92339800	-1.81284000
C	-1.17395000	4.16536700	-0.18379000
H	-1.32720800	4.72774100	0.74122200
H	-0.91408900	4.88709500	-0.96676000
H	-2.13192600	3.70802900	-0.45774000
C	1.27645500	3.80081800	0.30269500
H	1.22184200	4.29393700	1.27896100
H	2.10651800	3.08639300	0.32389700
H	1.51683300	4.56517400	-0.44654500
C	-0.95339000	-1.00490300	-0.76867500
O	-0.51931700	-1.98599900	-1.37878000
C	-2.44183800	-0.77854700	-0.74786500
C	-3.23341200	-1.91257200	-0.97631600
C	-3.09511800	0.45852300	-0.60699300
C	-4.62287100	-1.83914600	-1.03821800
H	-2.73533900	-2.86547900	-1.11730100
C	-4.47724800	0.55127800	-0.68447500
H	-2.52675100	1.36335600	-0.43985000
C	-5.25485200	-0.59739800	-0.89264400
H	-5.19656100	-2.74353000	-1.20577300
H	-4.98095500	1.50766900	-0.58537000
O	-6.60171500	-0.40077500	-0.94061600
C	-7.44016300	-1.52582500	-1.15767800
H	-7.33383800	-2.26842100	-0.35617800
H	-8.46145700	-1.14084900	-1.15970800
H	-7.23059100	-2.00436500	-2.12314000

6p

B3LYP/6-31G*

SCF Done: E(RB3LYP) = -1666.74917021

Zero-point correction= 0.496215
Thermal correction to Gibbs Free Energy (423.15 K)= 0.390890

C	-2.88758800	3.18021000	-0.27461800
C	-2.32073400	1.99950200	0.21058700
C	-1.75874600	1.05470100	-0.65956500
C	-1.78374300	1.31765500	-2.03872100
C	-2.34846300	2.49765200	-2.52347500
C	-2.90254200	3.43279300	-1.64600200
H	-3.31948300	3.89686300	0.41925900
H	-2.32382700	1.80385600	1.28053000
H	-1.33418000	0.60370400	-2.71953600
H	-2.35427700	2.68771500	-3.59391500
H	-3.34256200	4.34987500	-2.02920400
C	-1.18861100	-0.23349700	-0.07122000
H	-1.40460900	-0.20541600	0.99792900
C	-1.90515600	-1.50593000	-0.59460700
H	-1.92000200	-1.52762800	-1.68364200
H	-1.33471800	-2.38054800	-0.25912600
C	-3.30752100	-1.60957700	-0.03416500
C	-4.43596600	-1.56958800	-0.86150200
C	-5.70222100	-1.68269200	-0.29015100
H	-4.31671900	-1.44868200	-1.93398200
C	-4.62504300	-1.85231600	1.83729400
C	-5.80472800	-1.82947400	1.09185300
H	-6.59202800	-1.65492000	-0.91372700
H	-4.66101400	-1.96333400	2.92040900
H	-6.76869800	-1.92194200	1.58316800
N	-3.40529500	-1.74676700	1.30066100
N	0.31102200	-0.34090300	-0.11570000
C	0.97325600	-0.14159200	1.15328600
C	0.72351300	-1.15087700	2.10508000
C	1.76832600	0.97996400	1.49073300
C	1.24668600	-1.10322900	3.38867300
H	0.09769200	-1.98665200	1.80630100
C	2.28320300	0.98705400	2.80670400
C	2.04393000	-0.01399500	3.73922300
H	1.03697500	-1.89920500	4.09745100
H	2.89498200	1.82663000	3.11774500

H	2.47344900	0.06195100	4.73455300
C	2.13620200	2.22164600	0.62914900
C	1.68918600	2.20440200	-0.84479800
H	1.98252100	3.15824200	-1.29974000
H	2.18064800	1.41362400	-1.41660600
H	0.61061100	2.10426000	-0.96068600
C	1.48464400	3.46687200	1.28503400
H	1.78859100	3.59634800	2.32878800
H	1.77460500	4.37141600	0.73697400
H	0.39190000	3.39222500	1.25699000
C	3.67809200	2.39281200	0.61500900
H	4.10173700	2.55946400	1.60985900
H	4.16350900	1.50959600	0.18536500
H	3.94471000	3.25870200	-0.00226100
C	0.92687600	-0.82894300	-1.24663100
O	0.31178900	-0.93160100	-2.31144200
C	2.36663400	-1.26041600	-1.23486300
C	3.03835000	-1.37815900	-2.50244300
C	3.17143700	-1.66983300	-0.19539800
C	4.31108900	-1.84984300	-2.39831800
H	5.02990300	-2.02385600	-3.18752600
S	4.72771700	-2.18810900	-0.74495100
H	2.94722500	-1.71928700	0.85822800
H	2.55300700	-1.11856500	-3.43446600

11p

B3LYP/6-31G*

SCF Done: E(RB3LYP) = -1666.75138019

Zero-point correction= 0.496341
Thermal correction to Gibbs Free Energy (423.15 K)= 0.390783

C	2.83203500	-2.16926100	2.63669800
C	2.29238700	-1.15067300	1.84822600
C	1.59938100	-1.44236600	0.66654400
C	1.45637700	-2.78734300	0.28729500
C	1.98821700	-3.80539600	1.07874200
C	2.67923600	-3.50154200	2.25464800
H	3.36733800	-1.91939500	3.54920500
H	2.41333800	-0.11389400	2.15290900
H	0.92047100	-3.02648800	-0.62522800
H	1.86553600	-4.84151900	0.77301800
H	3.09509700	-4.29799700	2.86643500
C	1.04972400	-0.29465500	-0.17900800
H	1.29303200	0.63176100	0.34561500
C	1.75559100	-0.19003700	-1.55418500
H	1.15134000	0.43619900	-2.21882100
H	1.82978600	-1.16691600	-2.02957400
C	3.12858800	0.43745800	-1.41534900
C	4.27595800	-0.20666800	-1.89393700
C	5.51260300	0.42414900	-1.77304600
H	4.19332200	-1.18796300	-2.35158000
C	4.37729200	2.24444900	-0.71989700
C	5.57012200	1.68136400	-1.17419700
H	6.41507100	-0.05940500	-2.13788100
H	4.37721700	3.22372900	-0.24312400
H	6.50990400	2.21246400	-1.05668200
N	3.18560500	1.64828700	-0.83132000
N	-0.45019100	-0.26239300	-0.24735200
C	-1.06344200	0.23795600	0.96250900
C	-1.35784300	-0.73978200	1.92990600
C	-1.29350800	1.61011100	1.23238900
C	-1.87158800	-0.40964700	3.17642000
H	-1.16070700	-1.77640900	1.67783900
C	-1.78464800	1.90460600	2.52235900
C	-2.07287000	0.93570300	3.47736100
H	-2.09293700	-1.18705500	3.90196500
H	-1.95431400	2.94199600	2.78977600

H	-2.45488800	1.23525300	4.44969900
C	-1.09360900	2.82937000	0.28822200
C	-0.75415700	2.50103900	-1.17884500
H	-0.76052200	3.43733400	-1.75003600
H	0.24144100	2.07154600	-1.28521200
H	-1.48572300	1.82953100	-1.63632400
C	-2.40507700	3.65959600	0.25141300
H	-3.23528300	3.06542500	-0.14793300
H	-2.26994800	4.52635900	-0.40563800
H	-2.70452000	4.03854800	1.23247000
C	0.05161800	3.71119600	0.84629800
H	-0.13469100	4.02366700	1.87938500
H	0.15604400	4.61637100	0.23551000
H	1.00769300	3.17731900	0.81431100
C	-1.12192700	-1.05065000	-1.15919400
O	-0.51011300	-1.76618700	-1.95798000
C	-2.62208200	-1.02373500	-1.26062500
C	-3.23293000	-1.89449900	-2.23091400
C	-3.56579500	-0.27094000	-0.59643300
C	-4.58862100	-1.78593300	-2.28030600
H	-5.28292600	-2.32050800	-2.91433800
S	-5.17137300	-0.61026800	-1.14027000
H	-3.42676800	0.45629800	0.18657900
H	-2.64461400	-2.55694400	-2.85203800

6r

B3LYP/6-31G*

SCF Done: E(RB3LYP) = -1001.84174411

Zero-point correction= 0.452678
Thermal correction to Gibbs Free Energy (423.15 K)= 0.361033

C	0.84288300	0.30898600	-0.42983800
H	0.80275600	-0.78242100	-0.40079400
C	1.92683900	0.73025600	0.59512700
H	2.02130700	1.81875400	0.59560300
H	1.59700500	0.41590100	1.59226600
C	3.26460900	0.07792500	0.32499000
C	4.41379900	0.83573300	0.06375800
C	5.62350600	0.18198100	-0.16354600
H	4.35348200	1.91996000	0.04122700
C	4.45562800	-1.88239600	0.13286900
C	5.65096300	-1.21106200	-0.12852400
H	6.52743400	0.75103500	-0.36518100
H	4.43306200	-2.97107200	0.16711300
H	6.56884900	-1.76566600	-0.29916800
N	3.28996400	-1.26726500	0.35630200
N	-0.52225400	0.75410400	-0.01082300
C	-1.31523600	-0.19547600	0.74428500
C	-1.12738100	-0.16308600	2.13574400
C	-2.22374900	-1.12619600	0.16733600
C	-1.79219800	-1.02925600	2.99640900
H	-0.43903700	0.57352200	2.53854800
C	-2.87138100	-1.99004900	1.07142700
C	-2.67067800	-1.95723700	2.44987600
H	-1.62186200	-0.97706000	4.06801100
H	-3.57167400	-2.72177700	0.69091700
H	-3.20809800	-2.65494300	3.08658100
C	-2.52730600	-1.28345100	-1.34890100
C	-1.36786600	-2.04886000	-2.03195400
H	-1.60538900	-2.22359200	-3.08874500
H	-0.42576600	-1.50064900	-1.99086300
H	-1.21178900	-3.02337800	-1.55536900
C	-3.81287400	-2.11127800	-1.59271500
H	-4.67967300	-1.68628500	-1.07407000
H	-4.03437000	-2.11227800	-2.66539200
H	-3.70699500	-3.15812900	-1.28957500
C	-2.75513400	0.07167100	-2.05815100

H	-3.60186500	0.60866300	-1.61458100
H	-1.88055400	0.71930500	-2.02055300
H	-2.99630900	-0.10699500	-3.11275800
C	-0.79663900	2.11049900	0.06327700
O	0.01078300	2.95664600	-0.31262200
C	1.19992500	0.74768600	-1.85709200
H	0.41304900	0.46959800	-2.56456600
H	1.34329200	1.82736400	-1.91142200
H	2.12288300	0.24784100	-2.16969800
C	-2.16094300	2.53074700	0.61836400
H	-2.92418000	1.78887600	0.36638700
H	-2.09355900	2.52192500	1.71493300
C	-2.56423600	3.92454600	0.13366000
H	-1.79259000	4.65774600	0.37984000
H	-2.69763100	3.94104500	-0.95328600
H	-3.50720400	4.23157200	0.59882600

11r

B3LYP/6-31G*

SCF Done: E(RB3LYP) = -1001.84327196

Zero-point correction= 0.452513
Thermal correction to Gibbs Free Energy (423.15 K)= 0.361096

C	0.93228700	-0.24589200	0.21826700
H	1.29328800	0.37633700	1.04252000
C	2.00899600	-0.19725200	-0.90553100
H	1.85540100	-1.02587900	-1.60454200
H	1.89824100	0.74607900	-1.44666600
C	3.41401700	-0.27941300	-0.35414800
C	4.00238000	0.85071500	0.23601700
C	5.29164000	0.74986000	0.74990200
H	3.44087700	1.77971900	0.28339500
C	5.28796800	-1.54246700	0.06672900
C	5.95635900	-0.47453100	0.66302100
H	5.77158900	1.61110700	1.20774500
H	5.77058900	-2.51540300	-0.01792100
H	6.96460300	-0.60089400	1.04642700
N	4.04705300	-1.46205300	-0.43247100
N	-0.33540500	0.40349100	-0.22050000
C	-1.43959400	-0.38578500	-0.70160400
C	-1.33034500	-0.84363200	-2.02722300
C	-2.57243000	-0.71946400	0.07996100
C	-2.30587200	-1.63681600	-2.61633000
H	-0.44759600	-0.55520300	-2.59014700
C	-3.54294600	-1.52306500	-0.55515500
C	-3.42728400	-1.97937900	-1.86315800
H	-2.19301200	-1.97746400	-3.64162600
H	-4.42977800	-1.80810200	0.00107000
H	-4.21250300	-2.59813500	-2.28916300
C	-2.88872700	-0.31166300	1.54802000
C	-4.24094300	0.44866600	1.57552800
H	-4.48881900	0.72879100	2.60618400
H	-4.18920000	1.36891600	0.98229400
H	-5.06976700	-0.15086000	1.18777000
C	-3.02192700	-1.59949600	2.40201600
H	-2.08016200	-2.15872500	2.42286000
H	-3.28153400	-1.33750800	3.43461900
H	-3.79849600	-2.27193300	2.02520900
C	-1.86155000	0.59542500	2.25885800

H	-0.86749900	0.14952000	2.32155500
H	-1.76423600	1.57683400	1.78910900
H	-2.21055800	0.76327100	3.28494700
C	-0.29662000	1.77583000	-0.36376800
O	0.69014400	2.42423500	-0.01679300
C	0.73438600	-1.67651500	0.71823900
H	0.06774100	-1.72424500	1.58396800
H	0.33073000	-2.32839100	-0.06262300
H	1.70800000	-2.07989100	1.01235500
C	-1.51113000	2.46073100	-0.99218000
H	-1.57996400	2.13428100	-2.03901200
H	-2.43200700	2.10043000	-0.52088400
C	-1.41831200	3.98413700	-0.91175300
H	-1.38645800	4.32315200	0.12839300
H	-0.50908000	4.34667600	-1.39812500
H	-2.28518500	4.44361000	-1.39882100

6w

B3LYP/6-31G*

SCF Done: E(RB3LYP) = -1154.25840066

Zero-point correction= 0.476430
Thermal correction to Gibbs Free Energy (423.15 K)= 0.380444

C	-2.53590300	-0.43124300	-0.52374400
C	-3.48163600	-1.02815400	-1.38635600
C	-3.20369000	-2.11830200	-2.20185900
C	-1.92795400	-2.67921000	-2.18813000
C	-0.96418000	-2.11686400	-1.36282000
C	-1.24154800	-1.00585600	-0.54473000
H	-4.48263700	-0.61269200	-1.42704400
H	-3.98261100	-2.52406300	-2.84224300
H	-1.68574800	-3.53713700	-2.80951200
H	0.04667000	-2.51326200	-1.32587800
N	-0.10644700	-0.50174800	0.20391900
C	0.19579800	-0.95521100	1.47293400
O	1.19654200	-0.56577600	2.06853900
C	-0.73550700	-1.99609900	2.09207500
H	-1.78243400	-1.73019000	1.91644300
H	-0.57804200	-2.93320000	1.53977100
C	-0.46373900	-2.19992100	3.58212900
H	0.57971300	-2.47595400	3.75301700
H	-0.65480200	-1.28211300	4.14801200
H	-1.10851300	-2.99025600	3.98157300
C	0.96258500	0.17426800	-0.58979600
H	0.63044200	0.04035000	-1.62445200
C	1.05494000	1.68257700	-0.37328700
C	1.44885600	2.25286400	0.84880300
C	0.74771400	2.53536400	-1.44190400
C	1.52248600	3.63878100	0.98842900
H	1.68380300	1.59713200	1.67980400
C	0.82219500	3.92343600	-1.30196700
H	0.44826300	2.10981300	-2.39761900
C	1.21008300	4.47923100	-0.08348000
H	1.82587700	4.06453700	1.94152400
H	0.57813200	4.56518800	-2.14453100
H	1.26912700	5.55838400	0.03168100
C	2.31110600	-0.56862100	-0.51400200
C	3.53760000	0.10447000	-0.49070000
N	2.24004300	-1.90637400	-0.58435500

C	4.71667300	-0.63837000	-0.51264700
H	3.57032400	1.18606700	-0.44654500
C	3.37620700	-2.60892700	-0.59886800
C	4.64308800	-2.02828600	-0.56205700
H	5.67897000	-0.13372900	-0.48724800
H	3.26583300	-3.69138800	-0.64710000
H	5.53533200	-2.64677300	-0.57547100
C	-3.05407800	0.78410400	0.29759900
C	-3.35907500	1.93850900	-0.69087200
H	-4.07490100	1.64093000	-1.46421600
H	-3.78291700	2.79413900	-0.15103900
H	-2.44309000	2.27541400	-1.18868300
C	-4.36022300	0.38631800	1.03539800
H	-5.16847200	0.09664800	0.35810500
H	-4.18456000	-0.44972700	1.72250900
H	-4.71991000	1.23607400	1.62722500
C	-2.10732300	1.34555900	1.37628600
H	-2.61139300	2.18583100	1.86921000
H	-1.87562600	0.61147400	2.15337400
H	-1.17120700	1.71905300	0.96547700

11w

B3LYP/6-31G*

SCF Done: E(RB3LYP) = -1154.25913607

Zero-point correction= 0.476380
Thermal correction to Gibbs Free Energy (423.15 K)= 0.380625

C	-2.28866900	-0.15943400	0.50085200
C	-3.10725700	0.12919600	1.61371200
C	-2.65614700	0.10150000	2.92789100
C	-1.32797400	-0.22718600	3.19282900
C	-0.48734500	-0.51043900	2.12487100
C	-0.93922600	-0.47371300	0.79393400
H	-4.14733600	0.38638500	1.44307400
H	-3.34299900	0.33354800	3.73778000
H	-0.95198200	-0.26115300	4.21182000
H	0.56007700	-0.74908400	2.28018400
N	0.04673700	-0.80450200	-0.20769600
C	0.25549700	-2.10597900	-0.62500100
O	1.10923300	-2.36471300	-1.46952200
C	-0.59487600	-3.20391800	0.01294000
H	-0.35153900	-3.24146200	1.08311000
H	-1.65469500	-2.92914500	-0.02935700
C	-0.35848700	-4.56459000	-0.64154500
H	-0.62948600	-4.54539200	-1.70169600
H	0.69530600	-4.84921700	-0.58220200
H	-0.95917000	-5.33413200	-0.14449500
C	1.03122600	0.17722600	-0.74087100
H	1.04732200	0.00955900	-1.82167500
C	0.66680300	1.64171200	-0.51303200
C	0.27561200	2.42433700	-1.60801300
C	0.78444900	2.26124200	0.74010700
C	-0.01899600	3.78115200	-1.45692700
H	0.20798300	1.97010400	-2.59363800
C	0.48628700	3.61439500	0.89468300
H	1.12215700	1.68209400	1.59169000
C	0.08014000	4.37929100	-0.20099400
H	-0.31872300	4.36760600	-2.32149500
H	0.57871000	4.07459900	1.87494700
H	-0.14748100	5.43481700	-0.07773100
C	2.45694100	-0.10921200	-0.25203300
C	3.52434800	-0.03413800	-1.15522900
N	2.63944900	-0.38118300	1.04618300

C	4.81884100	-0.24448900	-0.69272000
H	3.33364800	0.17023000	-2.20434400
C	3.88967900	-0.58753200	1.48134100
C	5.01245000	-0.52881100	0.65982100
H	5.66201300	-0.19847100	-1.37686100
H	3.99294700	-0.81039100	2.54226600
H	6.00330600	-0.70589900	1.06689600
C	-2.99453500	-0.09537200	-0.88342500
C	-3.50800800	1.35045400	-1.10831300
H	-2.67526800	2.06066800	-1.13640400
H	-4.04244200	1.41222300	-2.06435300
H	-4.19753300	1.67287800	-0.32197400
C	-2.14345400	-0.46760900	-2.11455300
H	-2.76874400	-0.35351600	-3.00855100
H	-1.27822800	0.18435200	-2.23725400
H	-1.79488700	-1.50292500	-2.09652000
C	-4.20446700	-1.06662500	-0.87733700
H	-4.72078500	-1.02182600	-1.84371200
H	-3.87964200	-2.10173900	-0.71948400
H	-4.93668000	-0.82596100	-0.10118300

6za

B3LYP/6-31G*

SCF Done: E(RB3LYP) = -1306.67593545

Zero-point correction= 0.501195

Thermal correction to Gibbs Free Energy (423.15 K)= 0.400389

C	1.78336100	1.31044200	1.07075800
C	2.61810100	1.54742100	2.18740600
C	2.55252400	0.82747500	3.37221900
C	1.61896000	-0.20134800	3.49822700
C	0.79369000	-0.48212100	2.42029100
C	0.86655500	0.24246500	1.21294900
H	3.34879600	2.34653700	2.12527200
H	3.22378700	1.07317500	4.19094000
H	1.54327400	-0.78219200	4.41348100
H	0.07452100	-1.29597000	2.46293900
N	-0.09101900	-0.17592800	0.20952900
C	0.20067700	-0.95946200	-0.88776000
O	-0.67715700	-1.24439400	-1.70375700
C	-1.53272700	0.03168100	0.55716700
H	-1.49253900	0.32755700	1.60942200
C	-2.19983700	1.19846000	-0.16753600
C	-2.56157000	2.32556300	0.58341700
C	-2.47503300	1.19633000	-1.54550400
C	-3.17680800	3.42693800	-0.01596600
H	-2.35992800	2.34347500	1.65249300
C	-3.09079000	2.29580400	-2.14370800
H	-2.18764800	0.33347500	-2.13471000
C	-3.44388000	3.41426500	-1.38436400
H	-3.44648600	4.28965500	0.58761000
H	-3.29411900	2.27845400	-3.21146300
H	-3.92253800	4.26811700	-1.85677100
C	-2.32424600	-1.28784100	0.56013600
C	-3.63340800	-1.39021000	0.07872500
N	-1.70774600	-2.31570800	1.16325200
C	-4.30385600	-2.60592000	0.20193600
H	-4.11479400	-0.54109800	-0.39043500
C	-2.35744300	-3.47811800	1.27029600
C	-3.65570700	-3.68150500	0.80446400
H	-5.31872500	-2.70877400	-0.17326000
H	-1.81233300	-4.28491600	1.75817000
H	-4.13792200	-4.64784400	0.91620400

C	2.00064400	2.27828100	-0.12695600
C	3.46796400	2.15577200	-0.61268900
H	3.67849500	1.13880100	-0.96075400
H	3.63897600	2.84307100	-1.45004300
H	4.19430400	2.40089900	0.16843000
C	1.10266200	2.07251000	-1.36050500
H	1.32784100	2.86729600	-2.08216800
H	1.29461600	1.12320500	-1.86308800
H	0.04108300	2.13553500	-1.12252300
C	1.73643700	3.72571300	0.36663800
H	1.92322100	4.43530400	-0.44821500
H	0.69303400	3.84178600	0.68136300
H	2.37531500	4.01114900	1.20771700
C	1.59876500	-1.47373400	-1.13371300
C	2.00046000	-1.53508800	-2.47848200
C	2.45103800	-1.99505400	-0.15031000
C	3.24228700	-2.05805800	-2.82878500
H	1.31694500	-1.17588400	-3.24071000
C	3.68590500	-2.54085200	-0.50621800
H	2.15237100	-1.99440600	0.88997900
C	4.09142600	-2.56267200	-1.84074400
H	3.54282800	-2.08292800	-3.87285700
H	4.33098000	-2.95071300	0.26641500
H	5.05835200	-2.97897300	-2.11118100

11za

B3LYP/6-31G*

SCF Done: E(RB3LYP) = -1306.67662533

Zero-point correction= 0.501102
Thermal correction to Gibbs Free Energy (423.15 K)= 0.400690

C	1.18663500	-1.75673300	0.44432600
C	1.53260200	-2.56319800	1.55128800
C	1.15380200	-2.28428500	2.85785700
C	0.38712200	-1.15036100	3.12358400
C	0.04072700	-0.32112200	2.06674100
C	0.43461000	-0.59627800	0.74416700
H	2.12366400	-3.45616000	1.37845200
H	1.45413600	-2.95292900	3.66046300
H	0.07239500	-0.91089700	4.13578800
H	-0.54421600	0.57980600	2.22561700
N	0.01643000	0.37937300	-0.23504300
C	0.83690200	1.35208200	-0.77605100
O	0.41710200	2.08144200	-1.67651100
C	-1.38946000	0.43806700	-0.74296600
H	-1.29004200	0.53763000	-1.82700400
C	-2.22160200	-0.81550700	-0.48279200
C	-2.83447800	-1.07893800	0.75089000
C	-2.45145800	-1.70578200	-1.54122800
C	-3.62248700	-2.21602500	0.92604100
H	-2.70320700	-0.38275500	1.57079900
C	-3.24067800	-2.84466200	-1.36941400
H	-2.01731100	-1.50099000	-2.51659300
C	-3.82513200	-3.10613800	-0.13033200
H	-4.08521400	-2.40324900	1.89149800
H	-3.40452000	-3.51853000	-2.20623500
H	-4.44224600	-3.98988200	0.00861500
C	-2.11290700	1.70387500	-0.27184400
C	-2.84405800	2.46514500	-1.19159300
N	-2.04738100	2.02545700	1.02699700
C	-3.53193900	3.58892500	-0.74571700
H	-2.85359900	2.18510200	-2.24029200
C	-2.70829800	3.11265200	1.44597200
C	-3.46569800	3.92595400	0.60665600
H	-4.10020100	4.19879300	-1.44296500
H	-2.62440200	3.34036900	2.50749200
H	-3.97998300	4.79718200	1.00078400

C	1.68192700	-2.27859700	-0.93432800
C	1.29815400	-1.44641500	-2.17396200
H	1.77974500	-0.46800800	-2.19283100
H	1.63718300	-1.98686500	-3.06612500
H	0.22022200	-1.30751900	-2.26704500
C	3.22990100	-2.36233200	-0.91461200
H	3.59344400	-2.74736900	-1.87522600
H	3.67207000	-1.37293900	-0.75655700
H	3.60365700	-3.02731500	-0.12989300
C	1.09160000	-3.69642300	-1.15819900
H	1.44966400	-4.10072500	-2.11280200
H	1.37931900	-4.40169200	-0.37303500
H	-0.00279900	-3.66105200	-1.19167400
C	2.26336400	1.52914400	-0.31243600
C	3.18376200	1.89863200	-1.30776400
C	2.69919100	1.46894600	1.01867000
C	4.51527000	2.15306800	-0.99000200
H	2.83104100	1.99476100	-2.32925500
C	4.02922900	1.74830300	1.33832700
H	2.00654600	1.21877500	1.81163500
C	4.94341200	2.07767900	0.33766600
H	5.21587600	2.42232400	-1.77597300
H	4.34838000	1.70685800	2.37636100
H	5.98014700	2.28388100	0.59074100

6zb

B3LYP/6-31G*

SCF Done: E(RB3LYP) = -1114.94218734

Zero-point correction= 0.448351
Thermal correction to Gibbs Free Energy (423.15 K)= 0.356307

C	0.57750400	1.93389900	0.47247400
C	0.67371000	2.57483300	1.72509200
C	0.41309100	1.94582600	2.93706400
C	0.02484100	0.60869400	2.94625800
C	-0.11773000	-0.04756000	1.73192100
C	0.14663500	0.58430800	0.50394400
H	0.97287800	3.61689700	1.75443600
H	0.51476100	2.50125500	3.86551000
H	-0.18339100	0.08987200	3.87757300
H	-0.45679000	-1.07832000	1.71038400
N	-0.12951600	-0.21598800	-0.67263100
C	0.47359100	-1.45190500	-0.84932700
O	-0.07655800	-2.35797500	-1.47814600
C	-1.47868000	0.04959500	-1.27556300
H	-1.57810100	1.13532200	-1.22425300
C	-2.64485300	-0.46757600	-0.43074400
C	-2.85795400	-1.83112400	-0.17733000
N	-3.47660000	0.48581400	0.02031900
C	-3.96428500	-2.20608700	0.58110900
H	-2.16439100	-2.56403100	-0.57343000
C	-4.53921500	0.10820800	0.74198300
C	-4.82836400	-1.21866500	1.05521900
H	-4.15172700	-3.25549400	0.79420900
H	-5.18883300	0.91156500	1.08688000
H	-5.70362600	-1.46729800	1.64826400
C	0.96213000	2.80580800	-0.75832200
C	2.36176100	3.43525400	-0.52064900
H	3.13197700	2.66188900	-0.41832100
H	2.63183400	4.06342100	-1.37739700
H	2.40372900	4.06528900	0.37160600
C	1.05029800	2.07909900	-2.11628000
H	1.72660800	1.22087800	-2.09659600
H	0.07935000	1.74038100	-2.47505800
H	1.43672100	2.78660600	-2.85966100
C	-0.08110000	3.94260100	-0.90226000
H	0.17574000	4.58543900	-1.75318200

H	-1.08522000	3.53862700	-1.07346400
H	-0.12613300	4.57225300	-0.00778100
C	-1.61823300	-0.36117900	-2.75093000
H	-1.72623200	-1.43727500	-2.87168900
H	-2.50565300	0.13668000	-3.15792000
H	-0.74855000	-0.04148100	-3.33279600
C	1.85507100	-1.70819400	-0.29366400
C	2.17274000	-3.05301000	-0.04468800
C	2.84620700	-0.73630800	-0.09948700
C	3.43754500	-3.41446900	0.41155100
H	1.41269400	-3.80455300	-0.22801200
C	4.11920300	-1.10060900	0.33930200
H	2.63351100	0.30693100	-0.29129700
C	4.41715000	-2.43776800	0.60415600
H	3.66181400	-4.45925900	0.60932500
H	4.87847500	-0.33509500	0.47516200
H	5.40744100	-2.71751100	0.95390200

11zb

B3LYP/6-31G*

SCF Done: E(RB3LYP) = -1114.94698953

Zero-point correction= 0.448356
Thermal correction to Gibbs Free Energy (423.15 K)= 0.356421

C	1.73031800	-1.44417500	0.16749400
C	2.48973700	-2.05562700	1.18910100
C	2.01289200	-2.27484100	2.47538200
C	0.71400600	-1.88679200	2.80335900
C	-0.05010500	-1.24109500	1.84160900
C	0.44669800	-0.98580800	0.54978700
H	3.49822900	-2.38481200	0.96248800
H	2.65030300	-2.75846000	3.21102700
H	0.30859500	-2.06862400	3.79495500
H	-1.06229600	-0.91020900	2.05563600
N	-0.44164500	-0.25367700	-0.31898400
C	-0.26928400	1.06821000	-0.68114400
O	-0.98420900	1.56885500	-1.55178400
C	-1.63126300	-0.91237500	-0.93238600
H	-1.58343700	-0.67462200	-1.99935400
C	-2.93805300	-0.32550100	-0.39731600
C	-3.84282600	0.30768700	-1.25590400
N	-3.18490400	-0.48410200	0.91194400
C	-5.04867500	0.77706100	-0.74087100
H	-3.58693800	0.44702300	-2.30082500
C	-4.34144200	-0.02033200	1.39905100
C	-5.30985000	0.60989200	0.61856800
H	-5.76650800	1.27507400	-1.38729000
H	-4.50095800	-0.16348400	2.46686200
H	-6.23372500	0.96289100	1.06691400
C	2.40971800	-1.38271400	-1.23064900
C	2.85597500	-2.81813600	-1.62184000
H	1.99407400	-3.49242800	-1.68898900
H	3.34488100	-2.79792100	-2.60289300
H	3.56434400	-3.25428800	-0.91240800
C	1.53897600	-0.88169300	-2.40229200
H	0.61651900	-1.45874000	-2.51298500
H	1.27789700	0.17415800	-2.33057200
H	2.11119100	-1.00578100	-3.32952000
C	3.65792400	-0.46924400	-1.14938200
H	4.17211700	-0.45062000	-2.11829400

H	3.37762500	0.55742600	-0.89377600
H	4.37453500	-0.81938700	-0.39921300
C	-1.62739600	-2.43534900	-0.76618700
H	-1.76186100	-2.72816900	0.27728400
H	-2.46064100	-2.84812700	-1.34493500
H	-0.69997500	-2.87999100	-1.13921800
C	0.79763100	1.92372700	-0.04317300
C	1.39408500	2.88362000	-0.87749300
C	1.13726400	1.90719100	1.31693900
C	2.34178800	3.77282400	-0.37710100
H	1.08999300	2.92641100	-1.91809700
C	2.06974000	2.81520700	1.82144900
H	0.66985000	1.19934100	1.98961900
C	2.68374200	3.74044200	0.97712700
H	2.80429500	4.49895400	-1.04035800
H	2.31500000	2.79627800	2.87999600
H	3.41678900	4.43866000	1.37272800

9b

B3LYP/6-31G*

SCF Done: E(RB3LYP) = -1159.07461542

Zero-point correction= 0.567504
Thermal correction to Gibbs Free Energy= 0.505891
Thermal correction to Gibbs Free Energy (423.15 K)= 0.462707

C	0.47163200	-1.12961900	-0.50333300
H	-0.09170900	-0.23418800	-0.23147800
C	-0.03684500	-2.28019200	0.39201500
H	0.51573800	-3.19247300	0.14715800
H	0.18972400	-2.04424200	1.43771300
N	-2.39988800	-1.40312700	0.43361100
N	1.89776400	-0.78184900	-0.19768000
C	2.11619500	0.27358200	0.77001600
C	2.16273800	-0.13837900	2.11243700
C	2.26283700	1.65047500	0.44093900
C	2.33093700	0.75923300	3.16125200
H	2.07038700	-1.19974700	2.32086200
C	2.42340500	2.52750200	1.53105000
C	2.45544300	2.11039600	2.86020300
H	2.36287900	0.40509100	4.18779900
H	2.53620400	3.58691300	1.34260400
H	2.58507400	2.84560300	3.64991700
C	2.23381700	2.25202500	-0.99223900
C	3.10891200	1.45805800	-1.98984000
H	4.15670100	1.44520100	-1.66746200
H	3.07365200	1.94552100	-2.97137600
H	2.77563200	0.42894000	-2.11571300
C	0.77386700	2.31231700	-1.50365900
H	0.74089600	2.80640400	-2.48266900
H	0.14393700	2.88676200	-0.81471500
H	0.33218600	1.32171600	-1.61888900
C	2.77332800	3.70302400	-1.01523400
H	2.81996400	4.04604300	-2.05437100
H	2.12639900	4.40439600	-0.47784600
H	3.78354300	3.77263300	-0.59631600
C	2.88955000	-1.71299900	-0.46007600
O	2.65156000	-2.76178500	-1.05326000
C	0.23456300	-1.39937500	-1.99677300
H	0.71626800	-0.63680900	-2.61603600
H	0.62917500	-2.37241100	-2.29193600

H	-0.84004800	-1.36431700	-2.20483700
C	-1.53639000	-2.57440200	0.24597600
H	-1.72916500	-2.96524300	-0.75937800
H	-1.80268500	-3.38725500	0.95210400
C	-3.77493700	-1.68378700	0.01091800
H	-4.27830200	-2.39533100	0.69583700
H	-3.71691800	-2.18681000	-0.96370900
C	-2.36194800	-0.90135700	1.80518500
H	-3.02903300	-0.04041800	1.89769900
H	-1.35237900	-0.57110300	2.06465900
H	-2.67741300	-1.66438000	2.54429400
C	-4.63238500	-0.43912500	-0.13243300
C	-5.93730000	-0.40837300	0.37091400
C	-4.14783100	0.68661400	-0.81298600
C	-6.74793800	0.71521100	0.19245400
H	-6.32302900	-1.27262600	0.90774600
C	-4.95190300	1.81199200	-0.98804300
H	-3.12954900	0.67300800	-1.19094900
C	-6.25689000	1.82935700	-0.48803900
H	-7.75930100	0.72051300	0.59088800
H	-4.56108700	2.67756800	-1.51723000
H	-6.88372100	2.70652300	-0.62581100
C	4.31348800	-1.38562800	0.00031800
H	4.39004900	-1.64873800	1.06435700
H	4.49609700	-0.30819500	-0.04808300
C	5.36030800	-2.15923700	-0.80320800
H	6.36380800	-1.96349100	-0.41009000
H	5.34299500	-1.86649900	-1.85842500
H	5.16551900	-3.23332600	-0.75863900

17b

B3LYP/6-31G*

SCF Done: E(RB3LYP) = -1159.07680186

Zero-point correction= 0.567428
Thermal correction to Gibbs Free Energy= 0.506867
Thermal correction to Gibbs Free Energy (423.15 K)= 0.464175

C	-0.08815200	0.41864000	0.18714800
H	-0.44023100	0.76919600	-0.78602500
C	-0.78637500	1.26909300	1.27136500
H	-0.53123000	0.87460100	2.26451800
H	-0.38984600	2.28656900	1.22172800
N	-2.83716800	1.68288500	-0.14315600
N	1.38796200	0.67115200	0.18840800
C	2.28666300	-0.24460200	0.84244500
C	2.37780200	-0.11932200	2.24064000
C	3.03973800	-1.23467800	0.16397600
C	3.18282000	-0.95210400	3.00655200
H	1.79529900	0.66415100	2.71568300
C	3.84768200	-2.05845000	0.97603600
C	3.92572700	-1.93700900	2.35870800
H	3.23105100	-0.83130000	4.08510300
H	4.44305500	-2.83323500	0.50446000
H	4.56777300	-2.60795500	2.92313800
C	3.10025800	-1.52694800	-1.36391400
C	2.63884800	-2.98723800	-1.61047300
H	1.59685400	-3.13069900	-1.30431000
H	3.24737200	-3.71555700	-1.06600100
H	2.71157500	-3.22580900	-2.67822700
C	2.24945700	-0.62471000	-2.28359200
H	2.56619000	0.42046500	-2.26523100
H	2.37259900	-0.97957000	-3.31405500
H	1.18360700	-0.65906100	-2.05197700
C	4.56944800	-1.38225400	-1.84157600
H	4.93689500	-0.36132100	-1.68682800
H	5.25036700	-2.06321300	-1.32245900
H	4.63468800	-1.60207100	-2.91377800
C	1.81242900	1.89858200	-0.27367200
O	1.03149100	2.69651000	-0.79112800
C	-0.42566300	-1.06634300	0.32751200
H	0.07685800	-1.67499800	-0.42881100
H	-0.15290500	-1.45934400	1.31283900

H	-1.50266800	-1.20023800	0.19277100
C	-2.32001900	1.30417800	1.17517000
H	-2.72735900	0.31626300	1.41386800
H	-2.69699000	1.99320100	1.96017600
C	-4.29051300	1.50499900	-0.20855800
H	-4.80919900	2.05040600	0.60566000
H	-4.62730900	1.96459700	-1.14698000
C	-2.45526600	3.04306000	-0.52221300
H	-2.82087600	3.80612700	0.19365200
H	-2.87784600	3.27405800	-1.50592800
H	-1.36872000	3.12436000	-0.59924500
C	-4.72615300	0.05129000	-0.19559600
C	-4.17077000	-0.86045900	-1.10540900
C	-5.71331200	-0.40001100	0.68647100
C	-4.59469900	-2.18851400	-1.13076700
H	-3.39808600	-0.51575400	-1.78706400
C	-6.14377500	-1.72928500	0.66175000
H	-6.14959100	0.29602300	1.39991300
C	-5.58505300	-2.62749500	-0.24707800
H	-4.15572200	-2.88240400	-1.84339700
H	-6.91159200	-2.06149800	1.35586400
H	-5.91650800	-3.66240200	-0.26784600
C	3.29169600	2.25154200	-0.10000700
H	3.91989200	1.42801600	-0.45645600
H	3.49972600	2.32323100	0.97635500
C	3.65817900	3.55755700	-0.80412600
H	4.71386600	3.79646200	-0.63531500
H	3.04744000	4.38572600	-0.43547800
H	3.48810100	3.48551300	-1.88281700

A

B3LYP/6-31G*

SCF Done: E(RB3LYP) = -1002.21018751

Zero-point correction=   0.465534
Thermal correction to Gibbs Free Energy=   0.411531
Thermal correction to Gibbs Free Energy (353.15 K)=   0.396125

C	-3.19950900	0.26674500	-1.43659500
C	-4.11387300	-0.76263800	-1.48412900
C	-4.30983700	-1.53249600	-0.33451100
C	-3.58049600	-1.23245700	0.80912000
C	-2.65980400	-0.17958800	0.84060500
H	-5.02538500	-2.34876100	-0.33006600
H	-3.03085600	0.91287100	-2.28610000
H	-4.66579000	-0.94456700	-2.39905000
H	-3.71441400	-1.81573500	1.71355800
C	-1.62393900	1.82598700	-0.33665800
C	-0.71950500	1.98028700	-1.53075900
H	0.06211300	1.21508300	-1.42592800
H	-1.24338600	1.72800100	-2.45835100
C	-0.13459200	3.39246500	-1.61807300
H	0.41688800	3.65139300	-0.71174600
H	0.55003200	3.45059600	-2.46841500
H	-0.92167400	4.13881900	-1.75871000
O	-1.77484700	2.59235000	0.56560000
N	-2.48829000	0.54786800	-0.30932900
C	-1.83954700	0.05413400	2.07436500
H	-1.68807400	1.11641400	2.26301800
H	-2.40710300	-0.35093300	2.91745400
C	-0.45134600	-0.66733200	2.02454800
H	-0.61639400	-1.71760600	1.76913600
C	0.19216500	-0.60842800	3.41701800
H	-0.46589400	-1.04903200	4.17312200
H	1.13322300	-1.16534800	3.42315100
H	0.39808900	0.42653900	3.71649100
N	0.40278800	-0.07166500	0.98861200
H	1.03204500	0.60552800	1.39587600
C	1.03773100	-0.87458200	-0.00230400
C	2.38580800	-0.66847000	-0.42513400
C	0.25597000	-1.86571000	-0.62542200
C	2.84525000	-1.49986300	-1.46104400
C	0.76143400	-2.67919900	-1.63338500

H	-0.77495700	-2.00243300	-0.31292300
C	2.07058500	-2.49191900	-2.05999600
H	3.86141500	-1.38481600	-1.81333700
H	0.13170900	-3.44332600	-2.08034200
H	2.49415200	-3.10714200	-2.84773500
C	3.35578400	0.37335300	0.20081800
C	2.83843900	1.82234600	0.00986600
H	1.87354500	2.02367100	0.48687400
H	3.55559100	2.53407400	0.43359200
H	2.73187100	2.05265800	-1.05620300
C	4.75013000	0.33638800	-0.46702000
H	5.24155100	-0.63395500	-0.34340400
H	4.70524300	0.57027700	-1.53587400
H	5.39078600	1.08836600	0.00474700
C	3.59717300	0.07430200	1.70300200
H	3.97557100	-0.94483500	1.83552500
H	4.34539900	0.76637300	2.10508800
H	2.70387300	0.17565300	2.32489600

D3(BJ)-B3LYP/6-31G*

SCF Done: E(RB3LYP) = -1002.33295404

Zero-point correction= 0.467295
Thermal correction to Gibbs Free Energy= 0.415464

C	-2.67750700	-0.62274700	-1.46577100
C	-3.59363700	-1.56759900	-1.05854000
C	-4.06547500	-1.51885300	0.25429000
C	-3.56404600	-0.54727600	1.11137800
C	-2.62047200	0.38856400	0.68474400
H	-4.80213200	-2.23389200	0.60560300
H	-2.31051200	-0.59557500	-2.47940300
H	-3.93850500	-2.31249400	-1.76564400
H	-3.88302900	-0.50844100	2.14662900
C	-1.35627000	1.45707300	-1.22317200
C	-0.36085700	1.04600700	-2.27449300
H	0.35644600	0.36102500	-1.80435800
H	-0.84908400	0.46508100	-3.06389000
C	0.31034200	2.27019500	-2.89915500
H	0.79005500	2.89297500	-2.14245200
H	1.06968100	1.94513700	-3.61474700
H	-0.42087000	2.88789000	-3.42708500
O	-1.59001500	2.57215400	-0.86061700
N	-2.21837200	0.34347900	-0.62610900
C	-1.94415500	1.27122900	1.68004900
H	-1.79297600	2.28071900	1.30440900
H	-2.58095000	1.33105400	2.56636100
C	-0.56514600	0.65978500	2.09913100
H	-0.74307700	-0.33759800	2.51144100
C	0.05800100	1.53749700	3.18758000
H	-0.63523600	1.67503300	4.02284300
H	0.96918900	1.07449400	3.57550700
H	0.30891000	2.52968900	2.79393400
N	0.32210800	0.53238000	0.94210300
H	1.02869200	1.25196700	0.94179900
C	0.80825300	-0.72002400	0.49570700
C	2.13192100	-0.88880200	-0.00541200
C	-0.08601500	-1.80549100	0.48249000
C	2.43749000	-2.14516900	-0.55059900
C	0.27113900	-3.04290600	-0.04070700
H	-1.07947400	-1.68352400	0.89645700
C	1.53924700	-3.21056700	-0.58245000

H	3.42745900	-2.31574800	-0.95034300
H	-0.44223900	-3.86164100	-0.02481400
H	1.84245600	-4.16119100	-1.00882500
C	3.23891300	0.18969800	0.09589800
C	2.90632900	1.45040800	-0.73430200
H	1.99610600	1.97162200	-0.42334900
H	3.72537800	2.17298500	-0.65738100
H	2.78889800	1.19034900	-1.79111900
C	4.58904800	-0.32435200	-0.44644100
H	4.94680100	-1.19800100	0.10665400
H	4.53647400	-0.58083700	-1.50945500
H	5.33970800	0.46416600	-0.33692200
C	3.48564800	0.56079600	1.57823200
H	3.74315300	-0.33249500	2.15586300
H	4.32031600	1.26587000	1.65145400
H	2.62581900	1.02833800	2.06307800

B3LYP/6-31G*/SMD (MeCN)

SCF Done: E(RB3LYP) = -1002.30335516

Zero-point correction= 0.465382
Thermal correction to Gibbs Free Energy= 0.412154

C	-3.34000400	0.19846400	-1.41600100
C	-4.09330000	-0.95193200	-1.48695200
C	-4.10461900	-1.81317400	-0.38795300
C	-3.36520300	-1.48206700	0.74061900
C	-2.61187900	-0.30852700	0.80164300
H	-4.68886900	-2.72735500	-0.40861900
H	-3.31751500	0.91785400	-2.22264300
H	-4.66434900	-1.15686900	-2.38474800
H	-3.35911900	-2.13288400	1.60778100
C	-1.93141600	1.85861800	-0.29932600
C	-1.00911100	2.11770300	-1.45504400
H	-0.20016400	1.37968800	-1.36988500
H	-1.51310400	1.88787100	-2.39971300
C	-0.47721300	3.54906100	-1.45968900
H	0.05808300	3.78156100	-0.53464200
H	0.21286400	3.67707000	-2.29903300
H	-1.29224800	4.27098800	-1.57398100
O	-2.17848900	2.59833400	0.61082900
N	-2.61547500	0.50429000	-0.30234100
C	-1.78116600	-0.02621700	2.02185200
H	-1.63205800	1.04016200	2.18445100
H	-2.34394500	-0.41252600	2.87684400
C	-0.39636100	-0.73824600	1.99828200
H	-0.55332700	-1.78958100	1.74853200
C	0.23310800	-0.66362600	3.39529800
H	-0.42910800	-1.11468300	4.14240900
H	1.18652500	-1.20159500	3.41163400
H	0.41660900	0.37763000	3.68815200
N	0.48368800	-0.14311800	0.98107700
H	1.03814100	0.60011300	1.37882900
C	1.16361700	-0.91033400	0.01666300
C	2.49195200	-0.60731100	-0.42713400
C	0.46947400	-1.98660900	-0.57709800
C	3.02378200	-1.43357400	-1.43196500
C	1.04480000	-2.78604400	-1.55907500
H	-0.55248900	-2.18641200	-0.27268600
C	2.33761700	-2.51113300	-1.99478800

H	4.02638200	-1.24146900	-1.79253800
H	0.47393500	-3.60761600	-1.98479900
H	2.81138500	-3.11492800	-2.76397600
C	3.35868900	0.54994800	0.14398600
C	2.69050000	1.92914800	-0.09057900
H	1.72320800	2.04942700	0.40429600
H	3.34418700	2.72519900	0.28624400
H	2.53662100	2.10143900	-1.16227700
C	4.73913300	0.63260400	-0.54617000
H	5.33246800	-0.27655400	-0.39890200
H	4.65450800	0.81945800	-1.62255100
H	5.30214000	1.46581700	-0.11087600
C	3.65005100	0.33437400	1.65116600
H	4.16519700	-0.62063600	1.80875200
H	4.30367800	1.13421700	2.02020400
H	2.75608900	0.33298100	2.27955800

M06-2X/6-31G*

SCF Done: E(RM062X) = -1001.76921256

Zero-point correction= 0.471081
Thermal correction to Gibbs Free Energy= 0.419913

C	-2.67993500	-0.51585100	-1.50312700
C	-3.58126700	-1.49628000	-1.15640300
C	-4.03581300	-1.54391200	0.16007300
C	-3.53602600	-0.63212500	1.07739800
C	-2.60492900	0.33774100	0.70625700
H	-4.76243500	-2.28875100	0.46769100
H	-2.32728000	-0.40897400	-2.51819300
H	-3.92941700	-2.19017400	-1.91147000
H	-3.84600600	-0.66639300	2.11587000
C	-1.37406900	1.54091100	-1.12909900
C	-0.35331000	1.20757000	-2.18192000
H	0.37324300	0.51997700	-1.72707200
H	-0.81151100	0.65836800	-3.00928600
C	0.28687200	2.48500900	-2.71853100
H	0.72967800	3.07664000	-1.91594500
H	1.06841500	2.22906700	-3.43634300
H	-0.45654900	3.10689400	-3.22191000
O	-1.63185300	2.61938600	-0.70296900
N	-2.21858800	0.38359000	-0.60138200
C	-1.92526600	1.14982400	1.76293000
H	-1.75426800	2.18085100	1.45911600
H	-2.57207800	1.15808900	2.64415700
C	-0.56845800	0.48457000	2.13613800
H	-0.76057600	-0.54607700	2.45331700
C	0.05940400	1.24648100	3.30103500
H	-0.63576200	1.31987300	4.14143400
H	0.95962300	0.73367700	3.64846100
H	0.32852800	2.26453500	2.99699200
N	0.32084800	0.46213700	0.97317100
H	1.04908900	1.15493500	1.07435500
C	0.81364000	-0.75743100	0.44393400
C	2.14114300	-0.88921200	-0.04712400
C	-0.07773100	-1.83483000	0.33306000
C	2.46706500	-2.10974900	-0.65102700
C	0.29513900	-3.03676200	-0.25420700
H	-1.08482400	-1.73925900	0.72673000
C	1.57650000	-3.17325900	-0.76379800

H	3.46677300	-2.25404400	-1.03919400
H	-0.41754400	-3.85375900	-0.30958200
H	1.89417500	-4.09761000	-1.23354500
C	3.21944900	0.20677700	0.09745400
C	2.84259800	1.48252400	-0.68065000
H	1.90615500	1.94857000	-0.35933400
H	3.63115700	2.23353200	-0.56756400
H	2.74641100	1.25711300	-1.74878300
C	4.57138500	-0.24884400	-0.47669200
H	4.95807000	-1.13060100	0.04270900
H	4.50969600	-0.46971700	-1.54664500
H	5.29997700	0.55665900	-0.34823600
C	3.47852100	0.52366100	1.58509200
H	3.75492700	-0.38857600	2.12230800
H	4.30699100	1.23390900	1.67090900
H	2.62486500	0.96519700	2.10552300

PBE0/def2-TZVPP

SCF Done: E(RPBE1PBE) = -1001.38155721

Zero-point correction= 0.464495
Thermal correction to Gibbs Free Energy= 0.410919

C	-3.06123600	0.10676100	-1.48262300
C	-3.93391400	-0.94637200	-1.44234600
C	-4.14082400	-1.58427500	-0.22611000
C	-3.46120200	-1.13913700	0.88862700
C	-2.58083300	-0.06482100	0.82428700
H	-4.82932900	-2.41673600	-0.15048300
H	-2.88325800	0.66213600	-2.39059900
H	-4.44818700	-1.24748000	-2.34412500
H	-3.60236600	-1.62039700	1.84728400
C	-1.59863600	1.81214800	-0.53081100
C	-0.62154500	1.82230200	-1.65586700
H	0.14892300	1.08696200	-1.38968200
H	-1.07618600	1.42495900	-2.56561700
C	-0.04445600	3.20393900	-1.89510500
H	0.44025800	3.59069400	-1.00022500
H	0.69384300	3.16031200	-2.69478500
H	-0.82135400	3.91126500	-2.18546400
O	-1.83385900	2.68225200	0.23508100
N	-2.40710500	0.53362900	-0.38171900
C	-1.81359300	0.33001800	2.03521900
H	-1.64284300	1.40223400	2.07771000
H	-2.42254000	0.05972300	2.89912700
C	-0.45108800	-0.40532100	2.15228600
H	-0.62566100	-1.47947600	2.05607200
C	0.12974400	-0.13598800	3.53248200
H	-0.56814300	-0.42797200	4.31803800
H	1.05089700	-0.70167000	3.67338200
H	0.35150700	0.92668400	3.66313000
N	0.46648700	0.01330600	1.11016100
H	1.15372500	0.65753400	1.45924400
C	0.98308800	-0.86893900	0.15074100
C	2.29057500	-0.72138400	-0.37897900
C	0.15506700	-1.88600400	-0.33518500
C	2.65502600	-1.60563500	-1.39386000
C	0.56734100	-2.75476400	-1.32932800
H	-0.83805800	-2.00907500	0.07813600
C	1.82687000	-2.60775400	-1.87496500

H	3.64246300	-1.52601700	-1.82366600
H	-0.09758400	-3.53939800	-1.66968500
H	2.17588300	-3.26929000	-2.65723900
C	3.31462600	0.30587000	0.13551700
C	2.83972500	1.75021100	-0.08586500
H	1.90199300	2.00372900	0.41096900
H	3.59461200	2.44694400	0.28448100
H	2.70716400	1.94477400	-1.15229500
C	4.65126000	0.19274700	-0.60357300
H	5.11295900	-0.78676100	-0.46990800
H	4.54967100	0.38725100	-1.67279200
H	5.34079000	0.93636900	-0.20061500
C	3.63429000	0.05653100	1.61872700
H	4.01881600	-0.95521700	1.75822900
H	4.40251000	0.75713500	1.95246200
H	2.78298800	0.17343000	2.28957600

B

B3LYP/6-31G*

SCF Done: E(RB3LYP) = -1002.21747165

Zero-point correction= 0.466471
Thermal correction to Gibbs Free Energy= 0.414159
Thermal correction to Gibbs Free Energy (353.15 K)= 0.399163

C	4.32640300	1.54401900	0.66854900
C	4.91006100	0.83270000	-0.37941800
C	3.00432100	1.26217400	0.99952500
H	4.87713500	2.30282700	1.21417800
C	4.15550100	-0.13275000	-1.04400000
H	5.93605500	1.02741700	-0.67716900
H	2.50375700	1.80256700	1.79897900
N	2.27317800	0.32787400	0.37056900
C	2.83947400	-0.36772300	-0.63655400
H	4.58164400	-0.70250400	-1.86396300
C	-0.23107100	-2.01933600	0.69962200
C	2.00188000	-1.46528200	-1.26291600
O	-0.19887200	-3.09824700	0.19189000
C	-0.28024600	-1.69844800	2.16430700
N	-0.22218100	-0.76851500	-0.25645400
H	2.44981000	-1.76580100	-2.21588400
H	2.04809500	-2.35638600	-0.62615400
C	0.52820800	-1.12453700	-1.56991300
H	0.63297800	-1.13199100	2.39705500
H	-1.11325900	-1.00818000	2.33934100
C	-0.39681300	-2.95348800	3.03244600
C	-1.65204900	-0.33932500	-0.44252000
H	0.38005600	-0.05219100	0.20354600
H	0.04445600	-2.03901100	-1.91257000
C	0.38015800	-0.04096100	-2.63412300
H	0.44763600	-3.62833800	2.87043100
H	-0.41227200	-2.66667400	4.08724100
H	-1.31611300	-3.50237000	2.81117900
C	-2.17284800	0.93212400	-0.08125100
C	-2.45405500	-1.34122300	-1.00593800
H	0.77529300	-0.44345500	-3.57252100
H	-0.66387400	0.22911200	-2.80546300
H	0.94615200	0.86174400	-2.39539100
C	-3.54933000	1.08598300	-0.34372100
C	-1.40097900	2.13964800	0.53133100

C	-3.80585100	-1.13111900	-1.24656200
H	-2.02096800	-2.30397200	-1.25133200
C	-4.35435200	0.09950000	-0.90774300
H	-4.02285500	2.02531600	-0.09842800
C	-2.34291500	3.33339000	0.81863100
C	-0.75032800	1.77840200	1.88812400
C	-0.32629900	2.66137600	-0.45190300
H	-4.40951000	-1.91934100	-1.68434200
H	-5.40747800	0.30206500	-1.07679300
H	-1.75329300	4.14330200	1.25865000
H	-3.13297100	3.08088500	1.53340600
H	-2.80459000	3.72973300	-0.09114200
H	-1.49377700	1.39162300	2.59394800
H	0.06000500	1.05021000	1.80538100
H	-0.31451200	2.67960000	2.33295600
H	-0.76722700	2.90009100	-1.42530700
H	0.12001900	3.57920400	-0.05342400
H	0.49038000	1.95425600	-0.60617000

D3(BJ)-B3LYP/6-31G*

SCF Done: E(RB3LYP) = -1002.34258538

Zero-point correction= 0.467827
Thermal correction to Gibbs Free Energy= 0.415901

C	4.24263200	1.47217700	0.70663500
C	4.82232800	0.80526100	-0.37198500
C	2.93694700	1.14549100	1.05777000
H	4.78404800	2.23187600	1.25946000
C	4.08159900	-0.16379800	-1.04632700
H	5.83499600	1.03779600	-0.68625800
H	2.43485500	1.65187500	1.87732000
N	2.22222000	0.20753500	0.41887200
C	2.78370000	-0.44663900	-0.61784800
H	4.50359400	-0.69895700	-1.89066200
C	-0.30818800	-2.03350400	0.67201100
C	1.95226000	-1.53484500	-1.25995900
O	-0.28196800	-3.11291600	0.16397800
C	-0.40641800	-1.70809000	2.12936800
N	-0.23649300	-0.78669200	-0.26581300
H	2.40922100	-1.83341800	-2.20816300
H	1.97440000	-2.42657000	-0.62434700
C	0.49261300	-1.15921600	-1.57524200
H	0.52188100	-1.18376300	2.39750400
H	-1.21280800	-0.97958600	2.26555100
C	-0.61624900	-2.95483000	2.98777200
C	-1.63470700	-0.29220000	-0.44836400
H	0.40323600	-0.10884700	0.20893600
H	-0.01812300	-2.05609300	-1.92332000
C	0.38092600	-0.06252600	-2.62619100
H	0.20238900	-3.66676900	2.85666100
H	-0.66346000	-2.67037000	4.04187900
H	-1.54921200	-3.45959100	2.72348700
C	-2.08174600	0.99968500	-0.08159500
C	-2.48658900	-1.24784800	-1.01359400
H	0.73415500	-0.47571800	-3.57580200
H	-0.65031500	0.26586500	-2.76837700
H	0.99986200	0.80415700	-2.38868000
C	-3.44578100	1.23583400	-0.33720200
C	-1.23178000	2.14293800	0.53086400
C	-3.82450400	-0.96120700	-1.24919600
H	-2.10288300	-2.22962700	-1.26464300

C	-4.30346200	0.29661800	-0.90327400
H	-3.86320400	2.19947700	-0.08598800
C	-2.08657200	3.39674100	0.81483300
C	-0.61524900	1.73061500	1.88542200
C	-0.12298300	2.58282300	-0.44900600
H	-4.47262600	-1.71151300	-1.68895100
H	-5.34402200	0.55630300	-1.06931600
H	-1.44260900	4.16531500	1.25167100
H	-2.89092700	3.19830200	1.53006600
H	-2.52059000	3.81750100	-0.09711600
H	-1.38930800	1.39720600	2.58450800
H	0.13538900	0.94406200	1.80075000
H	-0.11522100	2.59537600	2.33395100
H	-0.54703300	2.85358600	-1.42084100
H	0.39222900	3.46215800	-0.04818600
H	0.63508900	1.81652700	-0.60369000

B3LYP/6-31G*/SMD (MeCN)

SCF Done: E(RB3LYP) = -1002.29837197

Zero-point correction= 0.465840
Thermal correction to Gibbs Free Energy= 0.413215

C	4.33096300	1.58735600	0.60658700
C	4.91529900	0.83455800	-0.41250900
C	3.00980800	1.31466200	0.95192200
H	4.87942900	2.37117600	1.11896500
C	4.16323000	-0.15974000	-1.03584300
H	5.94044100	1.02055800	-0.72026400
H	2.50772100	1.88476500	1.72969900
N	2.28291700	0.35336700	0.36207400
C	2.84750200	-0.38163100	-0.61601900
H	4.58528200	-0.76153900	-1.83482700
C	-0.27388600	-2.04248000	0.71546100
C	2.00907200	-1.49814500	-1.20187400
O	-0.29551400	-3.12372100	0.20519900
C	-0.28800800	-1.72565200	2.17800900
N	-0.22651600	-0.79541800	-0.23799200
H	2.46364600	-1.84310100	-2.13561000
H	2.04215200	-2.35557500	-0.51927800
C	0.54487800	-1.15681600	-1.54003300
H	0.63560600	-1.17070700	2.39316800
H	-1.10864000	-1.02502800	2.36933100
C	-0.40597600	-2.97758900	3.04479400
C	-1.64311500	-0.33512100	-0.45236700
H	0.37747900	-0.08594500	0.23005700
H	0.06623500	-2.06793700	-1.89634000
C	0.42115200	-0.07231700	-2.60308100
H	0.43173200	-3.66053100	2.87397100
H	-0.40339300	-2.68746600	4.09973500
H	-1.33646900	-3.51557300	2.83851400
C	-2.16032700	0.93708900	-0.08343200
C	-2.44341500	-1.31556300	-1.05384900
H	0.87742100	-0.46514900	-3.51792700
H	-0.62021800	0.16848000	-2.82781400
H	0.94876400	0.84407300	-2.33082900
C	-3.52894600	1.10923800	-0.38044700
C	-1.40216600	2.14045600	0.55804000
C	-3.78615700	-1.08822100	-1.32713100
H	-2.01347500	-2.27571900	-1.31169400

C	-4.33084800	0.14220800	-0.98154400
H	-4.00099900	2.04865400	-0.13254500
C	-2.36725800	3.29937000	0.90619000
C	-0.71118900	1.76333400	1.88934000
C	-0.37156100	2.72509500	-0.43698300
H	-4.38387500	-1.86373800	-1.79572300
H	-5.37733600	0.36103000	-1.17370200
H	-1.78662500	4.09633100	1.38215900
H	-3.14906600	2.99434500	1.61052900
H	-2.84306100	3.73156600	0.01981700
H	-1.42718700	1.32954200	2.59628100
H	0.12287200	1.06897500	1.77463500
H	-0.29855300	2.66994400	2.34656100
H	-0.85264400	2.99244200	-1.38469300
H	0.06555100	3.63648700	-0.01174100
H	0.45220300	2.04321900	-0.65198700

M06-2X/6-31G*

SCF Done: E(RM062X) = -1001.78043288

Zero-point correction= 0.471310
Thermal correction to Gibbs Free Energy= 0.419634

C	4.25000700	1.46766800	0.69468700
C	4.81228600	0.81583200	-0.39820600
C	2.95430900	1.13036900	1.06441800
H	4.79655100	2.22411300	1.24572600
C	4.06545800	-0.14707300	-1.06899500
H	5.81831400	1.05562400	-0.72690800
H	2.46551300	1.62511400	1.90039400
N	2.23479500	0.19738600	0.43155100
C	2.77883600	-0.43792000	-0.61976900
H	4.47330100	-0.67232700	-1.92626400
C	-0.29086600	-2.00805000	0.66481400
C	1.93872300	-1.51974800	-1.26200000
O	-0.27198300	-3.07971800	0.15211500
C	-0.36910600	-1.69290900	2.12563900
N	-0.24320800	-0.77008700	-0.26375200
H	2.38773400	-1.81201300	-2.21614000
H	1.96103900	-2.41471800	-0.63117900
C	0.48462900	-1.12672100	-1.56857500
H	0.55571700	-1.15724900	2.38234300
H	-1.19082100	-0.98343400	2.27713500
C	-0.54251500	-2.95655200	2.96066700
C	-1.64687100	-0.29948600	-0.44496400
H	0.36972700	-0.07606100	0.21509900
H	-0.03655400	-2.01276400	-1.93324700
C	0.39425700	-0.01316300	-2.60152800
H	0.28922500	-3.64579500	2.80312700
H	-0.58217900	-2.69501900	4.01923400
H	-1.46676100	-3.47378800	2.69528300
C	-2.10278000	0.98317300	-0.07122100
C	-2.48468100	-1.26082200	-1.01286600
H	0.70308000	-0.43053900	-3.56375600
H	-0.62400500	0.36588500	-2.71358800
H	1.06309600	0.81832800	-2.36980500
C	-3.46730600	1.20676100	-0.31891300
C	-1.25294300	2.12414500	0.53656900
C	-3.82410000	-0.98600100	-1.24238900
H	-2.08955000	-2.23891100	-1.26755500

C	-4.31419000	0.26198900	-0.88705000
H	-3.89653700	2.16440400	-0.06036900
C	-2.10794200	3.37254700	0.81316300
C	-0.63237700	1.72157400	1.88688200
C	-0.14671500	2.55768700	-0.44035400
H	-4.46603400	-1.73951500	-1.68450500
H	-5.35780500	0.51043900	-1.04797700
H	-1.46463800	4.14590300	1.24108100
H	-2.90806500	3.17443500	1.53268000
H	-2.54589700	3.78169700	-0.10179300
H	-1.40031700	1.37765400	2.58762800
H	0.13544200	0.94776500	1.80398900
H	-0.14548700	2.59585200	2.33154400
H	-0.56649400	2.81699500	-1.41742800
H	0.36259200	3.44222400	-0.04352200
H	0.61788000	1.79254600	-0.58280600

PBE0/def2-TZVPP

SCF Done: E(RPBE1PBE) = -1001.38548323

Zero-point correction= 0.465302
Thermal correction to Gibbs Free Energy= 0.413030

C	4.28258000	1.50041100	0.68310400
C	4.86729200	0.78526300	-0.34993800
C	2.96509800	1.22888300	1.00138200
H	4.83102600	2.25671400	1.22842900
C	4.11863400	-0.17337900	-1.01080900
H	5.89403400	0.97309700	-0.63911800
H	2.46377000	1.77701600	1.79191800
N	2.24145900	0.30219500	0.37447300
C	2.80628200	-0.39790000	-0.61489400
H	4.54581800	-0.74826800	-1.82280500
C	-0.25397600	-1.99375700	0.69570600
C	1.97531100	-1.48398200	-1.23973400
O	-0.18856300	-3.06852800	0.20760300
C	-0.37569600	-1.66039700	2.13934400
N	-0.21264000	-0.76693200	-0.24913500
H	2.42385500	-1.78250200	-2.18864400
H	2.02131700	-2.37115600	-0.60306700
C	0.51707100	-1.13500700	-1.54101200
H	0.52746800	-1.09763200	2.40660400
H	-1.20439100	-0.95762900	2.25816800
C	-0.54515000	-2.89058800	3.00898500
C	-1.61900400	-0.32578000	-0.44259400
H	0.40818200	-0.06341400	0.21007100
H	0.02549700	-2.04380200	-1.88227700
C	0.38714600	-0.06249400	-2.60116700
H	0.29625100	-3.57340500	2.89731100
H	-0.61123500	-2.59537200	4.05538000
H	-1.45332000	-3.43348500	2.74842000
C	-2.12205900	0.94304200	-0.09253100
C	-2.42660900	-1.31238700	-1.00029500
H	0.75867500	-0.47743000	-3.53903300
H	-0.64794300	0.23523100	-2.76109300
H	0.97834400	0.82314900	-2.37420200
C	-3.48610500	1.11429000	-0.35691600
C	-1.34226300	2.13086300	0.50708800
C	-3.76641700	-1.08946800	-1.24499600
H	-2.00271200	-2.27826400	-1.24147200

C	-4.29703400	0.14192600	-0.91514000
H	-3.94341600	2.06083900	-0.11625000
C	-2.26007600	3.32683300	0.78122500
C	-0.70112800	1.77364900	1.85317400
C	-0.26925600	2.62321200	-0.47056100
H	-4.37699900	-1.86963100	-1.68019700
H	-5.34434800	0.35596700	-1.08749900
H	-1.66005000	4.12838900	1.21383800
H	-3.05113900	3.09074100	1.49511500
H	-2.71371500	3.72061100	-0.12948500
H	-1.44716000	1.41006700	2.56252200
H	0.09425400	1.03320200	1.77654900
H	-0.25121500	2.67035700	2.28409900
H	-0.70511300	2.86594200	-1.44102400
H	0.19225000	3.53071300	-0.07558700
H	0.53094300	1.90193000	-0.62137500

TS_{AB}

B3LYP/6-31G*

SCF Done: E(RB3LYP) = -1002.19864371

Zero-point correction= 0.466180
Thermal correction to Gibbs Free Energy= 0.415397
Thermal correction to Gibbs Free Energy (353.15 K)= 0.400821

Imaginary frequency -- –175.33

C	4.75219700	0.90726700	0.63079300
C	5.16690900	-0.41095900	0.44068500
C	3.42077900	1.22155100	0.38687200
H	5.44051200	1.67947400	0.95635700
C	4.23399000	-1.36355000	0.03563200
H	6.20020900	-0.69532500	0.61428000
H	3.05086000	2.23259600	0.51651700
N	2.53107000	0.30043100	-0.01782300
C	2.91109200	-0.98078000	-0.19300600
H	4.52094900	-2.40149300	-0.09896500
C	0.85276900	0.79884500	-0.97160000
C	1.83296600	-1.96319500	-0.56863600
O	1.00259500	0.48437900	-2.10842200
C	0.46755700	2.12893100	-0.37601600
N	-0.21943100	-0.56404200	-0.15292600
H	1.58693700	-1.87969800	-1.63406700
H	2.21083700	-2.97752500	-0.41167100
C	0.56934400	-1.78342100	0.29426500
H	-0.62680100	2.16263100	-0.37482200
H	0.77352700	2.18525200	0.66990900
C	1.01524300	3.28861400	-1.22315000
C	-1.23834000	-0.11220800	0.79587400
H	-0.67260300	-0.81459600	-1.03081800
H	0.88237100	-1.57315700	1.31738400
C	-0.29533400	-3.04636300	0.29972600
H	0.70366100	4.23827600	-0.77868400
H	2.10705100	3.28144700	-1.28599100
H	0.62550200	3.23844200	-2.24293100
C	-2.60585000	0.05564500	0.44555400
C	-0.76906000	0.19926300	2.07987500
H	-1.22536700	-2.89280700	0.85306100
H	-0.53909600	-3.37760500	-0.71500300
H	0.25643200	-3.85471000	0.79018300

C	-3.43558600	0.51136500	1.48837800
C	-3.22975400	-0.19435800	-0.96204000
C	-1.62763300	0.65176200	3.07381000
H	0.28893700	0.09991900	2.30085600
C	-2.97706300	0.79962700	2.77185000
H	-4.49049600	0.64882300	1.29767800
C	-2.62732900	0.76815700	-2.01773900
C	-3.05951000	-1.66305300	-1.42739100
C	-4.75394300	0.07214800	-0.95963100
H	-1.24391000	0.88404600	4.06223700
H	-3.67790700	1.14464300	3.52582500
H	-2.76557000	1.81257800	-1.71607200
H	-1.56285300	0.60984700	-2.21584200
H	-3.14288900	0.63190000	-2.97454200
H	-2.02935000	-1.93994400	-1.67387200
H	-3.42229100	-2.36478700	-0.66928100
H	-3.64122700	-1.82509400	-2.34070500
H	-4.99456000	1.10891500	-0.70400200
H	-5.14496100	-0.11180900	-1.96483700
H	-5.29079500	-0.59172400	-0.27418100

D3(BJ)-B3LYP/6-31G*

SCF Done: E(RB3LYP) = -1002.32372634

Zero-point correction= 0.467796
Thermal correction to Gibbs Free Energy= 0.417643

Imaginary frequency -- –197.74

C	-4.63352300	-1.02292200	0.58657000
C	-5.09004600	0.29326700	0.51779500
C	-3.30377000	-1.27712300	0.27826000
H	-5.28954400	-1.83890900	0.86742400
C	-4.19841400	1.30410900	0.16522100
H	-6.12443800	0.53057200	0.74475000
H	-2.89910600	-2.28137200	0.31045800
N	-2.45772900	-0.29690300	-0.07432400
C	-2.87517500	0.98305900	-0.13426800
H	-4.51682200	2.34031300	0.12595100
C	-0.83577800	-0.66615100	-1.05991800
C	-1.83243700	2.01957400	-0.44528600
O	-0.98402200	-0.25621900	-2.17039100
C	-0.40528000	-2.03300000	-0.59721000
N	0.21287700	0.61188100	-0.12762900
H	-1.58612700	2.01599100	-1.51283700
H	-2.23336200	3.00878000	-0.20987000
C	-0.56960200	1.79263800	0.40064600
H	0.68802300	-2.04108100	-0.61822000
H	-0.68601400	-2.19474800	0.44424700
C	-0.94474800	-3.11531200	-1.54191400
C	1.18719900	0.06322400	0.80386200
H	0.69258000	0.92748400	-0.96839600
H	-0.88021700	1.51591700	1.40798800
C	0.30057100	3.04411600	0.48534400
H	-0.61275200	-4.09842500	-1.19720000
H	-2.03726000	-3.11869200	-1.59141900
H	-0.57068900	-2.95601000	-2.55605700
C	2.55326900	-0.10537700	0.47097000
C	0.67283900	-0.35810400	2.03578700
H	1.23034400	2.84232900	1.02305600
H	0.54442300	3.43536900	-0.50720900
H	-0.24252800	3.82326200	1.02862700
C	3.34789900	-0.68231500	1.47792400
C	3.19935400	0.26589200	-0.89090000

C	1.49471900	-0.92895500	2.99822400
H	-0.38906500	-0.25634800	2.23361300
C	2.84777100	-1.08279600	2.71429000
H	4.40294900	-0.82787500	1.29661800
C	2.59983300	-0.58109600	-2.03922000
C	3.05296800	1.77175100	-1.21376100
C	4.71606800	-0.02261500	-0.88753400
H	1.08127000	-1.24851300	3.94912100
H	3.51879800	-1.51979000	3.44676400
H	2.71357700	-1.65044000	-1.83104600
H	1.54316400	-0.38451700	-2.24008800
H	3.13672600	-0.36708700	-2.96906100
H	2.03493900	2.07667500	-1.47309800
H	3.38512500	2.39050000	-0.37456200
H	3.67119600	2.01872000	-2.08224000
H	4.93416900	-1.08296400	-0.72857100
H	5.12936600	0.25172800	-1.86218700
H	5.24511900	0.56283600	-0.12905700

B3LYP/6-31G*/SMD (MeCN)

SCF Done: E(RB3LYP) = -1002.28219317

Zero-point correction= 0.466170
Thermal correction to Gibbs Free Energy= 0.415733

Imaginary frequency -- –187.09

C	-4.71413500	-0.97132100	0.58363300
C	-5.15844500	0.34349500	0.44379500
C	-3.37531800	-1.24853400	0.33355400
H	-5.38427100	-1.77171900	0.87790800
C	-4.24658500	1.33266100	0.08152700
H	-6.19830600	0.59749800	0.62637400
H	-2.98573400	-2.25522100	0.42816400
N	-2.50752400	-0.29162800	-0.03275000
C	-2.91543300	0.98652200	-0.15571400
H	-4.55017700	2.37068700	-0.00790600
C	-0.80743300	-0.69998200	-0.99204800
C	-1.85256600	2.00188600	-0.47226400
O	-0.98105300	-0.35731400	-2.12914100
C	-0.43496500	-2.07498200	-0.48818000
N	0.19953400	0.56568900	-0.15428900
H	-1.60709100	2.00037100	-1.54050100
H	-2.23647600	2.99739200	-0.23467300
C	-0.59036100	1.76785500	0.37094600
H	0.65852000	-2.12532400	-0.50987900
H	-0.73122100	-2.20263000	0.55384700
C	-1.00430500	-3.17226200	-1.39609600
C	1.21241500	0.06380800	0.78425300
H	0.66314900	0.89141600	-1.00388500
H	-0.89652600	1.50250900	1.38180600
C	0.28395200	3.01848000	0.43198300
H	-0.71162000	-4.15064300	-1.00156700
H	-2.09613600	-3.14109100	-1.45566200
H	-0.60850000	-3.07845700	-2.41174500
C	2.58741800	-0.07787300	0.45021300
C	0.72154500	-0.31600300	2.04082000
H	1.21087800	2.83883900	0.98376600
H	0.53074400	3.38569200	-0.56952900
H	-0.27343100	3.80298100	0.95426000
C	3.40239000	-0.57672900	1.48604500
C	3.24160400	0.23721400	-0.93056800

C	1.56562100	-0.81374000	3.02643900
H	-0.34060600	-0.23756300	2.24578200
C	2.92245600	-0.93370400	2.74481800
H	4.46203100	-0.69758400	1.30957900
C	2.63831900	-0.64731500	-2.04985300
C	3.11422400	1.73289400	-1.31301500
C	4.75793000	-0.06723400	-0.91947400
H	1.16209500	-1.10214700	3.99241300
H	3.61328200	-1.31216600	3.49302300
H	2.74898300	-1.71089600	-1.81017500
H	1.58026800	-0.45469900	-2.24639800
H	3.17261800	-0.45710700	-2.98812100
H	2.09644100	2.04655600	-1.56071200
H	3.48044800	2.37960100	-0.50816500
H	3.72089000	1.92741600	-2.20490000
H	4.96957400	-1.12065300	-0.70760400
H	5.16321400	0.15584300	-1.91195200
H	5.30133900	0.55035600	-0.19601600

M06-2X/6-31G*

SCF Done: E(RM062X) = -1001.75831534

Zero-point correction= 0.471692
Thermal correction to Gibbs Free Energy= 0.422524

Imaginary frequency --   –206.25

C	-4.53170000	-1.09611200	0.54717600
C	-5.02591800	0.20475000	0.54281200
C	-3.19950200	-1.29736200	0.22345900
H	-5.16122100	-1.94402600	0.78888000
C	-4.16736700	1.25552600	0.23944700
H	-6.06593200	0.39974100	0.78269700
H	-2.76827100	-2.29136100	0.20376400
N	-2.38523700	-0.27816500	-0.08335900
C	-2.84070100	0.98799700	-0.07894000
H	-4.51308700	2.28345200	0.25162300
C	-0.85031500	-0.54098900	-1.03979200
C	-1.82209100	2.06227200	-0.32870400
O	-1.00273500	-0.06800700	-2.13221700
C	-0.40908900	-1.95427500	-0.72840100
N	0.18424100	0.59945500	-0.10399200
H	-1.58751300	2.13778500	-1.39596000
H	-2.23133200	3.02346600	-0.00701300
C	-0.55782400	1.77497600	0.48730400
H	0.68367500	-1.96351600	-0.77599900
H	-0.66349700	-2.23379200	0.29639700
C	-0.97077700	-2.91212300	-1.78078600
C	1.15675200	-0.00015300	0.80499900
H	0.67541000	0.95253300	-0.92784500
H	-0.85957400	1.46128300	1.48818200
C	0.34016700	2.99845700	0.60587300
H	-0.66612800	-3.93545000	-1.55239900
H	-2.06229400	-2.87925800	-1.83599200
H	-0.58965500	-2.64336300	-2.76792900
C	2.52085200	-0.14338800	0.47059700
C	0.63320600	-0.48477000	2.00512800
H	1.28219800	2.75056000	1.10211600
H	0.55569700	3.43426600	-0.37417800
H	-0.16925200	3.75717300	1.20558800
C	3.31174300	-0.75797900	1.45459400
C	3.16345300	0.28770800	-0.87149800

C	1.45091600	-1.09547100	2.94339100
H	-0.43329800	-0.40037500	2.19595200
C	2.80527200	-1.21958500	2.66436900
H	4.37038100	-0.88757700	1.27678900
C	2.55700200	-0.49135400	-2.05691000
C	3.03672600	1.80413200	-1.11972100
C	4.67118300	-0.01904700	-0.88346300
H	1.03348700	-1.46683200	3.87281700
H	3.47428700	-1.68424800	3.38075600
H	2.68317400	-1.56995100	-1.91372000
H	1.49557200	-0.29287900	-2.24042700
H	3.08567600	-0.21662500	-2.97517200
H	2.02683200	2.13306300	-1.38241500
H	3.36607600	2.37762600	-0.24778100
H	3.67086100	2.08278100	-1.96660400
H	4.87337000	-1.08733600	-0.76413600
H	5.08327300	0.28619700	-1.84877600
H	5.20802600	0.53244000	-0.10562000

PBE0/def2-TZVPP

SCF Done: E(RPBE1PBE) = -1001.36671860

Zero-point correction= 0.465604
Thermal correction to Gibbs Free Energy= 0.415330

Imaginary frequency -- –182.84

C	-4.60829000	-1.01762700	0.57962700
C	-5.07964600	0.28025700	0.45951300
C	-3.27263800	-1.25669500	0.32694900
H	-5.25935000	-1.83471700	0.85882400
C	-4.19657000	1.28922100	0.11513000
H	-6.12298900	0.50559000	0.64224800
H	-2.85922600	-2.25373100	0.40070600
N	-2.43458400	-0.28130600	-0.02067800
C	-2.86716200	0.97906800	-0.12457500
H	-4.52649600	2.31724700	0.03782100
C	-0.82302900	-0.63359000	-0.97935400
C	-1.82926800	2.00662100	-0.42903600
O	-0.99604100	-0.24826200	-2.09384000
C	-0.43210000	-2.01877700	-0.54862400
N	0.19246000	0.56326600	-0.13685900
H	-1.60618700	2.02338700	-1.49938600
H	-2.21430200	2.99156100	-0.16461600
C	-0.56530700	1.75537500	0.38646000
H	0.65925000	-2.05096800	-0.57111400
H	-0.70746000	-2.19990500	0.48940700
C	-0.98665000	-3.06710500	-1.50207700
C	1.18556700	0.04873500	0.79306800
H	0.67237900	0.86816700	-0.98014900
H	-0.86404800	1.48724500	1.39852600
C	0.31474700	2.98765500	0.45393600
H	-0.67480200	-4.05987900	-1.17764700
H	-2.07552800	-3.05213100	-1.55470700
H	-0.61374300	-2.90085900	-2.51180700
C	2.54360100	-0.09937900	0.44794800
C	0.70317500	-0.33308900	2.04074300
H	1.24649200	2.78683200	0.98201900
H	0.54751900	3.37454400	-0.53936100
H	-0.21446500	3.77029200	0.99870200
C	3.35740700	-0.61826900	1.46051000
C	3.17398600	0.23262200	-0.92242100

C	1.54352300	-0.84974100	3.00632100
H	-0.35373800	-0.23900500	2.25726500
C	2.88702100	-0.98344500	2.71003300
H	4.41153200	-0.74533400	1.26917500
C	2.58619300	-0.65206800	-2.03194300
C	3.00972200	1.71228500	-1.29951800
C	4.68315400	-0.03298900	-0.91674200
H	1.15080400	-1.14128200	3.97185200
H	3.57664500	-1.37689700	3.44616700
H	2.72044700	-1.71038400	-1.79887200
H	1.52790600	-0.47846800	-2.23344600
H	3.11321600	-0.45382700	-2.96731800
H	1.98976500	2.00238300	-1.55777900
H	3.35396400	2.36894800	-0.49916000
H	3.60982000	1.92299200	-2.18632200
H	4.91865600	-1.08049700	-0.72288000
H	5.08237800	0.21083700	-1.90191500
H	5.21013600	0.58585700	-0.18844100

C

B3LYP/6-31G*

SCF Done: E(RB3LYP) = -1002.21596507

Zero-point correction= 0.466299
Thermal correction to Gibbs Free Energy= 0.413756
Thermal correction to Gibbs Free Energy (353.15 K)= 0.398699

C	-4.34668800	1.51380200	0.76148400
C	-4.93965300	0.80918200	-0.28736600
C	-2.99266900	1.31386000	1.00447900
H	-4.91651100	2.20500700	1.37327000
C	-4.15888200	-0.06211800	-1.04218100
H	-5.99280100	0.94083400	-0.51706300
H	-2.48475000	1.85034600	1.80179700
N	-2.23515700	0.46601700	0.28710400
C	-2.80487700	-0.21617700	-0.72441500
H	-4.59009900	-0.61946800	-1.86820800
C	0.00806000	-1.86833700	0.75246200
C	-1.94242700	-1.20060200	-1.49255000
O	-0.21986600	-2.95166600	0.30549000
C	0.09318400	-1.47728800	2.20317800
N	0.22501200	-0.68361100	-0.23346300
H	-2.06026300	-2.20374900	-1.06651800
H	-2.32779800	-1.27179700	-2.51553000
C	-0.44194000	-0.87684800	-1.63591100
H	0.97019300	-0.83698700	2.34931300
H	-0.78258300	-0.84194400	2.40181800
C	0.11464800	-2.69100900	3.13524300
C	1.68583600	-0.33492600	-0.26371300
H	-0.36844700	0.07548300	0.17418600
H	-0.33283400	0.12006600	-2.06832500
C	0.26029900	-1.88134200	-2.54627600
H	0.99642300	-3.31206400	2.95419300
H	0.14065400	-2.35329700	4.17464100
H	-0.77266700	-3.31353600	2.99569000
C	2.19631300	0.98240400	-0.12276200
C	2.52726200	-1.44288800	-0.43500300
H	-0.23904400	-1.84407300	-3.52001400
H	1.30738700	-1.62375200	-2.71258300
H	0.19049600	-2.90270700	-2.16815000
C	3.59979400	1.07373300	-0.20630400
C	1.38273800	2.28150100	0.14348100

C	3.90776500	-1.29620200	-0.49766800
H	2.10063200	-2.43452700	-0.52623400
C	4.44356400	-0.01909900	-0.38975700
H	4.06358300	2.04553700	-0.11884800
C	0.35061100	2.55664500	-0.97757600
C	0.68622200	2.20055500	1.52484100
C	2.29675100	3.52882600	0.19665100
H	4.54148700	-2.16685400	-0.63095300
H	5.51673400	0.13721500	-0.44031000
H	0.82623900	2.53016200	-1.96418200
H	-0.07624500	3.55635100	-0.84400400
H	-0.49243700	1.86355400	-0.97336500
H	1.42163700	2.05081200	2.32277000
H	0.16037400	3.14002000	1.72815500
H	-0.05517700	1.40004900	1.59524400
H	1.67456100	4.40868100	0.38635200
H	3.03367600	3.47559800	1.00417900
H	2.82206100	3.69949800	-0.74874700

D3(BJ)-B3LYP/6-31G*

SCF Done: E(RB3LYP) = -1002.34048577

Zero-point correction= 0.467514
Thermal correction to Gibbs Free Energy= 0.415257

C	-4.28195100	1.46856100	0.78693900
C	-4.88508000	0.77348200	-0.26227900
C	-2.92850500	1.25944100	1.02078400
H	-4.84411700	2.15964800	1.40515700
C	-4.11530000	-0.09800500	-1.02745000
H	-5.93831000	0.91347500	-0.48458900
H	-2.40901200	1.78780500	1.81512000
N	-2.18479000	0.41072100	0.29318200
C	-2.76179100	-0.26236900	-0.71962100
H	-4.55378000	-0.64726300	-1.85441500
C	0.03049200	-1.85356400	0.74707600
C	-1.90706700	-1.24024600	-1.49896200
O	-0.20541000	-2.94404200	0.32115700
C	0.14117200	-1.43713300	2.18555200
N	0.22974000	-0.68671500	-0.24469500
H	-2.01527100	-2.24370400	-1.07303400
H	-2.29891100	-1.30796200	-2.51891300
C	-0.41288200	-0.90473500	-1.64172300
H	0.99747200	-0.76410700	2.29694700
H	-0.75233900	-0.83066800	2.39439700
C	0.22957700	-2.63562200	3.12966700
C	1.67344400	-0.31312000	-0.26713300
H	-0.38915500	0.06068500	0.15592300
H	-0.31072500	0.08462000	-2.09082300
C	0.31176400	-1.91716600	-2.51963700
H	1.12923600	-3.22443400	2.93034700
H	0.26867300	-2.28684600	4.16458900
H	-0.63677400	-3.29152400	3.01585200
C	2.14217000	1.01496100	-0.13676800
C	2.54223600	-1.40034600	-0.41536900
H	-0.18322000	-1.91943000	-3.49570500
H	1.35373100	-1.64009500	-2.68476600
H	0.26153100	-2.92646500	-2.10914400
C	3.54040300	1.15262400	-0.21037700
C	1.27681800	2.27330400	0.11747500
C	3.91747200	-1.21267600	-0.46834800
H	2.14098700	-2.40316200	-0.49548600

C	4.41487000	0.08146900	-0.37396200
H	3.97210500	2.13940500	-0.13065900
C	0.23307000	2.49198600	-1.00137300
C	0.59019900	2.16540900	1.49852600
C	2.13609500	3.55499300	0.15990700
H	4.57843900	-2.06481700	-0.58418600
H	5.48325900	0.26672100	-0.41872100
H	0.70753100	2.46215000	-1.98788000
H	-0.22666000	3.47835800	-0.88301300
H	-0.58480400	1.77227300	-0.97924100
H	1.33672800	2.05936600	2.29233900
H	0.01496200	3.07616500	1.69624800
H	-0.10315600	1.32589100	1.57530200
H	1.47901700	4.40991900	0.34318300
H	2.87479000	3.53522200	0.96670800
H	2.65315900	3.73557300	-0.78776100

B3LYP/6-31G*/SMD (MeCN)

SCF Done: E(RB3LYP) = -1002.29605371

Zero-point correction= 0.465589
Thermal correction to Gibbs Free Energy= 0.412540

C	-4.34874800	1.56209300	0.69766500
C	-4.95722400	0.77978300	-0.28647100
C	-2.98510600	1.39967300	0.91603500
H	-4.91390200	2.28367200	1.27867400
C	-4.18277500	-0.12725800	-1.00421400
H	-6.01870200	0.87980900	-0.49498500
H	-2.46367200	1.99171900	1.66375500
N	-2.23492100	0.51963700	0.23174000
C	-2.81667500	-0.23715000	-0.71520600
H	-4.62250300	-0.74551400	-1.78100300
C	0.03207200	-1.86748500	0.79451500
C	-1.95683300	-1.24873500	-1.44515700
O	-0.16770700	-2.96882200	0.37122300
C	0.11027900	-1.44452500	2.23222800
N	0.22192800	-0.70325300	-0.21655900
H	-2.07536200	-2.23220800	-0.97585000
H	-2.34664800	-1.36053300	-2.46215600
C	-0.46180300	-0.92210600	-1.61075300
H	0.98678600	-0.80113300	2.36404800
H	-0.76264000	-0.80065400	2.41346000
C	0.12871500	-2.63148000	3.19300100
C	1.67906400	-0.34123600	-0.27206700
H	-0.36715500	0.06449100	0.18132600
H	-0.36192400	0.06725800	-2.06106500
C	0.23138700	-1.93561900	-2.51462100
H	1.00459400	-3.26534400	3.02164200
H	0.16805900	-2.26270400	4.22234800
H	-0.76903100	-3.24694300	3.08264300
C	2.18968300	0.97781100	-0.13459700
C	2.52205800	-1.44409100	-0.46214500
H	-0.29400000	-1.91427400	-3.47523500
H	1.27207800	-1.67082000	-2.70893400
H	0.18064500	-2.95274600	-2.12148000
C	3.59226500	1.07168100	-0.24220500
C	1.38636200	2.28244700	0.14145600
C	3.90125500	-1.29544300	-0.54907600
H	2.09933000	-2.43732100	-0.54775300

C	4.43642200	-0.01793900	-0.44477800
H	4.05729200	2.04326000	-0.15825000
C	0.37067100	2.57893500	-0.98724800
C	0.68038300	2.21011200	1.51818500
C	2.31325000	3.51915200	0.20820300
H	4.53208600	-2.16625300	-0.69704100
H	5.50852700	0.14299400	-0.51256600
H	0.86589200	2.59050200	-1.96490400
H	-0.07421900	3.56725400	-0.82332800
H	-0.45643300	1.86871700	-1.02713500
H	1.40947900	2.03126700	2.31675500
H	0.18772600	3.16812100	1.72161600
H	-0.08841600	1.43771700	1.58548200
H	1.69394200	4.40072000	0.40374700
H	3.04801900	3.45001300	1.01749100
H	2.84452800	3.69309300	-0.73385700

M06-2X/6-31G*

SCF Done: E(RM062X) = -1001.77833681

Zero-point correction= 0.470912
Thermal correction to Gibbs Free Energy= 0.418783

C	-4.29502600	1.46216900	0.76395300
C	-4.88069800	0.77692000	-0.29768800
C	-2.94821900	1.24832200	1.01714900
H	-4.86510700	2.14957600	1.37789700
C	-4.10169600	-0.08818100	-1.05504300
H	-5.92984800	0.91971400	-0.53535500
H	-2.44192000	1.76917300	1.82631900
N	-2.19549800	0.40563800	0.29834600
C	-2.75572200	-0.25463400	-0.72544500
H	-4.52565000	-0.63206200	-1.89293600
C	0.00997200	-1.80825900	0.75692300
C	-1.89324000	-1.23020600	-1.49920200
O	-0.22449200	-2.89715400	0.34054400
C	0.11151800	-1.38059900	2.19243800
N	0.23701700	-0.66892500	-0.24364000
H	-2.00859700	-2.23555700	-1.07913800
H	-2.27450700	-1.28962300	-2.52365900
C	-0.39916900	-0.89896000	-1.63072300
H	0.99446000	-0.74206800	2.30910100
H	-0.76359000	-0.74286500	2.38363300
C	0.14235500	-2.58164300	3.13108100
C	1.68461600	-0.32422700	-0.26446300
H	-0.34951300	0.10206500	0.14823600
H	-0.28895200	0.08181600	-2.10063700
C	0.32137600	-1.93152700	-2.48643000
H	1.01760700	-3.20494800	2.93507600
H	0.18601800	-2.23975300	4.16631600
H	-0.74920000	-3.19835100	3.00426100
C	2.16261500	0.99646600	-0.13647700
C	2.53703000	-1.42160800	-0.39973000
H	-0.18359800	-1.95813800	-3.45594800
H	1.36058200	-1.65667700	-2.66988500
H	0.27282000	-2.93033600	-2.05010200
C	3.55955400	1.12074600	-0.20283800
C	1.29942500	2.25442200	0.10765300
C	3.91199600	-1.24749000	-0.44714200
H	2.12093600	-2.42103600	-0.47597400

C	4.42065600	0.04016400	-0.35719500
H	4.00334700	2.10344800	-0.12570100
C	0.26171200	2.47126500	-1.01052600
C	0.60556200	2.16082500	1.48067700
C	2.16432100	3.52593200	0.14597800
H	4.56551700	-2.10582700	-0.55450000
H	5.49082900	0.21305100	-0.39763800
H	0.73297400	2.41671800	-1.99740500
H	-0.17811700	3.46790300	-0.90580400
H	-0.57342100	1.76898500	-0.97423200
H	1.34474300	2.03987500	2.27918000
H	0.04923100	3.08486200	1.67104400
H	-0.11153600	1.33805900	1.55647200
H	1.51197500	4.38557100	0.32054800
H	2.89789700	3.50344300	0.95683900
H	2.68629200	3.69384300	-0.80100300

PBE0/def2-TZVPP

SCF Done: E(RPBE1PBE) = -1001.38381323

Zero-point correction= 0.465124
Thermal correction to Gibbs Free Energy= 0.412713

C	-4.29129200	1.49462100	0.77965400
C	-4.89481500	0.77677400	-0.24261200
C	-2.93736200	1.32149900	0.98602700
H	-4.85546200	2.17740100	1.40062900
C	-4.12413900	-0.07932900	-1.00672300
H	-5.95319600	0.88764800	-0.44409700
H	-2.42198500	1.87177500	1.76588800
N	-2.19072100	0.49004000	0.25696100
C	-2.76736300	-0.20440100	-0.72640700
H	-4.56405000	-0.64874200	-1.81602000
C	-0.01977700	-1.83160900	0.73635100
C	-1.91570900	-1.16377500	-1.51293000
O	-0.29738800	-2.89750300	0.30394700
C	0.11004300	-1.44089700	2.17025900
N	0.21057700	-0.67109900	-0.23596700
H	-2.05512800	-2.17534300	-1.12296700
H	-2.29015600	-1.19379400	-2.53832600
C	-0.42336800	-0.85747000	-1.62491300
H	1.00820500	-0.83005000	2.28983100
H	-0.73490500	-0.77109000	2.37606000
C	0.10352300	-2.63548000	3.10374500
C	1.65682600	-0.34251600	-0.25365700
H	-0.39477600	0.08945800	0.16128100
H	-0.30197300	0.13528200	-2.06014800
C	0.27550600	-1.85892700	-2.51971700
H	0.95388800	-3.28936900	2.91058700
H	0.16233400	-2.29666200	4.13726700
H	-0.80514300	-3.22435600	2.98582400
C	2.17573400	0.95998100	-0.12314000
C	2.48411000	-1.45209000	-0.40054300
H	-0.21448000	-1.82134700	-3.49387800
H	1.32196200	-1.60838100	-2.67904300
H	0.19674900	-2.87660800	-2.14285400
C	3.57075500	1.03992600	-0.19473300
C	1.37764000	2.25276400	0.12455500
C	3.85777300	-1.32170200	-0.44910600
H	2.04746100	-2.43813200	-0.48644700

C	4.40092600	-0.05579400	-0.35443600
H	4.03756500	2.00899000	-0.11482000
C	0.35973600	2.52175600	-0.99096500
C	0.68050000	2.18432100	1.49025600
C	2.29227500	3.48092300	0.17162900
H	4.48320700	-2.19731200	-0.56337600
H	5.47313200	0.08916100	-0.39645000
H	0.83583500	2.48686400	-1.97285500
H	-0.05963900	3.52157800	-0.86387000
H	-0.48522900	1.83607600	-0.98161300
H	1.40927200	2.03853500	2.28978000
H	0.15978500	3.12490600	1.68197000
H	-0.06169400	1.38927200	1.56190200
H	1.67599300	4.36286900	0.35069400
H	3.02219900	3.42821000	0.98079200
H	2.82121400	3.63914400	-0.76983700

TS$_{AC}$

B3LYP/6-31G*

SCF Done: E(RB3LYP) = -1002.18210168

Zero-point correction= 0.466066
Thermal correction to Gibbs Free Energy= 0.415225
Thermal correction to Gibbs Free Energy (353.15 K)= 0.400596

Imaginary frequency -- –153.48

C	-4.34812900	-0.46966900	-1.38651900
C	-4.76472100	-1.13899800	-0.23597900
C	-3.12163600	0.18508100	-1.35893100
H	-4.95526800	-0.45056900	-2.28506400
C	-3.93824000	-1.13595100	0.88596500
H	-5.71637900	-1.66121500	-0.21415800
H	-2.75321800	0.71807100	-2.23011500
N	-2.33455600	0.19469200	-0.27261500
C	-2.72096400	-0.45121900	0.84272100
H	-4.22883300	-1.65969900	1.79117300
C	-0.83738700	1.67311200	0.04715200
C	-1.77843700	-0.42137200	2.02142000
O	-1.31284700	2.40315500	0.84908400
C	-0.32337700	1.93359800	-1.34020900
N	0.31012100	0.50872600	0.99024000
H	-1.85911600	0.54141200	2.54337300
H	-2.09343300	-1.18458200	2.73916600
C	-0.30655800	-0.70589100	1.66082700
H	0.74990500	2.13256100	-1.23730700
H	-0.41620300	1.03583600	-1.94920600
C	-1.02685000	3.14847200	-1.96451200
C	1.73159900	0.48477400	0.56624200
H	0.30785800	1.19319400	1.74821100
H	-0.27233400	-1.52454200	0.94794300
C	0.50230800	-1.06272100	2.91231600
H	-0.62063300	3.32332000	-2.96493700
H	-2.10630000	2.99720400	-2.05308700
H	-0.86413700	4.04626800	-1.36311400
C	2.39375100	-0.41967600	-0.31691600
C	2.43071800	1.57147800	1.12913800
H	1.56033000	-1.21291200	2.68435900
H	0.42076100	-0.27626000	3.67271100
H	0.11570800	-1.98903900	3.34881600

C	3.76093600	-0.13638600	-0.52425500
C	1.84507900	-1.69965500	-1.02215500
C	3.77234600	1.81336300	0.87606900
H	1.90156000	2.25252900	1.79377900
C	4.44705900	0.93476200	0.03797500
H	4.32803800	-0.79249200	-1.16845800
C	1.87776900	-2.89400000	-0.03189100
C	0.43954400	-1.51377500	-1.63474800
C	2.73609500	-2.12222000	-2.22178700
H	4.27126700	2.66130800	1.33398200
H	5.50075800	1.07338100	-0.18345900
H	2.89285800	-3.04350400	0.35084600
H	1.21977300	-2.77395000	0.83098600
H	1.57703100	-3.81178600	-0.54965500
H	-0.30495400	-1.10179000	-0.95606500
H	0.49390800	-0.85669700	-2.51025200
H	0.06337900	-2.48179000	-1.98335600
H	3.70589800	-2.52447300	-1.91537600
H	2.22591900	-2.92156200	-2.76869500
H	2.90423600	-1.29602600	-2.92063300

D3(BJ)-B3LYP/6-31G*

SCF Done: E(RB3LYP) = -1002.30767717

Zero-point correction= 0.467660
Thermal correction to Gibbs Free Energy= 0.417499

Imaginary frequency --   –141.85

C	-4.21053500	-0.48050700	-1.43656900
C	-4.66714800	-1.12559700	-0.28744600
C	-2.99409800	0.18867700	-1.37639400
H	-4.78001400	-0.49117700	-2.35902500
C	-3.88790400	-1.08837800	0.86693500
H	-5.61325900	-1.65733900	-0.29223300
H	-2.59066200	0.70230700	-2.24202200
N	-2.25741400	0.23045800	-0.25771900
C	-2.67827700	-0.39253500	0.85749100
H	-4.20732200	-1.59560400	1.77120100
C	-0.81514400	1.66393600	0.05234100
C	-1.76803200	-0.34281000	2.05647800
O	-1.28632900	2.42417300	0.83493500
C	-0.26528100	1.90803200	-1.32254400
N	0.31170400	0.53552000	1.00469600
H	-1.84157500	0.63462600	2.54919900
H	-2.10277100	-1.08645100	2.78459100
C	-0.30293400	-0.65267200	1.70687300
H	0.79611200	2.14611200	-1.19143200
H	-0.30645200	0.99511000	-1.91330600
C	-0.98793100	3.08456500	-1.99121100
C	1.71505900	0.48278100	0.55207100
H	0.33012200	1.23660300	1.74670100
H	-0.27975000	-1.48921300	1.01692000
C	0.51729900	-0.97543700	2.95485100
H	-0.56823000	3.24931700	-2.98734100
H	-2.06107100	2.90233800	-2.09444000
H	-0.86277600	3.99798400	-1.40515800
C	2.33148100	-0.44845500	-0.32802400
C	2.44151600	1.57345400	1.06415600
H	1.57064100	-1.13426300	2.71183000
H	0.44966200	-0.16401500	3.68935000
H	0.13437600	-1.88633200	3.42445000
C	3.69048300	-0.18931300	-0.59802500
C	1.73714800	-1.73625600	-0.96020800

C	3.77415900	1.79325800	0.75356500
H	1.94006500	2.27530700	1.72778800
C	4.40658700	0.88828800	-0.08976200
H	4.22334100	-0.86867300	-1.24655200
C	1.79086100	-2.88576400	0.07681400
C	0.31628300	-1.54408300	-1.52590900
C	2.57399500	-2.22154400	-2.17041100
H	4.29970100	2.64573100	1.17041000
H	5.45144600	1.01179500	-0.35549600
H	2.82010000	-3.03524700	0.41806600
H	1.17816700	-2.71487600	0.96270700
H	1.45063800	-3.81796800	-0.38691800
H	-0.38173500	-1.06145300	-0.84887000
H	0.35552300	-0.94550900	-2.44259000
H	-0.10552400	-2.51820600	-1.79483300
H	3.54736300	-2.62676100	-1.88135300
H	2.03056200	-3.03229400	-2.66517900
H	2.72838600	-1.42492000	-2.90519700

B3LYP/6-31G*/SMD (MeCN)

SCF Done: E(RB3LYP) = -1002.26584884

Zero-point correction= 0.465828
Thermal correction to Gibbs Free Energy= 0.415580

Imaginary frequency -- –183.36

C	-4.27005500	-0.61798700	-1.36852600
C	-4.71341400	-1.16959100	-0.16623000
C	-3.05096400	0.05101600	-1.37890500
H	-4.84986100	-0.70090800	-2.28157700
C	-3.91944500	-1.04236200	0.97134400
H	-5.65957800	-1.70035400	-0.11727000
H	-2.66088500	0.49067000	-2.29084100
N	-2.29789400	0.18172200	-0.27579900
C	-2.70742100	-0.35137300	0.89014100
H	-4.22310000	-1.47724500	1.91827900
C	-0.82254400	1.65311700	-0.07580100
C	-1.78704800	-0.20687700	2.07421600
O	-1.32235700	2.46902100	0.63752900
C	-0.29971300	1.81900400	-1.47644200
N	0.29665900	0.62384500	0.94597100
H	-1.86950900	0.79788100	2.50737300
H	-2.10624000	-0.90802400	2.84981000
C	-0.31778100	-0.52029000	1.74599400
H	0.76392500	2.06386900	-1.37800900
H	-0.35789900	0.87876500	-2.02135300
C	-1.02361100	2.95813800	-2.20390100
C	1.71784000	0.53038100	0.51680400
H	0.31924400	1.37005700	1.64476200
H	-0.28027100	-1.41224100	1.12837300
C	0.49731100	-0.73187900	3.02219900
H	-0.61566600	3.05090900	-3.21555300
H	-2.09924400	2.77482900	-2.28403000
H	-0.87710200	3.90995900	-1.68482500
C	2.35965100	-0.45264800	-0.29704300
C	2.44805500	1.63160700	1.00734000
H	1.54701600	-0.94482100	2.80323800
H	0.44641400	0.15258900	3.66889700
H	0.08533900	-1.58362800	3.57280500
C	3.73423000	-0.21795000	-0.52292500
C	1.79790600	-1.77914300	-0.90155200

C	3.79576600	1.82165600	0.73977700
H	1.93646200	2.36485400	1.62590000
C	4.44821800	0.87275900	-0.03746500
H	4.28609100	-0.93359100	-1.11547800
C	1.85886700	-2.89261900	0.17620800
C	0.37613500	-1.65429100	-1.48527100
C	2.66170700	-2.28152400	-2.08889200
H	4.31437300	2.68703800	1.14100100
H	5.50519600	0.96888900	-0.26912800
H	2.88236600	-3.00611900	0.55174200
H	1.21059900	-2.70366100	1.03480900
H	1.55357400	-3.84856700	-0.26640000
H	-0.34992700	-1.19830800	-0.81716700
H	0.39341000	-1.06706000	-2.41009000
H	0.00350200	-2.65328500	-1.73992400
H	3.64014900	-2.66215900	-1.78070500
H	2.13522500	-3.11421500	-2.56812700
H	2.81176800	-1.50212300	-2.84436900

M06-2X/6-31G*

SCF Done: E(RM062X) = -1001.74057951

Zero-point correction= 0.471487
Thermal correction to Gibbs Free Energy= 0.422018

Imaginary frequency --   –199.70

C	-4.13530200	-0.50703400	-1.45559000
C	-4.62594400	-1.12097800	-0.30710400
C	-2.91513000	0.14773900	-1.38152300
H	-4.68201900	-0.52925700	-2.39074900
C	-3.87231200	-1.07615400	0.86037500
H	-5.57936600	-1.63858700	-0.32344600
H	-2.48527300	0.63858100	-2.24864200
N	-2.20748900	0.20382000	-0.24761000
C	-2.65693500	-0.39782000	0.86447100
H	-4.21373200	-1.56569700	1.76616000
C	-0.86537300	1.57570400	0.04848900
C	-1.75321500	-0.35830500	2.06699300
O	-1.33952900	2.34343500	0.82680500
C	-0.32270100	1.86876500	-1.32526500
N	0.27375100	0.53462600	0.97117600
H	-1.82555300	0.61476900	2.56869000
H	-2.08291700	-1.11558400	2.78330000
C	-0.29708000	-0.65979900	1.68979400
H	0.72553200	2.15703400	-1.19241400
H	-0.32312700	0.96832300	-1.94004100
C	-1.09956900	3.03052300	-1.94962600
C	1.68218500	0.51570800	0.52212100
H	0.28194600	1.23602300	1.71655700
H	-0.28249300	-1.50157500	1.00153500
C	0.55138000	-0.95818600	2.92008100
H	-0.72132800	3.22863000	-2.95451800
H	-2.17078300	2.82422800	-2.01530200
H	-0.97863800	3.93164700	-1.34607000
C	2.33257900	-0.42089400	-0.32036300
C	2.36946400	1.63824100	1.00991400
H	1.60381800	-1.09536300	2.65906000
H	0.47692500	-0.14287800	3.64895100
H	0.19601600	-1.87357200	3.40079300
C	3.68676500	-0.13744500	-0.57621800
C	1.77393400	-1.73049500	-0.92768200

C	3.69824000	1.88354000	0.71153400
H	1.83892700	2.34858900	1.64191400
C	4.36638100	0.97061100	-0.09036700
H	4.24681900	-0.82200900	-1.19777300
C	1.84041100	-2.85675900	0.12598000
C	0.36211400	-1.56601700	-1.51355700
C	2.63049900	-2.22210700	-2.11431100
H	4.19386500	2.76273100	1.10769600
H	5.41169700	1.11248400	-0.34272500
H	2.87020900	-2.97513900	0.47744000
H	1.21571200	-2.69013700	1.00470100
H	1.52884800	-3.80267000	-0.32924000
H	-0.33672400	-1.03969900	-0.86775500
H	0.41458300	-1.01714000	-2.46053400
H	-0.06221000	-2.55165400	-1.73205500
H	3.60114400	-2.61623300	-1.80307900
H	2.09896900	-3.04266400	-2.60526600
H	2.78921500	-1.43279800	-2.85546300

PBE0/def2-TZVPP

SCF Done: E(RPBE1PBE) = -1001.35017471

Zero-point correction= 0.465482
Thermal correction to Gibbs Free Energy= 0.415236

Imaginary frequency -- –166.85

C	-4.24001100	-0.45793800	-1.41371300
C	-4.70824600	-1.07411100	-0.26385900
C	-3.00210800	0.15287300	-1.37081000
H	-4.81891200	-0.44635500	-2.32719900
C	-3.91908000	-1.06667600	0.87324700
H	-5.67517000	-1.56171200	-0.25535800
H	-2.59362800	0.64597600	-2.24444900
N	-2.25502200	0.16755200	-0.26968200
C	-2.68743800	-0.42828800	0.84253200
H	-4.24773400	-1.55442800	1.78213700
C	-0.82466500	1.57991800	0.04015600
C	-1.76855000	-0.39931100	2.02169900
O	-1.31692100	2.33999700	0.80320400
C	-0.31114100	1.84461400	-1.33898000
N	0.27396500	0.53147500	0.96323100
H	-1.85920100	0.55963400	2.54295900
H	-2.07845800	-1.16772000	2.73043000
C	-0.31128400	-0.66622900	1.65468500
H	0.75582600	2.05883900	-1.23131800
H	-0.37810700	0.94721000	-1.94835800
C	-1.01539000	3.03865600	-1.96639100
C	1.68337700	0.50736600	0.53857200
H	0.28104300	1.22397500	1.70976100
H	-0.27214700	-1.49753100	0.95824600
C	0.50931900	-0.97676300	2.89329800
H	-0.61411200	3.21520300	-2.96428500
H	-2.09123000	2.88582300	-2.05221500
H	-0.86117900	3.93663900	-1.36997100
C	2.34850400	-0.41037200	-0.30848100
C	2.36484900	1.60951500	1.06491000
H	1.56449800	-1.11337900	2.65923500
H	0.42115400	-0.17566100	3.63222900
H	0.14450400	-1.89498600	3.35480000
C	3.69976800	-0.11368700	-0.53523200
C	1.82357000	-1.71298500	-0.95312400

C	3.69349100	1.86216200	0.80160600
H	1.82705800	2.30070800	1.70782700
C	4.36986900	0.97649300	-0.01300900
H	4.26778400	-0.77955100	-1.16528700
C	1.86941600	-2.85164800	0.07945400
C	0.43099300	-1.57390800	-1.57224000
C	2.71756100	-2.17191500	-2.11675000
H	4.18154300	2.72691000	1.23166000
H	5.41668100	1.12400000	-0.24653500
H	2.88458300	-2.97448700	0.46108700
H	1.21710900	-2.70297400	0.93733300
H	1.57721400	-3.78926400	-0.39825700
H	-0.31867800	-1.13878400	-0.91913300
H	0.48228600	-0.96281900	-2.47593900
H	0.06989100	-2.55960100	-1.87232900
H	3.68993700	-2.54000000	-1.78896200
H	2.22298400	-3.00405500	-2.62039800
H	2.87039500	-1.38139300	-2.85361100

D

B3LYP/6-31G*

SCF Done: E(RB3LYP) = -1002.17688301

Zero-point correction=   0.466750
Thermal correction to Gibbs Free Energy=   0.416473

C	4.17161800	-1.34588200	-0.92911300
C	4.74282600	-1.08544700	0.31842200
C	4.00278500	-0.39657700	1.27554000
C	2.71790600	0.05886700	0.97567800
C	2.89882500	-0.87042600	-1.18935300
H	4.70021800	-1.90678600	-1.69147300
H	5.74439600	-1.43364100	0.55124500
H	4.40199600	-0.22621700	2.27037000
H	2.34078700	-1.02369300	-2.10692400
C	0.83044000	0.46595000	-0.84530800
C	1.84613000	0.64544400	2.06100900
H	1.72510300	-0.15018700	2.80999900
H	2.39570000	1.44286900	2.57373400
C	0.45072300	1.18537200	1.69542300
H	0.54152000	2.21810700	1.36018700
C	-0.46634400	1.12236500	2.91895700
H	-1.42782900	1.60286400	2.73483200
H	-0.64744700	0.08609000	3.22665300
H	0.01852100	1.63940600	3.75382400
N	-0.11058800	0.41438200	0.51302800
O	0.31286500	-0.39333400	-1.78422800
C	1.11189400	1.90633100	-1.27412300
H	0.13560400	2.36612200	-1.44797300
H	1.61840400	2.47884200	-0.48665200
C	1.91719300	1.97669300	-2.57742300
H	1.98146500	3.01593300	-2.91302900
H	2.94196600	1.60962600	-2.45373600
H	1.42197400	1.38523800	-3.35031700
C	-1.53257100	0.69602800	0.17699200
C	-2.46125300	-0.33770800	-0.06678100
C	-1.88882700	2.05070100	0.09764300
C	-3.75248500	0.10592800	-0.42820000
C	-3.17647600	2.43373500	-0.24737000
H	-1.15786000	2.82108600	0.31414900
C	-4.11561200	1.44219600	-0.52322800
H	-4.50840100	-0.63751100	-0.65015900

H	-3.43583300	3.48605400	-0.30186900
H	-5.12971500	1.70583000	-0.80730500
C	-2.28800700	-1.88579100	-0.01181600
C	-2.49289400	-2.43815400	-1.44552600
H	-3.48300200	-2.19560700	-1.84183500
H	-1.73892700	-2.02214700	-2.11973000
H	-2.39793400	-3.53021300	-1.43989300
C	-0.92574000	-2.43063300	0.47376700
H	-0.11740800	-2.16559900	-0.21640900
H	-0.69108800	-2.13856200	1.51007000
H	-0.97447700	-3.52411200	0.49077400
C	-3.35468000	-2.47432800	0.94823400
H	-4.37808000	-2.24976600	0.63878200
H	-3.25759700	-3.56495900	0.97949700
H	-3.22428900	-2.09456300	1.96864700
N	2.21892300	-0.15931400	-0.26155900
H	-0.07173600	-0.58129300	0.73755200

8) ^{1}H and ^{13}C NMR Spectra

N-(2-(*tert*-Butyl)phenyl)-1-phenylmethanimine (S1)

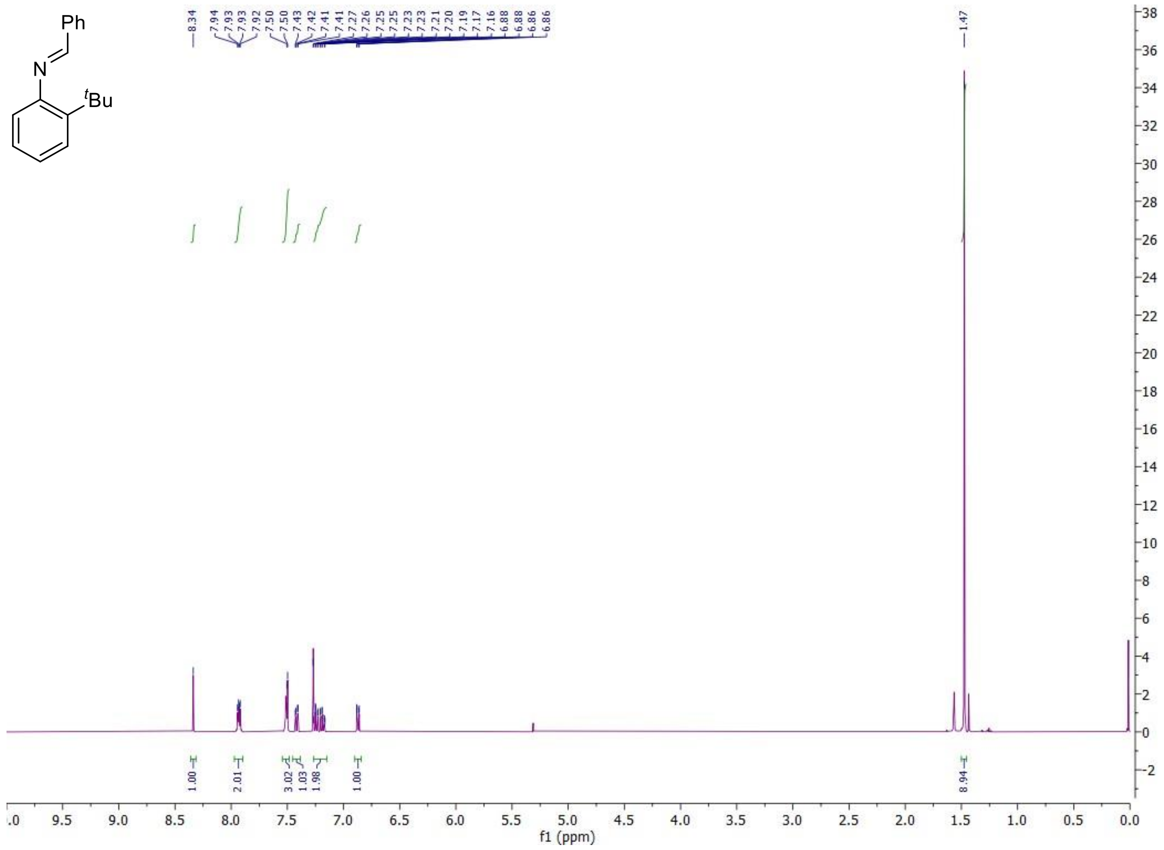

2-(*tert*-Butyl)-*N*-(1-phenyl-2-(pyridin-2-yl)ethyl)aniline (4a)

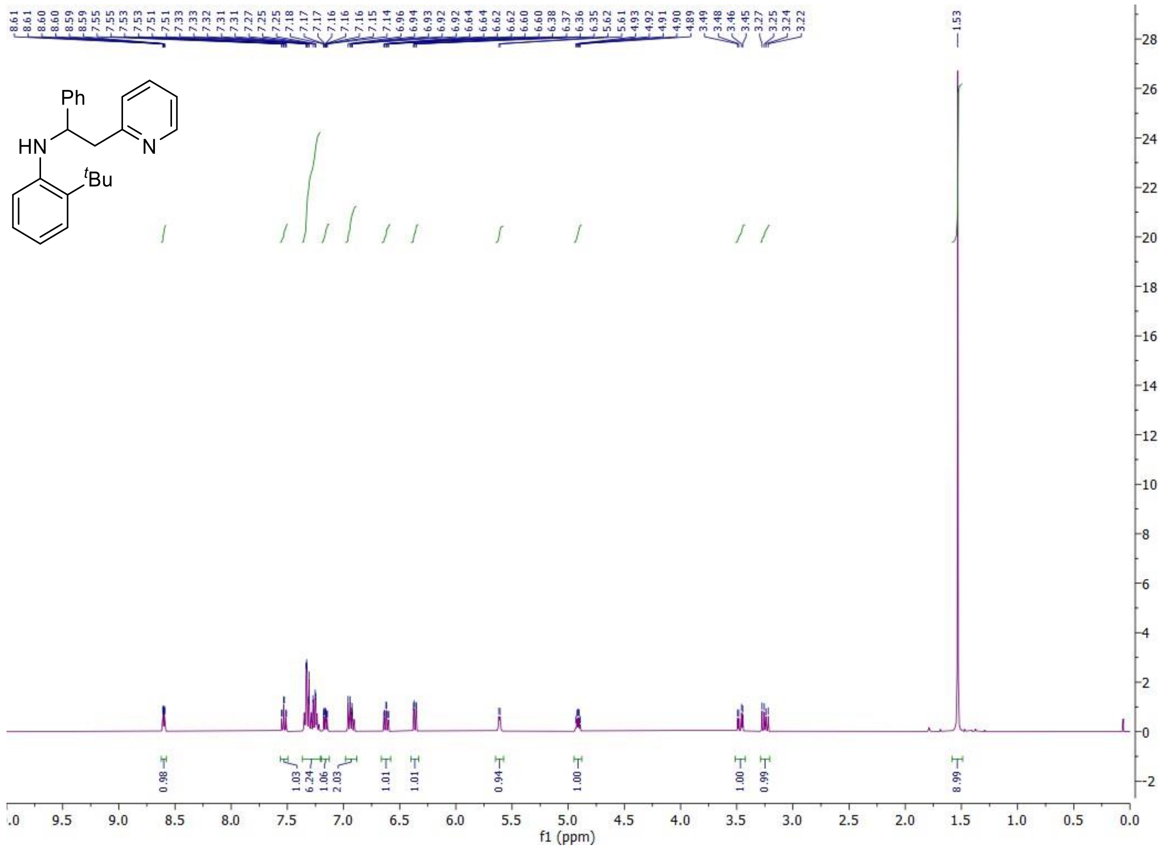

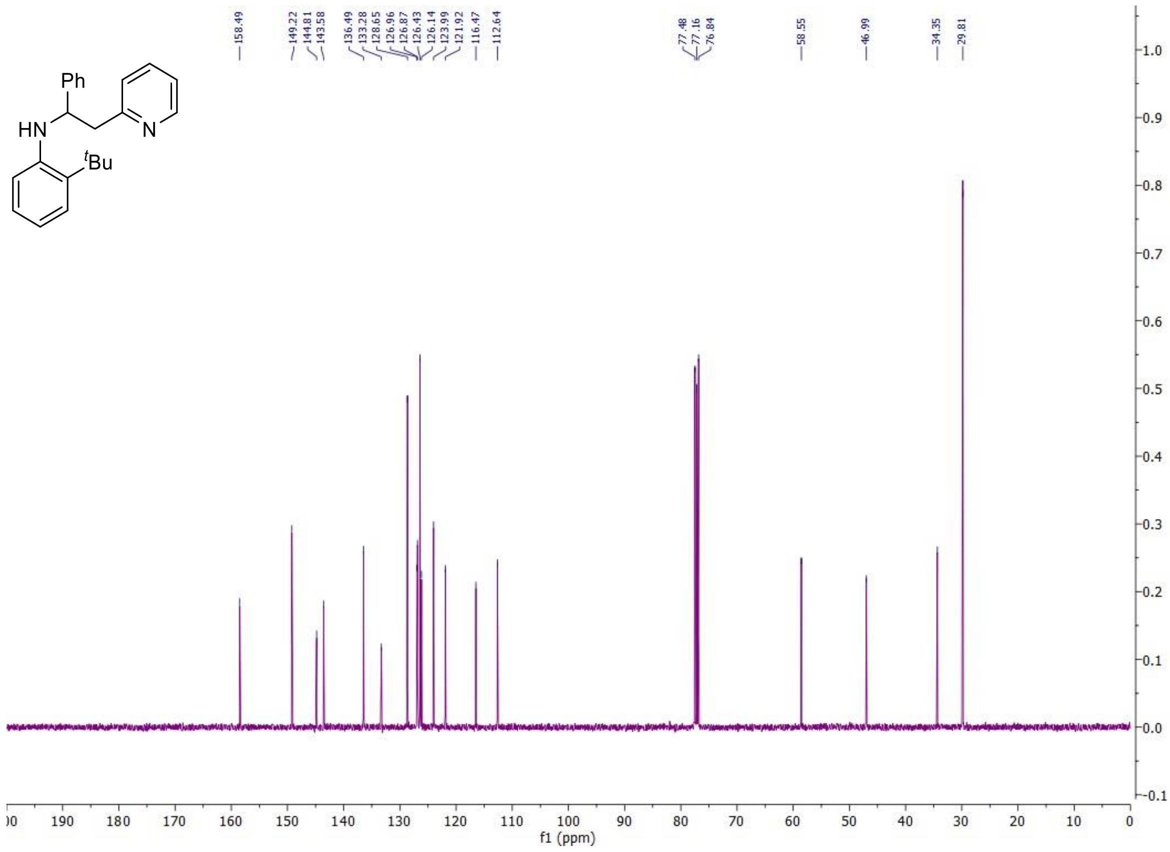

_N_-(2-(_tert_-Butyl)phenyl)-_N_-(1-phenyl-2-(pyridin-2-yl)ethyl)propionamide (6a)

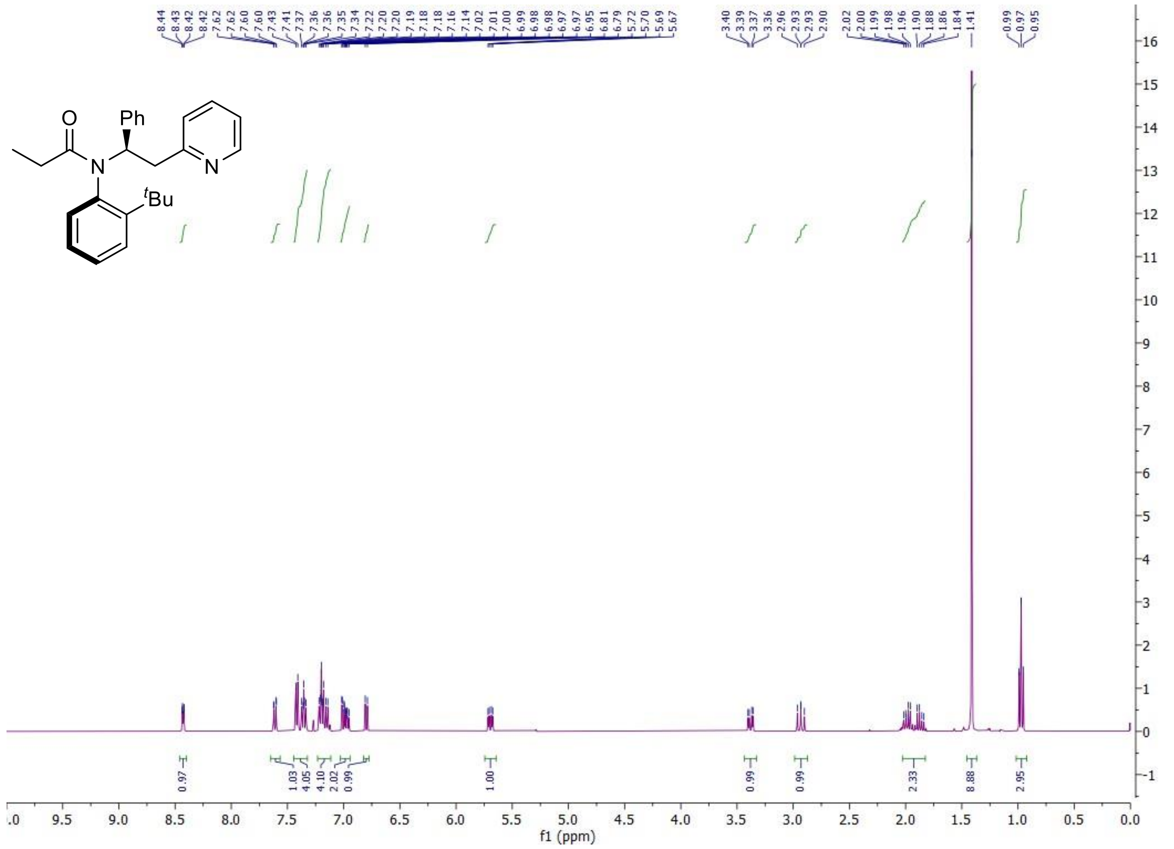

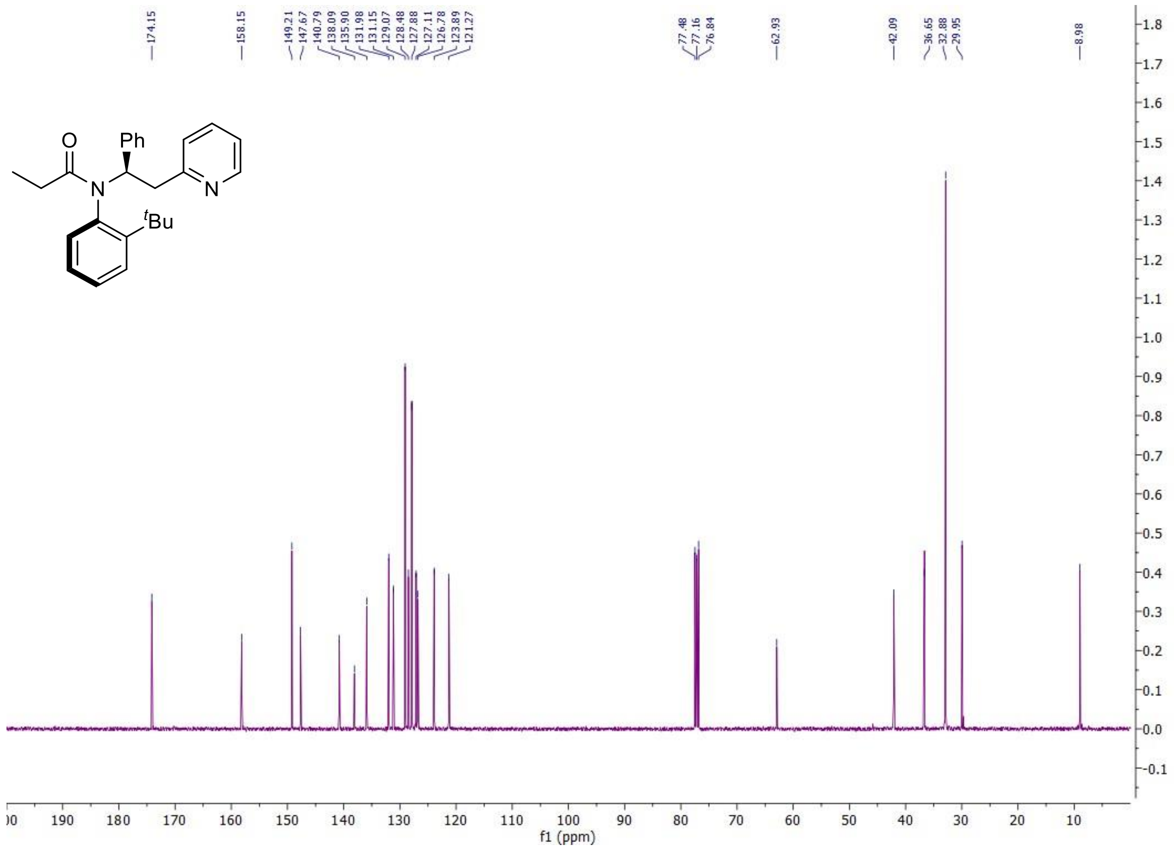

***N*-(2-(*tert*-Butyl)phenyl)-*N*-(1-phenyl-2-(pyridin-2-yl)ethyl)propionamide (11a)**

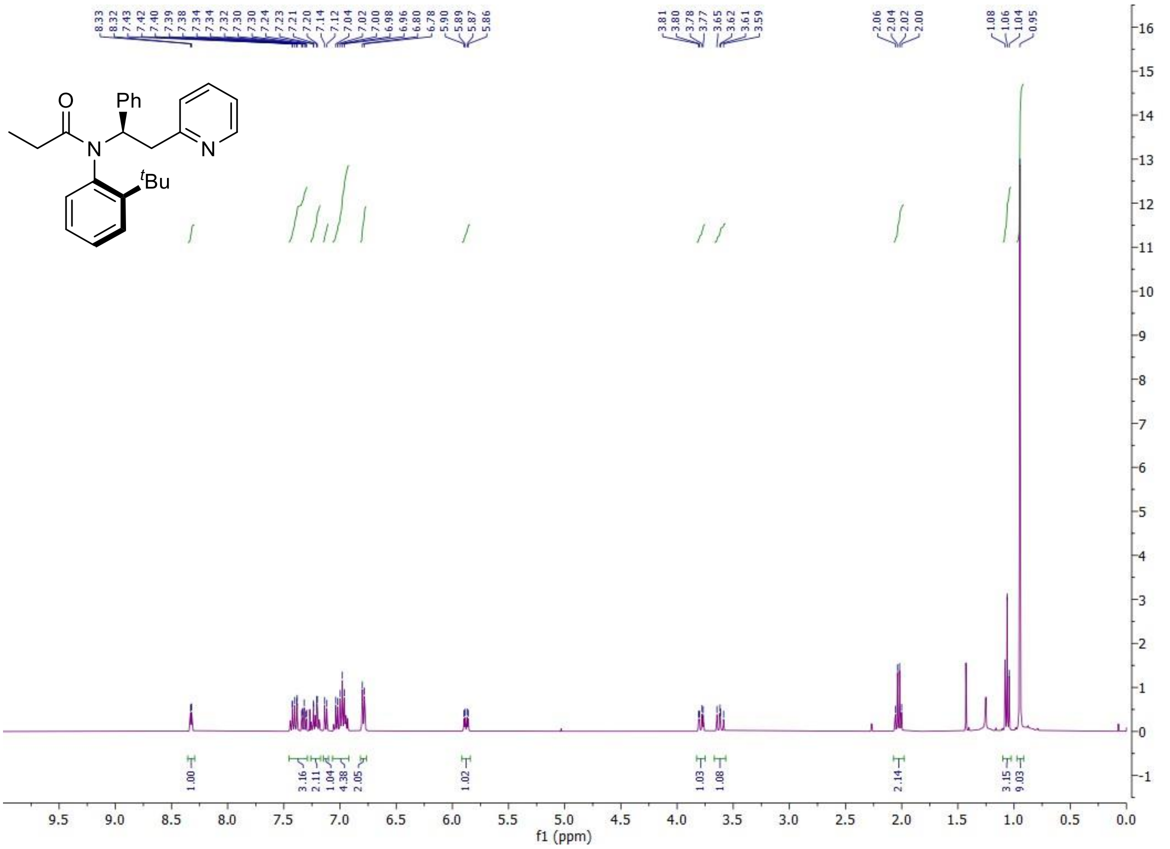

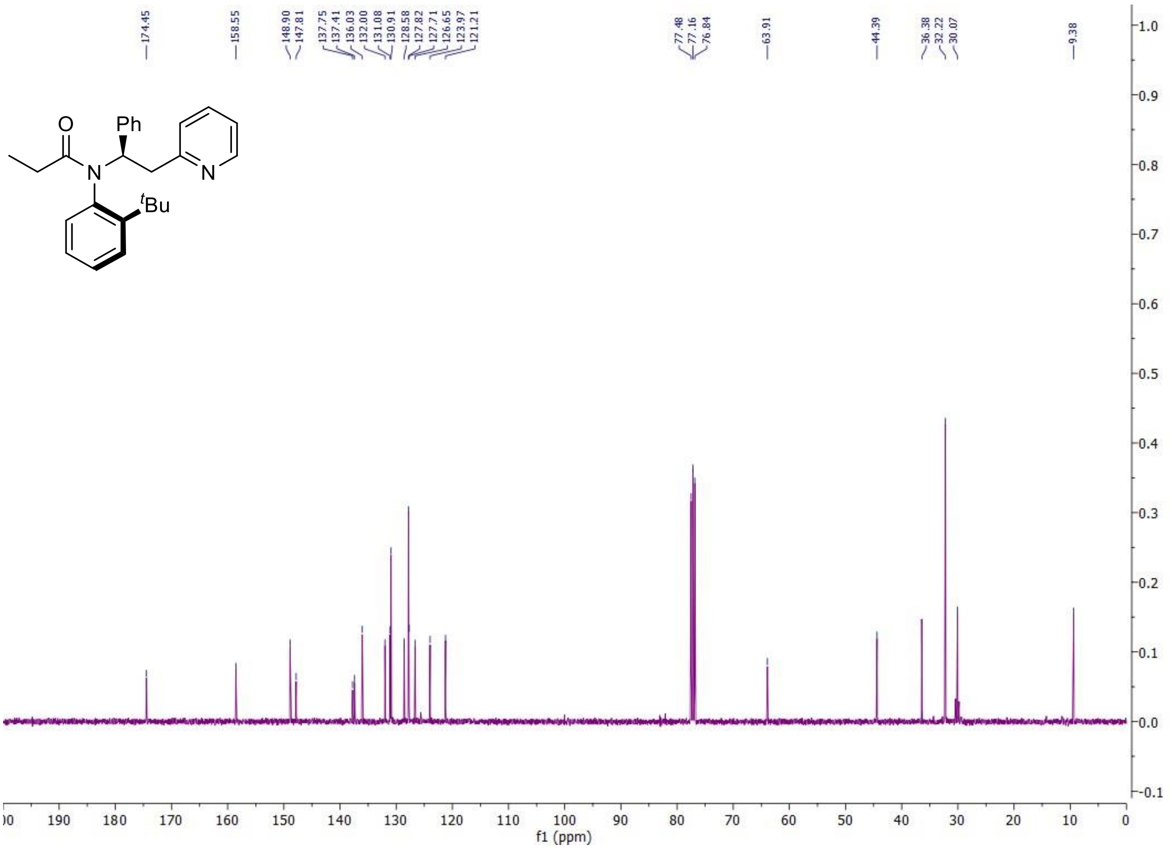

2-(*tert*-Butyl)-*N*-(1,2-diphenylethyl)aniline (12)

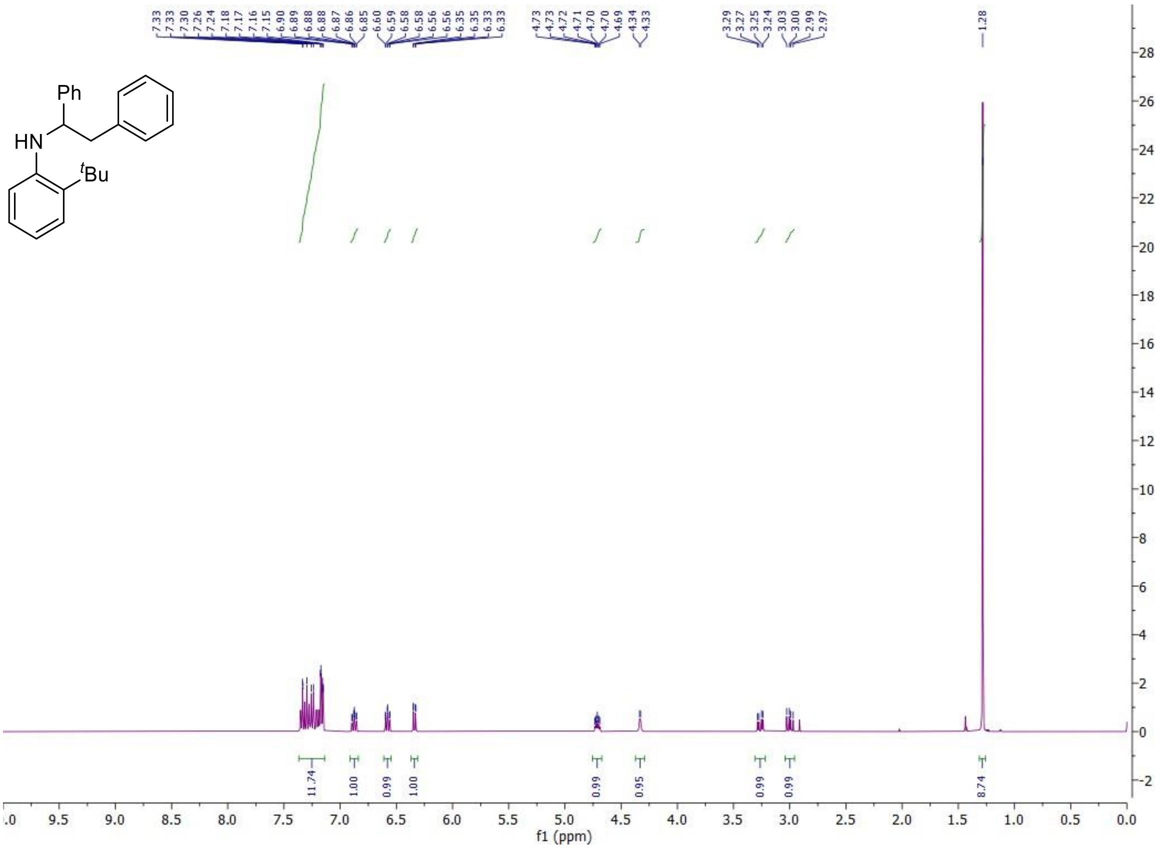

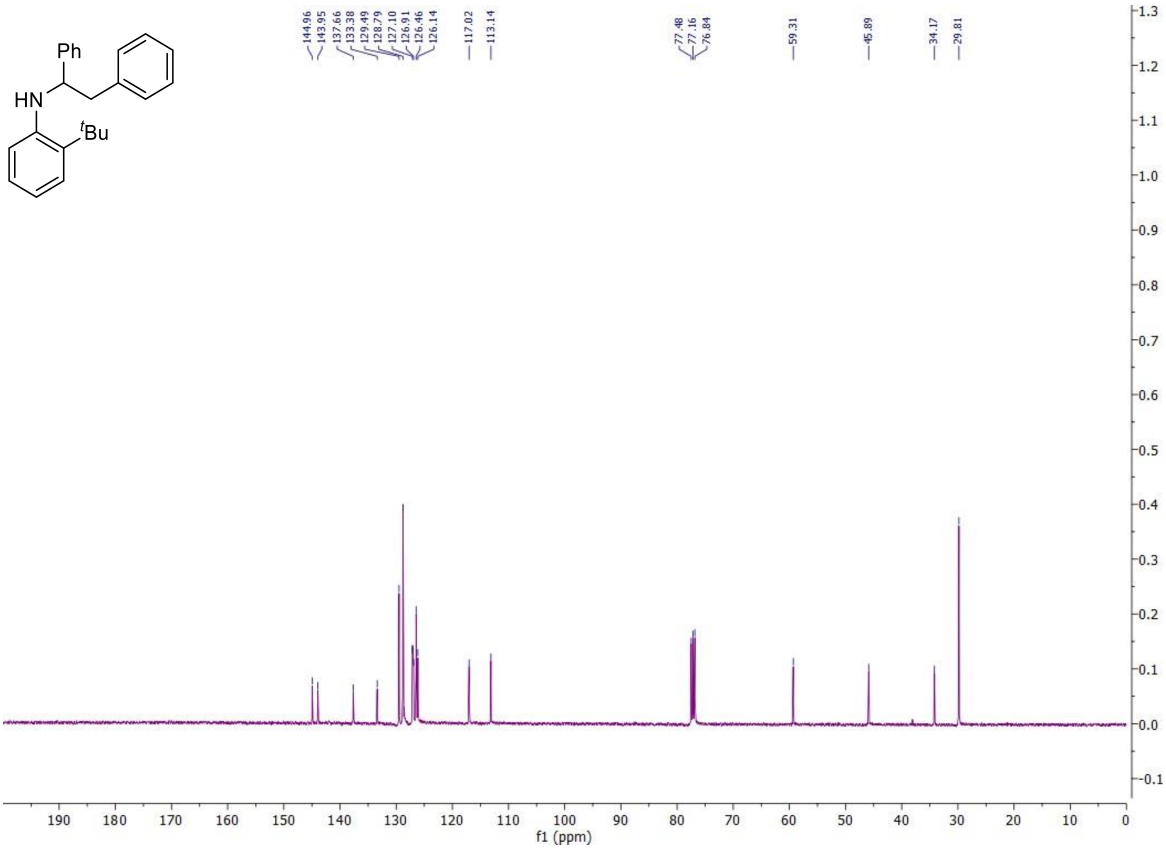

N-(2-(*tert*-Butyl)phenyl)-*N*-(1,2-diphenylethyl)propionamide (13)

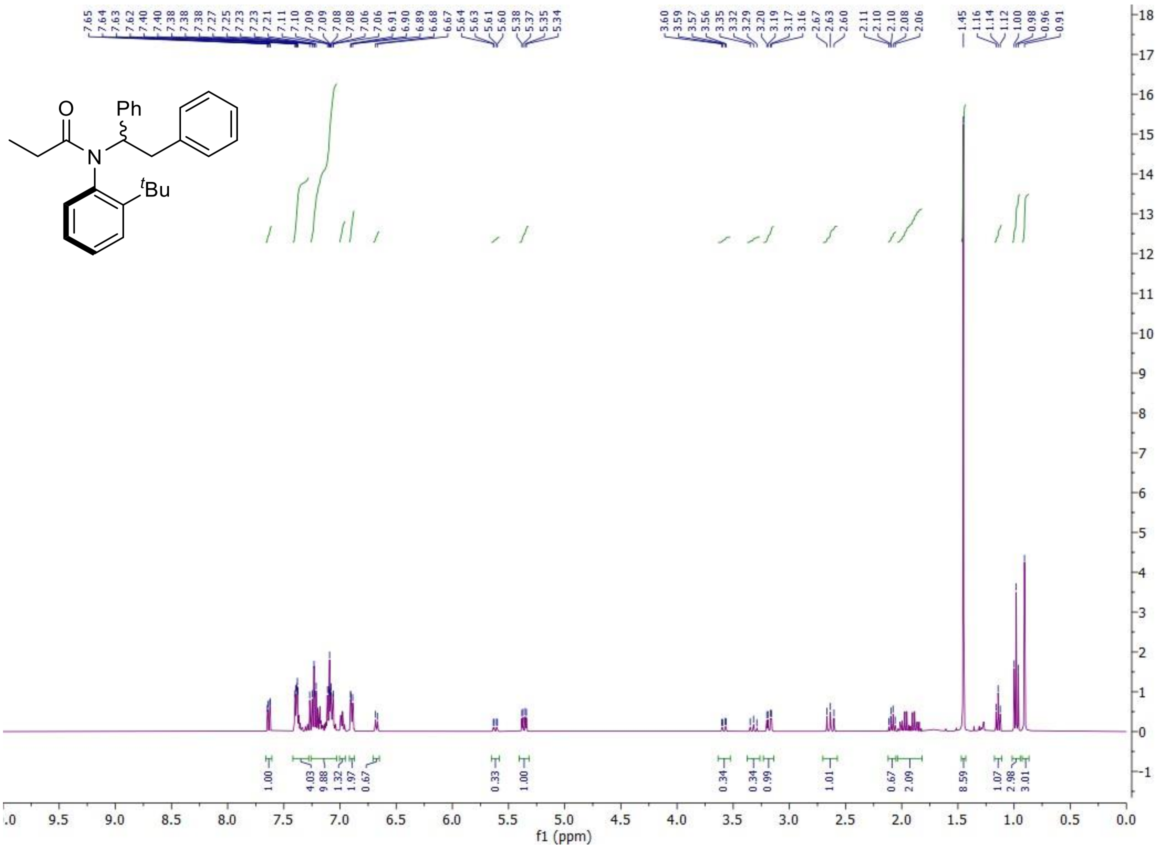

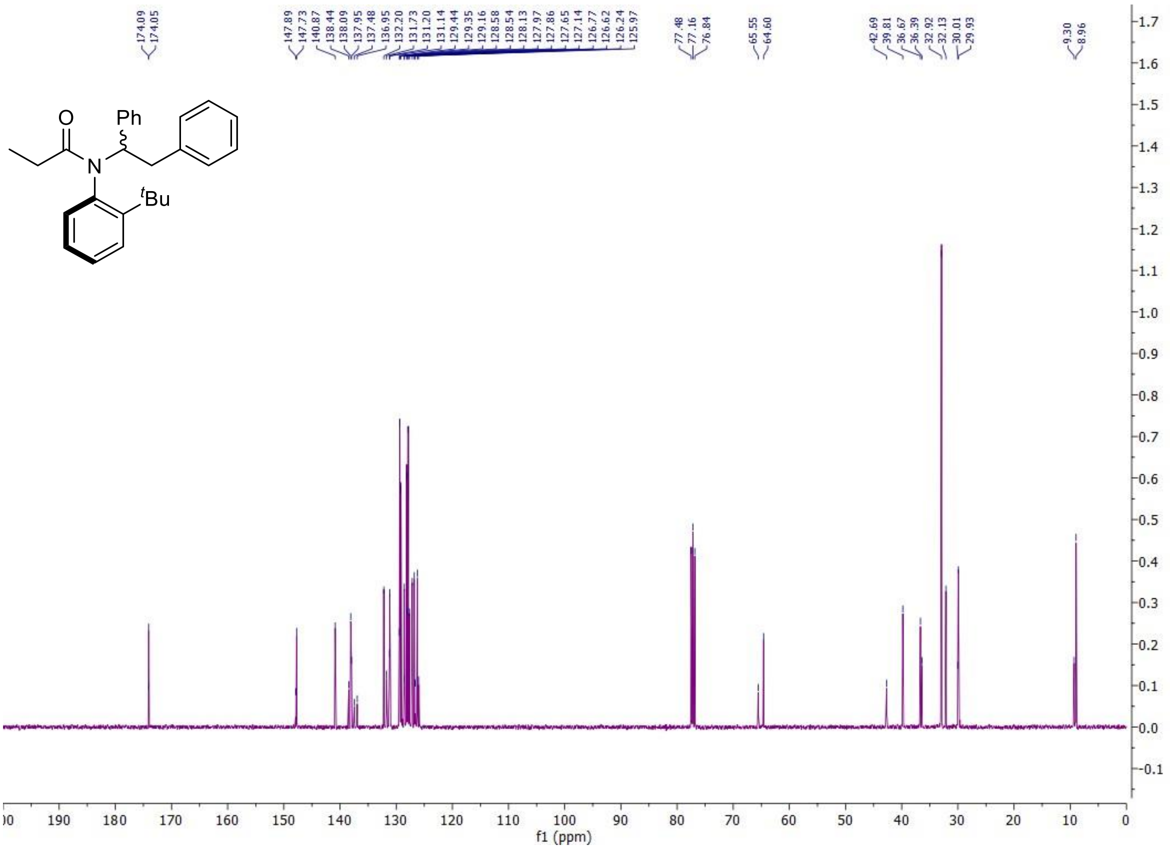

1-(Pyridin-2-yl)propan-2-one (S2) – mixture of keto and enol tautomers

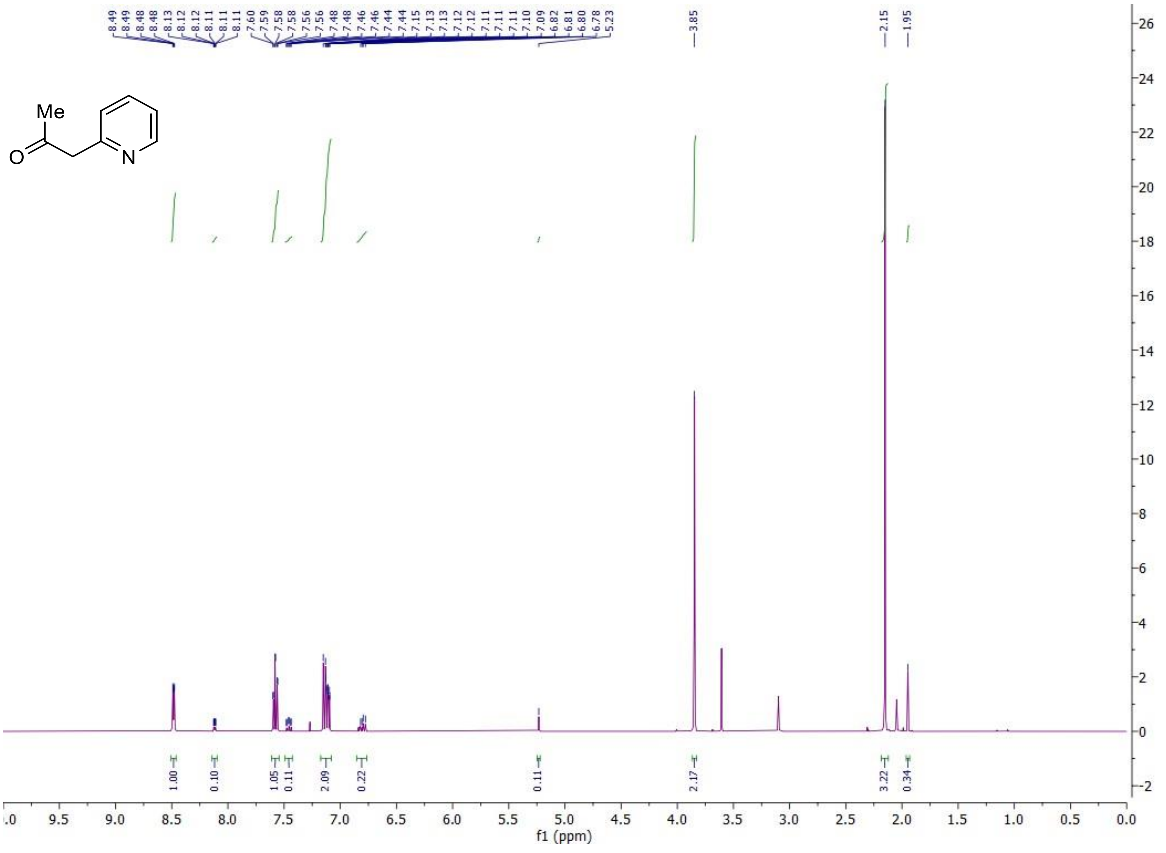

2-(*tert*-Butyl)-*N*-(1-(pyridin-2-yl)propan-2-yl)aniline (4b)

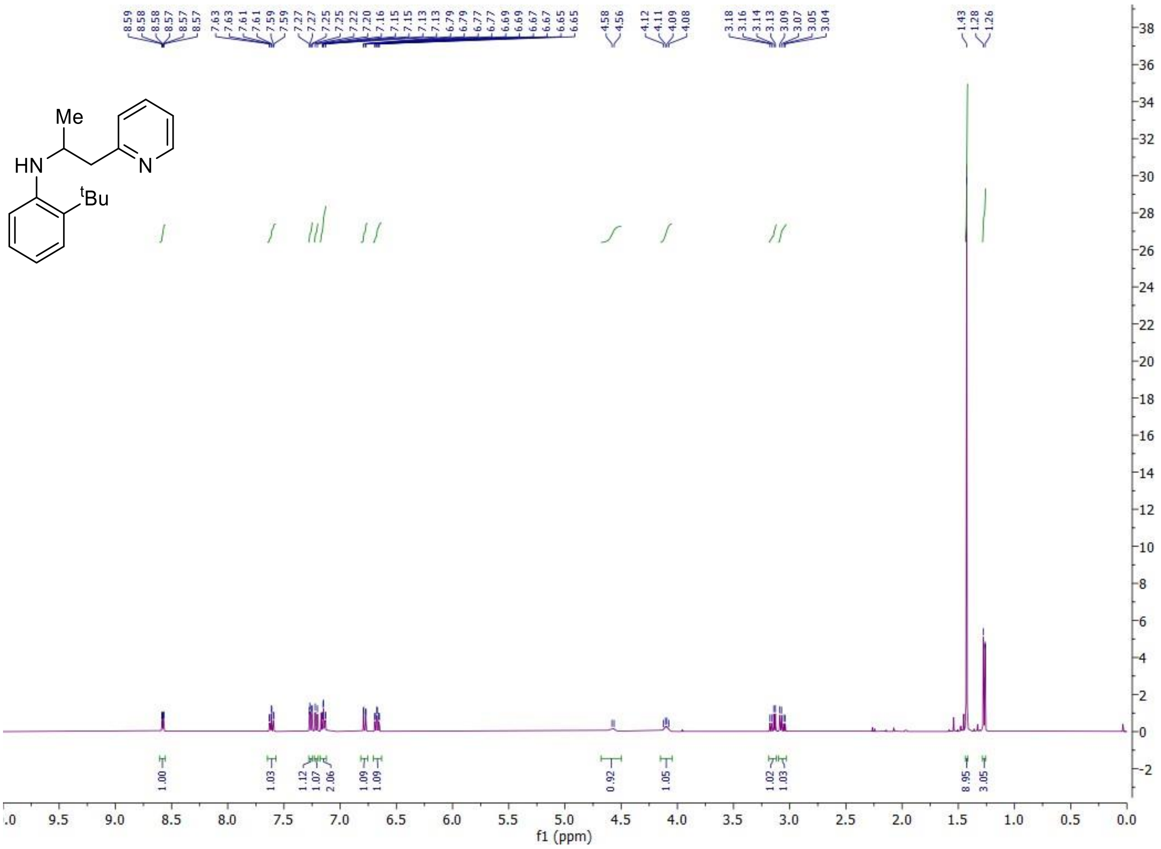

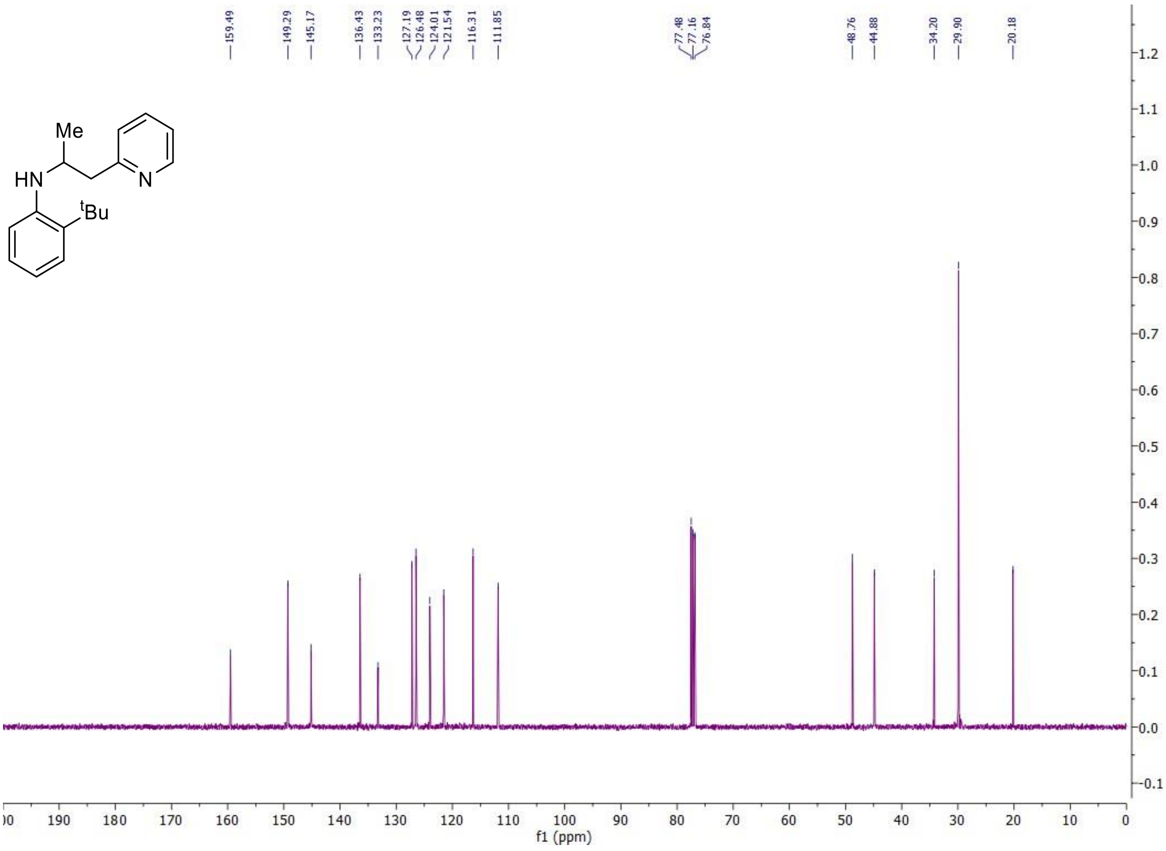

_N_-(2-(_tert_-Butyl)phenyl)-_N_-(1-phenyl-2-(pyridin-2-yl)ethyl)furan-2-carboxamide (6b)

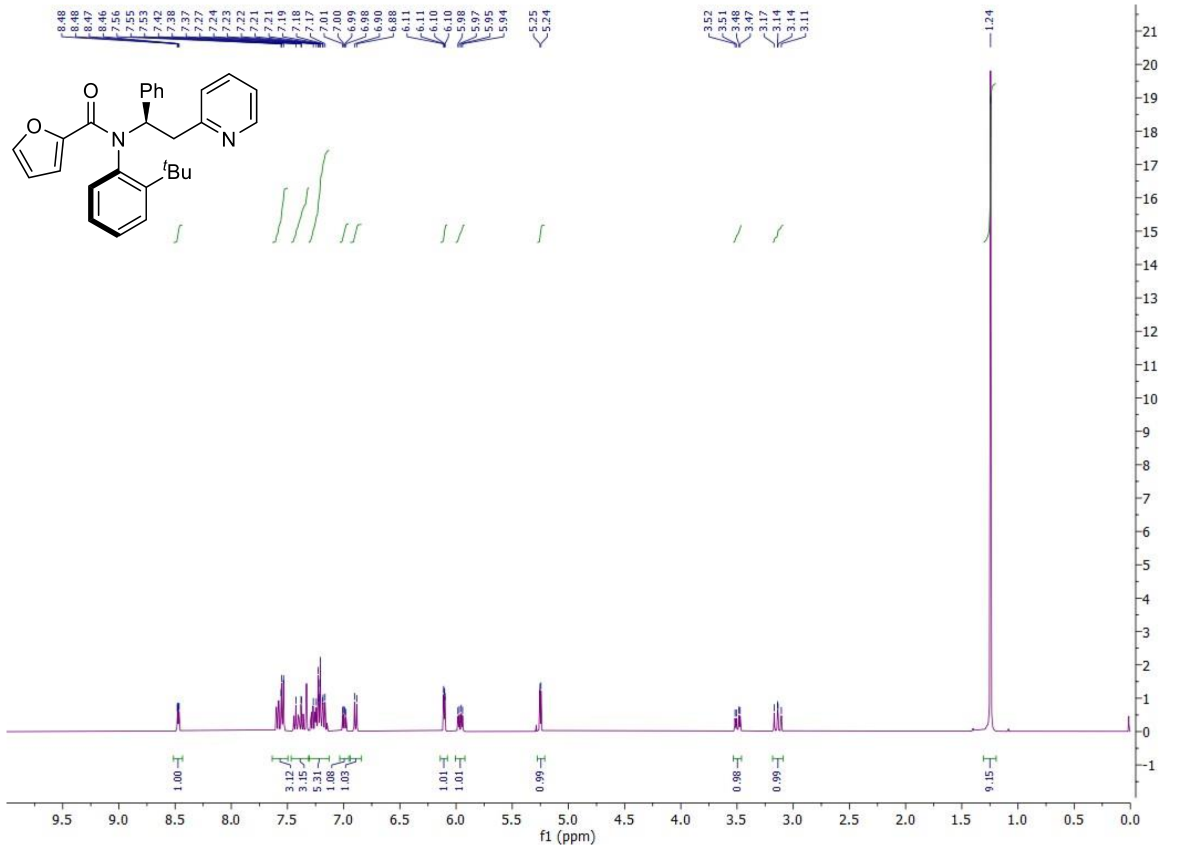

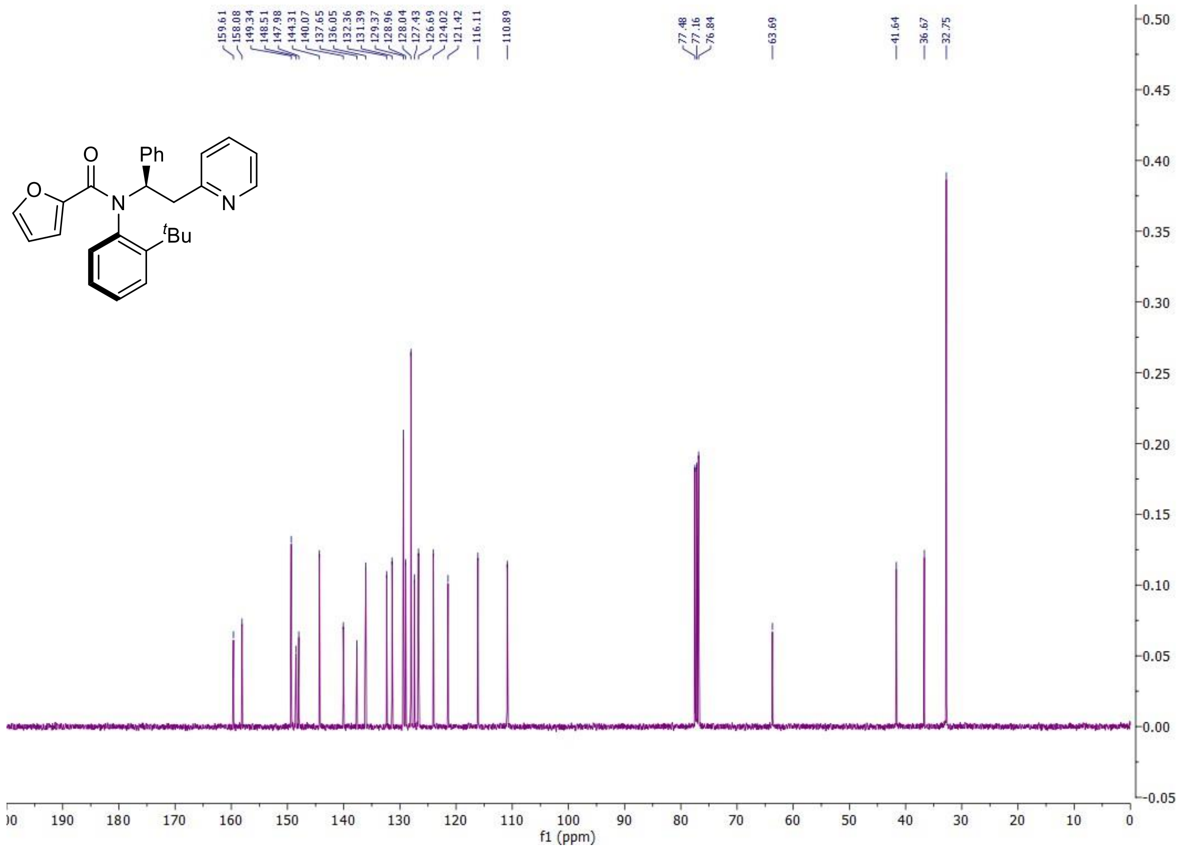

***N*-(2-(*tert*-Butyl)phenyl)-*N*-(1-phenyl-2-(pyridin-2-yl)ethyl)hexanamide (6c)**

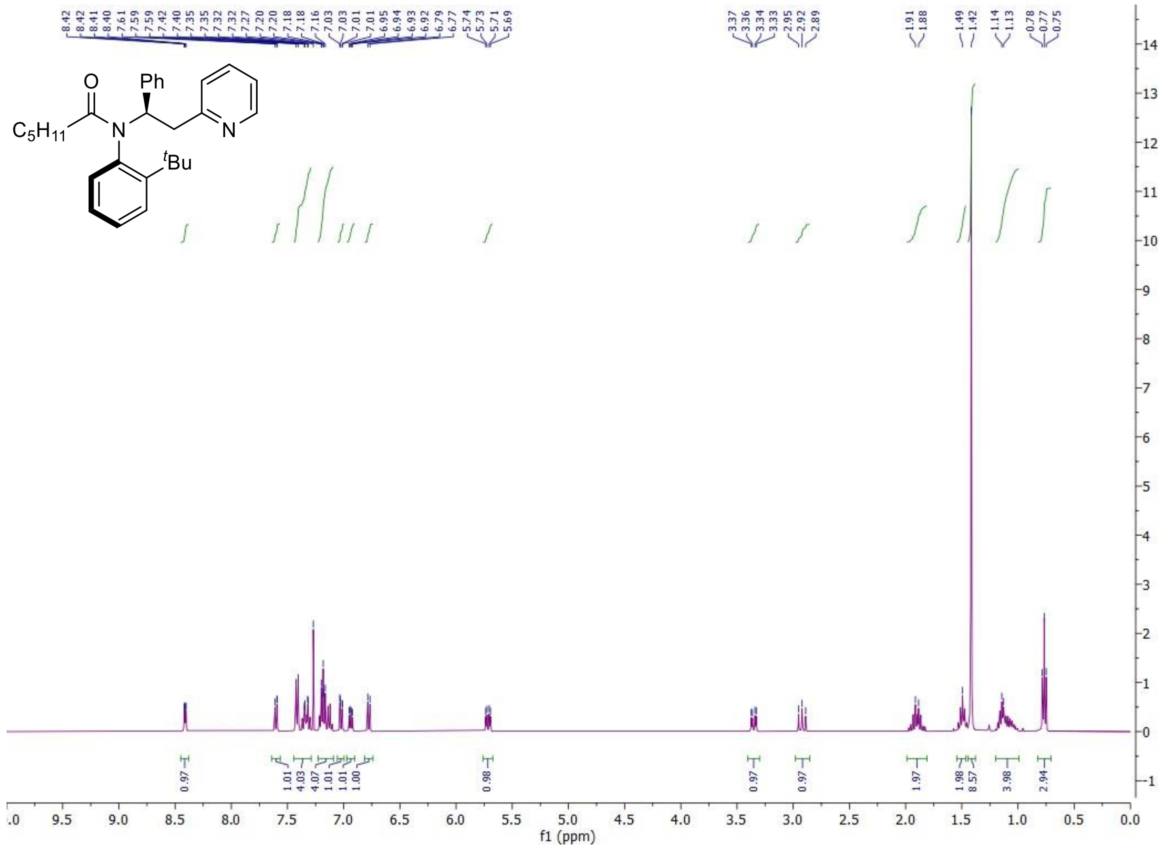

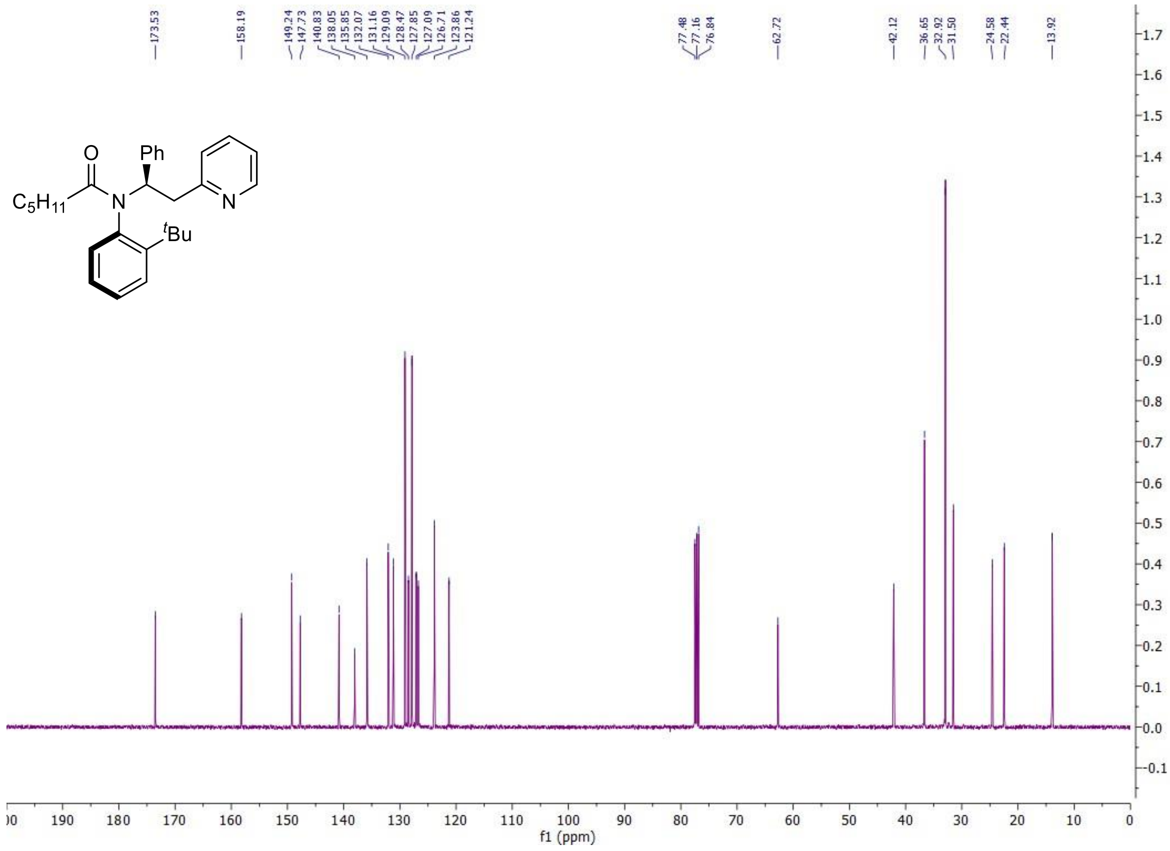

***N*-(2-(*tert*-Butyl)phenyl)-*N*-(1-phenyl-2-(pyridin-2-yl)ethyl)acrylamide (6d)**

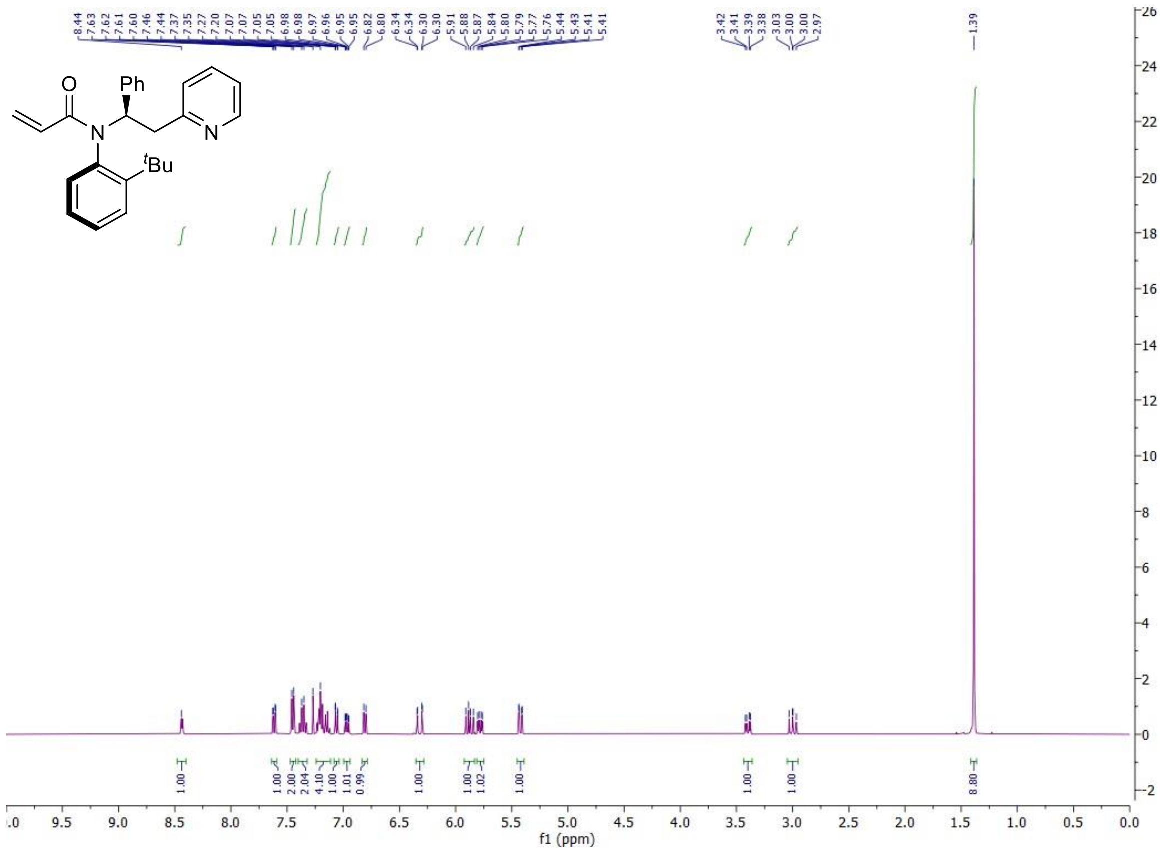

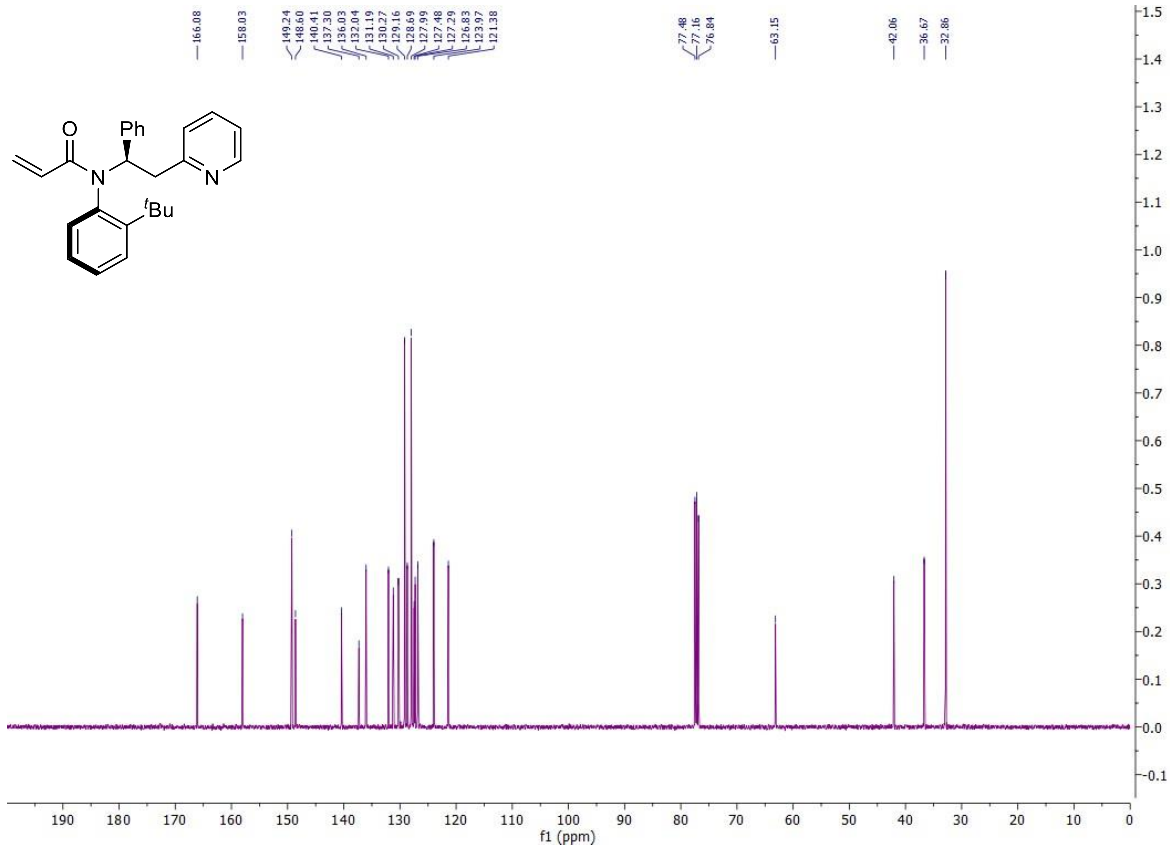

N-(2-(tert-Butyl)phenyl)-N-(1-phenyl-2-(pyridin-2-yl)ethyl)benzamide (6e)

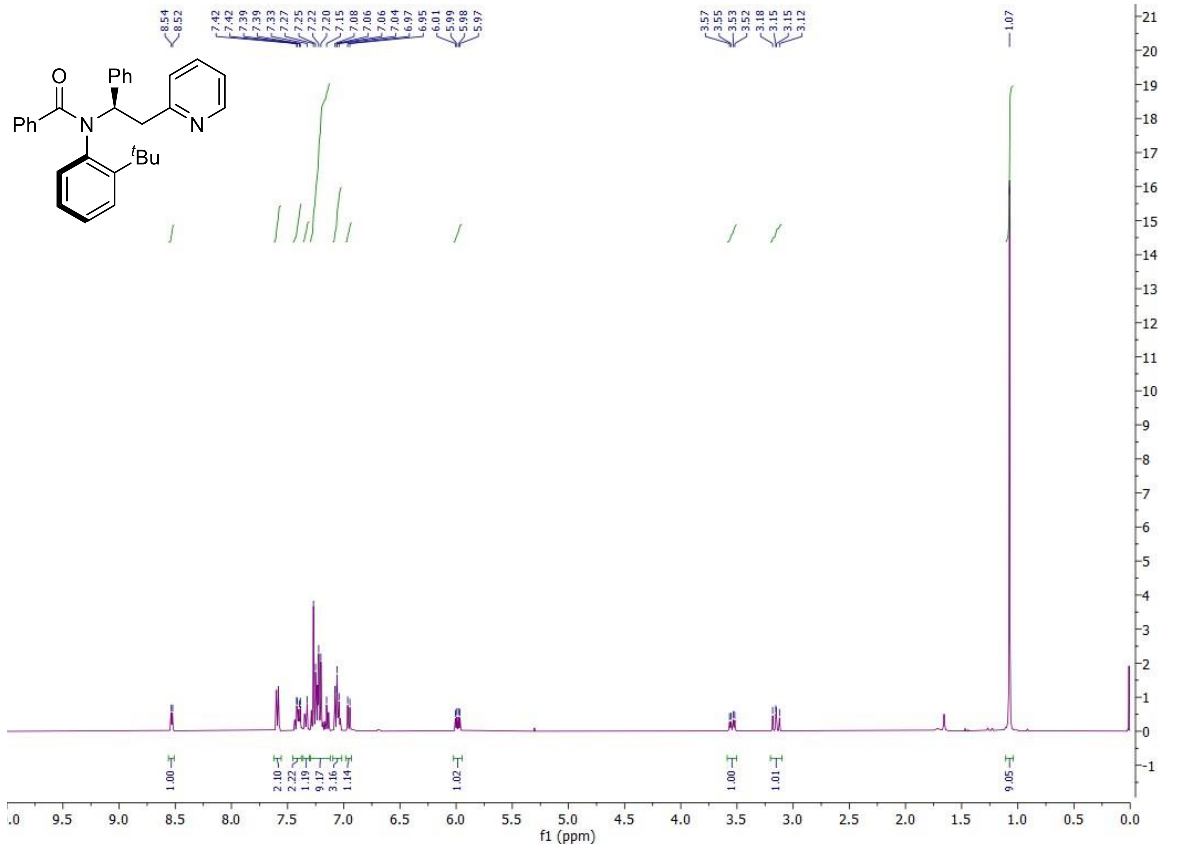

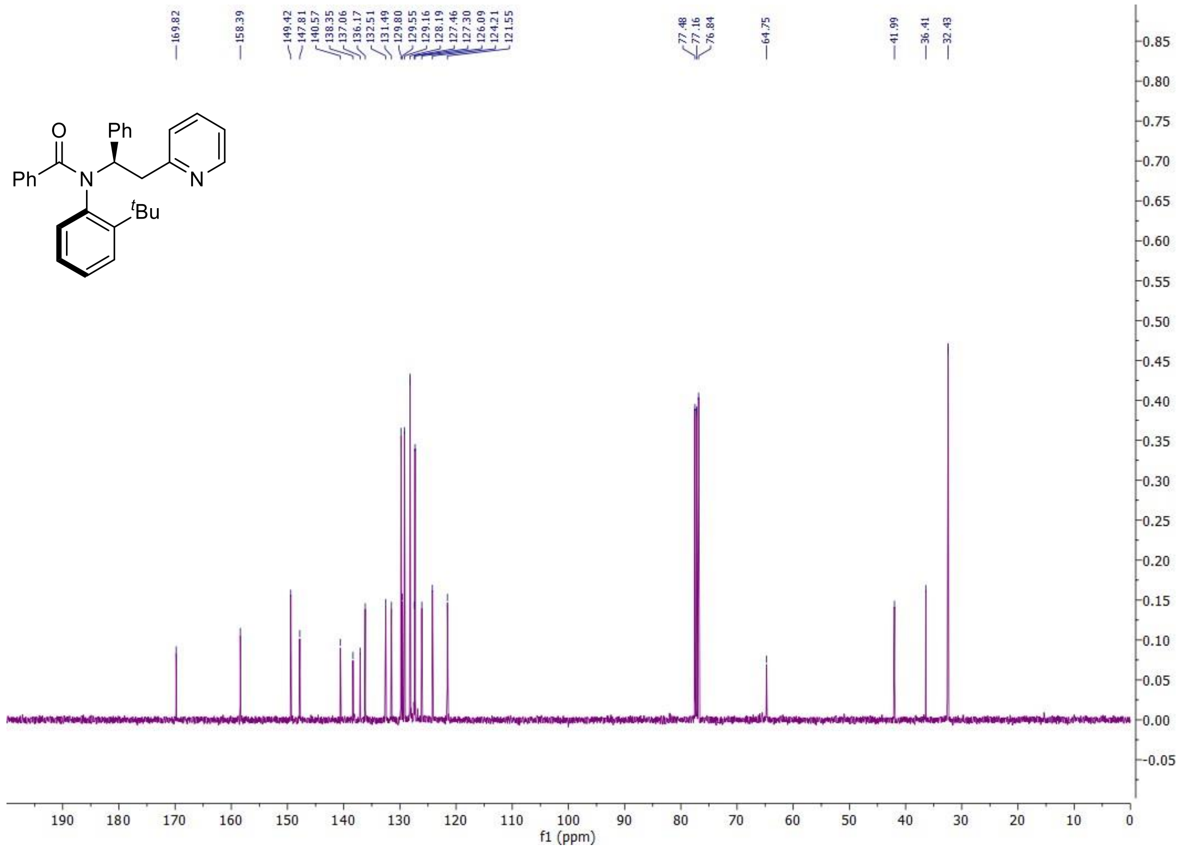

4-Bromo-*N*-(2-(*tert*-butyl)phenyl)-*N*-(1-phenyl-2-(pyridin-2-yl)ethyl)benzamide (6f)

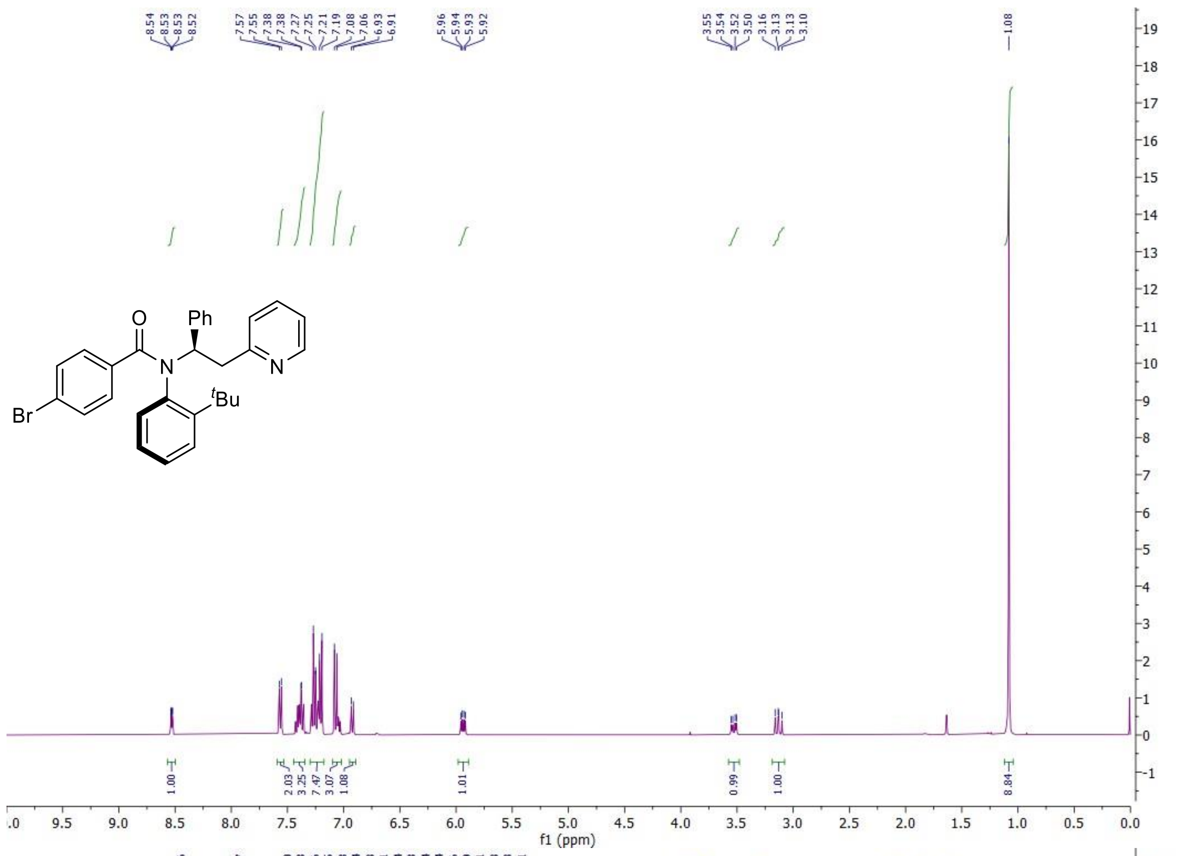

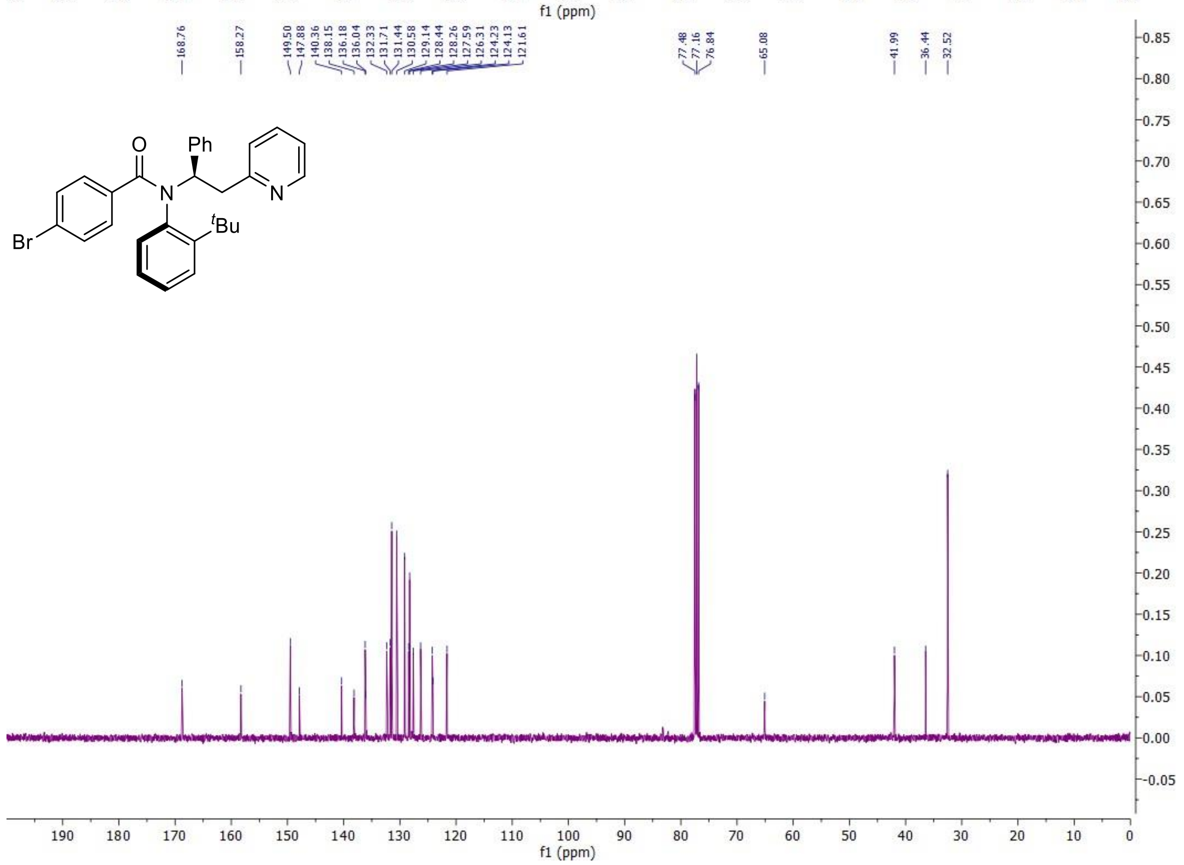

***N*-(2-(*tert*-Butyl)phenyl)-*N*-(1-phenyl-2-(pyridin-2-yl)ethyl)-3-(trifluoromethyl)benzamide (6g)**

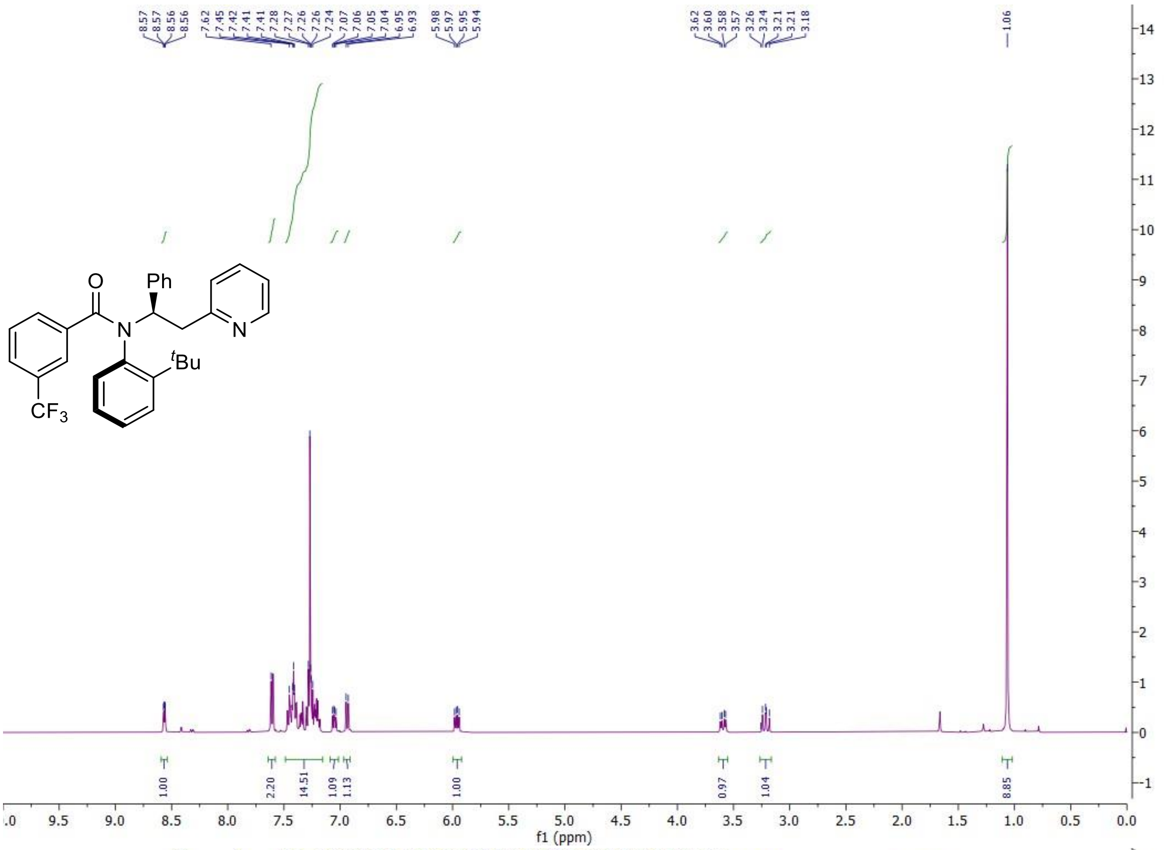

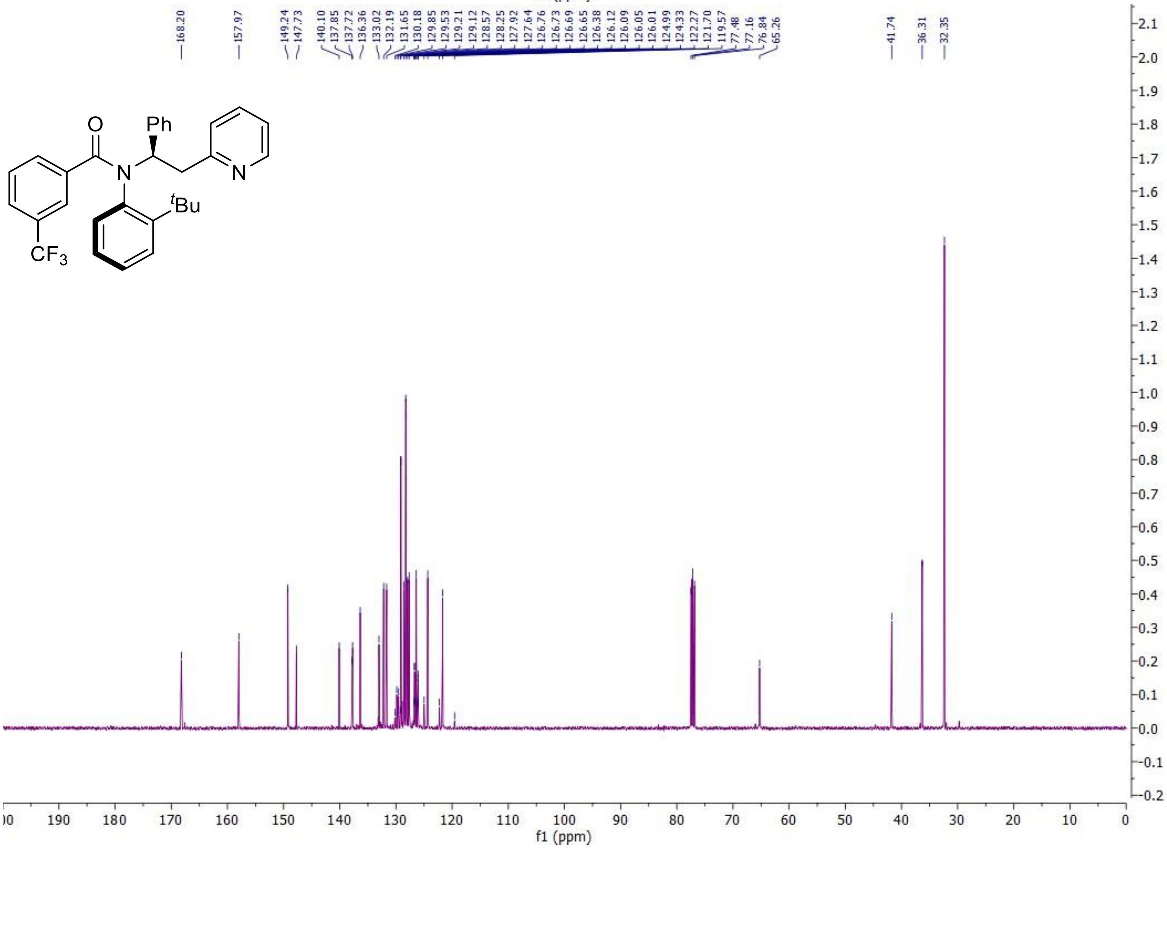

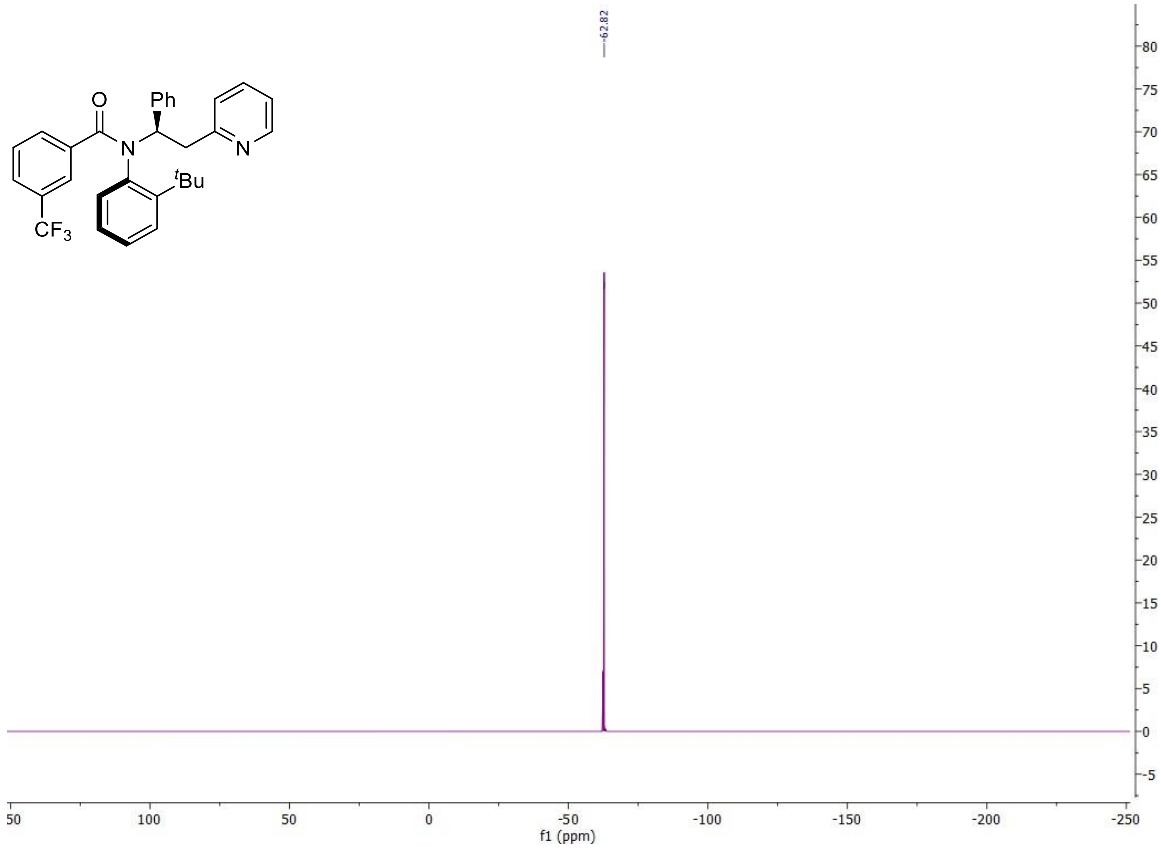
-62.82
O
Ph
N
N
tBu
CF3
50 100 50 0 -50 -100 -150 -200 -250
f1 (ppm)
80 75 70 65 60 55 50 45 40 35 30 25 20 15 10 5 0 -5

_N_-(2-(_tert_-Butyl)phenyl)-2,4,6-trifluoro-_N_-(1-phenyl-2-(pyridin-2-yl)ethyl)benzamide (6h)

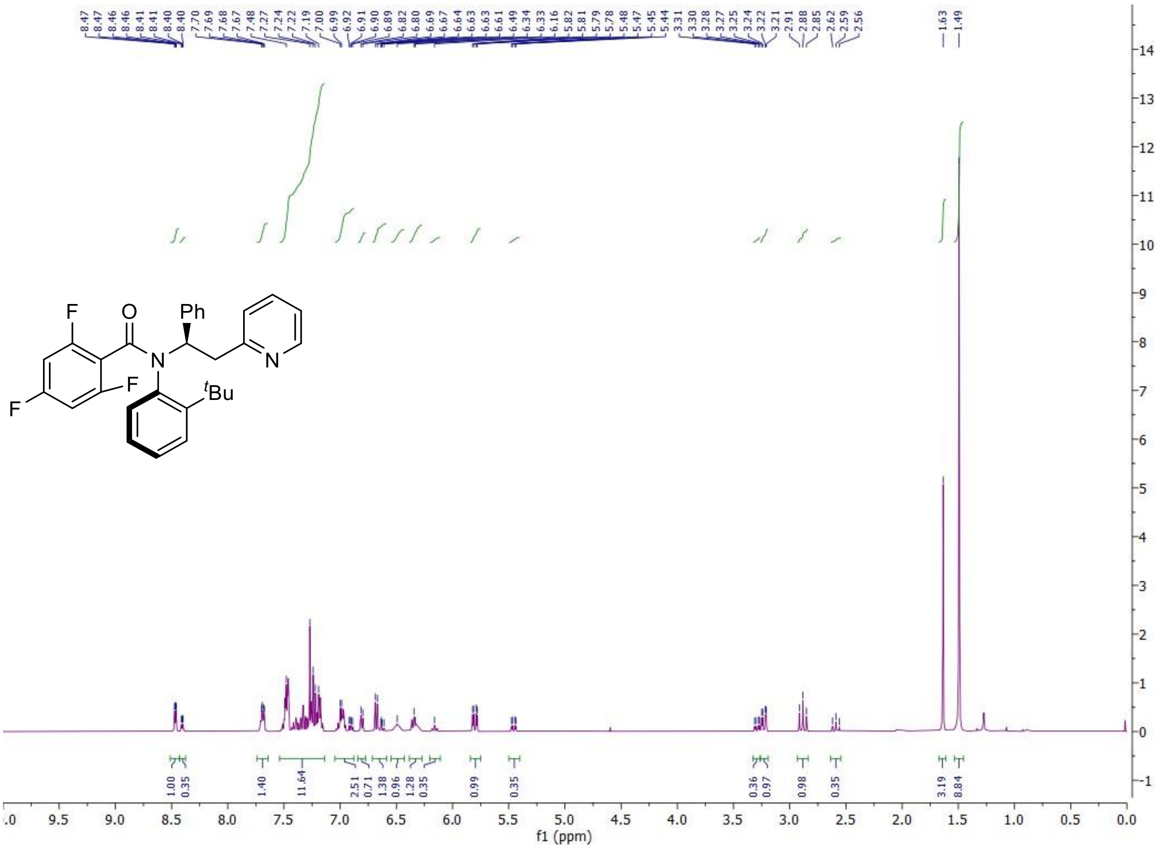

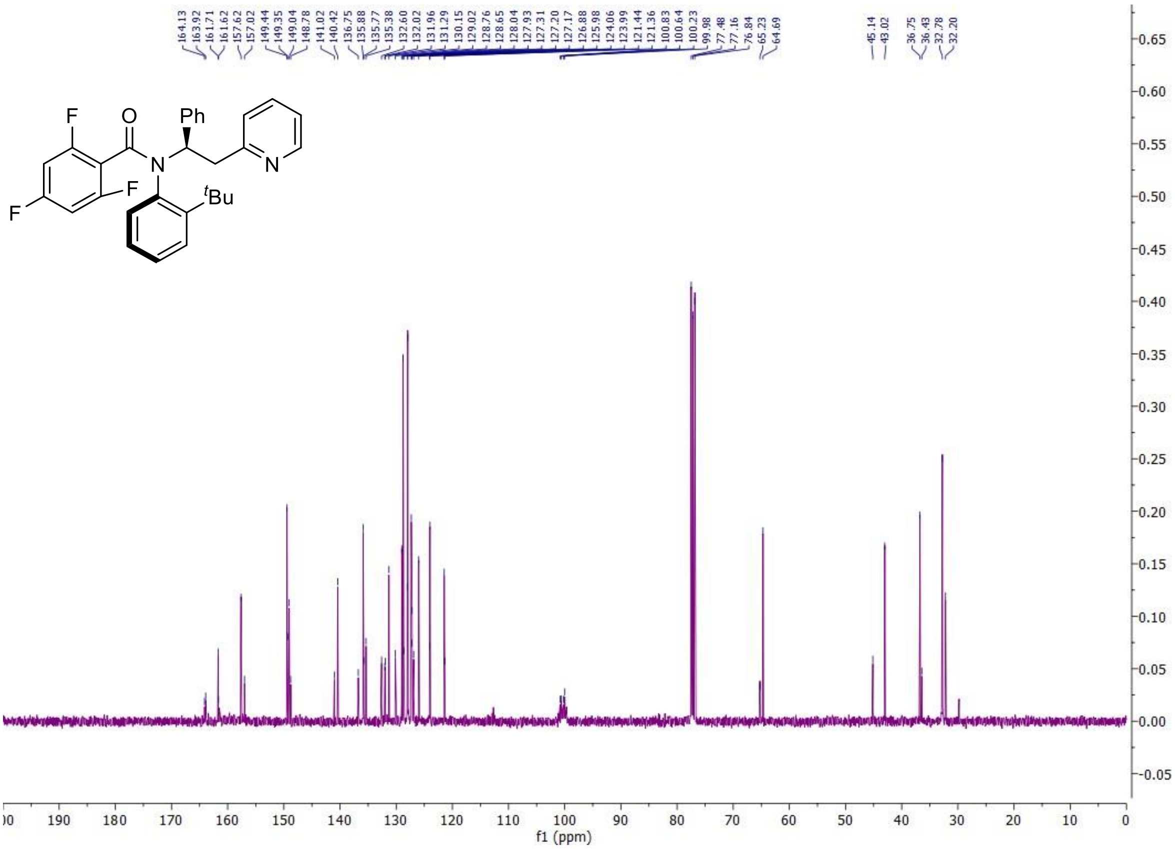

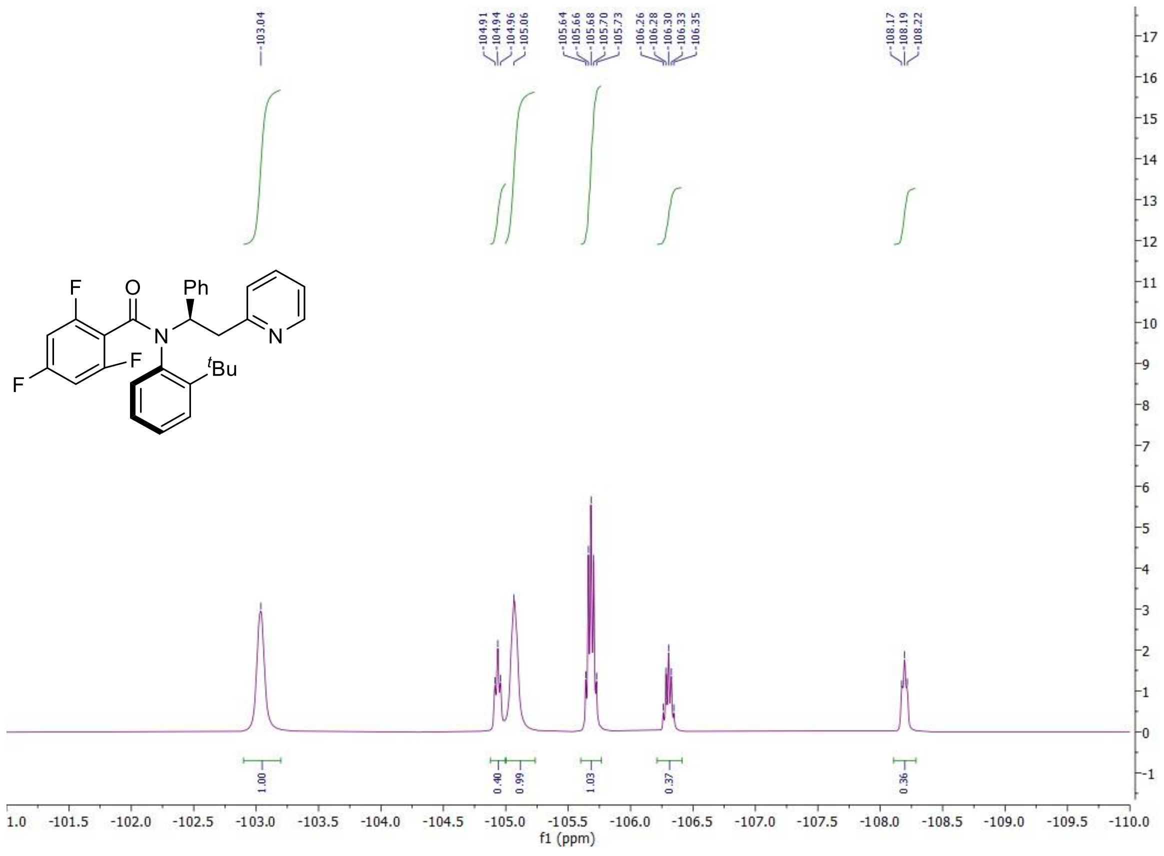

-103.04
-104.91
-104.94
-104.96
-105.06
-105.64
-105.66
-105.68
-105.70
-105.73
-106.26
-106.28
-106.30
-106.33
-106.35
-108.17
-108.19
-108.22
1.00
0.40
0.99
1.03
0.37
0.36
f1 (ppm)

N-(2-(*tert*-Butyl)phenyl)-4-nitro-*N*-(1-phenyl-2-(pyridin-2-yl)ethyl)benzamide (6i)

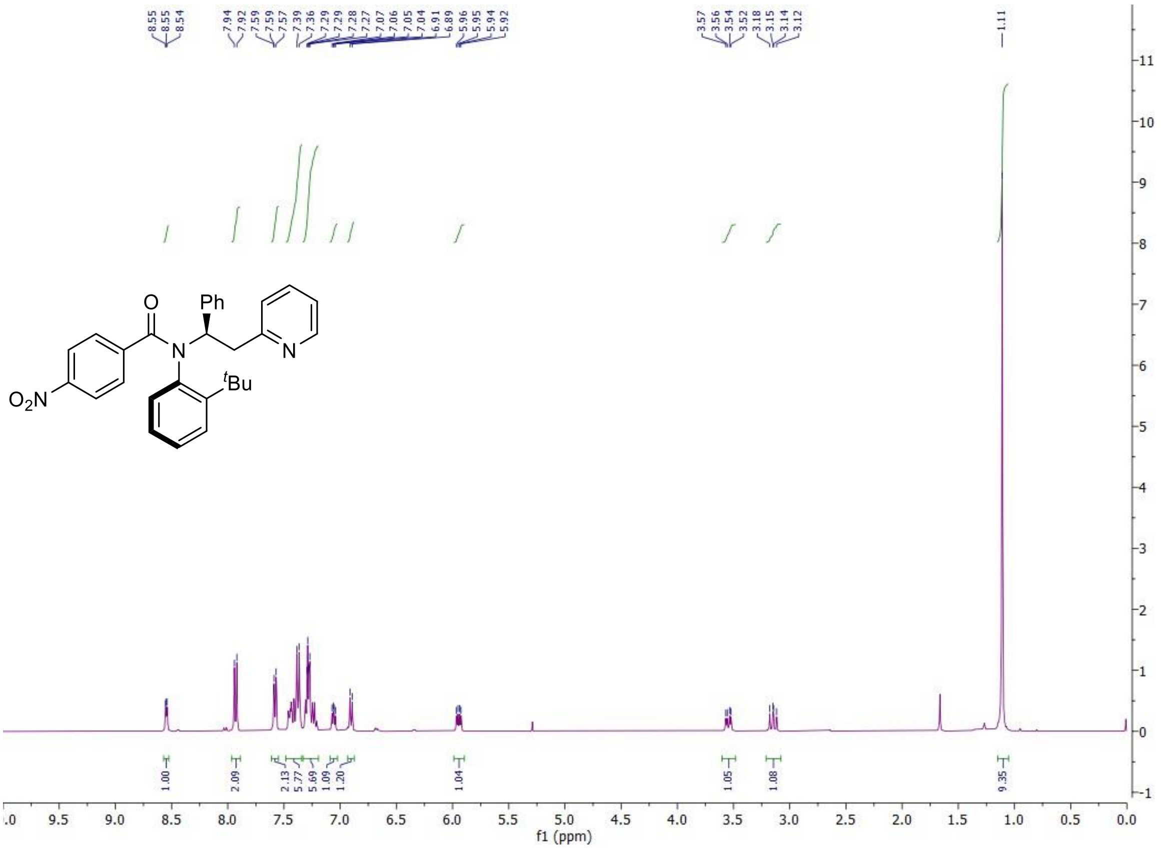

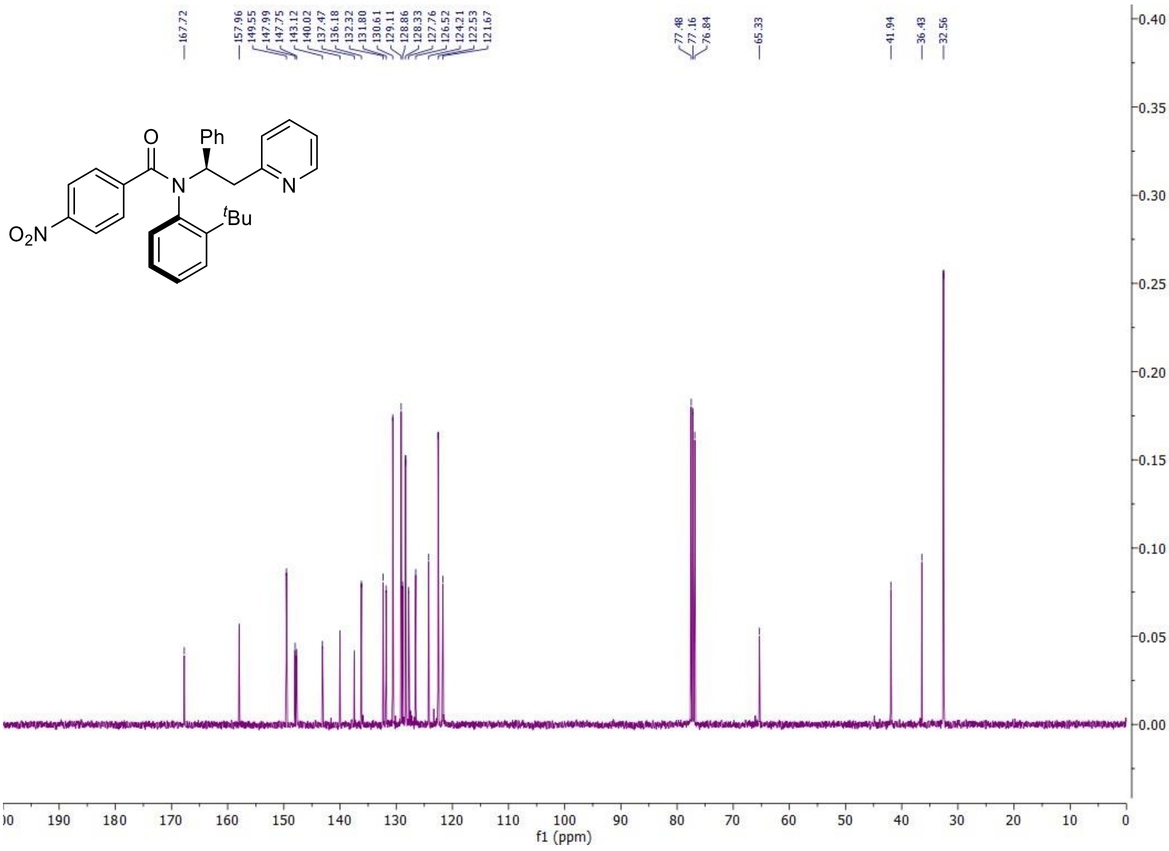

N-(2-(tert-Butyl)phenyl)-3,5-dinitro-N-(1-phenyl-2-(pyridin-2-yl)ethyl)benzamide (6j)

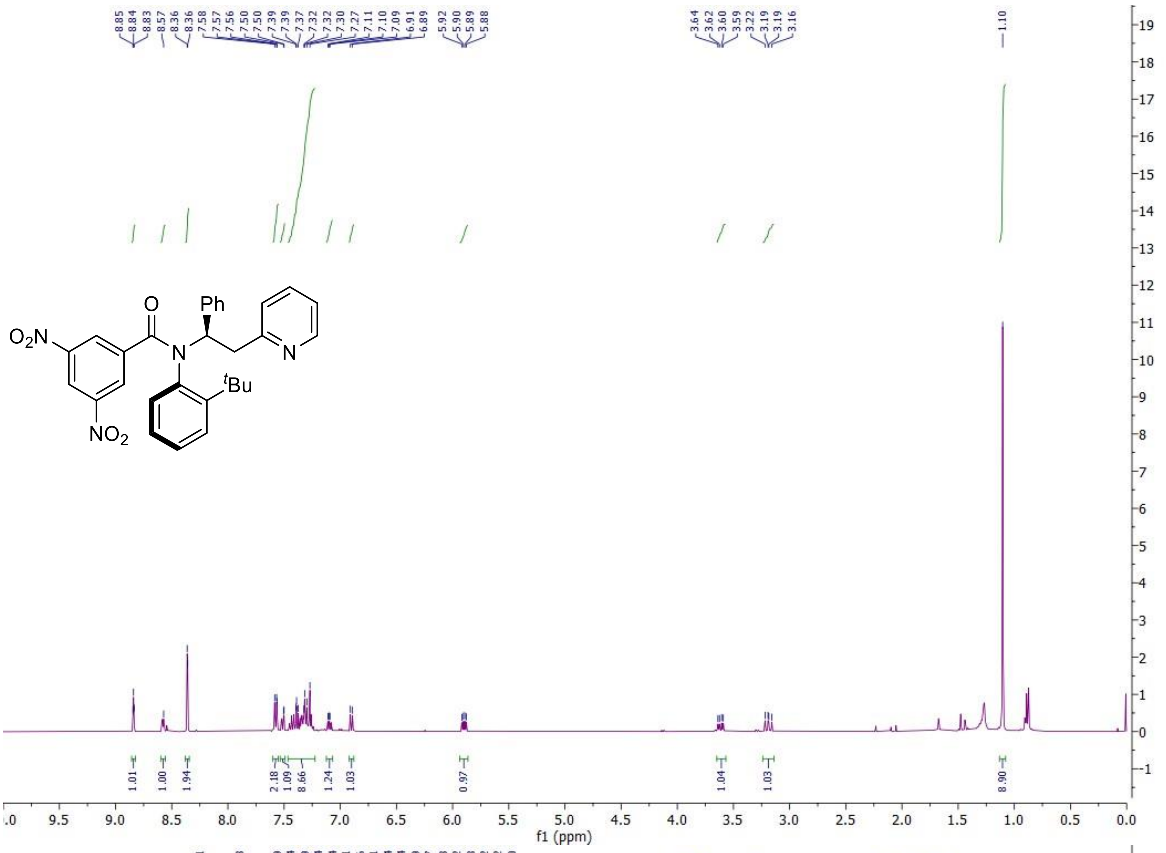

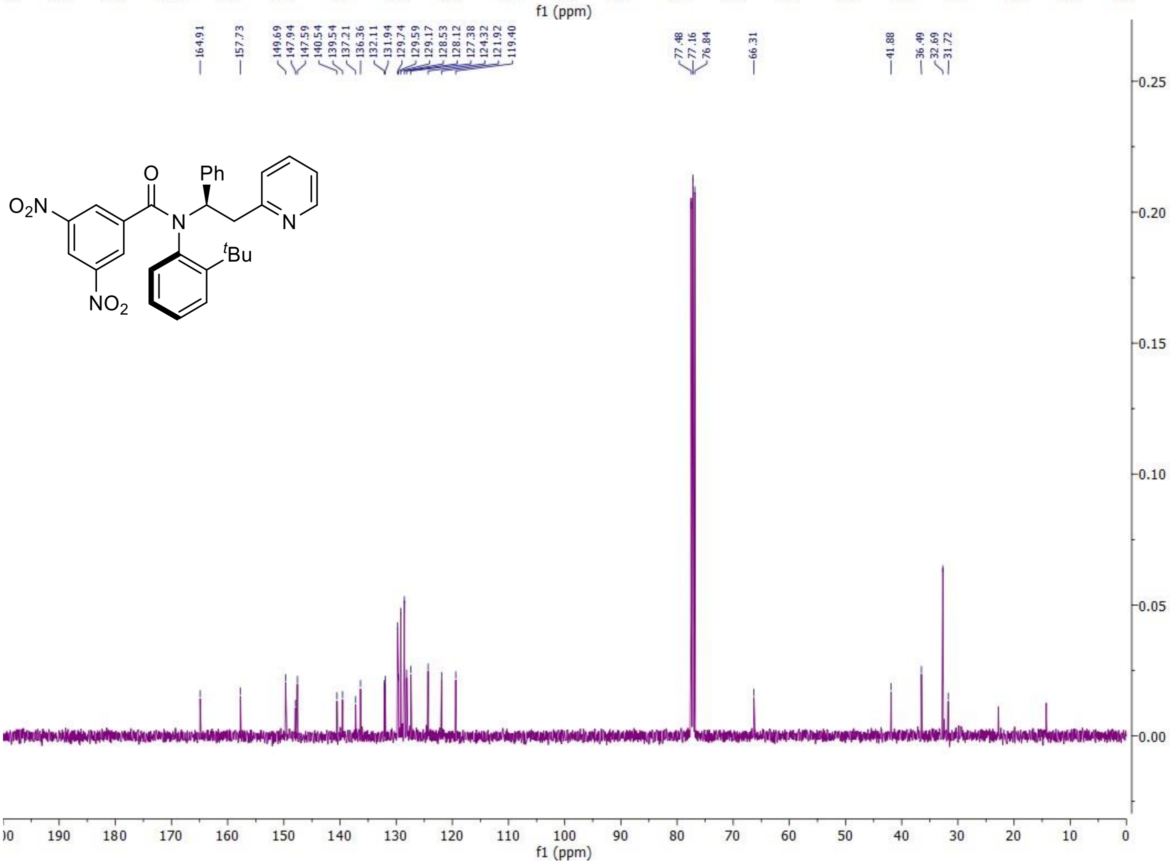

N-(2-(*tert*-Butyl)phenyl)-2-chloro-N-(1-phenyl-2-(pyridin-2-yl)ethyl)benzamide (6k)

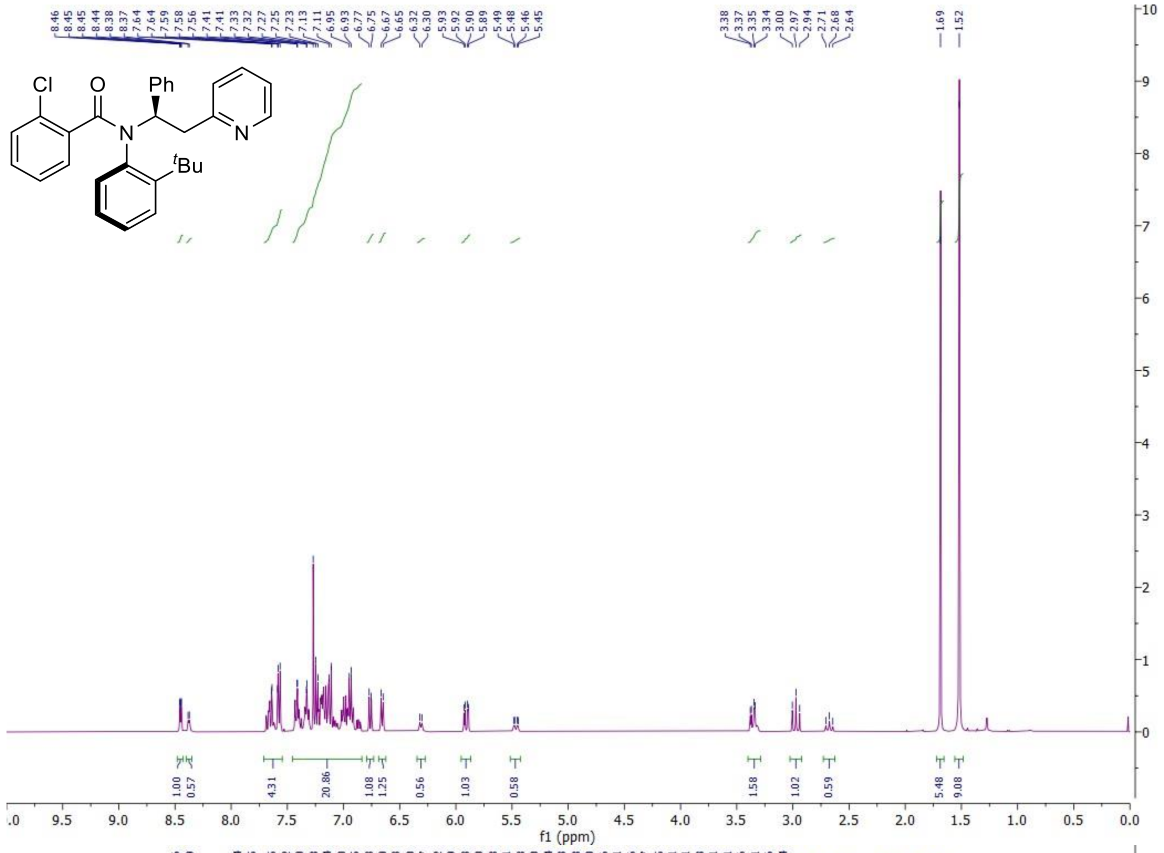

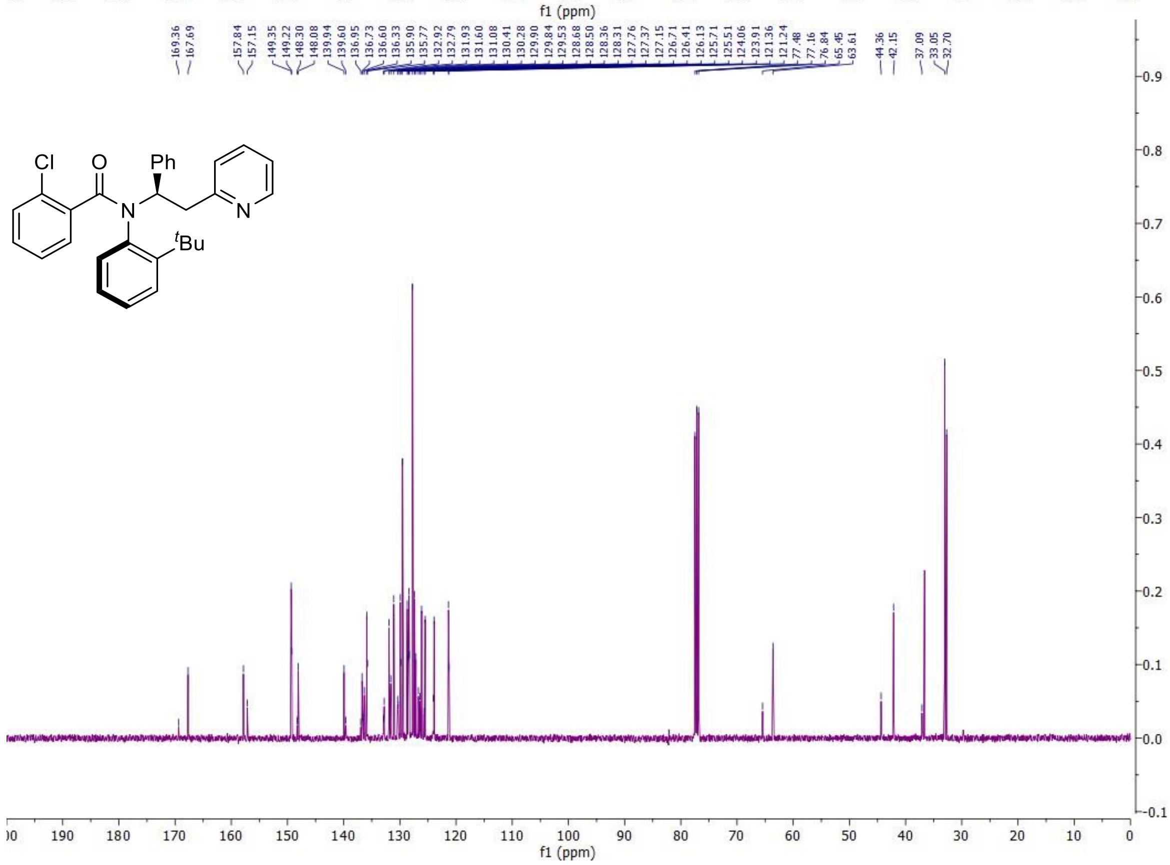

N-(2-(tert-Butyl)phenyl)-N-(1-phenyl-2-(pyridin-2-yl)ethyl)-2-naphthamide (6l)

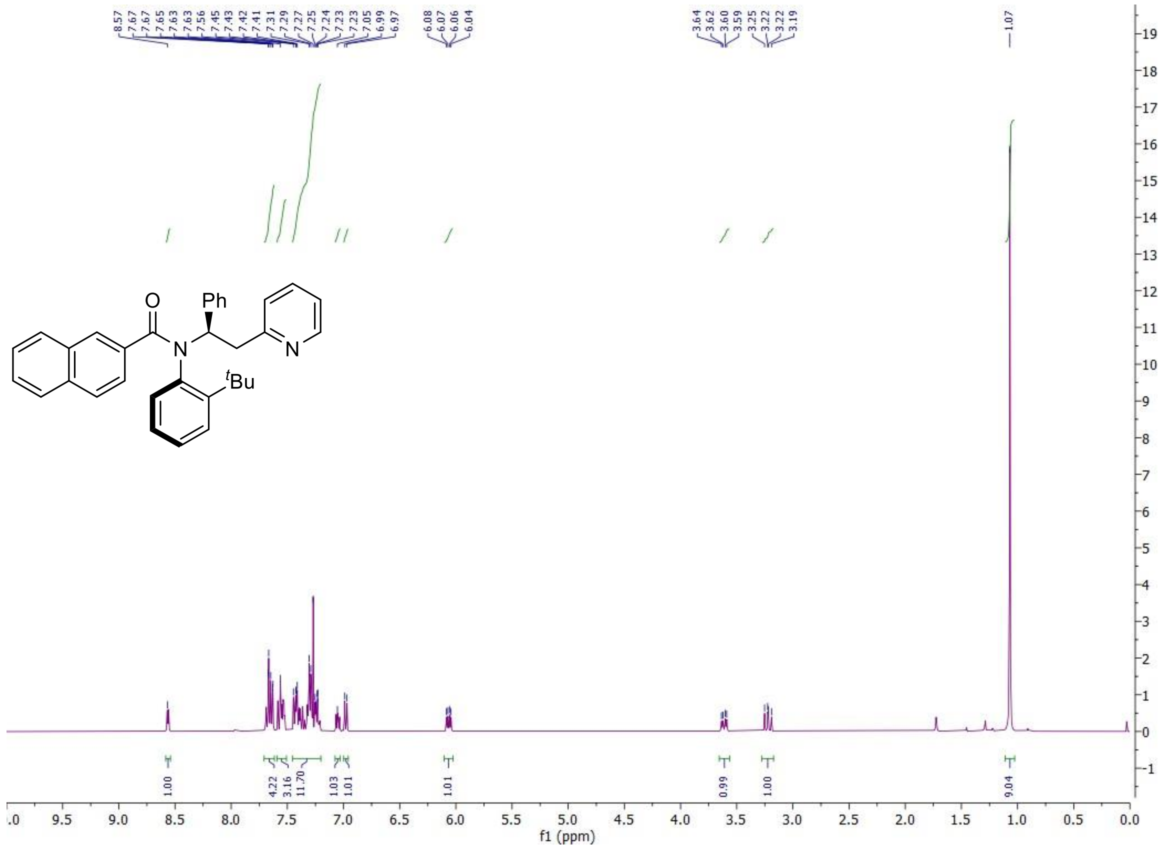

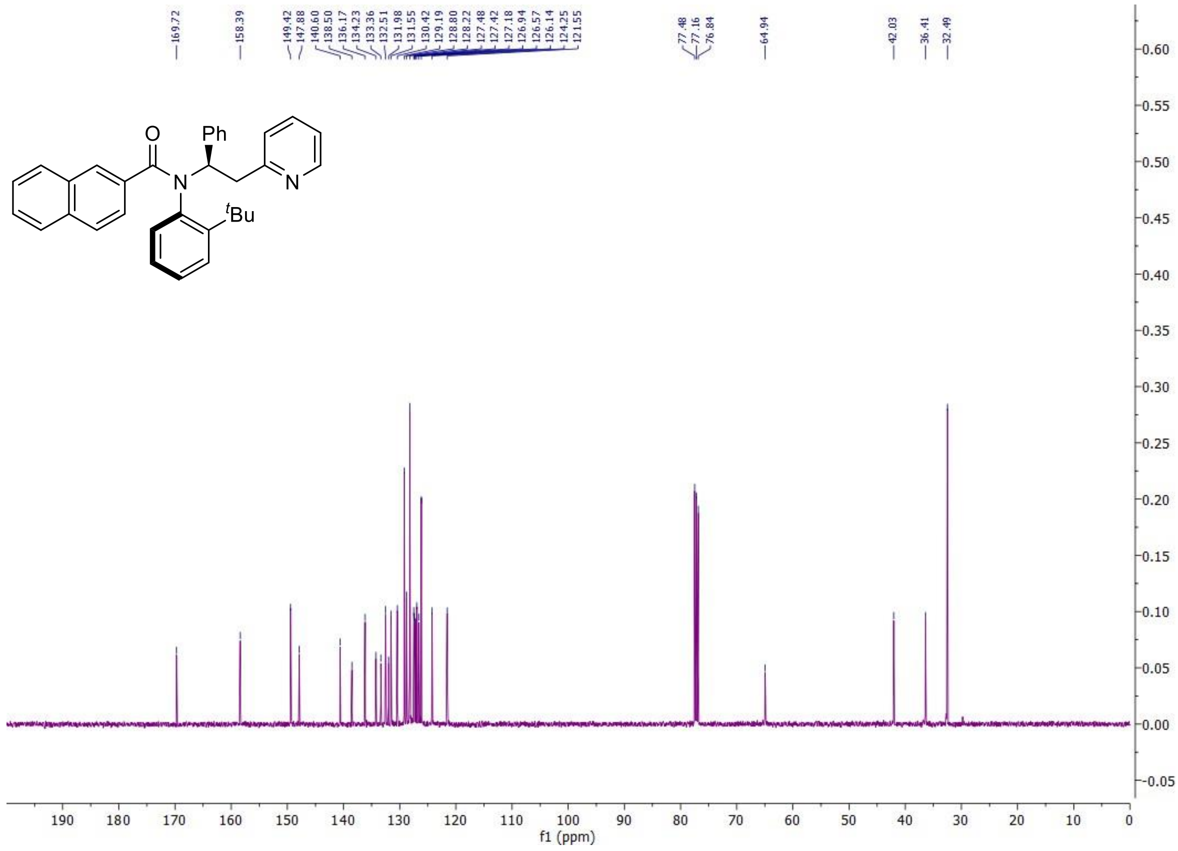

N-(2-(tert-Butyl)phenyl)-4-methoxy-N-(1-phenyl-2-(pyridin-2-yl)ethyl)benzamide (6m)

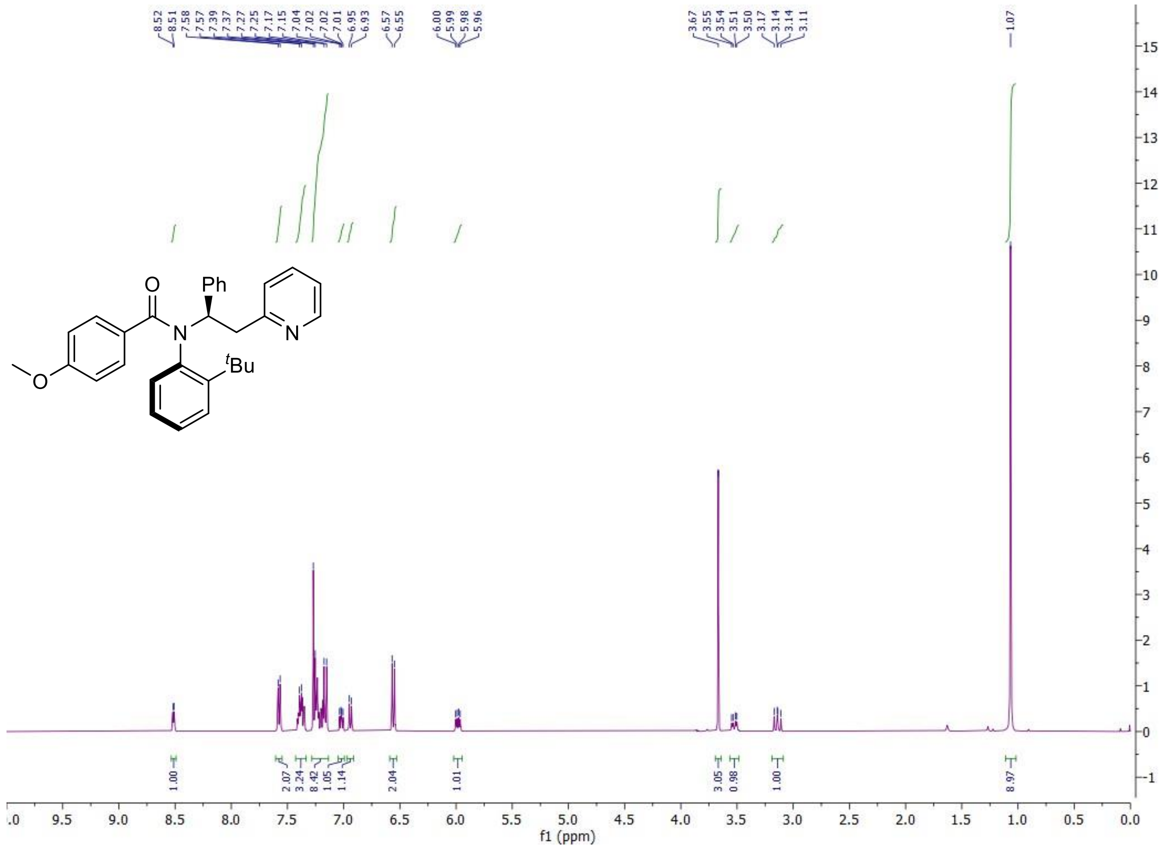

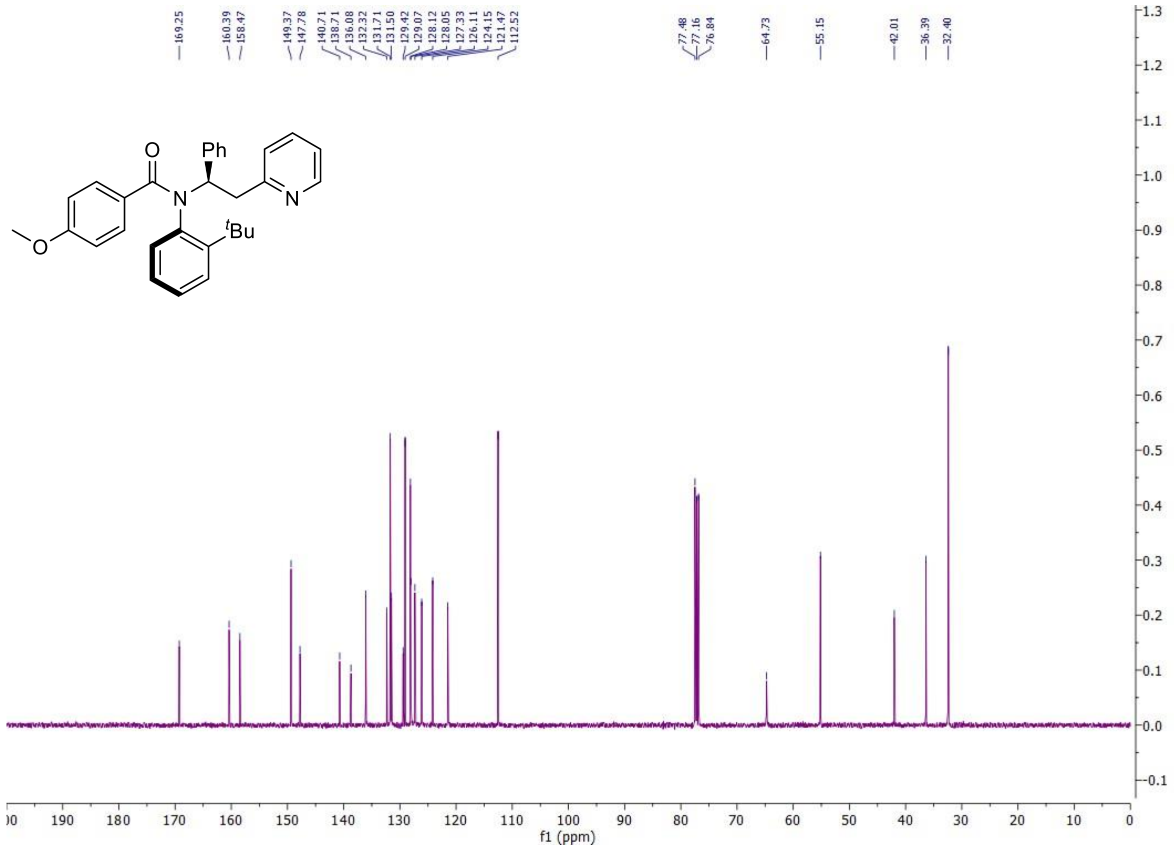

N-(2-(*tert*-Butyl)phenyl)-4-methoxy-*N*-(1-phenyl-2-(pyridin-2-yl)ethyl)benzamide (11m)

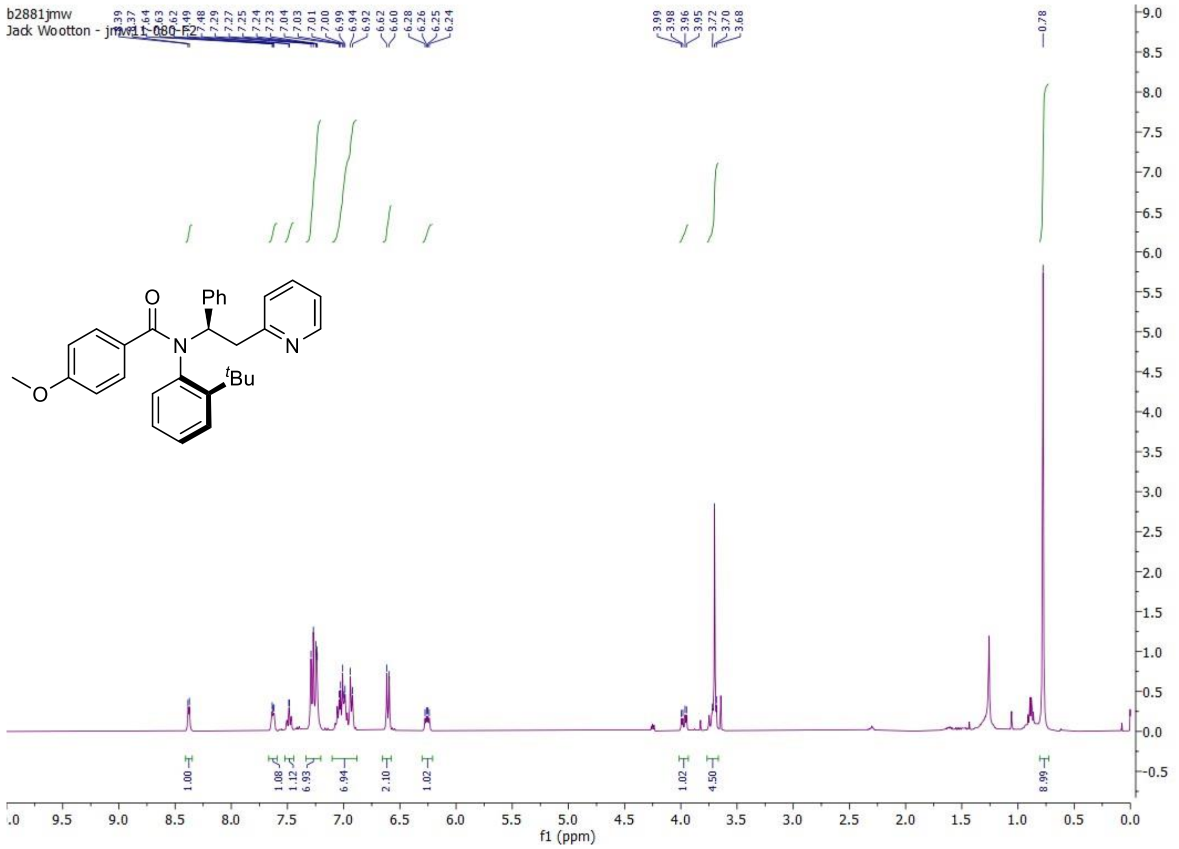

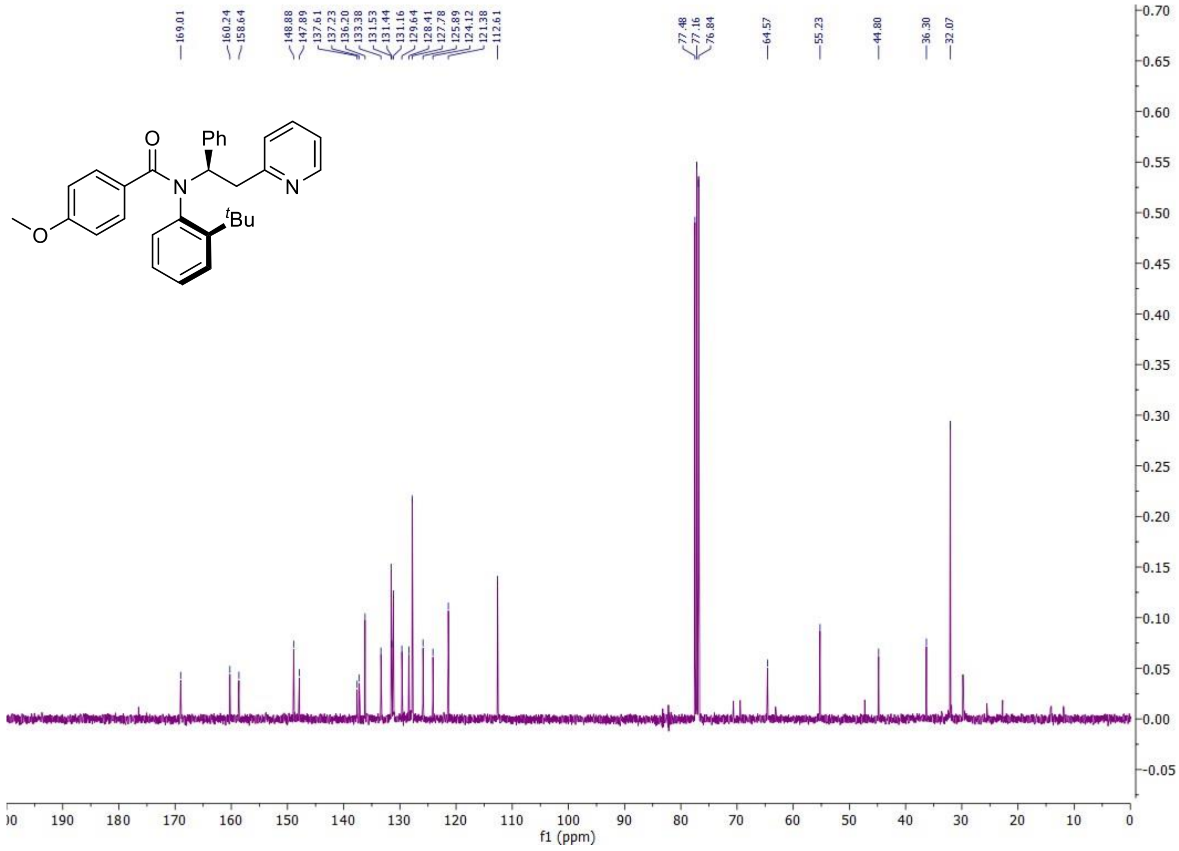

N-(2-(tert-Butyl)phenyl)-2-(1H-indol-3-yl)-2-oxo-N-(1-phenyl-2-(pyridin-2-yl)ethyl)acetamide (6n)

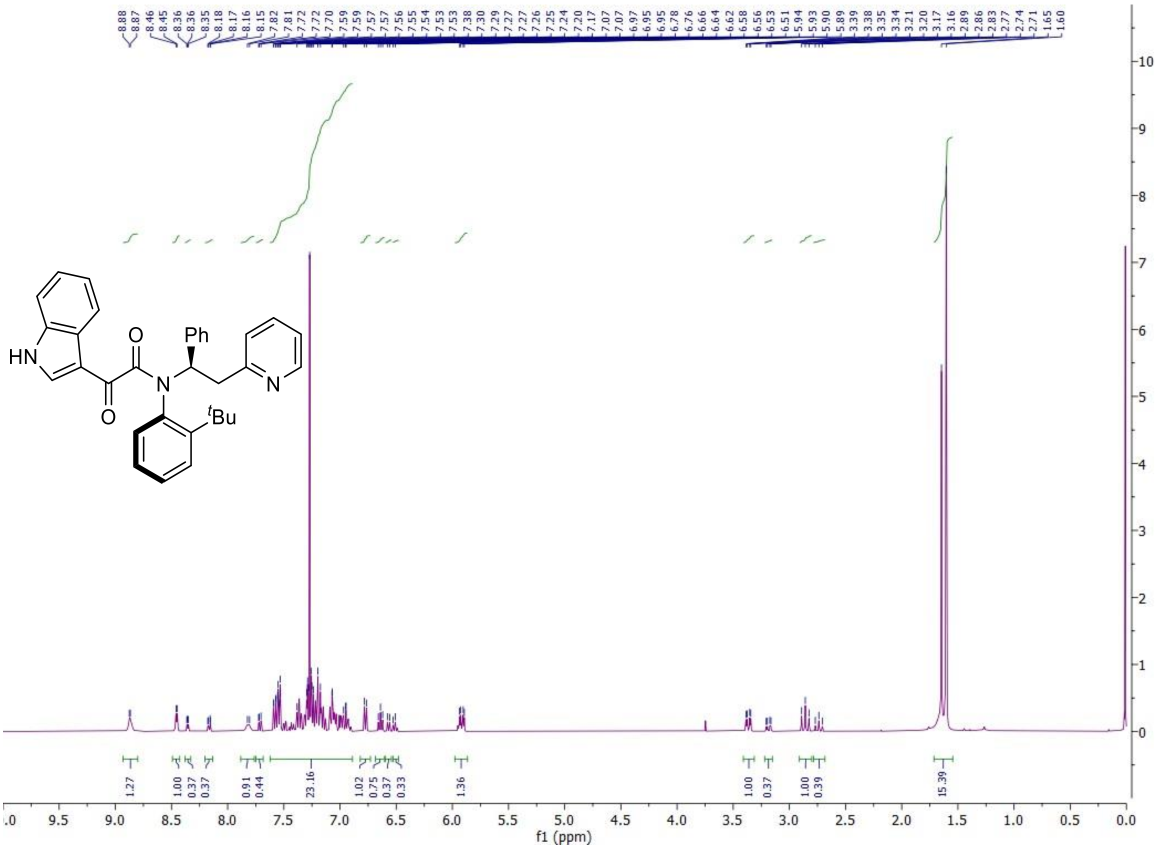

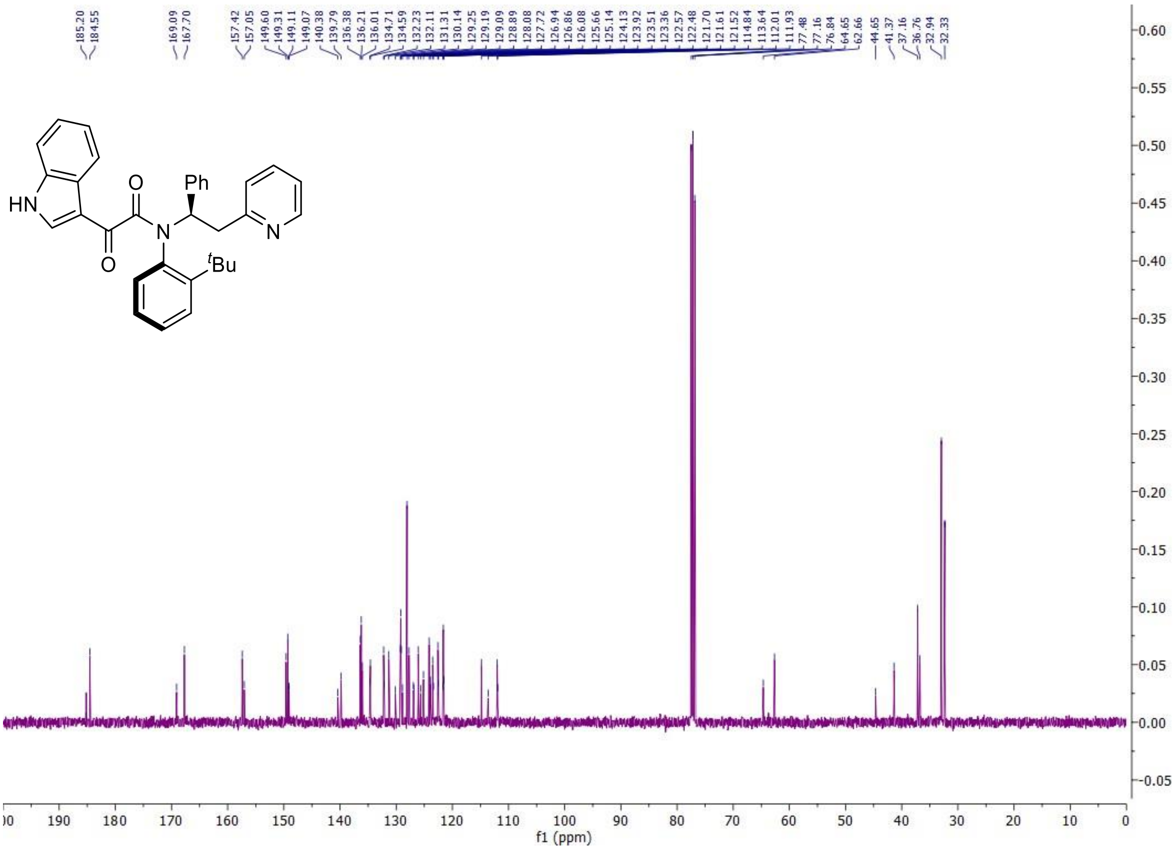

(4R)-*N*-(2-(*tert*-Butyl)phenyl)-4-((5S,8R,9S,10S,13R,14S,17R)-10,13-dimethyl-3,7,12-trioxohexadecahydro-1*H*-cyclopenta[a]phenanthren-17-yl)-*N*-(1-phenyl-2-(pyridin-2-yl)ethyl)pentanamide (6o)

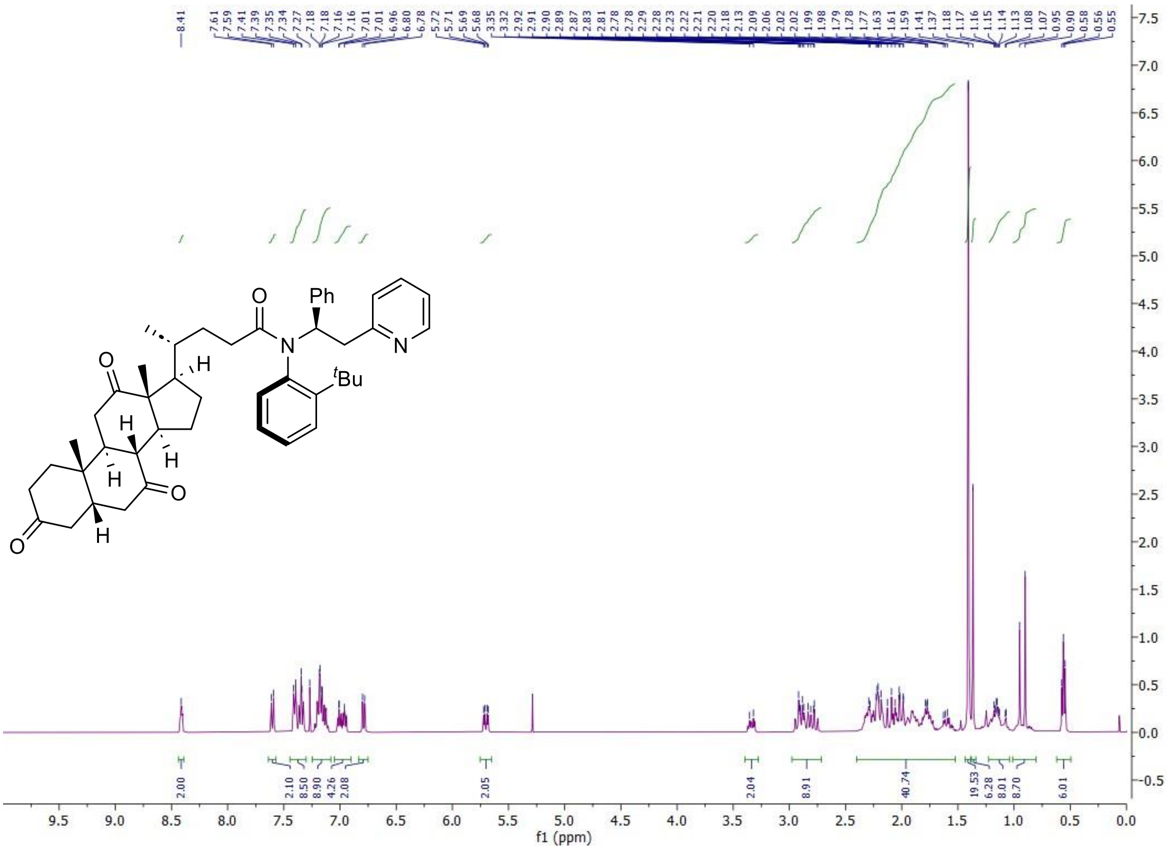

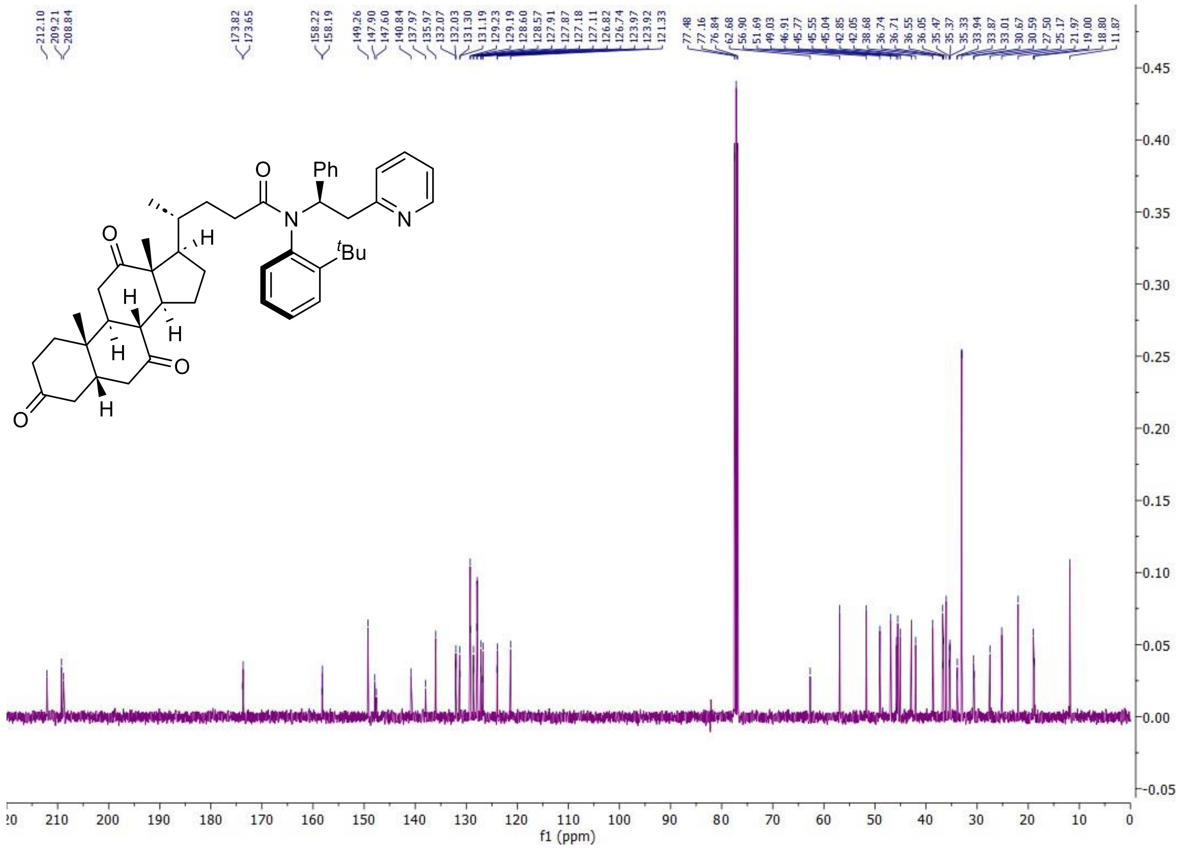

212.10
209.21
208.84
173.82
173.65
158.22
158.19
149.26
147.90
147.60
140.84
137.97
135.97
132.07
132.03
131.30
131.19
129.23
129.19
128.60
128.57
127.91
127.87
127.18
127.11
126.82
126.74
123.97
123.92
121.33
77.48
77.16
76.84
62.68
56.90
51.69
49.03
46.91
45.77
45.55
45.04
42.85
42.05
38.68
36.74
36.71
36.55
36.05
35.47
35.37
35.33
33.94
33.87
33.01
30.67
30.59
27.50
25.17
21.97
19.00
18.80
11.87
O
Ph
N
tBu
H
f1 (ppm)

***N*-(2-(*tert*-Butyl)phenyl)-*N*-(1-phenyl-2-(pyridin-2-yl)ethyl)thiophene-3-carboxamide (6p)**

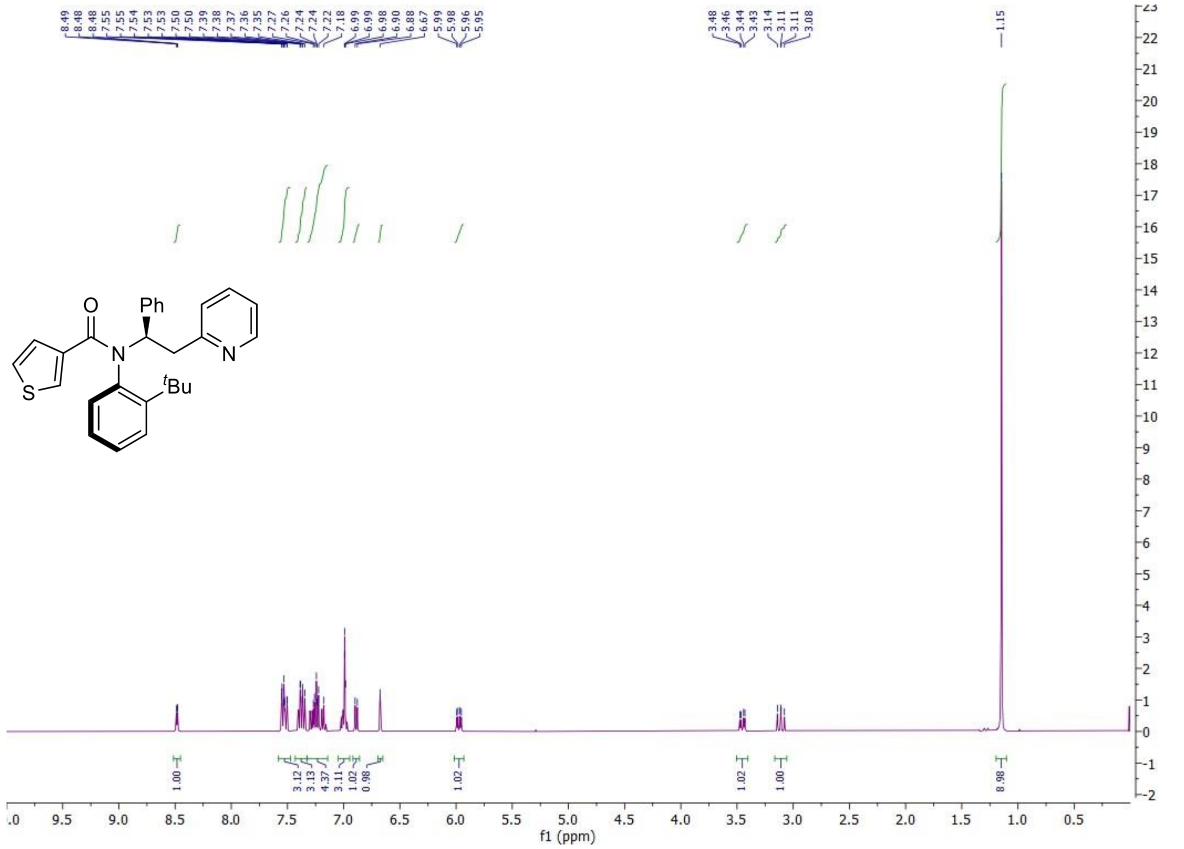

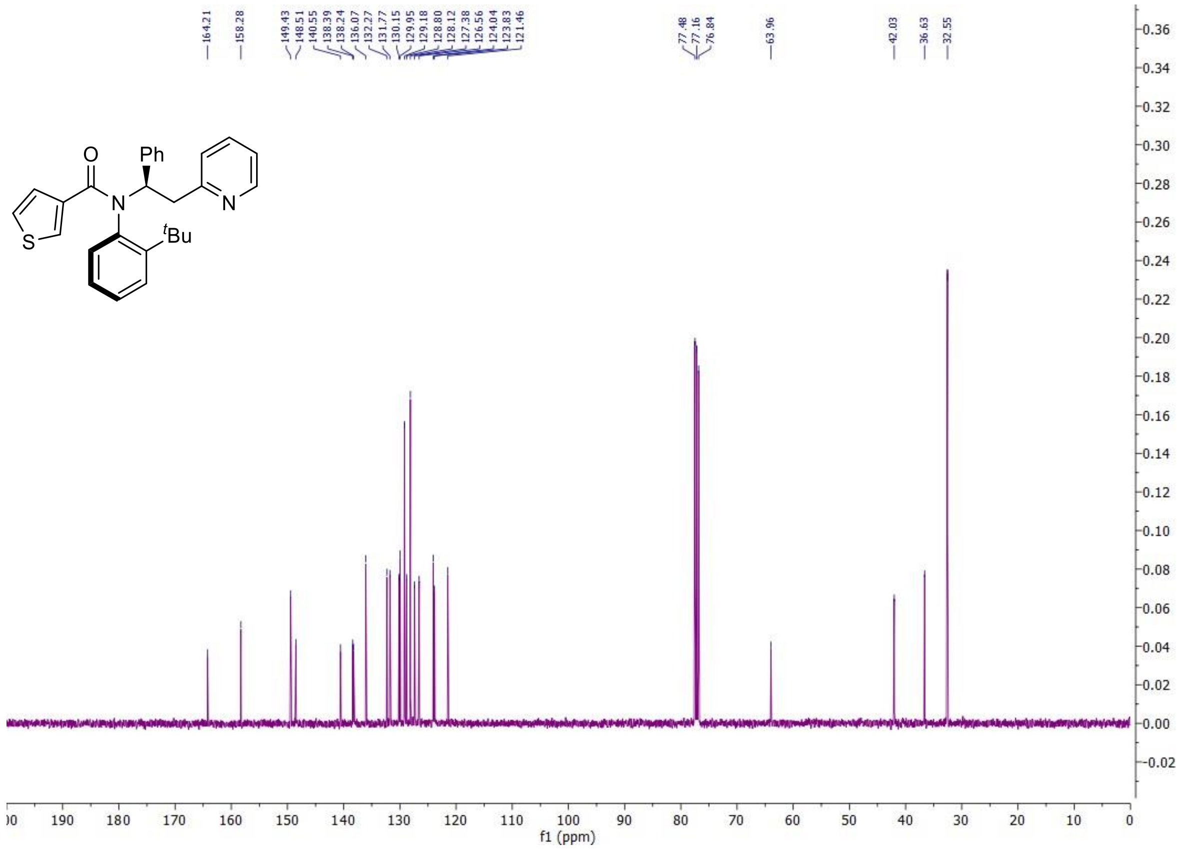

2-(Benzyloxy)-*N*-(2-(*tert*-butyl)phenyl)-*N*-(1-phenyl-2-(pyridin-2-yl)ethyl)acetamide (6q)

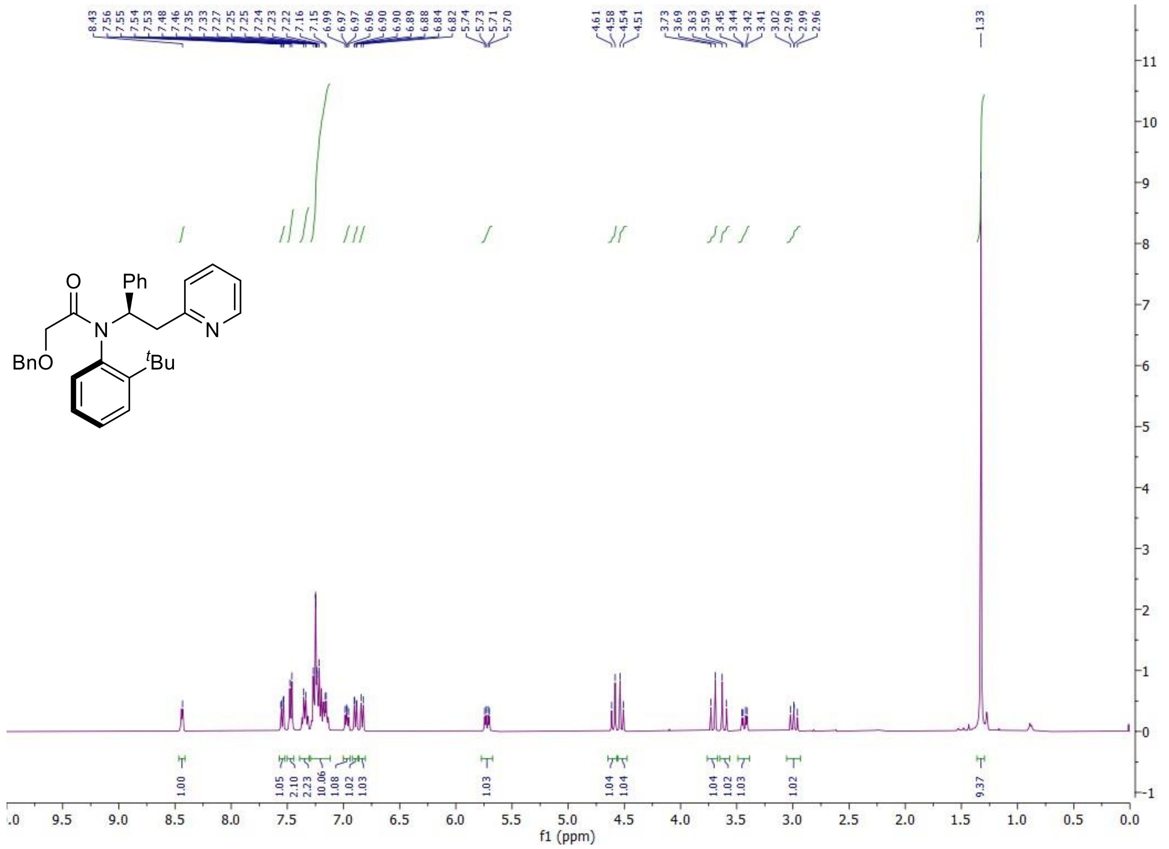

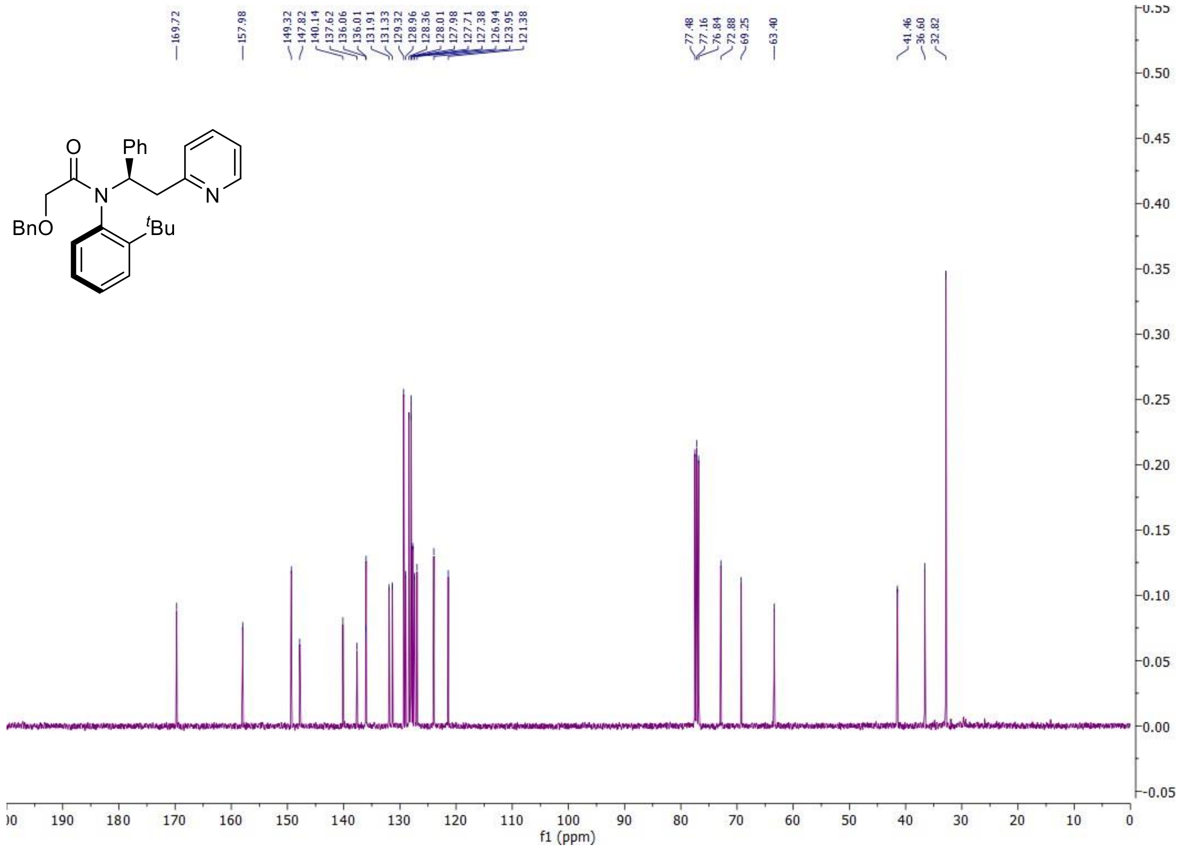

_N_-(2-(_tert_-Butyl)phenyl)-_N_-(1-(pyridin-2-yl)propan-2-yl)propionamide (6r)

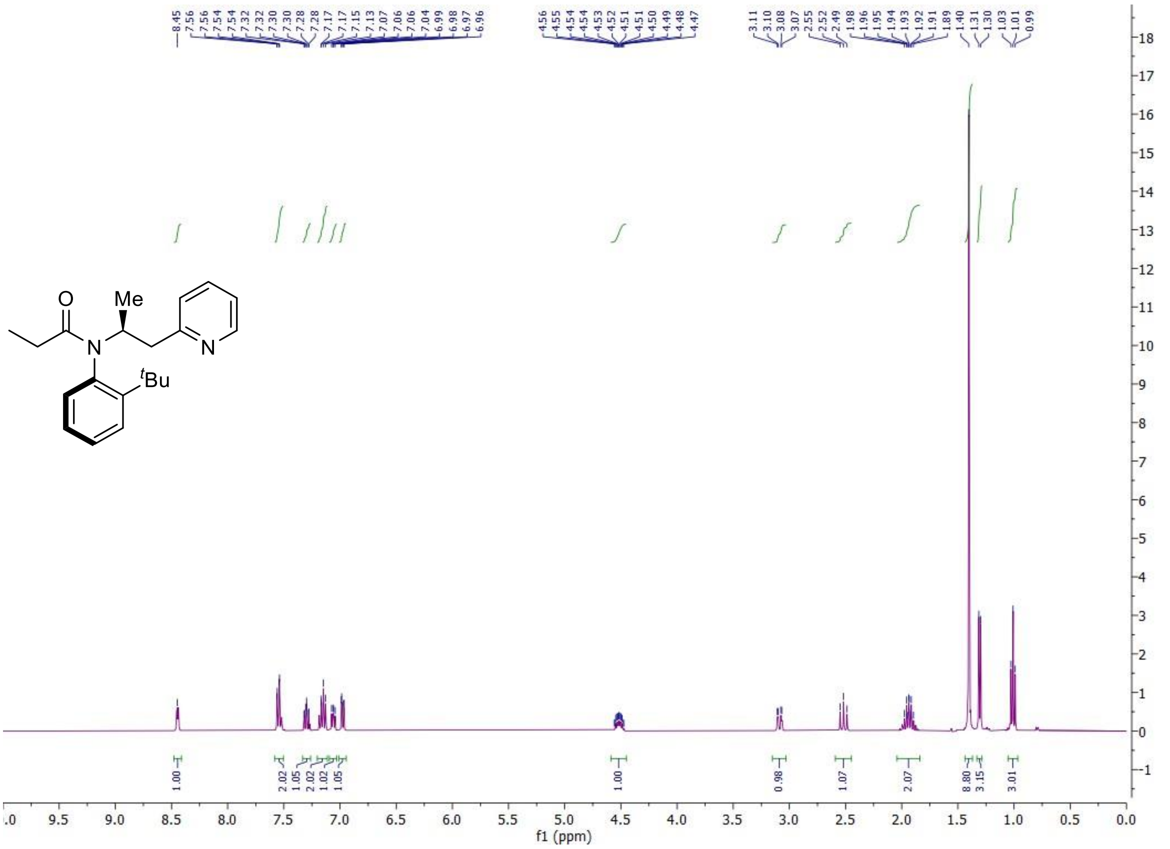

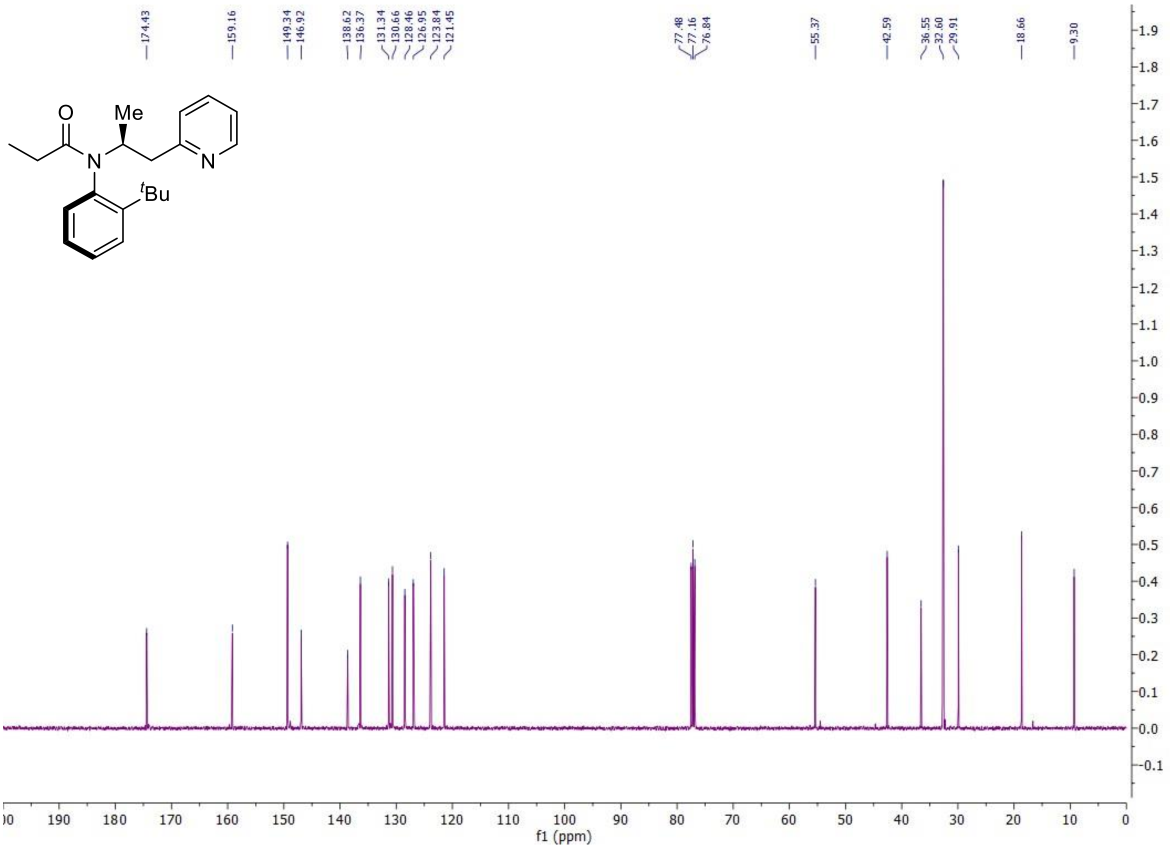

_N_-(2-(_tert_-Butyl)phenyl)-_N_-(1-phenyl-2-(pyridin-2-yl)ethyl)cyclohexanecarboxamide (6s)

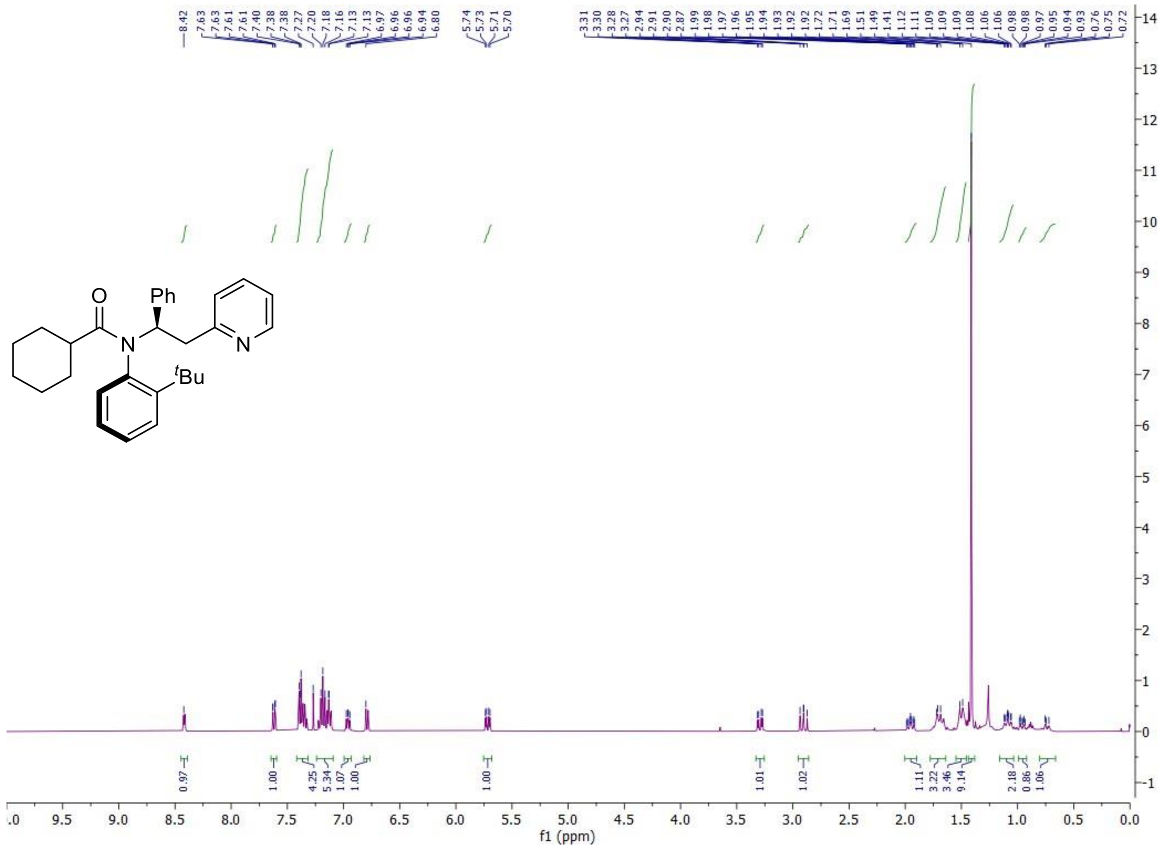

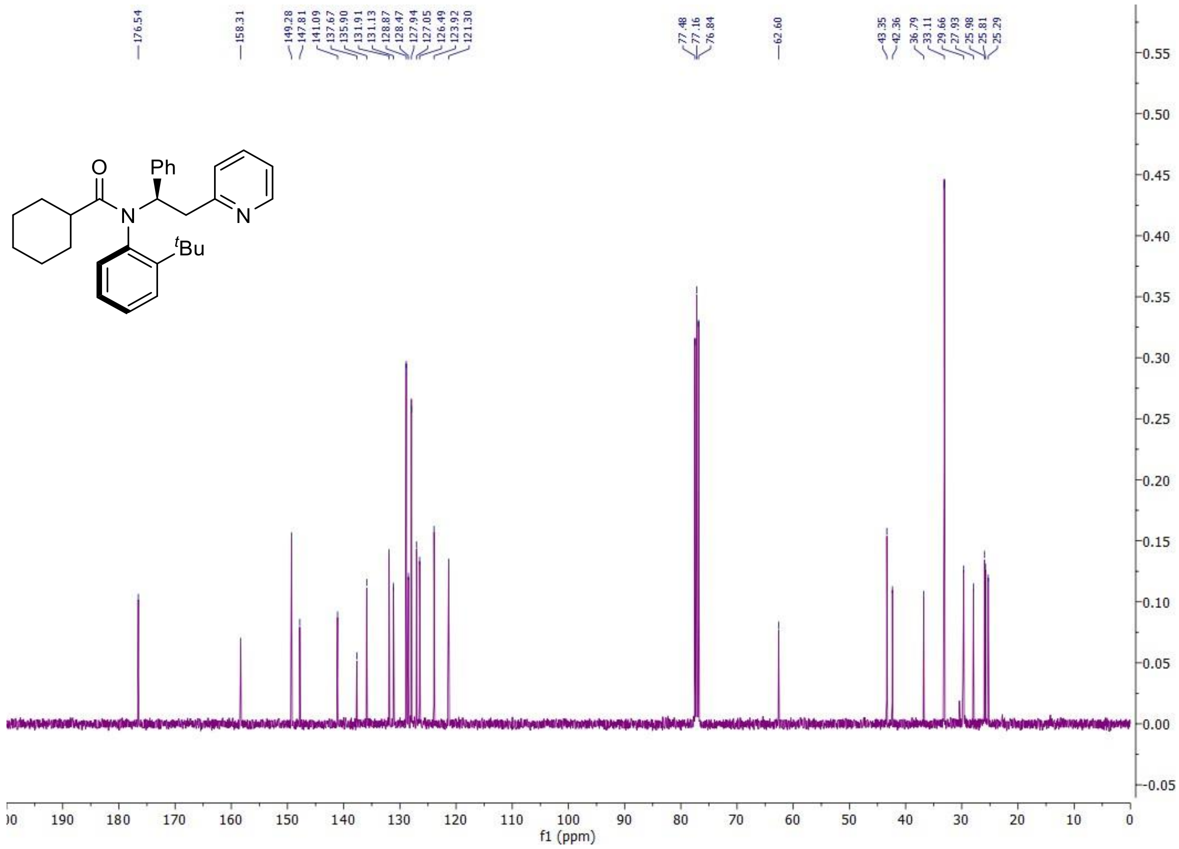

N-(2-(*tert*-Butyl)phenyl)-2-phenyl-*N*-(1-phenyl-2-(pyridin-2-yl)ethyl)acetamide (6u)

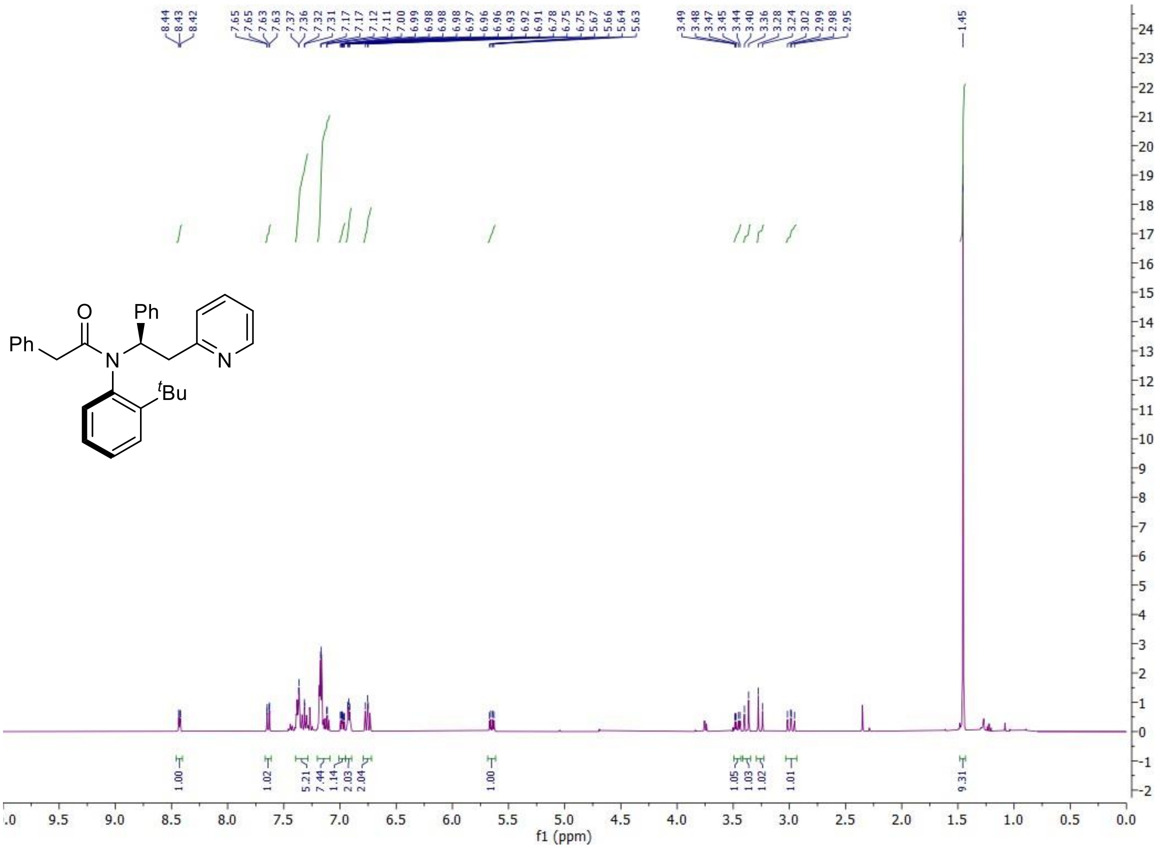

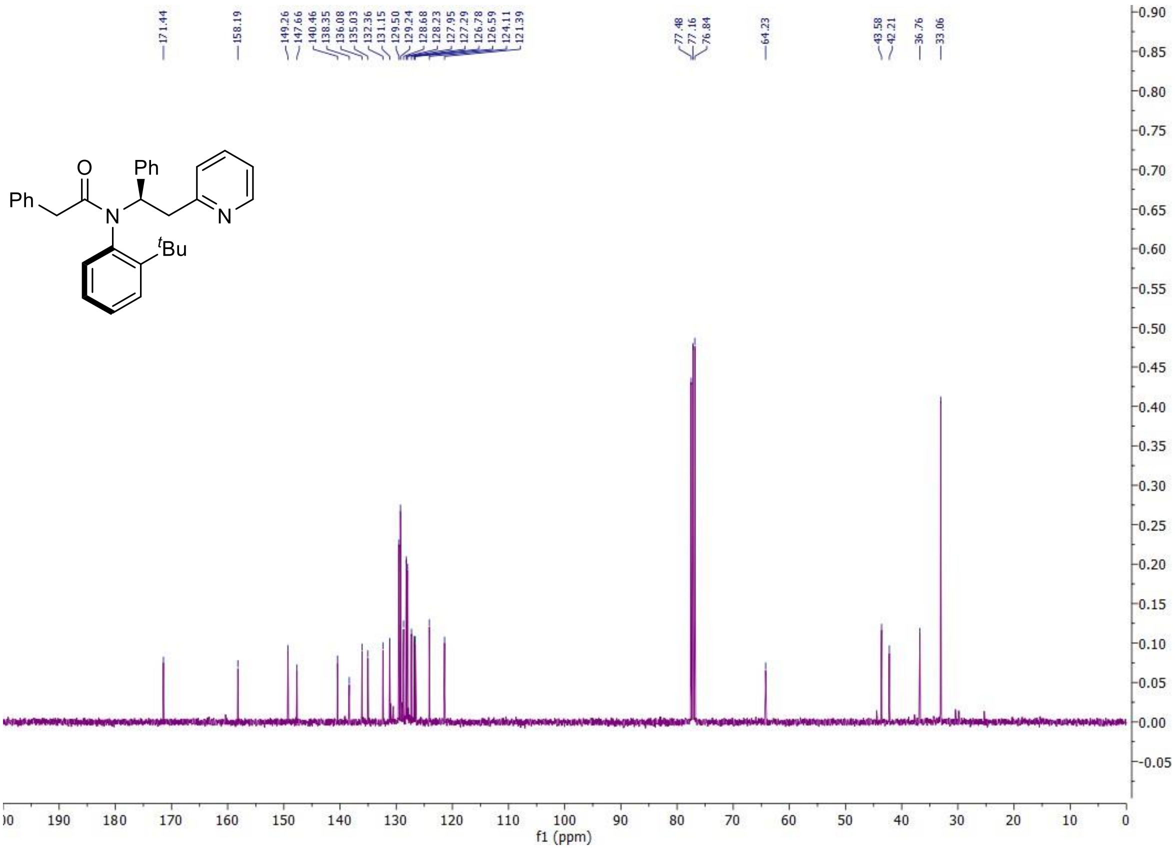

2-(*tert*-Butyl)-*N*-(phenyl(pyridin-2-yl)methyl)aniline (4c)

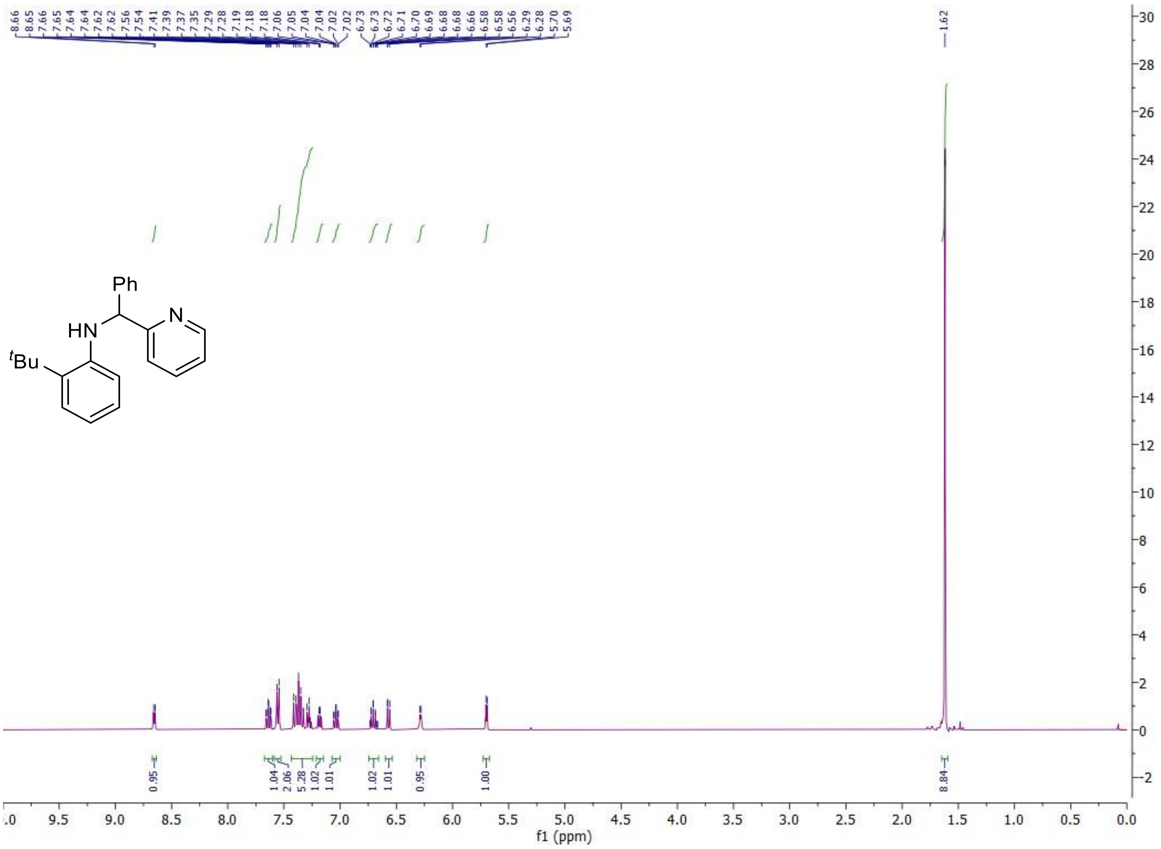

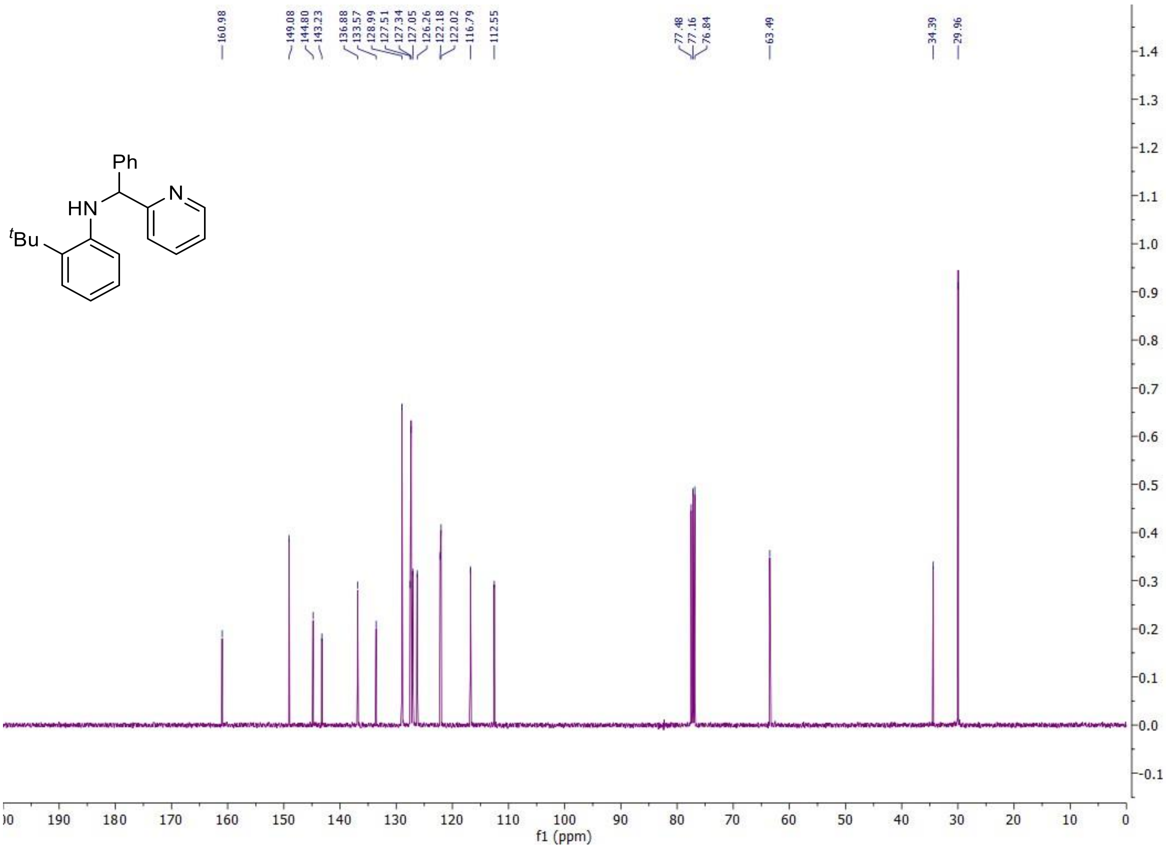

2-(*tert*-Butyl)-*N*-(1-(pyridin-2-yl)ethyl)aniline (4d)

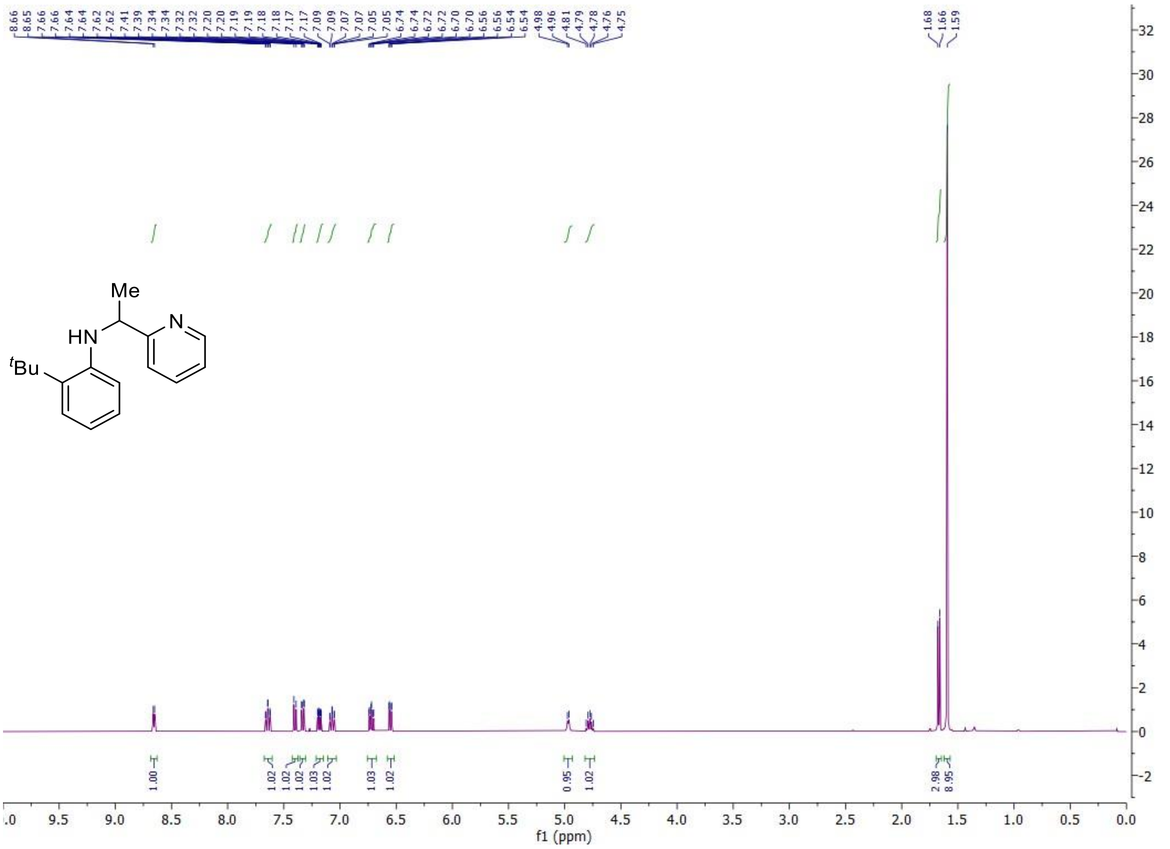

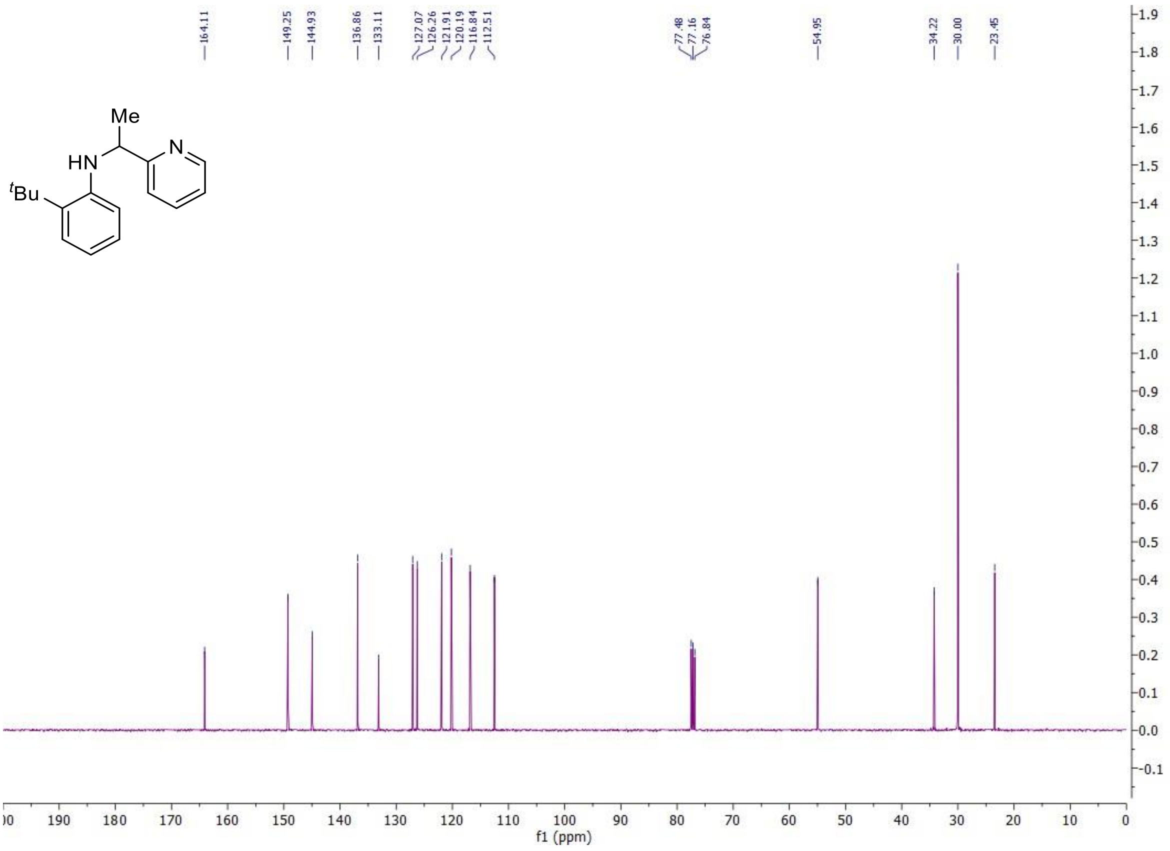

***N*-(2-(*tert*-Butyl)phenyl)-*N*-(phenyl(pyridin-2-yl)methyl)propionamide (6w)**

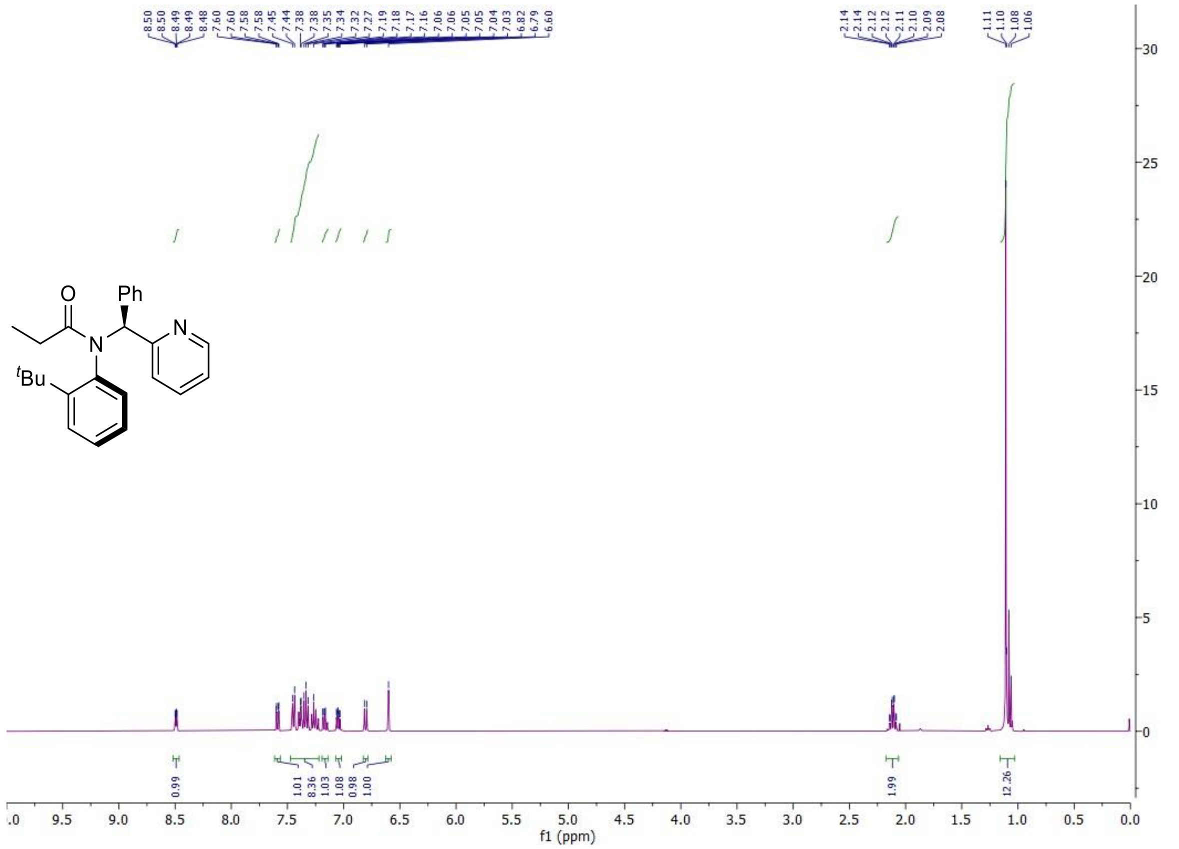

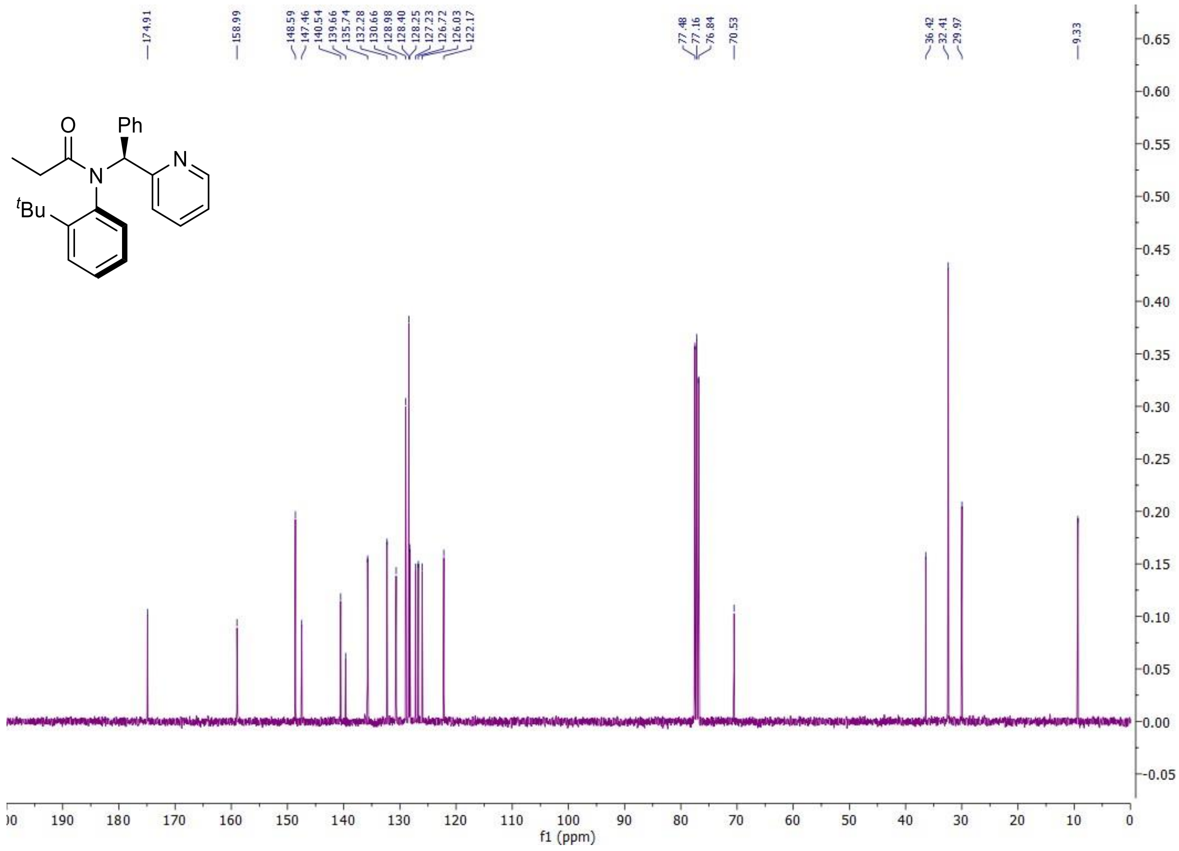

N-(2-(*tert*-Butyl)phenyl)-*N*-(phenyl(pyridin-2-yl)methyl)cyclohexanecarboxamide (S4)

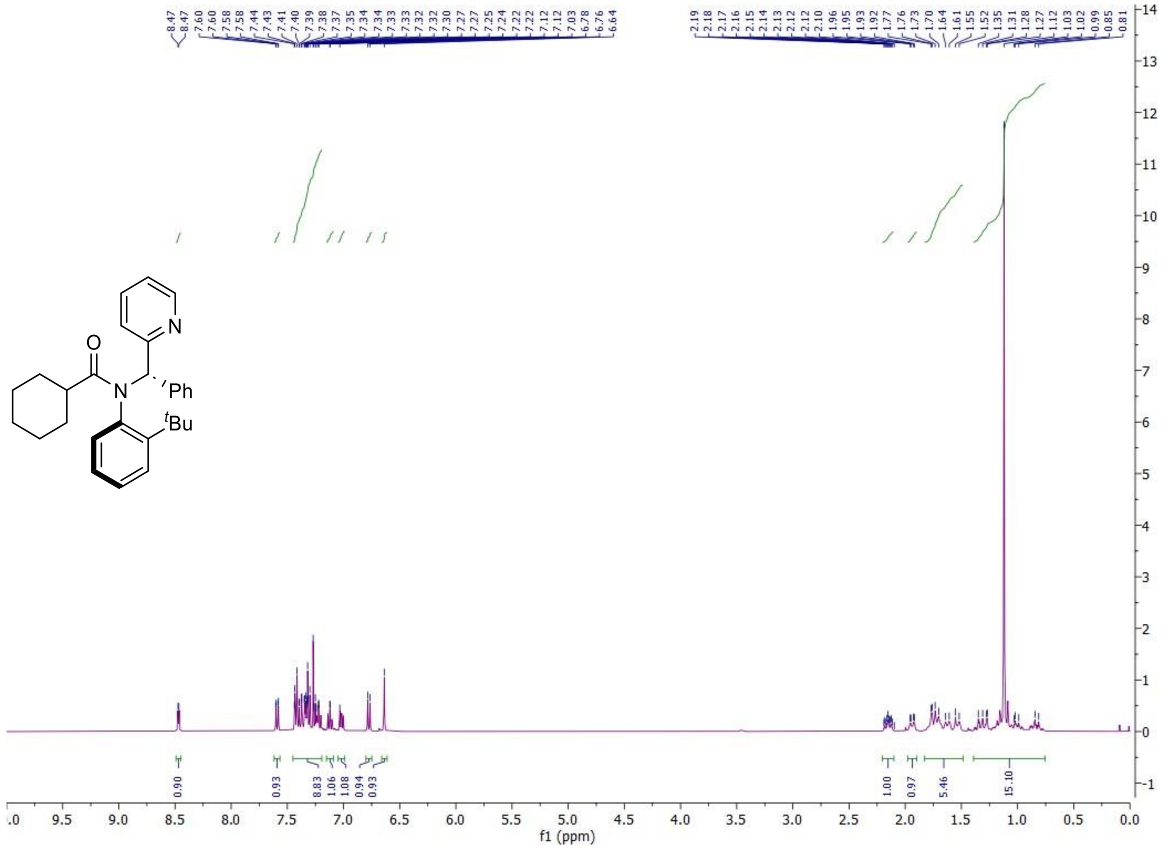

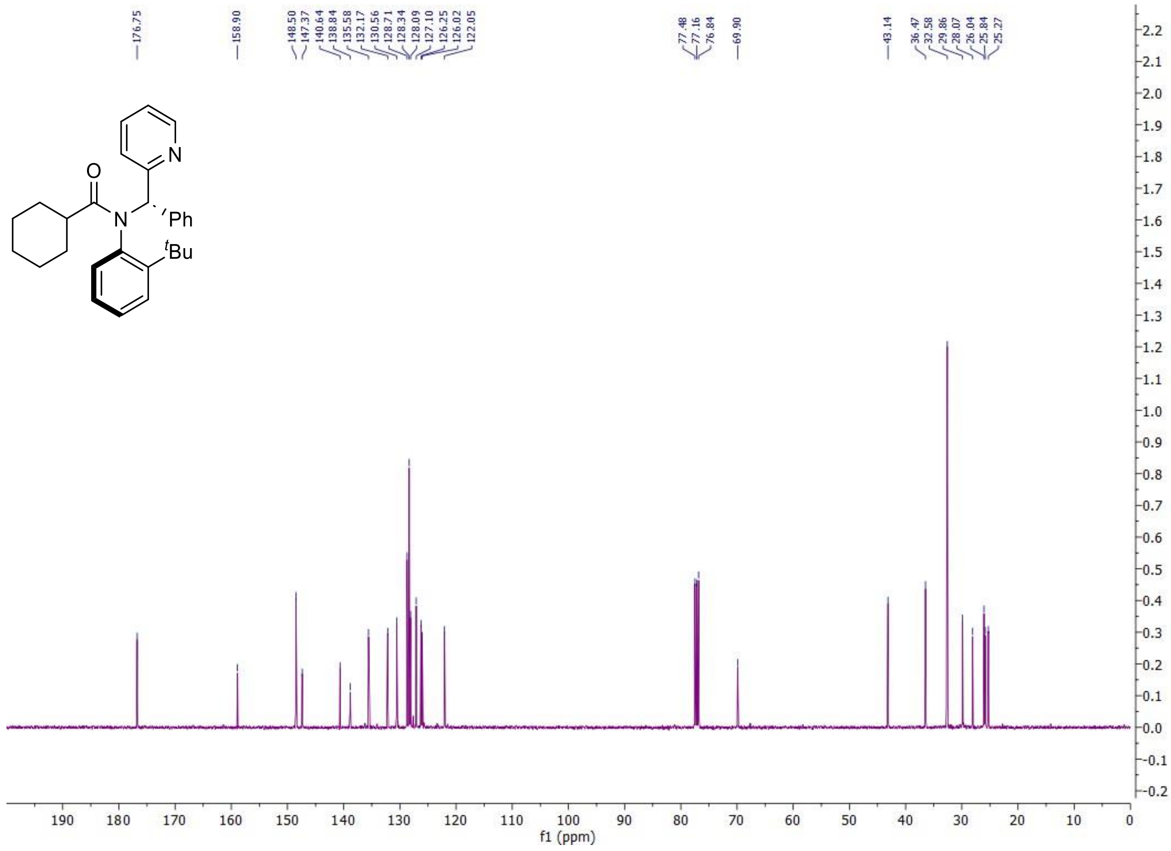

Benzyl 4-((2-(*tert*-butyl)phenyl)(phenyl(pyridin-2-yl)methyl)carbamoyl)piperidine-1-carboxylate (6x)

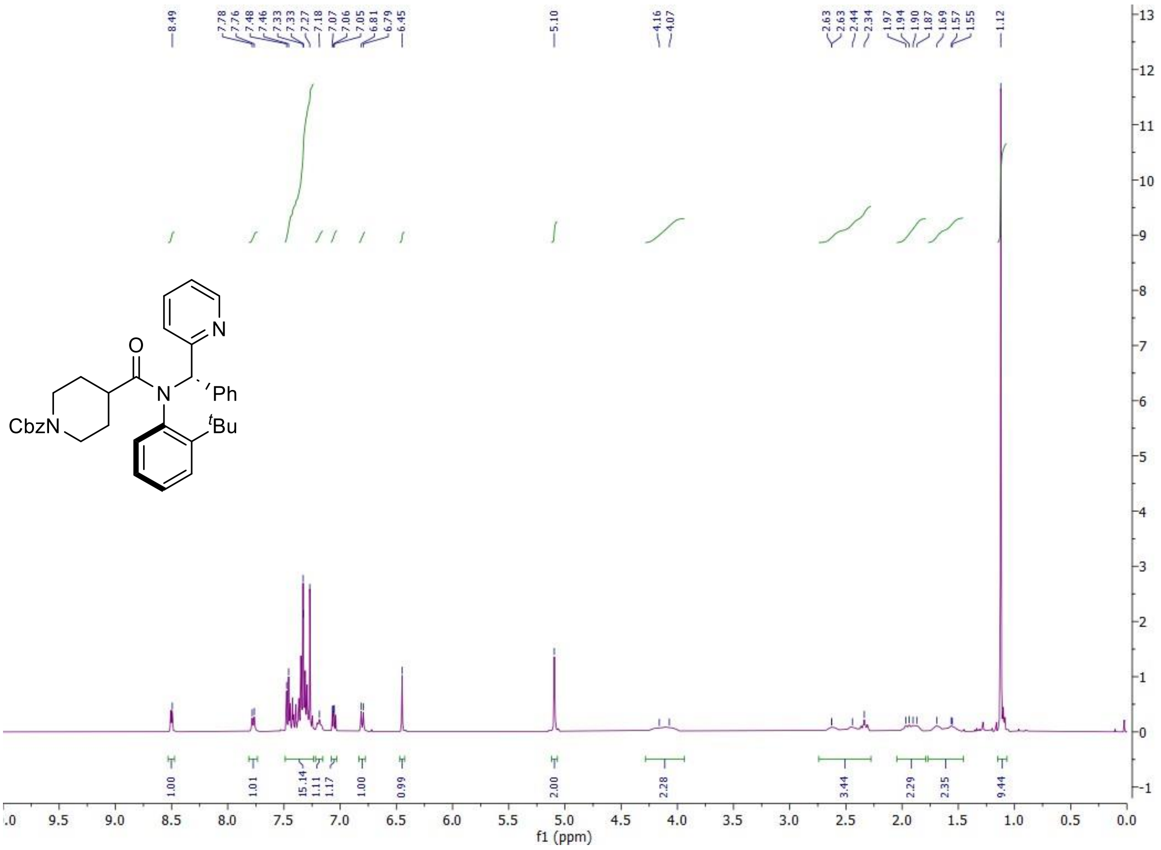

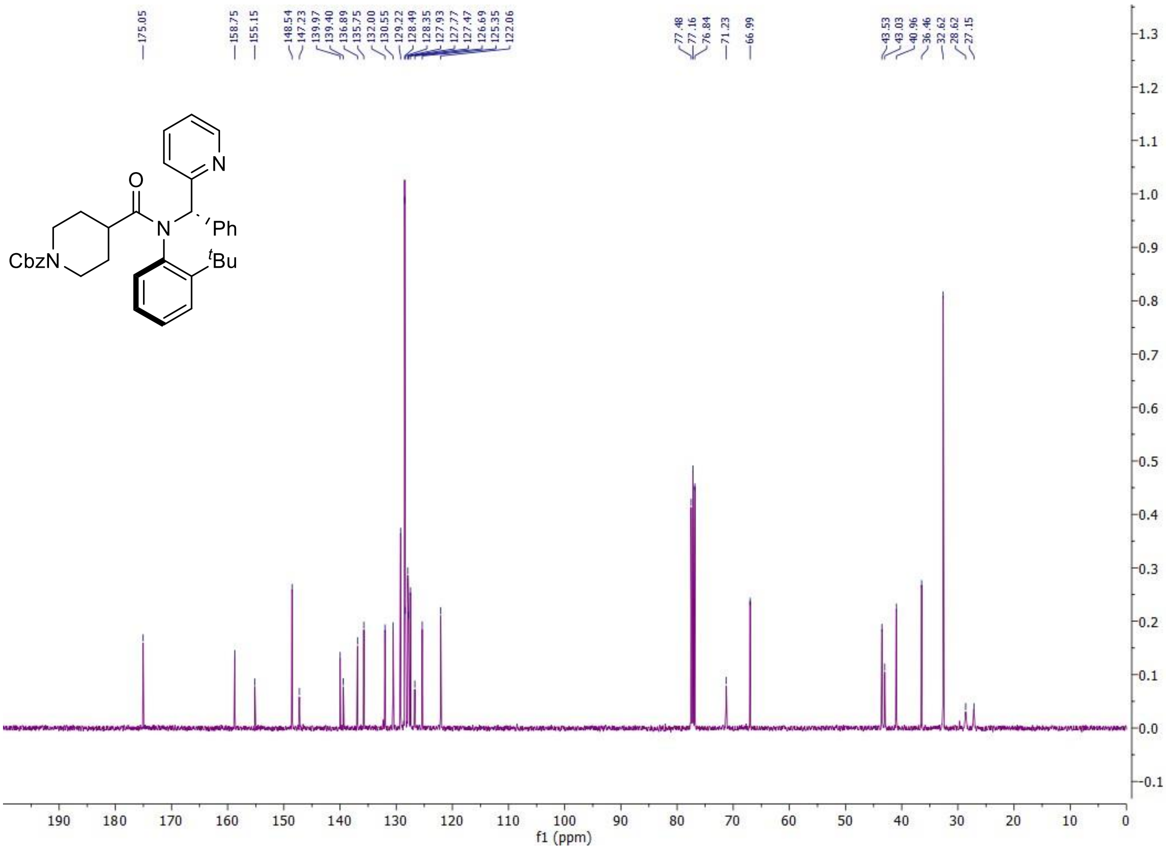

N-(2-(tert-Butyl)phenyl)-2-(1-(4-chlorobenzoyl)-5-methoxy-2-methyl-1H-indol-3-yl)-N-(phenyl(pyridin-2-yl)methyl)acetamide (6y)

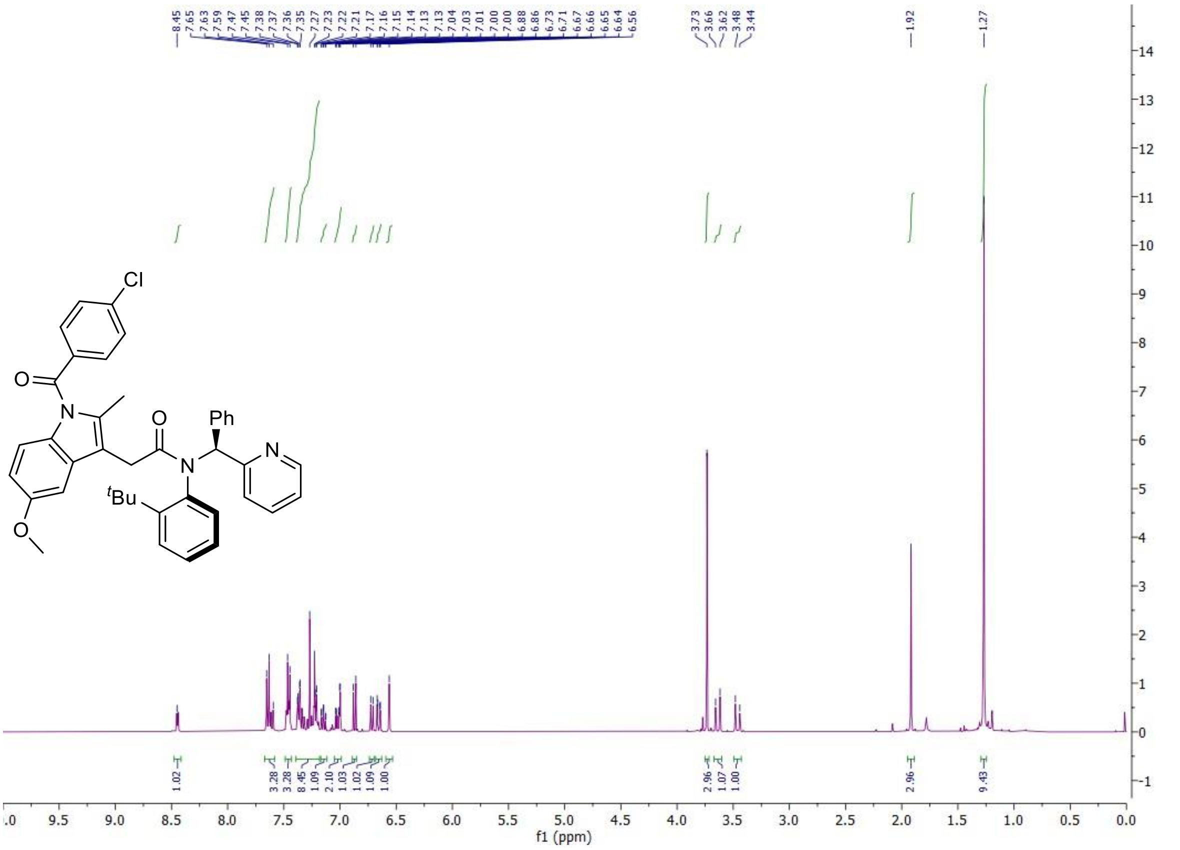

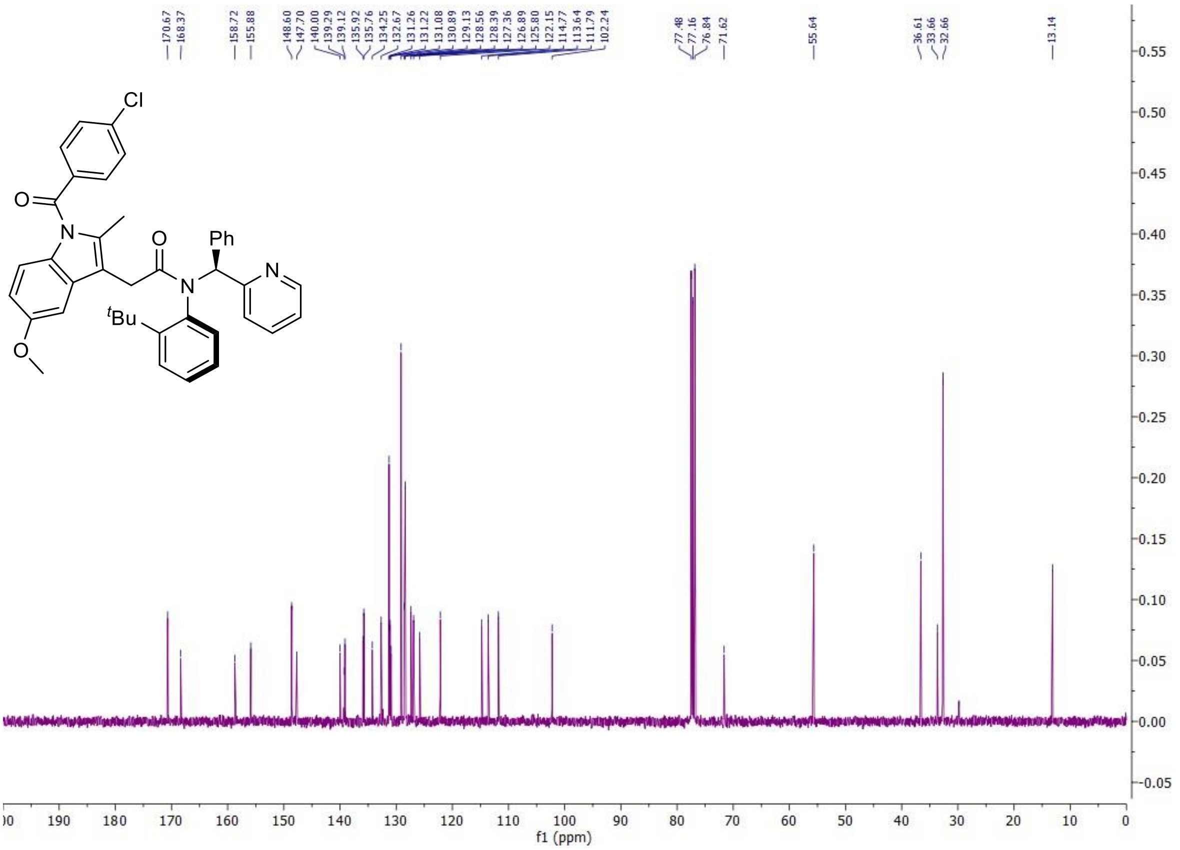

Methyl 5-((2-(*tert*-Butyl)phenyl)(phenyl(pyridin-2-yl)methyl)amino)-5-oxopentanoate (6z)

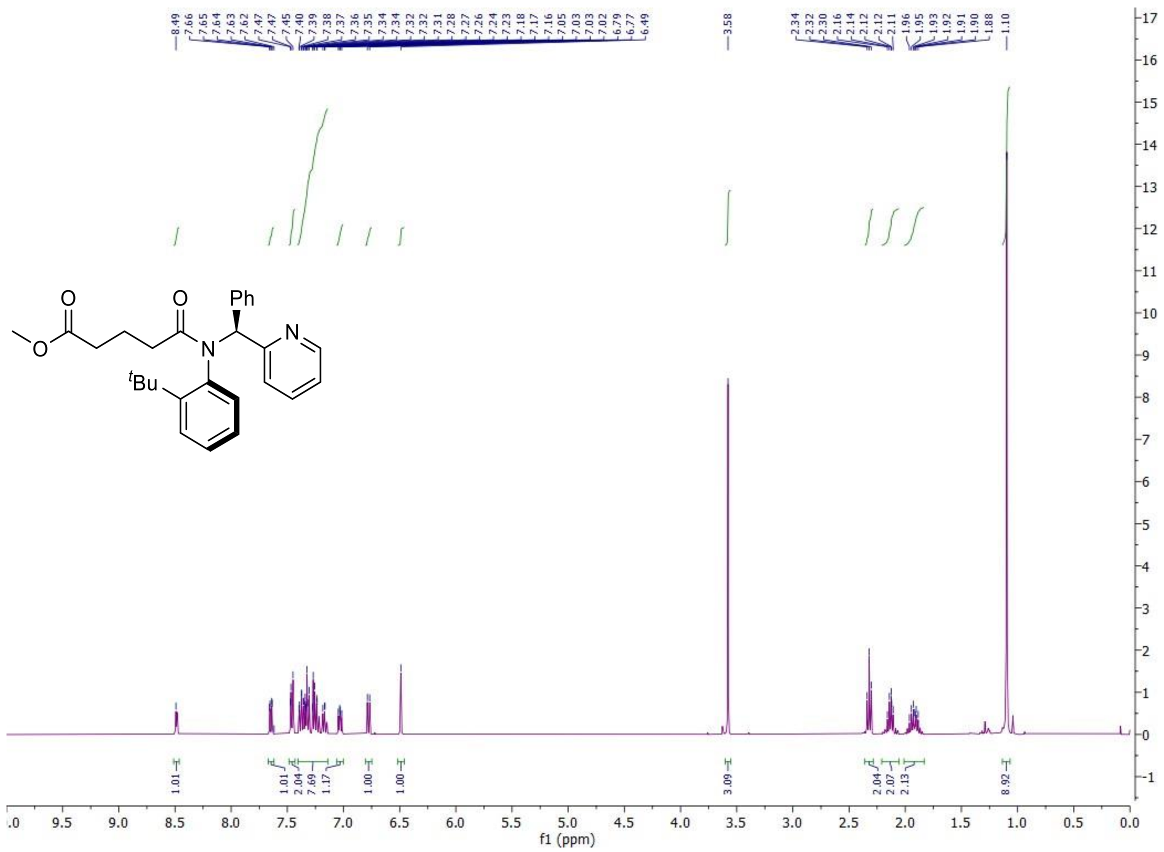

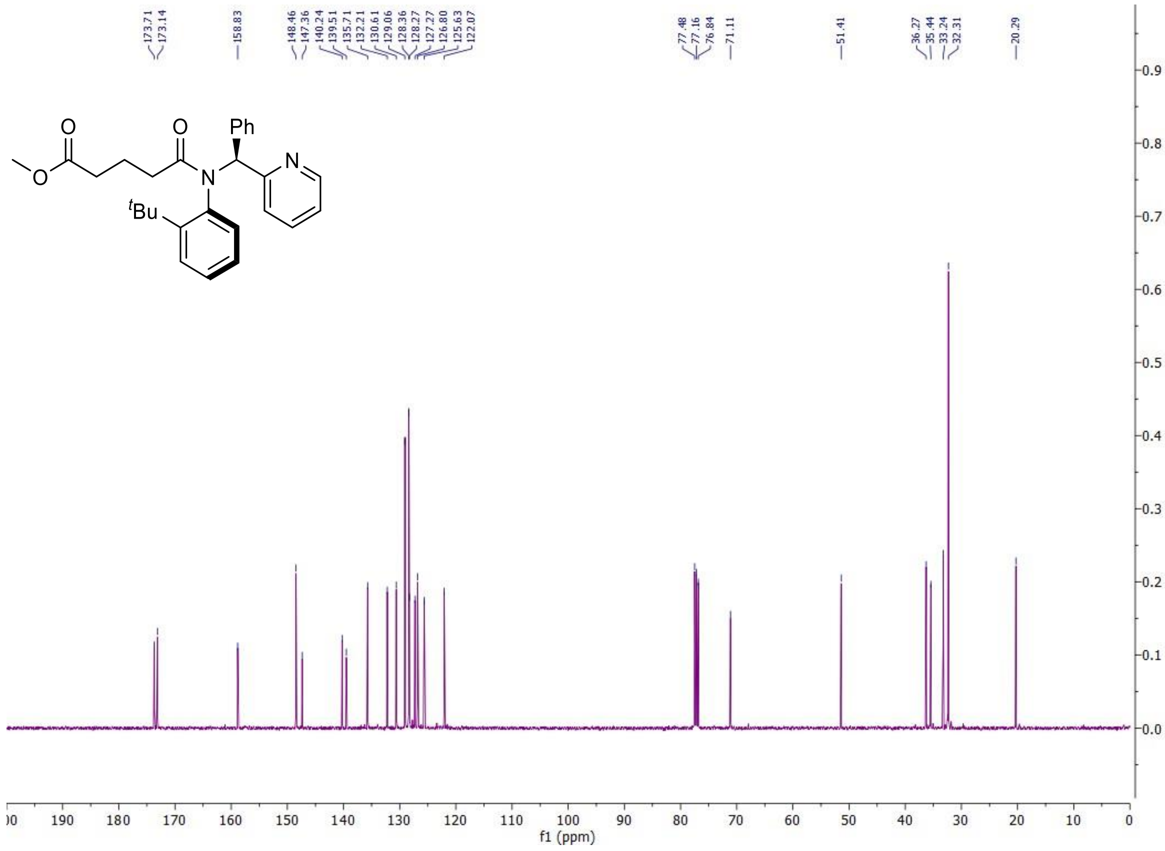

***N*-(2-(*tert*-Butyl)phenyl)-*N*-(phenyl(pyridin-2-yl)methyl)benzamide (6za)**

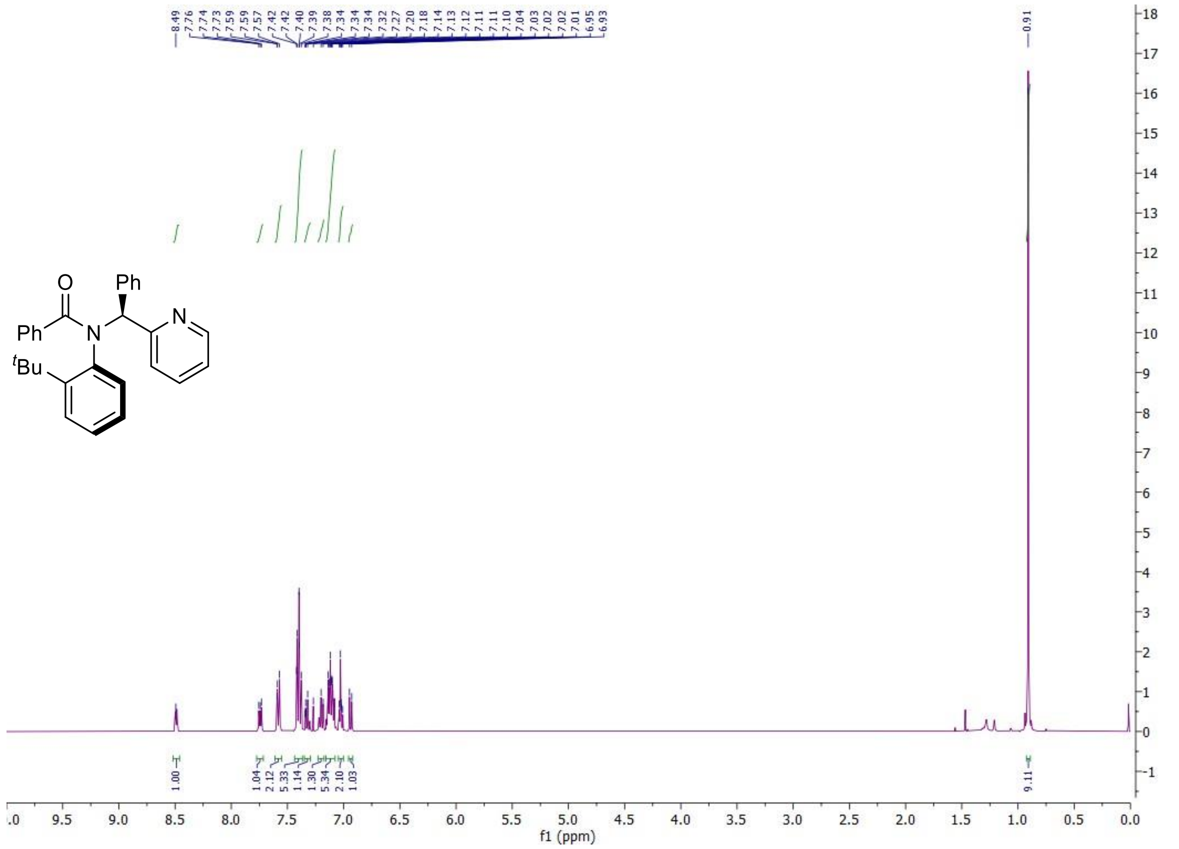

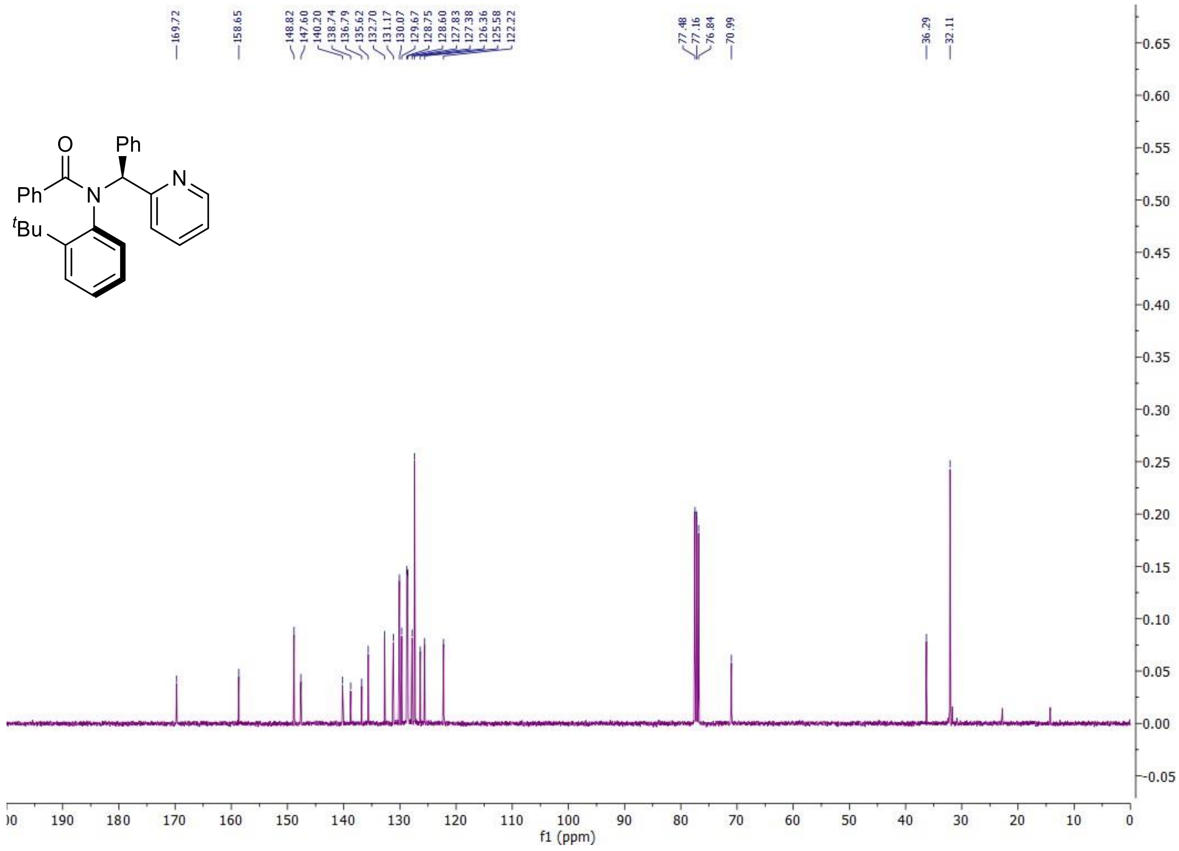

N-(2-(tert-Butyl)phenyl)-N-(1-(pyridin-2-yl)ethyl)benzamide (6zb)

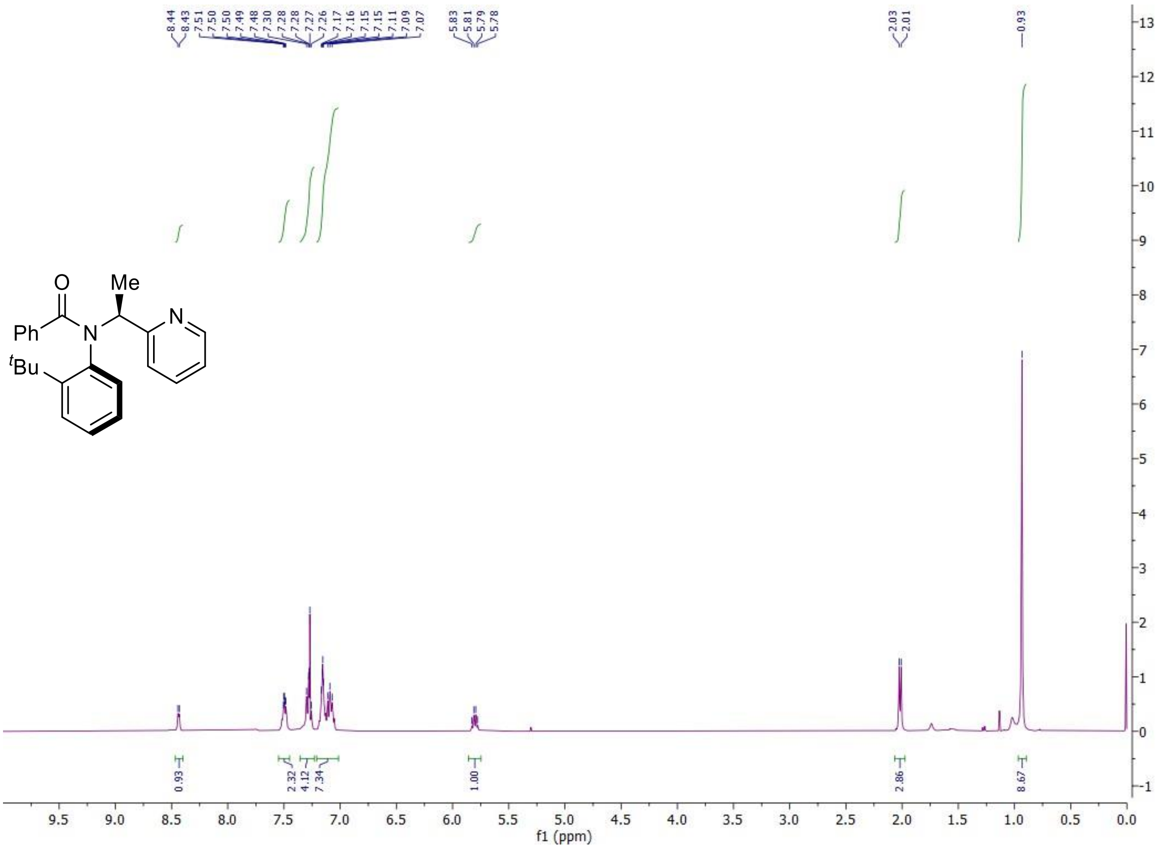

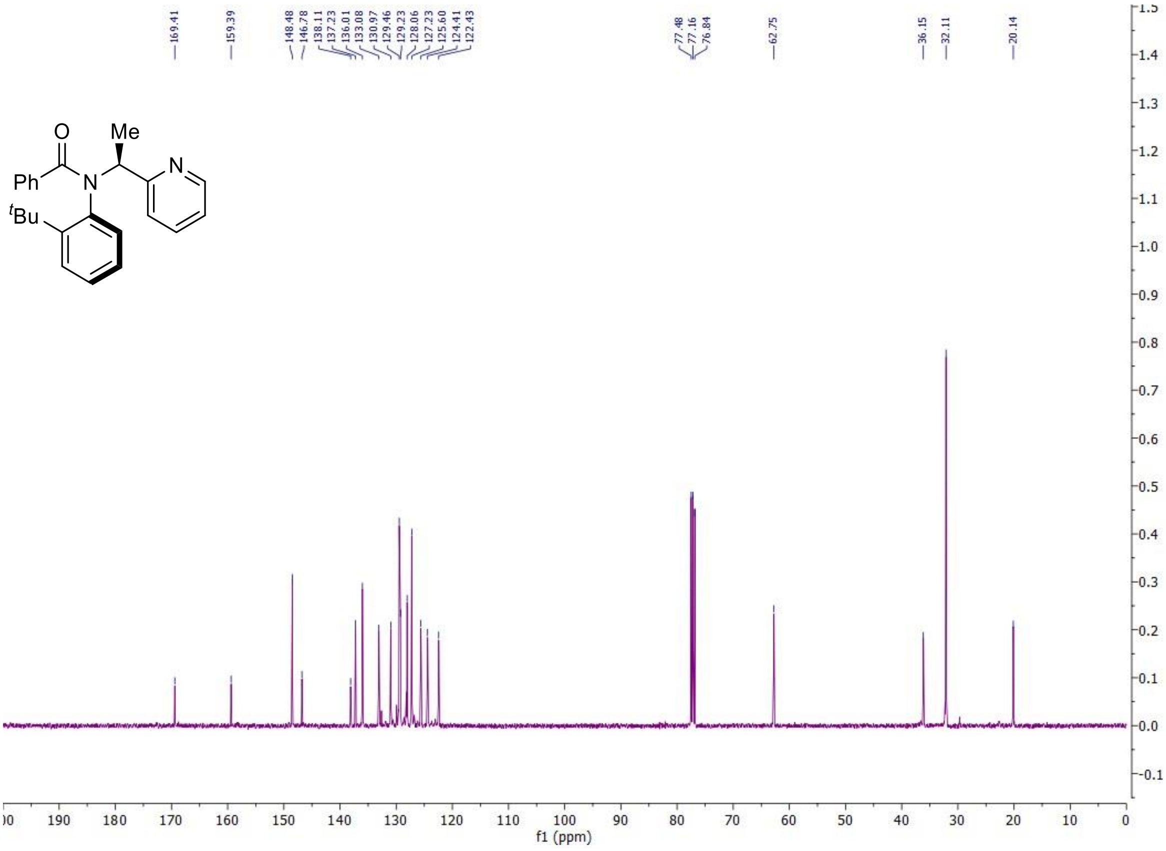

N-(2-(*tert*-Butyl)phenyl)-*N*-(1-(pyridin-2-yl)ethyl)benzamide (11zb)

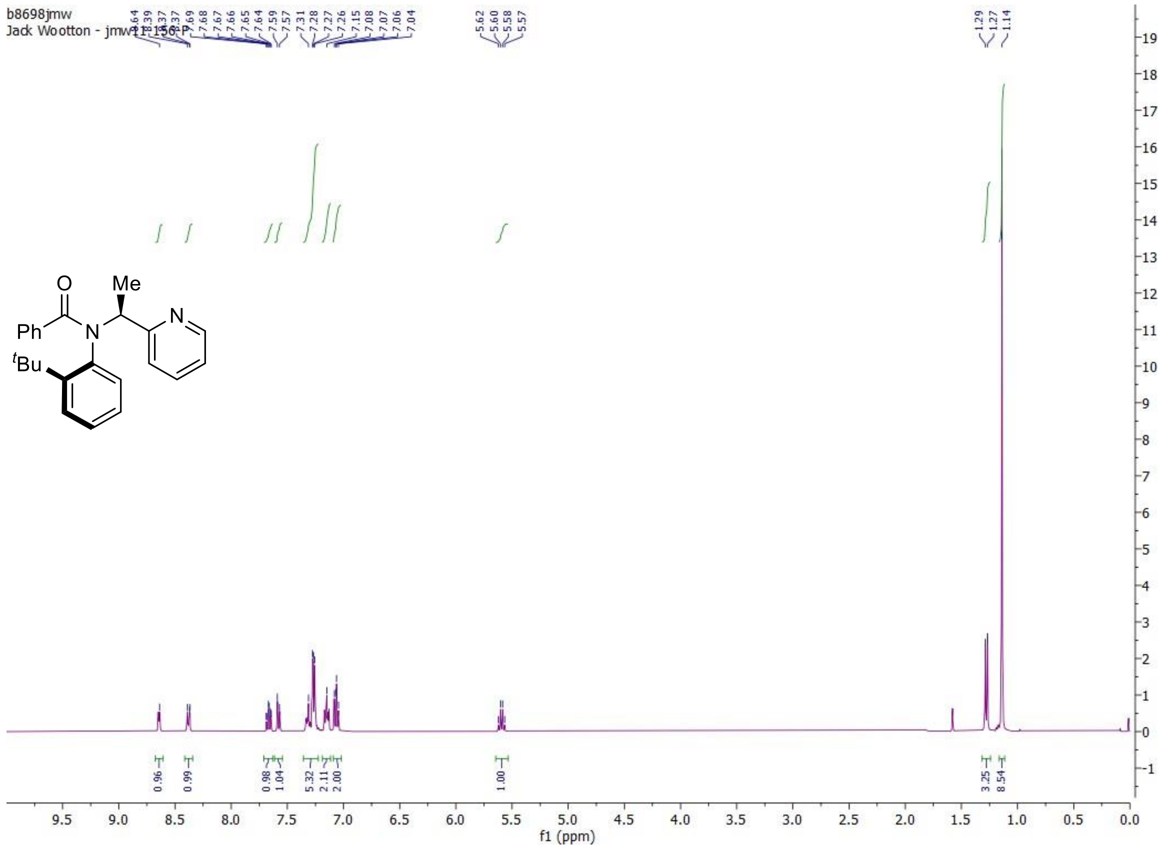

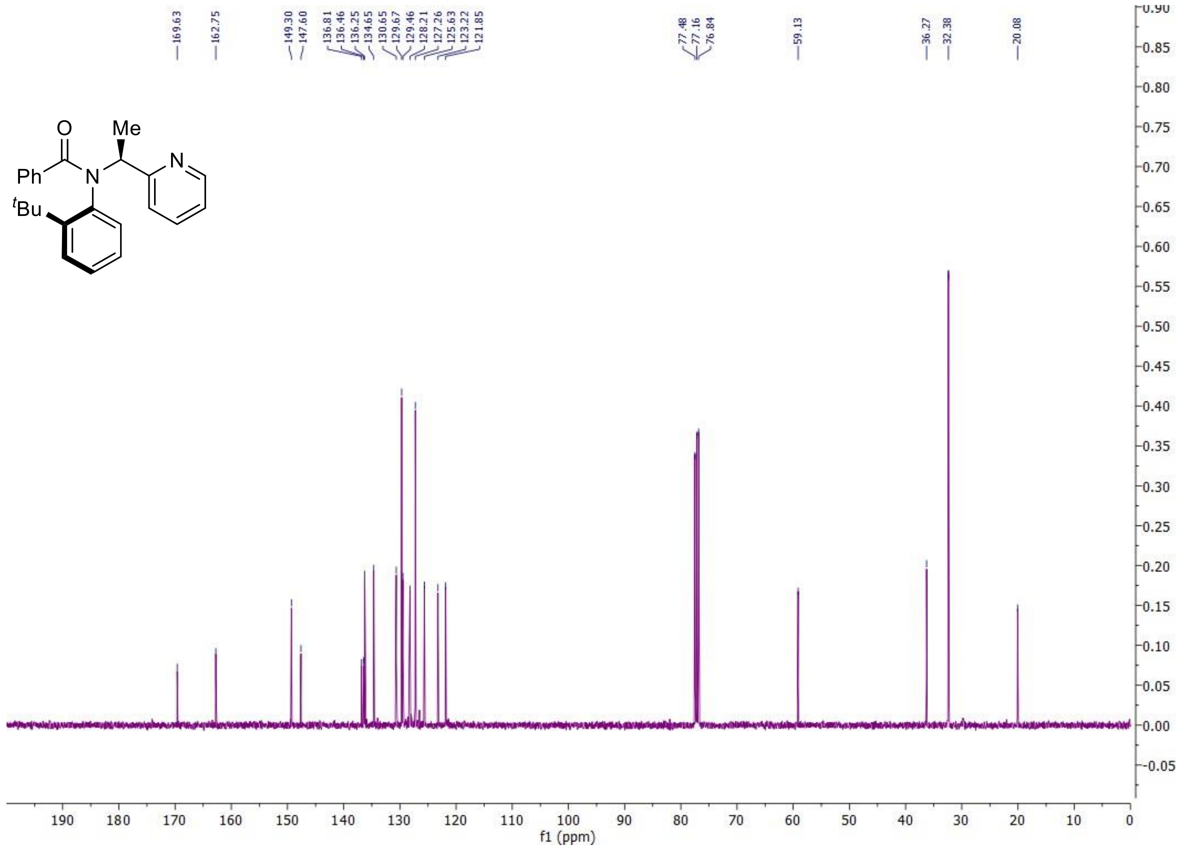

N-(2-(*tert*-Butyl)phenyl)-N-(1-(pyridin-2-yl)ethyl)tetrahydro-2*H*-pyran-4-carboxamide (6zc)

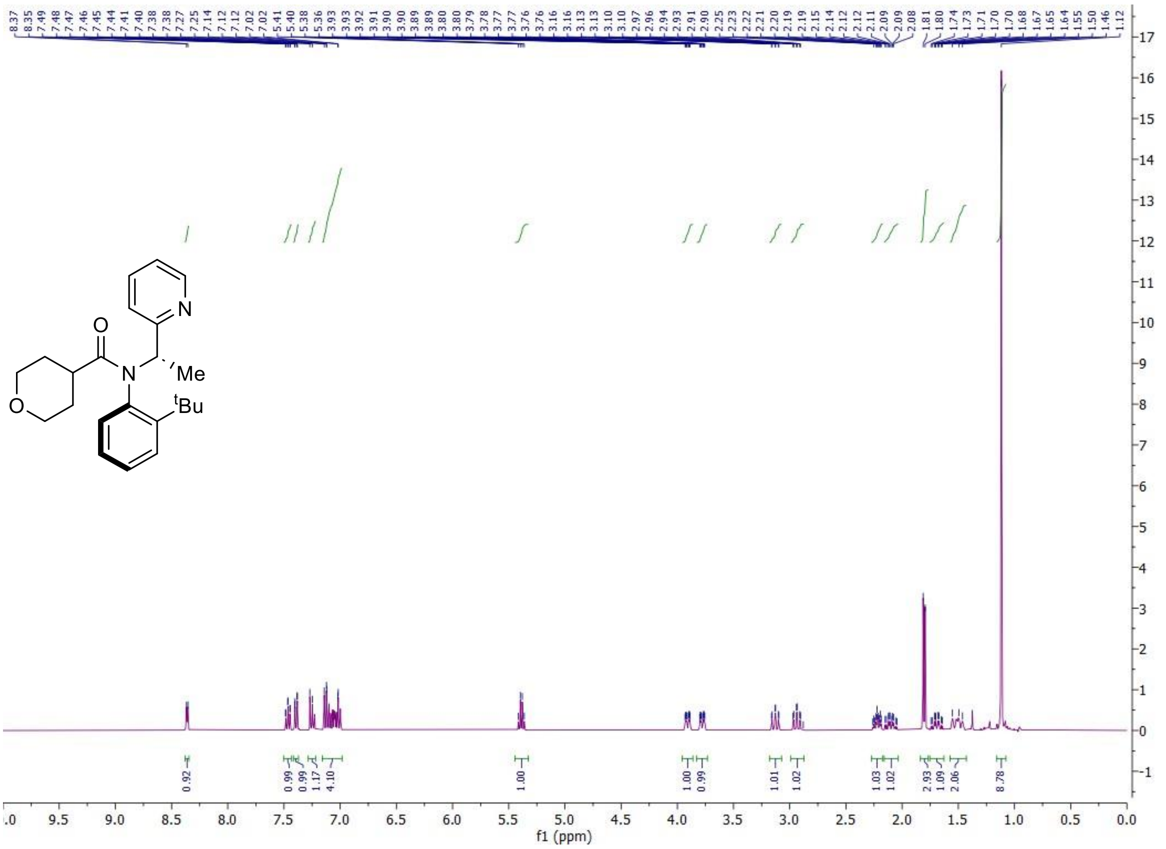

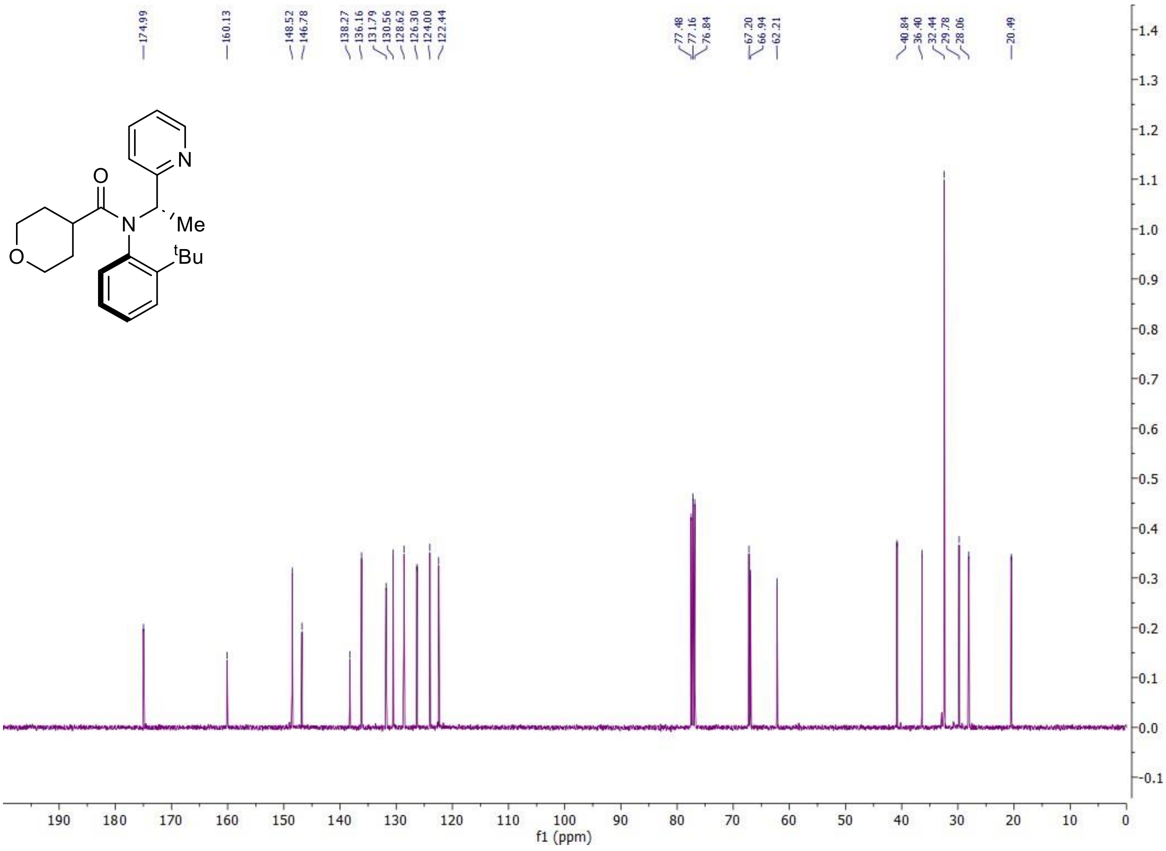

N-(2-(tert-Butyl)phenyl)-N-(1-(pyridin-2-yl)ethyl)propionamide (6zd)

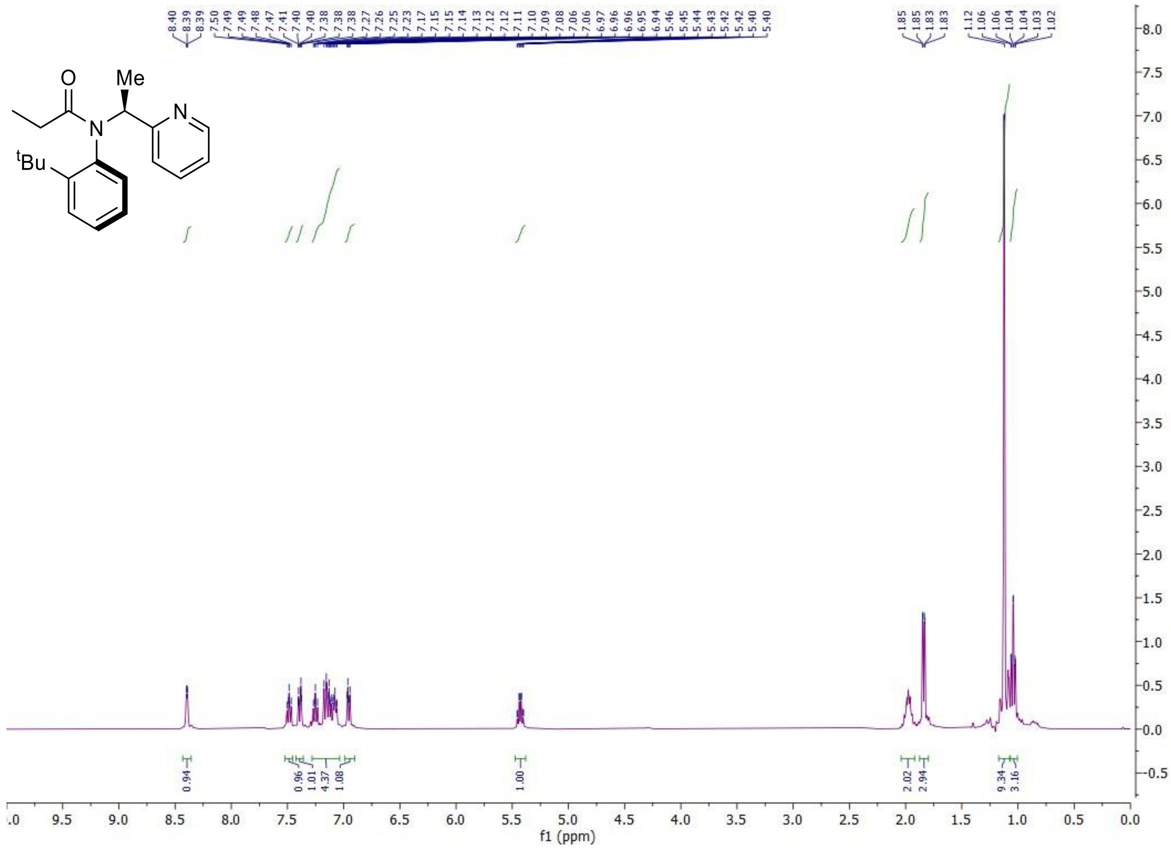

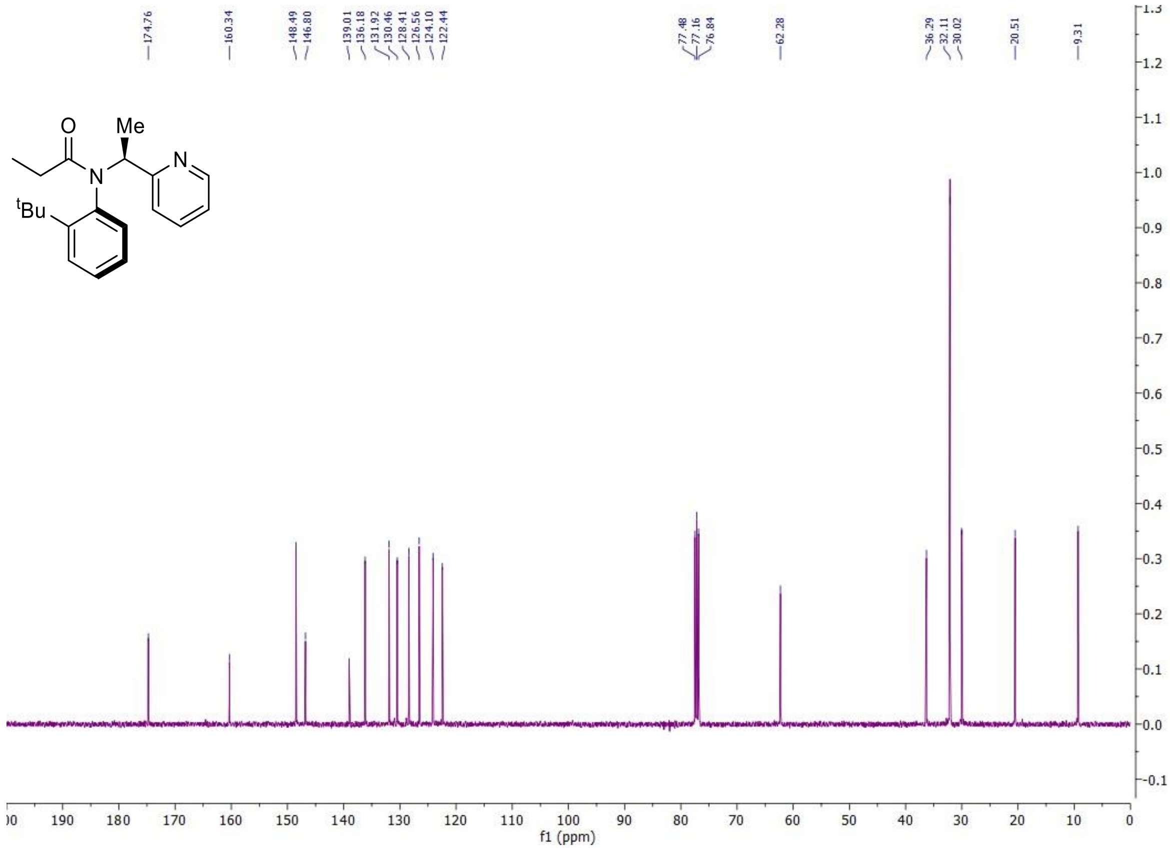

_N_-(2-(_tert_-Butyl)phenyl)-_N_-(1-(pyridin-2-yl)ethyl)propionamide (11zd)

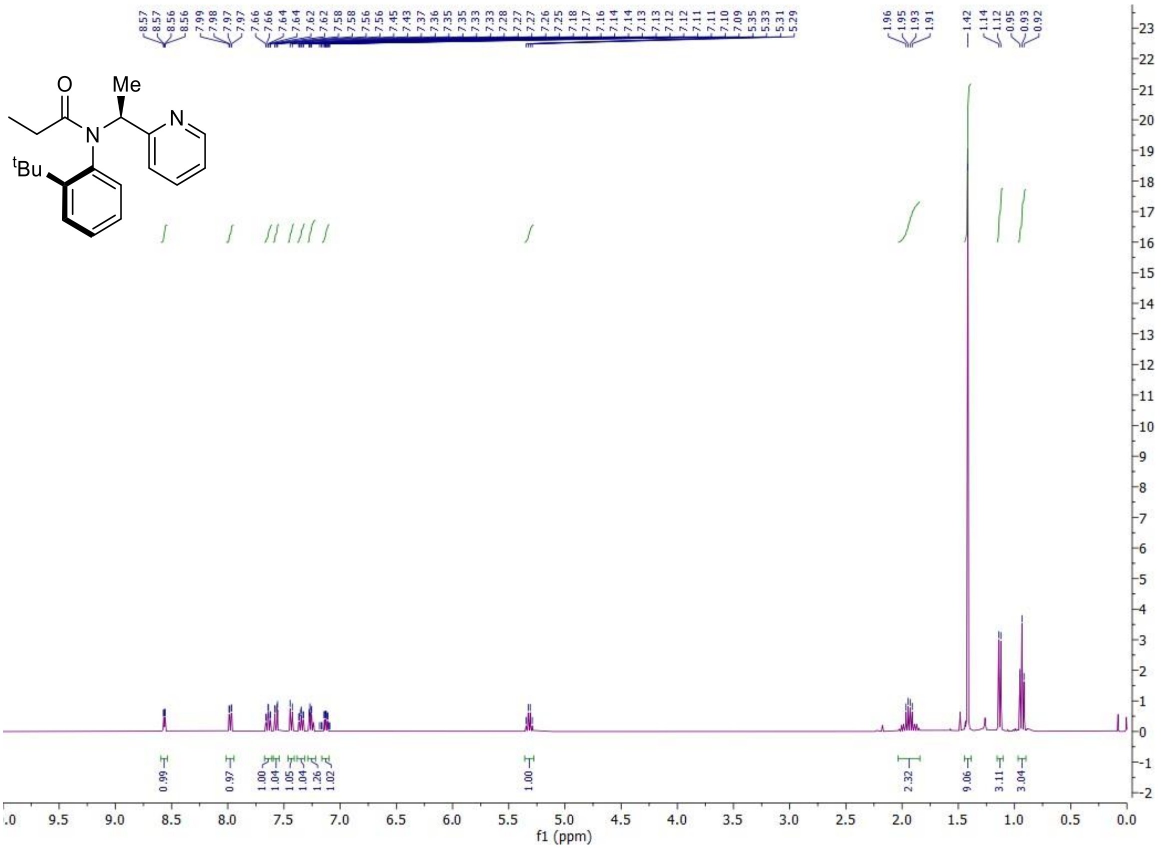

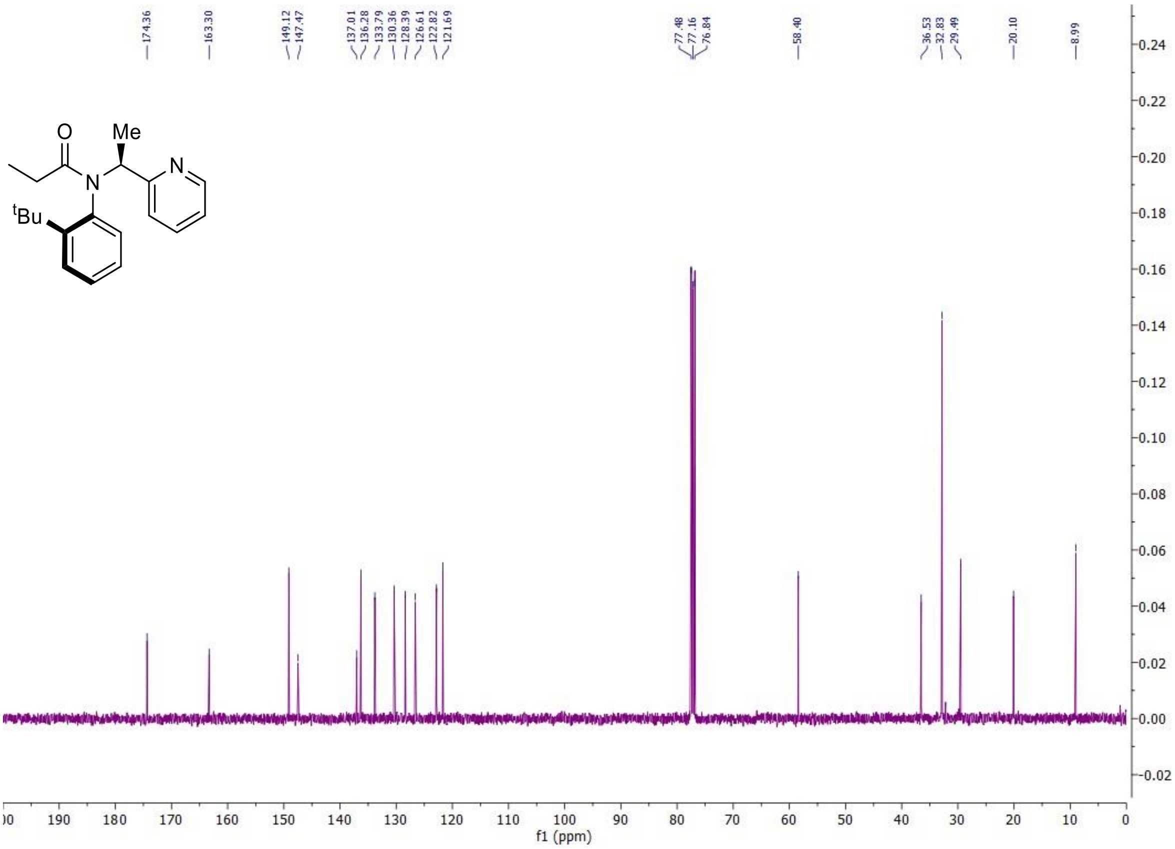

N-(2-(*tert*-Butyl)phenyl)-4-iodo-*N*-(1-(pyridin-2-yl)ethyl)benzamide (6ze)

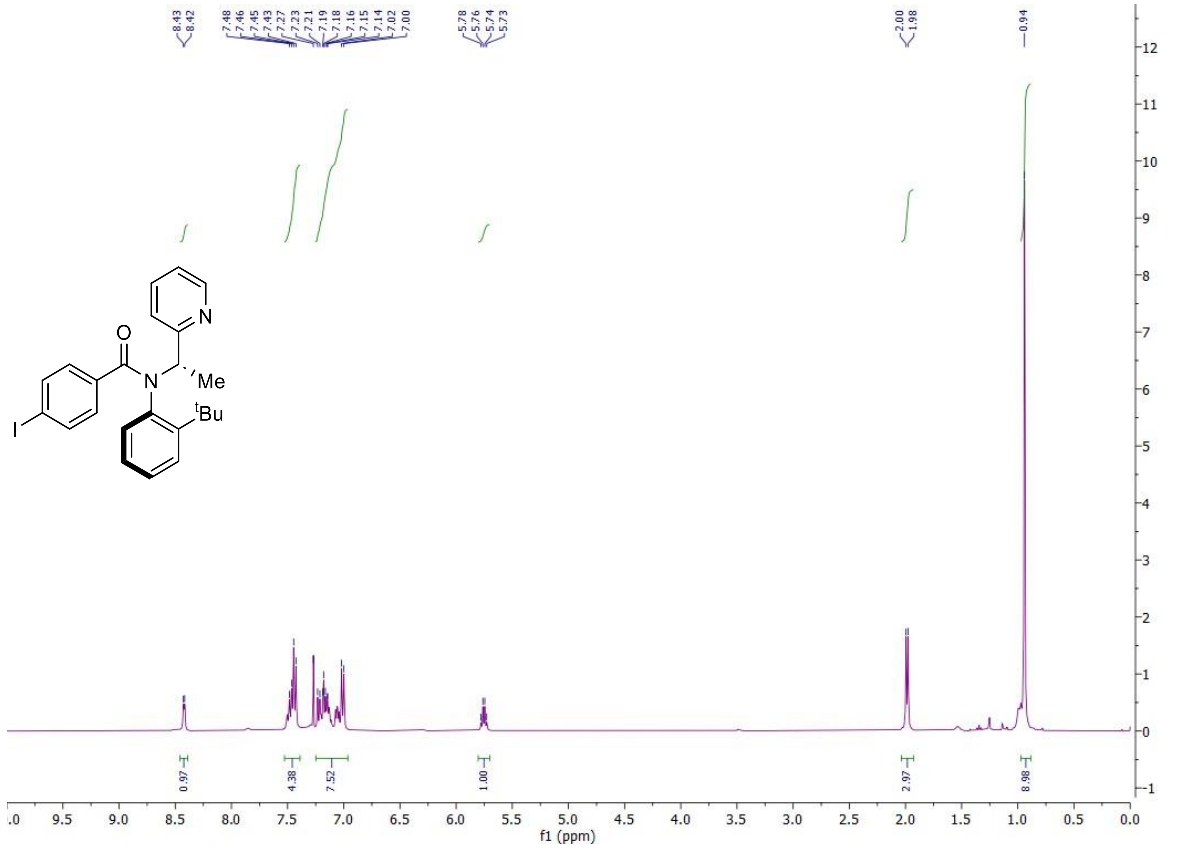

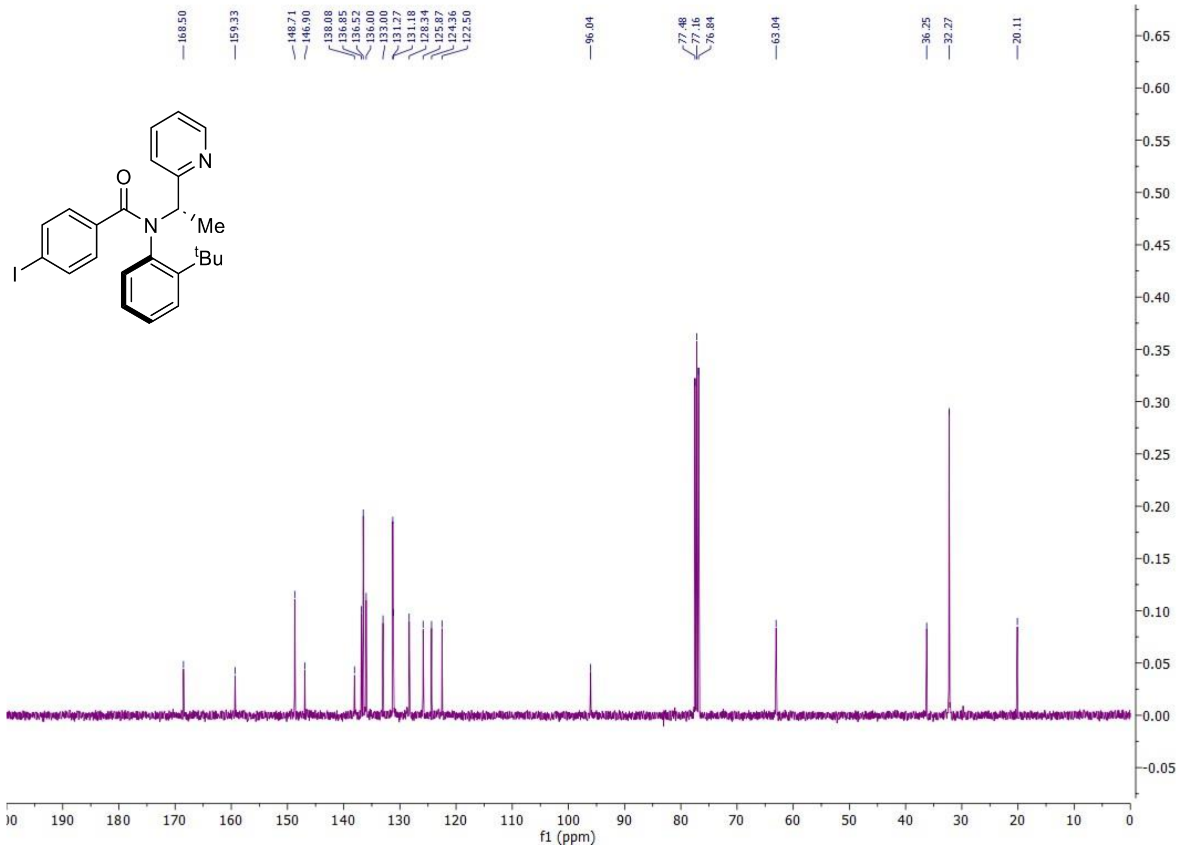

2-(*tert*-Butyl)-*N*-(1-phenylethyl)aniline (14)

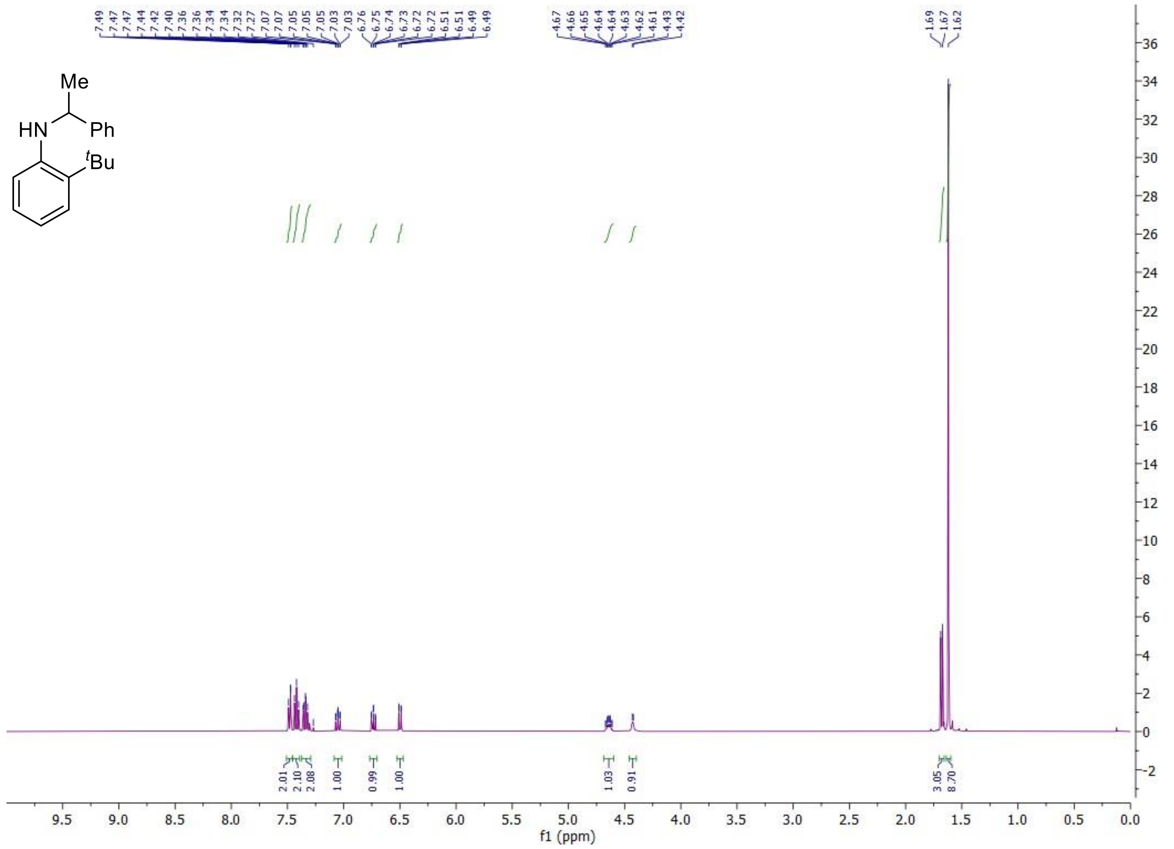

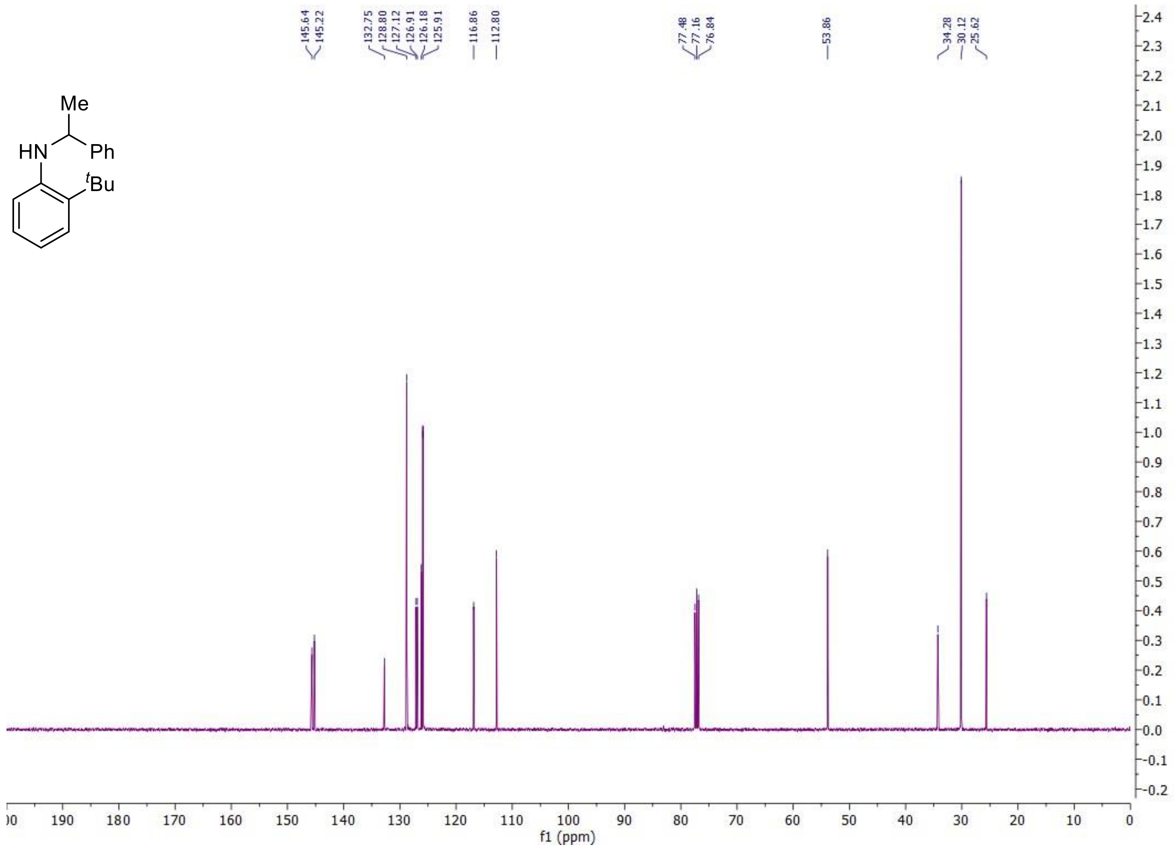

N-(2-(tert-Butyl)phenyl)-N-(1-phenylethyl)propionamide (15)

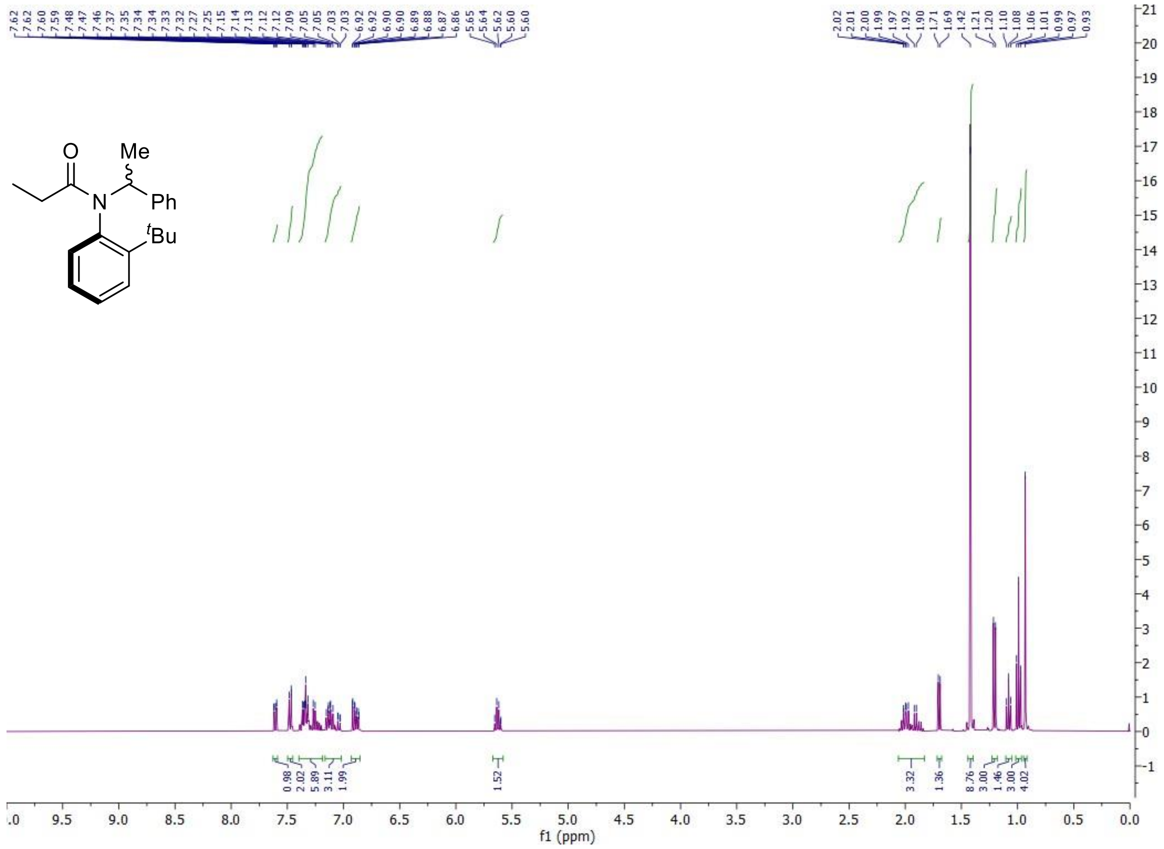

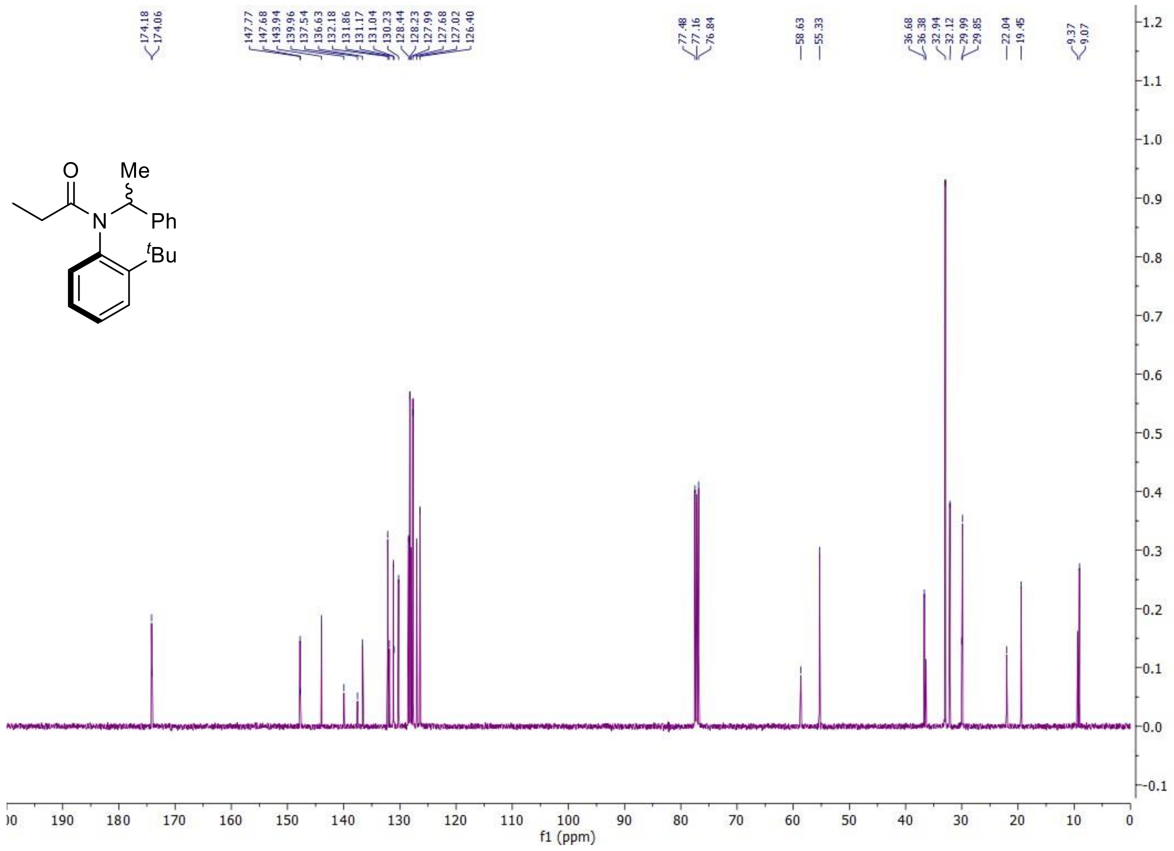

2-Iodo-4,6-dimethylaniline (S5)

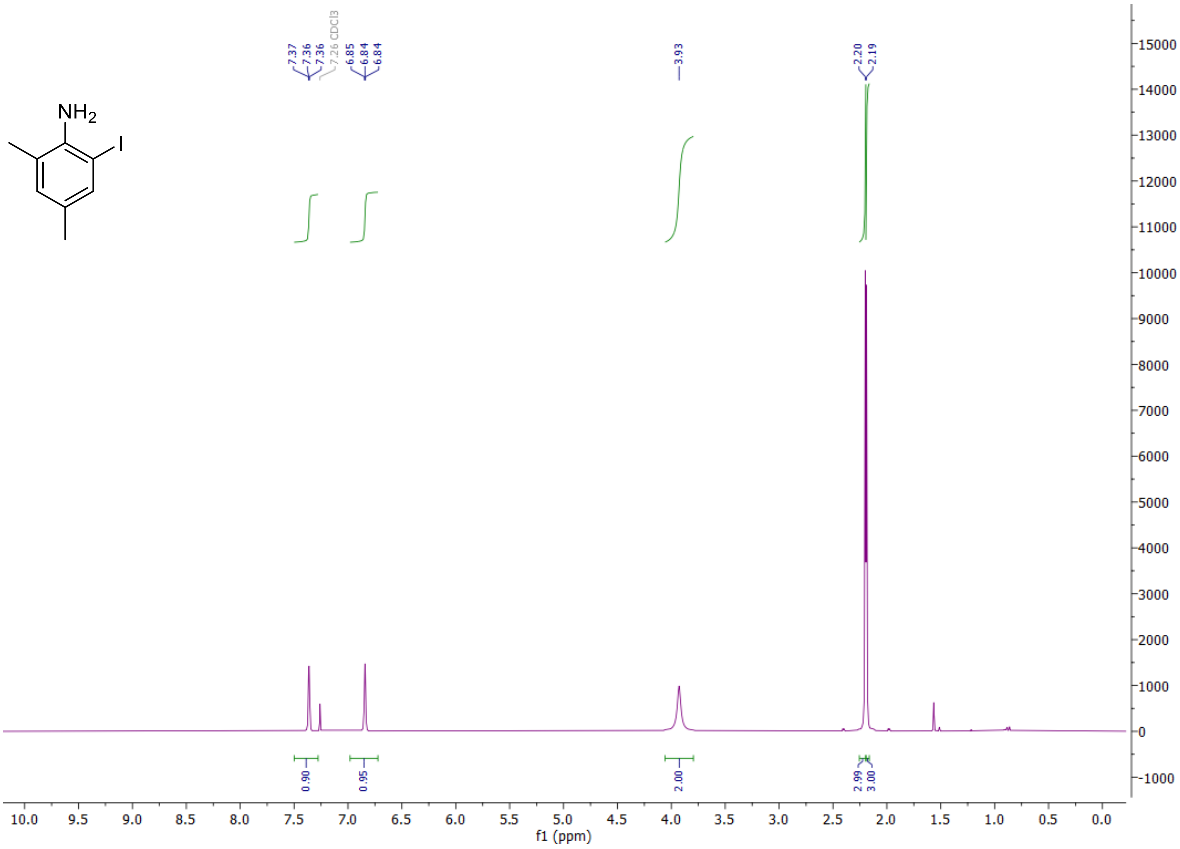

N-(2-Iodo-4,6-dimethylphenyl)-1-phenylmethanimine (S6)

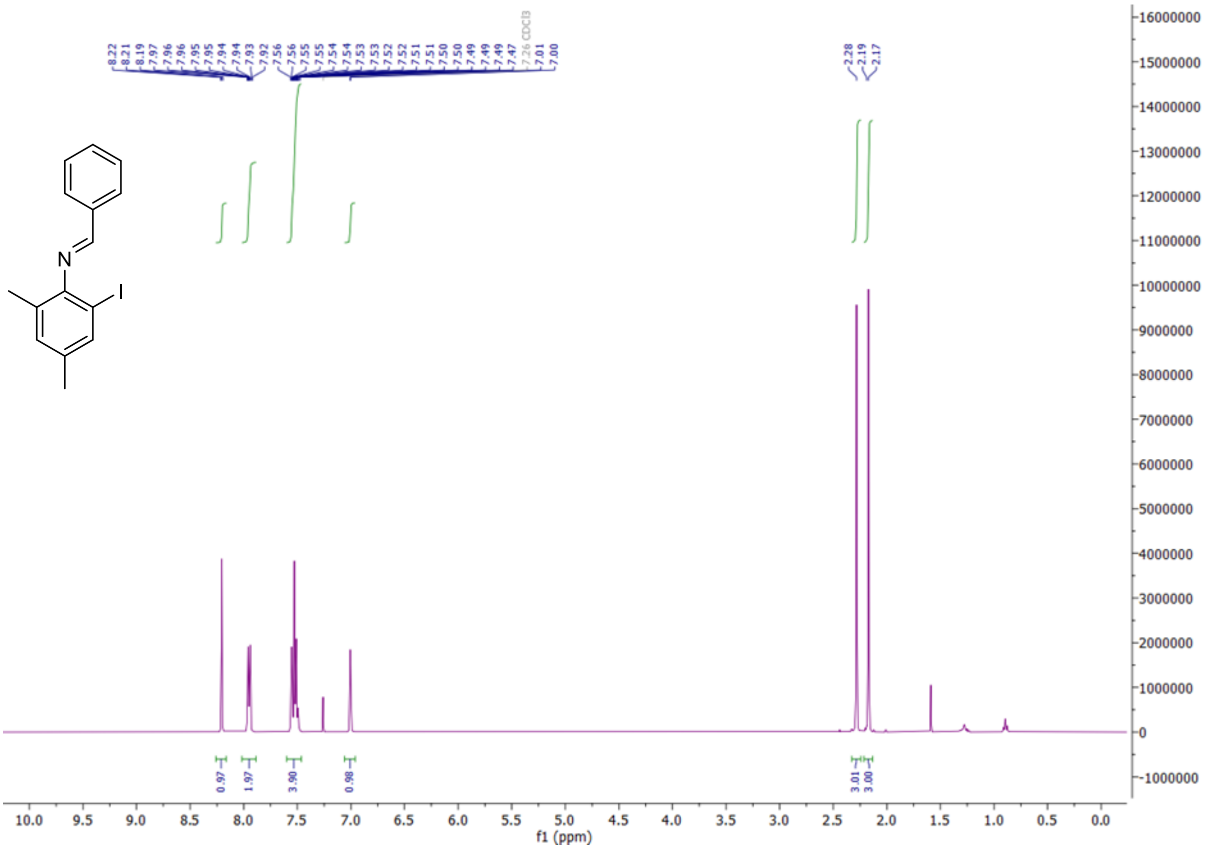

(2-Bromo-4,6-dimethylphenyl)-1-phenylmethanimine (S7)

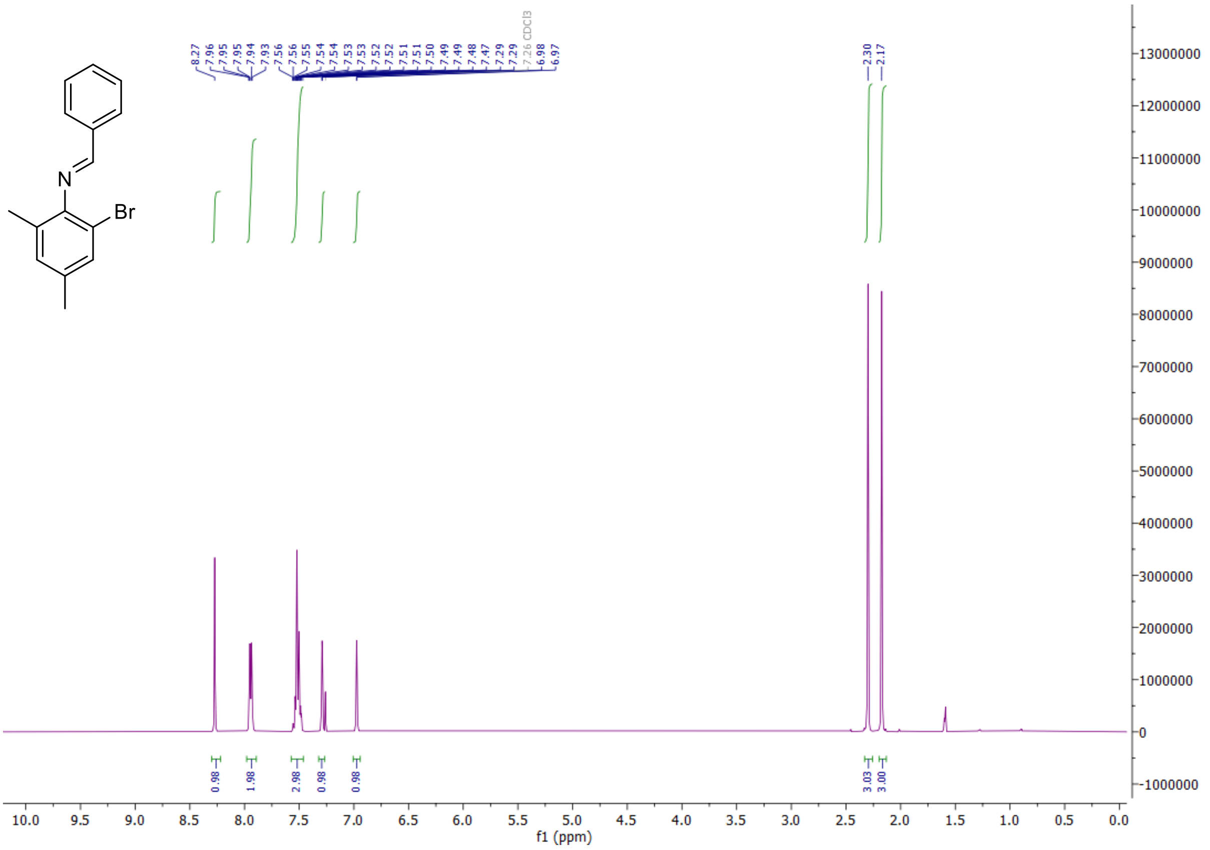

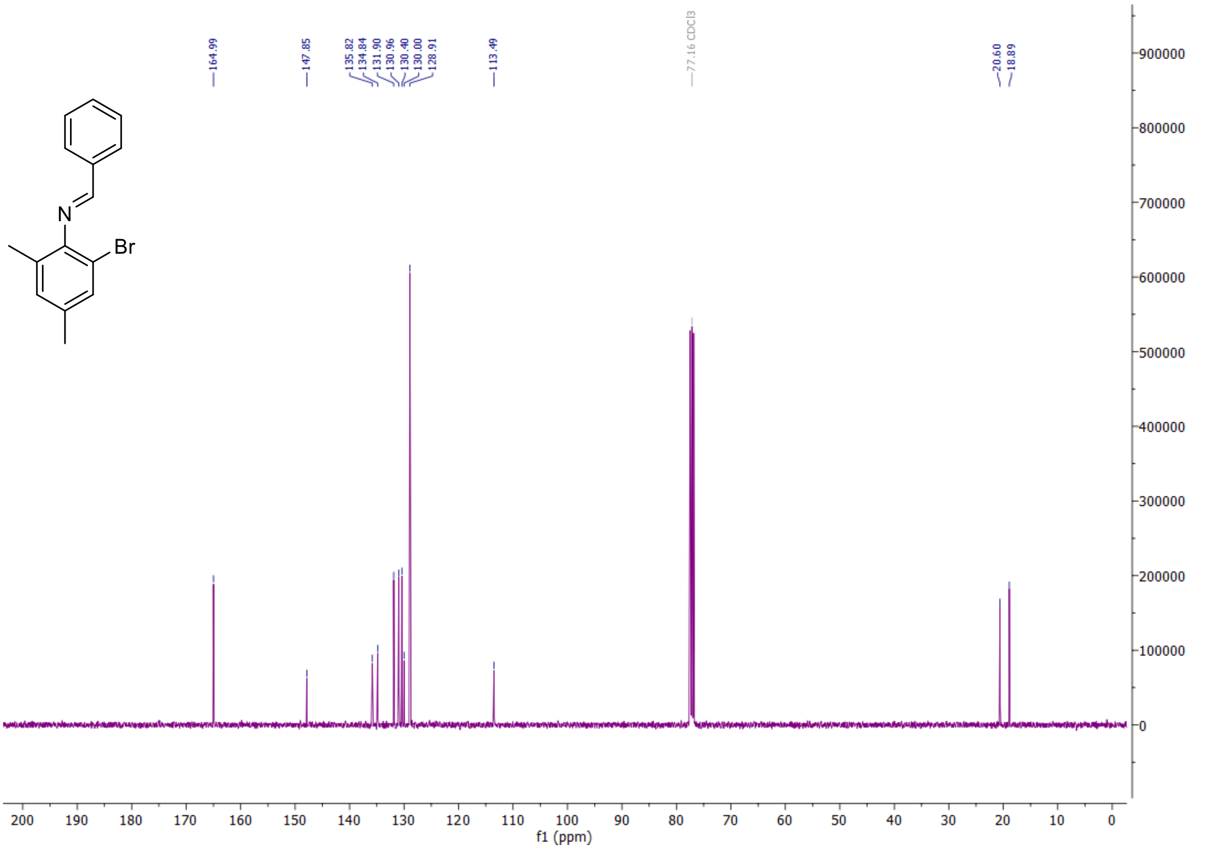

3,5-Dimethyl-[1,1'-biphenyl]-2-amine (S8)

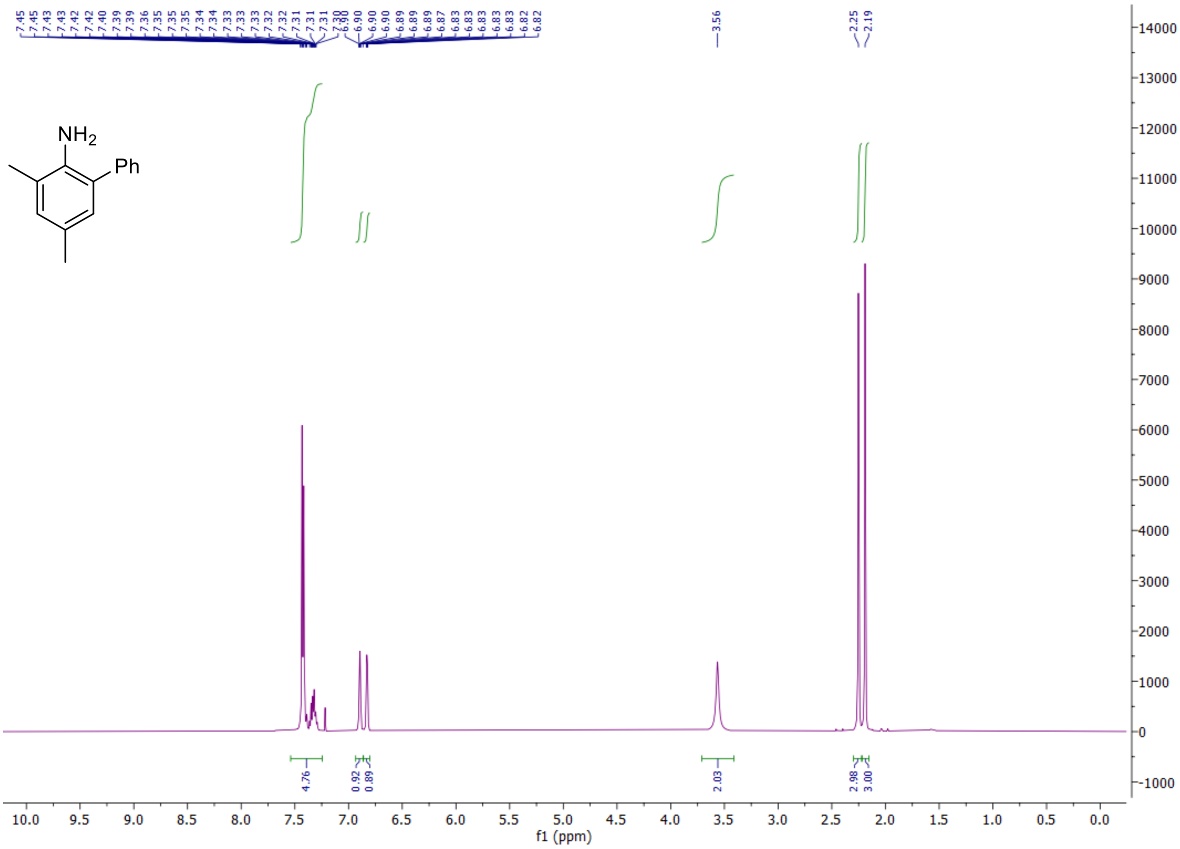

***N*-(5'-(*tert*-Butyl)-[1,1':3',1''-terphenyl]-4'-yl)-1-phenylmethanimine (S9)**

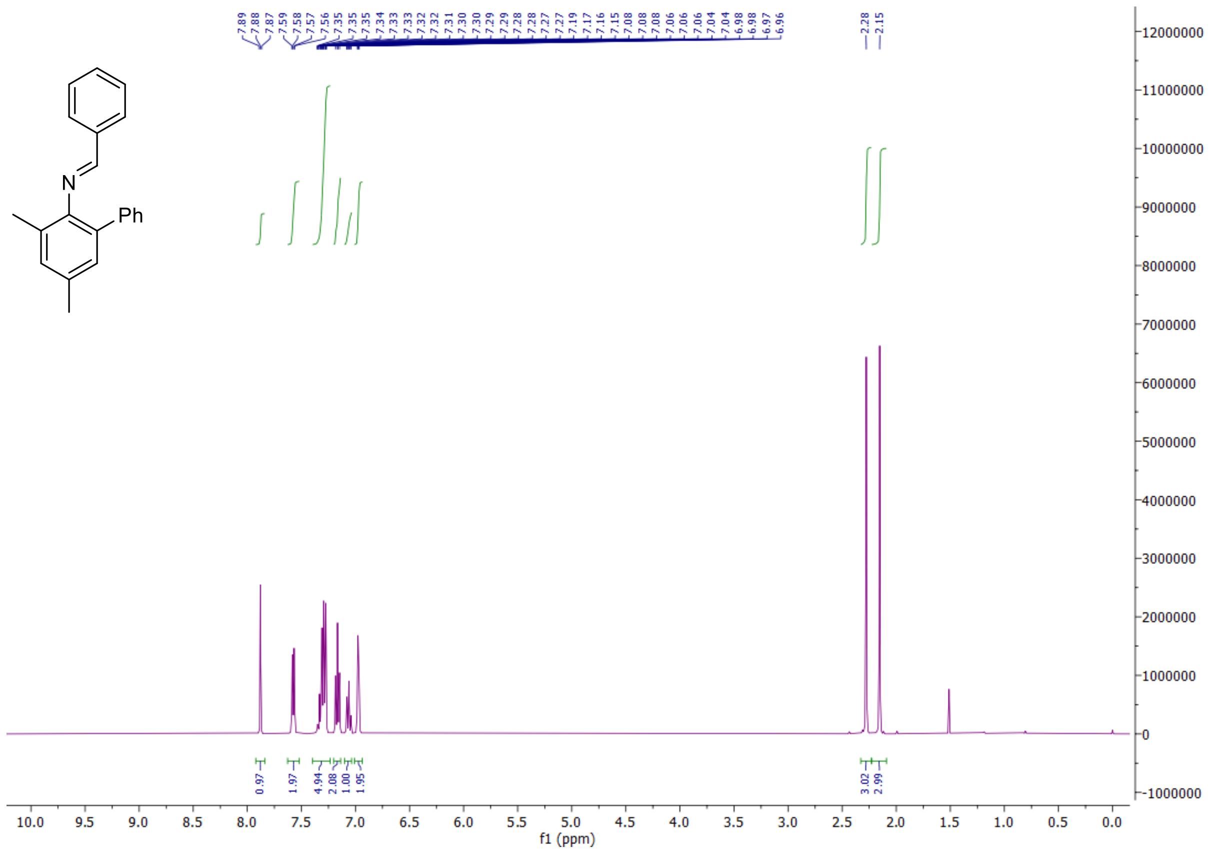

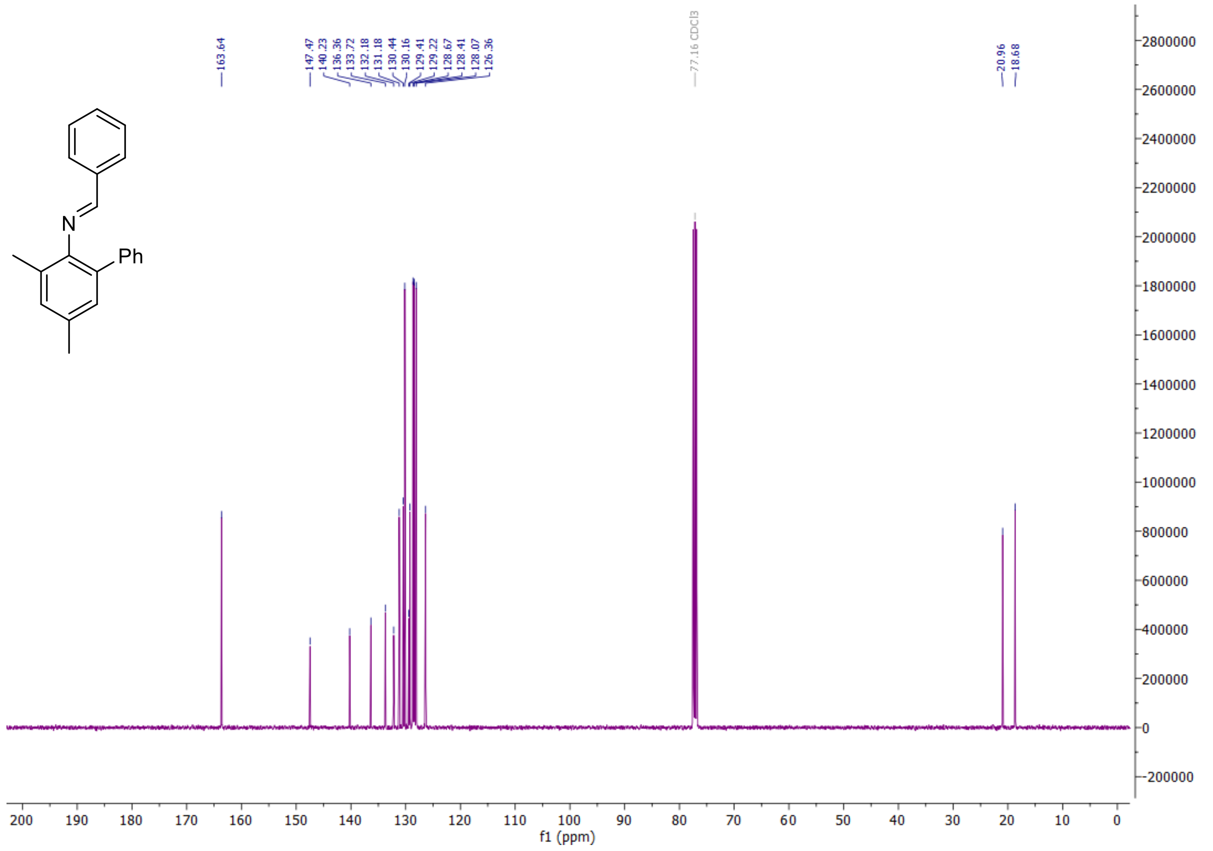

2-Iodo-4,6-dimethyl-*N*-(1-phenyl-2-(pyridin-2-yl)ethyl)aniline (4e)

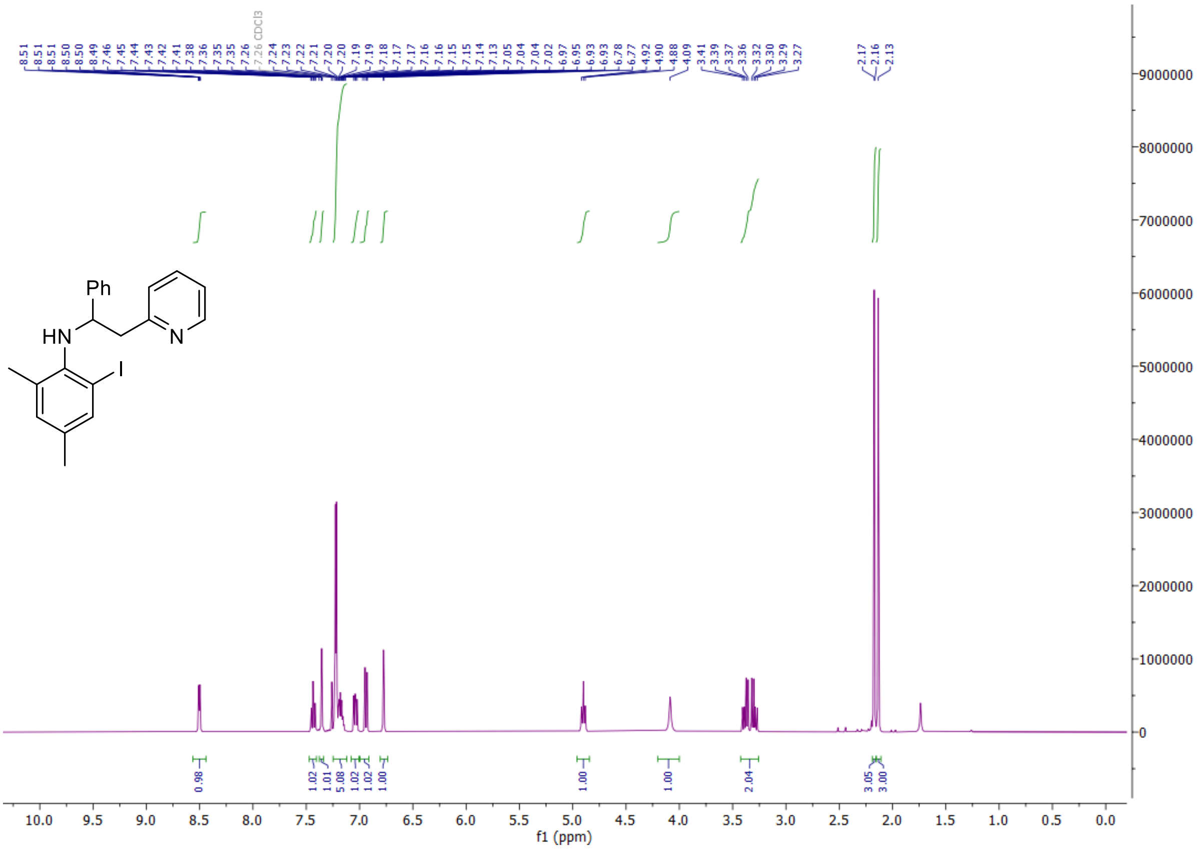

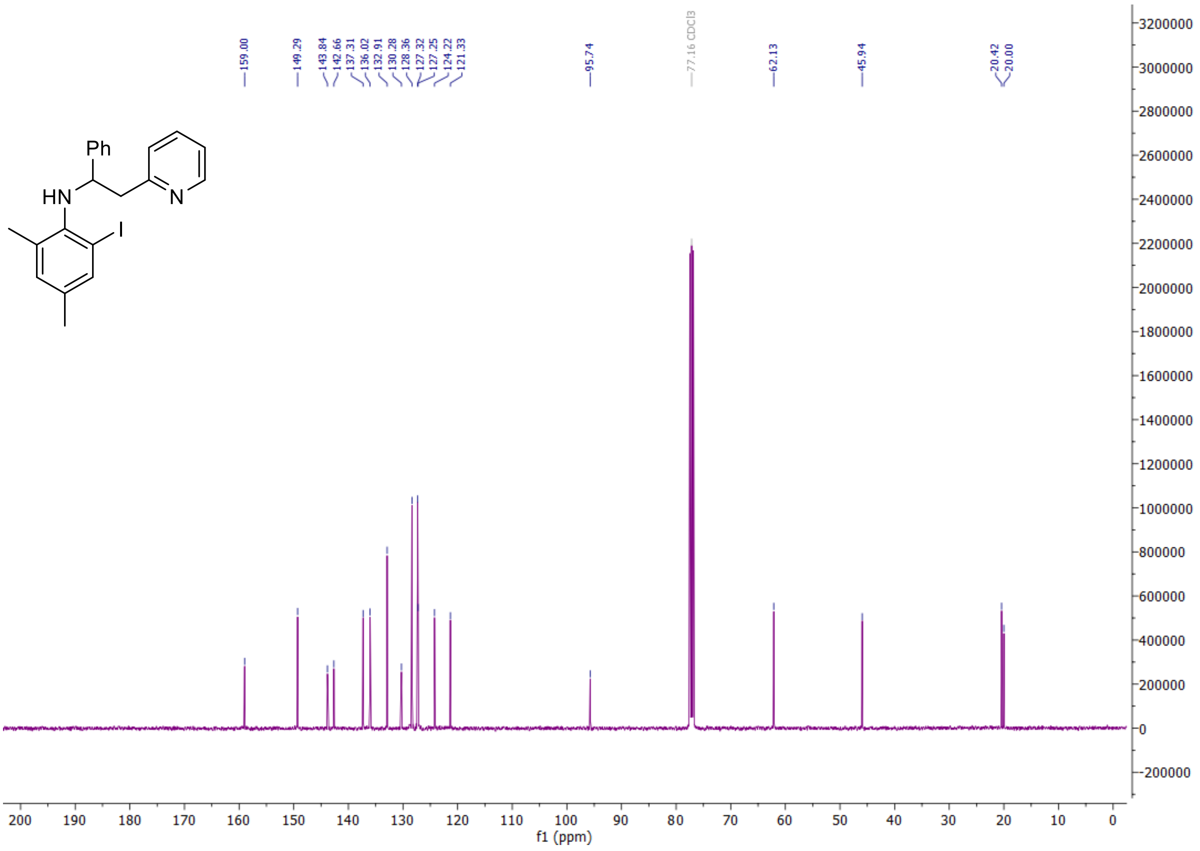

2-Bromo-4,6-dimethyl-*N*-(1-phenyl-2-(pyridin-2-yl)ethyl)aniline (4f)

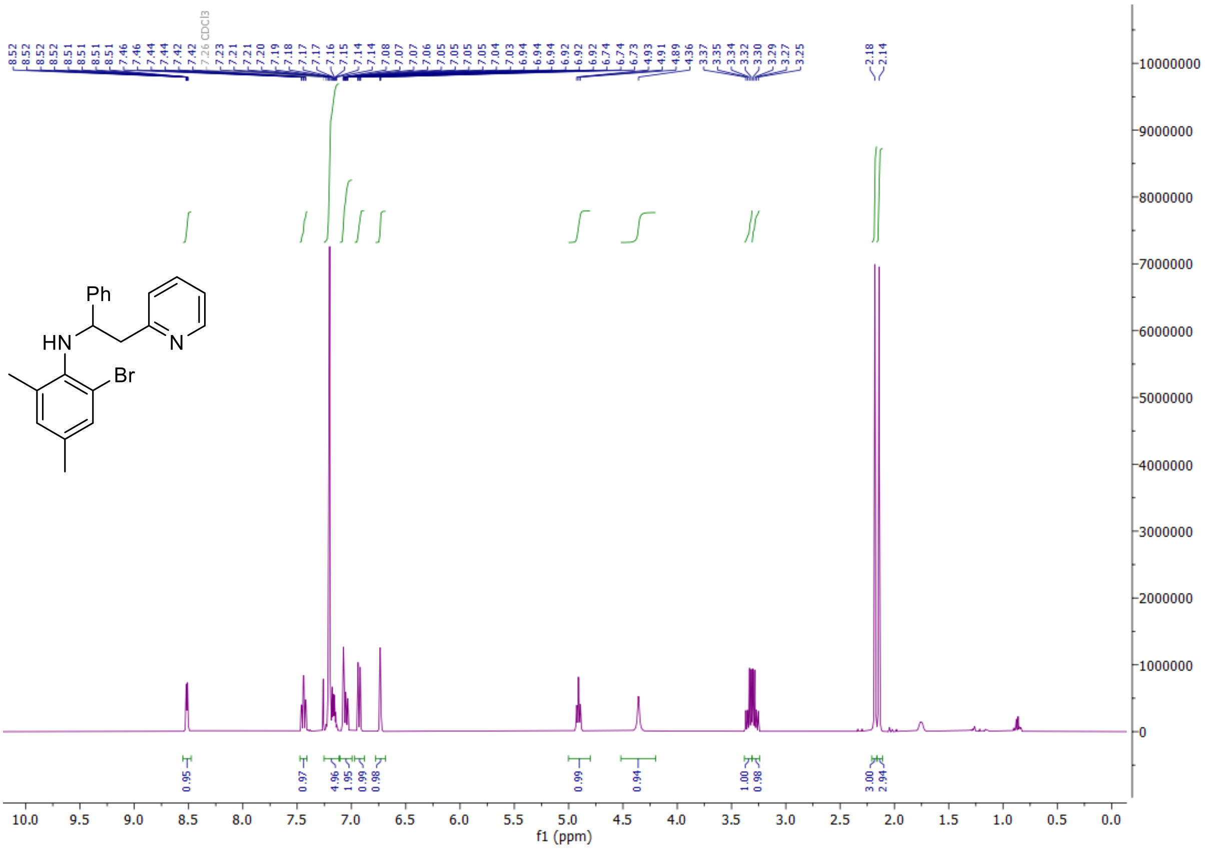

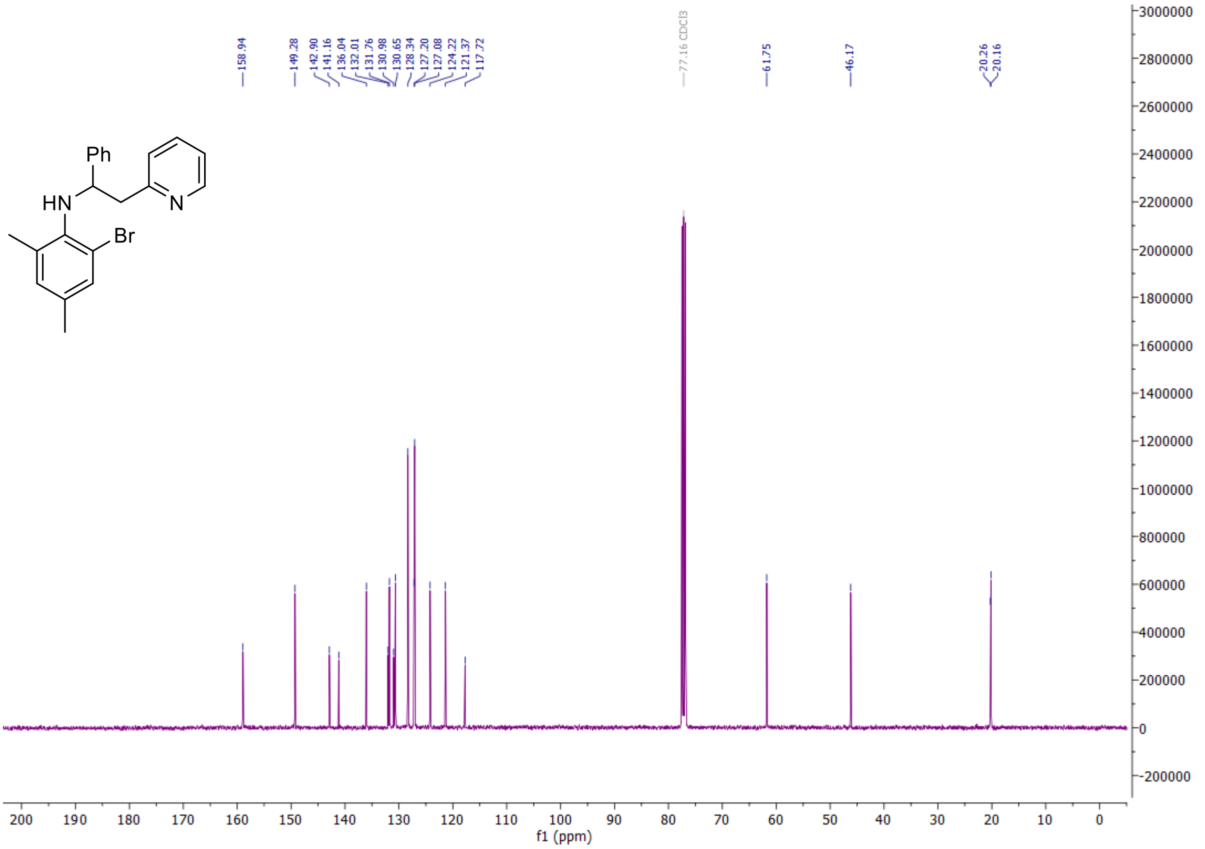

N-(3,5-Dimethyl-[1,1'-biphenyl]-2-yl)-1-phenylmethanimine (4g)

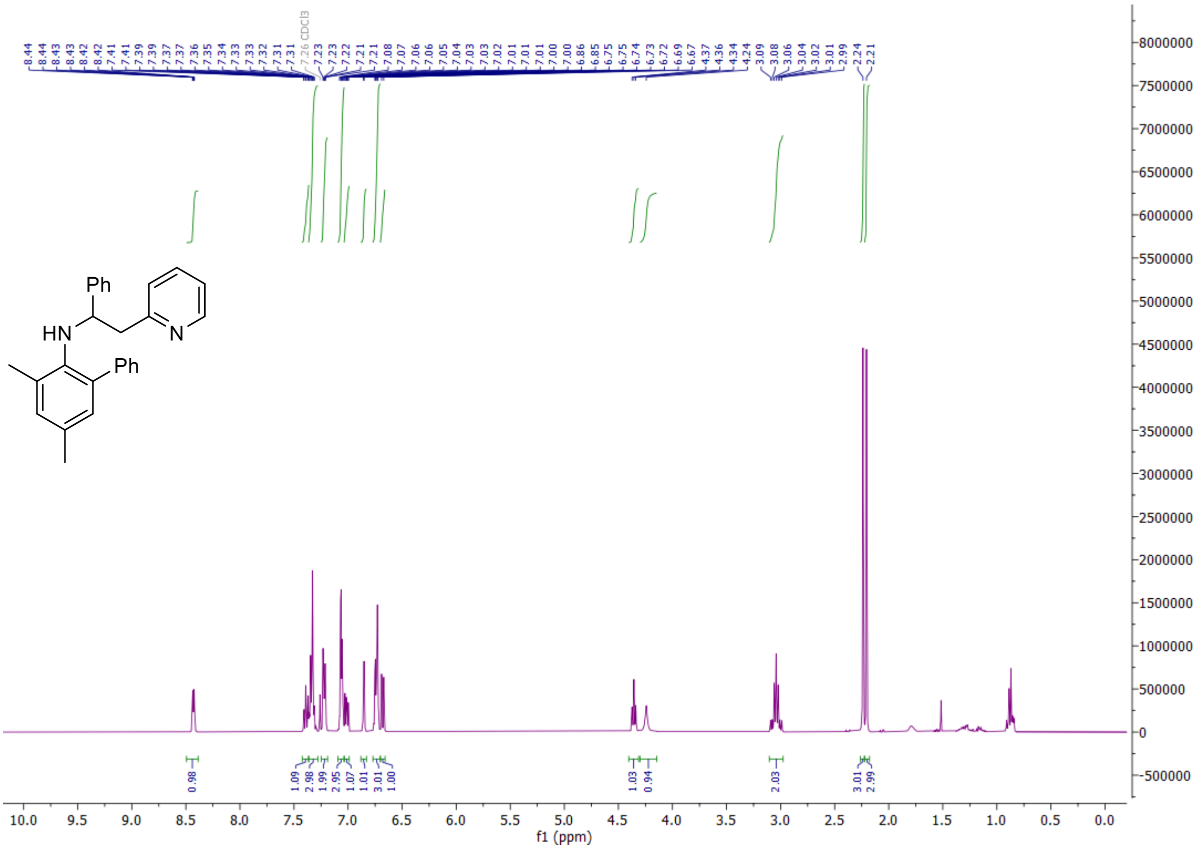

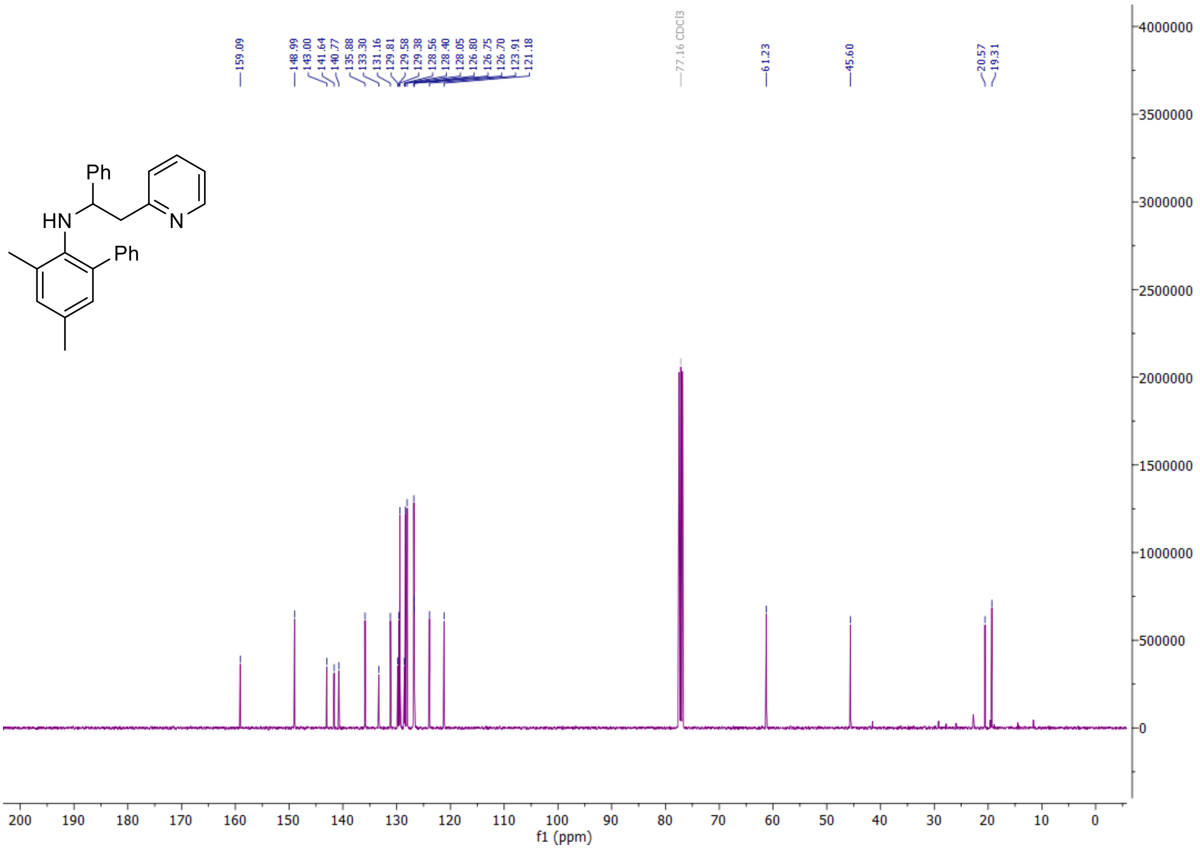

N-(2-Iodo-4,6-dimethylphenyl)-*N*-(1-phenyl-2-(pyridin-2-yl)ethyl)propionamide (6zf)

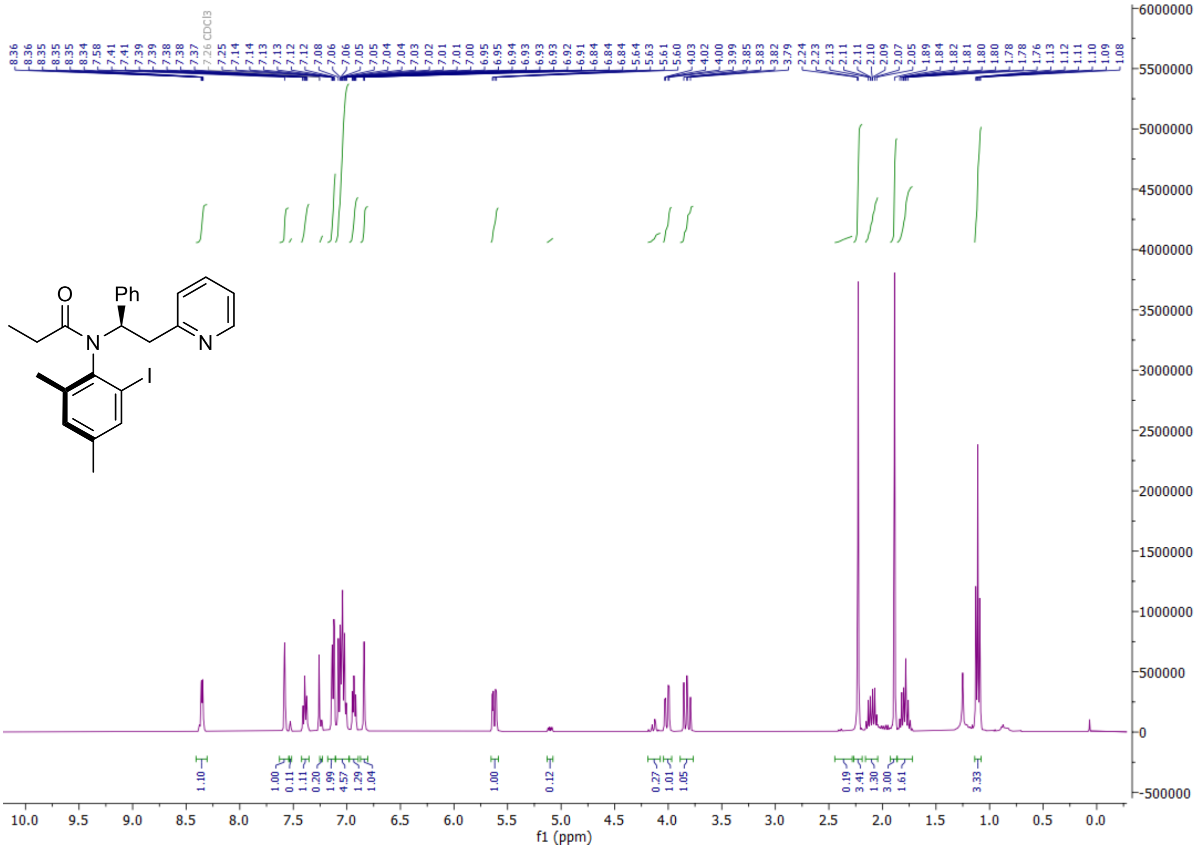

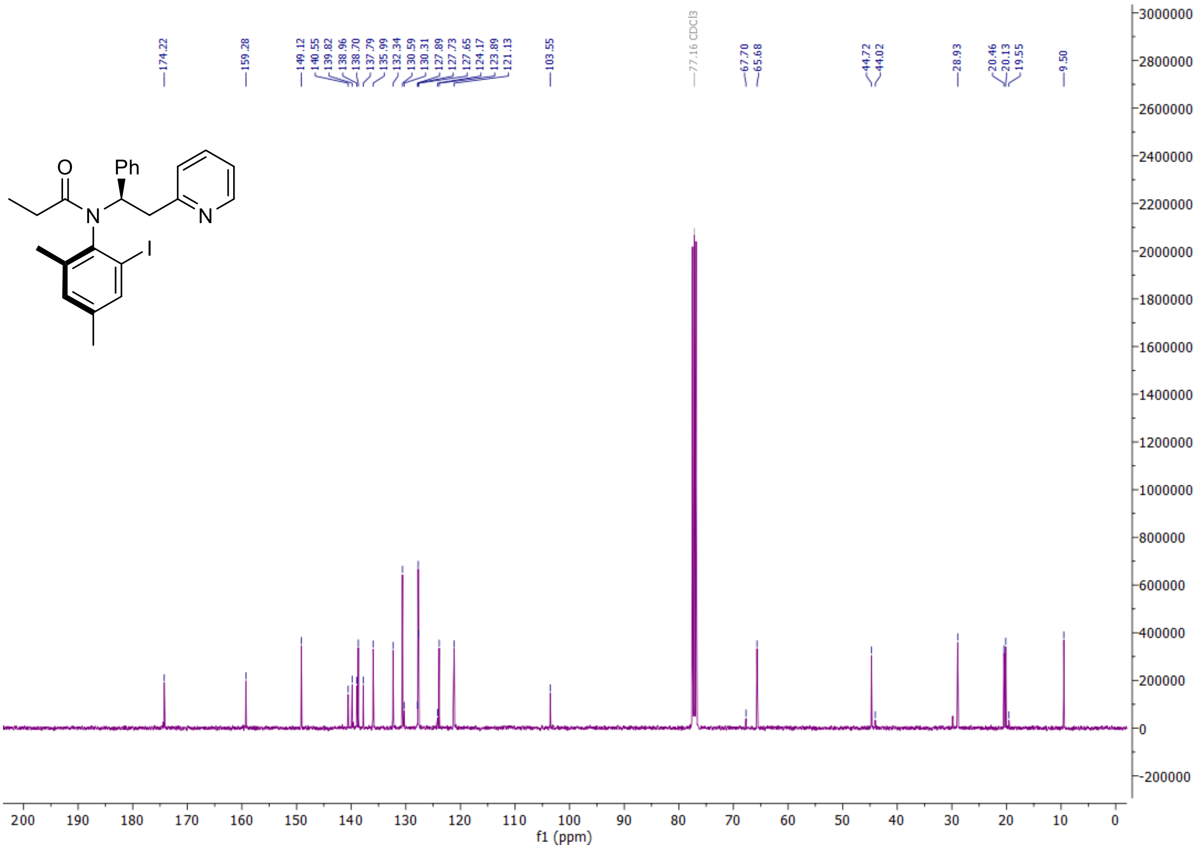

N-(2-Iodo-4,6-dimethylphenyl)-*N*-(1-phenyl-2-(pyridin-2-yl)ethyl)benzamide (6zg)

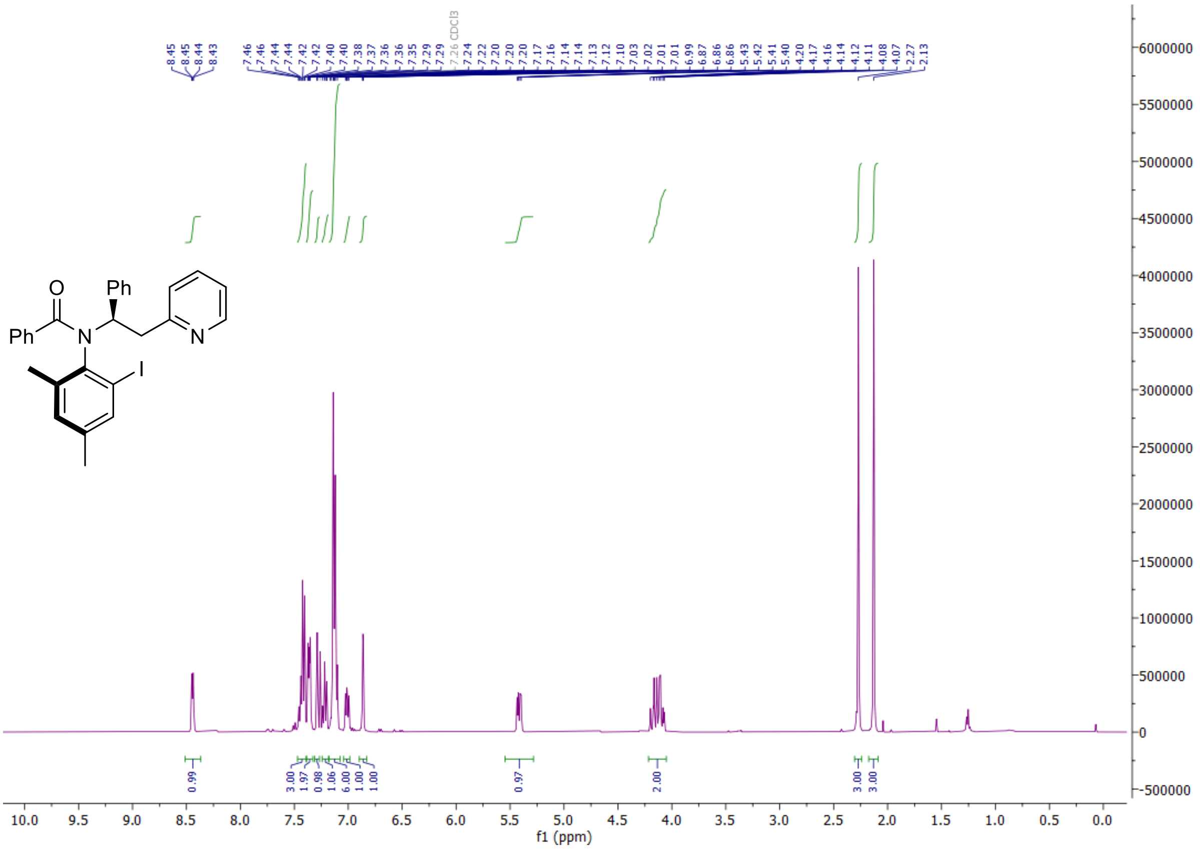

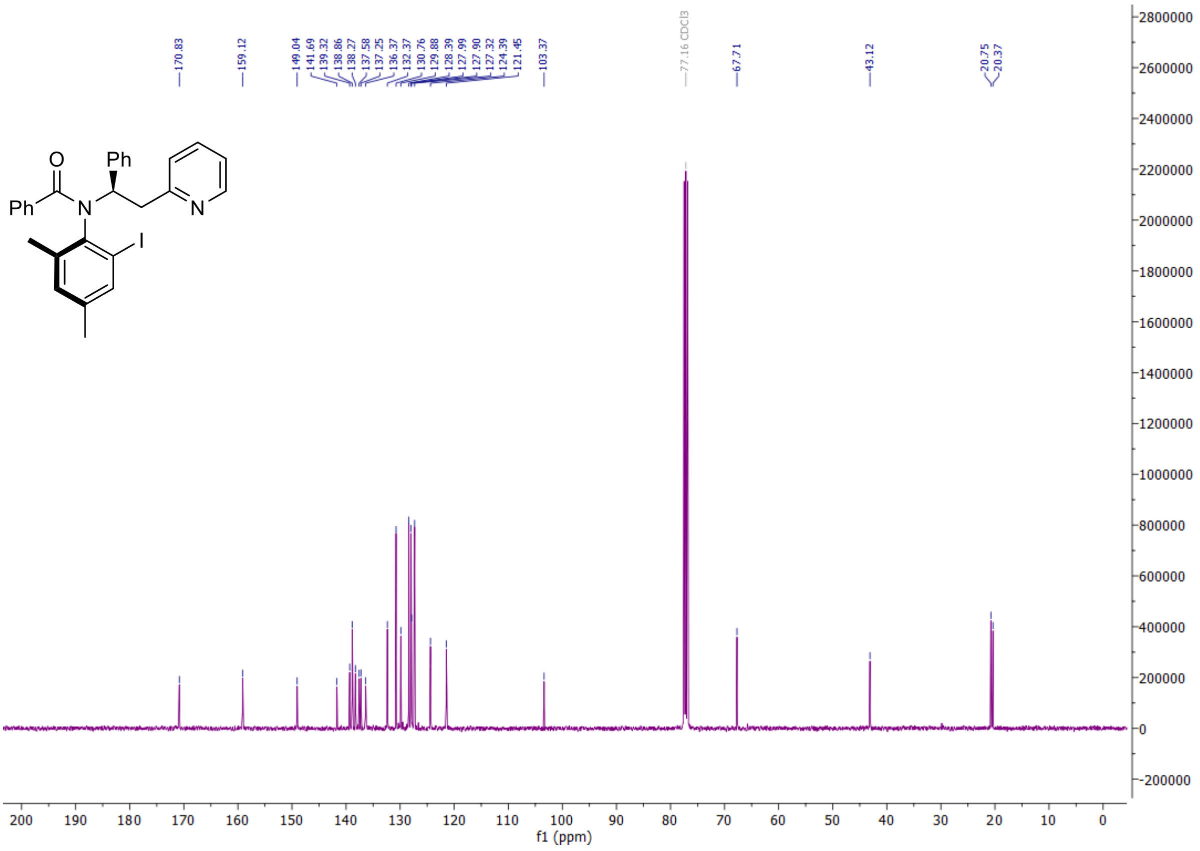

N-(2-Bromo-4,6-dimethylphenyl)-*N*-(1-phenyl-2-(pyridin-2-yl)ethyl)propionamide (6zh)

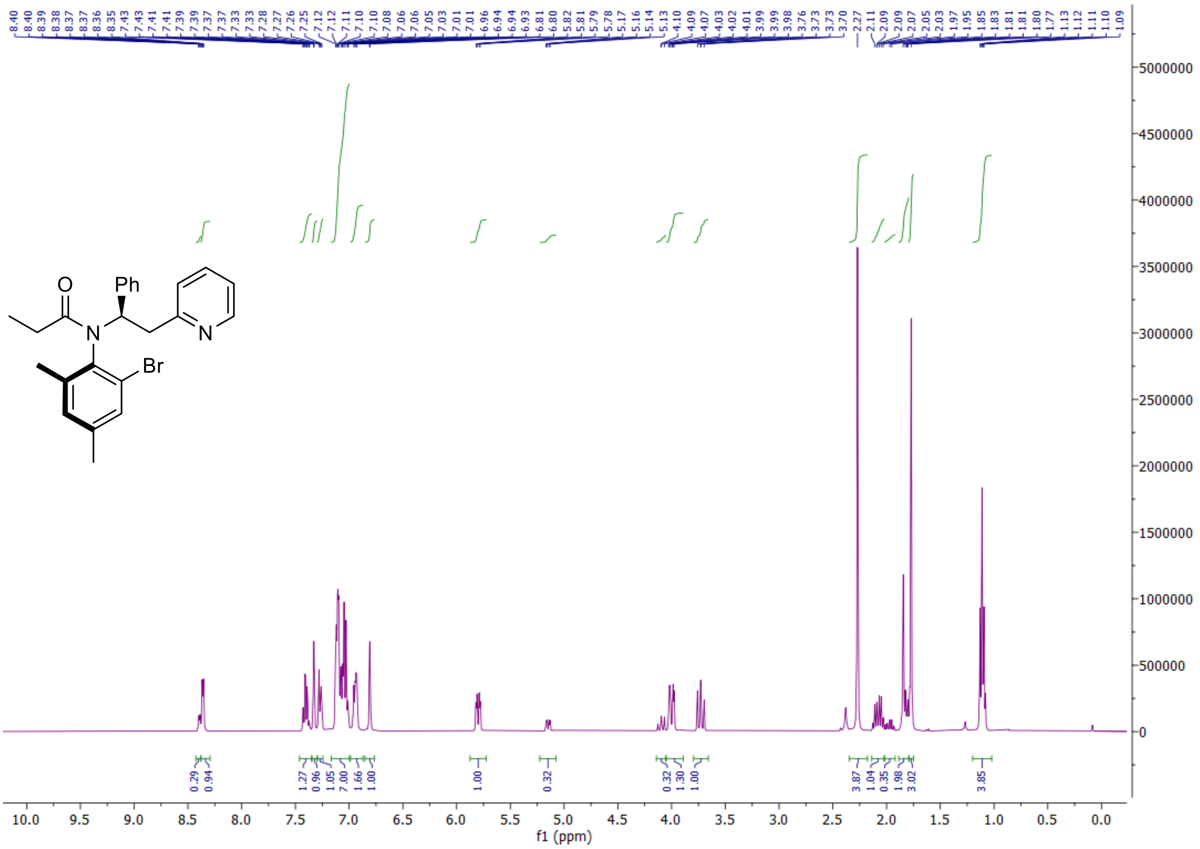

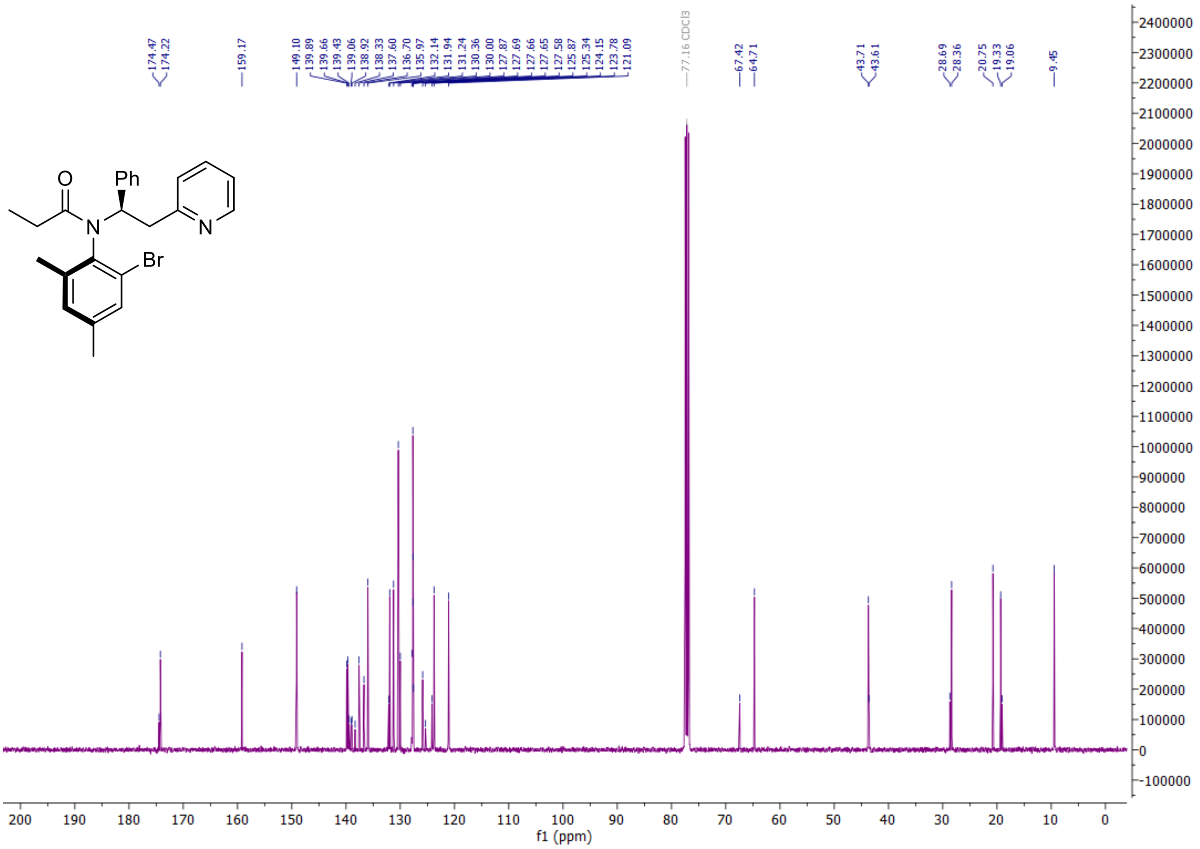

N-(3,5-Dimethyl-[1,1'-biphenyl]-2-yl)-*N*-(1-phenyl-2-(pyridin-2-yl)ethyl)propionamide (6zi)

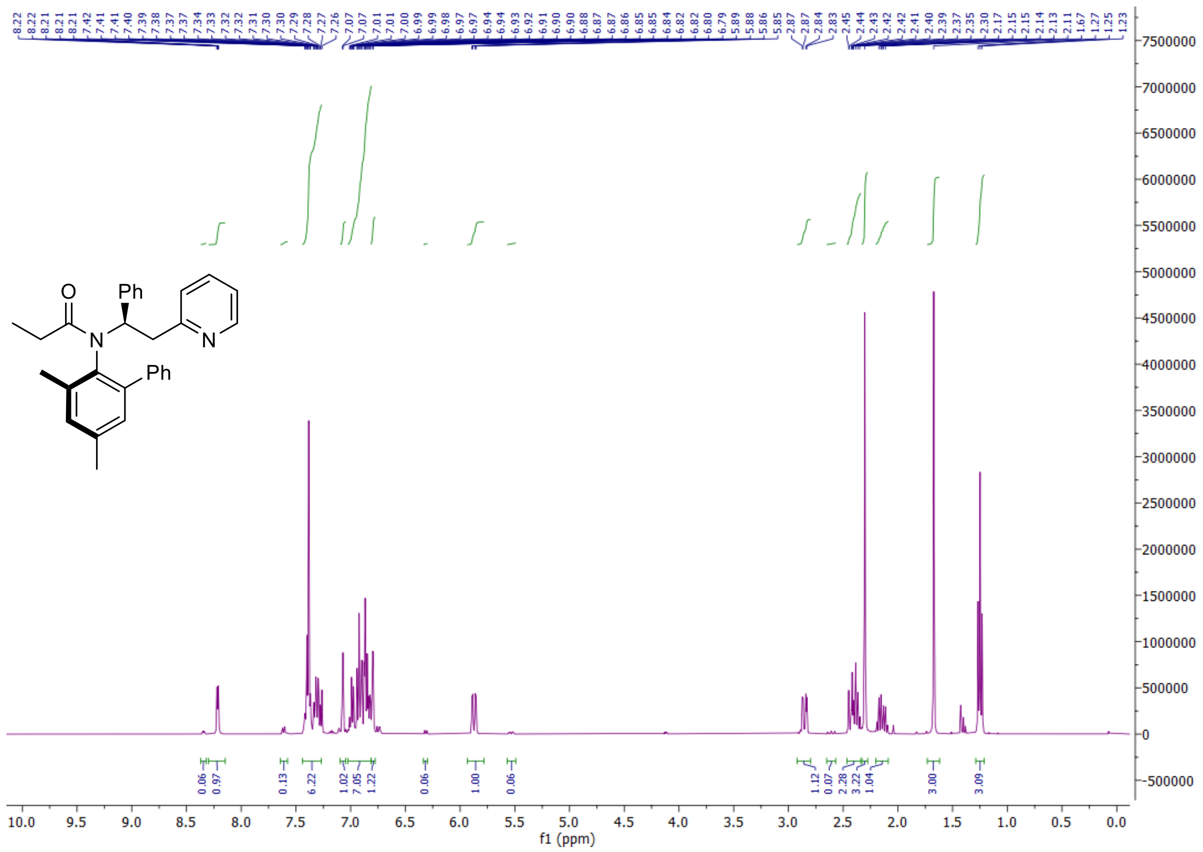

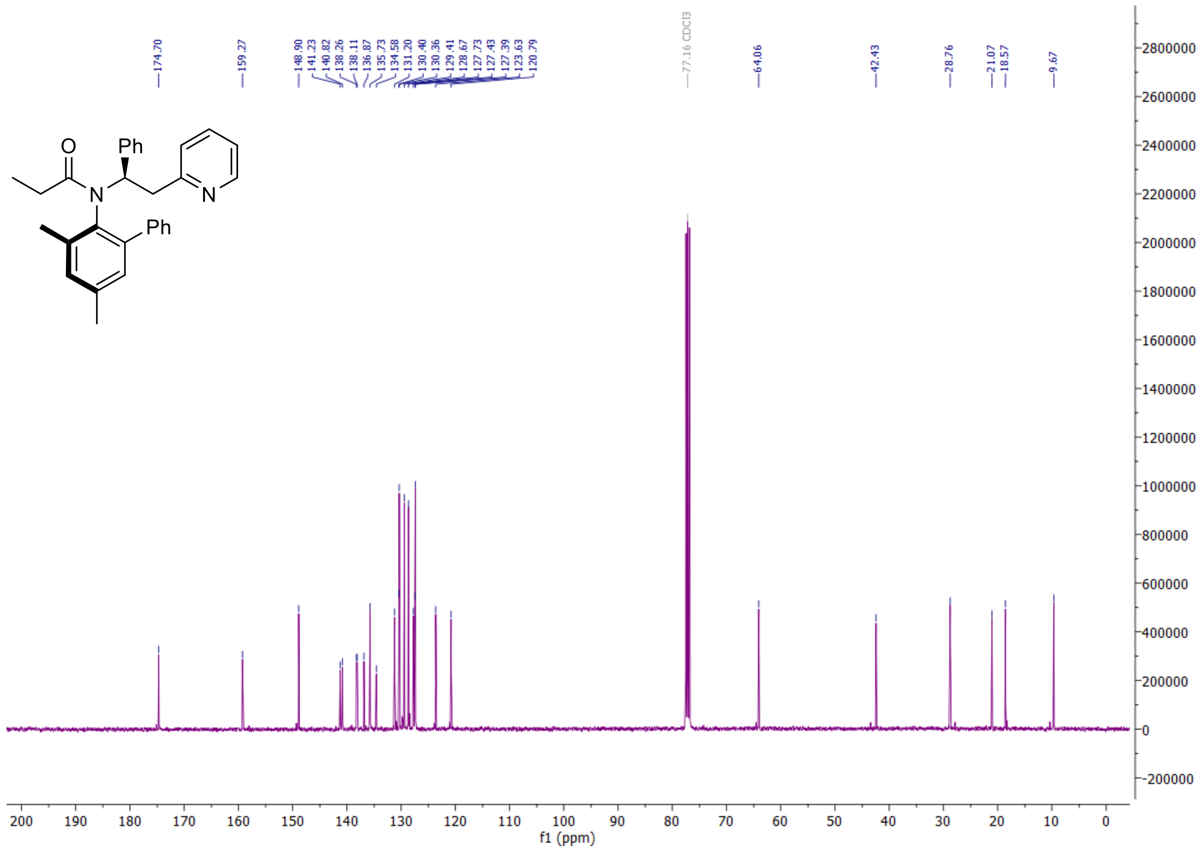

N-(3,5-Dimethyl-[1,1'-biphenyl]-2-yl)-*N*-(1-phenyl-2-(pyridin-2-yl)ethyl)propionamide (11zi)

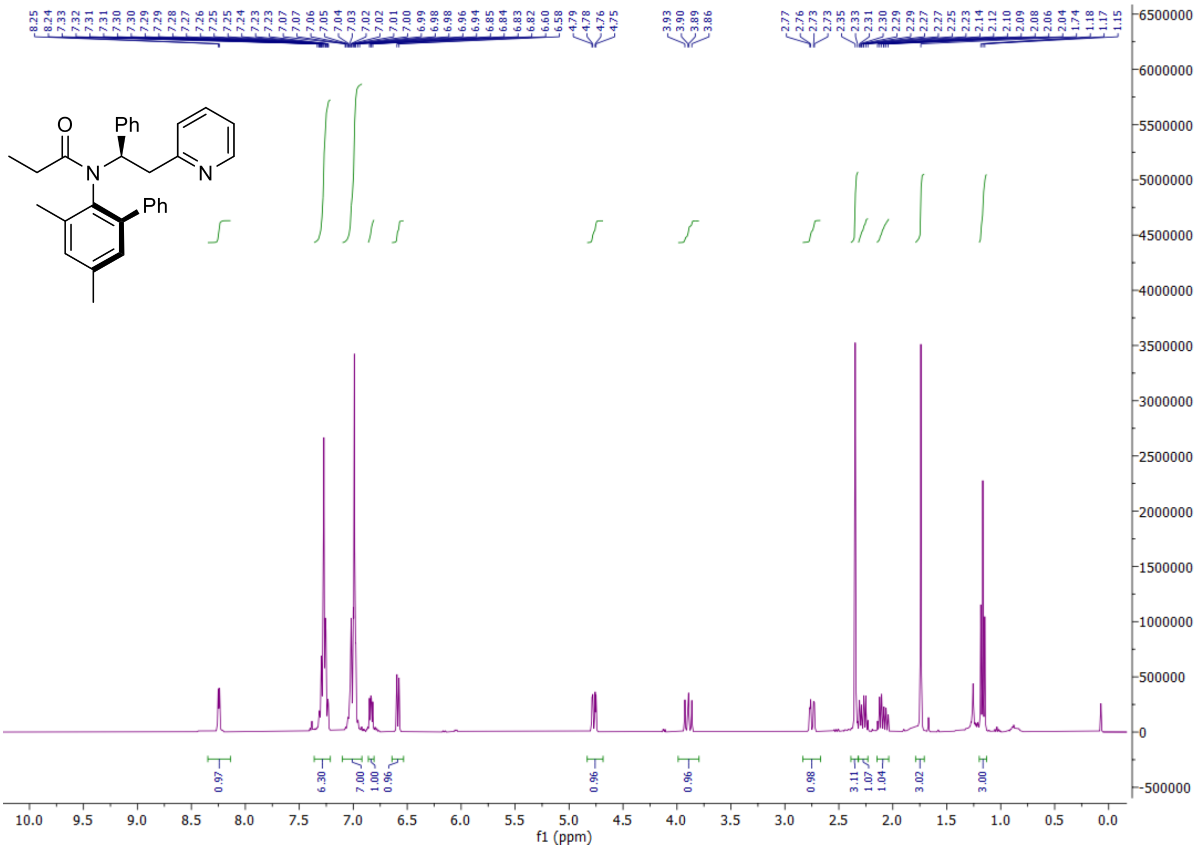

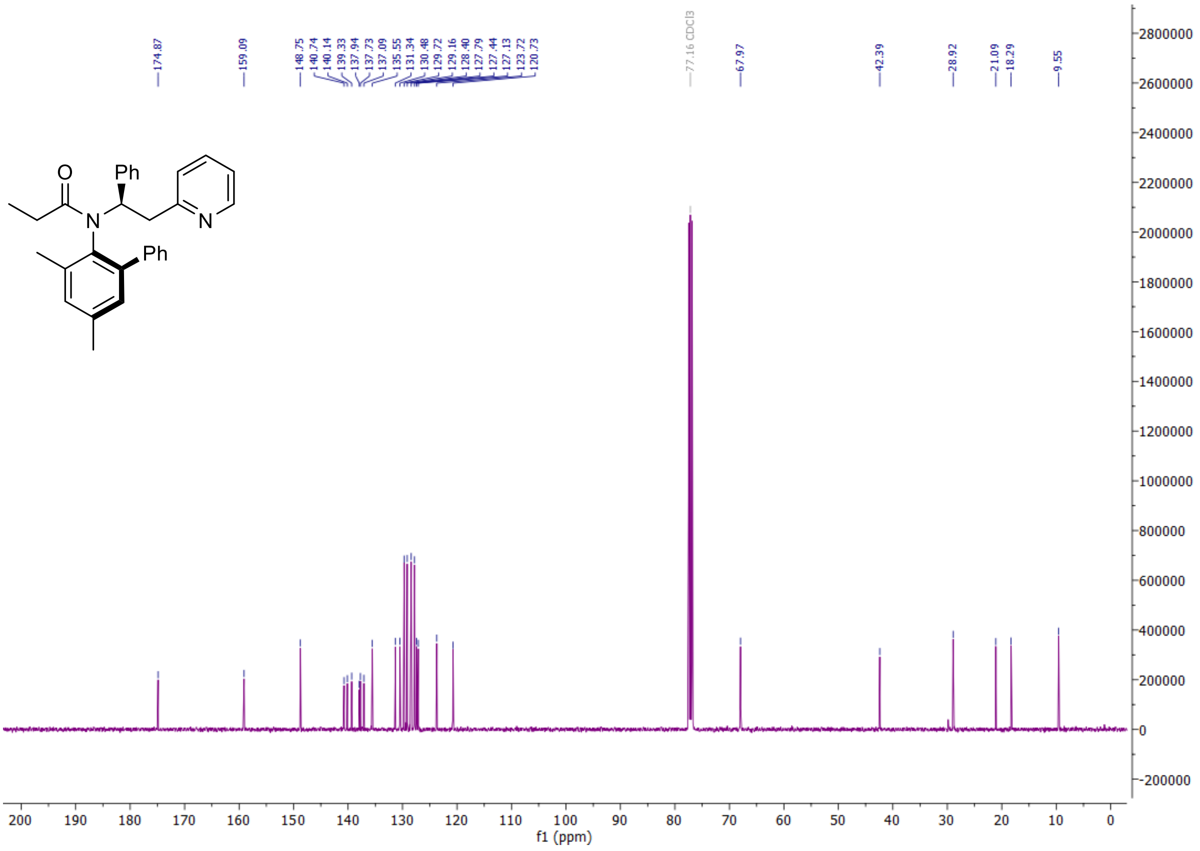

N-(2-Iodo-4,6-dimethylphenyl)-N-(1-phenyl-2-(pyridin-2-yl)ethyl)acrylamide (6zj)

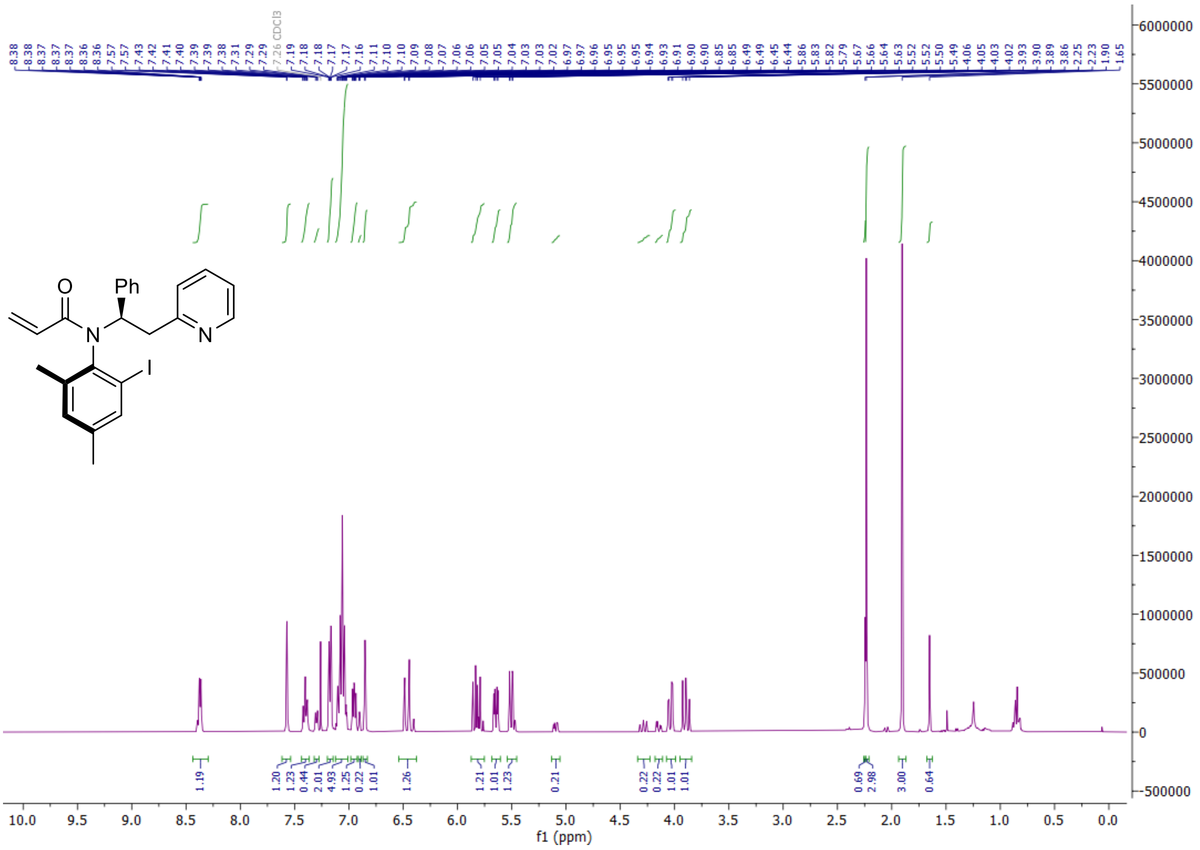

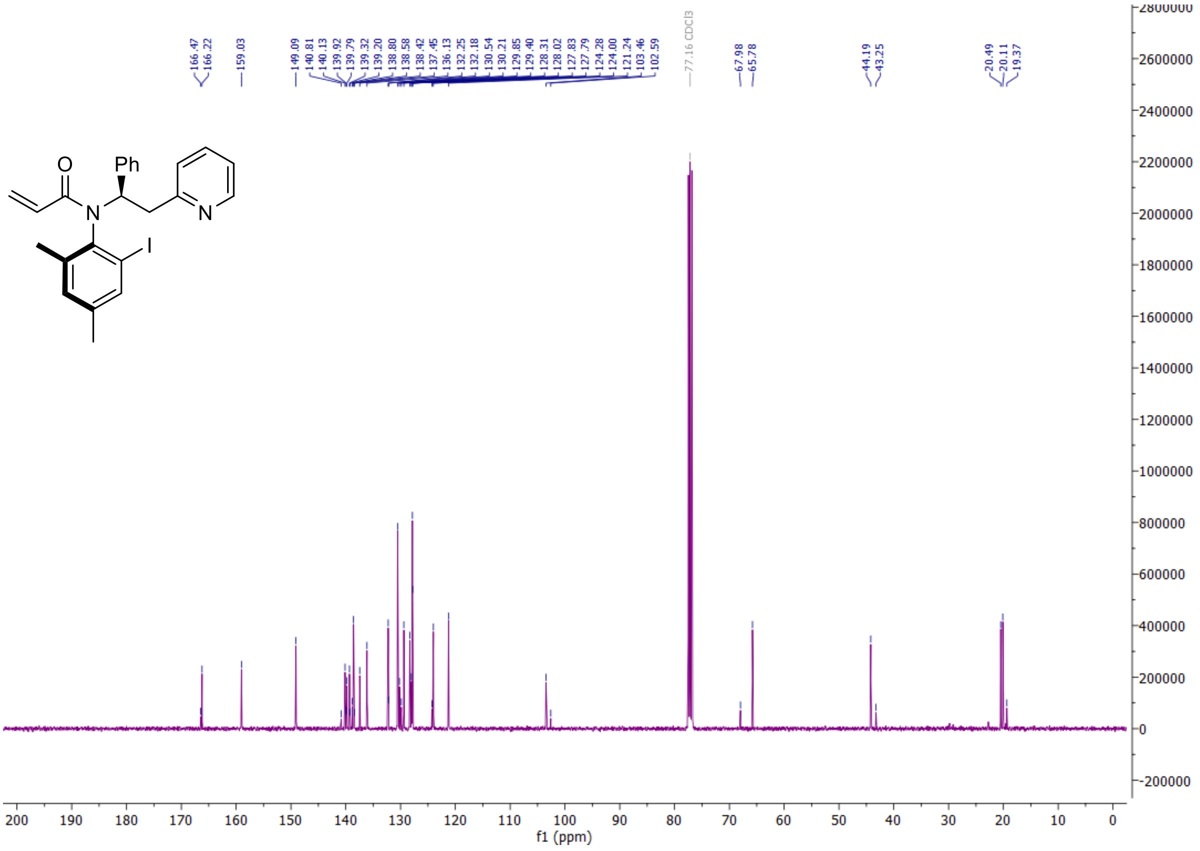

_N_-(2-Iodo-4,6-dimethylphenyl)-4-methoxy-_N_-(1-phenyl-2-(pyridin-2-yl)ethyl)benzamide (6zk)

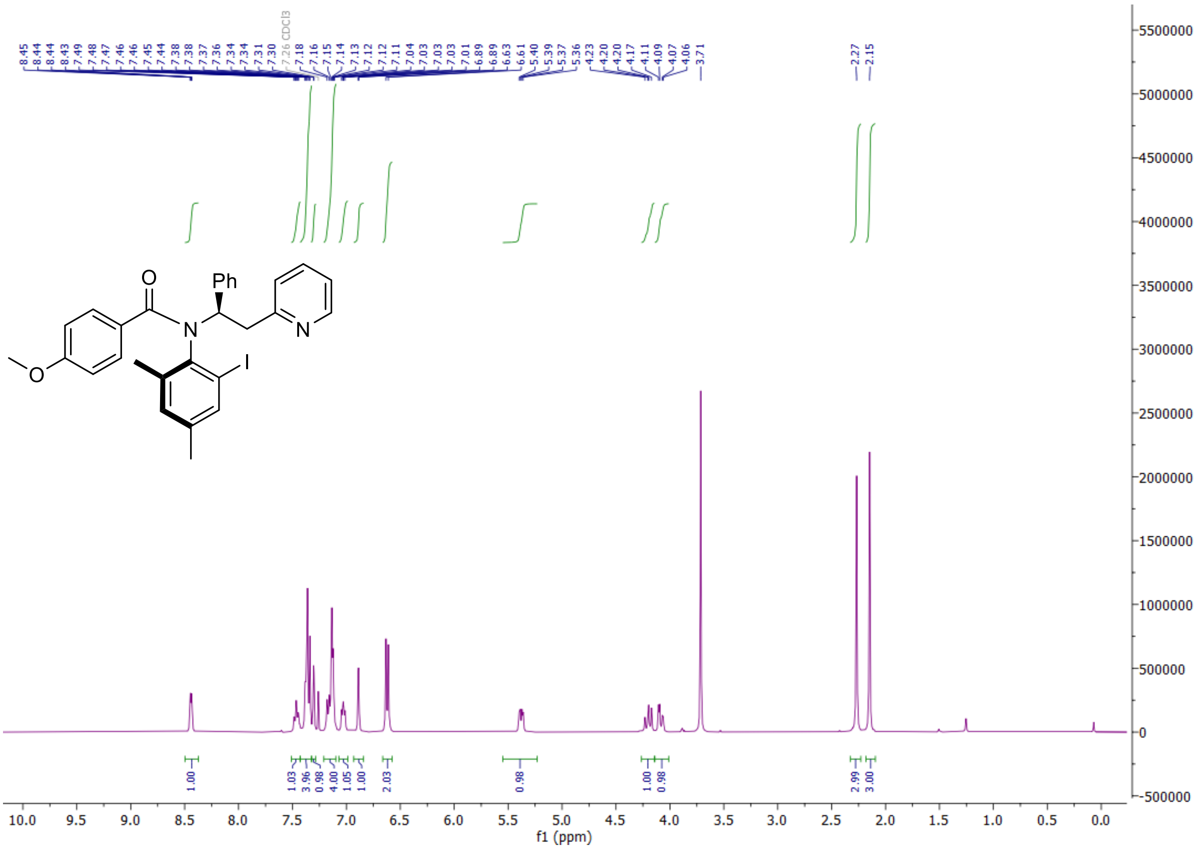

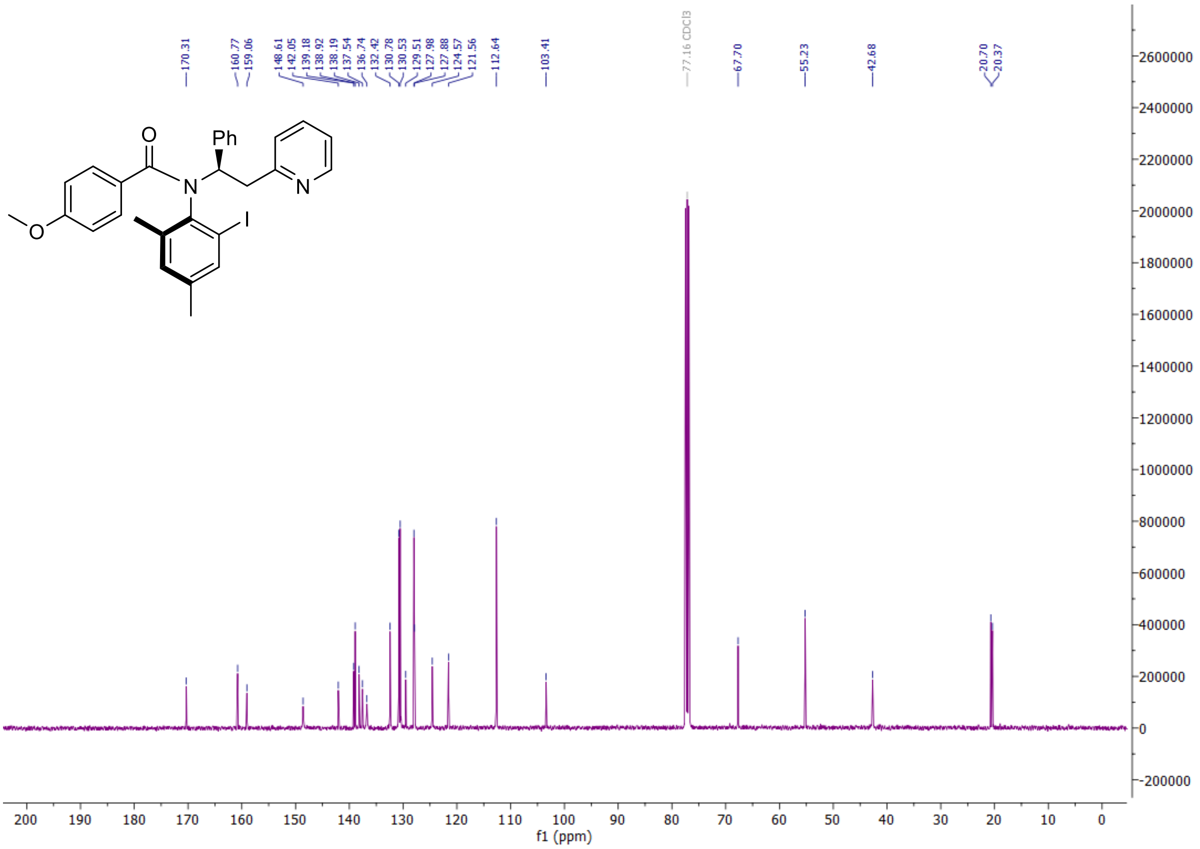

N-(2-Iodo-4,6-dimethylphenyl)-N-(1-phenyl-2-(pyridin-2-yl)ethyl)furan-2-carboxamide (6zl)

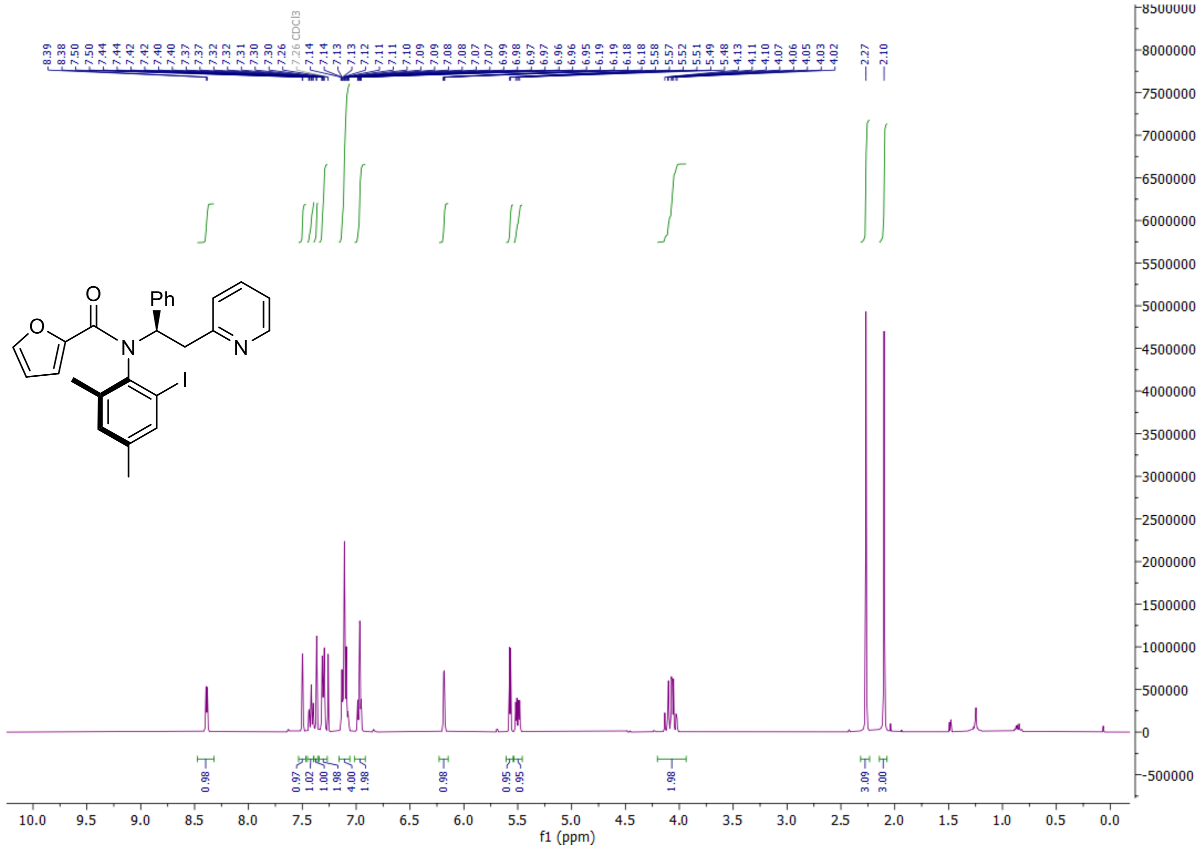

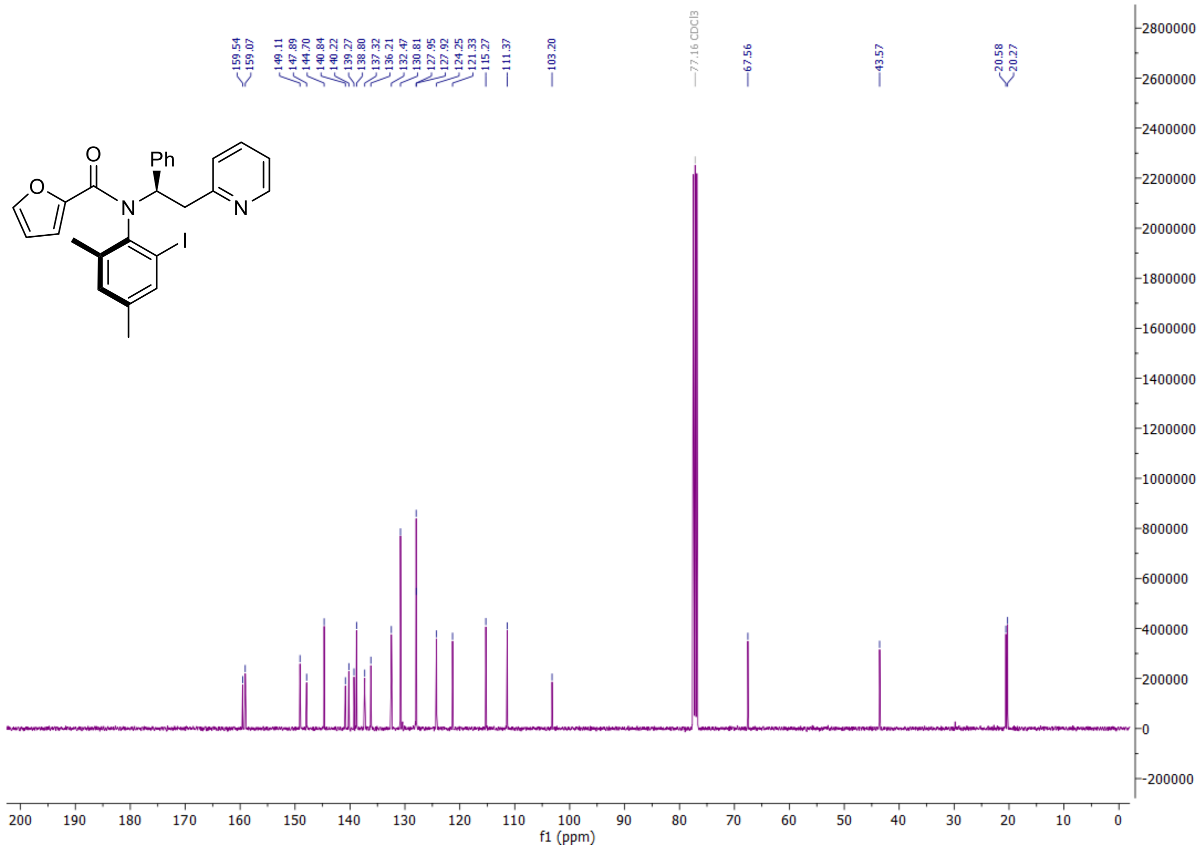

3-((2-(*tert*-Butyl)phenyl)amino)butan-1-ol (S10)

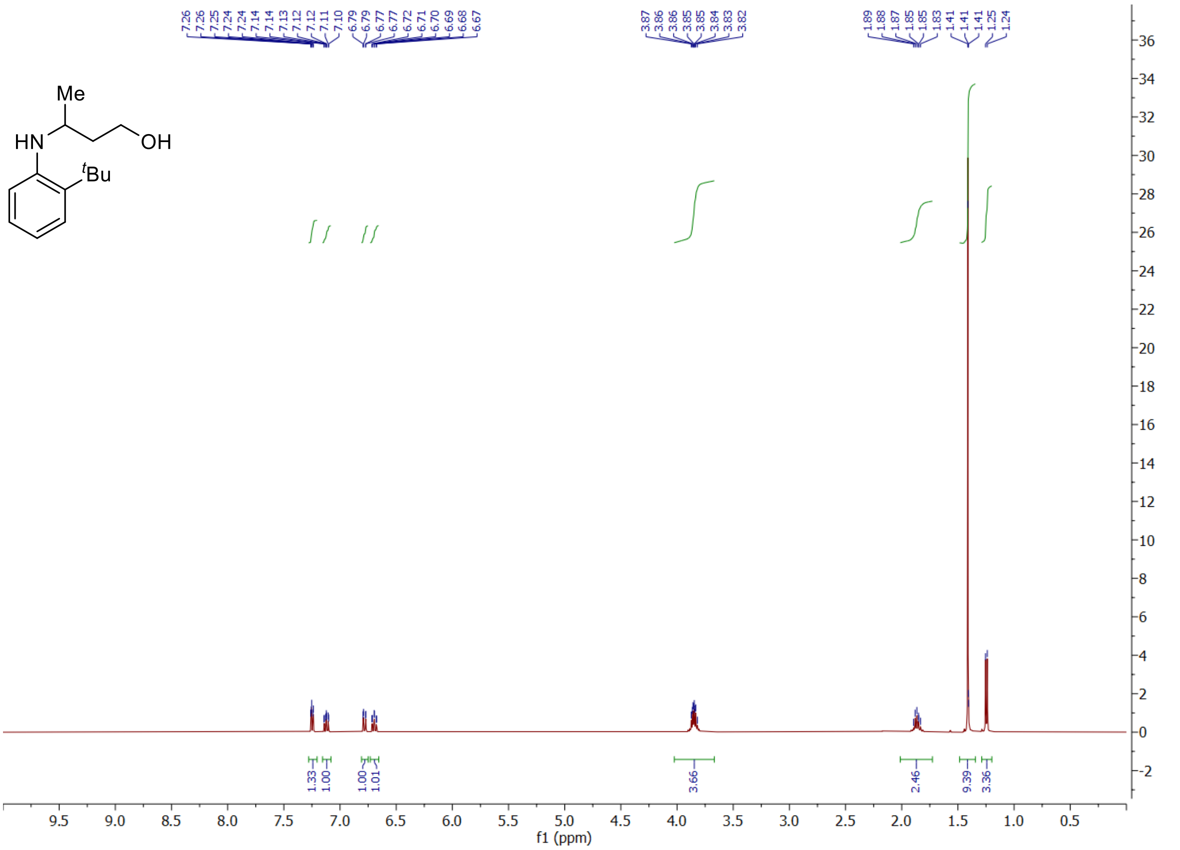

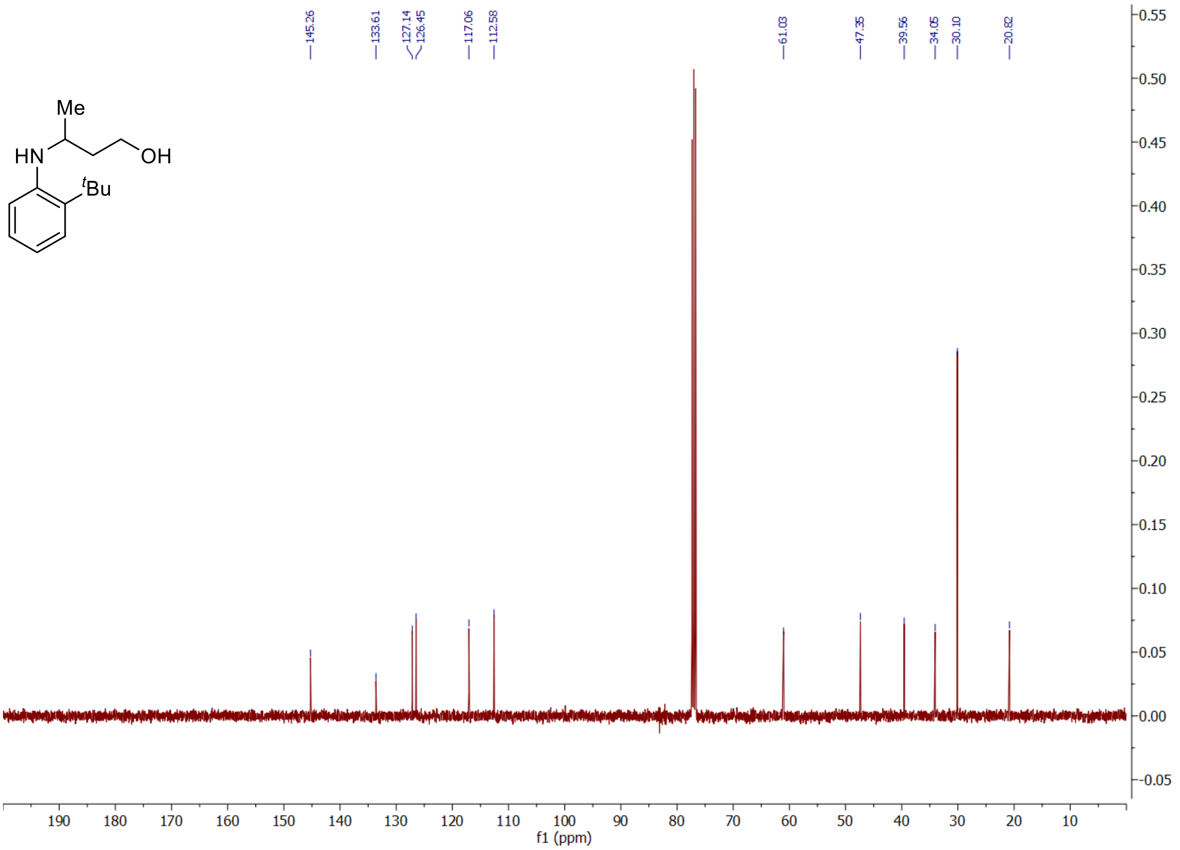

_N_-(4-Bromobutan-2-yl)-2-(_tert_-butyl)aniline (S11)

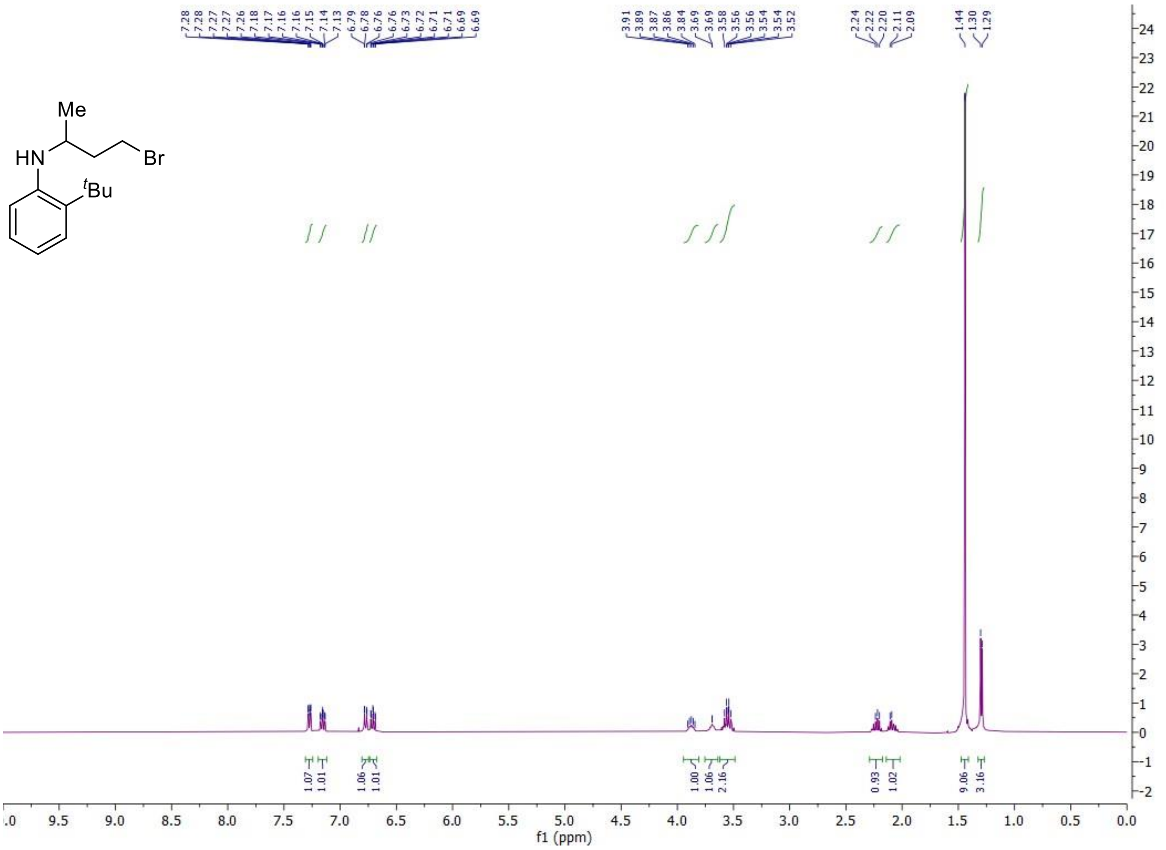

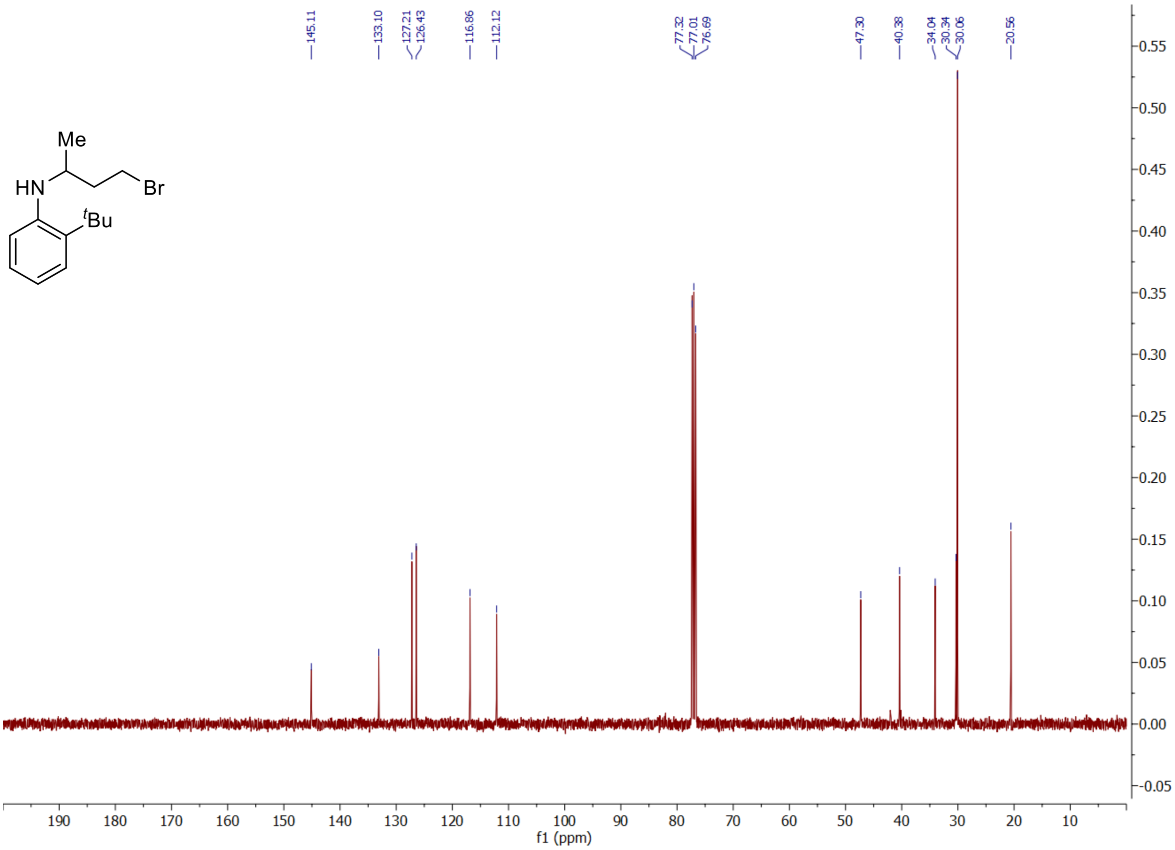

*N*¹-Benzyl-*N*¹-methyl-*N*³-phenylbutane-1,3-diamine (7a)

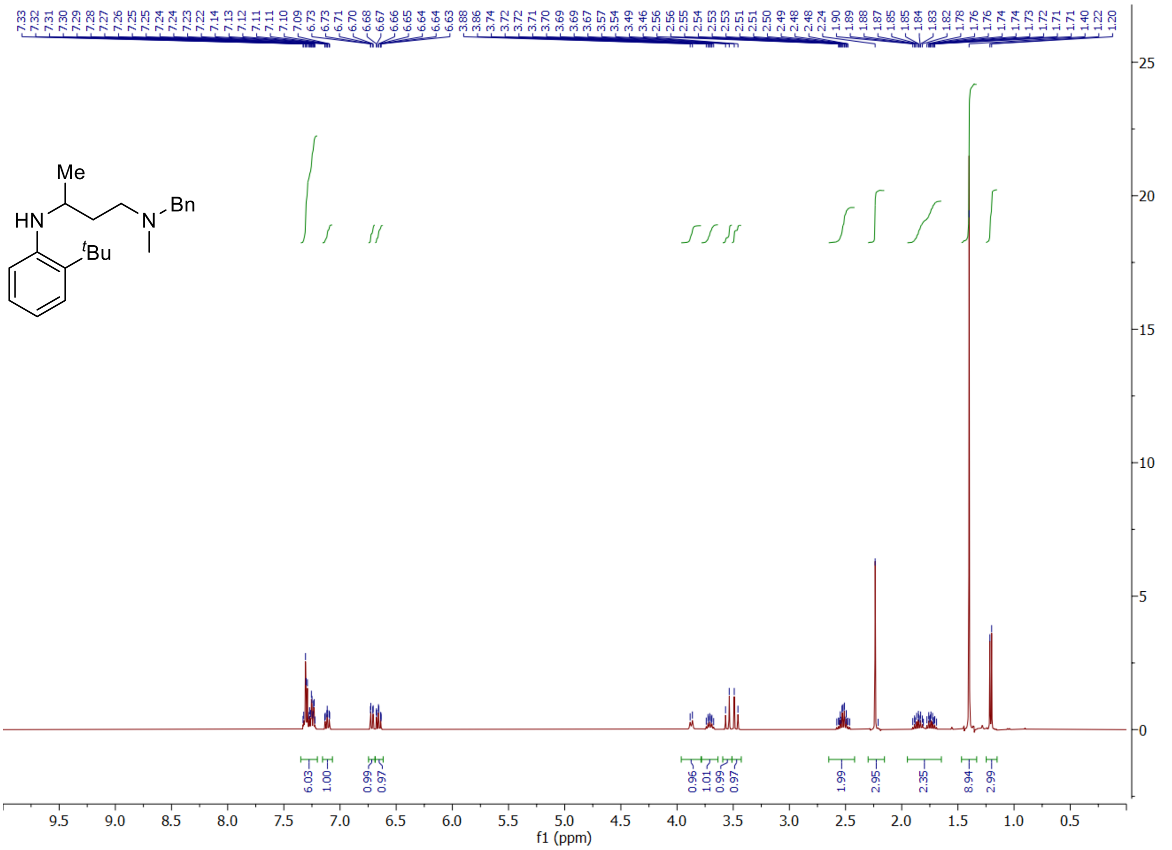

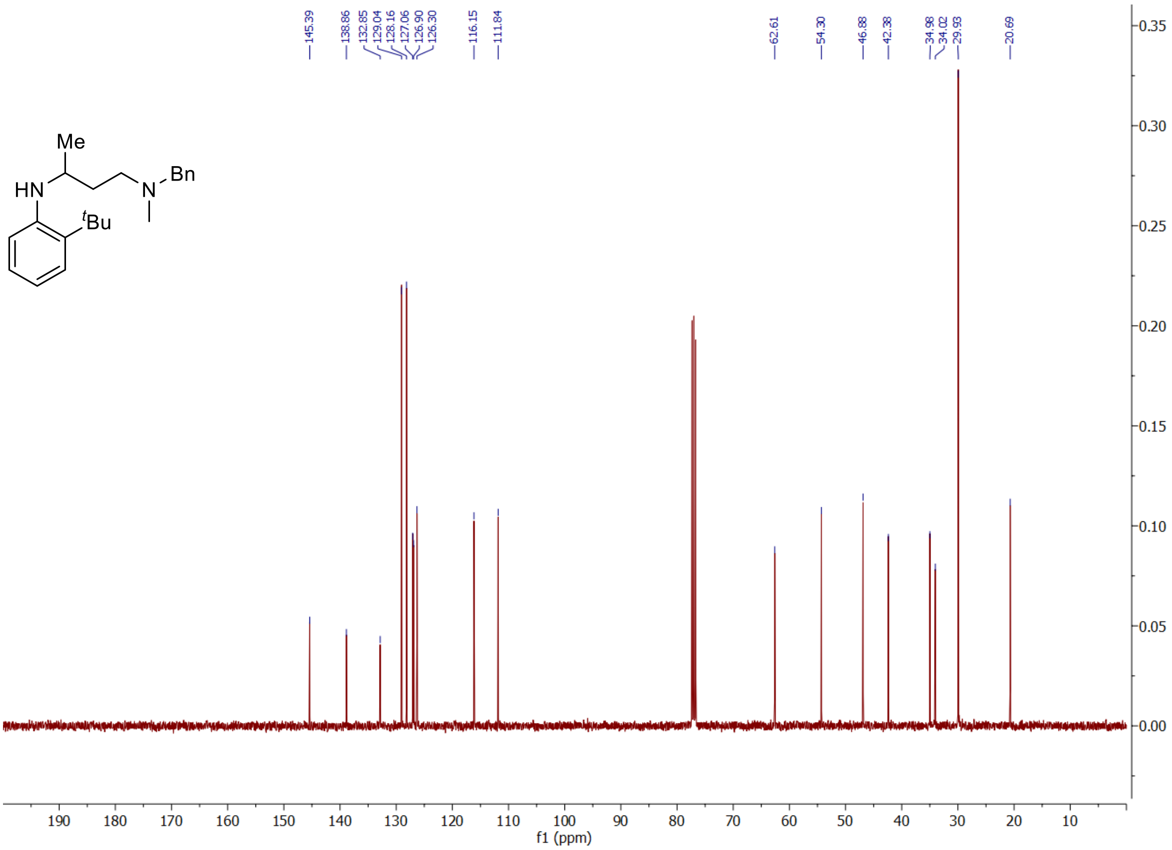

N^3-(2-(*tert*-Butyl)phenyl)-N^1,N^1-dipropylbutane-1,3-diamine (7b)

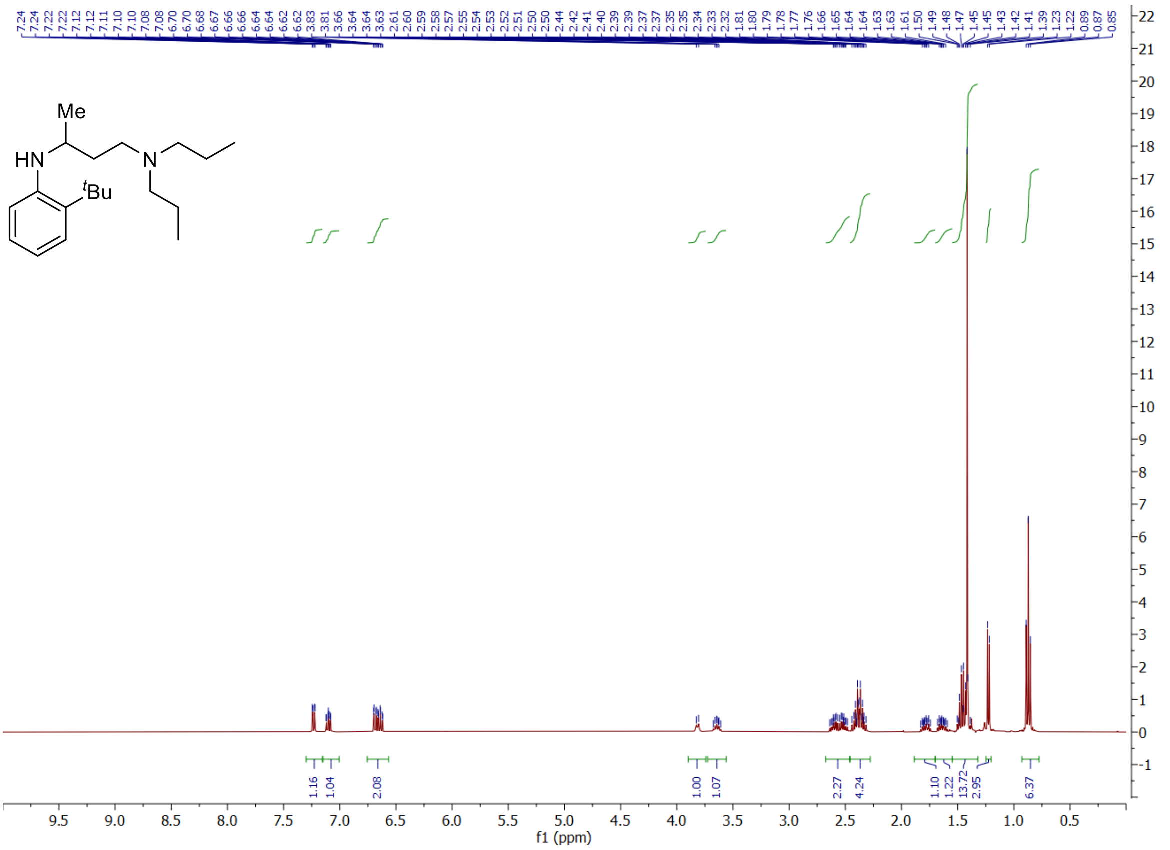

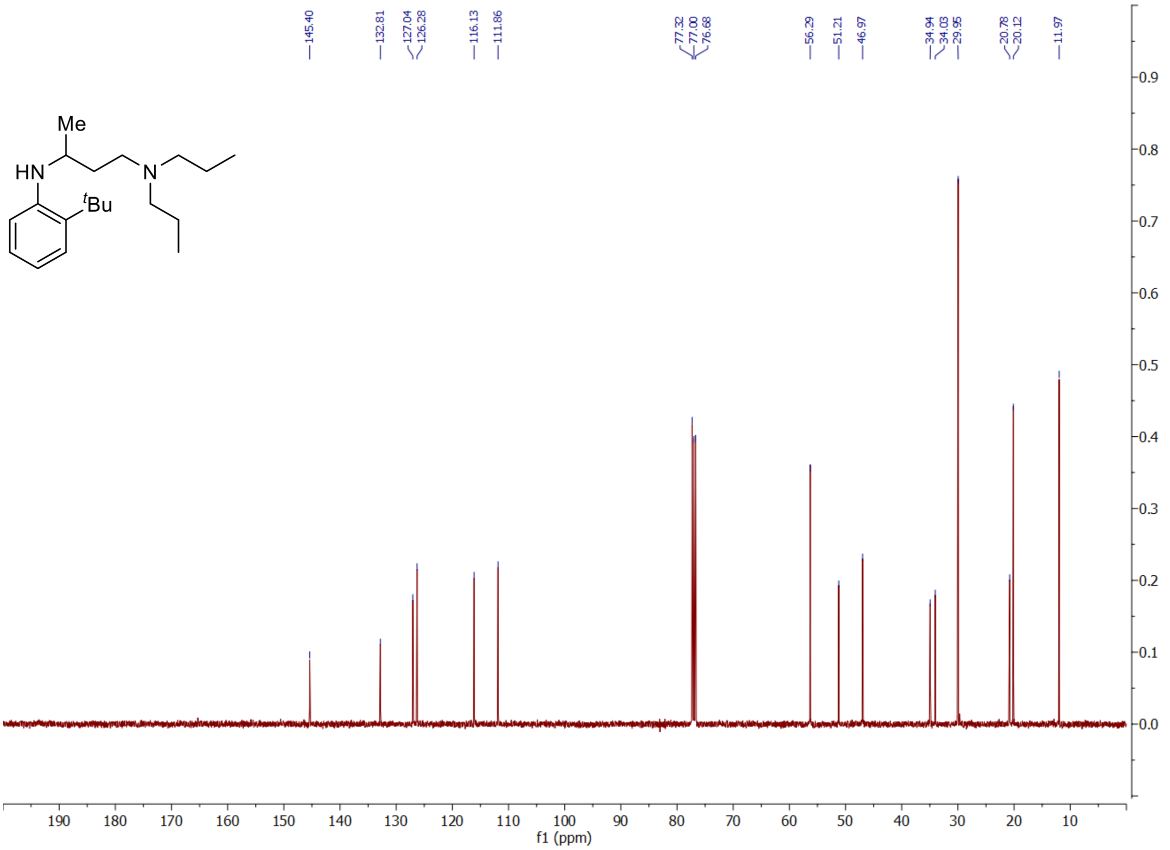

_N_-(4-(Benzyl(methyl)amino)butan-2-yl)-_N_-(2-(_tert_-butyl)phenyl)benzamide (9a)

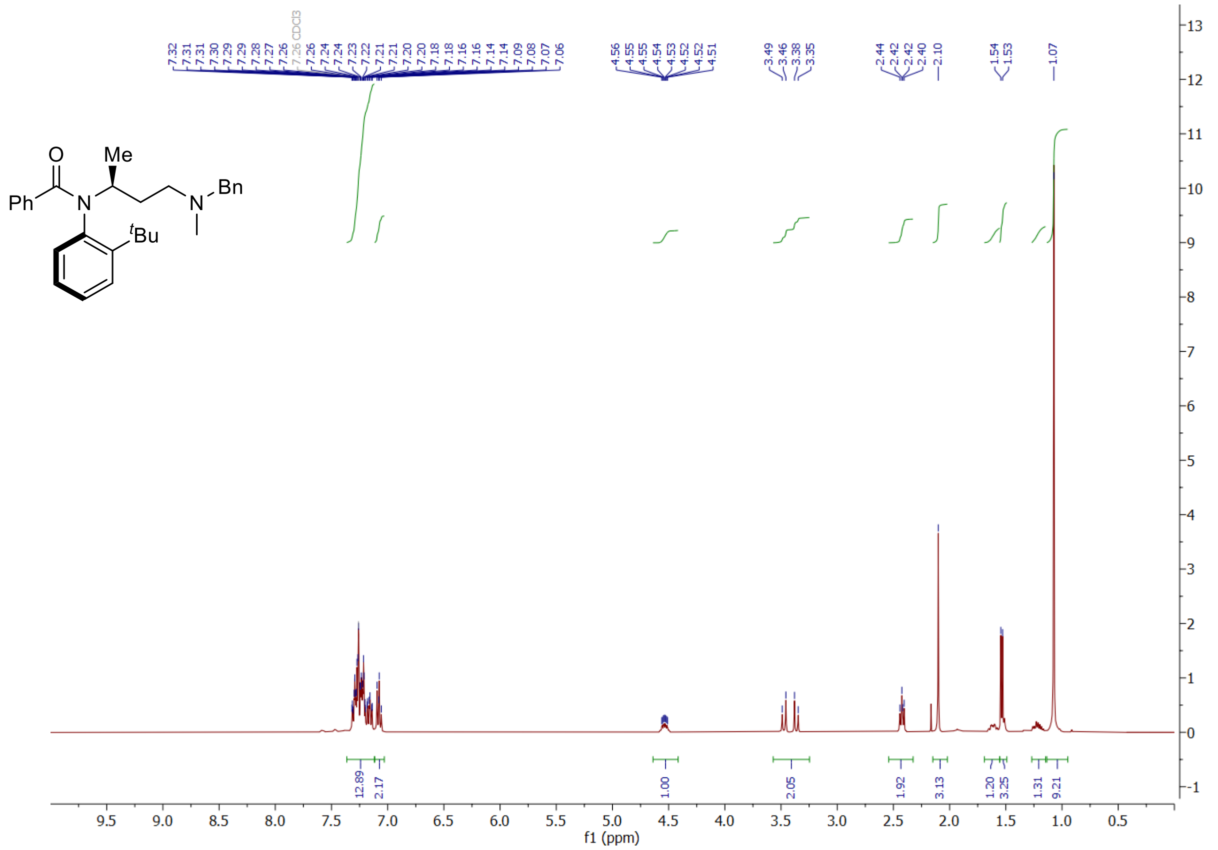

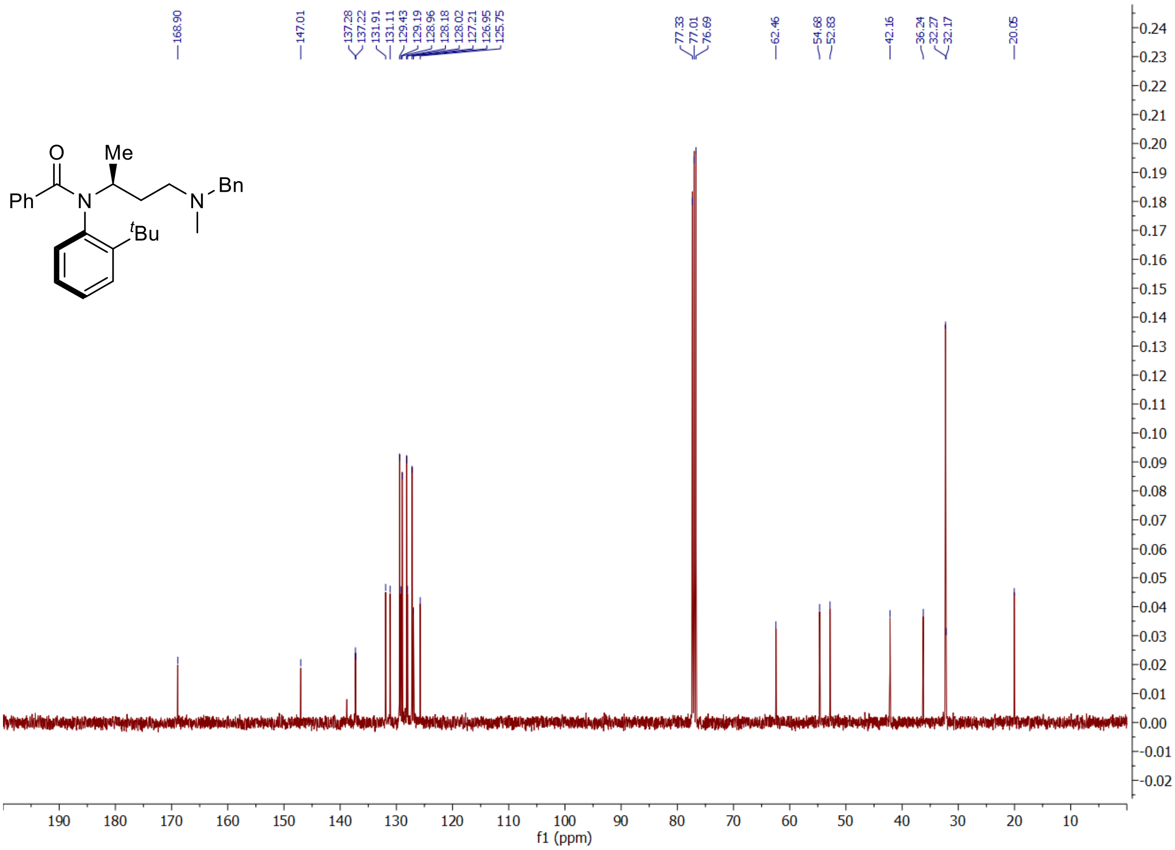

N-(4-(Benzyl(methyl)amino)butan-2-yl)-N-(2-(*tert*-butyl)phenyl)propionamide (9b)

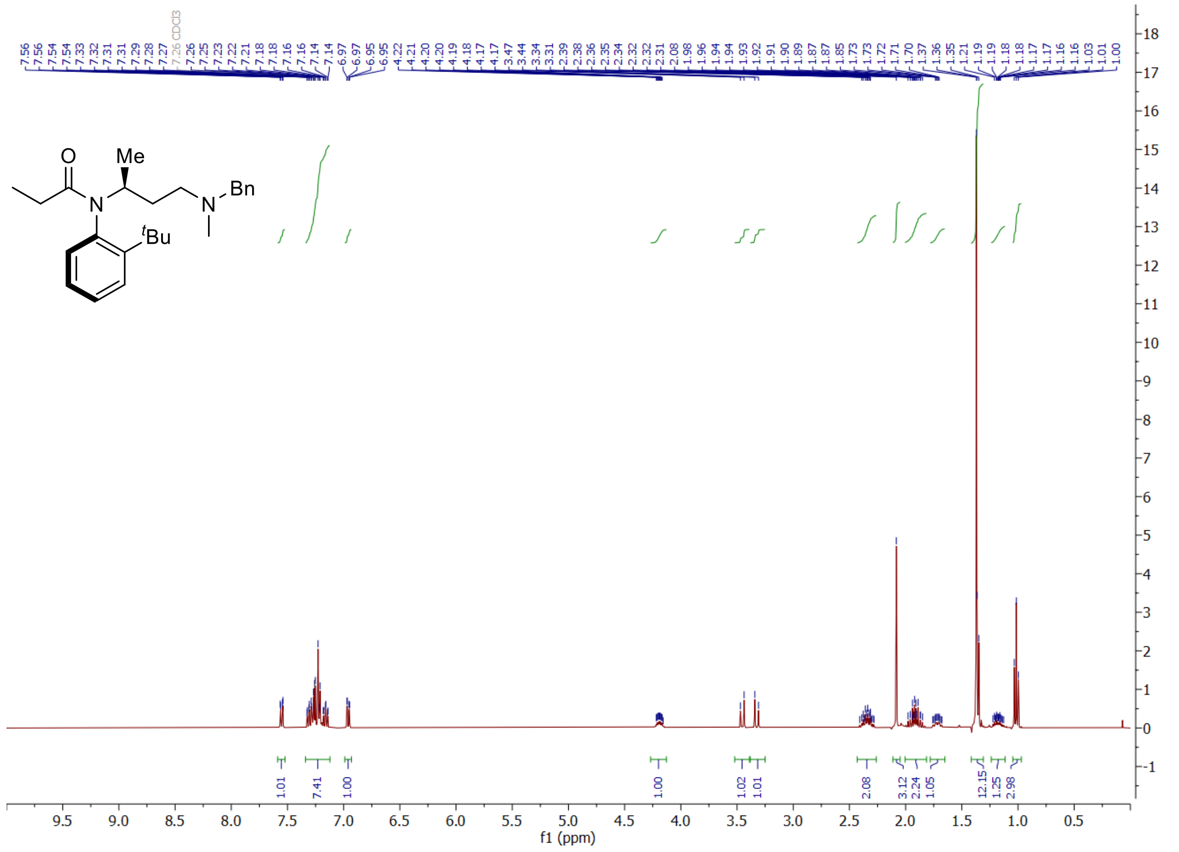

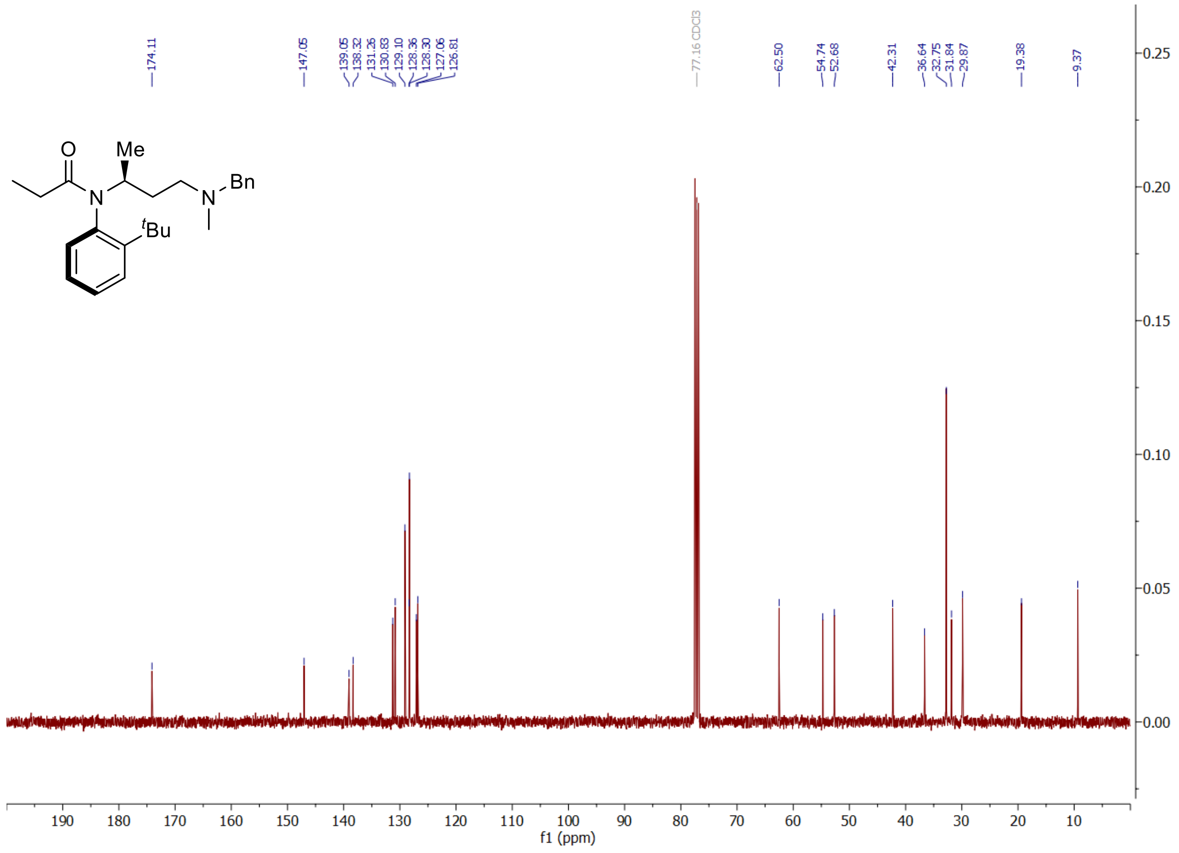

_N_-(4-(Benzyl(methyl)amino)butan-2-yl)-_N_-(2-(_tert_-butyl)phenyl)acrylamide (9c)

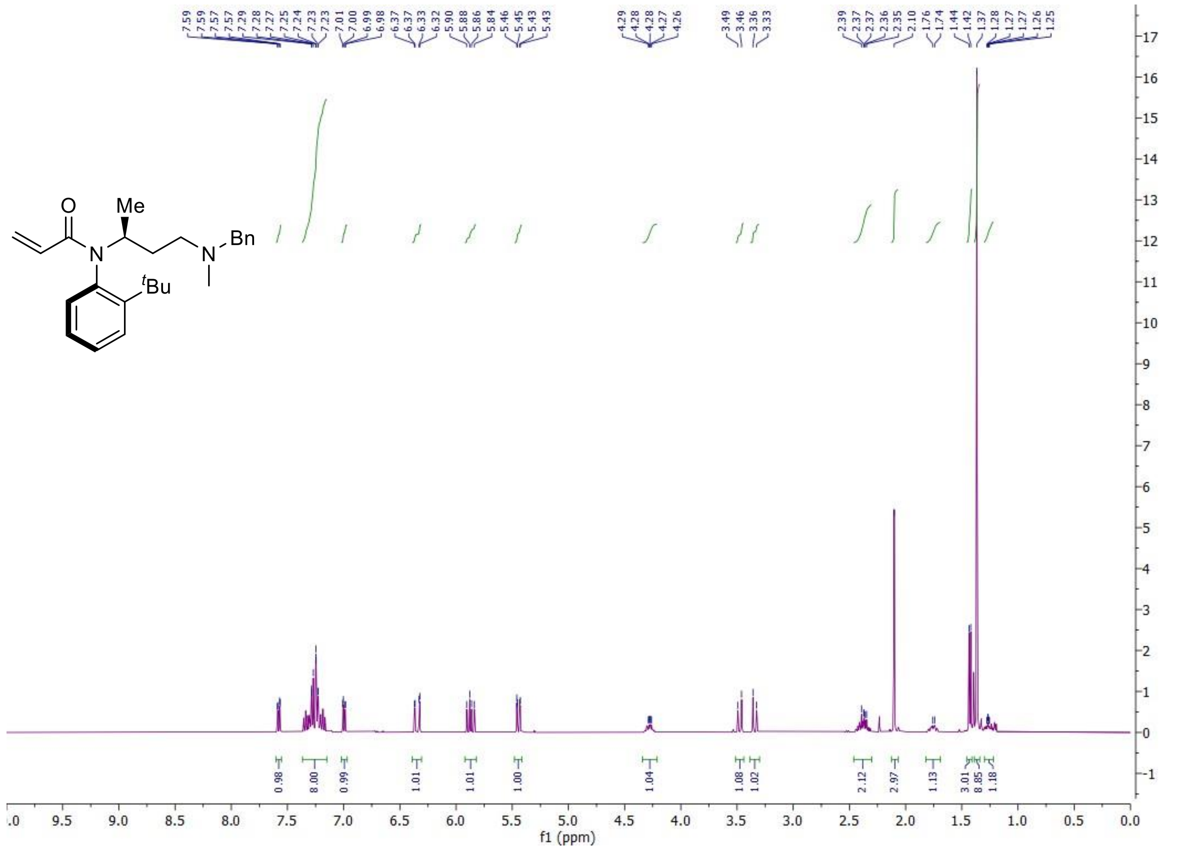

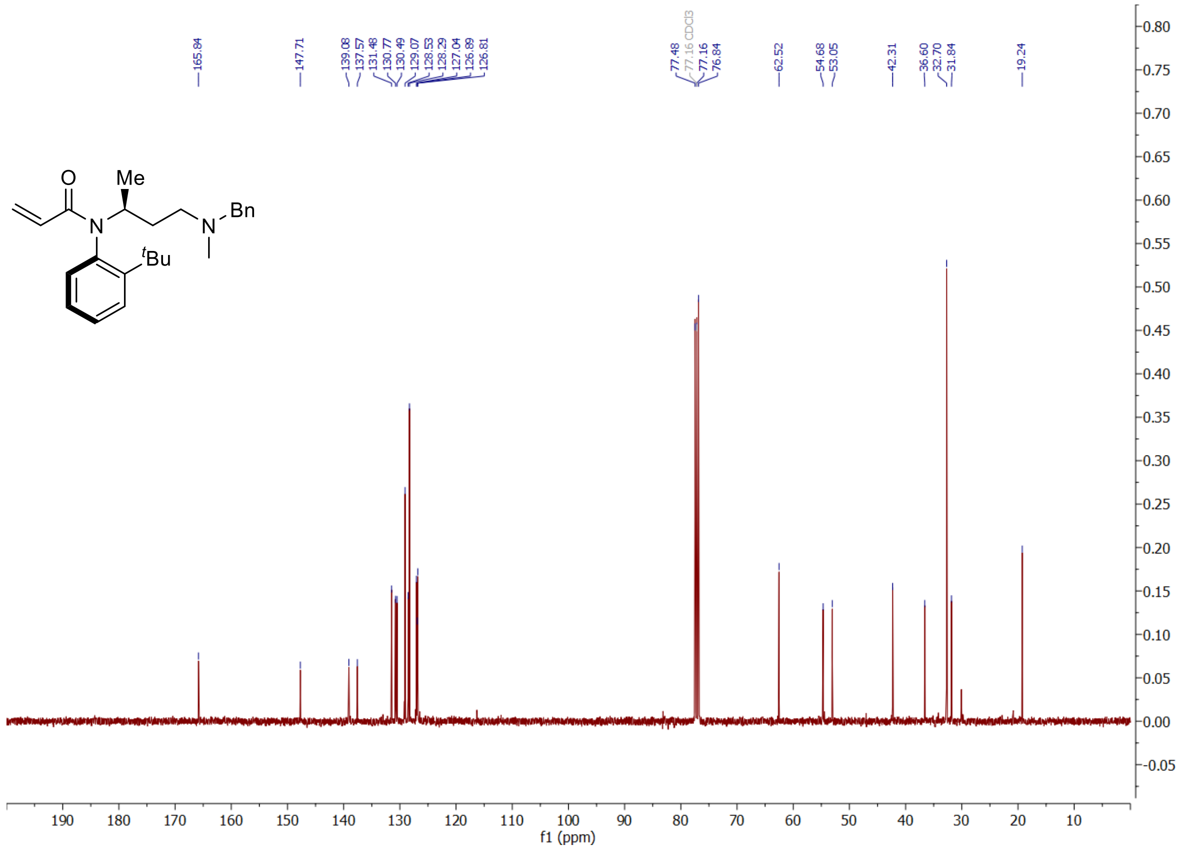

N-(4-(Benzyl(methyl)amino)butan-2-yl)-*N*-(2-(*tert*-butyl)phenyl)-2-methoxyacetamide (9d)

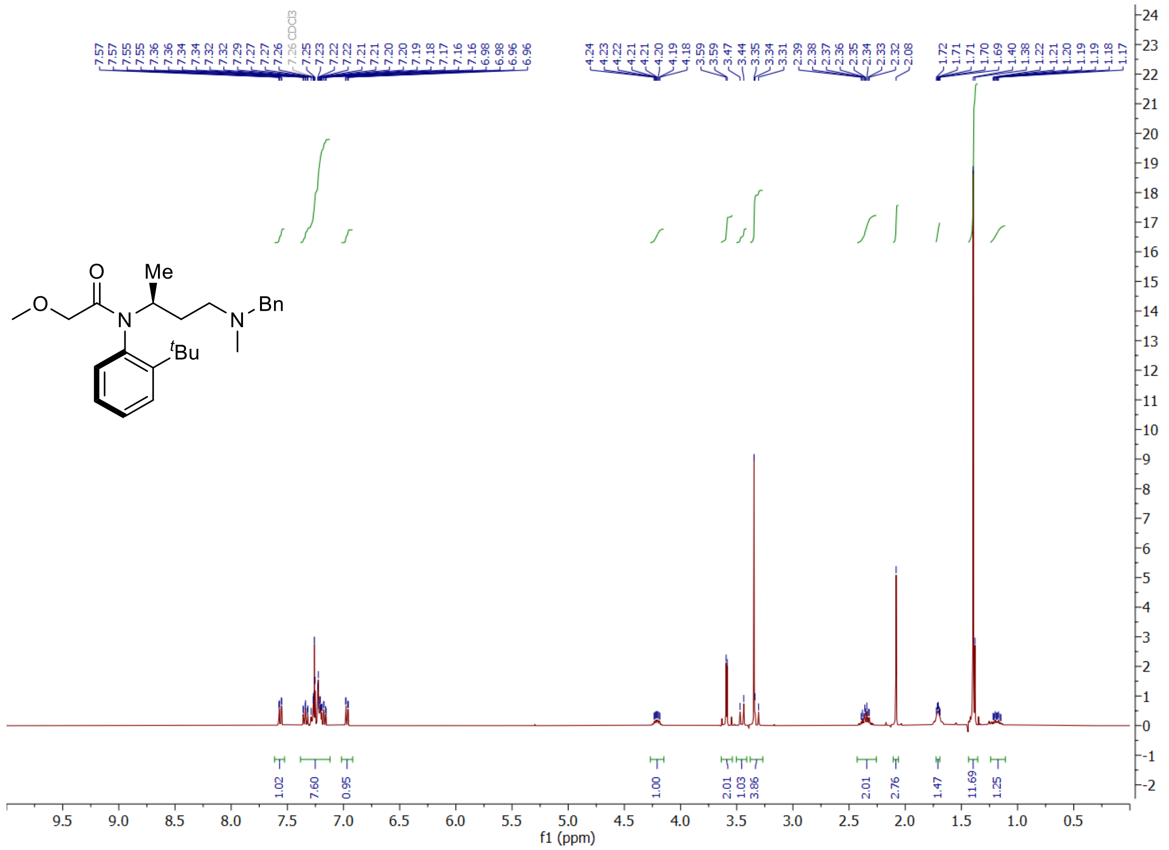

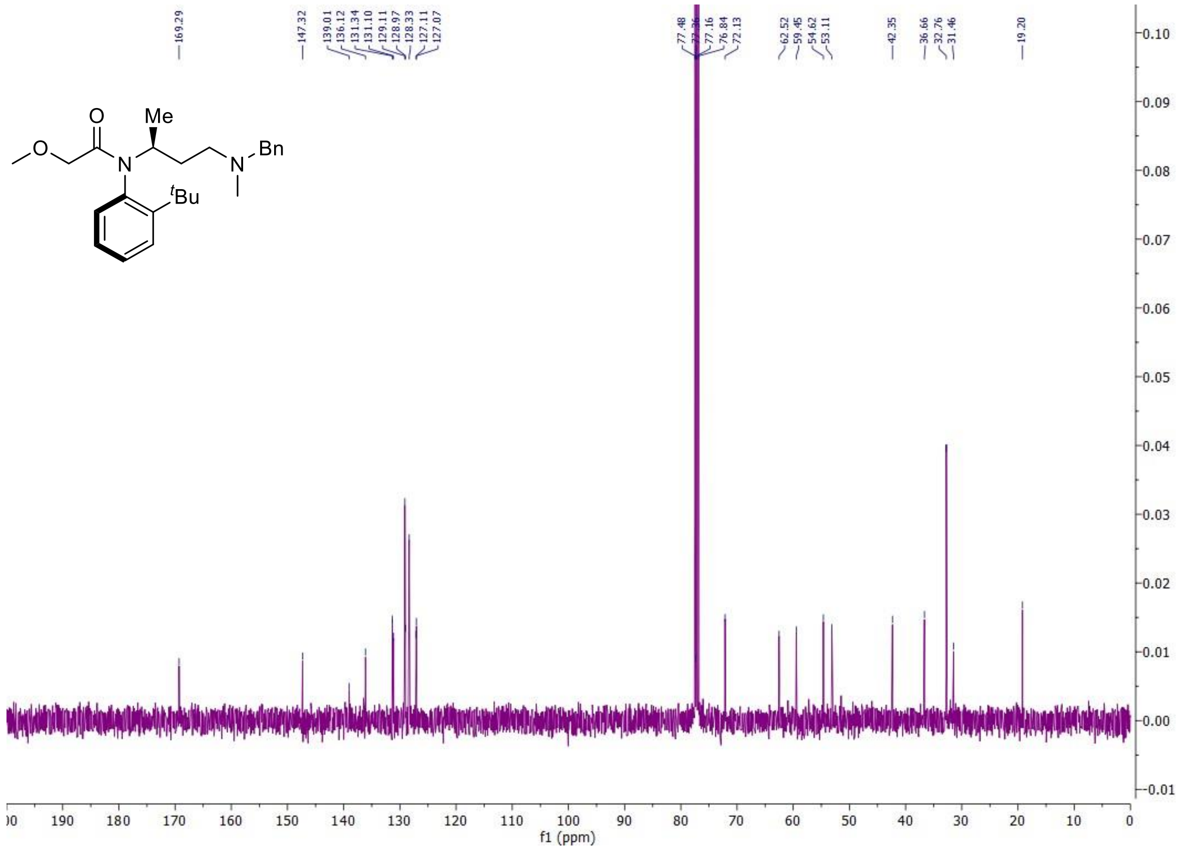

N-(2-(*tert*-Butyl)phenyl)-*N*-(4-(dipropylamino)butan-2-yl)benzamide (9e)

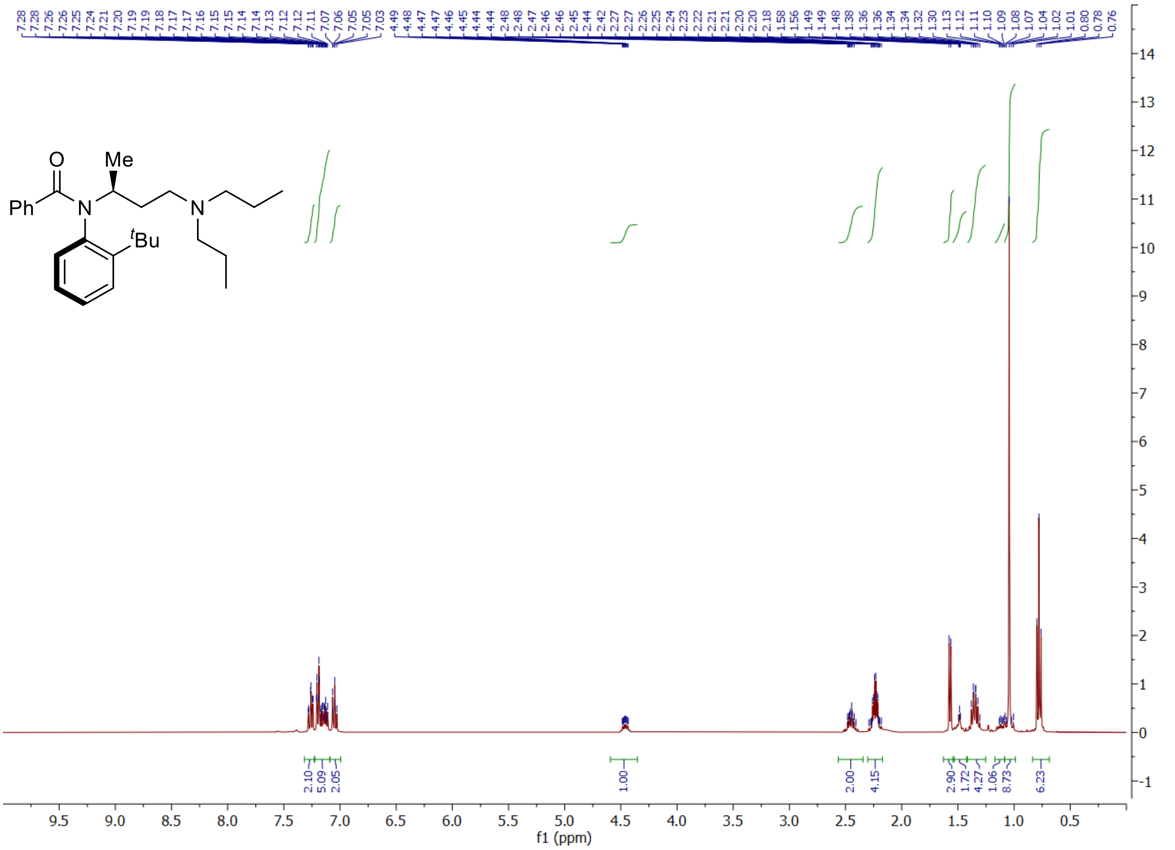

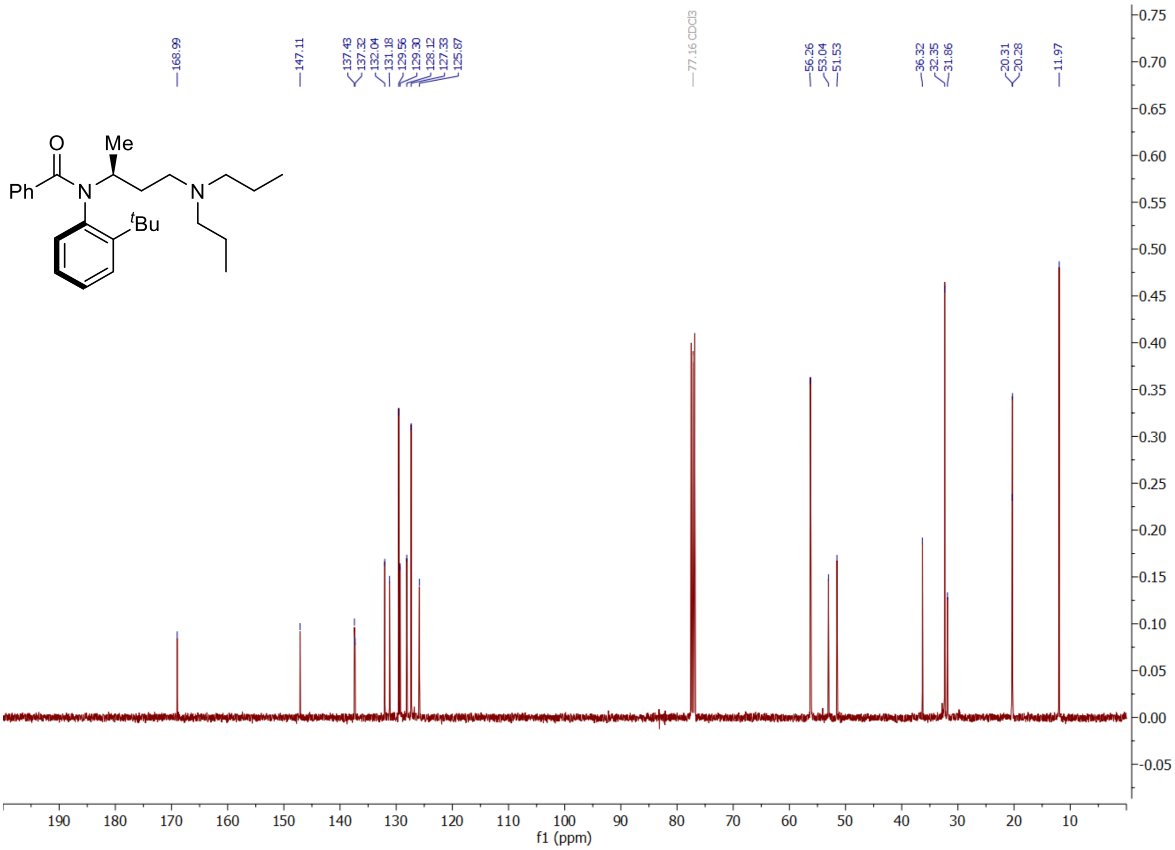

***N*-(4-(Benzyl(methyl)amino)butan-2-yl)-*N*-(2-(*tert*-butyl)phenyl)-2-methylbenzamide (9f)**

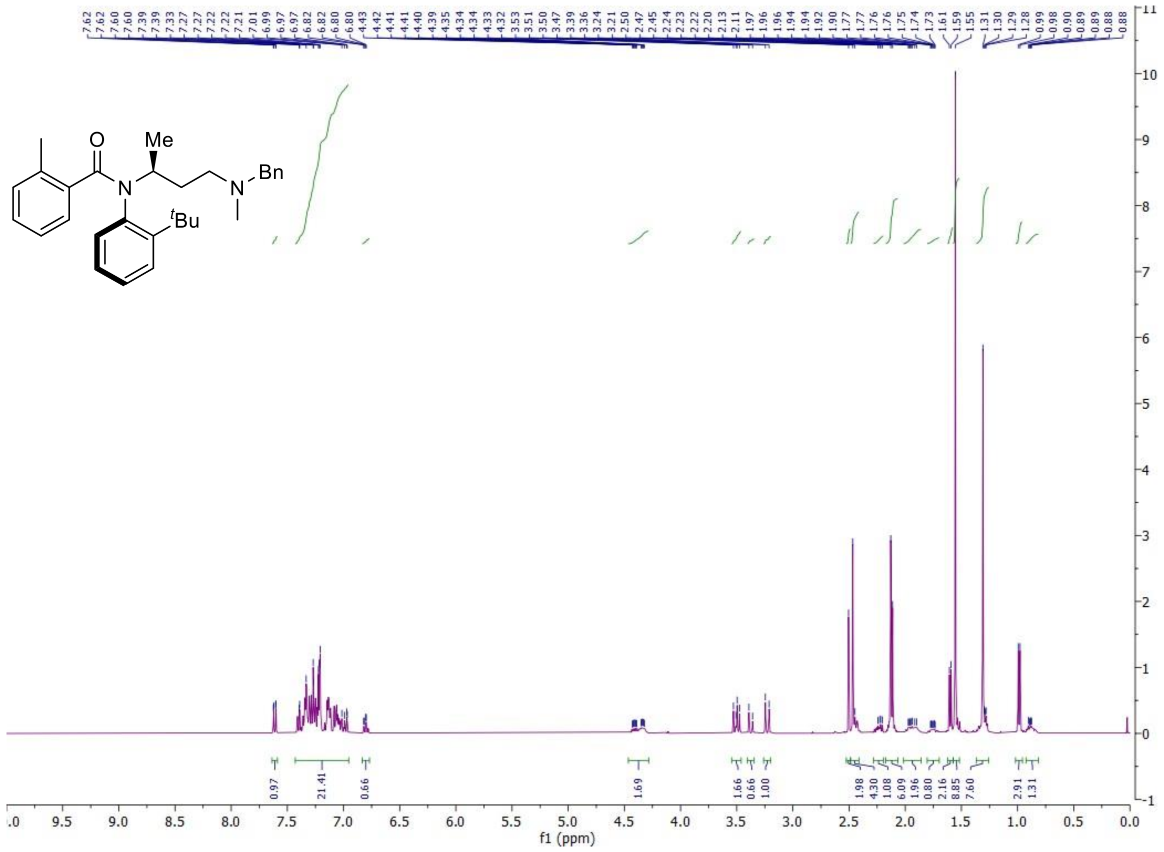

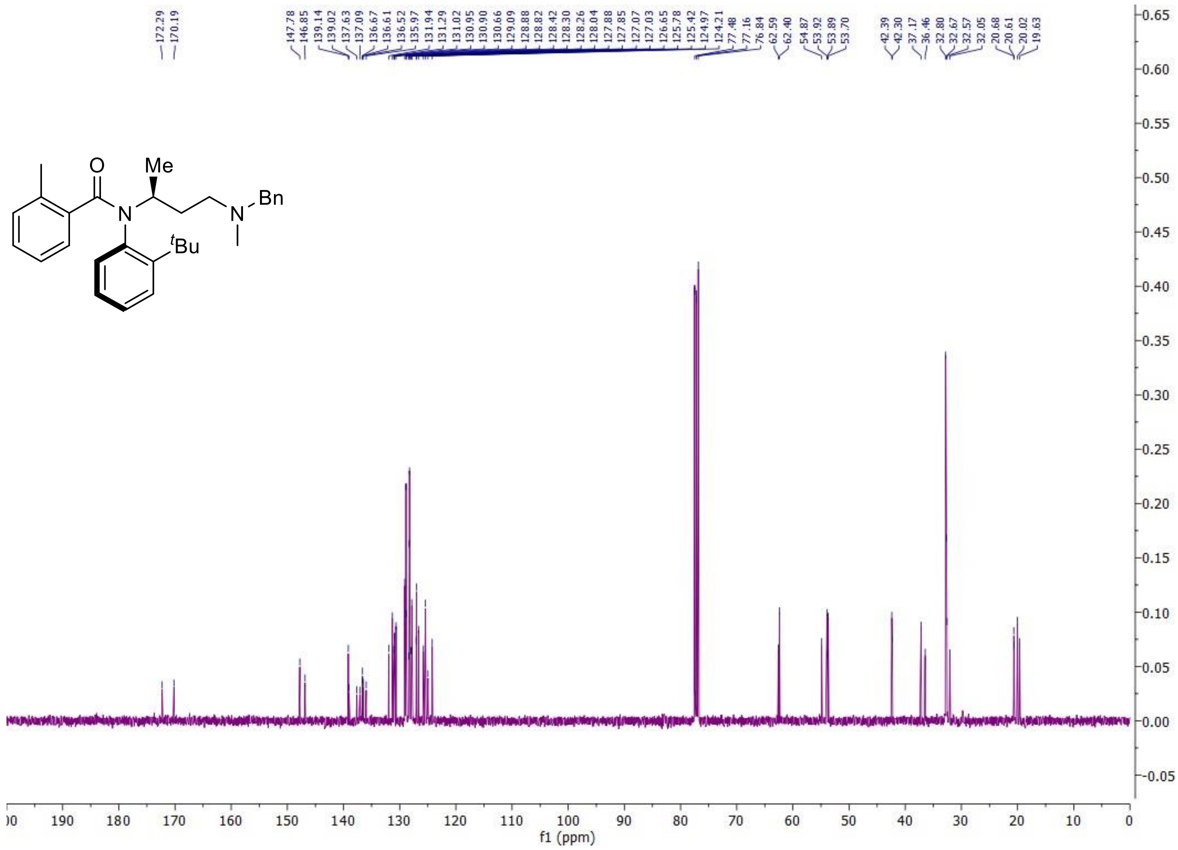

2-(*tert*-Butyl)-*N*-(hexan-2-yl)aniline (15)

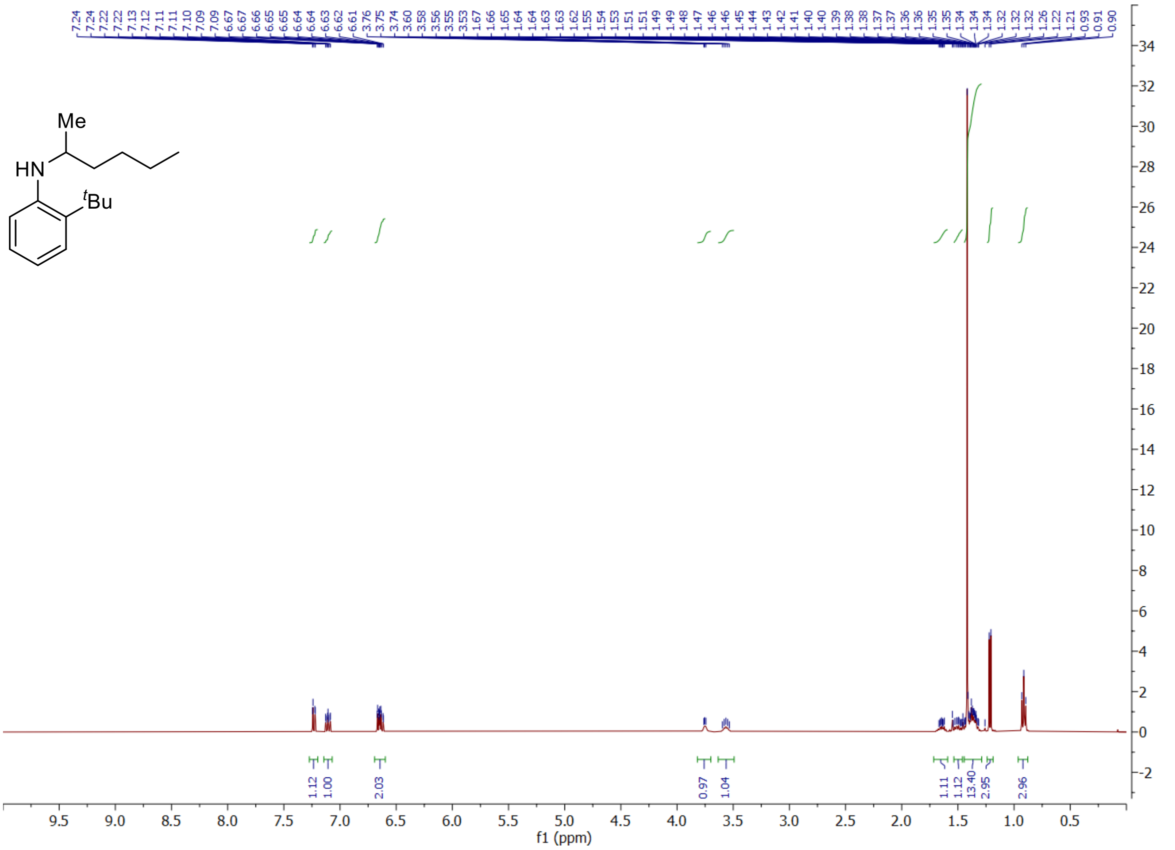

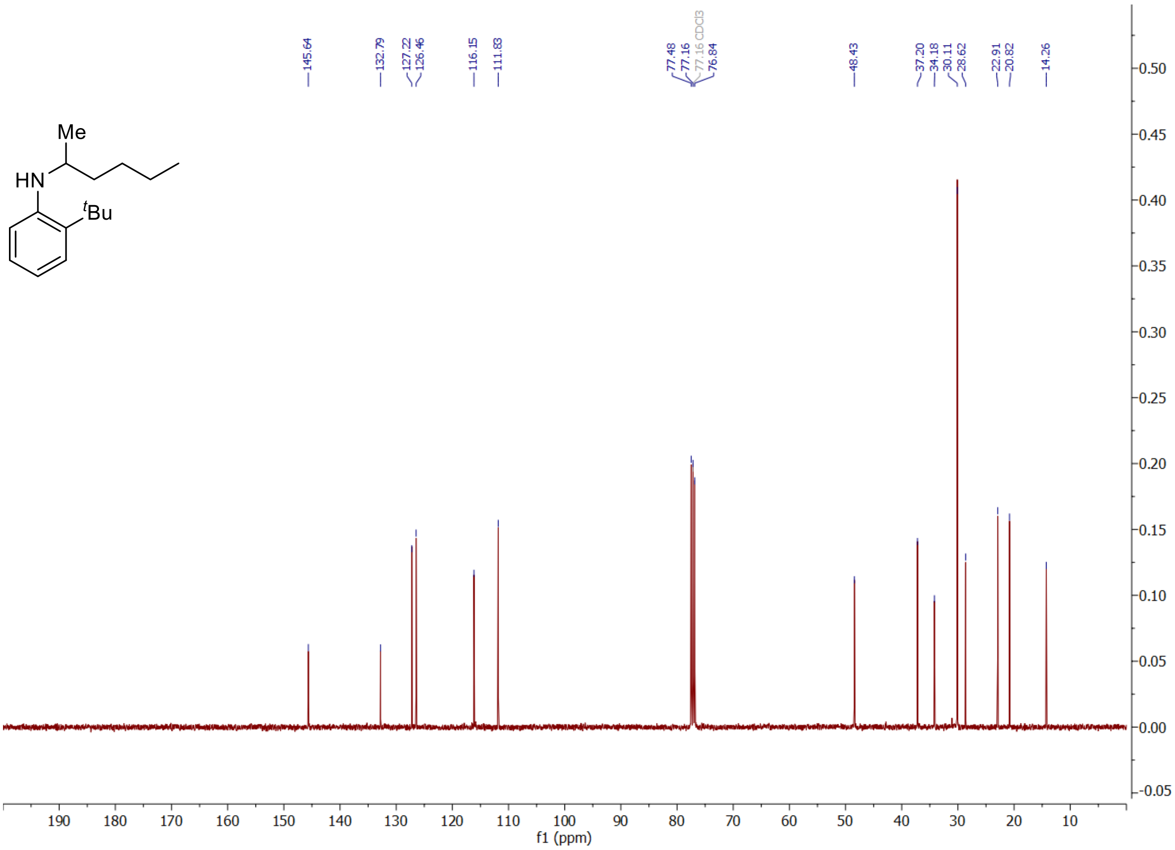

N-(2-(tert-Butyl)phenyl)-N-(hexan-2-yl)propionamide (16)

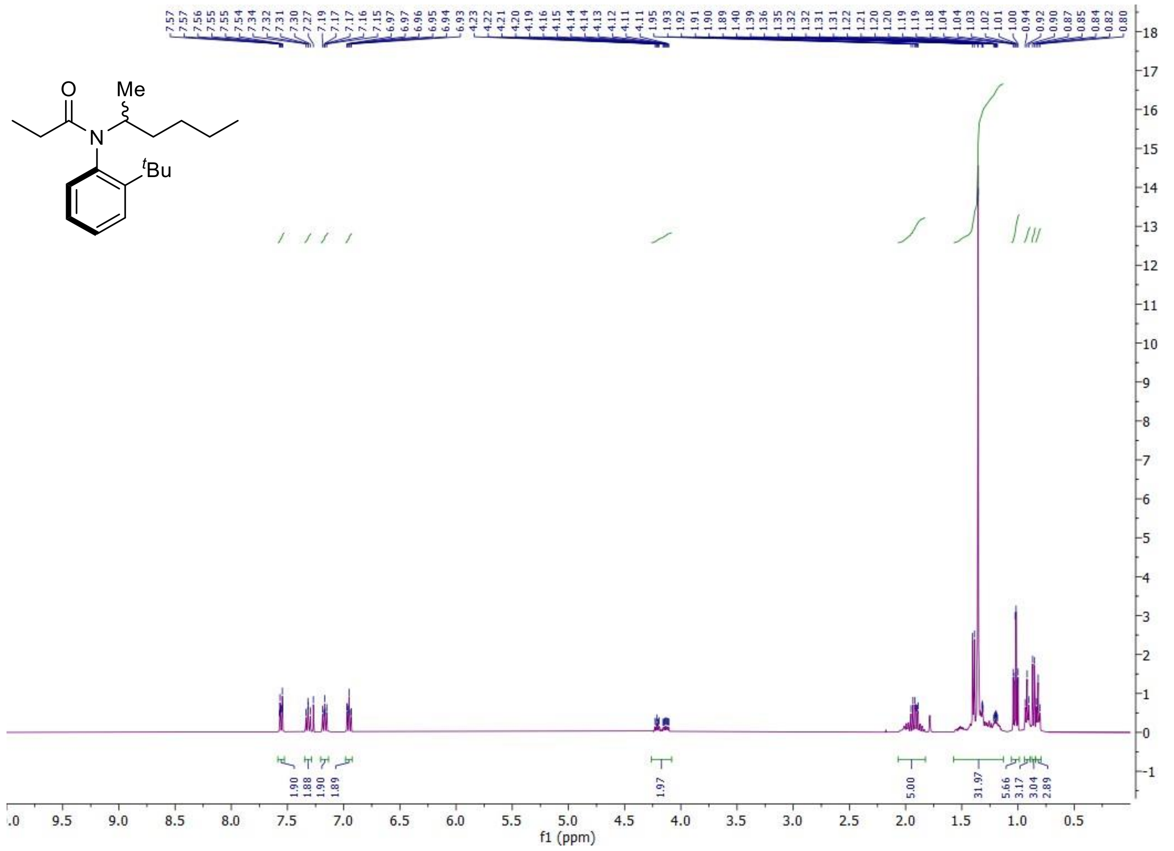

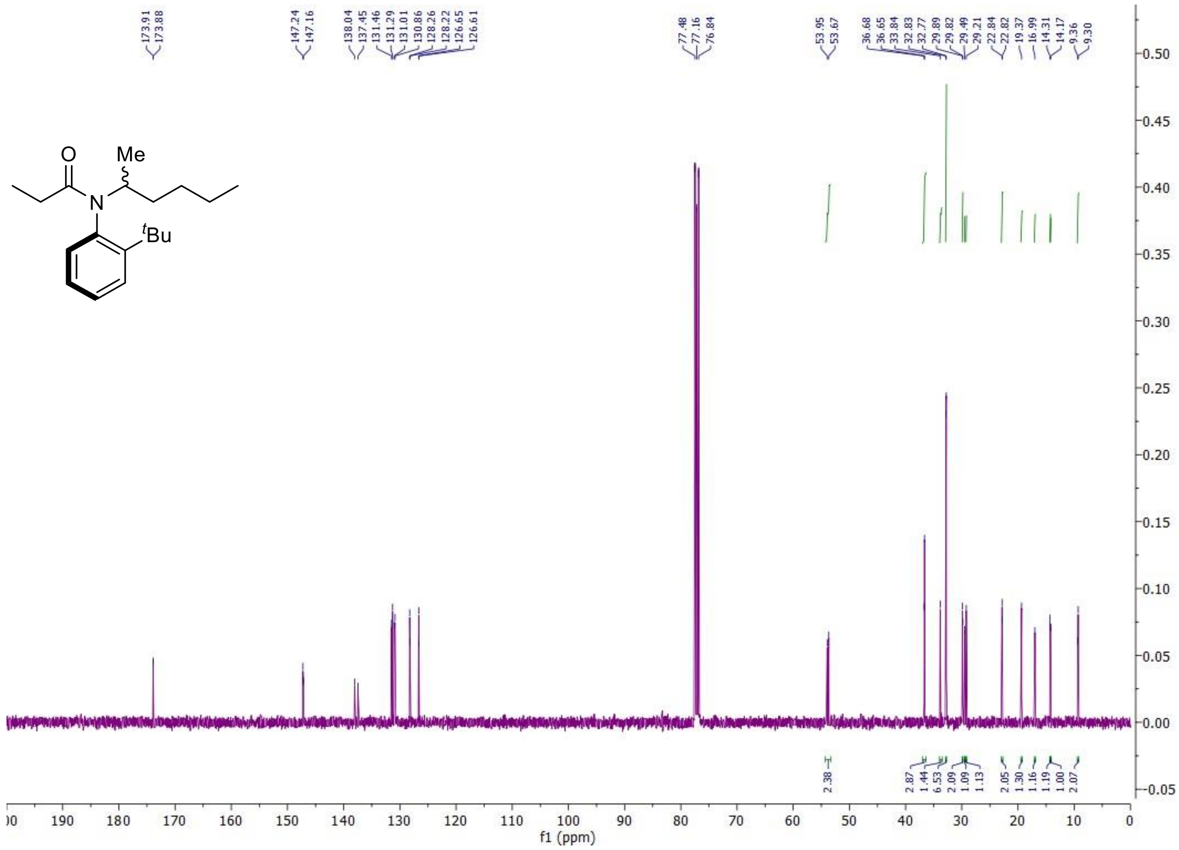

N,N,2-Trimethylpyridin-4-amine (S12)

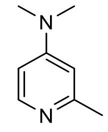

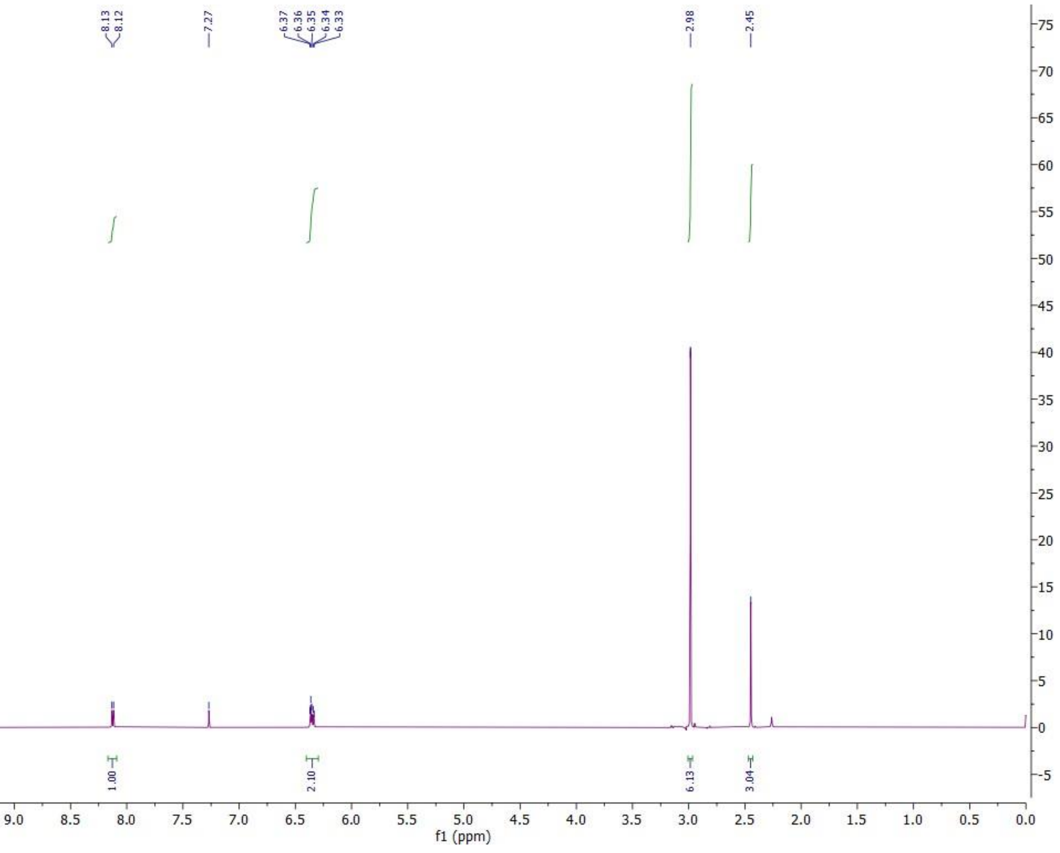

2-(2-((2-(*tert*-Butyl)phenyl)amino)-2-phenylethyl)-*N*,*N*-dimethylpyridin-4-amine (4h)

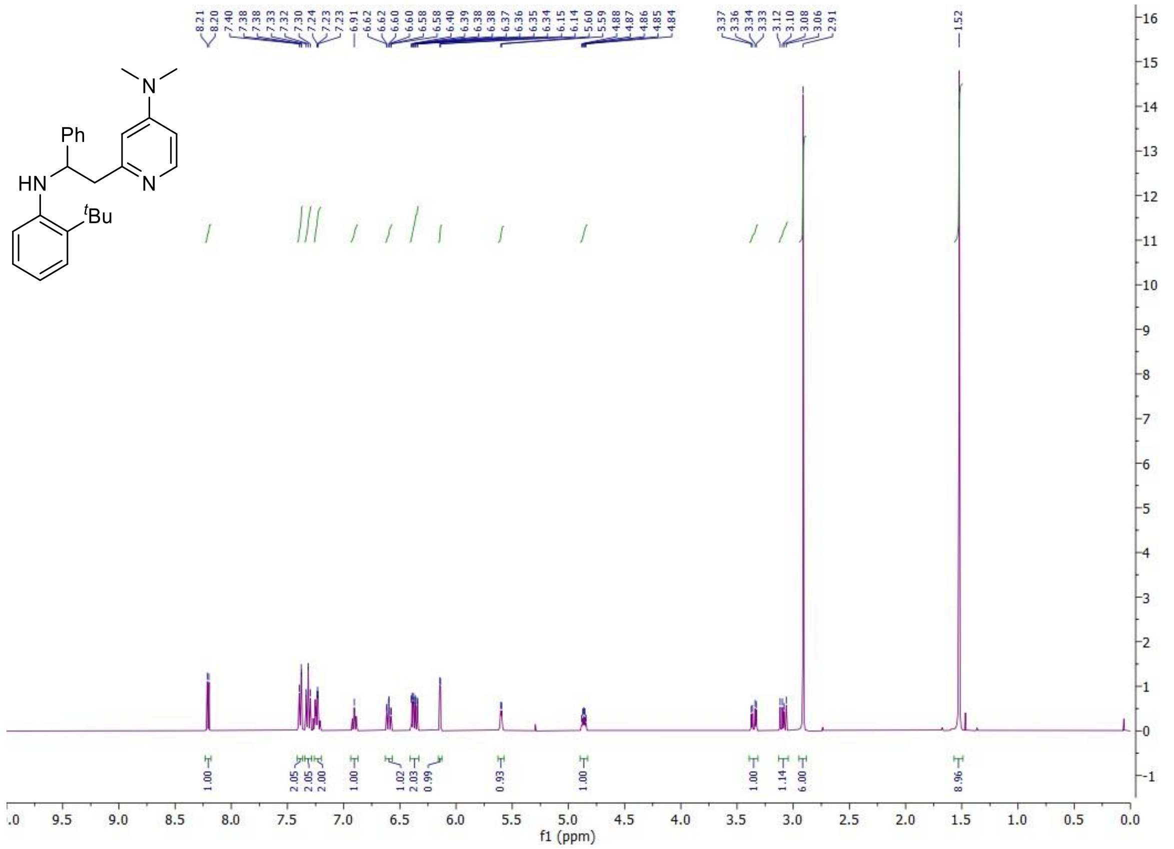

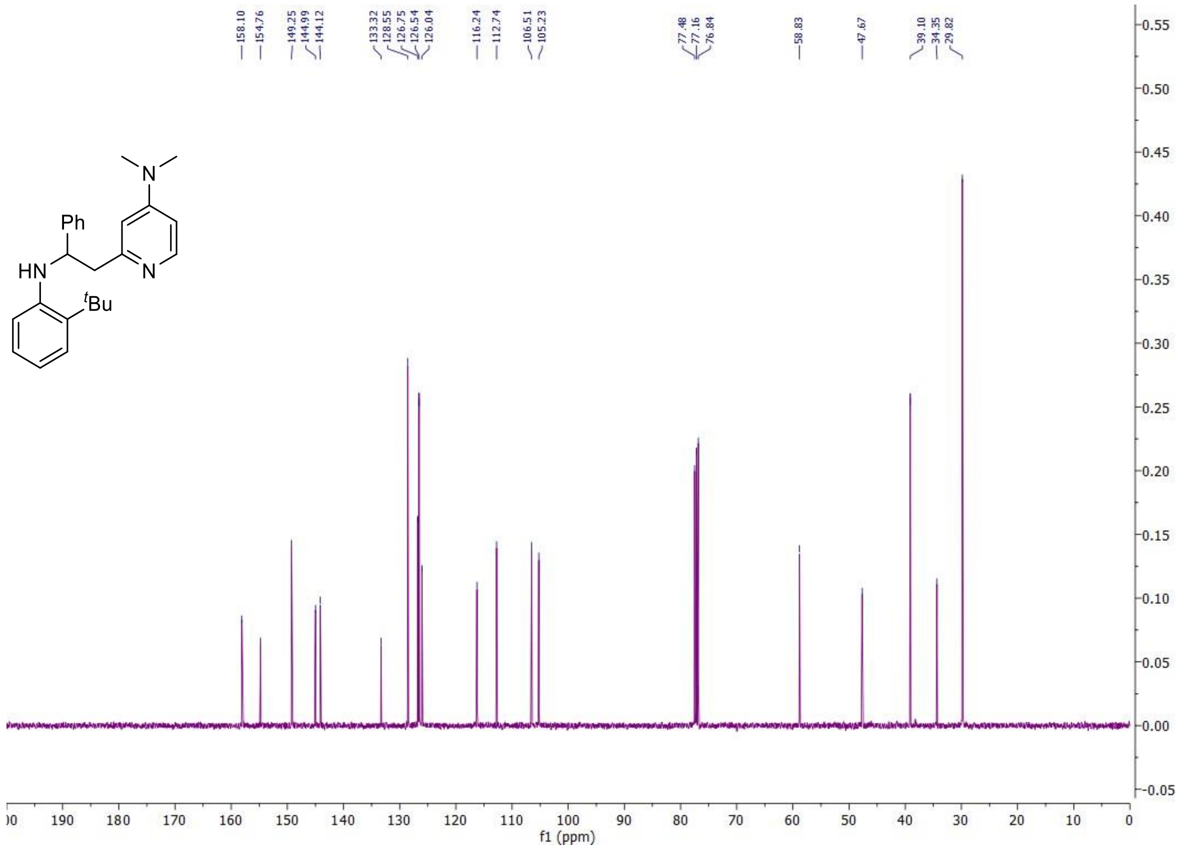

N-(2-(*tert*-Butyl)phenyl)-N-(phenyl(pyridin-2-yl)methyl)piperidine-4-carboxamide (18)

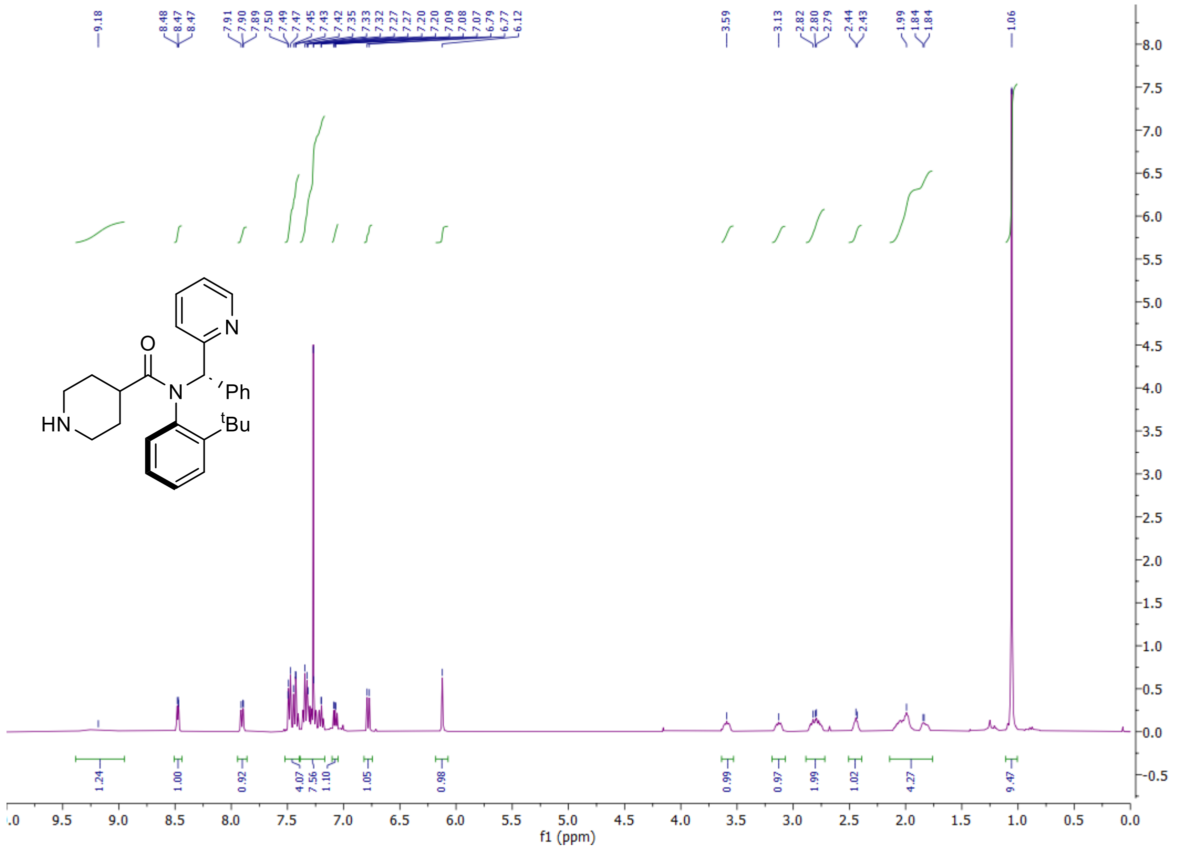

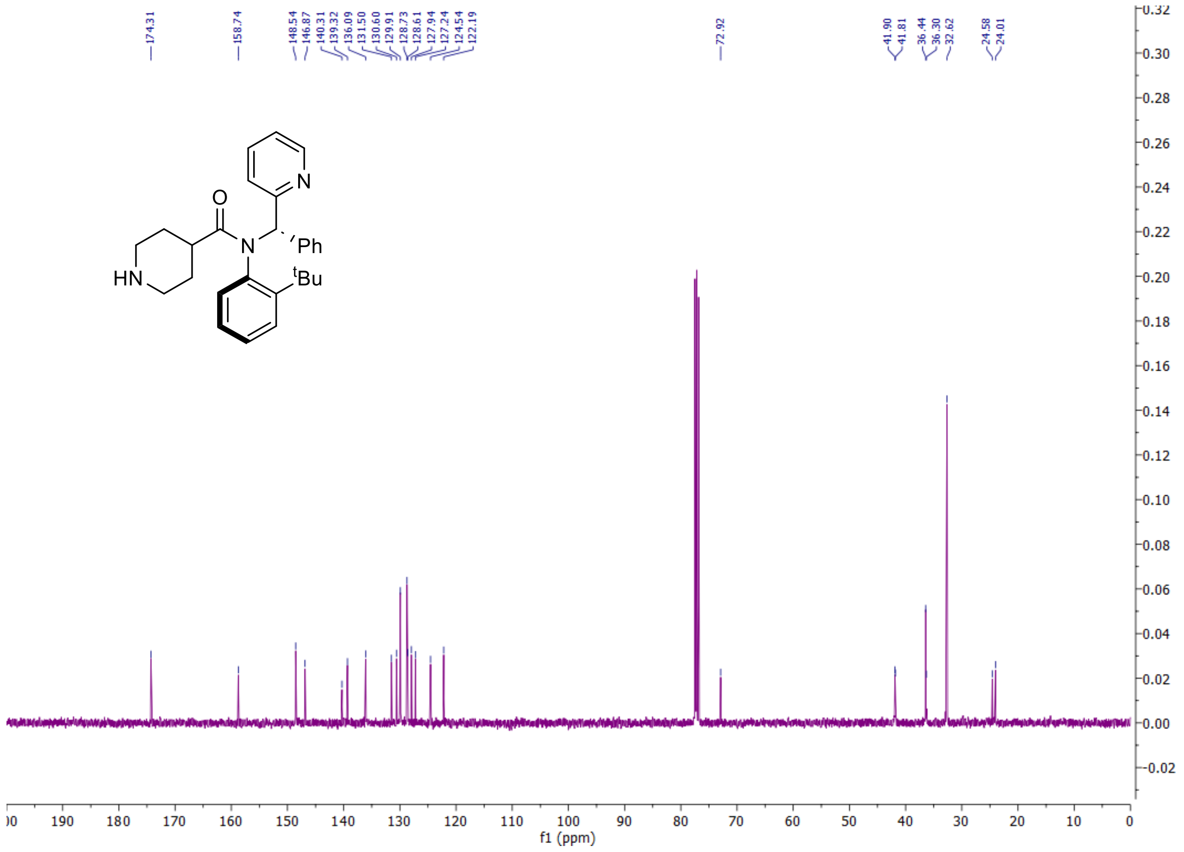

N-(2-(*tert*-Butyl)phenyl)-N-(phenyl(pyridin-2-yl)methyl)-1-tosylpiperidine-4-carboxamide (19)

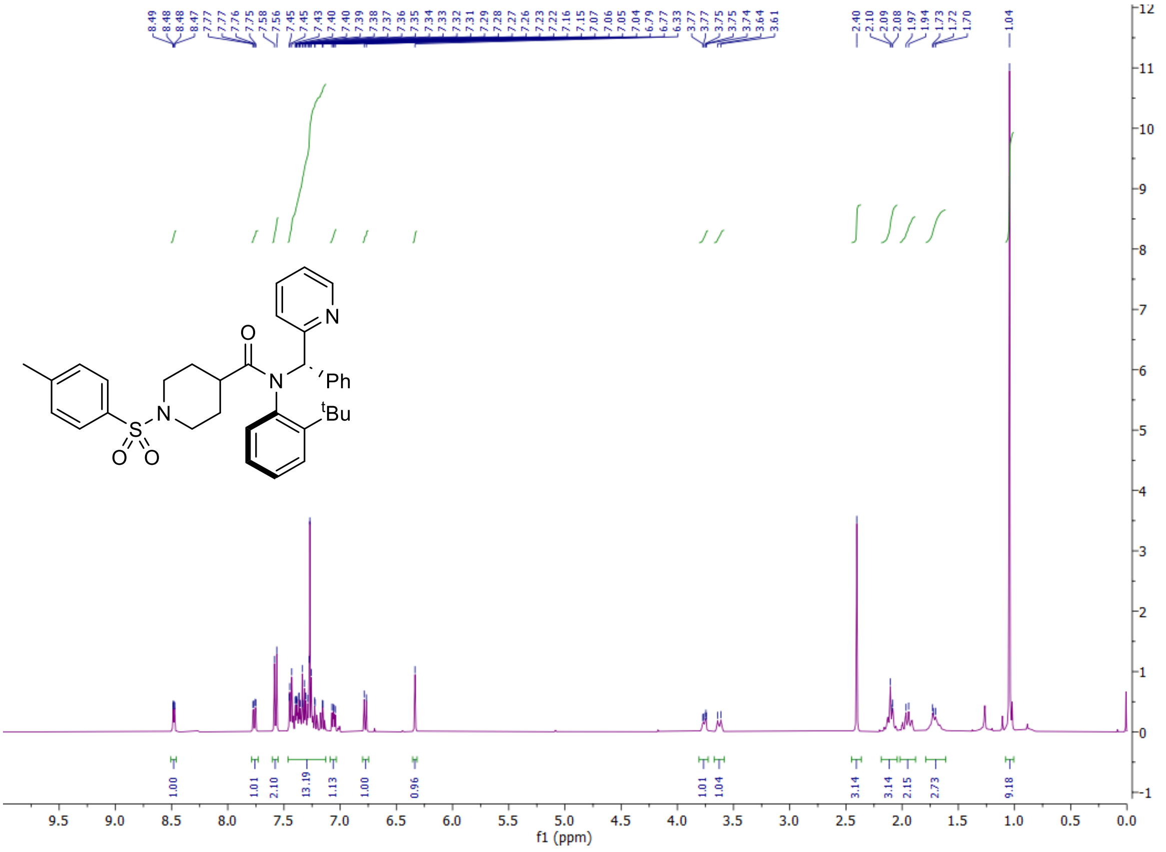

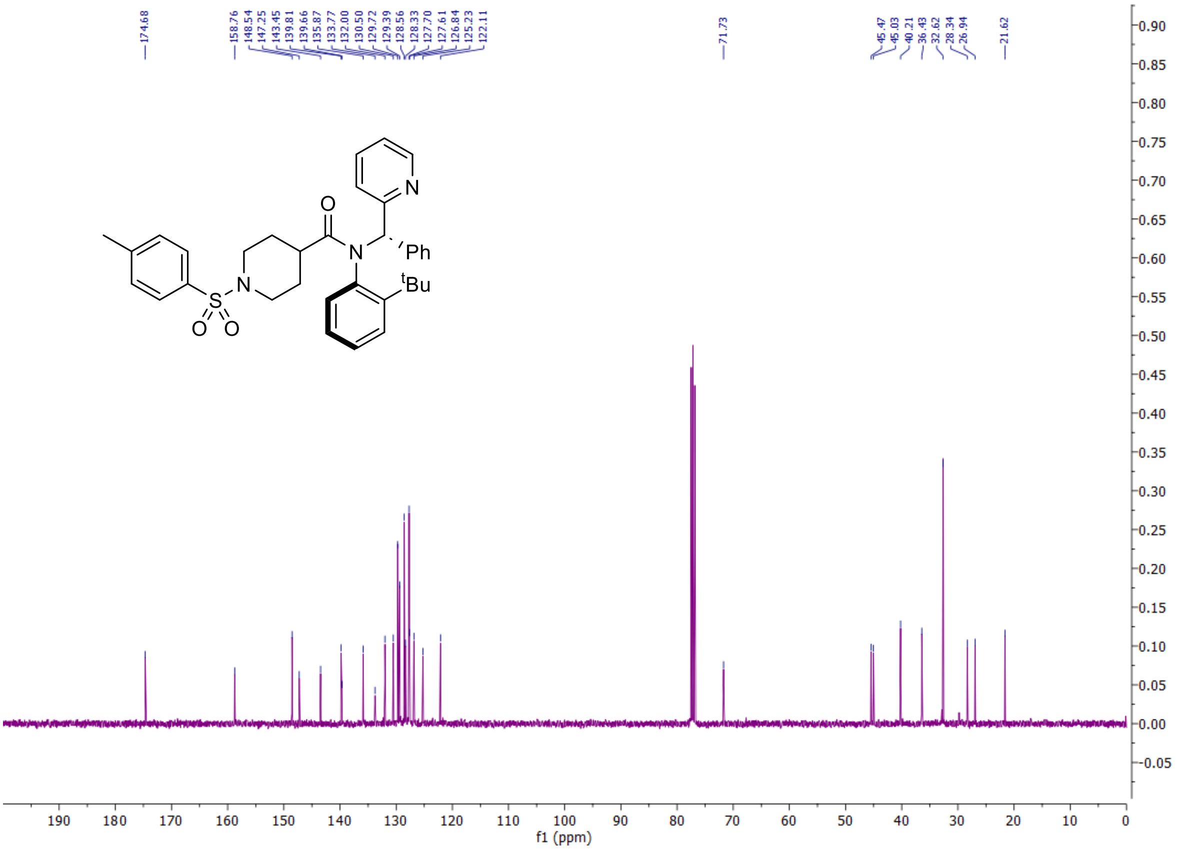

2-(2-(*N*-(2-Iodo-4,6-dimethylphenyl)benzamido)-2-phenylethyl)pyridine 1-oxide (20)

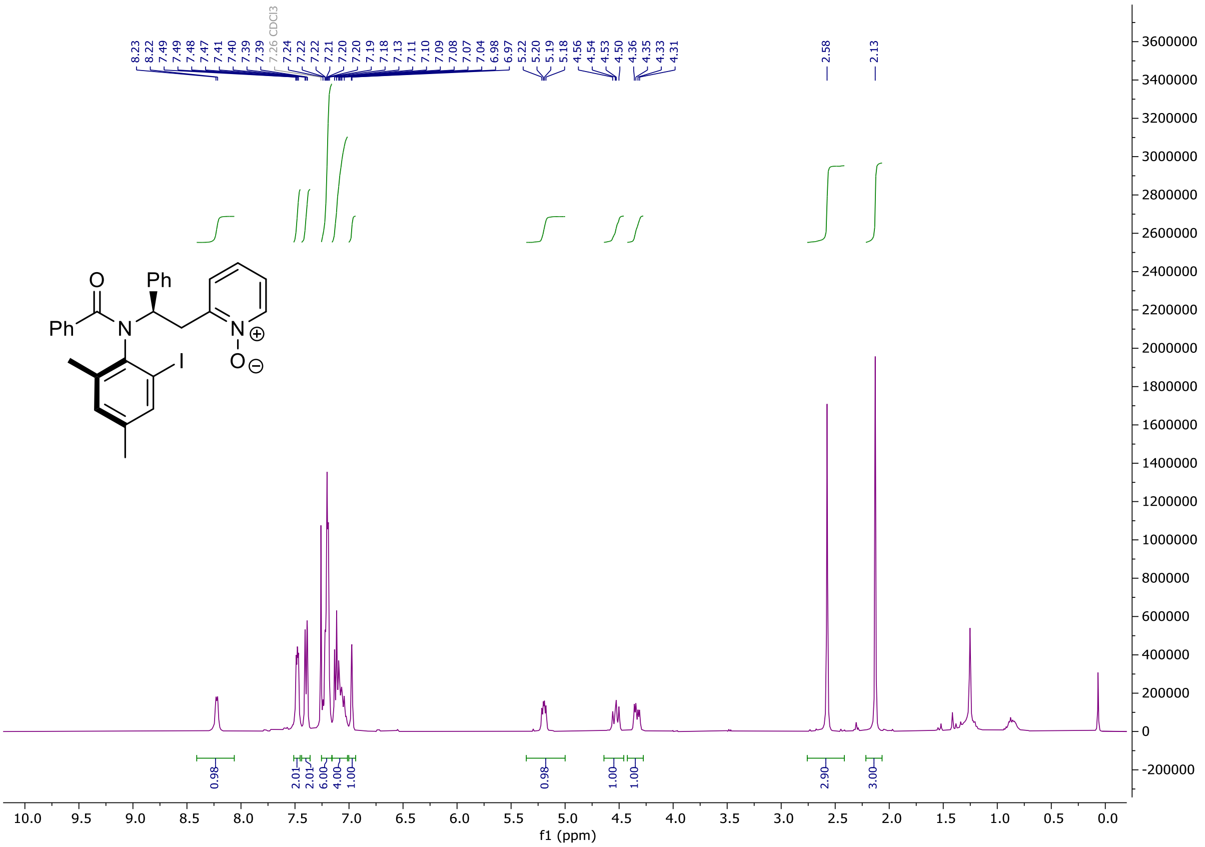

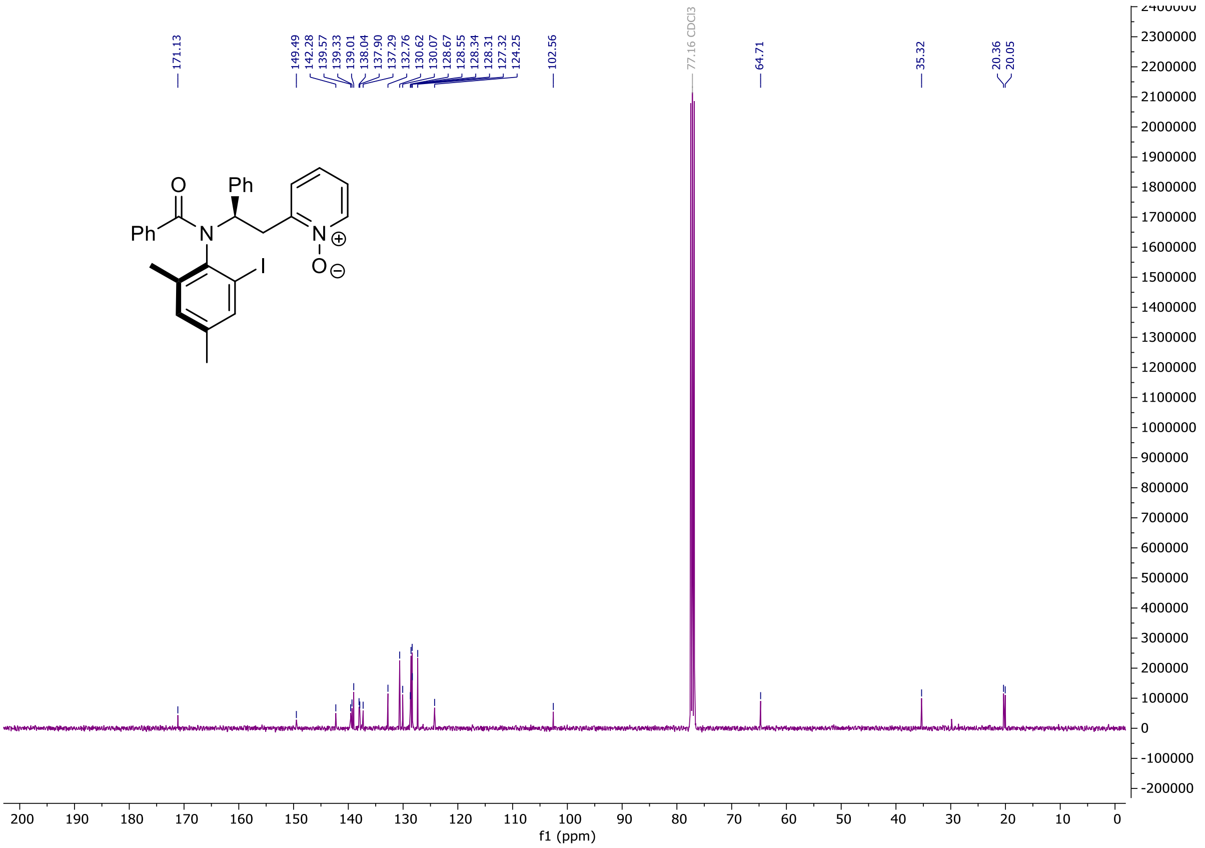

4-Amino-*N*-(2-(*tert*-butyl)phenyl)-*N*-(1-phenyl-2-(pyridin-2-yl)ethyl)benzamide (21)

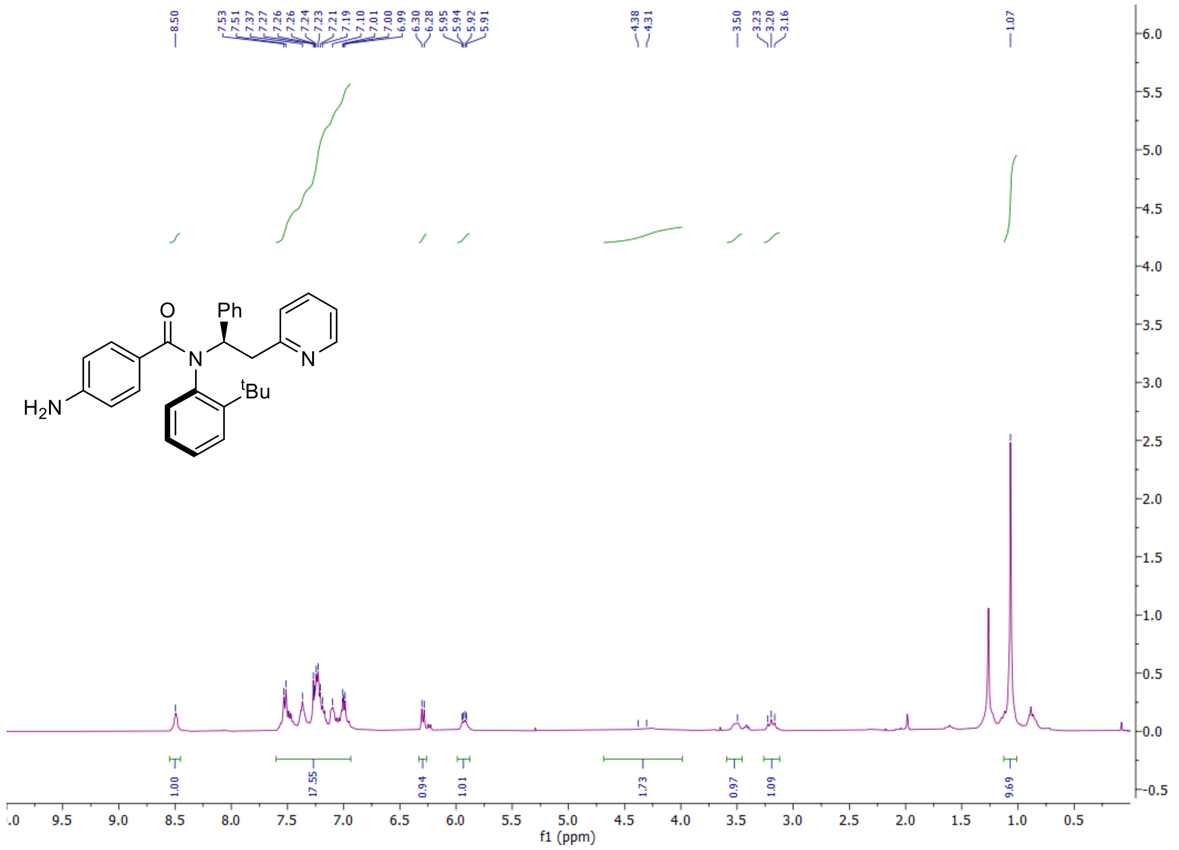

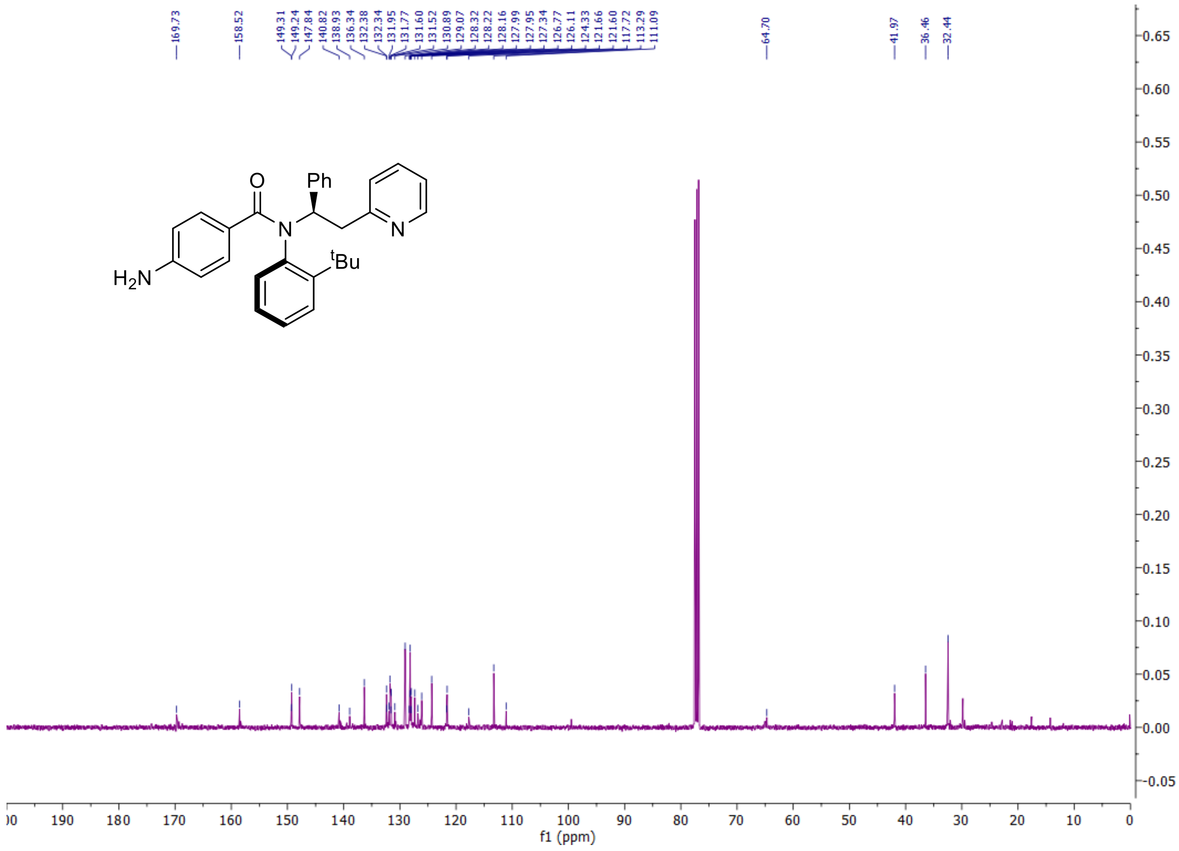

N-(2-(*tert*-Butyl)phenyl)-4-(4-methoxybenzamido)-N-(1-phenyl-2-(pyridin-2-yl)ethyl)benzamide (22)

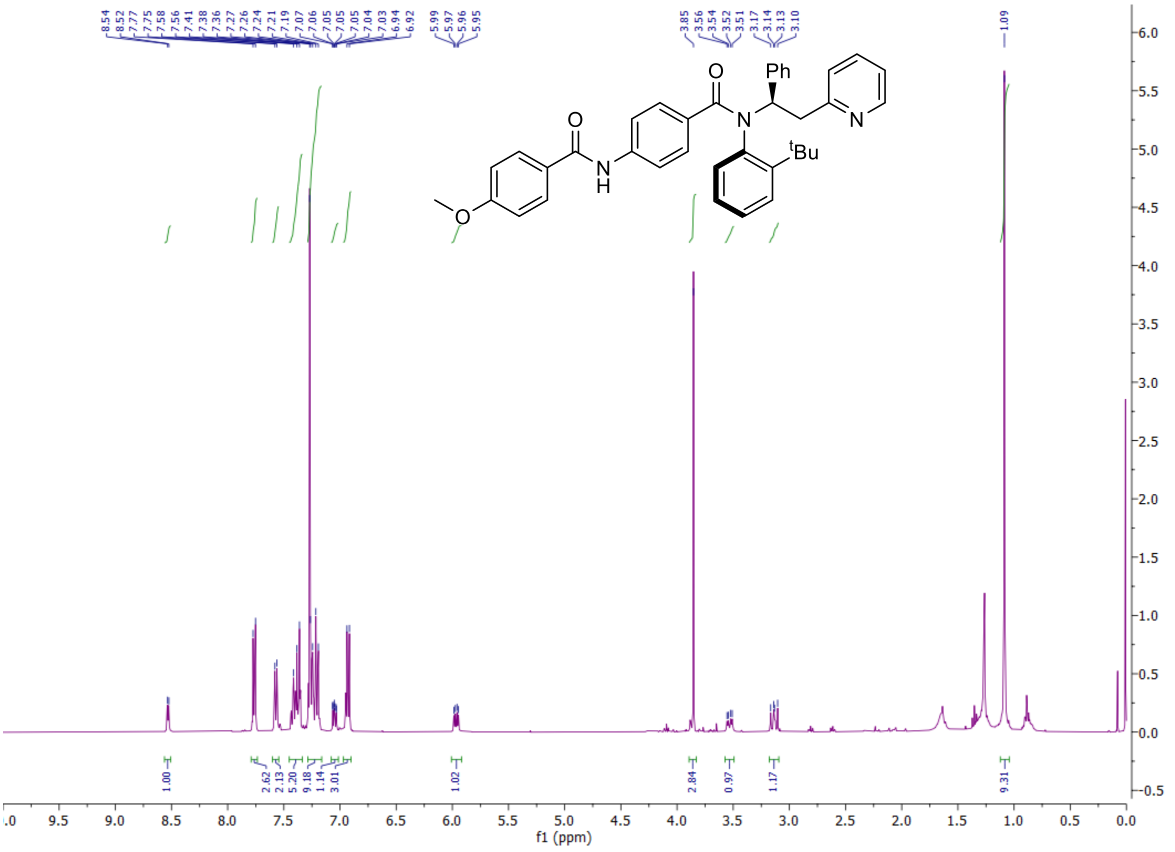

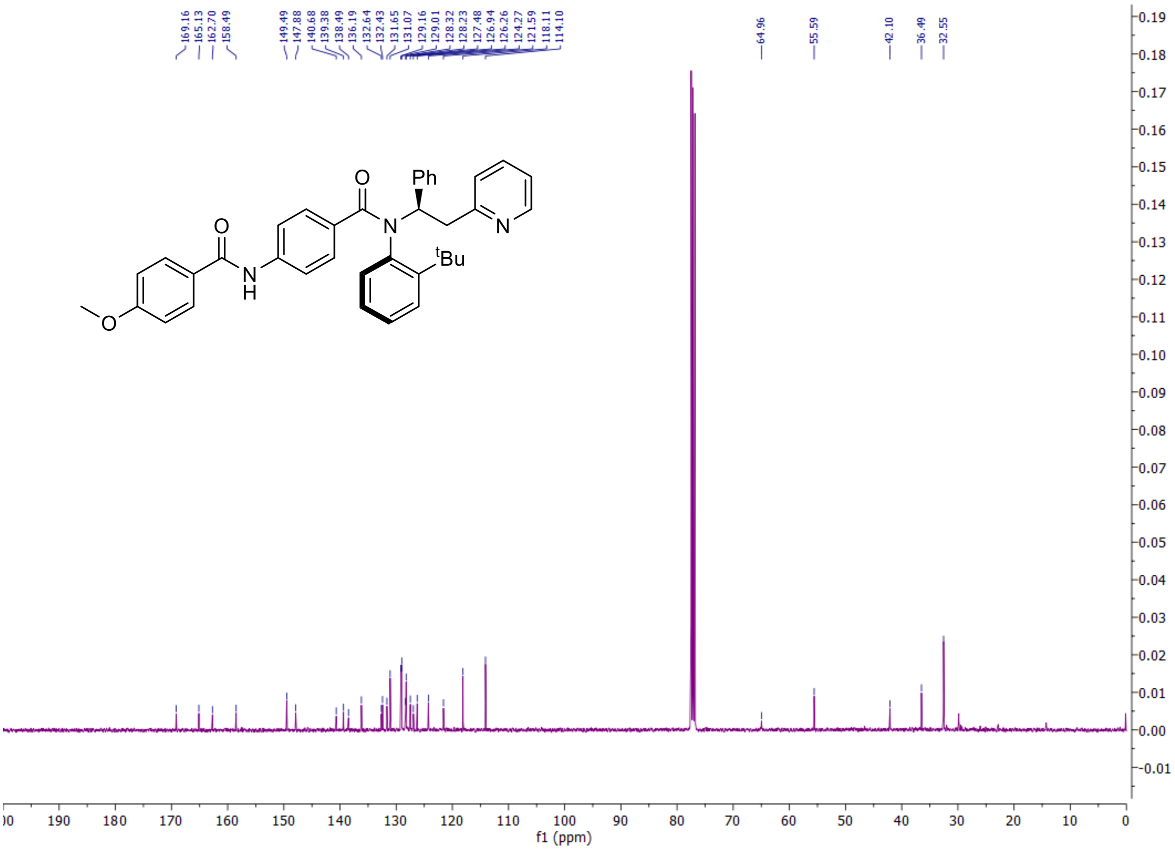

N-(2-(tert-Butyl)phenyl)-4'-methoxy-N-(1-phenyl-2-(pyridin-2-yl)ethyl)-[1,1'-biphenyl]-4-carboxamide (24)

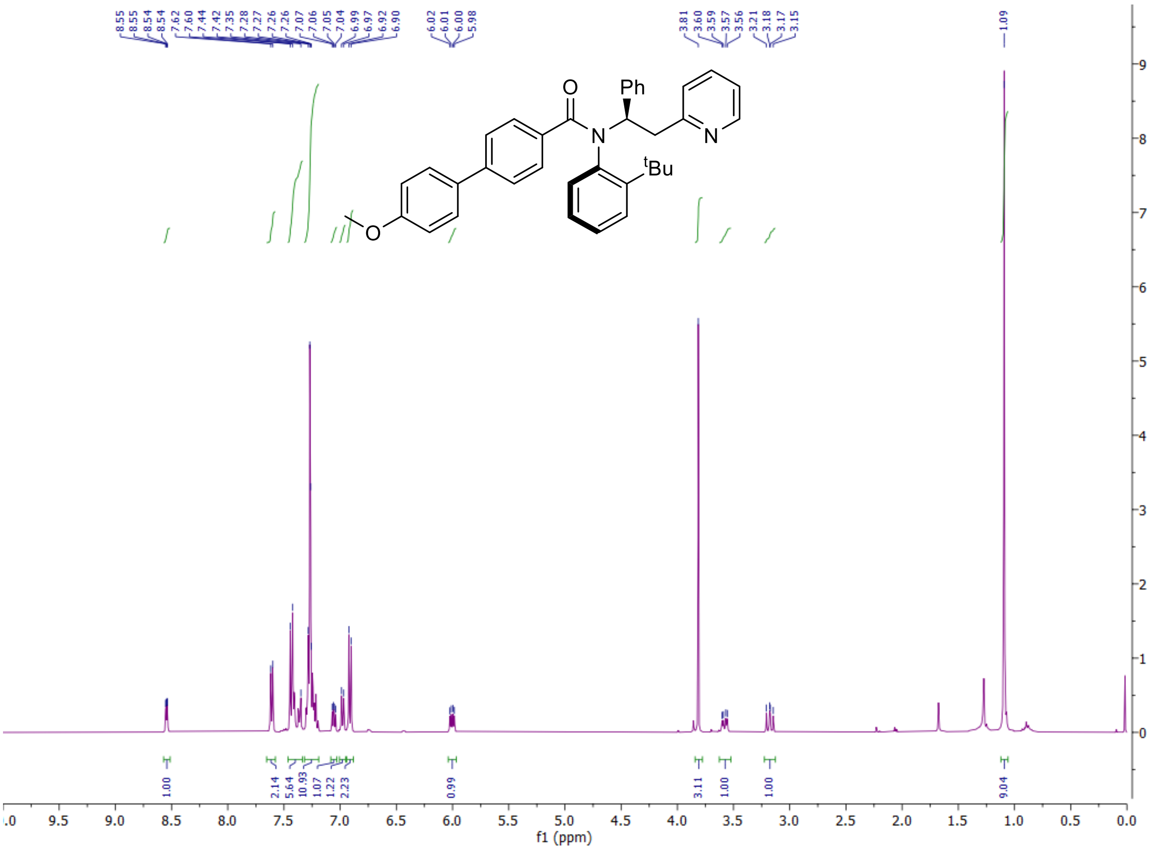

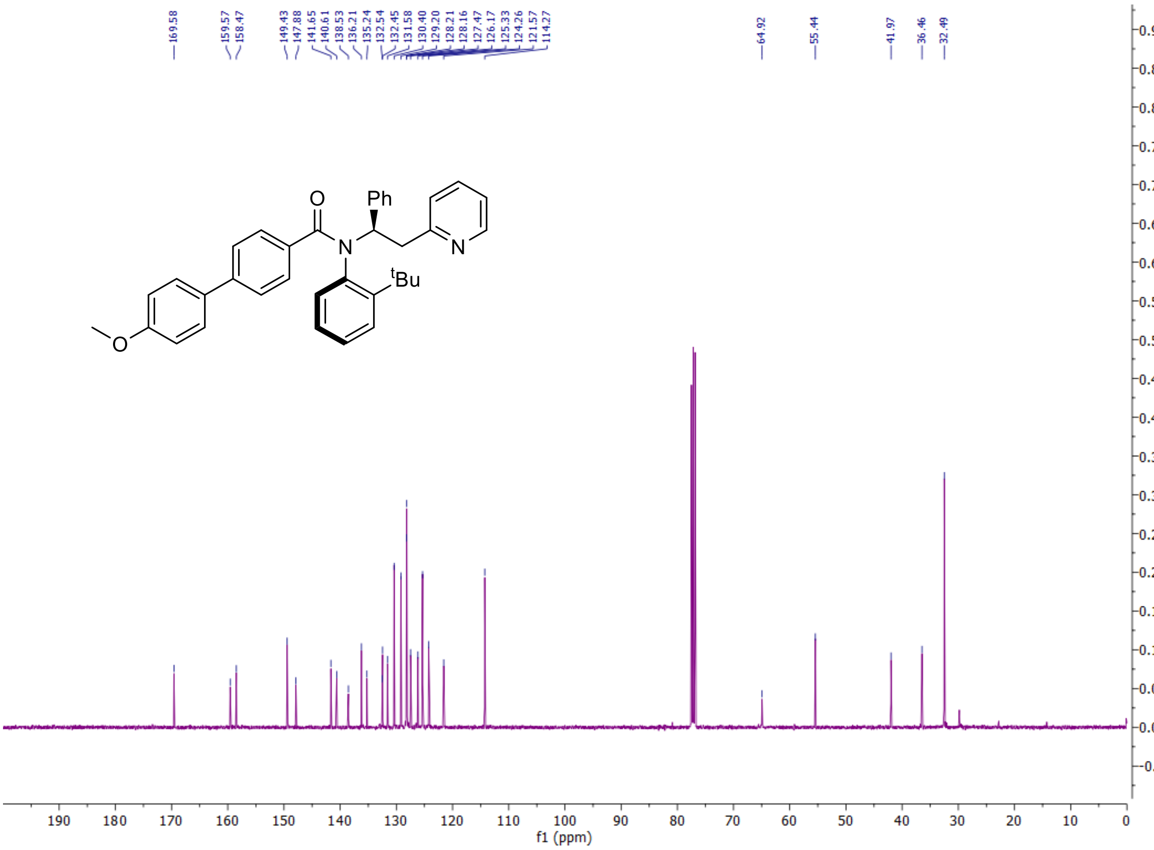

N^1-(2-(*tert*-Butyl)phenyl)-N^5-cyclopropyl-N^1-(phenyl(pyridin-2-yl)methyl)glutaramide (26)

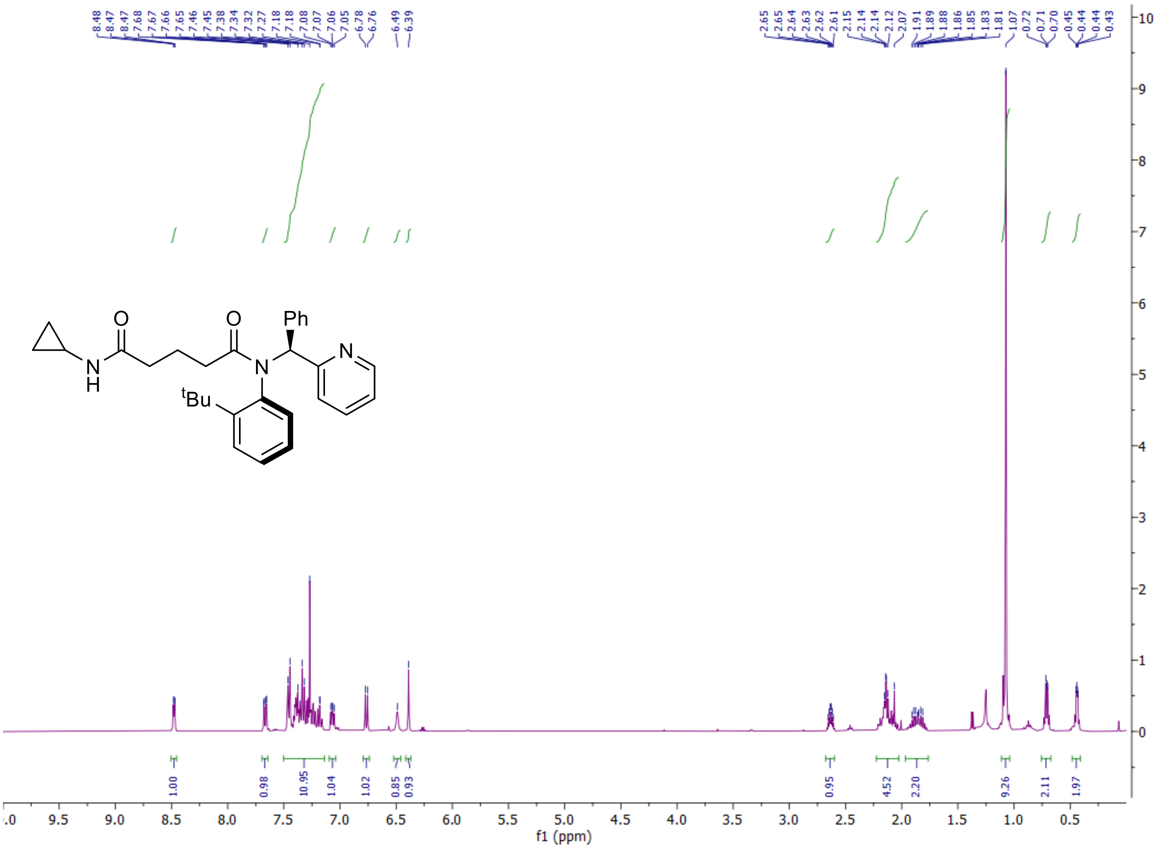

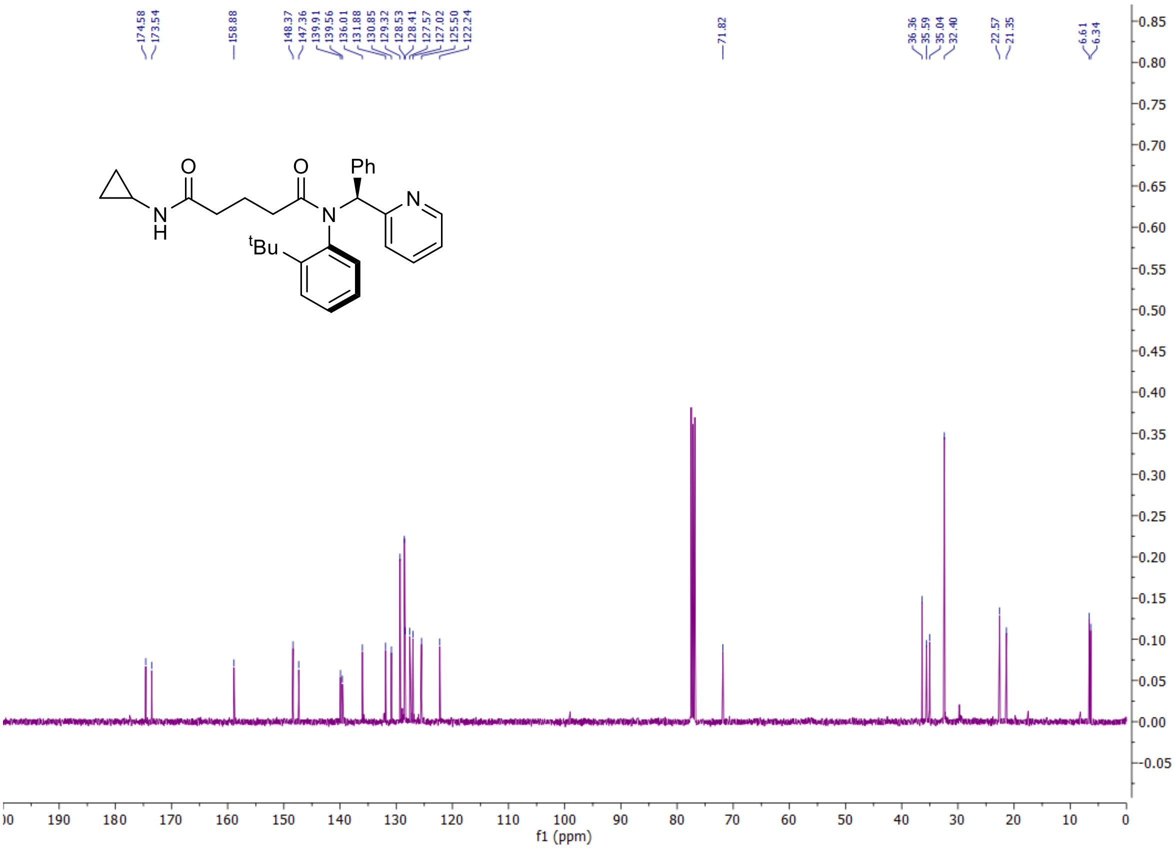

9) References

1. A. Takács, A. Fodor, J. Németh and Z. Hell, *Synth. Commun.*, 2014, **44**, 2269–2275
2. E. Pasquinet, P. Rocca, A. Godard, F. Marsais and G. Quéguiner, *J. Chem. Soc. Perkin Trans. 1*, **1998**, 3807–3812.
3. H. Alharbi, M. Elsherbini, J. Qurban and T. Wirth, *Chem. Eur. J.*, 2021, **27**, 4317–4321.
4. M. Lautens, E. Tayama and C. Herse, *J. Am. Chem. Soc.*, 2005, **127**, 72–73.
5. J. Rong, L. Deng, P. Tan, C. Ni, Y. Gu and J. Hu, *Angew. Chem. Int. Ed.*, 2016, **55**, 2743–2747.
6. W. D. Guerra, R. A. Rossi, A. B. Pierini and S. M. Barolo, *J. Org. Chem.*, 2015, **80**, 928–941.
7. H. Hansen, H. Wadepohl and M. Enders, *Chem. Eur. J.*, 2021, **27**, 11084–11093.
8. Z. Li, M. Tang, C. Hu and S. Yu, *Org. Lett.*, 2019, **21**, 8819–8823.
9. PCModel 10.075, K. E. Gilbert, Serena Software, Bloomington IN, 2020.
10. T. A. Halgren, *J. Comput. Chem.*, 1996, **17**, 490–519.
11. T. A. Halgren, *J. Comput. Chem.*, 1996, **17**, 520–552.
12. T. A. Halgren, *J. Comput. Chem.*, 1996, **17**, 553–586.
13. T. A. Halgren and R. B. Nachbar, *J. Comput. Chem.*, 1996, **17**, 587–615.
14. T. A. Halgren, *J. Comput. Chem.*, 1996, **17**, 616–641.
15. Gaussian 16, Revision C.02, M. J. Frisch, G. W. Trucks, H. B. Schlegel, G. E. Scuseria, M. A. Robb, J. R. Cheeseman, G. Scalmani, V. Barone, G. A. Petersson, H. Nakatsuji, X. Li, M. Caricato, A. V. Marenich, J. Bloino, B. G. Janesko, R. Gomperts, B. Mennucci, H. P. Hratchian, J. V. Ortiz, A. F. Izmaylov, J. L. Sonnenberg, D. Williams-Young, F. Ding, F. Lipparini, F. Egidi, J. Goings, B. Peng, A. Petrone, T. Henderson, D. Ranasinghe, V. G. Zakrzewski, J. Gao, N. Rega, G. Zheng, W. Liang, M. Hada, M. Ehara, K. Toyota, R. Fukuda, J. Hasegawa, M. Ishida, T. Nakajima, Y. Honda, O. Kitao, H. Nakai, T. Vreven, K. Throssell, J. A. Montgomery, Jr., J. E. Peralta, F. Ogliaro, M. J. Bearpark, J. J. Heyd, E. N. Brothers, K. N. Kudin, V. N. Staroverov, T. A. Keith, R. Kobayashi, J. Normand, K. Raghavachari, A. P. Rendell, J. C. Burant, S. S. Iyengar, J. Tomasi, M. Cossi, J. M. Millam, M. Klene, C. Adamo, R. Cammi, J. W. Ochterski, R. L. Martin, K. Morokuma, O. Farkas, J. B. Foresman, and D. J. Fox, Gaussian, Inc., Wallingford CT, 2019.
16. A. D. Becke, *J. Chem. Phys.*, 1993, **98**, 5648–5652.
17. A. D. Becke, *J. Chem. Phys.*, 1992, **97**, 9173–9177.
18. P. C. Hariharan and J. A. Pople, *Theor. Chim. Acta*, 1973, **28**, 213–222.
19. M. M. Francl, W. J. Pietro, W. J. Hehre, J. S. Binkley, M. S. Gordon, D. J. DeFrees and J. A. Pople, *J. Chem. Phys.*, 1982, **77**, 3654–3665.
20. C. Lee, W. Yang and R. G. Parr, *Phys. Rev. B*, 1988, **37**, 785–789.
21. Y. Zhao and D. G. Truhlar, *Theor. Chem. Acc.*, 2008, **120**, 215–241.
22. J. P. Perdew, K. Burke and M. Ernzerhof, *Phys. Rev. Lett.*, 1996, **77**, 3865–3868.
23. C. Adamo and V. Barone, *J. Chem. Phys.*, 1999, **110**, 6158–6170.
24. A. Schäfer, H. Horn and R. Ahlrichs, *J. Chem. Phys.*, 1992, **97**, 2571–2577.
25. A. Schäfer, C. Huber and R. Ahlrichs, *J. Chem. Phys.*, 1994, **100**, 5829–5835.
26. F. Weigend and R. Ahlrichs, *Phys. Chem. Chem. Phys.*, 2005, **7**, 3297.
27. F. Weigend, *Phys. Chem. Chem. Phys.*, 2006, **8**, 1057.

28. A. V. Marenich, C. J. Cramer and D. G. Truhlar, *J. Phys. Chem. B*, 2009, **113**, 6378–6396.
29. S. Grimme, S. Ehrlich and L. Goerigk, *J. Comput. Chem.*, 2011, **32**, 1456–1465.
30. H. B. Schlegel, *J. Comput. Chem.*, 1982, **3**, 214–218.
31. K. Fukui, *Acc. Chem. Res.*, 1981, **14**, 363–368.
32. H. P. Hratchian and H. B. Schlegel, *J. Chem. Phys.*, 2004, **120**, 9918–9924.
33. H. P. Hratchian and H. B. Schlegel, *J. Chem. Theory Comput.*, 2005, **1**, 61–69.

---

[1] *Purification of Laboratory Chemicals*, 3rd edition. D. D. Perrin, W. L. F. Armarego, *Pergamon Press*, Oxford, **1988**.
